# Identification of microRNAs Involved in Regeneration of the Secondary Vascular System in *Populus tomentosa* Carr

**DOI:** 10.3389/fpls.2016.00724

**Published:** 2016-05-31

**Authors:** Fang Tang, Hairong Wei, Shutang Zhao, Lijuan Wang, Huanquan Zheng, Mengzhu Lu

**Affiliations:** ^1^State Key Laboratory of Tree Genetics and Breeding, Research Institute of Forestry, Chinese Academy of ForestryBeijing, China; ^2^Key Laboratory of Science and Technology of Bamboo and Rattan of State Forestry Administration, International Centre for Bamboo and RattanBeijing, China; ^3^Co-Innovation Center for Sustainable Forestry in Southern China, Nanjing Forestry UniversityNanjing, China; ^4^School of Forestry Resources and Environmental Science, Michigan Technological UniversityHoughton, MI, USA; ^5^Department of Biology, McGill UniversityMontreal, QC, Canada

**Keywords:** microRNAs, high-throughput sequencing, degradome sequencing, *Populus*, secondary vascular system, regeneration

## Abstract

Wood formation is a complex developmental process primarily controlled by a regulatory transcription network. MicroRNAs (miRNAs) can modulate the expression of target genes involved in plant growth and development by inducing mRNA degradation and translational repression. In this study, we used a model of secondary vascular system regeneration established in *Populus tomentosa* to harvest differentiating xylem tissues over time for high-throughput sequencing of small RNAs. Analysis of the sequencing data identified 209 known and 187 novel miRNAs during this regeneration process. Degradome sequencing analysis was then performed, revealing 157 and 75 genes targeted by 21 known and 30 novel miRNA families, respectively. Gene ontology enrichment of these target genes revealed that the targets of 15 miRNAs were enriched in the auxin signaling pathway, cell differentiation, meristem development, and pattern specification process. The major biological events during regeneration of the secondary vascular system included the sequential stages of vascular cambium initiation, formation, and differentiation stages in sequence. This study provides the basis for further analysis of these miRNAs to gain greater insight into their regulatory roles in wood development in trees.

## Introduction

MicroRNAs (miRNAs) are endogenous ~22 nt non-coding small RNAs generated by the RNaseIII-type enzyme Dicer from hairpin structures that are formed from primary miRNAs (Ambros et al., 2003). After cleavage, miRNAs incorporate into an RNA-induced silencing complex (RISC), where they guide the cleavage or repress the translation of target mRNAs according to approximate base-pairing rules (Jones-Rhoades et al., 2006; Voinnet, 2009) or mediate mRNA degradation by directing rapid deadenylation of mRNAs (Wu et al., 2006; Djuranovic et al., 2012). In plants, miRNAs are master regulators of growth and development, including leaf polarity (Palatnik et al., 2003; Ori et al., 2007), floral identity and flowering time (Aukerman and Sakai, 2003; Zhu and Helliwell, 2011), organ boundaries and polarity (Laufs et al., 2004; Mallory et al., 2004), stress responses (Lu et al., 2008; Li et al., 2011), and wood formation (Demura and Fukuda, 2007). In poplar, miRNAs have been identified in differentiating xylem tissue, and the target genes of these miRNAs include key transcription factors (TFs) and enzymes that play indispensable roles in xylem differentiation and lignocellulosic biosynthesis (Puzey et al., 2012; Lu et al., 2013). For instance, overexpression of ptr-miR397a in *Populus trichocarpa* reduced the expression of its target laccase genes and resulted in a 40% decrease in total laccase activity. This decrease in laccase activity in turn led to a reduction in lignin content in transgenic poplars. However, the levels of monolignol biosynthetic gene transcripts remained unchanged, indicating the specificity of mRNA regulation by this miRNA (Lu et al., 2013). Because plant miRNAs have perfect or near-perfect complementarities with their target genes (Rhoades et al., 2002), identification of miRNAs and their targets could be a cost-effective strategy for elucidating the molecular mechanisms that govern dynamic biological processes such as wood formation in woody plants.

Secondary growth in trees or forest trees, including the radial expansion of stems, occurs as a result of division and differentiation of vascular cambium (VC) cells, which can grow both outward and inward to produce tree phloem and xylem; the latter then develops into mature wood (Du and Groover, 2010). Secondary vascular system (SVS) development results from this event. Although this process is known to be regulated by hormones (Aloni et al., 2006) and certain regulatory TFs (Carbon et al., 2009; Lin et al., 2013), SVS development is currently poorly understood. Therefore, further studies are needed to shed light on the molecular mechanisms underlying cambial activity. A model of regeneration of the SVS from debarked trunk has been developed, and anatomical studies showed that new cambium and phloem are regenerated from differentiating xylem cells that remained on the tree trunk surface after girdling. Consequently, the SVS fully regenerates within 3 weeks. This model was used to identify many differentially expressed genes during the stages of cambium formation and xylem differentiation in *Populus tomentosa* (Du et al., 2006; Wang et al., 2009; Zhang et al., 2011). This experimental system provides an excellent opportunity to identify miRNAs and to explore their regulatory roles in wood formation.

In this study, we combined small RNA and degradome high-throughput sequencing techniques to examine regenerated tissues harvested from debarked *P. tomentosa* trunks across six time points after girdling to detect known and novel miRNAs involved in SVS regeneration. Both these miRNAs and their targets were identified using global transcriptome and degradome data, and their roles in the initiation, formation and differentiation of the SVS were explored in the context of their biological processes involved in plant development.

## Materials and methods

### Plant materials and total RNA extraction

Four-year-old *P. tomentosa* trees growing in a clonal plantation located in Tangshan, Hebei Province, P.R. China, were chosen for debarking experiments. Sixty-five healthy and vigorous trees were initially selected and debarked in the same manner as described previously (Du et al., 2006; Wang et al., 2009). Samples were subsequently collected by scraping regenerating tissues from the entire trunk surface in the morning (between 10:00 and 11:00 a.m.) on the 7, 10, 12, 16, 18, and 21 days after girdling (DAG). These samples were immediately frozen and stored in liquid nitrogen. The samples were simultaneously harvested from 4 different clonal trees. We reviewed the anatomy of at least 3 small pieces (2 mm^3^) of regenerating tissues from different parts on each girdled trunk of these 4 clonal trees to assess their regeneration status. Among these trees, 3 clonal trees at the same developmental stage were chosen, and equal amounts of their regenerated tissues were pooled for extraction of total RNA for sequencing and quantitative real-time PCR (qRT-PCR). Total RNAs were isolated from the pooled samples using a Total RNA Purification Kit (#TRK-1001, LC Sciences) according to the manufacturer's instructions. The quality and purity of the total RNA samples was analyzed using an Agilent 2100 bioanalyzer.

### High-throughput sequencing and miRNA identification

Six independent cDNA libraries of small RNAs were generated from the total RNA samples prepared at each of the 6 regeneration stages and were sequenced using an Illumina Solexa sequencing platform. Among the 35 nt tags from Solexa sequencing, adapters were trimmed and contaminated and low-quality reads were removed to obtain clean reads. Then, clean small RNAs between 18 and 30 nt were counted and aligned with the *Populus trichocarpa* genome (http://www.phytozome.net/poplar.php, V3.0) using SOAP software (Li et al., 2008); the number of mismatched nucleotides allowed was set to“0.” The sequences with perfect matches were subjected to further classification: (I) the small RNA reads were classified into ribosomal RNAs (rRNAs), small cytoplasmic RNAs (scRNAs), small nucleolar RNAs (snoRNAs), small nuclear RNAs (snRNAs), and transfer RNAs (tRNAs); (II) the small RNAs were aligned with the precursor miRNAs (pre-miRNAs) and mature miRNAs in miRBase (Kozomara and Griffiths-Jones, 2011; http://www.mirbase.org/, V18.0) to identify known miRNAs; (III) the small RNA reads were aligned with the repeated associated RNAs; (IV) the small RNA reads were aligned with genomic exons and introns in the *P. trichocarpa* genome in the sense and antisense directions to identify mRNA degradation fragments; and (V) unknown or non-annotated small RNAs. Candidate miRNAs were predicted by examining the hairpin structure, the Dicer cleavage site and the minimum free energy of the non-annotated small RNAs using Mireap software (http://sourceforge.net/projects/mireap/), and the parameters were adjusted to meet the criteria of plant miRNAs as follows: (I) the miRNA reference sequence length range was 20–23 nt; (II) the maximal free energy allowed for a given pre-miRNA was −18 kcal/mol; (III) the maximal space between an miRNA and a corresponding miRNA^*^ was 300 nt; (IV) the minimum number of base pairs between an miRNA and a corresponding miRNA^*^ was 16, with no more than 4 bulges; and (V) the maximal asymmetry of miRNA/miRNA^*^ duplex was 4 bases. In addition to the primary criteria in Mireap, two additional requirements were incorporated into high-throughput sequencing data analysis for miRNA identification (Meyers et al., 2008): (I) the sequences should be represent in both miRNA and miRNA^*^; (II) the candidate miRNA sequences should be unique and be present in more than one of the six independent libraries if the miRNA^*^ was absent. The secondary structures of the pre-miRNAs were predicted using the mfold web server (http://mfold.rna.albany.edu/?q=mfold) with the default parameters. The raw data of small RNA sequencing had been submitted to Sequence Read Archive (SRA, SRP072959).

### Normalization and cluster analysis of miRNAs and target genes

The miRNA reads were normalized to total reads per million (RPM), and the members from a given miRNA family with identical sequences were classified into a unique miRNA. After log_2_ transformation and normalization, the miRNAs with greater than 10 RPM were clustered according to centered correlations via the hierarchical centroid linkage clustering method using Cluster3.0 (de Hoon et al., 2004). We also produced microarray expression data from the above samples using a GeneChip® 3′ IVT Express Kit and a GeneChip® Hybridization, Wash, and Stain Kit (Affymetrix, USA) on Affymetrix GeneChip Instrument System (Chinese Academy of Forestry, China) according to the manufacturer's instructions. The microarray data have been submitted to the Gene Expression Omnibus (GEO, GSE71094). The normalized data were also clustered in the same manner as described for the miRNA data. Heatmaps were constructed and visualized using JavaTreeView (Saldanha, 2004).

### Validation and quantification of miRNAs

Identified miRNAs were validated and quantified via a modified universal reverse transcription PCR protocol designed to specifically amplify mRNAs and miRNAs (Hurteau et al., 2008). The reactions included the enzymatic addition of poly(A) tails using the Poly(A) Tailing Kit (#AM1350, Ambion), and reverse transcription (RT) using an universal poly(T) RT primer using SuperScriptIII reverse transcriptase (#18080-051, Invitrogen). The RT products were diluted 1:20, and 2 μl of each product was used in the amplification of miRNAs for cloning or quantification using a miRNA-specific forward primer and the reverse primer complementary to the above RT primer. The PCR products were cloned into pCR2.1 vectors and sequenced using a BigDye® Terminator v3.1 Cycle Sequencing Kit (#4337455, Invitrogen) to confirm the sequences of novel miRNAs. qRT-PCR was performed using a SYBR® Premix ExTaq Kit (#RR820A, TaKaRa) on LightCycler® 480 Real-Time PCR System (Roche, USA) according to the manufacturer's instructions. As an endogenous control, 5.8S rRNA (AJ006440) was used. Statistical analysis of the data was performed using the −2^−ΔΔCt^ method. The relative expression levels based on qRT-PCR were analyzed via one-way analysis of variance (ANOVA) (SPSS 17.0) for comparisons of multiple means. A *P*-value of 0.05 was considered statistically significant for ANOVA. The primers used for cloning and qRT-PCR are listed in Table S5.

### Target genes obtained via degradome sequencing

The targets of both known and novel miRNAs were experimentally verified via degradome sequencing of *P. tomentosa* mRNA according to a previously published protocol for parallel analysis of RNA ends (PARE; German et al., 2009). Three degradome libraries, labeled as VC initiation stage, VC formation stage and VC differentiation stage, were constructed from the poly(A) tail-containing fraction of total RNA samples pooled at two adjacent time points, i.e., 7 and 10 DAG, 12 and 16 DAG, and 18 and 21 DAG, to identify target genes of miRNAs. The data were then analyzed using the CleaveLand pipeline (Addo-Quaye et al., 2009) to identify cleaved miRNA targets considering the *P. trichocarpa* genome transcripts (V 3.0), pre-miRNA sequences of *Populus* from miRBase (V18.0) and all novel pre-miRNAs found in this study. Based on the signature abundance at each occupied transcript position, the cleaved target transcripts were categorized into five credibility classes (categories 0, 1, 2, 3, and 4) according to the instructions of CleaveLand. The raw data of degradome sequencing had been submitted to Sequence Read Archive (SRA, SRP072959). psRNATarget (http://plantgrn.noble.org/psRNATarget/) was employed to predict the targets of known and novel miRNAs against the transcript sequences of poplar (V3.0). The threshold expectation value was 2.5 (range 0 ~ 5) and the allowed maximum energy to unpair the target site (UPE) was set to 25 (range 0 ~ 100).

### Pearson correlation coefficient analysis between miRNAs and target genes

The pearson correlation coefficients (PCCs) between miRNAs and their target genes were calculated using SPSS software (V 17.0). The normalized expression levels of miRNAs based on the small RNA sequencing data and of target genes based on the microarray expression data at the six time points during SVS regeneration were directly input into the software. A *p*-value below 0.05 was considered statistically significant.

### Gene ontology (GO) enrichment analysis

All target genes with homologous counterparts in *Arabidopsis* were used for GO enrichment analysis using the AgriGO Term Enrichment tool (http://bioinfo.cau.edu.cn/agriGO/). This tool uses the Perl module GO::Term-Finder to identify the enriched GO terms among a list of genes considering all genomic genes as the background. GO terms with a corrected *p* < 0.05 and including a minimum of three genes were considered to be significantly enriched.

## Results

### Regeneration of the SVS in *P. tomentosa*

The plant materials harvested during the regeneration of SVS were examined by microscopy, and the results (Figure S1) revealed similar anatomical features during SVS regeneration processes to those described in previous studies (Du et al., 2006; Wang et al., 2009). Briefly, phloem, the cambium zone and a considerable proportion of immature xylem were peeled off with the bark, and only a few layers of immature xylem cells remained on the surface of the girdled trunk. The formation of calli, accompanied by discontinuous meristem cells, was first observed on the entire surface of the girdled trunk on the 7 DAG. On the 10 DAG, continuous, flat meristem cells were arranged irregularly between the calli and immature xylem. On the 12 DAG, the flat meristem cell layers had increased in number and subsequently formed continuous and regular cell layers similar to a VC on the 16 DAG. The new cambium began to differentiate on the 18 DAG, and numerous xylem cells and phloem cells adaxial and abaxial to the cambium, respectively, were appeared on the 21 DAG. The presence of these cells implied the formation of a normal SVS structure capable of wood formation had been completed within 21 days.

### Identification and characterization of small RNAs during the regeneration of SVS

**Figure 1:**
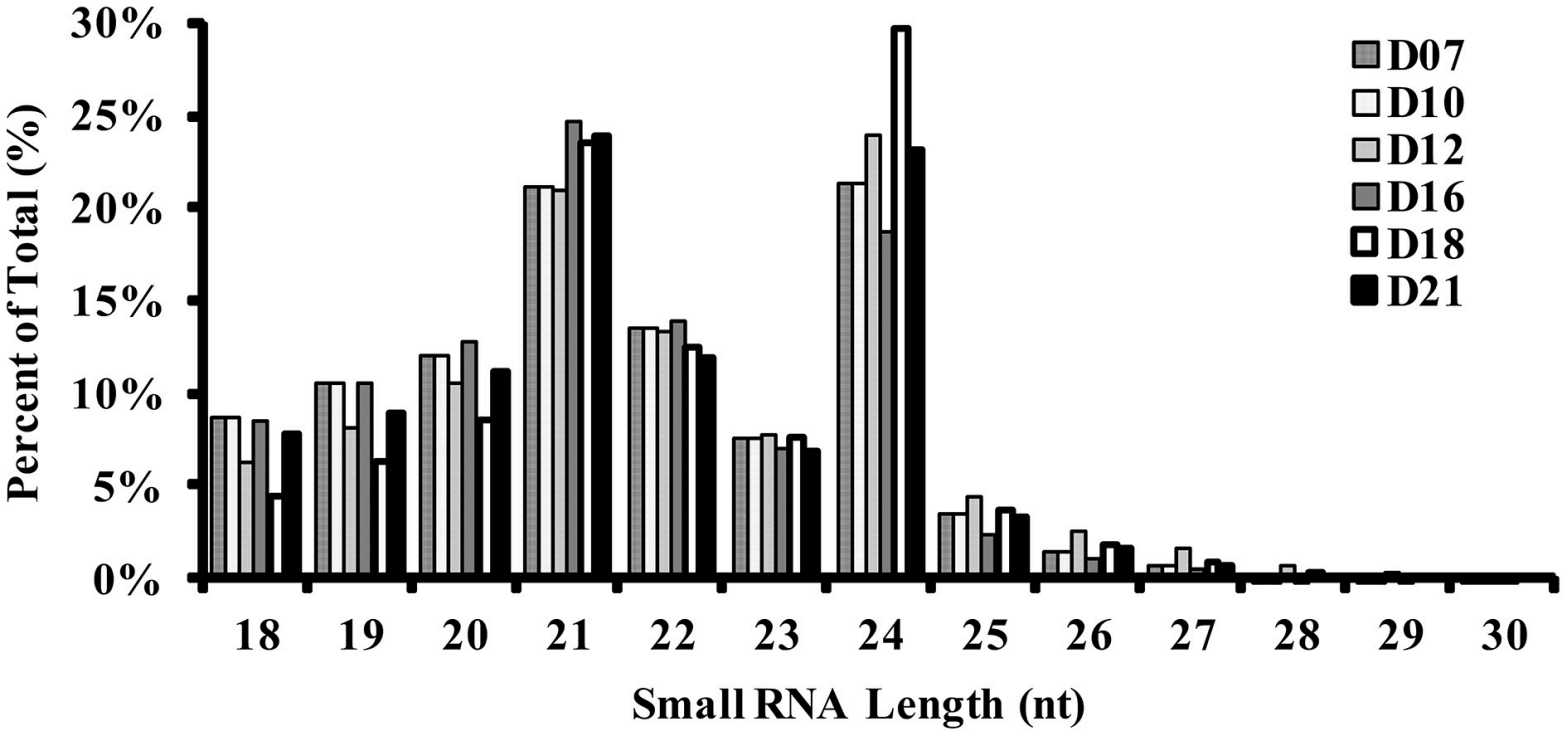
**Length distribution of small RNA reads during SVS regeneration in *P. tomentosa***.

**Figure 2:**
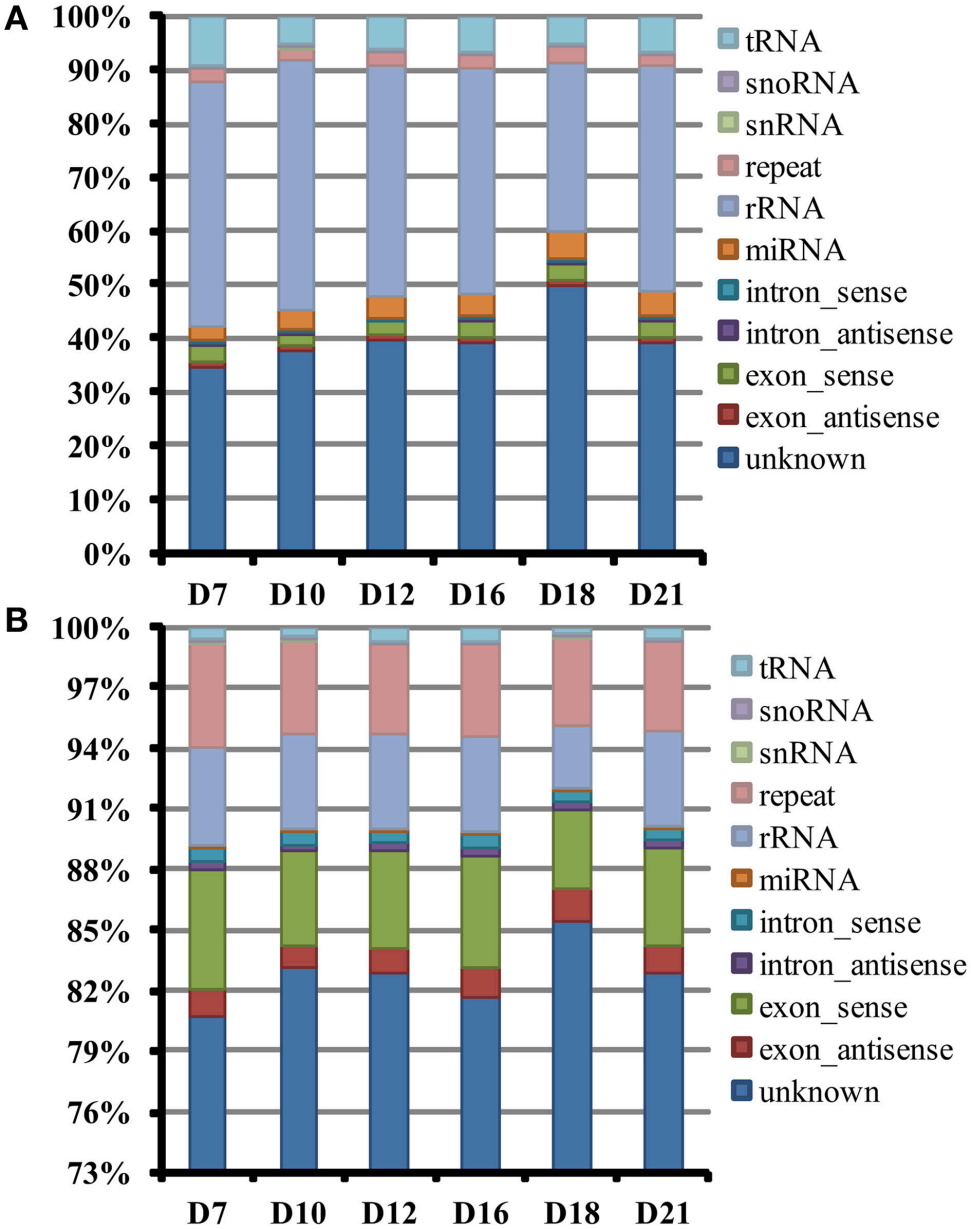
**Summary of sequence categories for six small RNA libraries during SVS regeneration. (A)** Sequence classification of total reads; **(B)** sequence classification of unique reads. tRNA, transfer RNA; snoRNA, small nucleolar RNA; snRNA, small nuclear RNA; repeat, repeat associate RNA; rRNA, ribosomal RNA; miRNA, microRNA.

To identify the known and novel miRNAs involved in SVS regeneration, we performed high-throughput sequencing of small RNA libraries generated from each of the six time points. Subsequently, the adaptors, contaminants, and small reads greater than 18–30 nt in length were removed. Ultimately, 8,212,881, 9,049,855, 9,331,993, 7,832,654, 9,827,614, and 7,347,728 total clean reads were obtained from the samples on the 7, 10, 12, 16, 18, and 21 DAG, respectively (Table S1). Of these reads, 65.77, 62.74, 59.96, 60.67, 52.19, and 61.59%, respectively, could be mapped to the genome of *P. trichocarpa* (V3.0). The length distributions of these clean reads before filtering are shown in Figure 1. Small RNAs of 21 and 24 nt accounted for approximately half of all reads in each library, representing the plurality of small RNAs. The number and the percentage of clean reads were classified and filtered using various RNA databases (Figure 2, Table S1). Among these different RNA classifications, non-annotated small RNAs accounted for the greatest proportion of small RNAs. Specifically, non-annotated small RNAs varied from 35 to 50% of the total reads and from 80 to 85% of the unique reads at the 6 time points of SVS regeneration. This result indicated that many non-annotated small RNAs could be involved in the regeneration process. The percentage of non-annotated small RNAs was higher on the 18 DAG than at any other time point. This result suggested that more small RNAs regulate this important stage of the SVS development process. These non-annotated small RNAs were used for prediction and identification of candidate novel miRNAs.

### Known miRNAs and their expression profiles during SVS regeneration

Among the *P. trichocarpa* sequences in miRBase 18.0, 237 identified miRNAs were detected, and 209 of these previously identified miRNAs were expressed in at least one of the six *P. tomentosa* small RNA libraries (corresponding to the 7, 10, 12, 16, 18, and 21 DAG), which contained 75, 78, 75, 77, 83, and 79 unique known miRNAs, respectively (Table 1, Table S2). In most situations, more than one *P. tomentosa* small RNAs could be completely mapped to a pre-miRNA sequence from miRBase 18.0, thus the sequence with the most reads was defined as the predominant sequence. In some cases, the predominant sequence was not the mature miRNA identified in miRBase 18.0; in particular, we detected 56 unique predominant sequences that were different from mature miRNA sequences (Table S2). The observed differences involved the addition and/or deletion of one or a few nucleotides at either the 5′ or the 3′ end of the miRNA compared with the mature miRNA sequence. This result implied that predominant sequences may arise from different cleavage sites of pre-miRNAs. Furthermore, miRNAs^*^ represent the opposite strand of mature miRNAs and more easily degrade in the nucleus. Therefore, miRNAs^*^are typically detected at a much lower frequency than miRNAs (Bartel, 2004). Among the 209 known miRNAs, 133 miRNAs^*^ were identified, and 41 miRNAs^*^ displayed more sequencing reads than their mature miRNAs in at least one library. This phenomenon also suggested that the strand of an miRNA::miRNA^*^ complex that enters a RISC complex might vary between species (Li et al., 2013).

**Table 1:** **Number of identified known miRNAs in *P. tomentosa* based on high-throughput sequencing**.

**Sample**	**Known miRNAs**	miRNA^*^	miRNA^*^ > miRNA	**Unique miRNAs**	**Difference**
D7	174	88	20	75	44
D10	175	98	21	78	48
D12	171	94	16	75	45
D16	178	100	20	77	47
D18	187	121	33	83	50
D21	193	106	27	79	44
Total	209	133	41	91	56
Consistent among six libraries	157	70	11	65	36

“Known miRNAs,” conserved miRNAs between P. tomentosa and P. trichocarpa; “Total,” number of reads identified in at least one library; “Consistent among six libraries,” number of reads identified during SVS regeneration in all six small RNA libraries; “miRNA^*^ ≥ miRNA,” the number of miRNAs^*^ matched to an equivalent or greater number of sequences than the corresponding miRNA. “Unique miRNAs,” the number of members of a given miRNA family with identical sequences; “Difference,” the number of instances in which the unique miRNA sequence with the most reads was not the mature miRNA in miRBase 18.0.

**Figure 3:**
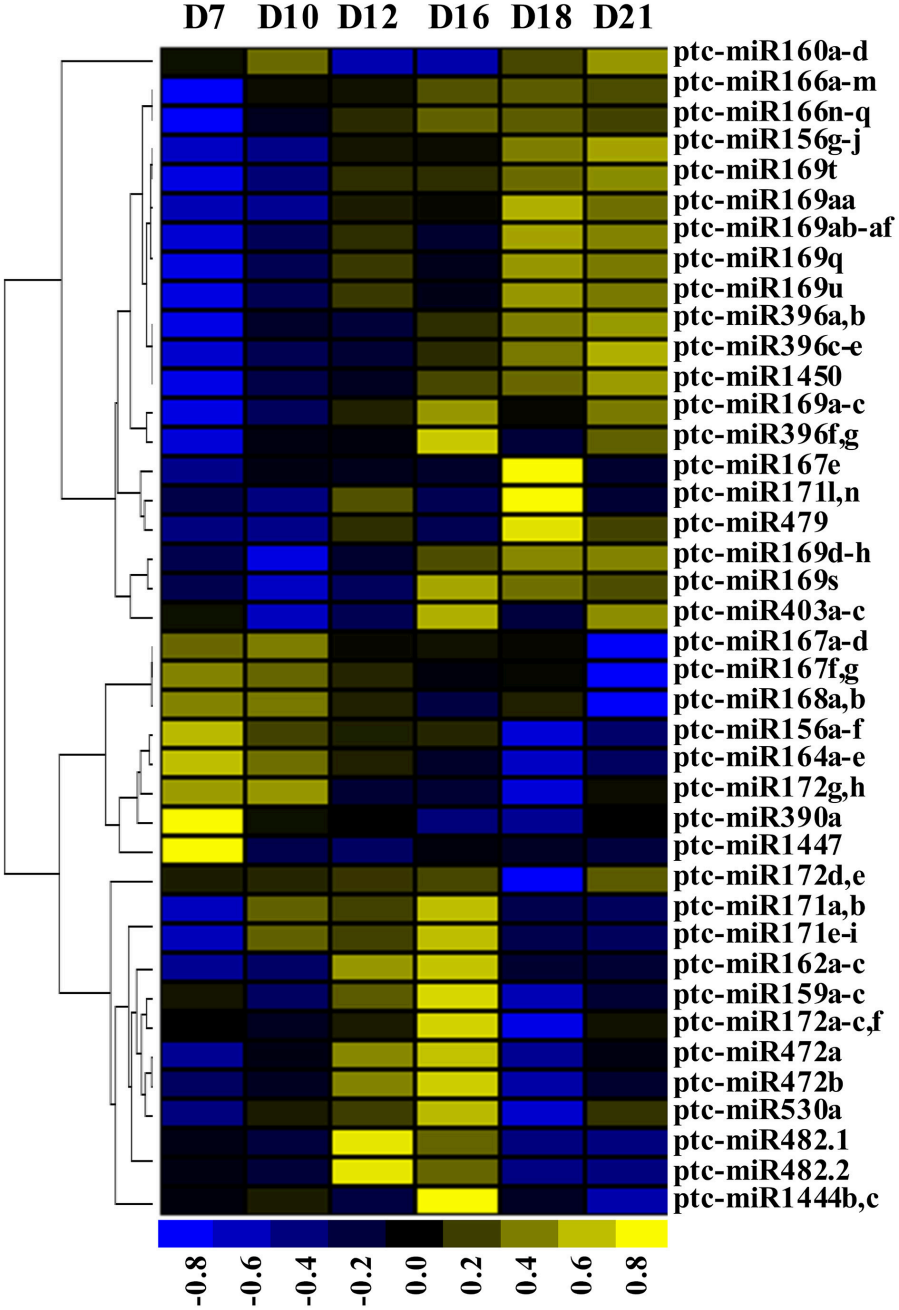
**Heatmap of known miRNAs expressed during SVS regeneration**. The expression levels of unique miRNAs were normalized to total RPM. High (yellow) or low (blue) expression levels were established based on normalized data (color bar under the map) generated using Cluster 3.0 software. D7, D10, D12, D16, D18, and D21 indicate 7, 10, 12, 16, 18, and 21 DAG, respectively.

The expression profiles of the 42 and 18 unique known miRNAs from 20 conserved and 12 non-conserved miRNA families, respectively, notably differed during SVS regeneration (Table S4). The conserved miRNA families generally displayed higher expression than non-conserved miRNA families. For instance, miR156, miR164, miR166, and miR168 accounted for approximately 80% of all known miRNA reads. In contrast, the expression levels of 6 non-conserved miRNA families, i.e., miR1446, miR1448, miR473, miR475, miR477, and miR481, were very low, displaying RPM values were below 10 at all 6 time points. The 40 unique known miRNAs displaying a high RPM (≥10) from 21 miRNA families were normalized and clustered, and the heatmap revealed that these miRNAs were highly expressed during one of the three regeneration stages: the VC initiation stage (between 7 and 10 DAG), the VC formation stage (between 12 and 16 DAG), and the VC differentiation stage (between 18 and 21 DAG; Figure 3). The expression levels of the miR164, miR167, miR168, and miR390 families were high during the VC initiation stage and were decreased thereafter. The expression levels of the miR159, miR162, miR171, miR472, and miR482 families were highest during the VC formation stage, whereas the expression level of miR160 was the lowest during this stage. Alternatively, miR166, miR169, miR396, and miR1450 were most highly expressed during the VC differentiation stage. In addition, different members of a given miRNA family displayed diverse expression patterns in different stages. For example, ptc-miR156a-f showed greatest expression during the VC initiation stage, but ptc-miR156g-j showed greatest expression during the VC differentiation stage. The expression levels of 15 unique known miRNAs were verified by qRT-PCR (Figure S2). Except for certain regeneration time points, the trends in the relative expression levels of most unique known miRNAs obtained via qRT-PCR were consistent with the trends from the sequencing data. These results indicated that the miRNA expression data based on small RNA sequencing were reliable.

### Identification of novel miRNAs and their expression patterns during SVS regeneration

Candidate miRNAs were identified from non-annotated small RNA using Mireap software. Ultimately, a total of 373 individual miRNAs from different genomic locations were initially identified and divided into 247 candidate miRNA families based on sequence similarity (Table S3). Considering the additional criteria for the annotation of novel miRNAs from high-throughput sequencing data analysis (Meyers et al., 2008), from those 373 candidate miRNAs, 187 miRNAs classified into 127 miRNA families were considered with high reliability to be novel miRNAs in *P. tomentosa*. Of these 187 novel miRNAs, 57 miRNAs^*^ were present in at least one of the six small RNA libraries, and 14 miRNAs^*^ displayed more sequencing reads than their mature miRNAs (Table 2).

**Table 2:** **Number of identified candidate and novel miRNAs in *P. tomentosa* based on high-throughput sequencing**.

**Sample**	**Candidate miRNAs**	**Novel miRNA**	**Novel miRNA**	miRNA^*^ families	miRNA^*^ > miRNA
D7	128	106	75	19	6
D10	133	94	72	23	3
D12	137	110	76	24	7
D16	135	112	77	21	2
D18	163	118	87	21	4
D21	130	101	70	21	3
Total	373	187	127	57	14
Consistent among six libraries	37	38	30	7	0

“Candidate miRNA,” candidate new miRNAs in P. tomentosa were identified from non-annotated small RNAs using Mireap software; “Novel miRNA,” novel miRNAs were identified among candidate miRNAs by applying two additional criteria that are required for miRNA identification via high-throughput sequencing data analysis; “Total,” number of reads identified in at least one library; “Consistent among six libraries,” number of reads identified during SVS regeneration in all six small RNA libraries. “miRNA^*^ ≥miRNA,” the number of miRNAs^*^ with more sequences than or an equal number of sequences to the corresponding miRNA.

**Figure 4:**
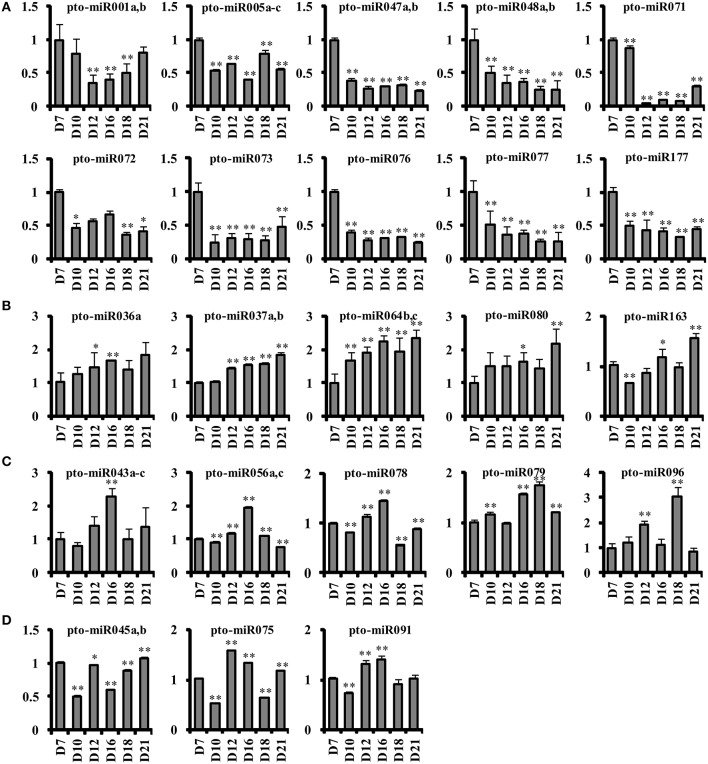
**Relative expression profiles of novel miRNAs during SVS regeneration based on universal qRT-PCR**. The relative expression levels of novel miRNAs that had been cloned or that displayed an RPM above 10 were determined via universal qRT-PCR. **(A)** Ten novel miRNAs displayed higher expression values on the 7 DAG than at any other time point. **(B)** The trends in the expression of 5 novel miRNAs increased gradually during SVS regeneration and peaked on the 21 DAG. **(C)** Five novel miRNAs were most highly expressed on the 16 or 18 DAG. **(D)** Three novel miRNAs were predominantly expressed on the 12 or 16 DAG. D7, D10, D12, D16, D18, and D21 indicate 7, 10, 12, 16, 18, and 21 DAG, respectively. ^**^P ≤ 0.01; ^*^0.01 < *P* ≤ 0.05.

To validate the reliability of the identified novel miRNAs, we randomly cloned the mature sequences of 21 novel miRNAs and 2 known miRNAs as positive controls (Table S6). In total, we obtained 53 cloned sequences for all 21 novel miRNAs, with 2–5 sequences for each miRNA. The cloned sequences of 7 novel miRNAs were consistent with mature miRNA sequences, whereas the remaining 14 novel miRNAs had 1 or 2 cloned sequences that were longer or shorter than the mature miRNA sequence at the 3′ end but that aligned with the pre-miRNA sequence. This observation indicated that the miRNA::miRNA^*^ pair was likely cleaved at several sites, of which one or two were dominant, although cleavage at other sites might occur and play a role in spatio-temporal transcriptional regulation. All pre-miRNA sequences of these cloned miRNAs could form stable stem-loop structures based on analysis using the mfold web server (Figure S3). In general, the expression levels of novel miRNAs were lower than those of known miRNAs during SVS regeneration in *P. tomentosa*. Among the 187 novel miRNAs, only 27 miRNAs from 20 novel miRNA families displayed an RPM above 10 in at least one library, and 40 miRNAs from 28 novel miRNA families were detected as sequencing reads at all six time points during SVS regeneration (Table S4). The expression of these novel miRNAs were generally low based on sequencing data (RPM < 100) and even absent in certain time points, thus it would be inaccurate to compare their expression at each time points during SVS regeneration. Therefore, we randomly selected 23 unique novel miRNAs and detected their expression in all six time points using qRT-PCR (Figure 4). In addition, we found that 4 unique novel miRNAs (pto-miR047, pto-miR071, pto-miR072, and pto-miR076) with RPM over 100 in six time points had the similar expression pattern with that obtained from qRT-PCR.

### Expression of known and novel miRNAs in different tissues of *P. tomentosa*

To verify that these miRNAs were specifically expressed during secondary growth, we performed qRT-PCR of 20 novel miRNAs and 22 known miRNAs on various tissues of 6-month-old *P. tomentosa* plants cultivated in a greenhouse (Figures S4, S5). These tissues included shoot apical meristem (SAM, little vascular tissue), leaf vein, mesophyll, root apical meristem (RAM, little vascular tissue), cambium zone (limited vascular tissue), phloem and xylem. Of the novel miRNAs analyzed, 12 novel miRNAs (60%) were specifically highly expressed in xylem, 4 (20%) were highly expressed in leaf vein, phloem and xylem, and the remaining 4 (20%) were predominantly expressed in RAM (Figure S4). In addition, 8 (36%) of the known miRNAs were highly expressed in xylem, 8 (36%) were especially expressed in leaf vein and mesophyll, and the remaining 6 (28%) were expressed in all tissues except RAM (Figure S5). This result suggested that most of the novel miRNAs detected during SVS regeneration were specific to the development of vascular tissues but that most known miRNAs were involved in diverse biological processes.

### Identification of cleaved miRNa targets by degradome sequencing

To further understand the roles of miRNAs during the SVS regeneration process, degradome sequencing was performed to identify miRNA targets using the same total RNA samples. We acquired 4,689,357, 2,469,256, and 3,454,664 unique sequences from three degradome libraries, and 68, 67, and 50% of these unique reads, respectively, could be mapped to the *P. trichocarpa* transcript (V3.0). The mapped ratio in the third degradome library was lower, suggesting this stage, during which the renewed vascular cambium began to differentiate xylem and phloem cells, might have certain amount of unique genes transcribed that could not be found in the sequenced *p. trichocarpa* wood-forming tissues. The mapped unique sequences were further analyzed using the CleaveLand pipeline to identify the cleaved targets of known and novel miRNAs (Addo-Quaye et al., 2008, 2009). The t-plot lists of predicted miRNA targets compared with the degradome sequences are shown in Figure S6. In this study, we identified a total of 157 target genes that were regulated by 21 known miRNA families (Table 3) and 75 target genes that were regulated by 30 novel miRNA families (Table 4). Most of the identified conserved miRNAs primarily targeted TFs. Therefore, these conserved miRNAs perform specific or dedicated gene-regulating functions during SVS regeneration. The PARE-verified targets of non-conserved miRNAs and novel miRNAs were diverse and included TFs, signal transduction factors and other proteins involved in various biological processes (Table S7).

**Table 3:** **The target genes of known miRNAs as validated by degradome sequencing**.

**miRNA**	**Target gene annotation**	Target gene ID^*^
miR156	SQUAMOSA promoter-binding proteins (SBPs)	potri.011G055900(2), potri.012G100700(4), potri.001G058600(0), potri.003G169400(0), potri.011G116800(1), potri.001G055900(4), potri.010G154300(4), potri.015G098900(4), potri.015G060400(4), potri.002G142400(4), potri.014G057800(4), potri.007G138800(0), potri.008G097900(4)
	Isocitrate dehydrogenase 1	potri.005G099600(4)
miR159	MYB	potri.001G036000(0), potri.003G189700(0), potri.001G224500(2), potri.009G018700(3)
miR160	Auxin response factor	potri.004G211700(0), potri.009G014800(0), potri.006G127500(0), potri.008G039000(0), potri.010G223200(0), potri.016G090300(0), potri.002G089900(0), potri.005G171300(0)
miR162	Dicer-like 1	potri.002G181400(0)
miR164	NAC domain-containing protein	potri.005G098200(0), potri.007G065400(0), potri.012G001400(0), potri.015G020000(0), potri.017G086200(0)
	UDP-XYL synthase 6	potri.010G207200(4)
miR166	Homeobox-leucine zipper family protein	potri.001G372300(0), potri.001G188800(2), potri.003G050100(2), potri.006G237500(2), potri.018G045100(0), potri.009G014500(2), potri.004G211300(4)
miR167	Auxin response factor	potri.004G078200(0), potri.017G141000(0)
	SBP-like protein	potri.014G114300(4)
miR169	NF-YA Family	potri.006G145100(0), potri.001G257600(0), potri.001G266000(2), potri.009G052900(2), potri.009G060600(0)
miR171	GRAS family transcription factor	potri.002G144200(2), potri.002G144700(2), potri.014G060200(2), potri.014G060500(2), potri.001G122800(0),
miR172	Related to AP2.7	potri.006G132400(2), potri.008G045300(0), potri.010G216200(0), potri.016G084500(0)
	AP2	potri.005G140700(0), potri.007G046200(2)
miR319	TCP family transcription factor	potri.004G065800(0), potri.011G096600(4), potri.013G119400(4)
miR393	Auxin signaling F-box 2	potri.001G323100(0), potri.017G061600(0)
	TIR1 protein	potri.002G207800(0), potri.014G134800(2)
	bHLH	potri.002G235400(2), potri.014G148900(2)
miR394	Galactose oxidase/kelch repeat protein	potri.001G057100(0), potri.003G171300(0)
miR396	Growth-regulating factor	potri.001G114000(0), potri.001G132600(2), potri.002G115100(0), potri.003G100800(0), potri.007G007100(0), potri.013G077500(0), potri.014G007200(0), potri.014G012800(1), potri.014G071800(0), potri.015G006200(4), potri.006G143200(4), potri.001G082700(4)
miR397	Laccase	potri.006G094100(4), potri.008G073700(3), potri.010G183600(2)
miR472	NB-ARC domain disease resistance protein	potri.001G028700(0), potri.001G363400(0), potri.003G200800(3), potri.011G009400(0), potri.019G002800(2), potri.T012900(4), potri.T013200(0), potri.T024900(0), potri.T025300(2), potri.T025800(0), potri.T025900(0), potri.T026200(0), potri.T026800(0), potri.T026900(0), potri.T027200(0), potri.T027500(0), potri.T028700(0), potri.T029000(0), potri.T039900(0), potri.T044500(0), potri.T052000(0)
	ATP binding	potri.011G008600(2), potri.011G060600(2), potri.T037600(0), potri.T037900(0),potri.T038300(0), potri.T039300(2), potri.T112200(2)
miR475	Pentatricopeptide repeat (PPR) protein	potri.006G242200(0), potri.006G257300(0), potri.006G271200(0), potri.011G057900(4), potri.006G271400(0), potri.013G034400(0), potri.019G021200(0)
miR482	NB-ARC domain disease resistance protein	potri.001G420000(2), potri.001G435100(2), potri.001G426500(4) potri.001G428100(2), potri.001G443700(2), potri.001G444100(0), potri.001G445000(2), potri.001G445900(2), potri.001G445100(4), potri.T087900(4), potri.T162100(4), potri.T176000(4)
	SERK1	potri.005G083300(4)
	AUX1	potri.008G066400(2), potri.010G191000(2)
	CAD4	potri.009G095800(2)
	Translation initiation factor 3B1	potri.018G015200(2)
	AFG1-like ATPase family protein	potri.006G252300(4)
	LUC7 N-terminal domain-containing protein	potri.001G124300(4)
	Unknown	potri.015G115500(2)
miR530	Zinc knuckle (CCHC-type) family protein	potri.008G162500(0), potri.010G076700(2)
	bHLH	potri.014G099700(0)
	Unknown	potri.006G180500(0), potri.018G102800(0), potri.010G062000(4)
miR1447	Ankyrin repeat family protein	potri.019G105700(0), potri.019G105900(0), potri.019G106000(0), potri.019G107700(0), potri.019G107800(0), potri.019G108000(0), potri.019G108200(0)
miR1450	Unknown protein	potri.004G215900(3), potri.006G030400(4)

* *The number in brackets represents the five classes (with 0 as the highest degradome peak) in which the cleaved target transcripts were categorized based on their signature abundance at the each occupied transcript position*.

**Table 4:** **The target genes of novel miRNAs as validated by degradome sequencing**.

**miRNA**	**Target gene annotation**	Target gene ID^*^
pto-miR001	XTH9	potri.019G125000(2)
	Cycloartenol synthase 1 (CAS1)	potri.006G079300(2)
pto-miR005	Endo-beta-mannanase 7 (MAN7)	potri.005G120500(4), potri.007G022000(4)
	Unknown protein	potri.008G203800(4)
pto-miR009	Unknown protein	potri.008G076300(4), potri.013G144000(4)
pto-miR011	Unknown protein	potri.001G077200(4), potri.T154400(4)
pto-miR016	NB-ARC domain disease resistance protein	potri.014G007900(4), potri.004G169800(4), potri.004G170200(4), potri.004G170700(4), potri.014G002200(4), potri.014G002300(4), potri.014G009300(4), potri.014G009600(4), potri.014G010900(4), potri.014G012000(4)
pto-miR017	Protein phosphatase 2A-3	potri.003G217900(4)
pto-miR030	TCTP	potri.008G221200(2), potri.008G226500(2)
pto-miR037	MAPR3	potri.015G125100(2)
	Expansin A4	potri.001G240900(2)
pto-miR040	Galactose oxidase/kelch repeat protein	potri.001G331500(4)
pto-miR042	Succinate dehydrogenase 1-1	potri.007G026400(2)
pto-miR043	NRB4	potri.003G013300(4), potri.003G029100(4)
pto-miR047	Ubiquitin-conjugating enzyme 2	potri.015G064000(4)
pto-miR056	NAC transcription factor	potri.002G181900(0), potri.002G182400(0), potri.013G079700(0), potri.014G071000(0), potri.014G108100(0)
pto-miR070	BON3	potri.013G029100(1)
pto-miR073	NB-ARC domain disease resistance protein	potri.005G237900(4), potri.017G143500(4), potri.017G143600(4), potri.017G144400(4), potri.T060300(4), potri.T060600(4), potri.T060400(4)
	SNF7 family protein	potri.018G069900(4)
pto-miR080	Chaperone DnaJ-domain superfamily protein	potri.005G181700(2)
pto-miR081	Auxin response factor 2	potri.012G106100(4)
pto-miR084	Lysophosphatidyl acyltransferase 2	potri.006G055000(4)
pto-miR087	Squamosa promoter-binding protein	potri.002G188700(4)
pto-miR091	Extensin 3	potri.002G070100(3)
	Tubby like protein 3	potri.008G195200(2)
pto-miR092	UDP-glycosyltransferase superfamily protein	potri.001G179000(4)
pto-miR095	Exocyst complex component SEC5	potri.005G191500(4)
pto-miR105	NB-ARC domain disease resistance protein	potri.019G022800(2), potri.T013600(4), potri.T015900(4), potri.T024700(2), potri.T024900(2), potri.T025500(2), potri.T025800(4), potri.T025900(4), potri.T026800(2), potri.T028100(4), potri.T029000(2), potri.T052000(2), potri.T053000(2)
pto-miR107	Ubiquitin-specific protease 7	potri.001G197400(4)
pto-miR143	NB-ARC domain disease resistance protein	potri.014G001900(4)
pto-miR154	Squamosa promoter binding protein	potri.001G058600(3), potri.003G169400(2), potri.010G154300(0), potri.011G055900(1), potri.018G149900(2)
	Casein kinase I-like 6	potri.005G226900(2)
pto-miR163	Cellulose synthase 3 (CESA3)	potri.016G054900(4)
pto-miR168	Copper/zinc superoxide dismutase 1	potri.005G044400(4)
pto-miR177	Auxin influx transporter	potri.016G113600(2)
pto-miR183	Kinase interacting (KIP1-like) family protein	potri.002G049600(4)
	ARM repeat superfamily protein	potri.008G225500(2), potri.008G225800(2)

* *The same as Table 2*.

However, four highly expressed miRNAs, i.e., miR168, miR390, miR403, and miR479, did not have targets in any degradome library, and a subset of novel miRNAs did not have any PARE-verified targets. To determine whether these miRNAs regulate their target genes via translational repression rather than mRNA degradation, we further predicted the targets of all miRNAs using the psRNATarget web server (Table S8). Among 232 degradome sequencing-validated miRNAs and their target pairs, 158 pairs were present in the psRNATarget results. The miRNAs that had no PARE-verified targets corresponded to predicted target genes in the psRNATarget results, and the inhibition mode was translational repression besides cleavage. However, the degradome analysis pipeline and psRNATarget use distinct algorithms to predict target genes. Degradome analysis focused on the enrichment of target cleavage sequences from high-throughput sequencing, whereas psRNATarget used a bioinformatics algorithm to predict miRNA targets similar to the calculation of sequence similarity and energy. Therefore, the target genes validated via degradome analysis are more reliable even though target genes regulated by translational repression could not be detected.

### Pre-miRNAs could be cleaved by their own miRNAs or other miRNAs

**Figure 5:**
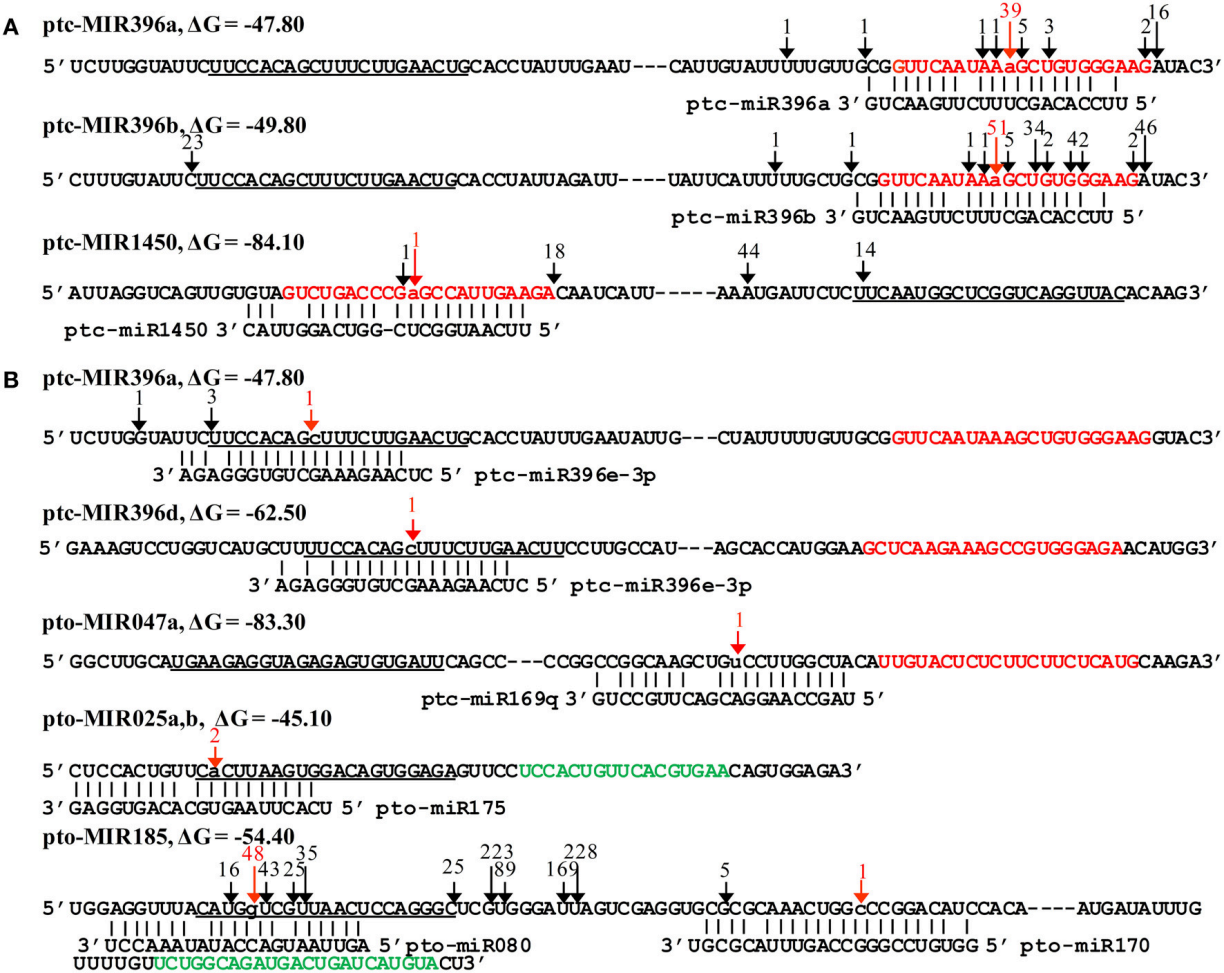
**Certain pre-miRNAs had signatures corresponding to the cleavage sites of their own and other mature miRNAs. (A)** The pre-miRNAs cleaved by their own mature miRNAs. The precursors of ptc-miR396a, ptc-miR396b, and ptc-miR1450 were cleaved by their own mature mRNAs at position 10 of the miRNA^*^ region. **(B)** The pre-miRNAs cleaved by other mature miRNAs. The precursors of ptc-miR396a and ptc-miR396d were cleaved by ptc-miR396e-3p at the cleavage sites of mature miRNAs; the precursors of pto-miR047a and pto-miR185 were targeted by ptc-miR169q and pto-miR170, respectively, at cleavage sites in the loop-forming region; and the precursors of pto-miR025a, b and pto-miR185 were targeted by pto-miR175 and pto-miR080, respectively, at cleavage sites located near the 5′ end of the mature miRNAs. The mature miRNAs are underlined. The miRNAs^*^ are presented in red, and the complementary regions of mature miRNAs are shown in green. The lowercase letters and red arrows indicate the cleavage sites of miRNAs.

Previous studies have found that several pre-miRNAs can be targeted by their own mature miRNAs based on high-throughput degradome sequencing (German et al., 2008; Meng et al., 2010). In this study, we considered all precursors of known and novel miRNAs as target sequences to detect possible cleavage sites. Three pre-miRNAs were found to be targeted by their own mature miRNAs, and five pre-miRNAs appeared to be cleaved by other mature miRNAs (Table S9). First, the precursors of ptc-miR396a, ptc-miR396b and ptc-miR1450 were self-regulated by their own mature miRNAs; the cleavage sites were in the middle of the miRNA^*^ coding regions, corresponding to position 10 of their mature miRNAs (Figure 5A). Second, ptc-miR396e^*^ could cleave the precursors of ptc-miR396a and ptc-miR396d in the middle site of their mature miRNAs. This finding suggested that one member of a miRNA family can regulate other members of the same family. Finally, ptc-miR169q and pto-miR170 targeted the precursors of pto-miR047a and pto-miR207, respectively, and pto-MIR185 was the target of pto-miR080 and pto-miR175. Their cleavage sites were located at the 5′ end of mature miRNAs or in the loop-forming region of pre-miRNAs (Figure 5B). These results provided the first evidence that some pre-miRNAs in poplar can be regulated by their own or other mature miRNAs via self-cleavage or destruction of the miRNA::miRNA^*^ structure, although these regulatory cleavage events may not be detectable in all degradome sequencing libraries (Table S9). These finding indicated that miRNA- or miRNA^*^-mediated cleavage of certain pre-miRNAs could be spatio-temporally regulated.

### Transcriptome profiles of miRNAs and their target genes during SVS regeneration

**Figure 6:**
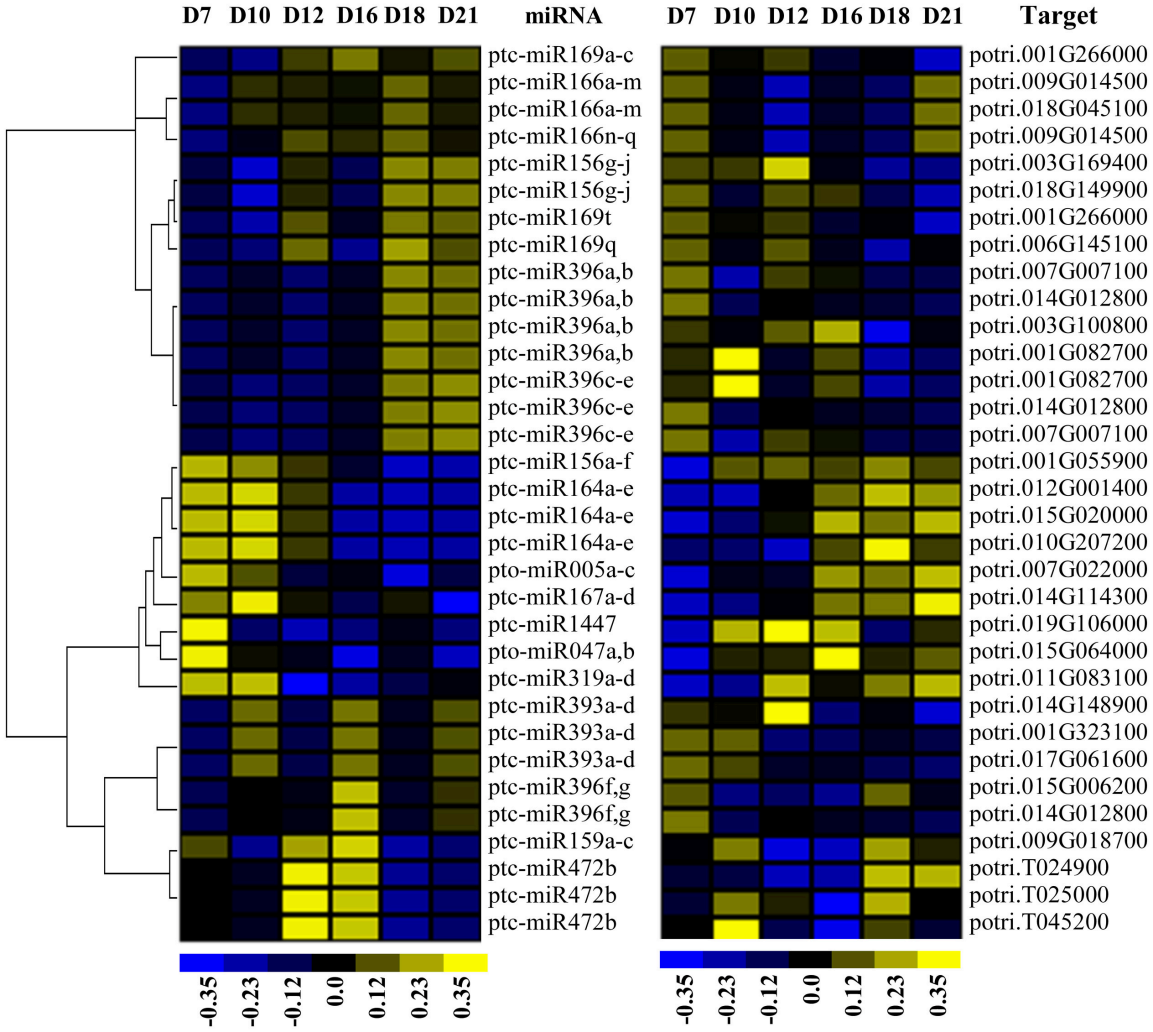
**Heatmap showing the expression of the miRNAs and their target genes exhibited a significantly negative correlation during SVS regeneration in *P. tomentosa***. The expression levels of miRNAs (left side) were calculated based on small RNA sequencing at six time points during SVS regeneration. The miRNA targets (right side) were validated by degradome sequencing, and their expression levels were determined using Affymetrix GeneChip analysis. High (yellow) or low (blue) expression levels were established based on normalized data (color bar under the map) generated using Cluster 3.0 software.

To study the correlation between miRNAs and their target genes, we obtained the expression profiles of 223 miRNA::target pairs at six time points during SVS regeneration (Table S10). Among these pairs, the expression levels of 33 miRNA::target pairs were significantly negatively correlated (Figure 6), and those of 9 pairs significantly positively correlated (Table S10). Eight significantly negatively correlated miRNA::target pairs were validated via qRT-PCR (Figure S7), and their expression levels generally displayed an opposing trend. In this study, few miRNAs and target pairs displayed negatively correlated expression profiles. Thus, other miRNA::target pairs may exhibit diverse expression patterns, and their mutual regulation may extend beyond opposing changes in expression, as indicated by previous research (Lopez-Gomollon et al., 2012). Using the expression levels of samples collected on the 7 DAG as the baseline, the expression levels of miRNAs and their targets at the other five regeneration stages were compared with those on the 7 DAG. We measured the changes in the expression of 223 miRNAs and their target genes and calculated PCCs for their gene expression levels (Table S11). The PCCs of these five groups of comparative data ranged between −0.313 and −0.196, which corresponded to weak negative correlations (Figure S8). In addition, some data showed positive correlations or no correlation. Taken together, these results indicated that miRNAs and their target genes are regulated by complex networks during SVS regeneration.

### miRNAs involved in the regulation of the biological processes leading to SVS regeneration

To further understand the roles of miRNA targets in SVS regeneration in *P. tomentosa*, we implemented GO enrichment analysis of the 232 target genes of both known and novel miRNAs. Among these, 221 targets could be annotated according to homologous sequences in *Arabidopsis*. These genes were used for GO enrichment analysis using the AgriGO Term Enrichment tool (Du et al., 2010), and the significantly enriched biological processes included cellular process (80.70%), metabolic process (71.93%), biological regulation (61.40%), developmental process (45.61%), response to stimulus (54.39%), and reproductive process (25.44%; Table S12). The primary molecular function of these genes was binding; in particular, 39 genes (34.21%) showed DNA binding activity, and 36 genes (31.58%) had transcription regulator activity.

**Figure 7:**
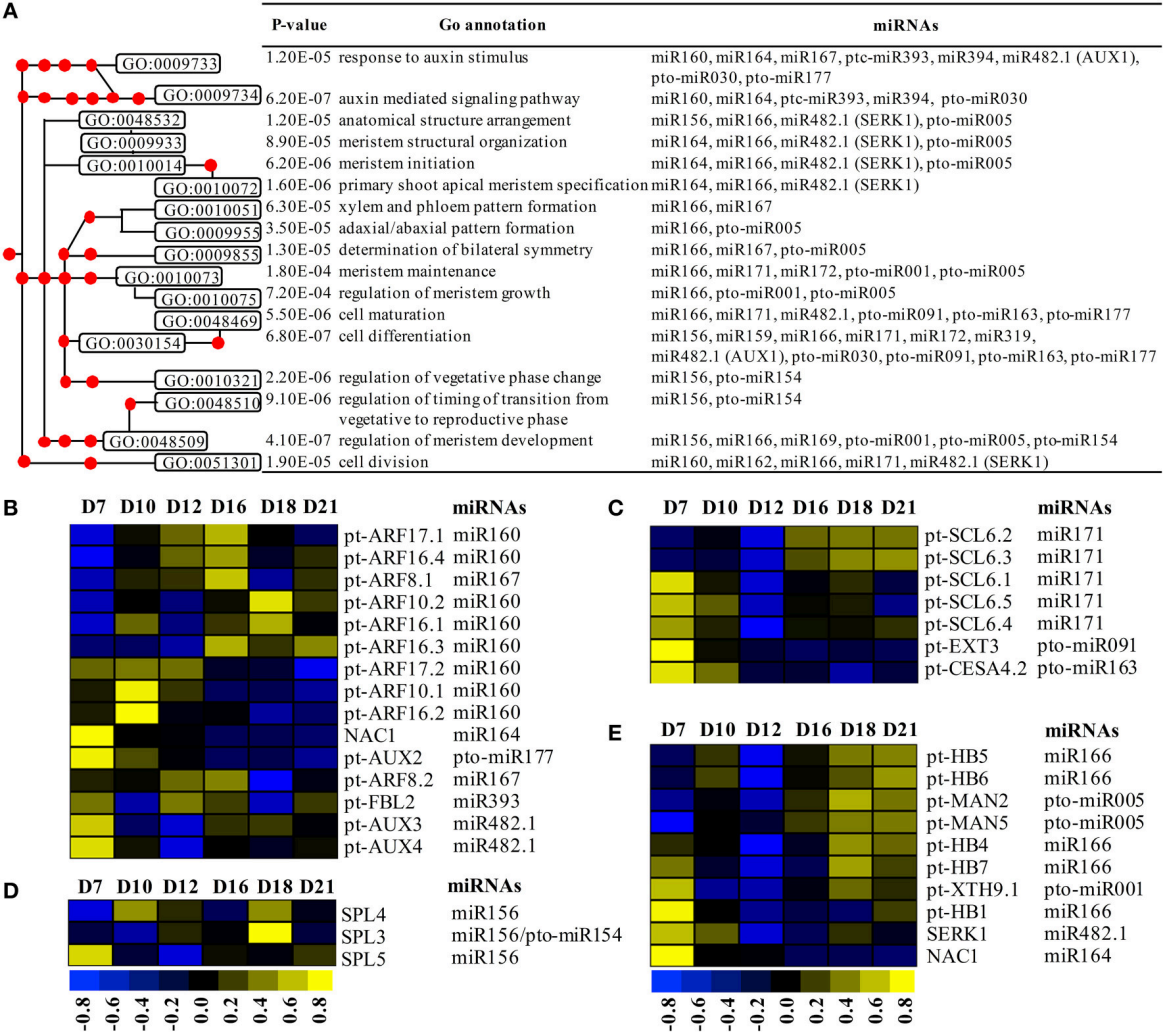
**GO enrichment analysis and the expression profile of enriched target genes**. **(A)** The GO enrichment tree based on biological processes related to the auxin pathway and developmental processes during SVS regeneration in *P. tomentosa*. **(B)** Six miRNAs and their target genes were involved in auxin stimulus and auxin signaling pathway. **(C)** Three miRNAs and their target genes participated in cell differentiation and maturation. **(D)** miR156 and pto-miR154 target three SPL genes that can regulate changes from the vegetative phase. **(E)** Five miRNAs and their target genes contributed to meristem development and pattern specification. Each red dot in A represents one GO node from the GO enrichment tree. The heatmaps in **(B–E)** show the expression profiles of target genes based on microarray data at 6 time points (7, 10, 12, 16, 18, and 21 DAG) during SVS regeneration.

We identified 9 known miRNAs and 6 novel miRNAs whose target genes were involved in the formation of secondary vascular tissue. These target genes were primarily enriched in the GO categories of auxin signaling pathway, cell differentiation, meristem development and pattern specification processes (Figure 7A). The expression levels of these target genes were also clustered (Figures 7B–E) based on the microarray data from the 6 time points of SVS regeneration (Table S10).

The target genes of miR160, miR167, miR393, miR482.1, and pto-miR177, which play important roles in auxin stimulus (GO:0009733) and auxin-mediated signaling pathway (GO:0009734), displayed different expression patterns during SVS regeneration (Figure 7A). Three auxin influx carrier genes, *pt-AUX2, pt-AUX3*, and *pt-AUX4*, displayed high expression levels during the VC initiation stage. In addition, *pt-FBL2*, the homolog of *Arabidopsis TIR1*, was dynamically expressed from the VC initiation stage to the VC formation stage. Additionally, *pt-ARF10.1, pt-ARF16.2*, and *pt-ARF17.2*, which were targeted by miR160, were highly expressed during the VC initiation stage. The remaining 5 *ARF* genes were primarily expressed during the VC formation or differentiation stage (Figure 7B).

The target genes of 6 miRNAs, miR166, miR171, miR482.1 (AUX1), pto-miR091, pto-miR163, and pto-miR177, were involved in cell differentiation (GO:0030154) and cell maturation (GO:0048469; Figure 7A). Among these target genes, *pt-EXT3* and *pt-CESA4.2*, which were targeted by pto-miR091 and pto-miR163, respectively, were highly expressed during the VC initiation stage. In addition, five GRAS TF genes, homologous to the *Arabidopsis SCL6* gene, were targeted by miR171. Specifically, *pt-SCL6.1, pt-SCL6.4*, and *pt-SCL6.5* displayed high expression on the 7 DAG, whereas *pt-SCL6.2* and *pt-SCL6.3* were highly expressed from 16 to 21 DAG (Figure 7C). Furthermore, the target genes of miR156 and pto-miR154 belonging to the *SPL* gene family were involved in regulation of the transition from the vegetative phase to the reproductive phase in *Arabidopsis*. These target genes were highly and specifically expressed on the 7, 10, and 18 DAG (Figure 7D); this observation indicated that these genes might be involved in the transitions of SVS developmental stages.

The target genes of miR164, miR166, miR482.1 (SERK1), pto-miR001 and pto-miR005 were enriched in several biological processes of meristem development and pattern specification (Figure 7A). Specifically, *pt-HB4*-*pt-HB7* displayed high expression during the VC differentiation stage, whereas *pt-HB1* was highly expressed on the 7 DAG. Moreover, *pt-XTH9.1*, the target gene of the newly identified pto-miR001, was highly expressed during the VC initiation and differentiation stages. In addition, *pt-MAN2* and *pt-MAN5*, homologs of *Arabidopsis MAN7*, showed high expression levels from 16 to 21 DAG, when cell expansion and differentiation occur (Figure 7E).

## Discussion

### Additional novel miRNAs were identified but were weekly expressed during SVS regeneration

The SVS regeneration model can mimic the entire process of wood formation in 1 month. Therefore, it has been used to investigate the genes involved in the processes of cambium initiation, formation and differentiation into xylem and phloem using proteomic and microarray approaches (Du et al., 2006; Wang et al., 2009; Zhang et al., 2011). Most identified genes were downstream genes that were directly involved in metabolism and structural development, and only a few master regulators were identified. Therefore, the SVS regeneration model allowed us to investigate the properties of miRNAs during dynamic and complex developmental processes. By monitoring multiple time points of this dynamic developmental process, we obtained more novel miRNAs than other studies of different tissues and organs at a specific developmental stage or under a certain treatment condition (Li et al., 2010, 2013; Pantaleo et al., 2010; Puzey et al., 2012). As expected, we have identified 209 known and 187 novel miRNAs displaying variable expression patterns during SVS regeneration. Parts of miRNAs had been verified via clone and degradome sequencing. These miRNAs could potentially regulate their target genes in a spatio-temporal manner to modulate gene expression profiles to meet the needs of the plant at each stage of development. From this perspective, the detection of novel and non-conserved known miRNAs displaying lower expression levels might not necessarily indicate that they performed a limited regulatory function during SVS regeneration. Instead, these miRNAs could be inducible or be expressed in a tissue- or even cell-specific manner. Under such circumstances, they would not easily be captured at the time points that we examined. Overall, this study has opened the door to the investigation of the regulatory network governing SVS regeneration.

### miRNAs can be regulated by themselves and other miRNAs

Although in some cases the cleavage sites of self-regulated miRNAs did not precisely correspond to position 10 or 11, cleavage signals surrounding the middle of the miRNA or miRNA^*^ region were detected on several pre-miRNAs of both *Arabidopsis* and rice, such as ath-MIR172, ath-MIR161 and osa-MIR455d (German et al., 2008; Meng et al., 2010). Similarly, the precursors of ptc-miR396a and ptc-miR396b possessed several cleavage sites in the miRNA^*^ region, and the most abundant degradome signatures were detected in the middle of their miRNAs^*^ in this study. These results indicated that their miRNAs^*^ are susceptible to cleavage and that they may be primarily cleaved by their own mature miRNAs. In addition, 3 novel pre-miRNAs were targeted by other miRNAs, and their cleavage sites were located at the 5′ end of their mature miRNAs or in the loop-forming region, which could influence the formation of miRNA::miRNA^*^ structures. There could exist a negative regulation circuit that excess pre-miRNAs are degraded upon self- or other miRNA (miRNA^*^) mediated cleavage because more pre-miRNAs lead to the accumulation of miRNA or miRNA^*^ (Meng et al., 2010). The findings of this study confirmed that the regulation of miRNAs by themselves or other miRNAs is a common phenomenon that may serve as negative feedback to balance the abundances of miRNAs and their targets.

### The correlations in expression between miRNAs and their targets are not always negative

We compared the expression profiles of 223 miRNAs and their target genes and found that only 33 miRNA::target pairs displayed significantly negative correlations; in contrast, 9 pairs displayed significantly positive correlations. Furthermore, certain miRNAs, such as miR160, miR393, and miR482, targeted TF families that contain multiple members that exhibit different expression patterns during SVS regeneration. Alternatively, other miRNA families, such as miR156, miR169, and miR171, contained multiple members that exhibited distinct expression patterns but that targeted the same gene families, whose expression levels often appeared to be irrelevant to the changes in the expression levels of their corresponding miRNAs. Although our present knowledge cannot explicitly explain this observation, several factors could be responsible for this result. First, miRNAs regulate target genes at the RNA level, but the mRNA abundance of targets is determined by multiple factors that act at many steps during transcriptional and post-transcriptional regulation (Li et al., 2007; Blencowe et al., 2009). Second, miRNAs do not exclusively regulate their targets via degradation; they can also induce translational repression of their target mRNAs by base-pairing with their targets (Gu and Kay, 2010). In this study, in addition to degradome sequencing, we predicted the targets of all miRNAs using psRNATarget, which employs a bioinformatics algorithm to predict miRNA targets. Based on these results, certain miRNAs can regulate a subset of their predicted target genes via mRNA degradation while also inhibiting other target genes via translational repression. Third, pre-miRNAs can be cleaved by their own or other mature miRNAs, and such cleavage can reduce the abundance of the miRNA. Thus, the cleavage of pre-miRNAs may not occur at the same rate as the degradation of their target genes. In brief, miRNAs may regulate the expression levels of target genes in a more complex manner and may be constrained by the entire gene regulatory network. Considering such a circumstance, we cannot always anticipate the detection of an inverse correlation between a miRNA and its target gene(s) by analyzing their correlation within the network in which they are embedded. This situation may be pronounced in transitional phases of biological processes such as regeneration of the SVS, during which gene expression dynamically changes.

### miRNAs regulate SVS regeneration partially via auxin transport and signaling

Auxin is involved in the establishment and maintenance of the VC and the formation and organization of phloem and xylem (Sundberg et al., 2000). The finding that *pt-AUX2, pt-AUX3*, and *pt-AUX4* exhibited high expression levels during the VC initiation stage indicating that they play a role in the initiation of cambium formation. TIR1 is known to promote the ubiquitination and subsequent degradation of AUX/IAAs, which release ARF proteins to trigger the expression of downstream auxin signaling genes (Dharmasiri et al., 2005; Tan et al., 2007; Villalobos et al., 2012). Furthermore, *pt-FBL2* was expressed from the VC initiation stage to the VC formation stage, and the 10 ARF TFs displayed high expression levels at different stages of SVS regeneration process. These findings indicated that these genes could coordinate in the auxin-mediated signaling pathway during SVS regeneration.

In *Arabidopsis*, NAC1 is induced by auxin and acts downstream of TIR1 to mediate auxin signaling, thereby promoting lateral root development (Xie et al., 2000). Moreover, auxin-induced increase of miR164 could direct the cleavage of *NAC1* transcripts to down-regulate auxin signals (Guo et al., 2005). The transcripts of *NAC1* homologs were enriched in the biological processes of meristem initiation, specification and arrangement and were highly expressed during the VC initiation stage. Therefore, *NAC1* might regulate the initiation and formation of meristematic cells together with hormone signaling pathways.

### miRNAs mediated cell and tissue differentiation during SVS regeneration

SVS regeneration is accomplished via the emergence of meristem cells from the immature xylem and the further division and expansion of these cells, as well as primary and secondary cell wall formation. In this study, *pt-EXT3* displayed high expression levels during the VC initiation stage; this result suggested that *pt-EXT3* plays a role in regulating the transition from immature xylem cells into meristem cells during the initial stage of SVS regeneration. Additionally, *pt-CESA4.2*, the homolog of *Arabidopsis CesA3*, which is required to form the primary cell wall (Desprez et al., 2007), was highly expressed during the VC initiation stage; this observation implied that *pt-CESA4.2* functions only in the accumulation of cellulose in cell walls of newly formed meristem cells. *SCL6* has been shown to be expressed in the peripheral zone and in vascular tissues of the SAM and has been found to promote the renewal of meristematic cells and the differentiation of the axillary meristem (Schulze et al., 2010; Curaba et al., 2013). The finding of high expression of poplar *SCL* genes during different developmental stages of SVS regeneration suggests that these genes are involved in the initiation and maintenance of nascent VC, which ultimately differentiates into xylem and phloem.

XTHs and MANs are able to hydrolyze xyloglucan- and mannan-type polysaccharides in cell wall remodeling (Hyodo et al., 2003; Schröder et al., 2009; Eklöf and Brumer, 2010). In particular, *pt-XTH9.1* was highly expressed during the VC initiation and differentiation stages; this result suggested that *pt-XTH9.1* is likely to be involved in the change in cell type during the initiation of cambial cell formation and the differentiation of cambial cells into xylem and phloem cells. In contrast, *pt-MAN2* and *pt-MAN5*, which displayed high expression levels during the VC differentiation stage, might primarily contribute to the differentiation of newly formed cambium. HD-Zip III TFs have been shown to regulate primary and secondary vascular tissue pattern formation (Ko et al., 2006) and to play key roles in SVS regeneration. Specifically, *ptrHB1*, the homolog of *Arabidopsis REV*, which is highly expressed in the SAM, cambial zone, and secondary vascular tissue of poplar (Robischon et al., 2011), was highly expressed on the 7 DAG. This observation suggested that *ptrHB1* performs an important function in cambium regeneration. The remaining 4 HD-ZIP III TFs, *ptrHB4*-*ptrHB7*, were specifically expressed during the VC differentiation stage, as evidenced by a sharp increase in their expression on the xylem side of the cambial zone in *P. trichocarpa* (Schrader et al., 2004). This finding indicates that these TFs play important roles in the differentiation of newly formed cambium into xylem and phloem at the late stage of SVS regeneration.

In summary, this study provided a holistic view of how miRNAs control different facets of SVS regeneration in poplar. A total of 157 and 75 target genes regulated by 21 known and 30 novel miRNA families, respectively, were dynamically expressed during the regeneration of SVS. Based on their corresponding GO terms, the targets of 15 miRNAs were enriched in auxin signaling pathway, cell differentiation, meristem development and pattern specification processes, and their roles in the VC initiation, formation, and differentiation stages were further explored. Therefore, the pathways through which these miRNA families regulate wood formation warrant further investigation.

## Author contributions

FT and ML designed the study. FT and SZ collected the plant materials and performed the experiments. FT and HW analyzed the data and drafted the manuscript. LW and HZ provided valuable suggestions and advices concerning the manuscript. All of the authors carefully checked and approved this manuscript.

### Conflict of interest statement

The authors declare that the research was conducted in the absence of any commercial or financial relationships that could be construed as a potential conflict of interest.

## Abbreviations

miRNAs: microRNAs
RISC: RNA-induced silencing complex
TF: transcription factor
VC: vascular cambium
SVS: secondary vascular system
DAG: days after girdling
pre-miRNA: precursor miRNA
qRT-PCR: quantitative real-time PCR
RPM: reads per million
RT: reverse transcription
ANOVA: one-way analysis of variance
PARE: parallel analysis of RNA ends
PCC: Pearson correlation coefficient
GO: gene ontology.
