# Supplementary material for: Identification of microRNAs Involved in Regeneration of the Secondary Vascular System in Populus tomentosa Carr
Source: Front Plant Sci. 2016 May 31;7:724. doi: 10.3389/fpls.2016.00724 (PMC4885845; doi:10.3389/fpls.2016.00724)
Supplement: Supplementary file 5 [file DataSheet1.DOCX]

Supplementary Material

**Identification of microRNAs Involved in Regeneration of the Secondary Vascular System in *Populus tomentosa* Carr.**

**Fang Tang, Hairong Wei, Shutang Zhao, Lijuan Wang, Huanquan Zheng, Mengzhu Lu^*^**

*** Correspondence:**

Corresponding Author: Mengzhu Lu

Email: [lumz@caf.ac.cn](mailto:lumz@caf.ac.cn)

**Supplementary material:**

Figure S1: Cross-sections of the regenerated tissues on the debarked trunk at six time points.

Figure S2: The expression profiles of known miRNAs during SVS regeneration based on sequencing and qRT-PCR analyses.

Figure S3: Predicted stem-loop structures of novel pre-miRNAs based on their cloned miRNA sequences.

Figure S4: The expression of novel miRNAs in different tissues of *P. tomentosa* based on universal qRT-PCR.

Figure S5: The expression of known miRNAs in different tissues of *P. tomentosa* based on universal qRT-PCR.

Figure S6: All t-plot lists of confirmed miRNA targets based on degradome sequencing analysis.

Figure S7: The expression profiles of miRNAs and their target genes during SVS regeneration as validated by universal qRT-PCR.

Figure S8: Pearson Correlation Coeffcients between the expression levels of miRNAs and their target genes during SVS regeneration.

Table S1: The annotation and classification of small RNAs in *P. tomentosa*.

Table S2: Summary of known miRNAs between *P. tomentosa* and *P. trichocarpa*.

Table S3: Summary of novel miRNAs that were identified in P. tomentosa.

Table S4: The expression levels of unique members of known and novel miRNA families.

Table S5: The primers were used for cloning and qRT-PCR of known and novel miRNAs.

Table S6: The novel miRNAs were confirmed by cloning and sequencing.

Table S7: The target genes of known and novel miRNAs were validated by degradome sequencing.

Table S8: The target genes of known and novel miRNAs were predicted using psRNAtarget.

Table S9: pre-miRNAs could be cleaved by their own or other mature miRNAs.

Table S10: The expression levels of miRNAs and their target genes during SVS regeneration.

Table S11: The expression levels of miRNAs and their target genes relative to the expression on the 7^th^ day AG.

Table S12: GO enrichment analysis of the targets of known and novel miRNAs based on degradome sequencing.
