# Supplementary material for: Identification of microRNAs Involved in Regeneration of the Secondary Vascular System in Populus tomentosa Carr
Source: Front Plant Sci. 2016 May 31;7:724. doi: 10.3389/fpls.2016.00724 (PMC4885845; doi:10.3389/fpls.2016.00724)
Supplement: Supplementary file 6 [file Image1.pdf]

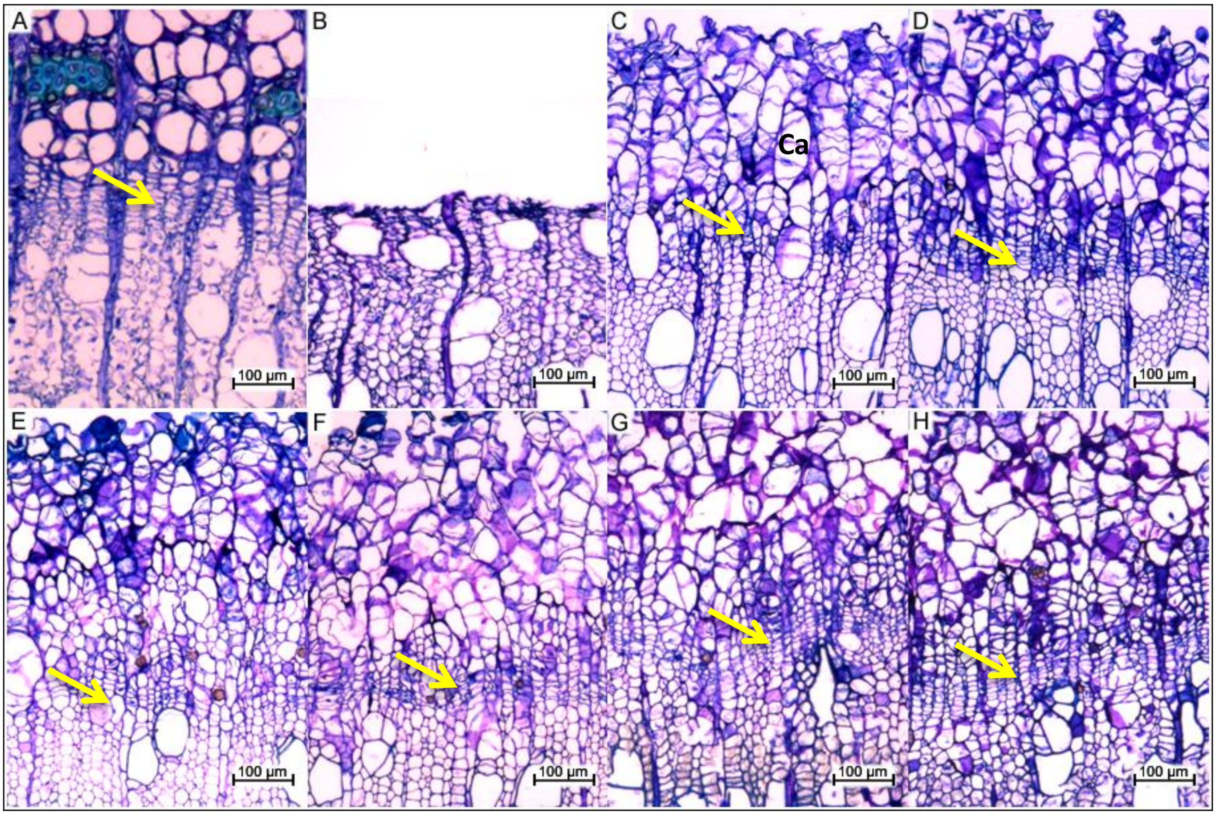

Figure S1. Cross sections of SVS regenerated tissues upon debarking. (A) the phloem, cambium zone and majority part of immature xylem were peeled off with the bark. (B) A few layers of immature xylem remained on the surface of the girdled trunk. (C) The callus covered the whole surface of the girdled trunk on the 7 DAG and discontinuous meristem cells were observed. (D) Continuous, flat meristem cells arranged irregularly between the callus and immature xylem on the 10 DAG. (E) The flat meristem cell layers had increased in size on the 12 DAG. (F) The flat meristem cell layers formed continuous and regular layers that are similar to a vascular cambium on the 16 DAG. (G) The newly cambium-like zone began to differentiate on the 18 DAG. (H) A large number of phloem cells outside the cambium and xylem cells inside the cambium were observed on the 21 DAG. The yellow arrows indicate the meristem cells and cambial region.

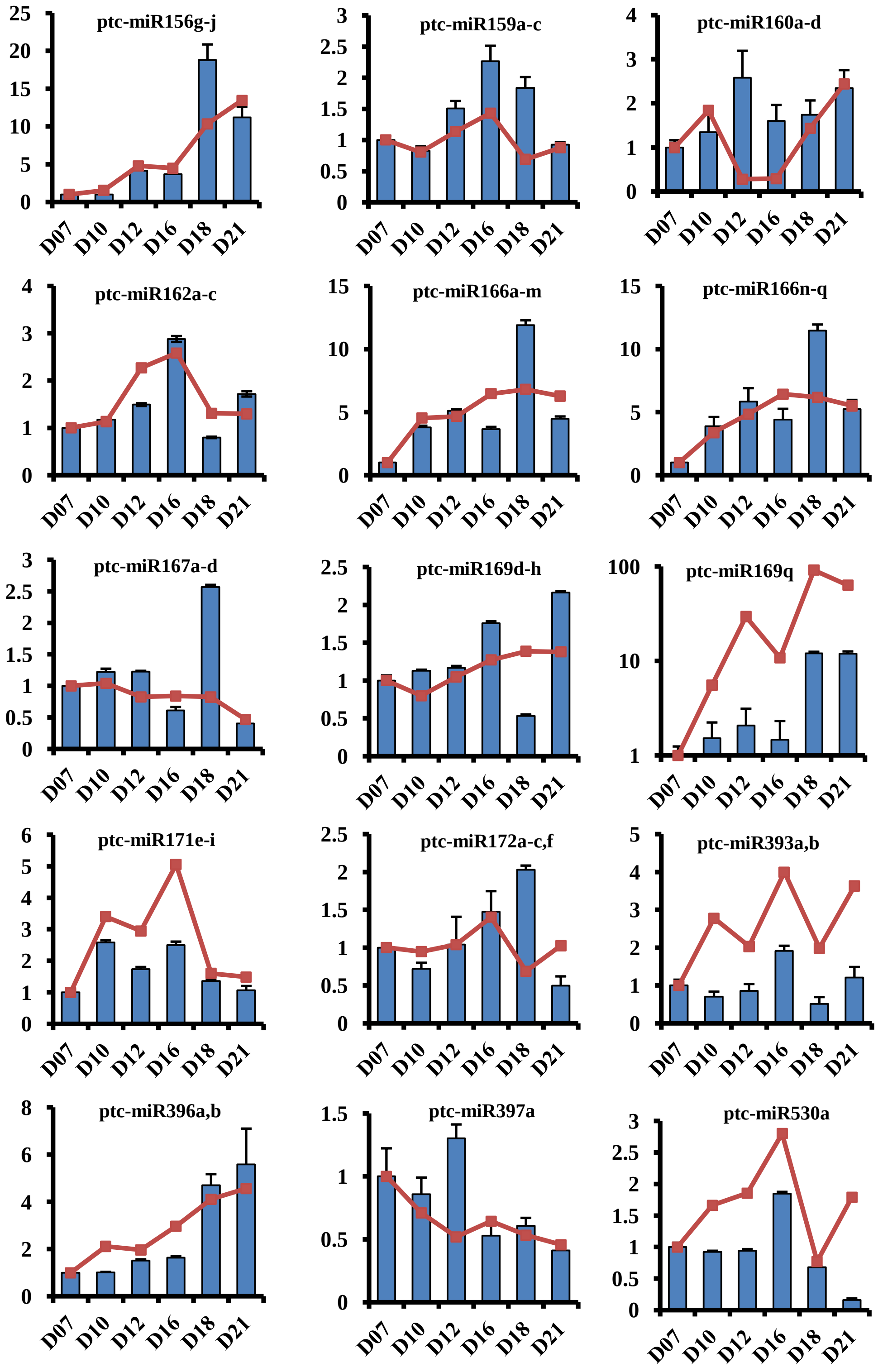

Figure S2. The expression profiles of known miRNAs during SVS regeneration based on sequencing and qRT-PCR analyses. The line chart represents the relative expression fold changes of known miRNAs from solexa sequencing reads and the histogram indicates the relative expression fold changes of known miRNAs analyzed by real-time quantity PCR. Transcript levels on the 7 DAG are set to 1. The X-axis shows the regeneration stages on the 7, 10, 12, 16, 18 and 21 DAG. The Y-axis shows the relative expression fold changes of known miRNAs.

pto-MIR003, ΔG = -61.00
```
    10        20        30          40         50
    U  U      UU      C          UG          AC-|
GAGGA CA AAAGA  GAUG AGCCAAGGA  ACUUGCCGACG    U
CUCCU GU UUUCU  UUAC UCGGUUCUU  UGGACGGUUGC    A
     C  C     UU     A          GU          CUA^
     130        120        110        100
```

pto-MIR014a, ΔG = -48.30
```
          10        20        30        40          50
ACAUAUCCAUU                           A      .-AAAAAUUA|
           AAAUGAUGACAUGGACACCAAAA CUUACAA           G
           UUUACUACUGUACCUGUGGUUUU GAAUGUU           C
AAAAAAAAAAU                           A      \ --------^
 .         180       170       160       150
```

pto-MIR037a, ΔG = -57.00
```
  10        20        30        40        50
     A               C            A     GAU
GUUAUA CAUCUUGAUCAAUGG CAUUGUAAGAGU GAAG   C
UAAUAU GUAGAAUUAGUUACC GUGACAUUCUCA CUUU   G
     G               C            -     AU-
  140       130       120       110
```

pto-MIR043c, ΔG = -29.80
```
        10        20          30         40
U       CUA  U         -     G         .-GCCCA|   A
 GGAGAGU   CA AUAUCAC AAAA UAGCUGA          CGAG \
 UUUUUCA   GU UAUAGUG UUUU GUUGACU          GUUC A
-       CC-  -         C     G         \ -----^   A
  130        120       110
```

pto-MIR045b, ΔG = -80.40
```
   10        20        30        40        50
   AAC-   A         GUA   A     .-U|      UUCAU
GUA    AAC CAACUGGAU   GAU GGAGU    GAGACUA     A
CAU    UUG GUUGAUCUG   UUG CCUCA    CUCUGGU     U
   AGGA   -         AUG   -     \ -^      UCUUU
      330       320       310       60
```

pto-MIR047a, ΔG = -81.10
```
         10        20        30        40
-   G        A     U          UC          AU
 GGG CUUACAUG AGAGG AGAGAGUGUAAU  AGCCAAGG  GA
 UCC GAAUGUAC UCUUC UCUCUCAUGUUA  UCGGUUCC  UU
A   G        -     U          CA          CG
  160       150       140       130       120
```

pto-MIR048b, ΔG = -72.60
```
 10        20        30        40        50
 GUCUAUUUUUUG                              CA-
A            AUCAGCAAUGUAGUCCUUCCGUCAACUUUGA   G
U            UGGUCGUUACAUCAGGAAGGCAGUUGAAACU   U
 ------------                              CUA
                 120       110       100
```

pto-MIR056a, ΔG = -53.20
```
          20         30        40          50
    -  UU   --      U                    C-
UGCC ACG  GU  UCAUUCC CUUCCUAAAAUGGCUUCUC  U
GUGG UGU  CA  AGUAAGG GAAGGAUUUUAUCGAAGAG  A
    C  GG   UA      -                    UA
    130       120       110       100
```

pto-MIR064b, ΔG = -43.90
```
       10        20            30          40
A         AUUA     A  --  --       .-UAAUUUAA|
 AUUUAUGUA    GUCGAG UA  CA  UGCAAGC          A
 UGAGUACGU    CAGCUU AU  GU  ACGUUUG          U
C         C---     A  UA  AA       \ --------^
     250           240       230
```

pto-MIR071, ΔG = -72.30
```
        10        20        30        40
AU|         UA   UU      G    C         -  CG
  GGAGAGGGAG  GGAU  UCGGAAG UUAU GGGUGGGUG AG  G
  CCUCUCCCUC  CUUA  AGCCUUU GAUA CCUACCCAC UC  C
CU^         --   CU      G    C         C  AU
   130        120       110       100        90
```

pto-MIR072, ΔG = -45.40
```
     10        20         30         40
U     ACAU      AGAU     GG     U     .-C
 UUGCC    UAGAUA    UUUGA  GUGGA GUUUU   G
 AACGG    GUUUGU    AAAUU  CGCCU CAAGA   C
-     ----      GC--     GA     -     \ -
           150        140        130
```

pto-MIR073, ΔG = -54.80
```
     10        20        30         40
G                    A          |   CUACAAA
 CAACACUUGCAUUACCAAAGUA CUUCUUCAA--GGAU       C
 GUUGUGAACGUAAUGGUUUUGU GAAGAAGUU  CCUG       G
-                    C          \ ^    UCA----
        150       140       130        70
```

pto-MIR074, ΔG = -71.50
```
     10        20        30         40
AU|         UA   UU      G   CU          -  G
  GGAGAGGGAG  GGGU  UCGGAAG UUA  GGAUGGGUG AGU G
  CCUCUCCCUC  CUUA  AGCCUUU GAU  CCUACCCAC UCA C
CU^         --   CU      G   AC          C  A
   120       110       100        90         80
```

pto-MIR075, ΔG = -20.90
```
         20        30        40         50
-------    U     UCU     UA    .-C|    CAUUA
       CUG CCGUU    UUACGA  GCU    AUAUG     U
       GAC GGCAA    GAUGUU  CGG    UAUAC     A
UUAAUAG    U     CCU     UG    \ -^    AAAUU
110        100       90              60
```

pto-MIR077, ΔG = -24.20
```
      10         20        30          40
    UUCAU    --       AG      .-ACAUAC      C
UCAA     GGAA  GAAAGAAUU AUGUC         AAGGAGC A
AGUU     CCUU  UUUUUUUGA UACAG         UUUUUCG U
    UUC--    UA       A-      \ ------      A
     150        140       130          100
```

pto-MIR078, ΔG = -44.80
```
    10        20         30
CAUCAACA  G    G   C-    U-    .-A   CG
        GU CUGGA UAG  UCAGU  GGUU   GAG  U
        CA GACCU GUC  GGUCA  CCAA   CUC  A
UACUAACC  -    G   UA    CC    \ -   A-
.         210       200       190
```

pto-MIR079, ΔG = -48.00
```
       20        30         40        50
      C          C          A     .-AAA
UGCACG UCUCGUAAUG UUCAUUCUCAC ACGGC     G
AUGUGU AGAGUAUUAC AAGUAAGGGUG UGCCG     U
      C          -          C     \ ---
   110       100        90          60
```

pto-MIR080, ΔG = -27.30
```
       10        20        30        40
C     UUG-----         AC  U      .-CUUUAAU    .-AUUA|
 GUGUC        AAGUUAAUG  CA AUAAAC         AGCC      U
 CAUAG        UUUAGUUGU  GU UGUUUG         UUGG      A
A     UGUCACUA         GA  -      \ -------    \ ----^
      190       180       170
```

pto-MIR093, ΔG = -38.90
```
     10        20         30         40
A        UG G          U  -    C      AU-------|  UC
 CUGGAAGU  U GGCAUGAGG GU UUGG AAGAAA          GGA  \
 GACCUUUA  A CCGUACUCU CA AACC UUCUUU          CCU  U
-        GU A          C  U    A      AGUAGUAUU^  UU
    90        80        70         60         50
```

pto-MIR094, ΔG = -28.30
```
     10        20          30       40
G     UCAG--  GU         CC  -   --|   UUCG
 GAGUG      GA  CCAUGCUCUC  UC UGA   AUCGG    \
 UUCAC      CU  GGUGCGGGAG  AG GCU   UGGUC    C
-     CCUAAA  UG         --  U   UC^   UUCU
   80        70          60        50
```

Figure S3. Predicted stem-loop structures of novel pre-miRNAs based on their cloned miRNA sequences.

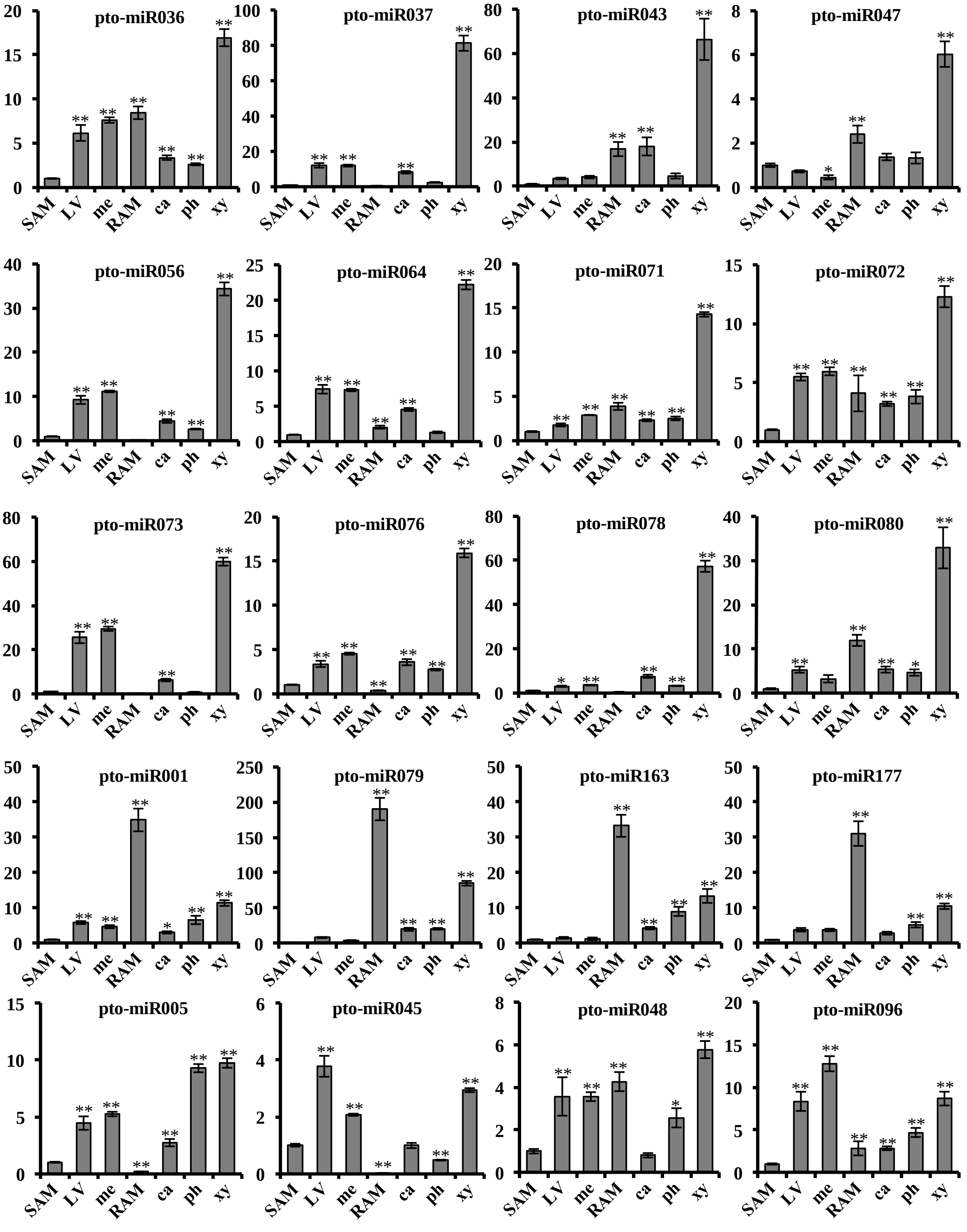

Figure S4. The expression of novel miRNAs in different tissues of *P. tomentosa* by universal qPCR. SAM, shoot apical meristem; LV, leaf vein; me, mesophyll; RAM, root apical meristem; ca, cambium; ph, phloem; xy, xylem. ** *P*-value≤0.01; * 0.01<*P*-value≤0.05.

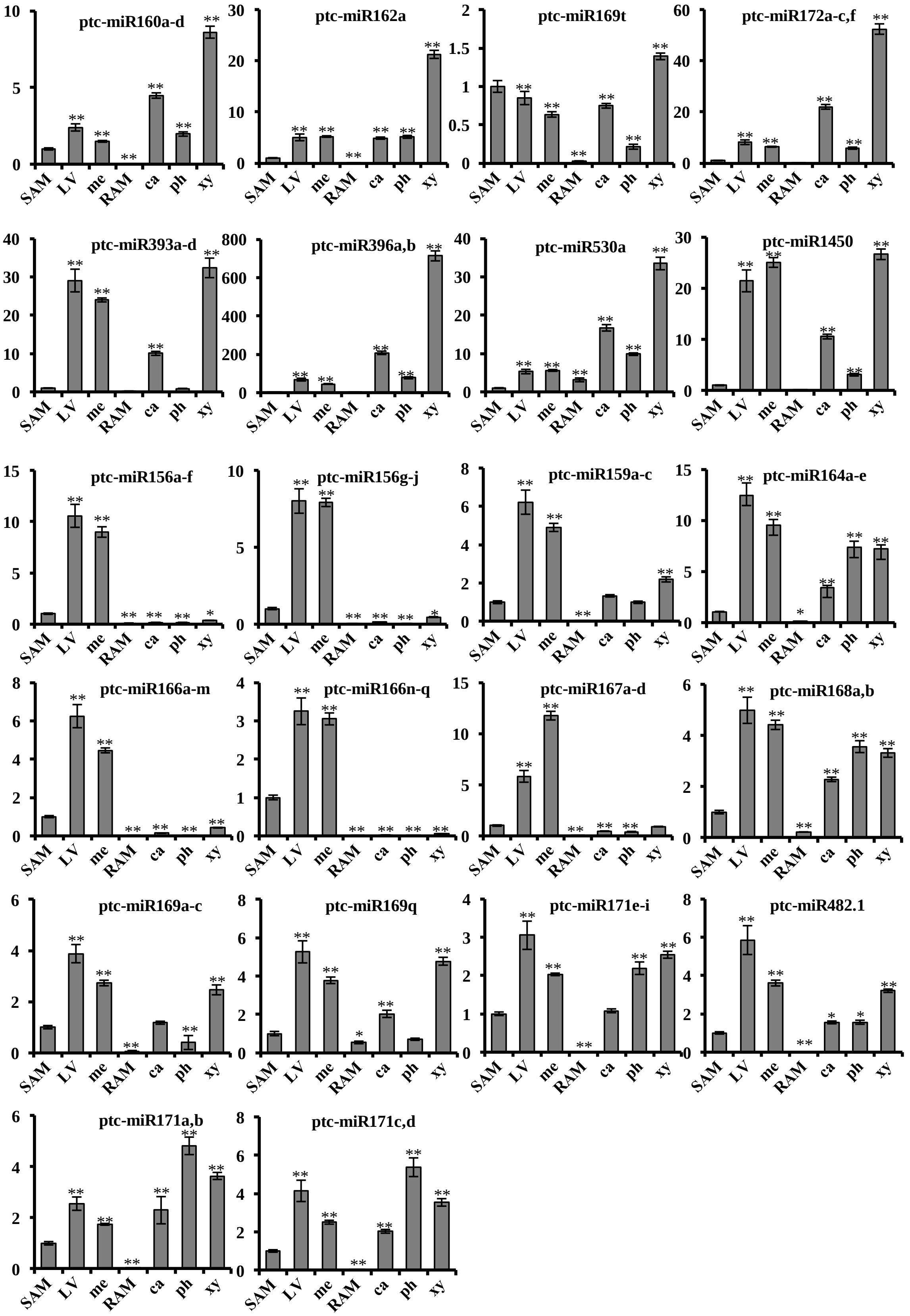

Figure S5. The expression of known miRNAs in different tissues of *P. tomentosa* by universal qPCR. SAM, shoot apical meristem; LV, leaf vein; me, mesophyll; RAM, root apical meristem; ca, cambium; ph, phloem; xy, xylem. ** *P*-value≤0.01; * 0.01<*P*-value≤0.05.

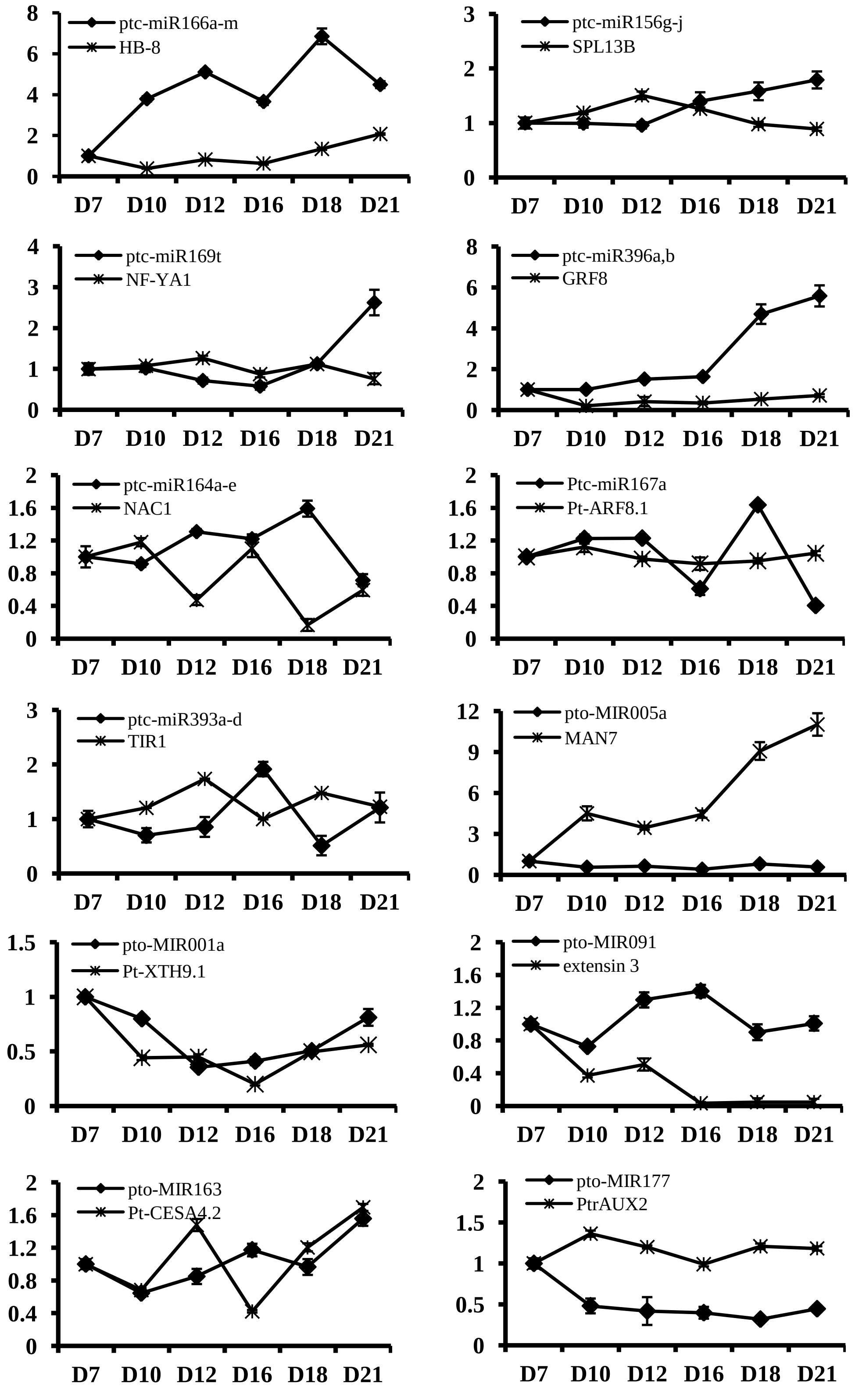

Figure S7. The expression profiles of miRNAs and their target genes during SVS regeneration as validated by universal qRT-PCR.

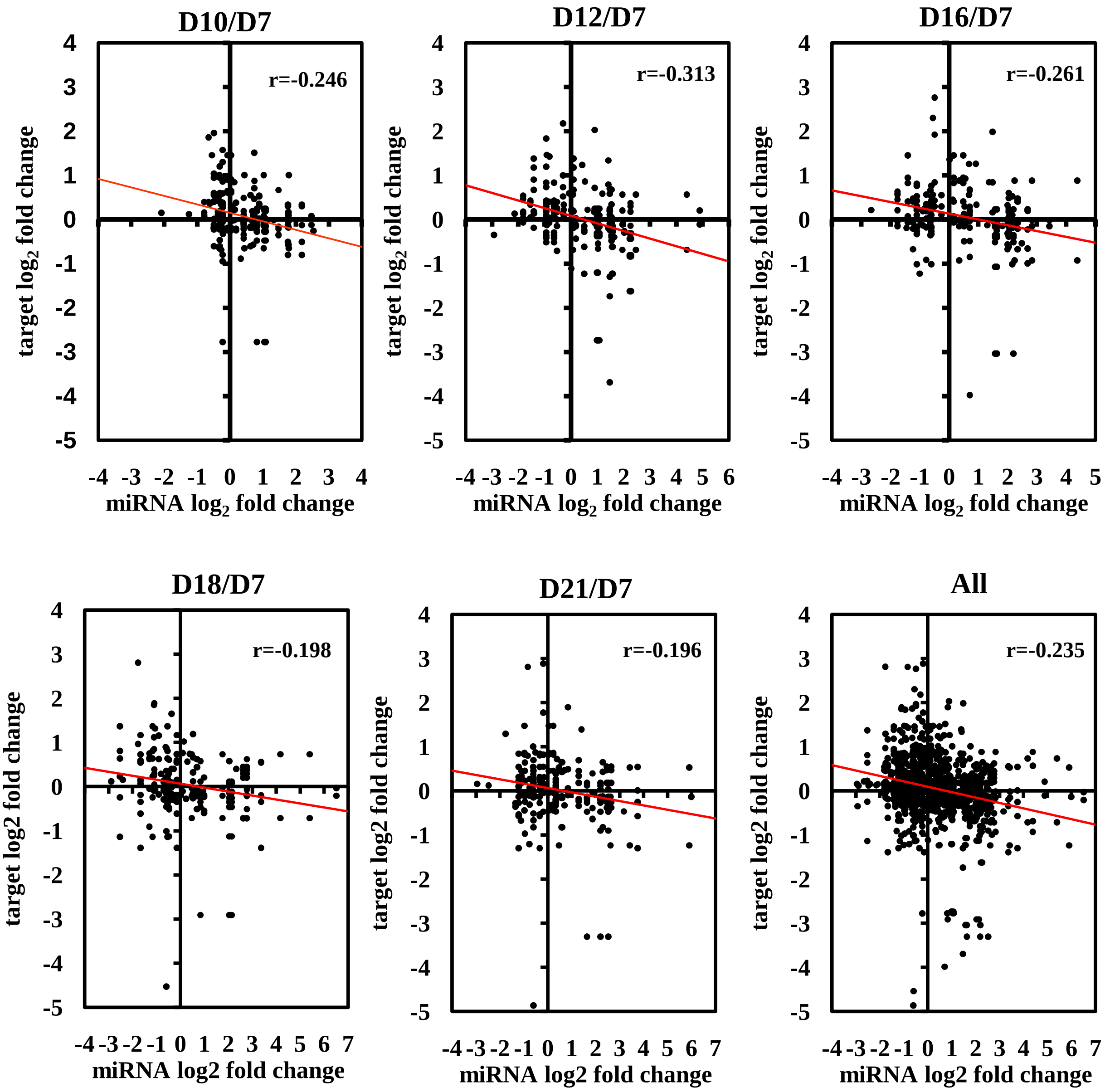

Figure S8. Pearson Correlation Coeffcient between the expression of miRNAs and their target genes during SVS regeneration.
