# Supplementary material for: Identification of microRNAs Involved in Regeneration of the Secondary Vascular System in Populus tomentosa Carr
Source: Front Plant Sci. 2016 May 31;7:724. doi: 10.3389/fpls.2016.00724 (PMC4885845; doi:10.3389/fpls.2016.00724)
Supplement: Supplementary file 7 [file Image2.PDF]

Figure S6. All t-plot lists of confirmed miRNA targets based on degradome sequencing analysis.

Example:

category=0, cleavage_site=1477
query=ptc-miR156a-f, target=Potri.003G169400.1,
score=1, range=1467-1486, strand=1

```
target  5' GUGCUCUCUCuCUUCUGUCA 3'
           :::::: :::::::::::::
query   3' CACGAGUGAGAGAAGACAGU 5'
```

>Potri.003G169400.1
#size=2113

position	Raw_reads	Rep_Norm_reads	Num_Uniq_mapped	Category	cleavage_site
223	1	1	1	4	
507	1	1	1	4	
795	1	0.5	0	4	
872	1	0.5	0	4	
936	1	0.5	0	4	
963	1	0.333333333333333	0	4	
1042	1	0.333333333333333	0	4	
1043	1	0.333333333333333	0	4	
1048	1	0.333333333333333	0	4	
1050	1	0.333333333333333	0	4	
1063	1	0.333333333333333	0	4	
1095	1	0.333333333333333	0	4	
1162	1	0.333333333333333	0	4	
1215	1	0.5	0	4	
1256	1	0.333333333333333	0	4	
1419	1	0.5	0	4	
1457	1	0.333333333333333	0	4	
1461	1	0.333333333333333	0	4	
1477	15	5	0	0	<<<
1487	1	0.333333333333333	0	4	
1539	1	0.333333333333333	0	4	
1544	1	0.333333333333333	0	4	
1638	1	0.5	0	4	
1641	1	0.5	0	4	
1675	1	0.5	0	4	
1676	1	0.5	0	4	
1690	1	0.333333333333333	0	4	
1701	2	0.666666666666667	0	2	
1714	1	0.333333333333333	0	4	
1726	1	0.333333333333333	0	4	
1732	1	0.333333333333333	0	4	
1823	1	0.5	0	4	
1872	1	0.5	0	4	
1880	1	0.5	0	4	
1884	1	0.5	0	4	

Position: the cleavage site in transcript sequences.
Raw_reads: the number of raw reads with 5' ends at that position.
Rep_Norm_reads: repeat-normalized number of reads with 5' ends at that position，The RPM (reads per million)abundances of any degradome sequences with multiple transcriptome hits are repeat normalized; the abundance is divided by the total number of hits to give ‘normalized reads per million’(NRPM)."
Num_Uniq_mapped: the number of uniquely mapped reads with 5' ends at that position.
Category: Category designation at that position.
Cleavage_site: miRNA target cleaveage position flag.

ptc-miR156a-f

category=4, cleavage_site=1392
query=ptc-miR156a-f, target=Potri.001G055900.1,
score=1, range=1382-1401, strand=1

```
target  5' GUGCUCUCUCuCUUCUGUCA 3'
            :::::: :::::::::::::
query   3' CACGAGUGAGAGAAGACAGU 5'
```

>Potri.001G055900.1
#size=2796

111	1	1	1	4	
112	1	1	1	4	
163	1	0.2	0	4	
167	3	0.6	0	2	
237	1	0.2	0	4	
277	1	0.2	0	4	
557	1	0.2	0	4	
750	1	0.2	0	4	
804	1	0.1	0	4	
841	1	0.1	0	4	
876	1	0.1	0	4	
885	1	0.1	0	4	
913	1	0.1	0	4	
922	1	0.1	0	4	
934	1	0.1	0	4	
936	1	0.1	0	4	
941	1	0.1	0	4	
942	1	0.1	0	4	
949	1	0.1	0	4	
955	1	0.1	0	4	
957	1	0.1	0	4	
971	1	0.1	0	4	
977	1	0.1	0	4	
978	1	0.1	0	4	
990	1	0.1	0	4	
997	2	0.4	0	2	
1013	2	0.4	0	2	
1021	1	0.2	0	4	
1022	1	0.2	0	4	
1035	1	0.2	0	4	
1088	1	0.2	0	4	
1117	1	0.2	0	4	
1124	1	0.2	0	4	
1156	1	0.1	0	4	
1169	1	0.2	0	4	
1239	1	0.1	0	4	
1244	1	0.1	0	4	
1249	1	0.2	0	4	
1289	1	0.25	0	4	
1392	1	0.2	0	4	<<<
1605	1	0.125	0	4	
1606	1	0.125	0	4	
1615	1	0.2	0	4	
1673	1	0.2	0	4	
1705	1	0.5	0	4	
1804	1	0.5	0	4	
1894	1	0.5	0	4	
2151	1	0.25	0	4	

2162	1	0.25	0	4	
2222	1	0.25	0	4	
2294	1	0.25	0	4	
2313	1	0.25	0	4	
2318	1	0.25	0	4	
2320	1	0.25	0	4	

category=0, cleavage_site=1417
query=ptc-miR156a-f, target=Potri.001G058600.1,
score=1, range=1407-1426, strand=1

```
target  5' GUGCUCUCUCuCUUCUGUCA 3'
           :::::: ::::::::::::
query   3' CACGAGUGAGAGAAGACAGU 5'
```

>Potri.001G058600.1
#size=2079

109	1	1	1	4	
459	1	1	1	4	
525	1	1	1	4	
530	1	1	1	4	
623	1	1	1	4	
653	1	1	1	4	
872	1	1	1	4	
875	1	1	1	4	
903	1	0.333333333333333	0	4	
982	1	0.333333333333333	0	4	
983	1	0.333333333333333	0	4	
988	1	0.333333333333333	0	4	
990	1	0.333333333333333	0	4	
1003	1	0.333333333333333	0	4	
1020	1	1	1	4	
1035	1	0.333333333333333	0	4	
1102	1	0.333333333333333	0	4	
1196	1	0.333333333333333	0	4	
1227	1	1	1	4	
1275	1	1	1	4	
1316	1	1	1	4	
1397	1	0.333333333333333	0	4	
1401	1	0.333333333333333	0	4	
1417	15	5	0	0	<<<
1427	1	0.333333333333333	0	4	
1428	1	1	1	4	
1435	1	1	1	4	
1479	1	0.333333333333333	0	4	
1484	1	0.333333333333333	0	4	
1494	2	2	2	2	
1528	1	1	1	4	
1529	1	1	1	4	
1559	1	1	1	4	
1566	2	2	2	2	
1609	1	1	1	4	
1615	1	1	1	4	
1630	1	0.333333333333333	0	4	
1641	2	0.666666666666667	0	3	
1654	1	0.333333333333333	0	4	
1666	1	0.333333333333333	0	4	
1672	1	0.333333333333333	0	4	
1682	1	1	1	4	
1693	1	1	1	4	

1727	1	1	1	4
1755	1	1	1	4
1756	1	1	1	4
1811	1	1	1	4
1818	1	1	1	4
1819	2	2	2	2
1822	1	1	1	4
1823	2	2	2	2
1864	1	1	1	4
1865	1	1	1	4

---

category=4, cleavage_site=1034
query=ptc-miR156a-f, target=Potri.002G142400.1,
score=1, range=1024-1043, strand=1

```
target  5' GUGCUCUCUCuCUUCUGUCA 3'
          :::::: ::::::::::::: 
query   3' CACGAGUGAGAGAAGACAGU 5'
```

>Potri.002G142400.1
#size=1662

465	1	1	1	4	
982	1	0.5	0	4	
1034	1	0.5	0	4	<<<
1193	1	1	1	4	
1335	1	1	1	4	

---

category=0, cleavage_site=1477
query=ptc-miR156a-f, target=Potri.003G169400.1,
score=1, range=1467-1486, strand=1

```
target  5' GUGCUCUCUCuCUUCUGUCA 3'
            :::::: ::::::::::::: 
query   3' CACGAGUGAGAGAAGACAGU 5'
```

>Potri.003G169400.1
#size=2113

223	1	1	1	4	
507	1	1	1	4	
795	1	0.5	0	4	
872	1	0.5	0	4	
936	1	0.5	0	4	
963	1	0.333333333333333	0	4	
1042	1	0.333333333333333	0	4	
1043	1	0.333333333333333	0	4	
1048	1	0.333333333333333	0	4	
1050	1	0.333333333333333	0	4	
1063	1	0.333333333333333	0	4	
1095	1	0.333333333333333	0	4	
1162	1	0.333333333333333	0	4	
1215	1	0.5	0	4	
1256	1	0.333333333333333	0	4	
1419	1	0.5	0	4	
1457	1	0.333333333333333	0	4	
1461	1	0.333333333333333	0	4	
1477	15	5	0	0	<<<
1487	1	0.333333333333333	0	4	
1539	1	0.333333333333333	0	4	
1544	1	0.333333333333333	0	4	
1638	1	0.5	0	4	
1641	1	0.5	0	4	
1675	1	0.5	0	4	

1676	1	0.5	0	4
1690	1	0.333333333333333	0	4
1701	2	0.666666666666667	0	2
1714	1	0.333333333333333	0	4
1726	1	0.333333333333333	0	4
1732	1	0.333333333333333	0	4
1823	1	0.5	0	4
1872	1	0.5	0	4
1880	1	0.5	0	4
1884	1	0.5	0	4

---

category=4, cleavage_site=1087
query=ptc-miR156a-f, target=Potri.005G099600.1,
score=3.5, range=1078-1096, strand=1

```
target  5' GUGCUU-CUCuCUUCUGCCA 3'
           ::::::. :::::::::: ::
query   3' CACGAGUGAGAGAAGACAGU 5'
```

>Potri.005G099600.1
#size=1672

175	1	0.25	0	4	
369	1	0.25	0	4	
376	2	1	0	2	
393	1	0.5	0	4	
442	1	0.25	0	4	
495	1	0.125	0	4	
512	1	0.25	0	4	
558	2	0.333333333333333	0	3	
559	1	0.125	0	4	
560	5	0.666666666666667	0	2	
611	1	0.25	0	4	
630	1	0.125	0	4	
669	1	0.25	0	4	
868	1	0.166666666666667	0	4	
878	3	0.375	0	3	
879	6	0.75	0	2	
915	1	0.166666666666667	0	4	
920	1	0.166666666666667	0	4	
929	1	0.166666666666667	0	4	
932	1	0.25	0	4	
959	3	0.75	0	2	
964	1	0.25	0	4	
1016	1	0.25	0	4	
1049	1	0.25	0	4	
1075	1	0.25	0	4	
1079	2	0.285714285714286	0	3	
1087	1	0.25	0	4	<<<
1091	8	2	0	2	
1102	1	0.25	0	4	
1109	1	0.25	0	4	
1113	2	0.5	0	2	
1114	1	0.25	0	4	
1115	3	0.75	0	2	
1116	1	0.25	0	4	
1118	1	0.25	0	4	
1119	1	0.25	0	4	
1120	1	0.25	0	4	
1121	3	0.75	0	2	
1122	2	0.5	0	2	

1123	3	0.75	0	2
1124	1	0.25	0	4
1125	4	1	0	2
1127	1	0.25	0	4
1130	10	2.5	0	2
1131	1	0.25	0	4
1133	1	0.25	0	4
1134	1	0.25	0	4
1137	6	1.5	0	2
1138	8	2	0	2
1177	2	0.5	0	2
1184	12	3	0	2
1185	4	1	0	2
1202	2	0.5	0	2
1203	2	0.5	0	2
1206	14	3.41666666666667	0	2
1207	13	3.25	0	2
1208	2	0.5	0	2
1209	7	1.75	0	2
1210	19	4.75	0	2
1211	38	9.5	0	0
1212	14	3.5	0	2
1213	1	0.25	0	4
1215	2	0.5	0	2
1216	2	0.5	0	2
1217	11	2.75	0	2
1219	7	1.75	0	2
1220	23	5.75	0	2
1221	15	3.75	0	2
1222	8	2	0	2
1223	3	0.75	0	2
1224	11	3	0	2
1225	1	0.25	0	4
1226	1	0.25	0	4
1227	1	0.25	0	4
1228	4	1	0	2
1229	7	1.75	0	2
1230	3	0.75	0	2
1231	6	1.5	0	2
1232	1	0.25	0	4
1233	1	0.25	0	4
1234	2	0.5	0	2
1419	1	0.25	0	4
1425	1	0.25	0	4
1440	3	1.5	0	2
1449	1	0.5	0	4
1493	1	0.5	0	4
1494	4	2	0	2
1497	1	0.5	0	4
1502	2	1	0	2
1503	1	0.5	0	4
1504	1	0.5	0	4
1530	1	0.5	0	4
1540	3	1.5	0	2
1541	1	0.5	0	4
1544	3	1.5	0	2
1564	1	0.5	0	4
1566	1	0.5	0	4

1569	1	0.5	0	4
1571	1	0.5	0	4
1574	2	1	0	2
1575	10	5	0	2
1576	1	0.5	0	4
1577	1	0.5	0	4
1582	7	3.5	0	2
1630	1	0.5	0	4

---

category=0, cleavage_site=994
query=ptc-miR156a-f, target=Potri.007G138800.1,
score=2, range=984-1003, strand=1

```
target  5' UUGCUCUCUCuCUUCUGUCA 3'
             ::::: ::::::::::::
query   3' CACGAGUGAGAGAAGACAGU 5'
```

>Potri.007G138800.1
#size=1410

363	1	0.333333333333333	0	4	
450	1	0.333333333333333	0	4	
550	1	0.333333333333333	0	4	
656	1	0.333333333333333	0	4	
760	1	0.333333333333333	0	4	
766	1	0.333333333333333	0	4	
994	2	0.666666666666667	0	0	<<<

---

category=4, cleavage_site=1751
query=ptc-miR156a-f, target=Potri.008G097900.1,
score=1, range=1741-1760, strand=1

```
target  5' GUGCUCUCUCuCUUCUGUCA 3'
            :::::: ::::::::::::
query   3' CACGAGUGAGAGAAGACAGU 5'
```

>Potri.008G097900.1
#size=2659

555	1	0.333333333333333	0	4	
953	1	0.2	0	4	
968	1	0.2	0	4	
972	1	0.333333333333333	0	4	
979	1	0.2	0	4	
981	1	0.2	0	4	
1214	1	0.333333333333333	0	4	
1242	1	0.2	0	4	
1317	1	0.333333333333333	0	4	
1751	1	0.333333333333333	0	4	<<<
1830	1	0.333333333333333	0	4	
2096	1	0.2	0	4	
2134	1	0.333333333333333	0	4	
2226	1	0.333333333333333	0	4	
2277	1	0.333333333333333	0	4	
2278	1	0.333333333333333	0	4	
2282	1	0.333333333333333	0	4	
2285	1	0.333333333333333	0	4	
2383	1	0.333333333333333	0	4	

category=4, cleavage_site=1185
query=ptc-miR156a-f, target=Potri.010G154300.1,
score=1, range=1175-1194, strand=1

```
target  5' GUGCUCUCUCuCUUCUGUCA 3'
          :::::: ::::::::::::
query   3' CACGAGUGAGAGAAGACAGU 5'
```

>Potri.010G154300.1
#size=2131

381	1	0.2	0	4	
396	1	0.2	0	4	
407	1	0.2	0	4	
409	1	0.2	0	4	
673	1	0.2	0	4	
893	1	0.5	0	4	
951	1	0.5	0	4	
1185	1	0.5	0	4	<<<
1186	2	1	0	0	
1294	1	0.5	0	4	
1367	1	0.5	0	4	
1384	1	0.5	0	4	
1533	1	0.2	0	4	
1691	1	0.5	0	4	
1771	1	0.5	0	4	

category=4, cleavage_site=739
query=ptc-miR156a-f, target=Potri.011G055900.1,
score=2, range=729-748, strand=1

```
target  5' AUGCUCUCUCuCUUCUGUCA 3'
             ::::: ::::::::::::
query   3' CACGAGUGAGAGAAGACAGU 5'
```

>Potri.011G055900.1
#size=864

70	1	1	1	4
71	1	1	1	4
82	2	2	2	2
106	1	1	1	4
121	1	1	1	4
148	1	1	1	4
155	1	1	1	4
167	1	0.5	0	4
187	1	0.5	0	4
192	2	2	2	2
200	1	1	1	4
201	1	1	1	4
207	2	2	2	2
222	1	1	1	4
229	3	3	3	0
231	1	1	1	4
244	1	0.5	0	4
256	1	1	1	4
271	1	1	1	4
279	1	1	1	4
293	1	1	1	4
331	1	1	1	4
354	1	0.5	0	4
361	1	0.5	0	4
372	1	0.5	0	4
373	1	0.5	0	4

379	1	0.5	0	4	
402	1	1	1	4	
403	1	1	1	4	
406	1	1	1	4	
431	1	1	1	4	
439	1	1	1	4	
456	1	0.5	0	4	
469	1	0.5	0	4	
486	1	0.5	0	4	
491	1	0.5	0	4	
493	1	0.5	0	4	
499	1	1	1	4	
513	1	0.5	0	4	
515	2	1	0	2	
519	1	0.5	0	4	
521	1	0.5	0	4	
532	1	1	1	4	
552	1	1	1	4	
553	2	2	2	2	
555	1	1	1	4	
556	1	1	1	4	
562	1	1	1	4	
593	1	1	1	4	
633	1	1	1	4	
637	2	2	2	2	
653	1	1	1	4	
657	2	2	2	2	
666	1	1	1	4	
669	1	1	1	4	
673	1	1	1	4	
677	1	1	1	4	
691	1	1	1	4	
739	1	1	1	4	<<<
740	1	1	1	4	

---

category=1, cleavage_site=1090
query=ptc-miR156a-f, target=Potri.011G116800.1,
score=2, range=1080-1099, strand=1

```
target  5' AUGCUCCCUCuCUUCUGUCA 3'
           ::::: ::::::::::::
query   3' CACGAGUGAGAGAAGACAGU 5'
```

>Potri.011G116800.1
#size=1360

349	1	1	1	4
350	1	1	1	4
370	1	1	1	4
400	1	1	1	4
401	1	1	1	4
407	1	1	1	4
421	1	1	1	4
526	1	1	1	4
561	1	1	1	4
603	1	1	1	4
606	1	1	1	4
621	1	1	1	4
625	1	1	1	4
642	1	1	1	4
643	1	1	1	4

668	1	1	1	4	
699	1	1	1	4	
707	1	1	1	4	
720	1	1	1	4	
730	1	0.5	0	4	
744	1	1	1	4	
746	1	1	1	4	
797	1	1	1	4	
815	3	3	3	1	
847	1	0.5	0	4	
850	1	0.5	0	4	
852	1	0.5	0	4	
860	1	0.5	0	4	
876	1	1	1	4	
881	1	1	1	4	
888	1	1	1	4	
892	1	1	1	4	
906	1	1	1	4	
915	2	2	2	2	
924	1	1	1	4	
989	1	1	1	4	
1090	3	3	3	1	<<<
1113	1	1	1	4	
1114	1	1	1	4	
1115	1	1	1	4	
1139	1	1	1	4	
1235	1	1	1	4	
1271	1	1	1	4	

---

category=4, cleavage_site=1087
query=ptc-miR156a-f, target=Potri.012G100700.1,
score=2, range=1077-1096, strand=1

```
target  5' GCGCUCUCUCuCUUCUGUCA 3'
           : :::: :::::::::::::
query   3' CACGAGUGAGAGAAGACAGU 5'
```

>Potri.012G100700.1
#size=1454

1087	1	0.142857142857143	0	4	<<<
1312	1	0.142857142857143	0	4	
1318	1	0.142857142857143	0	4	
1345	1	0.25	0	4	
1388	1	0.25	0	4	
1389	2	0.5	0	2	
1392	3	0.75	0	0	
1397	3	0.428571428571429	0	2	
1398	1	0.142857142857143	0	4	
1401	1	0.25	0	4	

---

category=4, cleavage_site=1157
query=ptc-miR156a-f, target=Potri.014G057800.1,
score=1, range=1147-1166, strand=1

```
target  5' GUGCUCUCUCuCUUCUGUCA 3'
           :::::: :::::::::::::
query   3' CACGAGUGAGAGAAGACAGU 5'
```

>Potri.014G057800.1
#size=1765

269	1	1	1	4
794	1	1	1	4

1105	1	0.5	0	4	
1157	1	0.5	0	4	<<<

---

category=4, cleavage_site=1586
query=ptc-miR156a-f, target=Potri.015G060400.1,
score=0.5, range=1576-1595, strand=1

```
target  5' GUGCUCGCUCuCUUCUGUCA 3'
           ::::::.:::::::::::::
query   3' CACGAGUGAGAGAAGACAGU 5'
```

>Potri.015G060400.1
#size=2383

531	1	0.2	0	4	
807	1	0.25	0	4	
1586	1	0.5	0	4	<<<
1866	1	0.5	0	4	
2159	1	0.5	0	4	
2177	1	0.5	0	4	
2183	3	1.5	0	0	
2184	2	1	0	2	
2188	1	0.5	0	4	
2203	1	0.5	0	4	
2205	2	1	0	2	
2262	1	0.5	0	4	

---

category=4, cleavage_site=1225
query=ptc-miR156a-f, target=Potri.015G098900.1,
score=1, range=1215-1234, strand=1

```
target  5' GUGCUCUCUCuCUUCUGUCA 3'
           :::::: :::::::::::::
query   3' CACGAGUGAGAGAAGACAGU 5'
```

>Potri.015G098900.1
#size=1722

31	1	0.5	0	4
55	1	0.5	0	4
161	1	0.5	0	4
203	1	0.333333333333333	0	4
223	2	0.666666666666667	0	1
250	1	0.333333333333333	0	4
274	1	0.333333333333333	0	4
276	1	0.333333333333333	0	4
354	1	0.333333333333333	0	4
356	1	0.333333333333333	0	4
365	1	0.333333333333333	0	4
383	1	0.333333333333333	0	4
387	1	0.142857142857143	0	4
395	2	0.666666666666667	0	1
446	1	0.142857142857143	0	4
449	1	0.142857142857143	0	4
481	1	0.333333333333333	0	4
545	1	0.333333333333333	0	4
554	1	0.333333333333333	0	4
596	1	0.333333333333333	0	4
628	1	0.333333333333333	0	4
629	1	0.333333333333333	0	4
639	1	0.142857142857143	0	4
645	1	0.142857142857143	0	4
646	1	0.142857142857143	0	4
692	1	0.333333333333333	0	4

720	1	0.333333333333333	0	4	
723	1	0.333333333333333	0	4	
726	1	0.333333333333333	0	4	
752	1	0.142857142857143	0	4	
758	1	0.142857142857143	0	4	
786	1	0.142857142857143	0	4	
798	1	0.142857142857143	0	4	
829	1	0.333333333333333	0	4	
851	1	0.333333333333333	0	4	
884	1	0.333333333333333	0	4	
904	1	0.333333333333333	0	4	
1027	1	0.333333333333333	0	4	
1075	1	0.333333333333333	0	4	
1113	1	0.333333333333333	0	4	
1165	1	0.333333333333333	0	4	
1225	1	0.333333333333333	0	4	<<<
1246	1	0.333333333333333	0	4	
1252	2	0.666666666666667	0	1	
1253	2	0.666666666666667	0	1	
1292	1	0.333333333333333	0	4	
1301	1	0.333333333333333	0	4	
1314	1	0.333333333333333	0	4	
1344	1	0.333333333333333	0	4	
1355	1	0.333333333333333	0	4	
1359	1	0.333333333333333	0	4	
1366	1	0.333333333333333	0	4	
1373	1	0.333333333333333	0	4	
1382	1	0.333333333333333	0	4	
1392	1	0.333333333333333	0	4	
1422	1	0.142857142857143	0	4	
1435	1	0.333333333333333	0	4	
1453	1	0.333333333333333	0	4	
1471	1	0.333333333333333	0	4	
1497	1	0.333333333333333	0	4	

ptc-miR156g-j

---

category=3, cleavage_site=1418
query=ptc-miR156g-j, target=Potri.001G058600.1,
score=3, range=1407-1427, strand=1

```
target  5' GUGCUCUCUCUcUUCUGUCAU 3'
           ::::::::: ::::::::::
query   3' CACGAGAGAUAGAAGACAGUU 5'
```

>Potri.001G058600.1
#size=2079

177	1	1	1	4
344	1	1	1	4
352	1	1	1	4
863	1	1	1	4
924	1	0.333333333333333	0	4
934	1	0.333333333333333	0	4
966	1	0.333333333333333	0	4
973	1	0.333333333333333	0	4
982	1	0.333333333333333	0	4
984	1	0.142857142857143	0	4
1007	2	2	2	2
1013	1	1	1	4
1016	1	1	1	4

1022	1	1	1	4			
1024	1	0.333333333333333			0	4	
1029	1	0.333333333333333			0	4	
1044	1	0.333333333333333			0	4	
1059	1	0.333333333333333			0	4	
1064	1	0.333333333333333			0	4	
1102	1	0.333333333333333			0	4	
1140	1	0.333333333333333			0	4	
1174	1	1	1	4			
1196	1	0.333333333333333			0	4	
1201	1	1	1	4			
1228	1	1	1	4			
1300	1	1	1	4			
1379	1	0.333333333333333			0	4	
1385	1	0.333333333333333			0	4	
1397	1	0.333333333333333			0	4	
1405	2	0.666666666666667			0	3	
1408	1	0.333333333333333			0	4	
1417	12	4	0	0			
1418	2	0.666666666666667			0	3	<<<
1428	1	0.333333333333333			0	4	
1439	1	1	1	4			
1442	1	1	1	4			
1444	1	1	1	4			
1446	1	1	1	4			
1447	3	3	3	2			
1454	1	1	1	4			
1477	1	0.333333333333333			0	4	
1515	1	1	1	4			
1529	1	1	1	4			
1530	1	1	1	4			
1543	1	1	1	4			
1554	1	1	1	4			
1573	1	1	1	4			
1634	1	0.333333333333333			0	4	
1636	1	0.333333333333333			0	4	
1640	1	0.333333333333333			0	4	
1641	1	0.333333333333333			0	4	
1642	1	0.333333333333333			0	4	
1657	1	0.333333333333333			0	4	
1667	1	0.333333333333333			0	4	
1673	1	0.333333333333333			0	4	
1674	1	0.333333333333333			0	4	
1678	1	1	1	4			
1696	1	1	1	4			
1700	1	1	1	4			
1701	1	1	1	4			
1702	2	2	2	2			
1703	2	2	2	2			
1704	1	1	1	4			
1712	1	1	1	4			
1715	2	2	2	2			
1722	1	1	1	4			
1728	1	1	1	4			
1772	1	1	1	4			
1811	2	2	2	2			
1812	1	1	1	4			
1815	1	1	1	4			

1817	1	1	1	4
1818	2	2	2	2
1824	1	1	1	4
1837	1	1	1	4
1914	1	1	1	4

---

category=2, cleavage_site=1478
query=ptc-miR156g-j, target=Potri.003G169400.1,
score=3, range=1467-1487, strand=1

```
target  5' GUGCUCUCUCUcUUCUGUCAU 3'
           ::::::::: ::::::::::
query   3' CACGAGAGAUAGAAGACAGUU 5'
```

>Potri.003G169400.1
#size=2113

57	1	1	1	4	
348	1	1	1	4	
529	1	0.5	0	4	
530	2	1	0	2	
558	1	0.5	0	4	
608	2	1	0	2	
697	1	0.5	0	4	
801	1	0.5	0	4	
984	1	0.333333333333333	0	4	
994	1	0.333333333333333	0	4	
1026	1	0.333333333333333	0	4	
1033	1	0.333333333333333	0	4	
1042	1	0.333333333333333	0	4	
1044	1	0.142857142857143	0	4	
1084	1	0.333333333333333	0	4	
1089	1	0.333333333333333	0	4	
1104	1	0.333333333333333	0	4	
1119	1	0.333333333333333	0	4	
1124	1	0.333333333333333	0	4	
1151	1	0.5	0	4	
1162	1	0.333333333333333	0	4	
1177	1	0.5	0	4	
1180	1	0.5	0	4	
1200	1	0.333333333333333	0	4	
1216	2	1	0	2	
1256	1	0.333333333333333	0	4	
1382	1	0.5	0	4	
1394	1	0.5	0	4	
1439	1	0.333333333333333	0	4	
1445	1	0.333333333333333	0	4	
1457	1	0.333333333333333	0	4	
1465	2	0.666666666666667	0	2	
1468	1	0.333333333333333	0	4	
1477	12	4	0	0	
1478	2	0.666666666666667	0	2	<<<
1488	1	0.333333333333333	0	4	
1537	1	0.333333333333333	0	4	
1557	1	0.5	0	4	
1559	1	0.5	0	4	
1567	1	0.5	0	4	
1580	1	0.5	0	4	
1582	1	0.5	0	4	
1585	1	0.5	0	4	
1590	1	0.5	0	4	

1598	1	0.5	0	4	
1630	1	0.5	0	4	
1632	1	0.5	0	4	
1694	1	0.333333333333333	0	4	
1696	1	0.333333333333333	0	4	
1700	1	0.333333333333333	0	4	
1701	1	0.333333333333333	0	4	
1702	1	0.333333333333333	0	4	
1717	1	0.333333333333333	0	4	
1727	1	0.333333333333333	0	4	
1733	1	0.333333333333333	0	4	
1734	1	0.333333333333333	0	4	
1739	2	1	0	2	
1751	1	0.5	0	4	
1754	1	0.5	0	4	

---

category=0, cleavage_site=1186
query=ptc-miR156g-j, target=Potri.010G154300.1,
score=2.5, range=1175-1195, strand=1

```
target  5' GUGCUCUCUCUcUUCUGUCAG 3'
           ::::::::: ::::::::::.
query   3' CACGAGAGAUAGAAGACAGUU 5'
```

>Potri.010G154300.1
#size=2131

381	1	0.2	0	4	
396	1	0.2	0	4	
407	1	0.2	0	4	
409	1	0.2	0	4	
673	1	0.2	0	4	
893	1	0.5	0	4	
951	1	0.5	0	4	
1185	1	0.5	0	4	
1186	2	1	0	0	<<<
1294	1	0.5	0	4	
1367	1	0.5	0	4	
1384	1	0.5	0	4	
1533	1	0.2	0	4	
1691	1	0.5	0	4	
1771	1	0.5	0	4	

---

category=4, cleavage_site=739
query=ptc-miR156g-j, target=Potri.011G055900.1,
score=2, range=729-748, strand=1

```
target  5' AUGCUCUCUCuCUUCUGUCA 3'
          ::::: :::::::::::::
query   3' CACGAGUGAGAGAAGACAGU 5'
```

>Potri.011G055900.1
#size=864

70	1	1	1	4
71	1	1	1	4
82	2	2	2	2
106	1	1	1	4
121	1	1	1	4
148	1	1	1	4
155	1	1	1	4
167	1	0.5	0	4
187	1	0.5	0	4
192	2	2	2	2

200	1	1	1	4	
201	1	1	1	4	
207	2	2	2	2	
222	1	1	1	4	
229	3	3	3	0	
231	1	1	1	4	
244	1	0.5	0	4	
256	1	1	1	4	
271	1	1	1	4	
279	1	1	1	4	
293	1	1	1	4	
331	1	1	1	4	
354	1	0.5	0	4	
361	1	0.5	0	4	
372	1	0.5	0	4	
373	1	0.5	0	4	
379	1	0.5	0	4	
402	1	1	1	4	
403	1	1	1	4	
406	1	1	1	4	
431	1	1	1	4	
439	1	1	1	4	
456	1	0.5	0	4	
469	1	0.5	0	4	
486	1	0.5	0	4	
491	1	0.5	0	4	
493	1	0.5	0	4	
499	1	1	1	4	
513	1	0.5	0	4	
515	2	1	0	2	
519	1	0.5	0	4	
521	1	0.5	0	4	
532	1	1	1	4	
552	1	1	1	4	
553	2	2	2	2	
555	1	1	1	4	
556	1	1	1	4	
562	1	1	1	4	
593	1	1	1	4	
633	1	1	1	4	
637	2	2	2	2	
653	1	1	1	4	
657	2	2	2	2	
666	1	1	1	4	
669	1	1	1	4	
673	1	1	1	4	
677	1	1	1	4	
691	1	1	1	4	
739	1	1	1	4	<<<
740	1	1	1	4	

---

category=2, cleavage_site=1856
query=ptc-miR156g-j, target=Potri.018G149900.1,
score=2, range=1845-1865, strand=1

```
target  5' GUGCUCUCUCUcUUCUGUCAA 3'
           ::::::::: :::::::::::
query   3' CACGAGAGAUAGAAGACAGUU 5'
```

>Potri.018G149900.1

#size=2752

80	1	0.333333333333333	0	4	
128	1	0.333333333333333	0	4	
201	1	0.5	0	4	
529	1	0.5	0	4	
535	1	0.5	0	4	
582	1	1	1	4	
653	1	0.25	0	4	
737	1	0.25	0	4	
756	1	0.25	0	4	
778	4	1	0	1	
995	1	0.25	0	4	
1145	1	0.25	0	4	
1196	1	0.25	0	4	
1259	1	0.25	0	4	
1356	1	0.25	0	4	
1388	1	0.2	0	4	
1394	1	0.2	0	4	
1422	1	0.25	0	4	
1425	2	0.5	0	2	
1489	1	0.25	0	4	
1583	1	0.25	0	4	
1646	1	0.25	0	4	
1759	1	0.25	0	4	
1762	1	0.25	0	4	
1812	1	0.25	0	4	
1856	3	0.75	0	2	<<<
2319	1	0.25	0	4	
2321	2	0.5	0	2	
2352	1	0.25	0	4	
2380	1	0.25	0	4	
2395	1	0.25	0	4	

ptc-miR156k

category=4, cleavage_site=1392
query=ptc-miR156k, target=Potri.001G055900.1,
score=0.5, range=1382-1401, strand=1

```
target  5' GUGCUCUCUCuCUUCUGUCA 3'
           ::::::.:::::::::::::
query   3' CACGAGGGAGAGAAGACAGU 5'
```

>Potri.001G055900.1
#size=2796

103	1	1	1	4
145	1	1	1	4
163	1	0.2	0	4
169	1	0.2	0	4
171	1	0.2	0	4
172	1	0.2	0	4
187	2	0.4	0	2
263	1	0.2	0	4
284	1	0.2	0	4
683	1	0.2	0	4
728	1	0.2	0	4
825	1	0.1	0	4
835	1	0.1	0	4
872	1	0.1	0	4
914	1	0.111111111111111	0	4

917	2	0.222222222222222	0	2	
930	1	0.1	0	4	
940	3	0.3	0	2	
943	2	0.2	0	2	
944	2	0.2	0	2	
946	2	0.2	0	2	
947	2	0.2	0	2	
949	2	0.2	0	2	
950	1	0.1	0	4	
951	1	0.1	0	4	
952	1	0.1	0	4	
953	1	0.1	0	4	
955	1	0.1	0	4	
956	2	0.2	0	2	
957	2	0.2	0	2	
958	1	0.1	0	4	
959	1	0.1	0	4	
961	2	0.2	0	2	
964	2	0.2	0	2	
965	1	0.1	0	4	
968	1	0.1	0	4	
969	1	0.1	0	4	
972	1	0.1	0	4	
977	2	0.2	0	2	
992	1	0.2	0	4	
1005	1	0.2	0	4	
1021	1	0.2	0	4	
1023	1	0.2	0	4	
1024	1	0.2	0	4	
1026	1	0.2	0	4	
1031	1	0.2	0	4	
1063	1	0.2	0	4	
1067	1	0.2	0	4	
1080	1	0.2	0	4	
1119	1	0.2	0	4	
1141	2	0.4	0	2	
1149	1	0.111111111111111	0	4	
1172	1	0.2	0	4	
1204	1	0.2	0	4	
1227	1	0.2	0	4	
1234	1	0.111111111111111	0	4	
1258	1	0.2	0	4	
1278	1	0.25	0	4	
1290	1	0.25	0	4	
1292	1	0.25	0	4	
1302	1	0.25	0	4	
1304	1	0.25	0	4	
1309	1	0.25	0	4	
1311	1	0.25	0	4	
1320	2	0.5	0	2	
1340	1	0.2	0	4	
1392	1	0.2	0	4	<<<
1419	1	0.2	0	4	
1447	1	0.2	0	4	
1452	1	0.2	0	4	
1500	1	0.2	0	4	
1511	1	0.2	0	4	
1528	1	0.2	0	4	

1599	2	0.25	0	2
1602	1	0.125	0	4
1603	1	0.125	0	4
1616	1	0.2	0	4
1641	1	0.2	0	4
1740	1	0.5	0	4
1766	1	0.5	0	4
1798	1	0.5	0	4
1804	1	0.5	0	4
1822	1	0.5	0	4
1976	1	0.5	0	4
2141	1	0.25	0	4
2142	1	0.25	0	4
2143	1	0.25	0	4
2144	2	0.5	0	2
2168	1	0.25	0	4
2317	1	0.25	0	4
2327	2	0.5	0	2
2334	1	0.25	0	4
2395	1	0.25	0	4
2410	2	0.5	0	2

---

category=0, cleavage_site=1417
query=ptc-miR156k, target=Potri.001G058600.1,
score=0.5, range=1407-1426, strand=1

```
target  5' GUGCUCUCUCuCUUCUGUCA 3'
           ::::::.:::::::::::::
query   3' CACGAGGGAGAGAAGACAGU 5'
```

>Potri.001G058600.1
#size=2079

177	1	1	1	4
344	1	1	1	4
352	1	1	1	4
863	1	1	1	4
924	1	0.333333333333333	0	4
934	1	0.333333333333333	0	4
966	1	0.333333333333333	0	4
973	1	0.333333333333333	0	4
982	1	0.333333333333333	0	4
984	1	0.142857142857143	0	4
1007	2	2	2	2
1013	1	1	1	4
1016	1	1	1	4
1022	1	1	1	4
1024	1	0.333333333333333	0	4
1029	1	0.333333333333333	0	4
1044	1	0.333333333333333	0	4
1059	1	0.333333333333333	0	4
1064	1	0.333333333333333	0	4
1102	1	0.333333333333333	0	4
1140	1	0.333333333333333	0	4
1174	1	1	1	4
1196	1	0.333333333333333	0	4
1201	1	1	1	4
1228	1	1	1	4
1300	1	1	1	4
1379	1	0.333333333333333	0	4
1385	1	0.333333333333333	0	4

1397	1	0.333333333333333	0	4	
1405	2	0.666666666666667	0	3	
1408	1	0.333333333333333	0	4	
1417	12	4	0	0	<<<
1418	2	0.666666666666667	0	3	
1428	1	0.333333333333333	0	4	
1439	1	1	1	4	
1442	1	1	1	4	
1444	1	1	1	4	
1446	1	1	1	4	
1447	3	3	3	2	
1454	1	1	1	4	
1477	1	0.333333333333333	0	4	
1515	1	1	1	4	
1529	1	1	1	4	
1530	1	1	1	4	
1543	1	1	1	4	
1554	1	1	1	4	
1573	1	1	1	4	
1634	1	0.333333333333333	0	4	
1636	1	0.333333333333333	0	4	
1640	1	0.333333333333333	0	4	
1641	1	0.333333333333333	0	4	
1642	1	0.333333333333333	0	4	
1657	1	0.333333333333333	0	4	
1667	1	0.333333333333333	0	4	
1673	1	0.333333333333333	0	4	
1674	1	0.333333333333333	0	4	
1678	1	1	1	4	
1696	1	1	1	4	
1700	1	1	1	4	
1701	1	1	1	4	
1702	2	2	2	2	
1703	2	2	2	2	
1704	1	1	1	4	
1712	1	1	1	4	
1715	2	2	2	2	
1722	1	1	1	4	
1728	1	1	1	4	
1772	1	1	1	4	
1811	2	2	2	2	
1812	1	1	1	4	
1815	1	1	1	4	
1817	1	1	1	4	
1818	2	2	2	2	
1824	1	1	1	4	
1837	1	1	1	4	
1914	1	1	1	4	

category=4, cleavage_site=1034
query=ptc-miR156k, target=Potri.002G142400.1,
score=0.5, range=1024-1043, strand=1

```
target  5' GUGCUCUCUCuCUUCUGUCA 3'
           ::::::.:::::::::::::
query   3' CACGAGGGAGAGAAGACAGU 5'
```

>Potri.002G142400.1
#size=1662

465	1	1	1	4

982	1	0.5	0	4	
1034	1	0.5	0	4	<<<
1193	1	1	1	4	
1335	1	1	1	4	

category=0, cleavage_site=1477
query=ptc-miR156k, target=Potri.003G169400.1,
score=0.5, range=1467-1486, strand=1

```
target  5' GUGCUCUCUCuCUUCUGUCA 3'
           ::::::.:::::::::::::
query   3' CACGAGGGAGAGAAGACAGU 5'
```

>Potri.003G169400.1
#size=2113

57	1	1	1	4	
348	1	1	1	4	
529	1	0.5	0	4	
530	2	1	0	2	
558	1	0.5	0	4	
608	2	1	0	2	
697	1	0.5	0	4	
801	1	0.5	0	4	
984	1	0.333333333333333	0	4	
994	1	0.333333333333333	0	4	
1026	1	0.333333333333333	0	4	
1033	1	0.333333333333333	0	4	
1042	1	0.333333333333333	0	4	
1044	1	0.142857142857143	0	4	
1084	1	0.333333333333333	0	4	
1089	1	0.333333333333333	0	4	
1104	1	0.333333333333333	0	4	
1119	1	0.333333333333333	0	4	
1124	1	0.333333333333333	0	4	
1151	1	0.5	0	4	
1162	1	0.333333333333333	0	4	
1177	1	0.5	0	4	
1180	1	0.5	0	4	
1200	1	0.333333333333333	0	4	
1216	2	1	0	2	
1256	1	0.333333333333333	0	4	
1382	1	0.5	0	4	
1394	1	0.5	0	4	
1439	1	0.333333333333333	0	4	
1445	1	0.333333333333333	0	4	
1457	1	0.333333333333333	0	4	
1465	2	0.666666666666667	0	2	
1468	1	0.333333333333333	0	4	
1477	12	4	0	0	<<<
1478	2	0.666666666666667	0	2	
1488	1	0.333333333333333	0	4	
1537	1	0.333333333333333	0	4	
1557	1	0.5	0	4	
1559	1	0.5	0	4	
1567	1	0.5	0	4	
1580	1	0.5	0	4	
1582	1	0.5	0	4	
1585	1	0.5	0	4	
1590	1	0.5	0	4	
1598	1	0.5	0	4	

1630	1	0.5	0	4
1632	1	0.5	0	4
1694	1	0.333333333333333	0	4
1696	1	0.333333333333333	0	4
1700	1	0.333333333333333	0	4
1701	1	0.333333333333333	0	4
1702	1	0.333333333333333	0	4
1717	1	0.333333333333333	0	4
1727	1	0.333333333333333	0	4
1733	1	0.333333333333333	0	4
1734	1	0.333333333333333	0	4
1739	2	1	0	2
1751	1	0.5	0	4
1754	1	0.5	0	4
1582	1	0.5	0	4
1585	1	0.5	0	4
1590	1	0.5	0	4
1598	1	0.5	0	4
1630	1	0.5	0	4
1632	1	0.5	0	4
1694	1	0.333333333333333	0	4
1696	1	0.333333333333333	0	4
1700	1	0.333333333333333	0	4
1701	1	0.333333333333333	0	4
1702	1	0.333333333333333	0	4
1717	1	0.333333333333333	0	4
1727	1	0.333333333333333	0	4
1733	1	0.333333333333333	0	4
1734	1	0.333333333333333	0	4
1739	2	1	0	2
1751	1	0.5	0	4
1754	1	0.5	0	4

---

category=4, cleavage_site=1087
query=ptc-miR156k, target=Potri.005G099600.1,
score=3.5, range=1078-1096, strand=1

```
target  5' GUGCU-UCUCuCUUCUGCCA 3'
           ::::: .:::::::::: ::
query   3' CACGAGGGAGAGAAGACAGU 5'
```

>Potri.005G099600.1
#size=1672

175	1	0.25	0	4
369	1	0.25	0	4
376	2	1	0	2
393	1	0.5	0	4
442	1	0.25	0	4
495	1	0.125	0	4
512	1	0.25	0	4
558	2	0.333333333333333	0	3
559	1	0.125	0	4
560	5	0.666666666666667	0	2
611	1	0.25	0	4
630	1	0.125	0	4
669	1	0.25	0	4
868	1	0.166666666666667	0	4
878	3	0.375	0	3
879	6	0.75	0	2
915	1	0.166666666666667	0	4

920	1	0.166666666666667	0	4	
929	1	0.166666666666667	0	4	
932	1	0.25	0	4	
959	3	0.75	0	2	
964	1	0.25	0	4	
1016	1	0.25	0	4	
1049	1	0.25	0	4	
1075	1	0.25	0	4	
1079	2	0.285714285714286	0	3	
1087	1	0.25	0	4	<<<
1091	8	2	0	2	
1102	1	0.25	0	4	
1109	1	0.25	0	4	
1113	2	0.5	0	2	
1114	1	0.25	0	4	
1115	3	0.75	0	2	
1116	1	0.25	0	4	
1118	1	0.25	0	4	
1119	1	0.25	0	4	
1120	1	0.25	0	4	
1121	3	0.75	0	2	
1122	2	0.5	0	2	
1123	3	0.75	0	2	
1124	1	0.25	0	4	
1125	4	1	0	2	
1127	1	0.25	0	4	
1130	10	2.5	0	2	
1131	1	0.25	0	4	
1133	1	0.25	0	4	
1134	1	0.25	0	4	
1137	6	1.5	0	2	
1138	8	2	0	2	
1177	2	0.5	0	2	
1184	12	3	0	2	
1185	4	1	0	2	
1202	2	0.5	0	2	
1203	2	0.5	0	2	
1206	14	3.41666666666667	0	2	
1207	13	3.25	0	2	
1208	2	0.5	0	2	
1209	7	1.75	0	2	
1210	19	4.75	0	2	
1211	38	9.5	0	0	
1212	14	3.5	0	2	
1213	1	0.25	0	4	
1215	2	0.5	0	2	
1216	2	0.5	0	2	
1217	11	2.75	0	2	
1219	7	1.75	0	2	
1220	23	5.75	0	2	
1221	15	3.75	0	2	
1222	8	2	0	2	
1223	3	0.75	0	2	
1224	11	3	0	2	
1225	1	0.25	0	4	
1226	1	0.25	0	4	
1227	1	0.25	0	4	
1228	4	1	0	2	

1229	7	1.75	0	2
1230	3	0.75	0	2
1231	6	1.5	0	2
1232	1	0.25	0	4
1233	1	0.25	0	4
1234	2	0.5	0	2
1419	1	0.25	0	4
1425	1	0.25	0	4
1440	3	1.5	0	2
1449	1	0.5	0	4
1493	1	0.5	0	4
1494	4	2	0	2
1497	1	0.5	0	4
1502	2	1	0	2
1503	1	0.5	0	4
1504	1	0.5	0	4
1530	1	0.5	0	4
1540	3	1.5	0	2
1541	1	0.5	0	4
1544	3	1.5	0	2
1564	1	0.5	0	4
1566	1	0.5	0	4
1569	1	0.5	0	4
1571	1	0.5	0	4
1574	2	1	0	2
1575	10	5	0	2
1576	1	0.5	0	4
1577	1	0.5	0	4
1582	7	3.5	0	2
1630	1	0.5	0	4

---

category=0, cleavage_site=994
query=ptc-miR156k, target=Potri.007G138800.1,
score=1.5, range=984-1003, strand=1

```
target  5' UUGCUCUCUCuCUUCUGUCA 3'
            :::::.:::::::::::::
query   3' CACGAGGGAGAGAAGACAGU 5'
```

>Potri.007G138800.1
#size=1410

363	1	0.333333333333333	0	4	
450	1	0.333333333333333	0	4	
550	1	0.333333333333333	0	4	
656	1	0.333333333333333	0	4	
760	1	0.333333333333333	0	4	
766	1	0.333333333333333	0	4	
994	2	0.666666666666667	0	0	<<<

---

category=4, p-value=0.999998500118614, cleavage_site=1225
query=ptc-miR156k, target=Potri.015G098900.1,
score=0.5, range=1215-1234, strand=1

```
target  5' GUGCUCUCUCuCUUCUGUCA 3'
           ::::::.:::::::::::::
query   3' CACGAGGGAGAGAAGACAGU 5'
```

>Potri.015G098900.1
#size=1722

31	1	0.5	0	4
55	1	0.5	0	4
161	1	0.5	0	4

203	1	0.333333333333333	0	4	
223	2	0.666666666666667	0	1	
250	1	0.333333333333333	0	4	
274	1	0.333333333333333	0	4	
276	1	0.333333333333333	0	4	
354	1	0.333333333333333	0	4	
356	1	0.333333333333333	0	4	
365	1	0.333333333333333	0	4	
383	1	0.333333333333333	0	4	
387	1	0.142857142857143	0	4	
395	2	0.666666666666667	0	1	
446	1	0.142857142857143	0	4	
449	1	0.142857142857143	0	4	
481	1	0.333333333333333	0	4	
545	1	0.333333333333333	0	4	
554	1	0.333333333333333	0	4	
596	1	0.333333333333333	0	4	
628	1	0.333333333333333	0	4	
629	1	0.333333333333333	0	4	
639	1	0.142857142857143	0	4	
645	1	0.142857142857143	0	4	
646	1	0.142857142857143	0	4	
692	1	0.333333333333333	0	4	
720	1	0.333333333333333	0	4	
723	1	0.333333333333333	0	4	
726	1	0.333333333333333	0	4	
752	1	0.142857142857143	0	4	
758	1	0.142857142857143	0	4	
786	1	0.142857142857143	0	4	
798	1	0.142857142857143	0	4	
829	1	0.333333333333333	0	4	
851	1	0.333333333333333	0	4	
884	1	0.333333333333333	0	4	
904	1	0.333333333333333	0	4	
1027	1	0.333333333333333	0	4	
1075	1	0.333333333333333	0	4	
1113	1	0.333333333333333	0	4	
1165	1	0.333333333333333	0	4	
1225	1	0.333333333333333	0	4	<<<
1246	1	0.333333333333333	0	4	
1252	2	0.666666666666667	0	1	
1253	2	0.666666666666667	0	1	
1292	1	0.333333333333333	0	4	
1301	1	0.333333333333333	0	4	
1314	1	0.333333333333333	0	4	
1344	1	0.333333333333333	0	4	
1355	1	0.333333333333333	0	4	
1359	1	0.333333333333333	0	4	
1366	1	0.333333333333333	0	4	
1373	1	0.333333333333333	0	4	
1382	1	0.333333333333333	0	4	
1392	1	0.333333333333333	0	4	
1422	1	0.142857142857143	0	4	
1435	1	0.333333333333333	0	4	
1453	1	0.333333333333333	0	4	
1471	1	0.333333333333333	0	4	
1497	1	0.333333333333333	0	4	

ptc-miR159a-c

category=0, cleavage_site=1293
query=ptc-miR159a-c, target=Potri.001G036000.1,
score=3.5, range=1282-1302, strand=1

```
target  5' UGGAGCUCCCUuCACUCCAAU 3'
           :.:::::::::::: :::::
query   3' AUCUCGAGGGAAGUUAGGUUU 5'
```

>Potri.001G036000.1
#size=2405
459 1 0.166666666666667 0 4
704 1 0.1 0 4
1293 10 1.66666666666667 0 0 <<<
1499 1 0.5 0 4
1597 1 0.166666666666667 0 4
1636 1 0.5 0 4
1729 1 0.5 0 4
1788 1 0.5 0 4
1815 1 0.5 0 4
1824 2 0.333333333333333 0 3
1839 2 0.333333333333333 0 3
1843 1 0.166666666666667 0 4
1865 1 0.5 0 4
1893 1 0.5 0 4
1902 1 0.166666666666667 0 4
1907 1 0.5 0 4
1961 1 0.5 0 4
1966 1 0.5 0 4
1980 1 0.166666666666667 0 4
2176 1 0.5 0 4
2232 1 0.5 0 4
2238 1 0.5 0 4
2285 1 0.5 0 4

category=2, cleavage_site=1375
query=ptc-miR159a-c, target=Potri.001G224500.1,
score=3, range=1364-1384, strand=1

```
target  5' UAGAGCUCCCUuCACUCCAAU 3'
           :::::::::::::: :::::
query   3' AUCUCGAGGGAAGUUAGGUUU 5'
```

>Potri.001G224500.1
#size=2302
119 1 0.333333333333333 0 4
289 1 0.333333333333333 0 4
370 1 0.333333333333333 0 4
514 11 2.75 0 0
519 1 0.25 0 4
771 1 0.1 0 4
1375 2 0.5 0 2 <<<
1434 1 0.25 0 4
1613 1 0.333333333333333 0 4
1721 1 0.333333333333333 0 4
1722 2 0.666666666666667 0 2
1725 1 0.333333333333333 0 4
1727 1 0.333333333333333 0 4
1838 1 0.25 0 4
1931 1 0.333333333333333 0 4
1932 2 0.666666666666667 0 2

1934 3 1 0 2
1935 3 1 0 2
1986 1 0.333333333333333 0 4
1987 2 0.666666666666667 0 2
1993 1 0.333333333333333 0 4
1999 1 0.333333333333333 0 4
2007 1 0.333333333333333 0 4
2014 1 0.333333333333333 0 4
2085 1 0.333333333333333 0 4

---

category=0, cleavage_site=1269
query=ptc-miR159a-c, target=Potri.003G189700.1,
score=4, range=1258-1278, strand=1

```
target  5' UUGAGCUCCCUuCACUCCAAU 3'
           : :::::::::::: :::::
query   3' AUCUCGAGGGAAGUUAGGUUU 5'
```

>Potri.003G189700.1
#size=2435
435 1 0.166666666666667 0 4
1269 10 1.6666666666667 0 0 <<<
1352 1 0.25 0 4
1499 1 0.25 0 4
1541 1 0.25 0 4
1573 1 0.166666666666667 0 4
1646 1 1 1 4
1648 1 1 1 4
1713 1 0.25 0 4
1725 1 0.25 0 4
1789 1 0.25 0 4
1800 2 0.333333333333333 0 2
1815 2 0.333333333333333 0 2
1819 1 0.166666666666667 0 4
1822 1 0.25 0 4
1834 1 0.25 0 4
1855 1 0.25 0 4
1875 1 0.25 0 4
1878 1 0.166666666666667 0 4
1909 3 0.75 0 2
1916 1 0.25 0 4
1927 1 0.25 0 4
1929 1 0.25 0 4
1956 1 0.166666666666667 0 4
2003 1 0.333333333333333 0 4
2009 1 0.333333333333333 0 4
2028 1 0.333333333333333 0 4
2082 1 0.333333333333333 0 4
2086 1 0.333333333333333 0 4
2088 1 0.333333333333333 0 4
2089 2 0.666666666666667 0 2
2094 1 0.333333333333333 0 4
2098 1 0.333333333333333 0 4
2103 1 0.333333333333333 0 4
2104 1 0.333333333333333 0 4
2105 1 0.333333333333333 0 4
2106 1 0.333333333333333 0 4
2210 1 0.333333333333333 0 4

category=3, cleavage_site=1587
query=ptc-miR159a-c, target=Potri.009G018700.1,
score=3, range=1576-1596, strand=1

```
target  5' UAGAGCUCCCUuCACUCCAAU 3'
           :::::::::::::: :::::
query   3' AUCUCGAGGGAAGUUAGGUUU 5'
```

>Potri.009G018700.1
#size=2473

837	11	2.75	0	0	
842	1	0.25	0	4	
1587	2	0.5	0	3	<<<
1591	1	1	1	4	
1646	1	0.25	0	4	
1756	1	1	1	4	
1786	1	1	1	4	
1930	1	1	1	4	
1932	1	1	1	4	
1933	1	1	1	4	
1936	2	2	2	2	
2009	1	1	1	4	
2050	1	0.25	0	4	
2141	1	1	1	4	
2166	2	2	2	2	
2189	1	1	1	4	
2220	1	1	1	4	
2221	1	1	1	4	
2233	1	1	1	4	
2241	1	1	1	4	
2245	1	1	1	4	

ptc-miR160a-d

category=0, cleavage_site=1322
query=ptc-miR160a-d, target=Potri.002G089900.1,
score=0.5, range=1311-1331, strand=1

```
target  5' UGGCAUGCAGGgAGCCAGGCA 3'
           ::::::.::::::::::::::
query   3' ACCGUAUGUCCCUCGGUCCGU 5'
```

>Potri.002G089900.1
#size=2124

1322	7	1.7	0	0	<<<
1455	1	0.2	0	4	
1505	1	0.5	0	4	
1708	1	0.2	0	4	
1713	1	0.2	0	4	
1807	1	1	1	4	
1814	1	1	1	4	
1873	1	1	1	4	

category=0, cleavage_site=1729
query=ptc-miR160a-d, target=Potri.004G211700.1,
score=1, range=1718-1738, strand=1

```
target  5' AGGCAUACAGGgAGCCAGGCA 3'
            ::::::::::::::::::::
query   3' ACCGUAUGUCCCUCGGUCCGU 5'
```

>Potri.004G211700.1
#size=2722

236	1	1	1	4			
885	1	0.333333333333333			0	4	
1238	1	0.333333333333333			0	4	
1729	20	11.3333333333333			7	0	<<<
1841	1	1	1	4			
2126	1	1	1	4			
2134	1	0.333333333333333			0	4	
2188	2	2	2	2			
2301	1	1	1	4			
2303	1	1	1	4			
2309	1	1	1	4			
2311	1	1	1	4			
2312	2	2	2	2			
2315	1	1	1	4			
2318	2	2	2	2			
2323	1	1	1	4			
2349	2	0.666666666666667			0	3	
2351	3	1	0	2			
2352	3	1	0	2			
2353	1	0.333333333333333			0	4	
2354	1	0.333333333333333			0	4	
2362	1	0.333333333333333			0	4	
2367	1	0.333333333333333			0	4	
2420	1	1	1	4			
2421	2	2	2	2			
2422	1	1	1	4			
2423	1	1	1	4			
2424	1	1	1	4			
2425	1	1	1	4			
2435	1	1	1	4			
2437	1	1	1	4			
2442	1	1	1	4			
2444	1	1	1	4			
2450	1	0.5	0	4			
2458	1	0.5	0	4			
2460	1	0.5	0	4			
2461	1	0.5	0	4			
2463	3	1.5	0	2			
2464	1	0.5	0	4			
2465	1	0.5	0	4			
2466	2	1	0	2			
2468	2	1	0	2			
2474	1	0.5	0	4			
2480	1	1	1	4			
2491	1	1	1	4			
2496	1	1	1	4			
2497	1	1	1	4			
2498	3	3	3	2			
2499	2	2	2	2			
2508	1	1	1	4			
2509	1	1	1	4			
2536	1	1	1	4			
2582	1	1	1	4			
2587	1	1	1	4			
2607	2	2	2	2			
2612	1	1	1	4			

category=0, p-value=0.036594165225991, cleavage_site=1610
query=ptc-miR160a-d, target=Potri.005G171300.1,
score=0.5, range=1599-1619, strand=1

```
target  5' UGGCAUGCAGGgAGCCAGGCA 3'
           ::::::.::::::::::::::
query   3' ACCGUAUGUCCCUCGGUCCGU 5'
```

>Potri.005G171300.1
#size=2501

704	1	0.333333333333333	0	4	
794	1	0.333333333333333	0	4	
860	1	0.333333333333333	0	4	
869	1	0.333333333333333	0	4	
1040	1	0.333333333333333	0	4	
1055	1	0.333333333333333	0	4	
1610	6	1.2	0	0	<<<
1677	2	0.666666666666667	0	2	
1694	1	0.333333333333333	0	4	
1743	1	0.2	0	4	
1799	1	0.333333333333333	0	4	
1996	1	0.2	0	4	
2001	1	0.2	0	4	
2008	1	0.333333333333333	0	4	
2014	1	0.333333333333333	0	4	
2015	1	0.333333333333333	0	4	
2086	1	0.5	0	4	
2123	1	0.5	0	4	
2219	1	0.5	0	4	

category=0, cleavage_site=2267
query=ptc-miR160a-d, target=Potri.006G127500.1,
score=0.5, range=2256-2276, strand=1

```
target  5' UGGCAUGCAGGgAGCCAGGCA 3'
           ::::::.::::::::::::::
query   3' ACCGUAUGUCCCUCGGUCCGU 5'
```

>Potri.006G127500.1
#size=3486

619	1	0.5	0	4	
626	1	0.5	0	4	
1234	2	1	0	2	
1393	2	0.4	0	3	
1509	1	0.5	0	4	
1528	1	0.2	0	4	
1653	1	0.5	0	4	
1705	1	0.5	0	4	
1952	1	0.5	0	4	
2050	1	0.2	0	4	
2115	1	0.2	0	4	
2123	1	0.2	0	4	
2139	1	0.5	0	4	
2267	66	13.2	0	0	<<<
2268	1	0.2	0	4	
2286	1	0.2	0	4	
2324	1	0.5	0	4	
2349	1	0.5	0	4	
2368	1	0.5	0	4	
2643	1	0.5	0	4	
2674	2	1	0	2	

2701	1	0.5	0	4
2702	1	0.5	0	4
2709	1	0.5	0	4
2720	1	0.5	0	4
2737	1	0.5	0	4
2775	1	0.5	0	4
2797	1	0.5	0	4
2815	1	0.2	0	4
2833	1	0.5	0	4
2890	1	0.5	0	4
3332	1	1	1	4

---

category=0, cleavage_site=1817
query=ptc-miR160a-d, target=Potri.008G039000.1, 、
score=1, range=1806-1826, strand=1

```
target  5' AGGCAUACAGGgAGCCAGGCA 3'
              :::::::::::::::::::: 
query   3' ACCGUAUGUCCCUCGGUCCGU 5'
```

>Potri.008G039000.1
#size=2772

136	1	1	1	4	
961	1	0.5	0	4	
970	1	0.333333333333333	0	4	
986	1	0.333333333333333	0	4	
1124	1	0.5	0	4	
1125	1	0.5	0	4	
1343	1	0.5	0	4	
1365	1	0.5	0	4	
1502	1	0.5	0	4	
1530	1	1	1	4	
1584	1	1	1	4	
1590	2	2	2	2	
1592	1	1	1	4	
1714	1	1	1	4	
1729	1	1	1	4	
1817	19	19	19	0	<<<
1818	1	0.5	0	4	
1853	1	0.5	0	4	
1941	2	2	2	2	
1964	1	0.5	0	4	
1965	1	0.5	0	4	
2149	1	1	1	4	
2193	1	1	1	4	
2247	1	1	1	4	
2255	2	2	2	2	
2272	2	1.5	1	2	
2276	1	1	1	4	
2281	1	0.5	0	4	
2288	1	1	1	4	
2293	1	0.5	0	4	
2294	1	0.5	0	4	
2299	1	0.5	0	4	
2300	1	0.5	0	4	
2305	1	0.5	0	4	
2309	2	1	0	2	
2328	1	0.5	0	4	
2331	6	5.5	5	2	
2335	1	1	1	4	

2336	2	2	2	2
2339	1	1	1	4
2340	3	1.5	0	2
2341	1	0.5	0	4
2359	1	1	1	4
2368	1	0.5	0	4
2373	2	1	0	2
2374	1	0.5	0	4
2375	3	1.5	0	2
2384	2	1.5	1	2
2387	1	0.5	0	4
2389	2	1	0	2
2396	1	0.5	0	4
2397	1	0.5	0	4
2404	1	0.5	0	4
2413	1	1	1	4
2414	1	0.5	0	4
2418	1	1	1	4
2427	2	2	2	2
2428	2	1	0	2
2429	1	0.5	0	4
2430	1	0.5	0	4
2431	5	3.5	2	2
2432	5	4	3	2
2433	1	1	1	4
2524	1	1	1	4
2536	1	1	1	4
2550	3	3	3	2
2551	1	1	1	4
2653	1	0.5	0	4
2655	1	0.5	0	4

---

category=0, cleavage_site=1418
query=ptc-miR160a-d, target=Potri.009G014800.1,
score=1, range=1407-1427, strand=1

```
target  5' AGGCAUACAGGgAGCCAGGCA 3'
           :::::::::::::::::::::
query   3' ACCGUAUGUCCCUCGGUCCGU 5'
```

>Potri.009G014800.1
#size=2450

17	1	0.5	0	4	
330	1	0.5	0	4	
577	1	0.333333333333333	0	4	
830	1	0.5	0	4	
833	1	0.5	0	4	
927	1	0.333333333333333	0	4	
1171	1	0.5	0	4	
1418	13	4.33333333333333	0	0	<<<
1668	2	1	0	2	
1705	1	0.5	0	4	
1829	1	0.333333333333333	0	4	
1926	1	0.5	0	4	
1931	1	0.5	0	4	
2001	1	0.5	0	4	
2020	1	0.5	0	4	
2044	2	0.666666666666667	0	2	
2046	3	1	0	2	

2047	3	1	0	2	
2048	1	0.333333333333333	0	4	
2049	1	0.333333333333333	0	4	
2057	1	0.333333333333333	0	4	
2062	1	0.333333333333333	0	4	
2140	1	0.5	0	4	
2148	1	0.5	0	4	
2150	1	0.5	0	4	
2151	1	0.5	0	4	
2153	3	1.5	0	2	
2154	1	0.5	0	4	
2155	1	0.5	0	4	
2156	2	1	0	2	
2158	2	1	0	2	
2161	1	1	1	4	
2164	1	0.5	0	4	
2196	1	1	1	4	

---

category=0, cleavage_site=1836
query=ptc-miR160a-d, target=Potri.010G223200.1,
score=1, range=1825-1845, strand=1

```
target  5' AGGCAUACAGGgAGCCAGGCA 3'
              ::::::::::::::::::::
query   3' ACCGUAUGUCCCUCGGUCCGU 5'
```

>Potri.010G223200.1
#size=2903

983	1	0.5	0	4	
992	1	0.333333333333333	0	4	
1008	1	0.333333333333333	0	4	
1146	1	0.5	0	4	
1147	1	0.5	0	4	
1362	1	0.5	0	4	
1384	1	0.5	0	4	
1521	1	0.5	0	4	
1596	1	1	1	4	
1835	1	1	1	4	
1836	23	23	23	0	<<<
1837	1	0.5	0	4	
1857	1	1	1	4	
1867	1	1	1	4	
1872	1	0.5	0	4	
1885	1	1	1	4	
1942	1	1	1	4	
1949	1	1	1	4	
1957	1	1	1	4	
1983	1	0.5	0	4	
1984	1	0.5	0	4	
2185	1	1	1	4	
2229	1	1	1	4	
2230	1	1	1	4	
2288	1	0.5	0	4	
2297	1	0.5	0	4	
2304	1	1	1	4	
2309	1	0.5	0	4	
2310	1	0.5	0	4	
2315	1	0.5	0	4	
2316	1	0.5	0	4	
2321	1	0.5	0	4	

2325	2	1	0	2
2344	1	0.5	0	4
2347	1	0.5	0	4
2356	3	1.5	0	2
2357	1	0.5	0	4
2384	1	0.5	0	4
2389	2	1	0	2
2390	1	0.5	0	4
2391	3	1.5	0	2
2400	1	0.5	0	4
2403	1	0.5	0	4
2405	2	1	0	2
2412	1	0.5	0	4
2413	1	0.5	0	4
2420	1	0.5	0	4
2430	1	0.5	0	4
2432	1	1	1	4
2443	1	1	1	4
2444	4	3	2	2
2445	1	0.5	0	4
2446	1	0.5	0	4
2447	3	1.5	0	2
2448	3	2	1	2
2450	1	1	1	4
2488	1	1	1	4
2505	1	1	1	4
2514	1	1	1	4
2521	1	1	1	4
2522	1	1	1	4
2523	1	1	1	4
2566	6	6	6	2
2568	1	1	1	4
2570	1	1	1	4
2603	1	1	1	4
2605	1	1	1	4
2617	1	1	1	4
2630	1	1	1	4
2635	2	2	2	2
2668	1	1	1	4
2693	1	0.5	0	4
2695	1	0.5	0	4

---

category=0, cleavage_site=2199
query=ptc-miR160a-d, target=Potri.016G090300.1,
score=0.5, range=2188-2208, strand=1

```
target  5' UGGCAUGCAGGgAGCCAGGCA 3'
           ::::::.::::::::::::::
query   3' ACCGUAUGUCCCUCGGUCCGU 5'
```

>Potri.016G090300.1
#size=3464

802	1	1	1	4
818	1	1	1	4
1271	1	0.333333333333333	0	4
1294	1	0.333333333333333	0	4
1325	2	0.4	0	2
1460	1	0.2	0	4
1493	1	0.333333333333333	0	4
1877	1	0.333333333333333	0	4

1982	1	0.2	0	4	
2047	1	0.2	0	4	
2055	1	0.2	0	4	
2059	1	0.333333333333333	0	4	
2199	66	13.2	0	0	<<<
2200	1	0.2	0	4	
2218	1	0.2	0	4	
2229	1	0.333333333333333	0	4	
2315	1	0.333333333333333	0	4	
2322	1	0.333333333333333	0	4	
2323	1	0.333333333333333	0	4	
2528	1	0.333333333333333	0	4	
2632	1	0.333333333333333	0	4	
2645	2	0.666666666666667	0	2	
2677	1	0.333333333333333	0	4	
2739	2	0.666666666666667	0	2	
2747	1	0.2	0	4	
2751	1	0.333333333333333	0	4	
2754	2	0.666666666666667	0	2	
2758	1	0.333333333333333	0	4	
2838	1	1	1	4	
2841	2	2	2	2	
2847	1	1	1	4	
2873	1	0.333333333333333	0	4	
2969	3	1	0	2	
3046	1	0.333333333333333	0	4	
3098	1	0.333333333333333	0	4	
3117	1	0.333333333333333	0	4	

ptc-miR162a-c

---

category=0, cleavage_site=4168
query=ptc-miR162a-c, target=Potri.002G181400.1,
score=2, range=4157-4178, strand=1

```
target  5' CUGGAUGCAGAgGUCUUAUCGA 3'
           ::::::::::::: :::::::
query   3' GACCUACGUCUCCA-AAUAGCU 5'
```

>Potri.002G181400.1
#size=7349

60	1	0.2	0	4
61	2	2	2	2
370	1	1	1	4
384	1	1	1	4
396	1	1	1	4
420	1	1	1	4
422	1	1	1	4
425	1	1	1	4
429	1	1	1	4
434	1	1	1	4
435	1	1	1	4
437	1	1	1	4
439	1	1	1	4
440	1	1	1	4
443	1	1	1	4
482	1	1	1	4
542	1	1	1	4
791	1	1	1	4
1072	1	1	1	4

1074	1	1	1	4
1134	1	1	1	4
1136	1	1	1	4
1149	1	1	1	4
1179	1	1	1	4
1181	1	1	1	4
1196	1	1	1	4
1249	1	1	1	4
1298	1	1	1	4
1346	1	1	1	4
1359	1	1	1	4
1397	1	1	1	4
1417	1	1	1	4
1421	1	1	1	4
1425	1	1	1	4
1431	1	1	1	4
1470	1	1	1	4
1471	1	1	1	4
1516	1	1	1	4
1547	1	1	1	4
1566	1	1	1	4
1573	1	1	1	4
1583	1	1	1	4
1584	1	1	1	4
1592	1	1	1	4
1599	1	1	1	4
1601	1	1	1	4
1602	1	1	1	4
1622	1	1	1	4
1638	1	1	1	4
1641	1	1	1	4
1655	1	1	1	4
1679	1	1	1	4
1691	1	1	1	4
1701	1	1	1	4
1728	1	1	1	4
1744	1	1	1	4
1751	1	1	1	4
1757	1	1	1	4
1760	1	1	1	4
1930	1	1	1	4
1941	1	1	1	4
2095	1	1	1	4
2109	1	1	1	4
2113	1	1	1	4
2117	4	4	4	2
2266	1	1	1	4
2292	1	1	1	4
2307	1	1	1	4
2310	2	2	2	2
2389	2	2	2	2
2391	1	1	1	4
2416	1	1	1	4
2438	1	1	1	4
2444	1	1	1	4
2458	1	1	1	4
2466	1	1	1	4
2493	1	1	1	4

2546	1	1	1	4	
2553	1	1	1	4	
2558	1	1	1	4	
2564	2	2	2	2	
2595	1	1	1	4	
2606	1	1	1	4	
2615	1	1	1	4	
2897	1	1	1	4	
2902	2	2	2	2	
2908	1	1	1	4	
2915	1	1	1	4	
2934	1	1	1	4	
3057	1	1	1	4	
3136	1	1	1	4	
3137	1	1	1	4	
3205	1	1	1	4	
3236	1	1	1	4	
3477	1	1	1	4	
3755	1	1	1	4	
3787	1	1	1	4	
3870	1	1	1	4	
4068	1	1	1	4	
4112	2	2	2	2	
4167	1	1	1	4	
4168	5	5	5	0	<<<
4416	1	1	1	4	
4481	1	1	1	4	
4583	1	1	1	4	
4622	1	1	1	4	
4633	1	1	1	4	
4639	1	1	1	4	
4646	1	1	1	4	
4647	1	1	1	4	
4655	1	1	1	4	
4744	1	1	1	4	
4769	1	1	1	4	
5047	1	1	1	4	
5111	1	1	1	4	
5139	1	1	1	4	
5168	1	1	1	4	
5170	3	3	3	2	
5209	1	1	1	4	
5232	1	1	1	4	
5247	1	1	1	4	
5248	2	2	2	2	
5250	1	1	1	4	
5252	2	2	2	2	
5266	1	1	1	4	
5288	1	1	1	4	
5315	1	1	1	4	
5358	1	1	1	4	
5386	1	1	1	4	
5398	1	1	1	4	
5444	1	1	1	4	
5464	1	1	1	4	
5510	1	1	1	4	
5511	1	1	1	4	
5532	1	1	1	4	

5535	1	1	1	4
5553	1	1	1	4
5573	1	1	1	4
5579	1	1	1	4
5681	1	1	1	4
5764	2	2	2	2
5769	1	1	1	4
5780	1	1	1	4
5798	1	1	1	4
5810	1	1	1	4
5841	1	1	1	4
5849	1	1	1	4
6031	1	1	1	4
6042	2	2	2	2
6114	1	1	1	4
6159	1	1	1	4
6164	2	2	2	2
6188	1	1	1	4
6197	1	1	1	4
6199	1	1	1	4
6245	1	1	1	4
6247	1	1	1	4
6249	1	1	1	4
6251	1	1	1	4
6252	1	1	1	4
6257	1	1	1	4
6280	1	1	1	4
6284	1	1	1	4
6298	1	1	1	4
6302	1	1	1	4
6308	1	1	1	4
6318	1	1	1	4
6347	1	1	1	4
6360	1	1	1	4
6398	1	1	1	4
6408	1	1	1	4
6411	1	1	1	4
6434	1	1	1	4
6443	1	1	1	4
6501	1	1	1	4
6506	1	1	1	4
6519	1	1	1	4
6520	2	2	2	2
6562	1	1	1	4
6566	1	1	1	4
6620	1	1	1	4
6627	3	3	3	2
6628	2	2	2	2
6631	1	1	1	4
6636	1	1	1	4
6643	1	1	1	4
6647	1	1	1	4
6658	1	1	1	4
6666	1	1	1	4
6706	1	1	1	4
6717	1	1	1	4
6747	2	2	2	2
6787	1	1	1	4

6807	1	1	1	4
6809	1	1	1	4
6812	1	1	1	4
6956	2	2	2	2
6963	1	1	1	4
6964	1	1	1	4
7031	1	1	1	4
7053	1	1	1	4
7067	1	1	1	4
7102	1	1	1	4
7171	1	1	1	4

ptc-miR164a-e

category=0, cleavage_site=784
query=ptc-miR164a-e, target=Potri.005G098200.1,
score=2, range=773-793, strand=1

```
target  5' AGCAAGUGCCCuGCUUCUCCA 3'
             ::: :::::::::::::::
query   3' ACGUGCACGGGACGAAGAGGU 5'
```

>Potri.005G098200.1
#size=1411

221	1	0.2	0	4	
614	1	0.333333333333333	0	4	
771	1	0.2	0	4	
784	5	1.66666666666667	0	0	<<<
989	1	0.2	0	4	
1234	1	0.333333333333333	0	4	

category=0, cleavage_site=730
query=ptc-miR164a-e, target=Potri.007G065400.1,
score=2, range=719-739, strand=1

```
target  5' AGCAAGUGCCCuGCUUCUCCA 3'
             ::: :::::::::::::::
query   3' ACGUGCACGGGACGAAGAGGU 5'
```

>Potri.007G065400.1
#size=1331

136	1	0.5	0	4	
161	1	0.2	0	4	
261	1	1	1	4	
322	1	0.5	0	4	
392	1	0.5	0	4	
627	1	0.5	0	4	
717	1	0.2	0	4	
730	35	17.5	0	0	<<<
827	1	0.5	0	4	
858	2	1	0	2	
921	1	0.5	0	4	
929	1	0.2	0	4	
969	1	0.5	0	4	
1151	1	0.5	0	4	

category=4, cleavage_site=671
query=ptc-miR164a-e, target=Potri.010G207200.1,
score=4, range=660-680, strand=1

```
target  5' CUUGCUUGCCCuGCUUCUCCA 3'
              ..: ::::::::::::::
query   3' ACGUGCACGGGACGAAGAGGU 5'
```

>Potri.010G207200.1
#size=1628

332	1	0.5	0	4
333	1	0.333333333333333	0	4
353	1	0.2	0	4
360	1	0.2	0	4
362	2	0.4	0	2
364	1	0.2	0	4
369	1	0.2	0	4
378	4	0.8	0	2
381	1	0.2	0	4
399	2	0.4	0	2
409	1	0.2	0	4
411	1	0.2	0	4
413	1	0.2	0	4
422	1	0.2	0	4
423	2	0.4	0	2
424	1	0.2	0	4
444	1	0.1	0	4
459	1	0.2	0	4
463	1	0.2	0	4
483	1	0.1	0	4
503	1	0.1	0	4
506	1	0.1	0	4
507	1	0.1	0	4
509	1	0.1	0	4
510	1	0.1	0	4
511	2	0.2	0	2
513	1	0.1	0	4
515	1	0.1	0	4
516	3	0.3	0	2
518	2	0.2	0	2
519	2	0.3	0	2
520	1	0.2	0	4
522	1	0.1	0	4
524	2	0.2	0	2
525	1	0.1	0	4
526	1	0.2	0	4
527	1	0.2	0	4
528	1	0.2	0	4
529	1	0.2	0	4
530	1	0.1	0	4
531	3	0.4	0	2
533	1	0.2	0	4
536	1	0.1	0	4
539	1	0.1	0	4
540	3	0.3	0	2
542	1	0.1	0	4
544	2	0.2	0	2
547	2	0.2	0	2
550	1	0.1	0	4
553	1	0.1	0	4
557	1	0.1	0	4
561	2	0.2	0	2
563	1	0.1	0	4
566	1	0.1	0	4
567	1	0.1	0	4
570	1	0.1	0	4

571	2	0.2	0	2	
573	3	0.3	0	2	
575	1	0.1	0	4	
576	2	0.2	0	2	
578	1	0.1	0	4	
579	1	0.1	0	4	
582	1	0.1	0	4	
585	1	0.1	0	4	
588	1	0.1	0	4	
589	2	0.2	0	2	
591	1	0.1	0	4	
592	1	0.1	0	4	
594	1	0.1	0	4	
596	1	0.1	0	4	
597	1	0.1	0	4	
599	1	0.1	0	4	
601	1	0.1	0	4	
602	1	0.1	0	4	
603	1	0.1	0	4	
608	2	0.2	0	2	
610	2	0.4	0	2	
611	1	0.1	0	4	
612	1	0.1	0	4	
613	2	0.2	0	2	
614	1	0.1	0	4	
619	3	0.3	0	2	
620	2	0.2	0	2	
621	2	0.3	0	2	
622	2	0.2	0	2	
624	2	0.2	0	2	
627	1	0.1	0	4	
634	1	0.1	0	4	
637	1	0.1	0	4	
639	1	0.1	0	4	
647	1	0.1	0	4	
648	2	0.2	0	2	
650	3	0.3	0	2	
651	2	0.2	0	2	
660	1	0.1	0	4	
661	1	0.1	0	4	
663	1	0.1	0	4	
671	1	0.1	0	4	<<<
672	1	0.1	0	4	
673	2	0.2	0	2	
674	1	0.1	0	4	
681	1	0.1	0	4	
685	2	0.2	0	2	
688	1	0.1	0	4	
690	1	0.1	0	4	
691	2	0.2	0	2	
692	2	0.3	0	2	
696	3	0.3	0	2	
697	1	0.1	0	4	
698	1	0.1	0	4	
699	5	0.5	0	2	
700	3	0.3	0	2	
702	2	0.2	0	2	
703	3	0.3	0	2	

704	1	0.1	0	4
707	2	0.2	0	2
708	2	0.2	0	2
709	1	0.1	0	4
710	2	0.2	0	2
711	3	0.3	0	2
712	2	0.2	0	2
714	1	0.1	0	4
715	1	0.1	0	4
716	2	0.2	0	2
720	2	0.2	0	2
722	1	0.1	0	4
724	3	0.3	0	2
727	1	0.1	0	4
728	1	0.1	0	4
730	1	0.1	0	4
731	4	0.4	0	2
732	1	0.1	0	4
733	1	0.1	0	4
734	1	0.1	0	4
736	1	0.1	0	4
737	4	0.4	0	2
738	1	0.1	0	4
739	1	0.1	0	4
740	5	0.5	0	2
742	1	0.1	0	4
743	3	0.3	0	2
744	2	0.2	0	2
745	2	0.2	0	2
747	1	0.1	0	4
748	6	0.6	0	2
749	3	0.3	0	2
751	5	0.5	0	2
752	1	0.1	0	4
753	1	0.1	0	4
754	5	0.5	0	2
755	3	0.3	0	2
756	2	0.2	0	2
757	1	0.1	0	4
758	4	0.4	0	2
759	1	0.2	0	4
760	2	0.4	0	2
762	2	0.4	0	2
768	4	0.4	0	2
769	2	0.2	0	2
770	3	0.3	0	2
771	4	0.5	0	2
772	1	0.1	0	4
773	1	0.1	0	4
774	1	0.1	0	4
777	1	0.1	0	4
778	2	0.2	0	2
779	5	0.5	0	2
780	4	0.8	0	2
781	1	0.2	0	4
782	1	0.2	0	4
784	2	0.4	0	2
788	1	0.2	0	4

794	1	0.2	0	4
796	1	0.2	0	4
798	1	0.2	0	4
799	1	0.2	0	4
800	1	0.2	0	4
808	2	0.4	0	2
812	1	0.2	0	4
813	1	0.2	0	4
816	1	0.2	0	4
817	1	0.1	0	4
819	2	0.2	0	2
821	1	0.1	0	4
822	1	0.1	0	4
825	2	0.2	0	2
826	3	0.3	0	2
827	2	0.2	0	2
828	3	0.3	0	2
829	2	0.2	0	2
830	1	0.1	0	4
831	1	0.1	0	4
832	2	0.2	0	2
833	1	0.1	0	4
834	3	0.3	0	2
835	3	0.3	0	2
836	1	0.1	0	4
837	1	0.1	0	4
838	1	0.1	0	4
839	1	0.1	0	4
840	1	0.1	0	4
841	1	0.1	0	4
842	1	0.1	0	4
843	1	0.1	0	4
844	1	0.1	0	4
846	1	0.1	0	4
847	2	0.2	0	2
850	2	0.2	0	2
851	1	0.1	0	4
852	1	0.1	0	4
853	2	0.2	0	2
856	1	0.1	0	4
857	1	0.1	0	4
860	2	0.2	0	2
862	3	0.3	0	2
863	3	0.3	0	2
864	1	0.1	0	4
865	1	0.1	0	4
866	1	0.1	0	4
867	2	0.2	0	2
869	1	0.1	0	4
870	2	0.2	0	2
873	2	0.2	0	2
874	1	0.1	0	4
875	2	0.2	0	2
876	2	0.2	0	2
877	1	0.1	0	4
878	1	0.1	0	4
879	1	0.1	0	4
880	2	0.2	0	2

881	1	0.1	0	4
882	3	0.3	0	2
884	6	0.6	0	2
885	1	0.1	0	4
886	1	0.1	0	4
887	1	0.1	0	4
888	1	0.1	0	4
889	1	0.1	0	4
890	1	0.1	0	4
891	3	0.3	0	2
892	2	0.2	0	2
893	1	0.1	0	4
894	2	0.2	0	2
895	3	0.3	0	2
898	1	0.1	0	4
899	1	0.1	0	4
900	3	0.4	0	2
901	1	0.2	0	4
902	4	0.6	0	2
905	1	0.2	0	4
908	4	0.4	0	2
910	1	0.1	0	4
911	4	0.8	0	2
912	1	0.1	0	4
913	4	0.6	0	2
914	1	0.1	0	4
915	1	0.1	0	4
917	2	0.2	0	2
918	5	0.5	0	2
919	3	0.3	0	2
920	3	0.3	0	2
921	1	0.2	0	4
924	1	0.1	0	4
925	3	0.3	0	2
926	1	0.1	0	4
927	4	0.4	0	2
928	1	0.1	0	4
929	3	0.3	0	2
930	6	0.6	0	2
931	2	0.2	0	2
932	1	0.1	0	4
933	2	0.2	0	2
934	10	1	0	2
935	3	0.3	0	2
936	3	0.3	0	2
937	1	0.1	0	4
939	4	0.4	0	2
940	2	0.2	0	2
941	3	0.3	0	2
942	4	0.4	0	2
944	1	0.1	0	4
945	4	0.4	0	2
949	1	0.1	0	4
951	5	0.5	0	2
952	2	0.2	0	2
955	2	0.2	0	2
957	1	0.1	0	4
958	2	0.2	0	2

959	1	0.1	0	4
961	1	0.1	0	4
963	2	0.2	0	2
965	3	0.3	0	2
966	1	0.1	0	4
967	2	0.2	0	2
969	2	0.2	0	2
972	3	0.3	0	2
973	2	0.2	0	2
974	2	0.2	0	2
976	1	0.1	0	4
977	2	0.2	0	2
978	2	0.2	0	2
979	3	0.3	0	2
982	3	0.3	0	2
983	2	0.2	0	2
985	3	0.3	0	2
986	3	0.3	0	2
987	4	0.4	0	2
988	5	0.5	0	2
989	2	0.2	0	2
990	3	0.3	0	2
991	2	0.2	0	2
992	1	0.1	0	4
993	6	0.6	0	2
995	4	0.4	0	2
996	2	0.2	0	2
997	1	0.1	0	4
999	1	0.1	0	4
1000	1	0.1	0	4
1002	2	0.2	0	2
1004	2	0.2	0	2
1005	1	0.1	0	4
1006	1	0.2	0	4
1007	1	0.1	0	4
1008	1	0.1	0	4
1009	1	0.1	0	4
1010	1	0.1	0	4
1013	2	0.2	0	2
1014	1	0.1	0	4
1016	1	0.1	0	4
1017	2	0.2	0	2
1018	1	0.1	0	4
1019	1	0.1	0	4
1020	1	0.1	0	4
1021	2	0.2	0	2
1022	1	0.1	0	4
1025	2	0.2	0	2
1027	2	0.2	0	2
1029	1	0.1	0	4
1030	6	0.6	0	2
1031	10	1	0	2
1032	1	0.1	0	4
1033	7	0.7	0	2
1034	3	0.3	0	2
1035	5	0.5	0	2
1036	5	0.5	0	2
1037	2	0.2	0	2

1038	5	0.5	0	2		
1039	5	0.5	0	2		
1041	2	0.2	0	2		
1042	3	0.3	0	2		
1043	3	0.3	0	2		
1044	4	0.4	0	2		
1045	4	0.4	0	2		
1046	3	0.3	0	2		
1047	1	0.1	0	4		
1048	1	0.1	0	4		
1049	5	0.5	0	2		
1050	5	0.5	0	2		
1051	3	0.3	0	2		
1052	4	0.4	0	2		
1053	1	0.1	0	4		
1054	1	0.1	0	4		
1057	1	0.1	0	4		
1059	1	0.1	0	4		
1060	2	0.2	0	2		
1061	2	0.2	0	2		
1062	2	0.2	0	2		
1063	4	0.4	0	2		
1064	5	0.5	0	2		
1065	4	0.4	0	2		
1066	1	0.1	0	4		
1067	1	0.1	0	4		
1068	2	0.2	0	2		
1069	3	0.3	0	2		
1070	5	0.575	0	2		
1071	2	0.25	0	2		
1072	9	1.125	0	2		
1073	11	1.375	0	2		
1074	4	0.5	0	2		
1075	3	0.375	0	2		
1076	2	0.25	0	2		
1077	1	0.1	0	4		
1078	3	0.35	0	2		
1080	1	0.25	0	4		
1082	1	0.125	0	4		
1083	2	0.25	0	2		
1085	2	0.25	0	2		
1086	1	0.125	0	4		
1089	1	0.125	0	4		
1091	1	0.125	0	4		
1098	2	0.311111111111111			0	2
1099	1	0.2	0	4		
1100	1	0.2	0	4		
1109	1	0.1	0	4		
1116	2	0.4	0	2		
1121	2	0.4	0	2		
1122	1	0.1	0	4		
1123	3	0.5	0	2		
1124	2	0.2	0	2		
1126	2	0.2	0	2		
1127	2	0.3	0	2		
1129	2	0.3	0	2		
1130	4	0.4	0	2		
1131	1	0.1	0	4		

1134	1	0.1	0	4
1135	1	0.1	0	4
1142	1	0.1	0	4
1144	1	0.1	0	4
1145	2	0.2	0	2
1147	2	0.2	0	2
1148	1	0.1	0	4
1150	1	0.1	0	4
1151	1	0.1	0	4
1152	1	0.1	0	4
1153	1	0.1	0	4
1154	1	0.1	0	4
1157	1	0.1	0	4
1159	1	0.1	0	4
1160	2	0.2	0	2
1161	1	0.1	0	4
1162	1	0.1	0	4
1163	1	0.1	0	4
1164	1	0.1	0	4
1165	1	0.1	0	4
1167	2	0.2	0	2
1168	2	0.2	0	2
1169	1	0.1	0	4
1172	2	0.2	0	2
1179	1	0.111111111111111	0	4
1180	2	0.222222222222222	0	2
1181	1	0.111111111111111	0	4
1182	1	0.111111111111111	0	4
1185	1	0.111111111111111	0	4
1188	1	0.111111111111111	0	4
1191	1	0.111111111111111	0	4
1197	1	0.2	0	4
1202	1	0.2	0	4
1205	1	0.2	0	4
1207	2	0.4	0	2
1211	2	0.4	0	2
1212	2	0.4	0	2
1213	1	0.2	0	4
1214	1	0.2	0	4
1216	1	0.2	0	4
1217	1	0.2	0	4
1219	2	0.4	0	2
1220	3	0.6	0	2
1221	1	0.2	0	4
1222	1	0.2	0	4
1223	2	0.4	0	2
1226	1	0.1	0	4
1227	1	0.2	0	4
1228	5	1	0	2
1232	1	0.2	0	4
1233	2	0.4	0	2
1234	1	0.1	0	4
1236	5	0.5	0	2
1239	2	0.2	0	2
1240	2	0.2	0	2
1241	3	0.3	0	2
1243	1	0.1	0	4
1246	12	1.2	0	2

1247	1	0.1	0	4
1248	6	0.6	0	2
1250	1	0.1	0	4
1251	1	0.1	0	4
1252	2	0.2	0	2
1257	1	0.1	0	4
1259	1	0.1	0	4
1260	1	0.1	0	4
1261	1	0.1	0	4
1262	1	0.1	0	4
1266	1	0.1	0	4
1267	1	0.1	0	4
1270	1	0.1	0	4
1271	7	0.7	0	2
1272	1	0.1	0	4
1275	1	0.1	0	4
1276	1	0.1	0	4
1278	2	0.2	0	2
1281	1	0.1	0	4
1283	3	0.3	0	2
1284	2	0.2	0	2
1286	1	0.1	0	4
1287	1	0.1	0	4
1288	6	0.6	0	2
1289	1	0.1	0	4
1291	2	0.2	0	2
1294	1	0.2	0	4
1296	1	0.2	0	4
1303	1	0.2	0	4
1312	1	0.2	0	4
1314	1	0.1	0	4
1316	1	0.1	0	4
1318	2	0.4	0	2
1321	1	0.1	0	4
1323	1	0.1	0	4
1324	1	0.1	0	4
1325	2	0.2	0	2
1326	1	0.1	0	4
1327	1	0.2	0	4
1329	1	0.1	0	4
1330	1	0.1	0	4
1333	1	0.1	0	4
1336	4	0.4	0	2
1340	1	0.2	0	4
1342	1	0.1	0	4
1343	1	0.2	0	4
1345	1	0.2	0	4
1346	1	0.2	0	4
1347	1	0.2	0	4
1348	3	0.6	0	2
1349	2	0.4	0	2
1350	1	0.2	0	4
1351	1	0.2	0	4
1353	1	0.2	0	4
1354	1	0.2	0	4
1355	2	0.3	0	2
1356	2	0.4	0	2
1364	4	0.4	0	2

1365	2	0.2	0	2
1366	1	0.1	0	4
1367	3	0.6	0	2
1374	2	0.4	0	2
1454	1	0.2	0	4
1460	1	0.2	0	4
1463	1	0.2	0	4
1477	1	0.2	0	4
1478	1	0.2	0	4
1480	1	0.2	0	4
1481	1	0.2	0	4
1484	1	0.2	0	4
1488	1	0.2	0	4
1492	2	0.4	0	2
1493	1	0.2	0	4
1495	1	0.2	0	4
1504	1	0.2	0	4
1508	3	0.6	0	2
1509	3	0.6	0	2
1510	3	0.6	0	2
1511	7	1.4	0	0
1512	1	0.2	0	4
1516	1	0.2	0	4
1517	1	0.2	0	4
1520	1	0.2	0	4
1533	1	0.2	0	4
1559	1	0.2	0	4

category=0, cleavage_site=1112
query=ptc-miR164a-e, target=Potri.012G001400.1,
score=2.5, range=1101-1121, strand=1

```
target  5' CCUACGUGCCCuGCUUCUCCA 3'
              .::::::::::::::::::
query   3' ACGUGCACGGGACGAAGAGGU 5'
```

>Potri.012G001400.1
#size=1934

419	2	0.666666666666667	0	2	
469	1	0.333333333333333	0	4	
476	1	0.333333333333333	0	4	
507	1	0.333333333333333	0	4	
520	1	0.333333333333333	0	4	
547	1	0.25	0	4	
591	1	0.333333333333333	0	4	
657	1	0.25	0	4	
672	1	0.25	0	4	
724	1	0.25	0	4	
845	1	0.166666666666667	0	4	
847	1	0.166666666666667	0	4	
971	1	0.333333333333333	0	4	
1112	21	7	0	0	<<<
1113	1	0.333333333333333	0	4	
1130	1	0.333333333333333	0	4	
1151	1	0.333333333333333	0	4	
1154	1	0.333333333333333	0	4	
1162	1	0.333333333333333	0	4	
1182	1	0.5	0	4	
1185	1	0.5	0	4	
1196	1	0.5	0	4	

1251	1	0.5	0	4
1327	1	0.333333333333333	0	4
1387	1	0.333333333333333	0	4
1391	1	0.333333333333333	0	4
1396	1	0.333333333333333	0	4
1400	1	0.333333333333333	0	4
1432	2	0.5	0	2
1477	1	0.333333333333333	0	4
1486	1	0.333333333333333	0	4
1489	1	0.333333333333333	0	4
1502	2	0.666666666666667	0	2
1512	1	0.333333333333333	0	4
1518	1	0.333333333333333	0	4
1524	1	0.333333333333333	0	4
1559	1	0.25	0	4
1575	1	0.333333333333333	0	4
1634	2	0.666666666666667	0	2
1635	1	0.333333333333333	0	4
1636	2	0.666666666666667	0	2
1637	1	0.333333333333333	0	4
1638	2	0.666666666666667	0	2
1698	1	0.333333333333333	0	4
1757	1	0.333333333333333	0	4
1759	1	0.333333333333333	0	4

---

category=0, cleavage_site=1057
query=ptc-miR164a-e, target=Potri.015G020000.1,
score=2.5, range=1046-1066, strand=1

```
target  5' CCUACGUGCCCuGCUUCUCCA 3'
              .::::::::::::::::::
query   3' ACGUGCACGGGACGAAGAGGU 5'
```

>Potri.015G020000.1
#size=1756

383	1	1	1	4	
425	1	1	1	4	
492	1	0.25	0	4	
540	1	1	1	4	
602	1	0.25	0	4	
617	1	0.25	0	4	
669	1	0.25	0	4	
1057	4	4	4	0	<<<
1130	1	1	1	4	
1136	1	1	1	4	
1242	1	1	1	4	
1335	1	1	1	4	
1377	2	0.5	0	3	
1465	1	1	1	4	
1504	1	0.25	0	4	
1583	3	3	3	2	

---

category=0, cleavage_site=878
query=ptc-miR164a-e, target=Potri.017G086200.1,
score=4, range=867-887, strand=1

```
target  5' CUCACGUGACCuGCUUCUCCA 3'
              :::::: :::::::::::
query   3' ACGUGCACGGGACGAAGAGGU 5'
```

>Potri.017G086200.1
#size=1520

721	1	0.5	0	4	
878	2	2	2	0	<<<
880	1	1	1	4	
1074	1	0.5	0	4	
1163	1	0.5	0	4	
1214	1	0.5	0	4	

ptc-miR166a-m

category=2, cleavage_site=1717
query=ptc-miR166a-m, target=Potri.001G188800.1,
score=2.5, range=1706-1726, strand=1

```
target  5' CUGGAAUGAAGcCUGGUCCGG 3'
              :::::::::::::::::::.
query   3' CCCCUUACUUCGGACCAGGCU 5'
```

>Potri.001G188800.1
#size=4154

1170	1	0.333333333333333	0	4	
1329	1	0.2	0	4	
1410	1	0.2	0	4	
1679	1	0.1	0	4	
1700	1	0.1	0	4	
1717	5	0.5	0	2	<<<
1718	1	0.1	0	4	
1730	1	0.1	0	4	
1766	1	0.333333333333333	0	4	
1784	1	0.333333333333333	0	4	
1799	1	0.166666666666667	0	4	
1823	1	0.1	0	4	
1900	1	0.166666666666667	0	4	
1981	1	0.142857142857143	0	4	
1998	1	0.142857142857143	0	4	
2023	1	0.142857142857143	0	4	
2026	1	0.142857142857143	0	4	
2037	1	0.142857142857143	0	4	
2043	1	0.142857142857143	0	4	
2083	1	0.142857142857143	0	4	
2095	1	0.142857142857143	0	4	
2098	1	0.142857142857143	0	4	
2113	1	0.142857142857143	0	4	
2133	1	0.142857142857143	0	4	
2139	2	0.285714285714286	0	2	
2155	1	0.142857142857143	0	4	
2169	1	0.25	0	4	
2222	1	0.25	0	4	
2289	1	0.142857142857143	0	4	
2308	1	0.25	0	4	
2319	1	0.25	0	4	
2373	2	0.285714285714286	0	2	
2412	1	0.142857142857143	0	4	
2420	1	0.142857142857143	0	4	
2421	1	0.142857142857143	0	4	
2434	1	0.142857142857143	0	4	
2435	1	0.142857142857143	0	4	
2484	1	0.142857142857143	0	4	
2496	1	0.25	0	4	
2502	1	0.25	0	4	
2585	1	0.142857142857143	0	4	

2588	1	0.142857142857143	0	4
2630	1	0.25	0	4
2637	1	0.25	0	4
2640	1	0.25	0	4
2645	1	0.25	0	4
2647	1	0.142857142857143	0	4
2650	1	0.142857142857143	0	4
2660	1	0.25	0	4
2678	1	0.25	0	4
2691	1	0.25	0	4
2707	2	0.5	0	2
2720	1	0.25	0	4
2819	1	0.142857142857143	0	4
2824	1	0.142857142857143	0	4
2838	1	0.142857142857143	0	4
2848	1	0.142857142857143	0	4
2851	1	0.142857142857143	0	4
2874	1	0.142857142857143	0	4
2896	2	0.285714285714286	0	2
2898	2	0.285714285714286	0	2
2902	1	0.25	0	4
2924	1	0.25	0	4
2935	1	0.142857142857143	0	4
2952	1	0.142857142857143	0	4
2953	1	0.142857142857143	0	4
2954	1	0.142857142857143	0	4
2955	1	0.142857142857143	0	4
2965	1	0.142857142857143	0	4
3021	1	0.25	0	4
3122	2	0.333333333333333	0	2
3129	2	0.333333333333333	0	2
3137	1	0.333333333333333	0	4
3151	1	0.166666666666667	0	4
3153	1	0.166666666666667	0	4
3165	1	0.166666666666667	0	4
3187	1	0.333333333333333	0	4
3245	1	0.142857142857143	0	4
3253	1	0.142857142857143	0	4
3258	1	0.142857142857143	0	4
3267	1	0.142857142857143	0	4
3275	2	0.333333333333333	0	2
3276	2	0.333333333333333	0	2
3277	2	0.333333333333333	0	2
3278	1	0.166666666666667	0	4
3279	6	1	0	2
3283	1	0.166666666666667	0	4
3286	1	0.166666666666667	0	4
3289	1	0.166666666666667	0	4
3293	1	0.25	0	4
3294	1	0.166666666666667	0	4
3324	1	0.25	0	4
3331	1	0.25	0	4
3341	2	0.285714285714286	0	2
3353	1	0.142857142857143	0	4
3354	1	0.142857142857143	0	4
3356	1	0.142857142857143	0	4
3358	1	0.142857142857143	0	4
3361	1	0.142857142857143	0	4

3363	1	0.142857142857143	0	4
3365	1	0.142857142857143	0	4
3366	1	0.142857142857143	0	4
3367	1	0.25	0	4
3368	1	0.142857142857143	0	4
3379	1	0.25	0	4
3382	1	0.25	0	4
3383	3	0.75	0	2
3384	1	0.25	0	4
3385	1	0.25	0	4
3399	2	0.5	0	2
3416	2	0.5	0	2
3453	1	0.142857142857143	0	4
3455	1	0.142857142857143	0	4
3461	2	0.285714285714286	0	2
3472	2	0.285714285714286	0	2
3478	1	0.1	0	4
3490	1	0.1	0	4
3491	1	0.1	0	4
3492	1	0.1	0	4
3494	1	0.142857142857143	0	4
3496	1	0.142857142857143	0	4
3497	1	0.142857142857143	0	4
3498	2	0.285714285714286	0	2
3499	1	0.142857142857143	0	4
3500	1	0.142857142857143	0	4
3501	1	0.142857142857143	0	4
3502	1	0.142857142857143	0	4
3504	2	0.285714285714286	0	2
3518	1	0.142857142857143	0	4
3531	1	0.142857142857143	0	4
3532	1	0.142857142857143	0	4
3533	1	0.142857142857143	0	4
3534	1	0.142857142857143	0	4
3535	1	0.142857142857143	0	4
3559	1	0.142857142857143	0	4
3560	1	0.142857142857143	0	4
3583	1	0.142857142857143	0	4
3588	2	0.285714285714286	0	2
3600	1	0.25	0	4
3609	1	0.25	0	4
3611	1	0.25	0	4
3614	1	0.25	0	4
3624	1	0.25	0	4
3627	2	0.285714285714286	0	2
3628	1	0.142857142857143	0	4
3630	1	0.142857142857143	0	4
3631	1	0.142857142857143	0	4
3634	2	0.285714285714286	0	2
3636	1	0.142857142857143	0	4
3640	1	0.142857142857143	0	4
3641	1	0.142857142857143	0	4
3643	1	0.142857142857143	0	4
3644	2	0.285714285714286	0	2
3645	1	0.142857142857143	0	4
3646	1	0.142857142857143	0	4
3648	1	0.142857142857143	0	4
3652	1	0.142857142857143	0	4

3656	1	0.142857142857143	0	4
3658	1	0.142857142857143	0	4
3676	1	0.142857142857143	0	4
3679	2	0.285714285714286	0	2
3683	3	0.535714285714286	0	2
3706	8	2	0	0
3707	4	1	0	2
3708	1	0.25	0	4
3710	2	0.5	0	2
3711	1	0.25	0	4
3712	6	1.5	0	2
3713	6	1.5	0	2
3714	4	1	0	2
3715	1	0.25	0	4
3741	1	0.25	0	4
3744	1	0.25	0	4
3746	3	0.75	0	2
3748	2	0.5	0	2
3749	1	0.25	0	4
3750	1	0.25	0	4
3752	1	0.25	0	4
3753	1	0.25	0	4
3756	1	0.25	0	4
3773	1	0.25	0	4
3777	1	0.25	0	4
3789	6	1.5	0	2
3790	1	0.25	0	4
3798	1	0.25	0	4
3808	2	0.5	0	2
3823	2	0.5	0	2
3825	1	0.25	0	4
3827	1	0.25	0	4
3833	2	0.5	0	2
3834	1	0.25	0	4
3841	1	0.25	0	4
3844	1	0.25	0	4
3850	2	0.5	0	2
3858	2	0.5	0	2
3861	1	0.25	0	4
3866	2	0.5	0	2
3867	5	1.25	0	2
3868	1	0.25	0	4
3875	1	0.25	0	4
3877	1	0.25	0	4
3878	1	0.25	0	4
3880	1	0.25	0	4
3885	1	0.25	0	4
3887	5	1.25	0	2
3888	3	0.75	0	2
3889	1	0.25	0	4
3890	3	0.75	0	2
3892	3	0.75	0	2
3905	1	0.25	0	4
3975	1	0.25	0	4
4009	1	0.25	0	4

category=0, cleavage_site=1139
query=ptc-miR166a-m, target=Potri.001G372300.1,
score=3.5, range=1128-1148, strand=1

```
target  5' UUGGUAUGAAGcCUGGUCCGG 3'
             :: :::::::::::::::.
query   3' CCCCUUACUUCGGACCAGGCU 5'
```

>Potri.001G372300.1
#size=3340

154	2	2	2	2	
170	1	1	1	4	
175	1	1	1	4	
176	1	1	1	4	
185	4	4	4	2	
189	1	1	1	4	
193	1	1	1	4	
202	1	1	1	4	
280	2	2	2	2	
290	1	1	1	4	
298	1	1	1	4	
307	1	1	1	4	
326	1	0.5	0	4	
335	1	0.5	0	4	
337	2	1	0	2	
338	1	0.5	0	4	
346	1	0.5	0	4	
428	1	1	1	4	
488	1	1	1	4	
561	1	0.5	0	4	
578	1	0.5	0	4	
609	1	0.5	0	4	
733	1	0.142857142857143	0	4	
736	1	0.142857142857143	0	4	
737	1	0.5	0	4	
742	1	0.5	0	4	
747	3	1.5	0	2	
766	1	0.333333333333333	0	4	
772	1	0.333333333333333	0	4	
817	1	0.333333333333333	0	4	
1135	1	0.333333333333333	0	4	
1139	24	8	0	0	<<<
1141	1	0.333333333333333	0	4	
1143	1	0.333333333333333	0	4	
1153	1	0.333333333333333	0	4	
1157	1	0.333333333333333	0	4	
1171	2	0.833333333333333	0	2	
1176	1	0.5	0	4	
1182	1	0.5	0	4	
1185	1	0.333333333333333	0	4	
1198	1	0.5	0	4	
1203	1	0.5	0	4	
1206	2	1	0	2	
1207	1	0.5	0	4	
1209	1	0.333333333333333	0	4	
1212	1	0.333333333333333	0	4	
1220	1	0.333333333333333	0	4	
1222	1	0.333333333333333	0	4	
1225	1	0.333333333333333	0	4	
1229	1	0.5	0	4	

1230	1	0.5	0	4
1234	1	0.333333333333333	0	4
1235	2	0.666666666666667	0	2
1240	1	0.333333333333333	0	4
1243	4	1.33333333333333	0	2
1244	2	0.666666666666667	0	2
1248	2	1	0	2
1252	1	0.333333333333333	0	4
1253	1	0.5	0	4
1256	2	1	0	2
1258	1	0.5	0	4
1267	1	0.5	0	4
1278	1	0.5	0	4
1284	1	0.5	0	4
1294	1	0.333333333333333	0	4
1298	1	0.333333333333333	0	4
1308	1	0.333333333333333	0	4
1317	1	0.333333333333333	0	4
1362	1	0.333333333333333	0	4
1408	1	0.5	0	4
1498	1	0.333333333333333	0	4
1516	1	0.5	0	4
1557	2	0.666666666666667	0	2
1559	1	0.333333333333333	0	4
1575	1	0.333333333333333	0	4
1602	1	0.333333333333333	0	4
1629	1	0.333333333333333	0	4
1631	1	0.333333333333333	0	4
1634	1	0.333333333333333	0	4
1640	1	0.333333333333333	0	4
1741	1	0.333333333333333	0	4
1757	1	0.333333333333333	0	4
1875	1	0.5	0	4
1938	1	0.333333333333333	0	4
1957	1	0.333333333333333	0	4
1968	1	0.333333333333333	0	4
1969	1	0.333333333333333	0	4
1970	1	0.333333333333333	0	4
1976	1	0.333333333333333	0	4
1989	1	0.5	0	4
2004	1	0.5	0	4
2016	1	0.5	0	4
2030	1	0.333333333333333	0	4
2083	1	0.333333333333333	0	4
2117	1	0.333333333333333	0	4
2129	1	0.333333333333333	0	4
2134	1	0.333333333333333	0	4
2135	1	0.333333333333333	0	4
2141	1	0.333333333333333	0	4
2165	1	0.333333333333333	0	4
2173	1	0.5	0	4
2181	1	0.5	0	4
2188	1	0.5	0	4
2197	2	1	0	2
2200	1	0.5	0	4
2202	1	0.5	0	4
2204	1	0.5	0	4
2213	1	0.333333333333333	0	4

2223	1	0.333333333333333	0	4
2232	1	0.333333333333333	0	4
2247	1	0.333333333333333	0	4
2277	1	0.333333333333333	0	4
2295	1	0.333333333333333	0	4
2326	1	0.5	0	4
2328	1	0.333333333333333	0	4
2336	1	0.333333333333333	0	4
2337	1	0.333333333333333	0	4
2338	1	0.333333333333333	0	4
2433	1	0.333333333333333	0	4
2462	1	0.333333333333333	0	4
2466	1	0.5	0	4
2562	1	0.333333333333333	0	4
2578	1	0.5	0	4
2587	2	1	0	2
2608	2	1	0	2
2619	1	0.5	0	4
2639	1	0.5	0	4
2640	1	0.5	0	4
2645	1	0.5	0	4
2646	2	1	0	2
2647	2	1	0	2
2648	1	0.5	0	4
2650	1	0.5	0	4
2663	1	0.5	0	4
2671	1	0.333333333333333	0	4
2677	1	0.333333333333333	0	4
2678	1	0.333333333333333	0	4
2692	1	0.333333333333333	0	4
2694	1	0.333333333333333	0	4
2696	1	0.333333333333333	0	4
2700	1	0.333333333333333	0	4
2701	2	0.666666666666667	0	2
2705	2	0.666666666666667	0	2
2706	2	0.666666666666667	0	2
2710	2	0.666666666666667	0	2
2711	1	0.333333333333333	0	4
2714	2	0.666666666666667	0	2
2719	1	0.333333333333333	0	4
2720	1	0.5	0	4
2728	1	0.5	0	4
2738	1	0.333333333333333	0	4
2741	1	0.333333333333333	0	4
2752	1	0.333333333333333	0	4
2753	1	0.333333333333333	0	4
2754	1	0.333333333333333	0	4
2770	1	0.333333333333333	0	4
2776	2	0.666666666666667	0	2
2783	1	0.333333333333333	0	4
2830	1	0.333333333333333	0	4
2842	1	0.333333333333333	0	4
2847	2	0.666666666666667	0	2
2863	3	1	0	2
2865	1	0.333333333333333	0	4
2894	1	0.333333333333333	0	4
2903	1	0.333333333333333	0	4
2905	1	0.333333333333333	0	4

2906	1	0.333333333333333	0	4
2914	2	0.666666666666667	0	2
2923	1	0.5	0	4
2925	2	1	0	2
2927	2	1	0	2
2929	1	0.5	0	4
2932	3	1.5	0	2
2938	1	0.5	0	4
2954	1	0.333333333333333	0	4
2959	1	0.333333333333333	0	4
2966	1	0.333333333333333	0	4
2967	1	0.5	0	4
2969	1	0.333333333333333	0	4
2990	1	0.5	0	4
2993	2	1	0	2
2994	2	1	0	2
2995	2	1	0	2
2996	1	0.5	0	4
3003	1	0.5	0	4
3005	1	0.5	0	4
3006	1	0.5	0	4
3007	1	0.5	0	4
3008	1	0.5	0	4
3012	1	0.5	0	4
3015	2	1	0	2
3020	1	0.5	0	4
3022	1	0.5	0	4
3025	1	0.333333333333333	0	4
3027	1	0.333333333333333	0	4
3029	1	0.5	0	4
3044	1	0.5	0	4
3061	1	0.5	0	4
3081	1	0.5	0	4
3085	2	1	0	2
3088	1	0.5	0	4
3118	1	0.5	0	4
3146	1	0.5	0	4
3153	1	0.5	0	4
3186	1	0.5	0	4
3191	1	0.5	0	4
3211	1	0.5	0	4
3220	1	0.5	0	4

category=2, cleavage_site=1538
query=ptc-miR166a-m, target=Potri.003G050100.1,
score=2.5, range=1527-1547, strand=1

```
target  5' CUGGAAUGAAGcCUGGUCCGG 3'
              :::::::::::::::::::.
query   3' CCCCUUACUUCGGACCAGGCU 5'
```

>Potri.003G050100.1
#size=3807

481	1	1	1	4
692	1	1	1	4
834	1	1	1	4
1150	1	0.2	0	4
1154	1	0.25	0	4
1165	1	0.5	0	4
1231	1	0.2	0	4

1500	1	0.1	0	4	
1521	1	0.1	0	4	
1538	4	0.4	0	2	<<<
1539	1	0.1	0	4	
1585	1	0.333333333333333	0	4	
1620	1	0.166666666666667	0	4	
1644	1	0.1	0	4	
1647	1	0.111111111111111	0	4	
1704	1	0.333333333333333	0	4	
1721	1	0.166666666666667	0	4	
1750	1	0.333333333333333	0	4	
1802	1	0.142857142857143	0	4	
1819	1	0.142857142857143	0	4	
1844	1	0.142857142857143	0	4	
1847	1	0.142857142857143	0	4	
1858	1	0.142857142857143	0	4	
1864	1	0.142857142857143	0	4	
1904	1	0.142857142857143	0	4	
1916	1	0.142857142857143	0	4	
1919	1	0.142857142857143	0	4	
1934	1	0.142857142857143	0	4	
1954	1	0.142857142857143	0	4	
1960	2	0.285714285714286	0	3	
1976	1	0.142857142857143	0	4	
2012	1	0.333333333333333	0	4	
2110	1	0.142857142857143	0	4	
2194	2	0.285714285714286	0	3	
2201	1	0.333333333333333	0	4	
2213	1	0.333333333333333	0	4	
2233	1	0.142857142857143	0	4	
2241	1	0.142857142857143	0	4	
2242	1	0.142857142857143	0	4	
2255	1	0.142857142857143	0	4	
2256	1	0.142857142857143	0	4	
2305	1	0.142857142857143	0	4	
2326	2	0.666666666666667	0	2	
2327	1	0.333333333333333	0	4	
2333	3	0.5	0	2	
2334	1	0.166666666666667	0	4	
2335	1	0.166666666666667	0	4	
2343	1	0.166666666666667	0	4	
2353	1	0.166666666666667	0	4	
2355	2	0.333333333333333	0	2	
2367	1	0.142857142857143	0	4	
2370	1	0.142857142857143	0	4	
2382	1	0.333333333333333	0	4	
2429	1	0.142857142857143	0	4	
2432	1	0.142857142857143	0	4	
2441	1	0.333333333333333	0	4	
2466	1	0.333333333333333	0	4	
2493	1	0.333333333333333	0	4	
2520	1	0.333333333333333	0	4	
2532	4	1.33333333333333	0	2	
2533	1	0.333333333333333	0	4	
2541	3	1	0	2	
2601	1	0.142857142857143	0	4	
2606	1	0.142857142857143	0	4	
2620	1	0.142857142857143	0	4	

2630	1	0.142857142857143	0	4
2633	1	0.142857142857143	0	4
2656	1	0.142857142857143	0	4
2678	2	0.285714285714286	0	3
2680	2	0.285714285714286	0	3
2717	1	0.142857142857143	0	4
2734	1	0.142857142857143	0	4
2735	1	0.142857142857143	0	4
2736	1	0.142857142857143	0	4
2737	1	0.142857142857143	0	4
2747	1	0.142857142857143	0	4
2804	1	0.333333333333333	0	4
2815	1	0.333333333333333	0	4
2907	2	0.333333333333333	0	2
2914	2	0.333333333333333	0	2
2936	1	0.166666666666667	0	4
2938	1	0.166666666666667	0	4
2950	1	0.166666666666667	0	4
3027	1	0.142857142857143	0	4
3035	1	0.142857142857143	0	4
3040	1	0.142857142857143	0	4
3049	1	0.142857142857143	0	4
3057	2	0.333333333333333	0	2
3058	2	0.333333333333333	0	2
3059	2	0.333333333333333	0	2
3060	1	0.166666666666667	0	4
3061	6	1	0	2
3065	1	0.166666666666667	0	4
3068	1	0.166666666666667	0	4
3071	1	0.166666666666667	0	4
3076	1	0.166666666666667	0	4
3086	1	0.5	0	4
3097	1	0.333333333333333	0	4
3098	2	0.666666666666667	0	2
3105	1	0.333333333333333	0	4
3106	1	0.333333333333333	0	4
3107	1	0.333333333333333	0	4
3109	1	0.333333333333333	0	4
3110	2	0.666666666666667	0	2
3119	1	0.333333333333333	0	4
3123	2	0.285714285714286	0	3
3135	1	0.142857142857143	0	4
3136	1	0.142857142857143	0	4
3138	1	0.142857142857143	0	4
3140	2	0.476190476190476	0	2
3143	1	0.142857142857143	0	4
3145	1	0.142857142857143	0	4
3147	1	0.142857142857143	0	4
3148	1	0.142857142857143	0	4
3150	1	0.142857142857143	0	4
3157	1	0.333333333333333	0	4
3160	1	0.333333333333333	0	4
3162	1	0.333333333333333	0	4
3163	2	0.666666666666667	0	2
3164	1	0.333333333333333	0	4
3165	7	2.33333333333333	0	0
3193	1	0.333333333333333	0	4
3220	1	0.333333333333333	0	4

3223	2	0.666666666666667	0	2
3230	1	0.333333333333333	0	4
3234	1	0.333333333333333	0	4
3235	1	0.142857142857143	0	4
3237	1	0.142857142857143	0	4
3238	1	0.333333333333333	0	4
3243	2	0.285714285714286	0	3
3244	1	0.333333333333333	0	4
3254	2	0.285714285714286	0	3
3260	1	0.1 0 4		
3274	1	0.1 0 4		
3276	1	0.142857142857143	0	4
3278	1	0.142857142857143	0	4
3279	1	0.142857142857143	0	4
3280	2	0.285714285714286	0	3
3281	1	0.142857142857143	0	4
3282	1	0.142857142857143	0	4
3283	1	0.142857142857143	0	4
3284	1	0.142857142857143	0	4
3286	2	0.285714285714286	0	3
3297	1	0.333333333333333	0	4
3300	1	0.142857142857143	0	4
3313	1	0.142857142857143	0	4
3314	1	0.142857142857143	0	4
3315	1	0.142857142857143	0	4
3316	1	0.142857142857143	0	4
3317	1	0.142857142857143	0	4
3341	1	0.142857142857143	0	4
3342	1	0.142857142857143	0	4
3365	1	0.142857142857143	0	4
3370	2	0.285714285714286	0	3
3372	3	1 0 2		
3373	2	0.666666666666667	0	2
3384	1	0.333333333333333	0	4
3385	5	1.66666666666667	0	2
3386	1	0.333333333333333	0	4
3387	4	1.33333333333333	0	2
3398	1	0.333333333333333	0	4
3399	1	0.333333333333333	0	4
3400	1	0.333333333333333	0	4
3401	1	0.333333333333333	0	4
3402	4	1.33333333333333	0	2
3404	1	0.333333333333333	0	4
3406	1	0.333333333333333	0	4
3409	2	0.285714285714286	0	3
3410	1	0.142857142857143	0	4
3411	1	0.333333333333333	0	4
3412	1	0.142857142857143	0	4
3413	1	0.142857142857143	0	4
3416	2	0.285714285714286	0	3
3418	1	0.142857142857143	0	4
3422	1	0.142857142857143	0	4
3423	1	0.142857142857143	0	4
3425	1	0.142857142857143	0	4
3426	2	0.285714285714286	0	3
3427	1	0.142857142857143	0	4
3428	1	0.142857142857143	0	4
3430	1	0.142857142857143	0	4

3434	1	0.142857142857143	0	4
3438	1	0.142857142857143	0	4
3440	1	0.142857142857143	0	4
3458	1	0.142857142857143	0	4
3461	2	0.285714285714286	0	3
3465	3	0.619047619047619	0	2
3466	1	0.333333333333333	0	4
3467	1	0.333333333333333	0	4
3468	1	0.333333333333333	0	4
3470	2	0.666666666666667	0	2
3471	2	0.666666666666667	0	2
3474	1	0.333333333333333	0	4
3476	1	0.333333333333333	0	4
3477	1	0.333333333333333	0	4
3483	1	0.333333333333333	0	4
3484	1	0.333333333333333	0	4
3485	2	0.666666666666667	0	2
3486	1	0.333333333333333	0	4
3487	1	0.333333333333333	0	4
3488	4	1.33333333333333	0	2
3489	1	0.333333333333333	0	4
3491	1	0.333333333333333	0	4
3492	1	0.333333333333333	0	4
3495	4	1.33333333333333	0	2
3496	1	0.333333333333333	0	4
3497	1	0.333333333333333	0	4
3498	1	0.333333333333333	0	4
3499	1	0.333333333333333	0	4
3500	1	0.333333333333333	0	4
3501	1	0.333333333333333	0	4
3502	2	0.666666666666667	0	2
3515	1	0.333333333333333	0	4
3517	2	0.666666666666667	0	2
3518	1	0.333333333333333	0	4
3519	2	0.666666666666667	0	2
3520	1	0.333333333333333	0	4
3521	1	0.333333333333333	0	4
3523	1	0.333333333333333	0	4
3526	1	0.333333333333333	0	4
3532	1	0.333333333333333	0	4
3538	1	0.333333333333333	0	4
3539	1	0.333333333333333	0	4
3548	2	0.666666666666667	0	2
3555	1	0.333333333333333	0	4
3559	1	0.333333333333333	0	4
3568	1	0.333333333333333	0	4
3569	1	0.333333333333333	0	4
3572	1	0.333333333333333	0	4
3578	1	0.333333333333333	0	4
3581	1	0.333333333333333	0	4
3585	1	0.333333333333333	0	4
3586	1	0.333333333333333	0	4
3587	1	0.333333333333333	0	4
3588	1	0.333333333333333	0	4
3589	1	0.333333333333333	0	4
3590	1	0.333333333333333	0	4
3596	5	1.66666666666667	0	2
3599	1	0.333333333333333	0	4

3609	1	0.333333333333333	0	4
3647	1	0.333333333333333	0	4
3651	1	0.333333333333333	0	4
3694	1	0.333333333333333	0	4

category=4, cleavage_site=1310
query=ptc-miR166a-m, target=Potri.004G211300.1,
score=2.5, range=1299-1319, strand=1

```
target  5' CUGGAAUGAAGcCUGGUCCGG 3'
              ::::::::::::::::::.
query   3' CCCCUUACUUCGGACCAGGCU 5'
```

>Potri.004G211300.1
#size=3307

603	1	1	1	4	
616	1	1	1	4	
631	1	1	1	4	
928	1	0.2	0	4	
943	1	0.142857142857143	0	4	
946	1	0.142857142857143	0	4	
947	1	0.2	0	4	
1001	1	0.2	0	4	
1310	1	0.333333333333333	0	4	<<<
1466	1	0.142857142857143	0	4	
1650	1	0.142857142857143	0	4	
1755	1	0.142857142857143	0	4	
1864	1	0.142857142857143	0	4	
1911	1	0.142857142857143	0	4	
1932	1	0.142857142857143	0	4	
2074	1	0.333333333333333	0	4	
2158	1	0.142857142857143	0	4	
2169	1	0.142857142857143	0	4	
2170	1	0.142857142857143	0	4	
2174	1	0.142857142857143	0	4	
2301	1	0.142857142857143	0	4	
2310	1	0.142857142857143	0	4	
2360	1	0.142857142857143	0	4	
2402	1	0.142857142857143	0	4	
2515	1	0.333333333333333	0	4	
2551	1	0.333333333333333	0	4	
2639	1	0.142857142857143	0	4	
2723	1	0.142857142857143	0	4	
2743	2	0.285714285714286	0	2	
2765	1	0.142857142857143	0	4	
2805	1	0.333333333333333	0	4	
2809	1	0.333333333333333	0	4	
2876	1	0.142857142857143	0	4	
2907	1	0.142857142857143	0	4	
2908	1	0.142857142857143	0	4	
2940	1	0.142857142857143	0	4	
3013	1	0.142857142857143	0	4	
3021	1	0.142857142857143	0	4	
3022	1	0.142857142857143	0	4	
3103	1	0.333333333333333	0	4	
3106	1	0.2	0	4	
3140	1	0.142857142857143	0	4	
3157	1	0.142857142857143	0	4	

3158	1	0.142857142857143	0	4
3160	1	0.142857142857143	0	4
3169	1	0.142857142857143	0	4
3172	1	0.333333333333333	0	4
3174	1	0.333333333333333	0	4
3176	1	0.333333333333333	0	4
3188	1	0.333333333333333	0	4
3205	1	0.333333333333333	0	4
3239	1	0.142857142857143	0	4
3261	1	0.166666666666667	0	4

---

category=2, cleavage_site=583
query=ptc-miR166a-m, target=Potri.006G237500.1,
score=3, range=572-592, strand=1

```
target  5' CUGGGAUGAAGcCUGGUCCGG 3'
              ::.:::::::::::::::.
query   3' CCCCUUACUUCGGACCAGGCU 5'
```

>Potri.006G237500.1
#size=2787

3	1	0.166666666666667	0	4	
189	1	0.166666666666667	0	4	
196	1	0.25	0	4	
545	1	0.1	0	4	
566	1	0.1	0	4	
583	6	0.6	0	2	<<<
584	1	0.1	0	4	
596	1	0.1	0	4	
692	1	0.111111111111111	0	4	
706	1	0.166666666666667	0	4	
736	1	0.5	0	4	
760	1	0.166666666666667	0	4	
836	1	0.166666666666667	0	4	
864	1	0.166666666666667	0	4	
1024	1	0.5	0	4	
1252	1	0.166666666666667	0	4	
1418	1	0.166666666666667	0	4	
1442	1	0.166666666666667	0	4	
1479	1	0.5	0	4	
1487	1	0.5	0	4	
1557	2	0.333333333333333	0	2	
1632	1	0.166666666666667	0	4	
1663	2	1	0	1	
1700	2	1	0	1	
1820	1	0.5	0	4	
1912	1	0.5	0	4	
1913	1	0.5	0	4	
1917	2	1	0	1	
1947	1	0.166666666666667	0	4	
2000	1	0.166666666666667	0	4	
2016	1	0.5	0	4	
2031	1	0.166666666666667	0	4	
2132	1	0.5	0	4	
2137	1	0.5	0	4	
2144	1	0.5	0	4	
2154	1	0.5	0	4	
2170	1	0.166666666666667	0	4	
2174	1	0.166666666666667	0	4	
2177	1	0.166666666666667	0	4	

2178	1	0.166666666666667	0	4
2193	1	0.5	0	4
2200	1	0.5	0	4
2237	1	0.166666666666667	0	4
2248	1	0.5	0	4
2270	1	0.166666666666667	0	4
2272	1	0.166666666666667	0	4
2284	1	0.166666666666667	0	4
2300	1	0.166666666666667	0	4
2304	1	0.166666666666667	0	4
2305	1	0.166666666666667	0	4
2311	1	0.1	0	4
2323	1	0.1	0	4
2324	1	0.1	0	4
2346	1	0.166666666666667	0	4
2348	2	0.333333333333333	0	2
2352	1	0.166666666666667	0	4
2366	1	0.166666666666667	0	4
2368	1	0.166666666666667	0	4
2380	1	0.166666666666667	0	4
2391	1	0.166666666666667	0	4
2399	1	0.166666666666667	0	4
2403	1	0.166666666666667	0	4
2420	1	0.166666666666667	0	4
2448	1	0.166666666666667	0	4
2449	1	0.5	0	4
2465	1	0.166666666666667	0	4
2468	1	0.5	0	4
2478	1	0.166666666666667	0	4
2481	1	0.166666666666667	0	4
2484	1	0.166666666666667	0	4
2487	1	0.166666666666667	0	4
2496	1	0.166666666666667	0	4
2508	1	0.2	0	4
2510	1	0.2	0	4
2511	1	0.2	0	4
2512	1	0.2	0	4
2522	1	0.5	0	4
2523	2	1	0	1
2533	1	0.5	0	4
2536	1	0.5	0	4
2537	1	0.5	0	4
2545	1	0.5	0	4
2547	1	0.5	0	4
2549	1	0.5	0	4
2553	1	0.5	0	4
2563	2	1	0	1
2583	1	0.5	0	4
2650	1	0.5	0	4

---

category=4, cleavage_site=574
query=ptc-miR166a-m, target=Potri.009G014500.1,
score=3, range=563-583, strand=1

```
target  5' CUGGGAUGAAGcCUGGUCCGG 3'
              ::.:::::::::::::::.
query   3' CCCCUUACUUCGGACCAGGCU 5'
```

>Potri.009G014500.1
#size=2568

70	1	0.25	0	4	
108	1	0.25	0	4	
151	1	0.25	0	4	
169	1	0.25	0	4	
186	1	0.2	0	4	
201	1	0.142857142857143	0	4	
204	1	0.142857142857143	0	4	
205	1	0.2	0	4	
259	1	0.2	0	4	
533	1	0.25	0	4	
574	1	0.25	0	4	<<<
591	1	0.25	0	4	
730	1	0.142857142857143	0	4	
872	1	0.25	0	4	
914	1	0.142857142857143	0	4	
1022	1	0.142857142857143	0	4	
1084	1	0.25	0	4	
1131	1	0.142857142857143	0	4	
1178	1	0.142857142857143	0	4	
1199	1	0.142857142857143	0	4	
1331	1	0.25	0	4	
1337	1	0.25	0	4	
1347	1	0.25	0	4	
1368	2	0.5	0	1	
1425	1	0.142857142857143	0	4	
1436	1	0.142857142857143	0	4	
1437	1	0.142857142857143	0	4	
1441	1	0.142857142857143	0	4	
1458	1	0.25	0	4	
1491	1	0.25	0	4	
1515	1	0.25	0	4	
1527	1	0.25	0	4	
1530	1	0.25	0	4	
1568	1	0.142857142857143	0	4	
1577	1	0.142857142857143	0	4	
1627	1	0.142857142857143	0	4	
1669	1	0.142857142857143	0	4	
1765	1	0.25	0	4	
1815	1	0.25	0	4	
1903	1	0.142857142857143	0	4	
1987	1	0.142857142857143	0	4	
2007	2	0.285714285714286	0	2	
2029	1	0.142857142857143	0	4	
2093	1	0.25	0	4	
2152	1	0.142857142857143	0	4	
2183	1	0.142857142857143	0	4	
2184	1	0.142857142857143	0	4	
2216	1	0.142857142857143	0	4	
2289	1	0.142857142857143	0	4	
2297	1	0.142857142857143	0	4	
2298	1	0.142857142857143	0	4	
2309	1	0.25	0	4	
2322	2	0.5	0	1	
2324	1	0.25	0	4	
2341	2	0.5	0	1	
2367	1	0.25	0	4	
2425	1	0.142857142857143	0	4	
2442	1	0.142857142857143	0	4	

2443	1	0.142857142857143	0	4
2445	1	0.142857142857143	0	4
2452	1	0.25	0	4
2454	1	0.142857142857143	0	4
2456	1	0.25	0	4
2457	1	0.25	0	4
2460	2	0.5	0	1
2491	1	0.25	0	4
2514	2	0.5	0	1
2517	1	0.25	0	4
2518	1	0.25	0	4
2519	2	0.5	0	1
2524	1	0.142857142857143	0	4

---

category=2, cleavage_site=1392
query=ptc-miR166a-m, target=Potri.018G045100.1,
score=3, range=1381-1401, strand=1

```
target  5' CUGGGAUGAAGcCUGGUCCGG 3'
              ::.:::::::::::::::.
query   3' CCCCUUACUUCGGACCAGGCU 5'
```

>Potri.018G045100.1
#size=3670

276	1	0.25	0	4	
812	1	0.166666666666667	0	4	
928	1	0.25	0	4	
998	1	0.166666666666667	0	4	
1354	1	0.1	0	4	
1375	1	0.1	0	4	
1392	6	0.6	0	2	<<<
1393	1	0.1	0	4	
1405	1	0.1	0	4	
1498	1	0.1	0	4	
1501	1	0.111111111111111	0	4	
1515	1	0.166666666666667	0	4	
1569	1	0.166666666666667	0	4	
1645	1	0.166666666666667	0	4	
1673	1	0.166666666666667	0	4	
1816	1	0.25	0	4	
2061	1	0.166666666666667	0	4	
2227	1	0.166666666666667	0	4	
2251	1	0.166666666666667	0	4	
2366	2	0.333333333333333	0	2	
2426	1	0.166666666666667	0	4	
2677	1	0.25	0	4	
2741	1	0.166666666666667	0	4	
2794	1	0.166666666666667	0	4	
2825	1	0.166666666666667	0	4	
2890	1	0.25	0	4	
2902	1	0.25	0	4	
2964	1	0.166666666666667	0	4	
2968	1	0.166666666666667	0	4	
2971	1	0.166666666666667	0	4	
2972	1	0.166666666666667	0	4	
2997	1	0.25	0	4	
2998	1	0.25	0	4	
3031	1	0.166666666666667	0	4	
3036	1	0.25	0	4	
3059	1	0.25	0	4	

3064	1	0.166666666666667	0	4
3066	1	0.166666666666667	0	4
3078	1	0.166666666666667	0	4
3094	1	0.166666666666667	0	4
3098	1	0.166666666666667	0	4
3099	1	0.166666666666667	0	4
3105	1	0.1	0	4
3117	1	0.1	0	4
3118	1	0.1	0	4
3119	1	0.1	0	4
3140	1	0.166666666666667	0	4
3142	2	0.333333333333333	0	2
3146	1	0.166666666666667	0	4
3160	1	0.166666666666667	0	4
3162	1	0.166666666666667	0	4
3174	1	0.166666666666667	0	4
3185	1	0.166666666666667	0	4
3193	1	0.166666666666667	0	4
3197	1	0.166666666666667	0	4
3214	1	0.166666666666667	0	4
3242	1	0.166666666666667	0	4
3247	1	0.25	0	4
3259	1	0.166666666666667	0	4
3270	1	0.25	0	4
3272	1	0.166666666666667	0	4
3275	2	0.416666666666667	0	2
3278	1	0.166666666666667	0	4
3279	1	0.25	0	4
3281	1	0.166666666666667	0	4
3290	1	0.166666666666667	0	4
3302	1	0.2	0	4
3304	1	0.2	0	4
3305	1	0.2	0	4
3306	1	0.2	0	4
3398	2	0.666666666666667	0	0
3410	1	0.333333333333333	0	4
3433	1	0.333333333333333	0	4

ptc-miR166n-q

category=2, cleavage_site=1717
query=ptc-miR166n-q, target=Potri.001G188800.1,
score=2.5, range=1706-1726, strand=1

```
target  5' CUGGAAUGAAGcCUGGUCCGG 3'
              ::::::::::::::::::::.
query   3' UUCCUUACUUCGGACCAGGCU 5'
```

>Potri.001G188800.1
#size=4154

1170	1	0.333333333333333	0	4	
1329	1	0.2	0	4	
1410	1	0.2	0	4	
1679	1	0.1	0	4	
1700	1	0.1	0	4	
1717	5	0.5	0	2	<<<
1718	1	0.1	0	4	
1730	1	0.1	0	4	
1766	1	0.333333333333333	0	4	
1784	1	0.333333333333333	0	4	

1799	1	0.166666666666667	0	4
1823	1	0.1	0	4
1900	1	0.166666666666667	0	4
1981	1	0.142857142857143	0	4
1998	1	0.142857142857143	0	4
2023	1	0.142857142857143	0	4
2026	1	0.142857142857143	0	4
2037	1	0.142857142857143	0	4
2043	1	0.142857142857143	0	4
2083	1	0.142857142857143	0	4
2095	1	0.142857142857143	0	4
2098	1	0.142857142857143	0	4
2113	1	0.142857142857143	0	4
2133	1	0.142857142857143	0	4
2139	2	0.285714285714286	0	2
2155	1	0.142857142857143	0	4
2169	1	0.25	0	4
2222	1	0.25	0	4
2289	1	0.142857142857143	0	4
2308	1	0.25	0	4
2319	1	0.25	0	4
2373	2	0.285714285714286	0	2
2412	1	0.142857142857143	0	4
2420	1	0.142857142857143	0	4
2421	1	0.142857142857143	0	4
2434	1	0.142857142857143	0	4
2435	1	0.142857142857143	0	4
2484	1	0.142857142857143	0	4
2496	1	0.25	0	4
2502	1	0.25	0	4
2585	1	0.142857142857143	0	4
2588	1	0.142857142857143	0	4
2630	1	0.25	0	4
2637	1	0.25	0	4
2640	1	0.25	0	4
2645	1	0.25	0	4
2647	1	0.142857142857143	0	4
2650	1	0.142857142857143	0	4
2660	1	0.25	0	4
2678	1	0.25	0	4
2691	1	0.25	0	4
2707	2	0.5	0	2
2720	1	0.25	0	4
2819	1	0.142857142857143	0	4
2824	1	0.142857142857143	0	4
2838	1	0.142857142857143	0	4
2848	1	0.142857142857143	0	4
2851	1	0.142857142857143	0	4
2874	1	0.142857142857143	0	4
2896	2	0.285714285714286	0	2
2898	2	0.285714285714286	0	2
2902	1	0.25	0	4
2924	1	0.25	0	4
2935	1	0.142857142857143	0	4
2952	1	0.142857142857143	0	4
2953	1	0.142857142857143	0	4
2954	1	0.142857142857143	0	4
2955	1	0.142857142857143	0	4

2965	1	0.142857142857143	0	4
3021	1	0.25	0	4
3122	2	0.333333333333333	0	2
3129	2	0.333333333333333	0	2
3137	1	0.333333333333333	0	4
3151	1	0.166666666666667	0	4
3153	1	0.166666666666667	0	4
3165	1	0.166666666666667	0	4
3187	1	0.333333333333333	0	4
3245	1	0.142857142857143	0	4
3253	1	0.142857142857143	0	4
3258	1	0.142857142857143	0	4
3267	1	0.142857142857143	0	4
3275	2	0.333333333333333	0	2
3276	2	0.333333333333333	0	2
3277	2	0.333333333333333	0	2
3278	1	0.166666666666667	0	4
3279	6	1	0	2
3283	1	0.166666666666667	0	4
3286	1	0.166666666666667	0	4
3289	1	0.166666666666667	0	4
3293	1	0.25	0	4
3294	1	0.166666666666667	0	4
3324	1	0.25	0	4
3331	1	0.25	0	4
3341	2	0.285714285714286	0	2
3353	1	0.142857142857143	0	4
3354	1	0.142857142857143	0	4
3356	1	0.142857142857143	0	4
3358	1	0.142857142857143	0	4
3361	1	0.142857142857143	0	4
3363	1	0.142857142857143	0	4
3365	1	0.142857142857143	0	4
3366	1	0.142857142857143	0	4
3367	1	0.25	0	4
3368	1	0.142857142857143	0	4
3379	1	0.25	0	4
3382	1	0.25	0	4
3383	3	0.75	0	2
3384	1	0.25	0	4
3385	1	0.25	0	4
3399	2	0.5	0	2
3416	2	0.5	0	2
3453	1	0.142857142857143	0	4
3455	1	0.142857142857143	0	4
3461	2	0.285714285714286	0	2
3472	2	0.285714285714286	0	2
3478	1	0.1	0	4
3490	1	0.1	0	4
3491	1	0.1	0	4
3492	1	0.1	0	4
3494	1	0.142857142857143	0	4
3496	1	0.142857142857143	0	4
3497	1	0.142857142857143	0	4
3498	2	0.285714285714286	0	2
3499	1	0.142857142857143	0	4
3500	1	0.142857142857143	0	4
3501	1	0.142857142857143	0	4

3502	1	0.142857142857143	0	4
3504	2	0.285714285714286	0	2
3518	1	0.142857142857143	0	4
3531	1	0.142857142857143	0	4
3532	1	0.142857142857143	0	4
3533	1	0.142857142857143	0	4
3534	1	0.142857142857143	0	4
3535	1	0.142857142857143	0	4
3559	1	0.142857142857143	0	4
3560	1	0.142857142857143	0	4
3583	1	0.142857142857143	0	4
3588	2	0.285714285714286	0	2
3600	1	0.25	0	4
3609	1	0.25	0	4
3611	1	0.25	0	4
3614	1	0.25	0	4
3624	1	0.25	0	4
3627	2	0.285714285714286	0	2
3628	1	0.142857142857143	0	4
3630	1	0.142857142857143	0	4
3631	1	0.142857142857143	0	4
3634	2	0.285714285714286	0	2
3636	1	0.142857142857143	0	4
3640	1	0.142857142857143	0	4
3641	1	0.142857142857143	0	4
3643	1	0.142857142857143	0	4
3644	2	0.285714285714286	0	2
3645	1	0.142857142857143	0	4
3646	1	0.142857142857143	0	4
3648	1	0.142857142857143	0	4
3652	1	0.142857142857143	0	4
3656	1	0.142857142857143	0	4
3658	1	0.142857142857143	0	4
3676	1	0.142857142857143	0	4
3679	2	0.285714285714286	0	2
3683	3	0.535714285714286	0	2
3706	8	2	0	0
3707	4	1	0	2
3708	1	0.25	0	4
3710	2	0.5	0	2
3711	1	0.25	0	4
3712	6	1.5	0	2
3713	6	1.5	0	2
3714	4	1	0	2
3715	1	0.25	0	4
3741	1	0.25	0	4
3744	1	0.25	0	4
3746	3	0.75	0	2
3748	2	0.5	0	2
3749	1	0.25	0	4
3750	1	0.25	0	4
3752	1	0.25	0	4
3753	1	0.25	0	4
3756	1	0.25	0	4
3773	1	0.25	0	4
3777	1	0.25	0	4
3789	6	1.5	0	2
3790	1	0.25	0	4

3798	1	0.25	0	4
3808	2	0.5	0	2
3823	2	0.5	0	2
3825	1	0.25	0	4
3827	1	0.25	0	4
3833	2	0.5	0	2
3834	1	0.25	0	4
3841	1	0.25	0	4
3844	1	0.25	0	4
3850	2	0.5	0	2
3858	2	0.5	0	2
3861	1	0.25	0	4
3866	2	0.5	0	2
3867	5	1.25	0	2
3868	1	0.25	0	4
3875	1	0.25	0	4
3877	1	0.25	0	4
3878	1	0.25	0	4
3880	1	0.25	0	4
3885	1	0.25	0	4
3887	5	1.25	0	2
3888	3	0.75	0	2
3889	1	0.25	0	4
3890	3	0.75	0	2
3892	3	0.75	0	2
3905	1	0.25	0	4
3975	1	0.25	0	4
4009	1	0.25	0	4

---

category=0, cleavage_site=1139
query=ptc-miR166n-q, target=Potri.001G372300.1,
score=3.5, range=1128-1148, strand=1

```
target  5' UUGGUAUGAAGcCUGGUCCGG 3'
             :: :::::::::::::::.
query   3' UUCCUUACUUCGGACCAGGCU 5'
```

>Potri.001G372300.1
#size=3340

154	2	2	2	2
170	1	1	1	4
175	1	1	1	4
176	1	1	1	4
185	4	4	4	2
189	1	1	1	4
193	1	1	1	4
202	1	1	1	4
280	2	2	2	2
290	1	1	1	4
298	1	1	1	4
307	1	1	1	4
326	1	0.5	0	4
335	1	0.5	0	4
337	2	1	0	2
338	1	0.5	0	4
346	1	0.5	0	4
428	1	1	1	4
488	1	1	1	4
561	1	0.5	0	4
578	1	0.5	0	4

609	1	0.5	0	4	
733	1	0.142857142857143	0	4	
736	1	0.142857142857143	0	4	
737	1	0.5	0	4	
742	1	0.5	0	4	
747	3	1.5	0	2	
766	1	0.333333333333333	0	4	
772	1	0.333333333333333	0	4	
817	1	0.333333333333333	0	4	
1135	1	0.333333333333333	0	4	
1139	24	8	0	0	<<<
1141	1	0.333333333333333	0	4	
1143	1	0.333333333333333	0	4	
1153	1	0.333333333333333	0	4	
1157	1	0.333333333333333	0	4	
1171	2	0.833333333333333	0	2	
1176	1	0.5	0	4	
1182	1	0.5	0	4	
1185	1	0.333333333333333	0	4	
1198	1	0.5	0	4	
1203	1	0.5	0	4	
1206	2	1	0	2	
1207	1	0.5	0	4	
1209	1	0.333333333333333	0	4	
1212	1	0.333333333333333	0	4	
1220	1	0.333333333333333	0	4	
1222	1	0.333333333333333	0	4	
1225	1	0.333333333333333	0	4	
1229	1	0.5	0	4	
1230	1	0.5	0	4	
1234	1	0.333333333333333	0	4	
1235	2	0.666666666666667	0	2	
1240	1	0.333333333333333	0	4	
1243	4	1.33333333333333	0	2	
1244	2	0.666666666666667	0	2	
1248	2	1	0	2	
1252	1	0.333333333333333	0	4	
1253	1	0.5	0	4	
1256	2	1	0	2	
1258	1	0.5	0	4	
1267	1	0.5	0	4	
1278	1	0.5	0	4	
1284	1	0.5	0	4	
1294	1	0.333333333333333	0	4	
1298	1	0.333333333333333	0	4	
1308	1	0.333333333333333	0	4	
1317	1	0.333333333333333	0	4	
1362	1	0.333333333333333	0	4	
1408	1	0.5	0	4	
1498	1	0.333333333333333	0	4	
1516	1	0.5	0	4	
1557	2	0.666666666666667	0	2	
1559	1	0.333333333333333	0	4	
1575	1	0.333333333333333	0	4	
1602	1	0.333333333333333	0	4	
1629	1	0.333333333333333	0	4	
1631	1	0.333333333333333	0	4	
1634	1	0.333333333333333	0	4	

1640	1	0.333333333333333	0	4
1741	1	0.333333333333333	0	4
1757	1	0.333333333333333	0	4
1875	1	0.5	0	4
1938	1	0.333333333333333	0	4
1957	1	0.333333333333333	0	4
1968	1	0.333333333333333	0	4
1969	1	0.333333333333333	0	4
1970	1	0.333333333333333	0	4
1976	1	0.333333333333333	0	4
1989	1	0.5	0	4
2004	1	0.5	0	4
2016	1	0.5	0	4
2030	1	0.333333333333333	0	4
2083	1	0.333333333333333	0	4
2117	1	0.333333333333333	0	4
2129	1	0.333333333333333	0	4
2134	1	0.333333333333333	0	4
2135	1	0.333333333333333	0	4
2141	1	0.333333333333333	0	4
2165	1	0.333333333333333	0	4
2173	1	0.5	0	4
2181	1	0.5	0	4
2188	1	0.5	0	4
2197	2	1	0	2
2200	1	0.5	0	4
2202	1	0.5	0	4
2204	1	0.5	0	4
2213	1	0.333333333333333	0	4
2223	1	0.333333333333333	0	4
2232	1	0.333333333333333	0	4
2247	1	0.333333333333333	0	4
2277	1	0.333333333333333	0	4
2295	1	0.333333333333333	0	4
2326	1	0.5	0	4
2328	1	0.333333333333333	0	4
2336	1	0.333333333333333	0	4
2337	1	0.333333333333333	0	4
2338	1	0.333333333333333	0	4
2433	1	0.333333333333333	0	4
2462	1	0.333333333333333	0	4
2466	1	0.5	0	4
2562	1	0.333333333333333	0	4
2578	1	0.5	0	4
2587	2	1	0	2
2608	2	1	0	2
2619	1	0.5	0	4
2639	1	0.5	0	4
2640	1	0.5	0	4
2645	1	0.5	0	4
2646	2	1	0	2
2647	2	1	0	2
2648	1	0.5	0	4
2650	1	0.5	0	4
2663	1	0.5	0	4
2671	1	0.333333333333333	0	4
2677	1	0.333333333333333	0	4
2678	1	0.333333333333333	0	4

2692	1	0.333333333333333	0	4
2694	1	0.333333333333333	0	4
2696	1	0.333333333333333	0	4
2700	1	0.333333333333333	0	4
2701	2	0.666666666666667	0	2
2705	2	0.666666666666667	0	2
2706	2	0.666666666666667	0	2
2710	2	0.666666666666667	0	2
2711	1	0.333333333333333	0	4
2714	2	0.666666666666667	0	2
2719	1	0.333333333333333	0	4
2720	1	0.5	0	4
2728	1	0.5	0	4
2738	1	0.333333333333333	0	4
2741	1	0.333333333333333	0	4
2752	1	0.333333333333333	0	4
2753	1	0.333333333333333	0	4
2754	1	0.333333333333333	0	4
2770	1	0.333333333333333	0	4
2776	2	0.666666666666667	0	2
2783	1	0.333333333333333	0	4
2830	1	0.333333333333333	0	4
2842	1	0.333333333333333	0	4
2847	2	0.666666666666667	0	2
2863	3	1	0	2
2865	1	0.333333333333333	0	4
2894	1	0.333333333333333	0	4
2903	1	0.333333333333333	0	4
2905	1	0.333333333333333	0	4
2906	1	0.333333333333333	0	4
2914	2	0.666666666666667	0	2
2923	1	0.5	0	4
2925	2	1	0	2
2927	2	1	0	2
2929	1	0.5	0	4
2932	3	1.5	0	2
2938	1	0.5	0	4
2954	1	0.333333333333333	0	4
2959	1	0.333333333333333	0	4
2966	1	0.333333333333333	0	4
2967	1	0.5	0	4
2969	1	0.333333333333333	0	4
2990	1	0.5	0	4
2993	2	1	0	2
2994	2	1	0	2
2995	2	1	0	2
2996	1	0.5	0	4
3003	1	0.5	0	4
3005	1	0.5	0	4
3006	1	0.5	0	4
3007	1	0.5	0	4
3008	1	0.5	0	4
3012	1	0.5	0	4
3015	2	1	0	2
3020	1	0.5	0	4
3022	1	0.5	0	4
3025	1	0.333333333333333	0	4
3027	1	0.333333333333333	0	4

3029	1	0.5	0	4
3044	1	0.5	0	4
3061	1	0.5	0	4
3081	1	0.5	0	4
3085	2	1	0	2
3088	1	0.5	0	4
3118	1	0.5	0	4
3146	1	0.5	0	4
3153	1	0.5	0	4
3186	1	0.5	0	4
3191	1	0.5	0	4
3211	1	0.5	0	4
3220	1	0.5	0	4

---

category=2, cleavage_site=1538
query=ptc-miR166n-q, target=Potri.003G050100.1,
score=2.5, range=1527-1547, strand=1

```
target  5' CUGGAAUGAAGcCUGGUCCGG 3'
             ::::::::::::::::::.
query   3' UUCCUUACUUCGGACCAGGCU 5'
```

>Potri.003G050100.1
#size=3807

481	1	1	1	4	
692	1	1	1	4	
834	1	1	1	4	
1150	1	0.2	0	4	
1154	1	0.25	0	4	
1165	1	0.5	0	4	
1231	1	0.2	0	4	
1500	1	0.1	0	4	
1521	1	0.1	0	4	
1538	4	0.4	0	2	<<<
1539	1	0.1	0	4	
1585	1	0.333333333333333	0	4	
1620	1	0.166666666666667	0	4	
1644	1	0.1	0	4	
1647	1	0.111111111111111	0	4	
1704	1	0.333333333333333	0	4	
1721	1	0.166666666666667	0	4	
1750	1	0.333333333333333	0	4	
1802	1	0.142857142857143	0	4	
1819	1	0.142857142857143	0	4	
1844	1	0.142857142857143	0	4	
1847	1	0.142857142857143	0	4	
1858	1	0.142857142857143	0	4	
1864	1	0.142857142857143	0	4	
1904	1	0.142857142857143	0	4	
1916	1	0.142857142857143	0	4	
1919	1	0.142857142857143	0	4	
1934	1	0.142857142857143	0	4	
1954	1	0.142857142857143	0	4	
1960	2	0.285714285714286	0	3	
1976	1	0.142857142857143	0	4	
2012	1	0.333333333333333	0	4	
2110	1	0.142857142857143	0	4	
2194	2	0.285714285714286	0	3	
2201	1	0.333333333333333	0	4	
2213	1	0.333333333333333	0	4	

2233	1	0.142857142857143	0	4
2241	1	0.142857142857143	0	4
2242	1	0.142857142857143	0	4
2255	1	0.142857142857143	0	4
2256	1	0.142857142857143	0	4
2305	1	0.142857142857143	0	4
2326	2	0.666666666666667	0	2
2327	1	0.333333333333333	0	4
2333	3	0.5	0	2
2334	1	0.166666666666667	0	4
2335	1	0.166666666666667	0	4
2343	1	0.166666666666667	0	4
2353	1	0.166666666666667	0	4
2355	2	0.333333333333333	0	2
2367	1	0.142857142857143	0	4
2370	1	0.142857142857143	0	4
2382	1	0.333333333333333	0	4
2429	1	0.142857142857143	0	4
2432	1	0.142857142857143	0	4
2441	1	0.333333333333333	0	4
2466	1	0.333333333333333	0	4
2493	1	0.333333333333333	0	4
2520	1	0.333333333333333	0	4
2532	4	1.33333333333333	0	2
2533	1	0.333333333333333	0	4
2541	3	1	0	2
2601	1	0.142857142857143	0	4
2606	1	0.142857142857143	0	4
2620	1	0.142857142857143	0	4
2630	1	0.142857142857143	0	4
2633	1	0.142857142857143	0	4
2656	1	0.142857142857143	0	4
2678	2	0.285714285714286	0	3
2680	2	0.285714285714286	0	3
2717	1	0.142857142857143	0	4
2734	1	0.142857142857143	0	4
2735	1	0.142857142857143	0	4
2736	1	0.142857142857143	0	4
2737	1	0.142857142857143	0	4
2747	1	0.142857142857143	0	4
2804	1	0.333333333333333	0	4
2815	1	0.333333333333333	0	4
2907	2	0.333333333333333	0	2
2914	2	0.333333333333333	0	2
2936	1	0.166666666666667	0	4
2938	1	0.166666666666667	0	4
2950	1	0.166666666666667	0	4
3027	1	0.142857142857143	0	4
3035	1	0.142857142857143	0	4
3040	1	0.142857142857143	0	4
3049	1	0.142857142857143	0	4
3057	2	0.333333333333333	0	2
3058	2	0.333333333333333	0	2
3059	2	0.333333333333333	0	2
3060	1	0.166666666666667	0	4
3061	6	1	0	2
3065	1	0.166666666666667	0	4
3068	1	0.166666666666667	0	4

3071	1	0.166666666666667	0	4
3076	1	0.166666666666667	0	4
3086	1	0.5	0	4
3097	1	0.333333333333333	0	4
3098	2	0.666666666666667	0	2
3105	1	0.333333333333333	0	4
3106	1	0.333333333333333	0	4
3107	1	0.333333333333333	0	4
3109	1	0.333333333333333	0	4
3110	2	0.666666666666667	0	2
3119	1	0.333333333333333	0	4
3123	2	0.285714285714286	0	3
3135	1	0.142857142857143	0	4
3136	1	0.142857142857143	0	4
3138	1	0.142857142857143	0	4
3140	2	0.476190476190476	0	2
3143	1	0.142857142857143	0	4
3145	1	0.142857142857143	0	4
3147	1	0.142857142857143	0	4
3148	1	0.142857142857143	0	4
3150	1	0.142857142857143	0	4
3157	1	0.333333333333333	0	4
3160	1	0.333333333333333	0	4
3162	1	0.333333333333333	0	4
3163	2	0.666666666666667	0	2
3164	1	0.333333333333333	0	4
3165	7	2.33333333333333	0	0
3193	1	0.333333333333333	0	4
3220	1	0.333333333333333	0	4
3223	2	0.666666666666667	0	2
3230	1	0.333333333333333	0	4
3234	1	0.333333333333333	0	4
3235	1	0.142857142857143	0	4
3237	1	0.142857142857143	0	4
3238	1	0.333333333333333	0	4
3243	2	0.285714285714286	0	3
3244	1	0.333333333333333	0	4
3254	2	0.285714285714286	0	3
3260	1	0.1	0	4
3274	1	0.1	0	4
3276	1	0.142857142857143	0	4
3278	1	0.142857142857143	0	4
3279	1	0.142857142857143	0	4
3280	2	0.285714285714286	0	3
3281	1	0.142857142857143	0	4
3282	1	0.142857142857143	0	4
3283	1	0.142857142857143	0	4
3284	1	0.142857142857143	0	4
3286	2	0.285714285714286	0	3
3297	1	0.333333333333333	0	4
3300	1	0.142857142857143	0	4
3313	1	0.142857142857143	0	4
3314	1	0.142857142857143	0	4
3315	1	0.142857142857143	0	4
3316	1	0.142857142857143	0	4
3317	1	0.142857142857143	0	4
3341	1	0.142857142857143	0	4
3342	1	0.142857142857143	0	4

3365	1	0.142857142857143	0	4
3370	2	0.285714285714286	0	3
3372	3	1 0 2		
3373	2	0.666666666666667	0	2
3384	1	0.333333333333333	0	4
3385	5	1.66666666666667	0	2
3386	1	0.333333333333333	0	4
3387	4	1.33333333333333	0	2
3398	1	0.333333333333333	0	4
3399	1	0.333333333333333	0	4
3400	1	0.333333333333333	0	4
3401	1	0.333333333333333	0	4
3402	4	1.33333333333333	0	2
3404	1	0.333333333333333	0	4
3406	1	0.333333333333333	0	4
3409	2	0.285714285714286	0	3
3410	1	0.142857142857143	0	4
3411	1	0.333333333333333	0	4
3412	1	0.142857142857143	0	4
3413	1	0.142857142857143	0	4
3416	2	0.285714285714286	0	3
3418	1	0.142857142857143	0	4
3422	1	0.142857142857143	0	4
3423	1	0.142857142857143	0	4
3425	1	0.142857142857143	0	4
3426	2	0.285714285714286	0	3
3427	1	0.142857142857143	0	4
3428	1	0.142857142857143	0	4
3430	1	0.142857142857143	0	4
3434	1	0.142857142857143	0	4
3438	1	0.142857142857143	0	4
3440	1	0.142857142857143	0	4
3458	1	0.142857142857143	0	4
3461	2	0.285714285714286	0	3
3465	3	0.619047619047619	0	2
3466	1	0.333333333333333	0	4
3467	1	0.333333333333333	0	4
3468	1	0.333333333333333	0	4
3470	2	0.666666666666667	0	2
3471	2	0.666666666666667	0	2
3474	1	0.333333333333333	0	4
3476	1	0.333333333333333	0	4
3477	1	0.333333333333333	0	4
3483	1	0.333333333333333	0	4
3484	1	0.333333333333333	0	4
3485	2	0.666666666666667	0	2
3486	1	0.333333333333333	0	4
3487	1	0.333333333333333	0	4
3488	4	1.33333333333333	0	2
3489	1	0.333333333333333	0	4
3491	1	0.333333333333333	0	4
3492	1	0.333333333333333	0	4
3495	4	1.33333333333333	0	2
3496	1	0.333333333333333	0	4
3497	1	0.333333333333333	0	4
3498	1	0.333333333333333	0	4
3499	1	0.333333333333333	0	4
3500	1	0.333333333333333	0	4

3501	1	0.333333333333333	0	4
3502	2	0.666666666666667	0	2
3515	1	0.333333333333333	0	4
3517	2	0.666666666666667	0	2
3518	1	0.333333333333333	0	4
3519	2	0.666666666666667	0	2
3520	1	0.333333333333333	0	4
3521	1	0.333333333333333	0	4
3523	1	0.333333333333333	0	4
3526	1	0.333333333333333	0	4
3532	1	0.333333333333333	0	4
3538	1	0.333333333333333	0	4
3539	1	0.333333333333333	0	4
3548	2	0.666666666666667	0	2
3555	1	0.333333333333333	0	4
3559	1	0.333333333333333	0	4
3568	1	0.333333333333333	0	4
3569	1	0.333333333333333	0	4
3572	1	0.333333333333333	0	4
3578	1	0.333333333333333	0	4
3581	1	0.333333333333333	0	4
3585	1	0.333333333333333	0	4
3586	1	0.333333333333333	0	4
3587	1	0.333333333333333	0	4
3588	1	0.333333333333333	0	4
3589	1	0.333333333333333	0	4
3590	1	0.333333333333333	0	4
3596	5	1.66666666666667	0	2
3599	1	0.333333333333333	0	4
3609	1	0.333333333333333	0	4
3647	1	0.333333333333333	0	4
3651	1	0.333333333333333	0	4
3694	1	0.333333333333333	0	4

---

category=4, cleavage_site=1310
query=ptc-miR166n-q, target=Potri.004G211300.1,
score=2.5, range=1299-1319, strand=1

```
target  5' CUGGAAUGAAGcCUGGUCCGG 3'
             ::::::::::::::::::.
query   3' UUCCUUACUUCGGACCAGGCU 5'
```

>Potri.004G211300.1
#size=3307

603	1	1	1	4	
616	1	1	1	4	
631	1	1	1	4	
928	1	0.2	0	4	
943	1	0.142857142857143	0	4	
946	1	0.142857142857143	0	4	
947	1	0.2	0	4	
1001	1	0.2	0	4	
1310	1	0.333333333333333	0	4	<<<
1466	1	0.142857142857143	0	4	
1650	1	0.142857142857143	0	4	
1755	1	0.142857142857143	0	4	
1864	1	0.142857142857143	0	4	
1911	1	0.142857142857143	0	4	
1932	1	0.142857142857143	0	4	
2074	1	0.333333333333333	0	4	

2158	1	0.142857142857143	0	4
2169	1	0.142857142857143	0	4
2170	1	0.142857142857143	0	4
2174	1	0.142857142857143	0	4
2301	1	0.142857142857143	0	4
2310	1	0.142857142857143	0	4
2360	1	0.142857142857143	0	4
2402	1	0.142857142857143	0	4
2515	1	0.333333333333333	0	4
2551	1	0.333333333333333	0	4
2639	1	0.142857142857143	0	4
2723	1	0.142857142857143	0	4
2743	2	0.285714285714286	0	2
2765	1	0.142857142857143	0	4
2805	1	0.333333333333333	0	4
2809	1	0.333333333333333	0	4
2876	1	0.142857142857143	0	4
2907	1	0.142857142857143	0	4
2908	1	0.142857142857143	0	4
2940	1	0.142857142857143	0	4
3013	1	0.142857142857143	0	4
3021	1	0.142857142857143	0	4
3022	1	0.142857142857143	0	4
3103	1	0.333333333333333	0	4
3106	1	0.2	0	4
3140	1	0.142857142857143	0	4
3157	1	0.142857142857143	0	4
3158	1	0.142857142857143	0	4
3160	1	0.142857142857143	0	4
3169	1	0.142857142857143	0	4
3172	1	0.333333333333333	0	4
3174	1	0.333333333333333	0	4
3176	1	0.333333333333333	0	4
3188	1	0.333333333333333	0	4
3205	1	0.333333333333333	0	4
3239	1	0.142857142857143	0	4
3261	1	0.166666666666667	0	4

---

category=2, cleavage_site=583
query=ptc-miR166n-q, target=Potri.006G237500.1,
score=3, range=572-592, strand=1

```
target  5' CUGGGAUGAAGcCUGGUCCGG 3'
              ::.:::::::::::::::::.
query   3' UUCCUUACUUCGGACCAGGCU 5'
```

>Potri.006G237500.1
#size=2787

3	1	0.166666666666667	0	4	
189	1	0.166666666666667	0	4	
196	1	0.25	0	4	
545	1	0.1	0	4	
566	1	0.1	0	4	
583	6	0.6	0	2	<<<
584	1	0.1	0	4	
596	1	0.1	0	4	
692	1	0.111111111111111	0	4	
706	1	0.166666666666667	0	4	
736	1	0.5	0	4	
760	1	0.166666666666667	0	4	

836	1	0.166666666666667	0	4
864	1	0.166666666666667	0	4
1024	1	0.5	0	4
1252	1	0.166666666666667	0	4
1418	1	0.166666666666667	0	4
1442	1	0.166666666666667	0	4
1479	1	0.5	0	4
1487	1	0.5	0	4
1557	2	0.333333333333333	0	2
1632	1	0.166666666666667	0	4
1663	2	1	0	1
1700	2	1	0	1
1820	1	0.5	0	4
1912	1	0.5	0	4
1913	1	0.5	0	4
1917	2	1	0	1
1947	1	0.166666666666667	0	4
2000	1	0.166666666666667	0	4
2016	1	0.5	0	4
2031	1	0.166666666666667	0	4
2132	1	0.5	0	4
2137	1	0.5	0	4
2144	1	0.5	0	4
2154	1	0.5	0	4
2170	1	0.166666666666667	0	4
2174	1	0.166666666666667	0	4
2177	1	0.166666666666667	0	4
2178	1	0.166666666666667	0	4
2193	1	0.5	0	4
2200	1	0.5	0	4
2237	1	0.166666666666667	0	4
2248	1	0.5	0	4
2270	1	0.166666666666667	0	4
2272	1	0.166666666666667	0	4
2284	1	0.166666666666667	0	4
2300	1	0.166666666666667	0	4
2304	1	0.166666666666667	0	4
2305	1	0.166666666666667	0	4
2311	1	0.1	0	4
2323	1	0.1	0	4
2324	1	0.1	0	4
2346	1	0.166666666666667	0	4
2348	2	0.333333333333333	0	2
2352	1	0.166666666666667	0	4
2366	1	0.166666666666667	0	4
2368	1	0.166666666666667	0	4
2380	1	0.166666666666667	0	4
2391	1	0.166666666666667	0	4
2399	1	0.166666666666667	0	4
2403	1	0.166666666666667	0	4
2420	1	0.166666666666667	0	4
2448	1	0.166666666666667	0	4
2449	1	0.5	0	4
2465	1	0.166666666666667	0	4
2468	1	0.5	0	4
2478	1	0.166666666666667	0	4
2481	1	0.166666666666667	0	4
2484	1	0.166666666666667	0	4

2487	1	0.166666666666667	0	4
2496	1	0.166666666666667	0	4
2508	1	0.2	0	4
2510	1	0.2	0	4
2511	1	0.2	0	4
2512	1	0.2	0	4
2522	1	0.5	0	4
2523	2	1	0	1
2533	1	0.5	0	4
2536	1	0.5	0	4
2537	1	0.5	0	4
2545	1	0.5	0	4
2547	1	0.5	0	4
2549	1	0.5	0	4
2553	1	0.5	0	4
2563	2	1	0	1
2583	1	0.5	0	4
2650	1	0.5	0	4

---

category=4, cleavage_site=574
query=ptc-miR166n-q, target=Potri.009G014500.1,
score=3, range=563-583, strand=1

```
target  5' CUGGGAUGAAGcCUGGUCCGG 3'
              ::.:::::::::::::::.
query   3' UUCCUUACUUCGGACCAGGCU 5'
```

>Potri.009G014500.1
#size=2568

70	1	0.25	0	4	
108	1	0.25	0	4	
151	1	0.25	0	4	
169	1	0.25	0	4	
186	1	0.2	0	4	
201	1	0.142857142857143	0	4	
204	1	0.142857142857143	0	4	
205	1	0.2	0	4	
259	1	0.2	0	4	
533	1	0.25	0	4	
574	1	0.25	0	4	<<<
591	1	0.25	0	4	
730	1	0.142857142857143	0	4	
872	1	0.25	0	4	
914	1	0.142857142857143	0	4	
1022	1	0.142857142857143	0	4	
1084	1	0.25	0	4	
1131	1	0.142857142857143	0	4	
1178	1	0.142857142857143	0	4	
1199	1	0.142857142857143	0	4	
1331	1	0.25	0	4	
1337	1	0.25	0	4	
1347	1	0.25	0	4	
1368	2	0.5	0	1	
1425	1	0.142857142857143	0	4	
1436	1	0.142857142857143	0	4	
1437	1	0.142857142857143	0	4	
1441	1	0.142857142857143	0	4	
1458	1	0.25	0	4	
1491	1	0.25	0	4	
1515	1	0.25	0	4	

1527	1	0.25	0	4
1530	1	0.25	0	4
1568	1	0.142857142857143	0	4
1577	1	0.142857142857143	0	4
1627	1	0.142857142857143	0	4
1669	1	0.142857142857143	0	4
1765	1	0.25	0	4
1815	1	0.25	0	4
1903	1	0.142857142857143	0	4
1987	1	0.142857142857143	0	4
2007	2	0.285714285714286	0	2
2029	1	0.142857142857143	0	4
2093	1	0.25	0	4
2152	1	0.142857142857143	0	4
2183	1	0.142857142857143	0	4
2184	1	0.142857142857143	0	4
2216	1	0.142857142857143	0	4
2289	1	0.142857142857143	0	4
2297	1	0.142857142857143	0	4
2298	1	0.142857142857143	0	4
2309	1	0.25	0	4
2322	2	0.5	0	1
2324	1	0.25	0	4
2341	2	0.5	0	1
2367	1	0.25	0	4
2425	1	0.142857142857143	0	4
2442	1	0.142857142857143	0	4
2443	1	0.142857142857143	0	4
2445	1	0.142857142857143	0	4
2452	1	0.25	0	4
2454	1	0.142857142857143	0	4
2456	1	0.25	0	4
2457	1	0.25	0	4
2460	2	0.5	0	1
2491	1	0.25	0	4
2514	2	0.5	0	1
2517	1	0.25	0	4
2518	1	0.25	0	4
2519	2	0.5	0	1
2524	1	0.142857142857143	0	4

ptc-miR167a-d

category=0, cleavage_site=2408
query=ptc-miR167a-d, target=Potri.004G078200.1,
score=4, range=2397-2418, strand=1

```
target  5' UAGAUCAGGCUgGCAGCUUGUA 3'
           ::::::: :::::::::: .:
query   3' AUCUAGUACGACCGUCGAA-GU 5'
```

>Potri.004G078200.1
#size=3078

147	1	0.2	0	4
217	2	0.4	0	2
241	1	0.2	0	4
280	2	0.333333333333333	0	2
305	1	0.166666666666667	0	4
316	2	0.333333333333333	0	2
318	3	0.5	0	2

337	1	0.166666666666667	0	4
347	1	0.166666666666667	0	4
542	1	0.2	0	4
582	1	0.142857142857143	0	4
621	1	0.142857142857143	0	4
743	1	0.142857142857143	0	4
757	1	0.142857142857143	0	4
845	1	0.1	0	4
846	1	0.1	0	4
896	1	0.166666666666667	0	4
913	1	0.166666666666667	0	4
982	2	0.285714285714286	0	2
989	1	0.142857142857143	0	4
1002	1	0.142857142857143	0	4
1023	1	0.1	0	4
1061	1	0.142857142857143	0	4
1104	3	0.428571428571429	0	2
1111	1	0.142857142857143	0	4
1132	1	0.142857142857143	0	4
1146	1	0.2	0	4
1208	1	0.142857142857143	0	4
1209	2	0.285714285714286	0	2
1216	1	0.142857142857143	0	4
1221	1	0.142857142857143	0	4
1222	1	0.142857142857143	0	4
1236	1	0.2	0	4
1247	1	0.2	0	4
1274	1	0.125	0	4
1305	1	0.2	0	4
1324	1	0.125	0	4
1325	1	0.125	0	4
1342	1	0.125	0	4
1394	1	0.125	0	4
1435	1	0.125	0	4
1458	1	0.125	0	4
1468	1	0.125	0	4
1489	1	0.2	0	4
1494	1	0.2	0	4
1498	1	0.2	0	4
1522	1	0.2	0	4
1535	1	0.2	0	4
1557	1	0.125	0	4
1562	1	0.2	0	4
1580	1	0.2	0	4
1627	1	0.125	0	4
1639	1	0.125	0	4
1640	1	0.125	0	4
1644	1	0.125	0	4
1654	1	0.125	0	4
1660	1	0.125	0	4
1669	1	0.125	0	4
1682	1	0.125	0	4
1684	1	0.2	0	4
1706	1	0.2	0	4
1718	1	0.2	0	4
1725	1	0.125	0	4
1726	1	0.125	0	4
1741	1	0.125	0	4

1768	1	0.125	0	4
1779	1	0.125	0	4
1800	1	0.2	0	4
1807	1	0.2	0	4
1811	1	0.2	0	4
1815	1	0.2	0	4
1819	1	0.2	0	4
1830	1	0.125	0	4
1872	1	0.2	0	4
1907	2	0.25	0	2
1909	1	0.125	0	4
1914	1	0.125	0	4
1918	1	0.125	0	4
1943	1	0.2	0	4
1957	1	0.2	0	4
1989	1	0.2	0	4
1999	1	0.2	0	4
2007	1	0.2	0	4
2017	1	0.2	0	4
2018	1	0.2	0	4
2103	1	0.2	0	4
2138	3	0.6	0	2
2173	1	0.125	0	4
2176	1	0.125	0	4
2196	1	0.125	0	4
2213	1	0.2	0	4
2225	1	0.125	0	4
2231	1	0.125	0	4
2232	1	0.125	0	4
2235	1	0.125	0	4
2239	1	0.125	0	4
2246	1	0.125	0	4
2251	2	0.25	0	2
2270	1	0.125	0	4
2276	1	0.125	0	4
2284	1	0.125	0	4
2287	1	0.125	0	4
2293	1	0.125	0	4
2296	2	0.25	0	2
2297	1	0.125	0	4
2298	1	0.125	0	4
2304	1	0.125	0	4
2307	1	0.125	0	4
2314	1	0.125	0	4
2333	1	0.2	0	4
2337	1	0.125	0	4
2349	2	0.25	0	2
2354	1	0.125	0	4
2357	1	0.2	0	4
2368	2	0.25	0	2
2369	1	0.125	0	4
2370	1	0.125	0	4
2373	1	0.125	0	4
2375	1	0.125	0	4
2377	1	0.125	0	4
2379	1	0.125	0	4
2383	1	0.125	0	4
2395	1	0.125	0	4

2396	1	0.125	0	4	
2404	1	0.1	0	4	
2408	14	1.725	0	0	<<<
2411	1	0.125	0	4	
2413	1	0.1	0	4	
2435	1	0.125	0	4	
2437	1	0.125	0	4	
2445	1	0.125	0	4	
2447	1	0.125	0	4	
2452	1	0.125	0	4	
2473	1	0.125	0	4	
2481	1	0.125	0	4	
2482	2	0.25	0	2	
2484	1	0.125	0	4	
2491	1	0.2	0	4	
2497	1	0.2	0	4	
2535	1	0.2	0	4	
2537	1	0.2	0	4	
2561	2	0.4	0	2	
2565	1	0.2	0	4	
2568	2	0.4	0	2	
2572	2	0.4	0	2	
2574	1	0.2	0	4	
2575	1	0.2	0	4	
2583	1	0.2	0	4	
2591	1	0.2	0	4	
2598	1	0.2	0	4	
2599	2	0.4	0	2	
2600	1	0.2	0	4	
2608	1	0.2	0	4	
2616	1	0.2	0	4	
2617	1	0.2	0	4	
2621	1	0.2	0	4	
2624	1	0.2	0	4	
2626	3	0.6	0	2	
2630	2	0.4	0	2	
2631	1	0.2	0	4	
2636	1	0.2	0	4	
2676	1	0.2	0	4	
2677	2	0.4	0	2	
2678	2	0.4	0	2	
2680	1	0.2	0	4	
2687	1	0.2	0	4	
2689	1	0.2	0	4	
2692	2	0.4	0	2	
2693	1	0.2	0	4	
2703	1	0.2	0	4	
2710	1	0.2	0	4	
2712	2	0.4	0	2	
2714	1	0.2	0	4	
2719	2	0.4	0	2	
2724	1	0.2	0	4	
2726	1	0.2	0	4	
2745	1	0.2	0	4	
2756	1	0.2	0	4	
2759	1	0.2	0	4	
2781	1	0.2	0	4	
2791	1	0.2	0	4	

2792	1	0.2	0	4
2793	1	0.2	0	4
2798	1	0.2	0	4
2818	1	0.2	0	4
2881	1	0.125	0	4

---

category=4, cleavage_site=2420
query=ptc-miR167a-d, target=Potri.014G114300.1,
score=3.5, range=2409-2429, strand=1

```
target  5' UGGAUGAUGUUgGUAGCUUCA 3'
           :.::: :::.:::.:::::::
query   3' AUCUAGUACGACCGUCGAAGU 5'
```

>Potri.014G114300.1
#size=3803

53	1	0.5	0	4
82	1	0.5	0	4
85	1	0.5	0	4
169	1	0.5	0	4
334	2	0.285714285714286	0	2
363	2	0.285714285714286	0	2
380	1	0.142857142857143	0	4
388	1	0.142857142857143	0	4
428	1	0.5	0	4
442	1	0.5	0	4
457	1	0.5	0	4
512	1	0.5	0	4
526	1	0.5	0	4
574	1	0.5	0	4
575	1	0.5	0	4
632	2	0.285714285714286	0	2
635	1	0.142857142857143	0	4
650	1	0.111111111111111	0	4
746	1	0.5	0	4
754	1	0.5	0	4
764	1	0.5	0	4
770	2	1	0	2
774	1	0.142857142857143	0	4
777	1	0.142857142857143	0	4
787	1	0.142857142857143	0	4
798	1	0.142857142857143	0	4
803	2	0.285714285714286	0	2
807	1	0.142857142857143	0	4
837	1	0.142857142857143	0	4
874	1	0.5	0	4
910	1	0.5	0	4
1006	1	0.142857142857143	0	4
1012	1	0.142857142857143	0	4
1022	1	0.142857142857143	0	4
1073	1	0.142857142857143	0	4
1156	1	0.142857142857143	0	4
1160	1	0.142857142857143	0	4
1162	1	0.142857142857143	0	4
1164	1	0.142857142857143	0	4
1168	1	0.142857142857143	0	4
1170	1	0.142857142857143	0	4
1172	1	0.142857142857143	0	4
1173	1	0.142857142857143	0	4

1174	1	0.142857142857143	0	4
1187	1	0.142857142857143	0	4
1204	1	0.5	0	4
1223	1	0.5	0	4
1232	1	0.5	0	4
1253	1	0.142857142857143	0	4
1255	1	0.142857142857143	0	4
1257	1	0.142857142857143	0	4
1260	1	0.142857142857143	0	4
1261	1	0.142857142857143	0	4
1263	1	0.142857142857143	0	4
1264	1	0.142857142857143	0	4
1266	1	0.142857142857143	0	4
1275	1	0.142857142857143	0	4
1280	2	0.285714285714286	0	2
1286	1	0.142857142857143	0	4
1287	1	0.142857142857143	0	4
1305	1	0.5	0	4
1306	2	0.333333333333333	0	2
1307	1	0.166666666666667	0	4
1311	1	0.5	0	4
1351	1	0.166666666666667	0	4
1382	1	0.5	0	4
1388	1	0.5	0	4
1403	1	0.5	0	4
1414	1	0.5	0	4
1422	1	0.142857142857143	0	4
1426	3	1.14285714285714	0	2
1487	2	1	0	2
1492	1	0.5	0	4
1547	1	0.5	0	4
1548	1	0.5	0	4
1604	1	0.142857142857143	0	4
1612	1	0.142857142857143	0	4
1619	1	0.5	0	4
1639	1	0.142857142857143	0	4
1700	2	0.285714285714286	0	2
1703	1	0.142857142857143	0	4
1708	1	0.142857142857143	0	4
1710	1	0.142857142857143	0	4
1717	1	0.142857142857143	0	4
1718	2	0.285714285714286	0	2
1719	2	0.285714285714286	0	2
1728	1	0.142857142857143	0	4
1729	1	0.142857142857143	0	4
1731	1	0.142857142857143	0	4
1733	1	0.142857142857143	0	4
1737	1	0.142857142857143	0	4
1738	1	0.142857142857143	0	4
1747	4	0.571428571428571	0	2
1758	2	0.285714285714286	0	2
1761	1	0.142857142857143	0	4
1774	1	0.142857142857143	0	4
1790	1	0.142857142857143	0	4
1811	1	0.142857142857143	0	4
1852	1	0.142857142857143	0	4
1865	1	0.142857142857143	0	4
1879	1	0.142857142857143	0	4

1889	1	0.142857142857143			0	4	
1941	1	0.142857142857143			0	4	
1978	2	1	0	2			
2017	1	0.142857142857143			0	4	
2170	1	0.142857142857143			0	4	
2233	1	0.5	0	4			
2247	1	0.142857142857143			0	4	
2295	1	0.5	0	4			
2309	2	1	0	2			
2312	1	0.5	0	4			
2328	1	0.142857142857143			0	4	
2339	4	0.571428571428571			0	2	
2345	1	0.142857142857143			0	4	
2356	1	0.142857142857143			0	4	
2357	1	0.5	0	4			
2403	1	0.5	0	4			
2408	1	0.142857142857143			0	4	
2420	1	0.142857142857143			0	4	<<<
2429	1	0.142857142857143			0	4	
2436	1	0.142857142857143			0	4	
2451	1	0.5	0	4			
2457	1	0.5	0	4			
2461	1	0.5	0	4			
2470	1	0.5	0	4			
2481	1	0.142857142857143			0	4	
2493	1	0.5	0	4			
2498	2	1	0	2			
2499	1	0.5	0	4			
2546	1	0.142857142857143			0	4	
2587	1	0.5	0	4			
2614	1	0.142857142857143			0	4	
2667	1	0.142857142857143			0	4	
2690	1	0.5	0	4			
2695	1	0.5	0	4			
2766	1	0.142857142857143			0	4	
2881	1	0.142857142857143			0	4	
2887	1	0.142857142857143			0	4	
2910	1	0.142857142857143			0	4	
2911	1	0.142857142857143			0	4	
2915	1	0.142857142857143			0	4	
2930	1	0.142857142857143			0	4	
2932	1	0.142857142857143			0	4	
2937	1	0.142857142857143			0	4	
2955	1	0.5	0	4			
2959	1	0.5	0	4			
2966	1	0.5	0	4			
2972	1	0.5	0	4			
2994	2	0.285714285714286			0	2	
2997	1	0.142857142857143			0	4	
3003	1	0.142857142857143			0	4	
3007	1	0.142857142857143			0	4	
3022	1	0.142857142857143			0	4	
3056	1	0.166666666666667			0	4	
3098	1	1	1	4			
3109	1	0.2	0	4			
3111	1	0.2	0	4			
3118	1	0.2	0	4			
3121	1	1	1	4			

3135	1	0.2	0	4
3142	1	1	1	4
3145	1	1	1	4
3160	1	0.2	0	4
3165	5	1	0	2
3175	1	1	1	4
3180	2	0.4	0	2
3181	2	0.4	0	2
3199	1	1	1	4
3211	1	1	1	4
3220	1	1	1	4
3233	1	1	1	4
3239	1	1	1	4
3243	1	1	1	4
3255	1	1	1	4
3284	1	1	1	4
3296	1	1	1	4
3375	1	0.2	0	4
3376	1	0.2	0	4
3390	1	0.2	0	4
3404	1	0.5	0	4
3408	1	0.5	0	4
3410	1	0.166666666666667	0	4
3418	2	0.333333333333333	0	2
3434	1	0.166666666666667	0	4
3437	1	0.166666666666667	0	4
3438	1	0.166666666666667	0	4
3458	1	0.166666666666667	0	4
3471	1	0.5	0	4
3477	1	0.5	0	4
3478	1	0.5	0	4
3480	1	0.5	0	4
3496	2	1	0	2
3498	1	0.5	0	4
3502	1	0.5	0	4
3503	2	1	0	2
3505	2	1	0	2
3518	1	0.5	0	4
3537	1	0.5	0	4
3539	1	0.5	0	4
3544	4	1.64285714285714	0	0
3546	1	0.5	0	4
3549	1	0.5	0	4
3555	1	0.5	0	4
3557	1	0.5	0	4
3559	1	0.5	0	4
3562	1	0.5	0	4
3576	1	0.5	0	4
3577	1	0.5	0	4
3589	1	0.5	0	4
3638	1	0.5	0	4
3660	1	0.5	0	4
3665	1	0.5	0	4
3685	1	0.5	0	4

category=0, cleavage_site=2865
query=ptc-miR167a-d, target=Potri.017G141000.1,
score=4, range=2854-2875, strand=1

```
target  5' UAGAUCAGGCUgGCAGCUUGUA 3'
           ::::::: :::::::::: .:
query   3' AUCUAGUACGACCGUCGAA-GU 5'
```

>Potri.017G141000.1
#size=3472

331	1	0.5	0	4
498	1	0.5	0	4
631	1	0.2	0	4
673	1	0.5	0	4
701	2	0.4	0	2
725	1	0.2	0	4
759	1	0.5	0	4
764	2	0.333333333333333	0	2
789	2	0.666666666666667	0	2
800	2	0.333333333333333	0	2
802	3	0.5	0	2
821	1	0.166666666666667	0	4
909	1	0.5	0	4
922	1	0.5	0	4
969	1	0.142857142857143	0	4
1066	1	0.142857142857143	0	4
1105	1	0.142857142857143	0	4
1227	1	0.142857142857143	0	4
1241	1	0.142857142857143	0	4
1329	1	0.1	0	4
1330	1	0.1	0	4
1380	1	0.166666666666667	0	4
1397	1	0.166666666666667	0	4
1466	2	0.285714285714286	0	2
1473	1	0.142857142857143	0	4
1486	1	0.142857142857143	0	4
1507	2	0.2	0	2
1545	1	0.142857142857143	0	4
1588	3	0.428571428571429	0	2
1595	1	0.142857142857143	0	4
1616	1	0.142857142857143	0	4
1692	1	0.142857142857143	0	4
1693	2	0.285714285714286	0	2
1700	1	0.142857142857143	0	4
1705	1	0.142857142857143	0	4
1706	1	0.142857142857143	0	4
1758	1	0.125	0	4
1759	1	0.333333333333333	0	4
1808	1	0.125	0	4
1809	1	0.125	0	4
1826	1	0.125	0	4
1878	1	0.125	0	4
1919	1	0.125	0	4
1942	1	0.125	0	4
1952	1	0.125	0	4
2035	1	0.125	0	4
2086	1	0.333333333333333	0	4
2092	1	0.333333333333333	0	4
2099	2	0.666666666666667	0	2
2105	1	0.125	0	4

2117	1	0.125	0	4
2118	1	0.125	0	4
2122	1	0.125	0	4
2132	1	0.125	0	4
2138	1	0.125	0	4
2147	1	0.125	0	4
2160	1	0.125	0	4
2193	1	0.333333333333333	0	4
2203	1	0.125	0	4
2204	1	0.125	0	4
2219	1	0.125	0	4
2246	1	0.125	0	4
2257	1	0.125	0	4
2264	1	0.333333333333333	0	4
2273	1	0.333333333333333	0	4
2308	1	0.125	0	4
2315	1	0.333333333333333	0	4
2322	1	0.333333333333333	0	4
2338	1	0.333333333333333	0	4
2378	1	0.333333333333333	0	4
2385	2	0.25	0	2
2387	1	0.125	0	4
2392	1	0.125	0	4
2396	1	0.125	0	4
2405	1	0.333333333333333	0	4
2482	1	0.333333333333333	0	4
2483	1	0.333333333333333	0	4
2493	1	0.333333333333333	0	4
2512	1	0.333333333333333	0	4
2525	1	0.333333333333333	0	4
2530	1	0.333333333333333	0	4
2535	1	0.333333333333333	0	4
2537	2	0.666666666666667	0	2
2539	3	1	0	2
2545	1	0.333333333333333	0	4
2546	1	0.333333333333333	0	4
2548	1	0.333333333333333	0	4
2630	1	0.125	0	4
2633	1	0.125	0	4
2653	1	0.125	0	4
2682	1	0.125	0	4
2688	1	0.125	0	4
2689	1	0.125	0	4
2692	1	0.125	0	4
2696	1	0.125	0	4
2703	1	0.125	0	4
2705	1	0.333333333333333	0	4
2708	2	0.25	0	2
2727	1	0.125	0	4
2733	1	0.125	0	4
2741	1	0.125	0	4
2744	1	0.125	0	4
2750	1	0.125	0	4
2753	2	0.25	0	2
2754	1	0.125	0	4
2755	1	0.125	0	4
2761	1	0.125	0	4
2764	1	0.125	0	4

2771	1	0.125	0	4	
2794	1	0.125	0	4	
2806	2	0.25	0	2	
2811	1	0.125	0	4	
2825	2	0.25	0	2	
2826	1	0.125	0	4	
2827	1	0.125	0	4	
2830	1	0.125	0	4	
2832	1	0.125	0	4	
2834	1	0.125	0	4	
2836	1	0.125	0	4	
2840	1	0.125	0	4	
2852	1	0.125	0	4	
2853	1	0.125	0	4	
2861	1	0.1	0	4	
2865	14	1.725	0	0	<<<
2868	1	0.125	0	4	
2892	1	0.125	0	4	
2894	1	0.125	0	4	
2902	1	0.125	0	4	
2904	1	0.125	0	4	
2909	1	0.125	0	4	
2930	1	0.125	0	4	
2938	1	0.125	0	4	
2939	2	0.25	0	2	
2941	1	0.125	0	4	
2942	1	0.333333333333333	0	4	
2947	1	0.333333333333333	0	4	
2951	1	0.333333333333333	0	4	
2961	1	0.333333333333333	0	4	
2962	1	0.333333333333333	0	4	
2973	1	0.333333333333333	0	4	
2984	1	0.333333333333333	0	4	
2985	1	0.333333333333333	0	4	
3003	1	0.333333333333333	0	4	
3004	1	0.333333333333333	0	4	
3018	1	0.333333333333333	0	4	
3025	1	0.333333333333333	0	4	
3075	1	0.333333333333333	0	4	
3178	1	0.333333333333333	0	4	
3179	1	0.333333333333333	0	4	
3185	2	0.666666666666667	0	2	
3188	1	0.333333333333333	0	4	
3189	1	0.333333333333333	0	4	
3197	1	0.333333333333333	0	4	
3244	1	0.333333333333333	0	4	
3246	1	0.333333333333333	0	4	
3269	1	0.333333333333333	0	4	
3275	1	0.333333333333333	0	4	
3343	1	0.125	0	4	

ptc-miR167e

category=4, cleavage_site=2420
query=ptc-miR167e, target=Potri.014G114300.1,
score=4, range=2409-2429, strand=1

```
target  5' UGGAUGAUGUUgGUAGCUUCA 3'
             ..::: :::.:::.::::::
query   3' GUCUAGUACGACCGUCGAAGU 5'
```

>Potri.014G114300.1
#size=3803

53	1	0.5	0	4
82	1	0.5	0	4
85	1	0.5	0	4
169	1	0.5	0	4
334	2	0.285714285714286	0	2
363	2	0.285714285714286	0	2
380	1	0.142857142857143	0	4
388	1	0.142857142857143	0	4
428	1	0.5	0	4
442	1	0.5	0	4
457	1	0.5	0	4
512	1	0.5	0	4
526	1	0.5	0	4
574	1	0.5	0	4
575	1	0.5	0	4
632	2	0.285714285714286	0	2
635	1	0.142857142857143	0	4
650	1	0.111111111111111	0	4
746	1	0.5	0	4
754	1	0.5	0	4
764	1	0.5	0	4
770	2	1	0	2
774	1	0.142857142857143	0	4
777	1	0.142857142857143	0	4
787	1	0.142857142857143	0	4
798	1	0.142857142857143	0	4
803	2	0.285714285714286	0	2
807	1	0.142857142857143	0	4
837	1	0.142857142857143	0	4
874	1	0.5	0	4
910	1	0.5	0	4
1006	1	0.142857142857143	0	4
1012	1	0.142857142857143	0	4
1022	1	0.142857142857143	0	4
1073	1	0.142857142857143	0	4
1156	1	0.142857142857143	0	4
1160	1	0.142857142857143	0	4
1162	1	0.142857142857143	0	4
1164	1	0.142857142857143	0	4
1168	1	0.142857142857143	0	4
1170	1	0.142857142857143	0	4
1172	1	0.142857142857143	0	4
1173	1	0.142857142857143	0	4
1174	1	0.142857142857143	0	4
1187	1	0.142857142857143	0	4
1204	1	0.5	0	4
1223	1	0.5	0	4
1232	1	0.5	0	4
1253	1	0.142857142857143	0	4
1255	1	0.142857142857143	0	4
1257	1	0.142857142857143	0	4
1260	1	0.142857142857143	0	4
1261	1	0.142857142857143	0	4
1263	1	0.142857142857143	0	4
1264	1	0.142857142857143	0	4
1266	1	0.142857142857143	0	4

1275	1	0.142857142857143	0	4
1280	2	0.285714285714286	0	2
1286	1	0.142857142857143	0	4
1287	1	0.142857142857143	0	4
1305	1	0.5	0	4
1306	2	0.333333333333333	0	2
1307	1	0.166666666666667	0	4
1311	1	0.5	0	4
1351	1	0.166666666666667	0	4
1382	1	0.5	0	4
1388	1	0.5	0	4
1403	1	0.5	0	4
1414	1	0.5	0	4
1422	1	0.142857142857143	0	4
1426	3	1.14285714285714	0	2
1487	2	1	0	2
1492	1	0.5	0	4
1547	1	0.5	0	4
1548	1	0.5	0	4
1604	1	0.142857142857143	0	4
1612	1	0.142857142857143	0	4
1619	1	0.5	0	4
1639	1	0.142857142857143	0	4
1700	2	0.285714285714286	0	2
1703	1	0.142857142857143	0	4
1708	1	0.142857142857143	0	4
1710	1	0.142857142857143	0	4
1717	1	0.142857142857143	0	4
1718	2	0.285714285714286	0	2
1719	2	0.285714285714286	0	2
1728	1	0.142857142857143	0	4
1729	1	0.142857142857143	0	4
1731	1	0.142857142857143	0	4
1733	1	0.142857142857143	0	4
1737	1	0.142857142857143	0	4
1738	1	0.142857142857143	0	4
1747	4	0.571428571428571	0	2
1758	2	0.285714285714286	0	2
1761	1	0.142857142857143	0	4
1774	1	0.142857142857143	0	4
1790	1	0.142857142857143	0	4
1811	1	0.142857142857143	0	4
1852	1	0.142857142857143	0	4
1865	1	0.142857142857143	0	4
1879	1	0.142857142857143	0	4
1889	1	0.142857142857143	0	4
1941	1	0.142857142857143	0	4
1978	2	1	0	2
2017	1	0.142857142857143	0	4
2170	1	0.142857142857143	0	4
2233	1	0.5	0	4
2247	1	0.142857142857143	0	4
2295	1	0.5	0	4
2309	2	1	0	2
2312	1	0.5	0	4
2328	1	0.142857142857143	0	4
2339	4	0.571428571428571	0	2
2345	1	0.142857142857143	0	4

2356	1	0.142857142857143	0	4	
2357	1	0.5	0	4	
2403	1	0.5	0	4	
2408	1	0.142857142857143	0	4	
2420	1	0.142857142857143	0	4	<<<
2429	1	0.142857142857143	0	4	
2436	1	0.142857142857143	0	4	
2451	1	0.5	0	4	
2457	1	0.5	0	4	
2461	1	0.5	0	4	
2470	1	0.5	0	4	
2481	1	0.142857142857143	0	4	
2493	1	0.5	0	4	
2498	2	1	0	2	
2499	1	0.5	0	4	
2546	1	0.142857142857143	0	4	
2587	1	0.5	0	4	
2614	1	0.142857142857143	0	4	
2667	1	0.142857142857143	0	4	
2690	1	0.5	0	4	
2695	1	0.5	0	4	
2766	1	0.142857142857143	0	4	
2881	1	0.142857142857143	0	4	
2887	1	0.142857142857143	0	4	
2910	1	0.142857142857143	0	4	
2911	1	0.142857142857143	0	4	
2915	1	0.142857142857143	0	4	
2930	1	0.142857142857143	0	4	
2932	1	0.142857142857143	0	4	
2937	1	0.142857142857143	0	4	
2955	1	0.5	0	4	
2959	1	0.5	0	4	
2966	1	0.5	0	4	
2972	1	0.5	0	4	
2994	2	0.285714285714286	0	2	
2997	1	0.142857142857143	0	4	
3003	1	0.142857142857143	0	4	
3007	1	0.142857142857143	0	4	
3022	1	0.142857142857143	0	4	
3056	1	0.166666666666667	0	4	
3098	1	1	1	4	
3109	1	0.2	0	4	
3111	1	0.2	0	4	
3118	1	0.2	0	4	
3121	1	1	1	4	
3135	1	0.2	0	4	
3142	1	1	1	4	
3145	1	1	1	4	
3160	1	0.2	0	4	
3165	5	1	0	2	
3175	1	1	1	4	
3180	2	0.4	0	2	
3181	2	0.4	0	2	
3199	1	1	1	4	
3211	1	1	1	4	
3220	1	1	1	4	
3233	1	1	1	4	
3239	1	1	1	4	

3243	1	1	1	4
3255	1	1	1	4
3284	1	1	1	4
3296	1	1	1	4
3375	1	0.2	0	4
3376	1	0.2	0	4
3390	1	0.2	0	4
3404	1	0.5	0	4
3408	1	0.5	0	4
3410	1	0.166666666666667	0	4
3418	2	0.333333333333333	0	2
3434	1	0.166666666666667	0	4
3437	1	0.166666666666667	0	4
3438	1	0.166666666666667	0	4
3458	1	0.166666666666667	0	4
3471	1	0.5	0	4
3477	1	0.5	0	4
3478	1	0.5	0	4
3480	1	0.5	0	4
3496	2	1	0	2
3498	1	0.5	0	4
3502	1	0.5	0	4
3503	2	1	0	2
3505	2	1	0	2
3518	1	0.5	0	4
3537	1	0.5	0	4
3539	1	0.5	0	4
3544	4	1.64285714285714	0	0
3546	1	0.5	0	4
3549	1	0.5	0	4
3555	1	0.5	0	4
3557	1	0.5	0	4
3559	1	0.5	0	4
3562	1	0.5	0	4
3576	1	0.5	0	4
3577	1	0.5	0	4
3589	1	0.5	0	4
3638	1	0.5	0	4
3660	1	0.5	0	4
3665	1	0.5	0	4
3685	1	0.5	0	4

ptc-miR169aa

---

category=0, cleavage_site=1450
query=ptc-miR169aa, target=Potri.001G257600.1,
score=3, range=1439-1459, strand=1

```
target  5' GCGGCAAAUCAuUCUUGGCUU 3'
             ::.::: :::::::::::.
query   3' GGCUGUUCAGUAAGAACCGAG 5'
```

>Potri.001G257600.1
#size=2034

119	1	0.5	0	4
136	1	0.5	0	4
493	1	0.5	0	4
498	1	0.5	0	4
558	1	0.5	0	4
573	2	1	0	2

634	1	0.125	0	4	
1074	1	0.1	0	4	
1450	3	1.5	0	0	<<<
1491	1	0.5	0	4	

---

category=4, cleavage_site=1214
query=ptc-miR169aa, target=Potri.001G266000.1,
score=3, range=1203-1223, strand=1

```
target  5' CAGGCAAUUCAuUCUUGGCUU 3'
           : :.::: :::::::::::.
query   3' GGCUGUUCAGUAAGAACCGAG 5'
```

>Potri.001G266000.1
#size=1681

197	1	0.1	0	4	
244	1	0.2	0	4	
347	1	0.2	0	4	
385	1	0.2	0	4	
401	1	0.2	0	4	
540	1	0.1	0	4	
580	1	0.2	0	4	
686	1	0.1	0	4	
724	1	0.1	0	4	
727	1	0.1	0	4	
730	1	0.1	0	4	
781	2	0.2	0	2	
808	1	0.2	0	4	
978	1	0.2	0	4	
1214	1	0.2	0	4	<<<
1280	1	0.1	0	4	
1339	6	1.2	0	0	
1370	1	0.2	0	4	

---

category=2, cleavage_site=1444
query=ptc-miR169aa, target=Potri.009G052900.1,
score=3, range=1433-1453, strand=1

```
target  5' GCGGCAAAUCAuUCUUGGCUU 3'
            ::.::: :::::::::::.
query   3' GGCUGUUCAGUAAGAACCGAG 5'
```

>Potri.009G052900.1
#size=1848

130	1	0.166666666666667	0	4	
317	1	0.142857142857143	0	4	
598	1	0.125	0	4	
622	1	0.125	0	4	
669	1	0.142857142857143	0	4	
807	1	0.125	0	4	
978	1	0.125	0	4	
1051	1	0.125	0	4	
1057	1	0.125	0	4	
1062	1	0.1	0	4	
1310	1	0.125	0	4	
1444	8	1	0	2	<<<
1506	2	0.25	0	2	
1532	1	0.125	0	4	
1585	2	0.25	0	2	
1586	12	1.5	0	0	
1587	4	0.5	0	2	

1588	4	0.5	0	2
1589	1	0.125	0	4
1595	1	0.125	0	4
1599	1	0.125	0	4
1603	1	0.125	0	4
1608	1	0.125	0	4
1615	1	0.125	0	4
1650	1	0.125	0	4
1668	1	0.125	0	4
1669	2	0.25	0	2
1696	1	0.125	0	4

---

category=0, cleavage_site=1262
query=ptc-miR169aa, target=Potri.009G060600.1,
score=3, range=1251-1271, strand=1

```
target  5' CAGGCAAUUCAuUCUUGGCUU 3'
             : :.::: ::::::::::::.
query   3' GGCUGUUCAGUAAGAACCGAG 5'
```

>Potri.009G060600.1
#size=1930

92	1	0.2	0	4	
122	1	0.2	0	4	
144	1	0.2	0	4	
226	1	0.1	0	4	
239	1	0.1	0	4	
241	1	0.1	0	4	
275	1	0.166666666666667	0	4	
280	2	0.333333333333333	0	2	
292	1	0.166666666666667	0	4	
383	1	0.166666666666667	0	4	
435	1	0.166666666666667	0	4	
449	1	0.166666666666667	0	4	
454	1	0.166666666666667	0	4	
477	1	0.166666666666667	0	4	
508	1	0.166666666666667	0	4	
585	1	0.1	0	4	
620	1	0.166666666666667	0	4	
636	1	0.166666666666667	0	4	
704	1	0.166666666666667	0	4	
731	1	0.1	0	4	
769	1	0.1	0	4	
772	1	0.1	0	4	
775	1	0.1	0	4	
801	1	0.166666666666667	0	4	
826	1	0.1	0	4	
877	1	0.1	0	4	
1186	1	0.166666666666667	0	4	
1205	1	0.166666666666667	0	4	
1215	1	0.166666666666667	0	4	
1222	1	0.166666666666667	0	4	
1262	5	0.833333333333333	0	0	<<<
1263	1	0.166666666666667	0	4	
1274	1	0.166666666666667	0	4	
1296	1	0.166666666666667	0	4	
1314	1	0.166666666666667	0	4	
1317	1	0.166666666666667	0	4	
1322	1	0.1	0	4	
1342	3	0.5	0	2	

1343	2	0.333333333333333	0	2
1348	1	0.166666666666667	0	4
1353	1	0.166666666666667	0	4
1361	2	0.333333333333333	0	2
1376	1	0.166666666666667	0	4
1379	1	0.166666666666667	0	4
1547	1	0.166666666666667	0	4

ptc-miR169a-c

category=0, cleavage_site=1450
query=ptc-miR169a-c, target=Potri.001G257600.1,
score=4, range=1439-1459, strand=1

```
target  5' GCGGCAAAUCAuUCUUGGCUU 3'
             :::::: ::::.:::::::
query   3' AGCCGUUCAGUAGGAACCGAC 5'
```

>Potri.001G257600.1
#size=2034

119	1	0.5	0	4	
136	1	0.5	0	4	
493	1	0.5	0	4	
498	1	0.5	0	4	
558	1	0.5	0	4	
573	2	1	0	2	
634	1	0.125	0	4	
1074	1	0.1	0	4	
1450	3	1.5	0	0	<<<
1491	1	0.5	0	4	

category=4, cleavage_site=1214
query=ptc-miR169a-c, target=Potri.001G266000.1,
score=4, range=1202-1223, strand=1

```
target  5' UCAGGCAAUUCAuUCUUGGCUU 3'
            :: ::::: ::::.:::::::
query   3' AG-CCGUUCAGUAGGAACCGAC 5'
```

>Potri.001G266000.1
#size=1681

197	1	0.1	0	4	
244	1	0.2	0	4	
347	1	0.2	0	4	
385	1	0.2	0	4	
401	1	0.2	0	4	
540	1	0.1	0	4	
580	1	0.2	0	4	
686	1	0.1	0	4	
724	1	0.1	0	4	
727	1	0.1	0	4	
730	1	0.1	0	4	
781	2	0.2	0	2	
808	1	0.2	0	4	
978	1	0.2	0	4	
1214	1	0.2	0	4	<<<
1280	1	0.1	0	4	
1339	6	1.2	0	0	
1370	1	0.2	0	4	

category=0, cleavage_site=1390
query=ptc-miR169a-c, target=Potri.006G145100.1,
score=4, range=1379-1399, strand=1

```
target  5' CAGGCAAUUCAuCCUUGGCUU 3'
              ::::: :::::::::::
query   3' AGCCGUUCAGUAGGAACCGAC 5'
```

>Potri.006G145100.1
#size=1643

85	1	0.166666666666667	0	4	
187	1	0.5	0	4	
373	1	0.25	0	4	
410	1	0.25	0	4	
454	1	0.25	0	4	
509	1	0.166666666666667	0	4	
536	1	0.25	0	4	
582	1	0.25	0	4	
617	1	0.2	0	4	
619	1	0.2	0	4	
621	1	0.2	0	4	
631	1	0.2	0	4	
636	1	0.2	0	4	
674	1	0.2	0	4	
691	1	0.2	0	4	
734	1	0.2	0	4	
795	1	0.166666666666667	0	4	
816	1	0.111111111111111	0	4	
875	2	0.333333333333333	0	2	
890	1	0.166666666666667	0	4	
914	1	0.111111111111111	0	4	
917	1	0.166666666666667	0	4	
931	1	0.166666666666667	0	4	
939	1	0.111111111111111	0	4	
943	1	0.111111111111111	0	4	
948	1	0.111111111111111	0	4	
1002	1	0.111111111111111	0	4	
1064	1	0.166666666666667	0	4	
1065	2	0.333333333333333	0	2	
1068	2	0.333333333333333	0	2	
1081	1	0.166666666666667	0	4	
1083	1	0.166666666666667	0	4	
1144	1	0.166666666666667	0	4	
1156	1	0.166666666666667	0	4	
1166	1	0.166666666666667	0	4	
1172	1	0.166666666666667	0	4	
1179	1	0.166666666666667	0	4	
1184	1	0.166666666666667	0	4	
1204	1	0.166666666666667	0	4	
1205	1	0.166666666666667	0	4	
1231	1	0.166666666666667	0	4	
1236	1	0.166666666666667	0	4	
1239	2	0.333333333333333	0	2	
1246	1	0.166666666666667	0	4	
1390	19	3.16666666666667	0	0	<<<
1391	1	0.166666666666667	0	4	
1392	1	0.166666666666667	0	4	
1419	2	0.333333333333333	0	2	
1421	2	0.333333333333333	0	2	

1422	4	0.666666666666667	0	2
1423	2	0.222222222222222	0	2
1425	1	0.166666666666667	0	4
1427	1	0.166666666666667	0	4
1429	3	0.5	0	2
1430	6	1	0	2
1431	1	0.166666666666667	0	4
1433	1	0.111111111111111	0	4
1434	5	0.833333333333333	0	2
1452	1	0.166666666666667	0	4
1453	1	0.166666666666667	0	4
1463	1	0.166666666666667	0	4
1469	1	0.166666666666667	0	4
1538	1	0.166666666666667	0	4
1541	1	0.166666666666667	0	4
1564	2	0.222222222222222	0	2
1604	1	1	1	4

---

category=2, cleavage_site=1444
query=ptc-miR169a-c, target=Potri.009G052900.1,
score=4, range=1433-1453, strand=1

```
target  5' GCGGCAAAUCAuUCUUGGCUU 3'
           :::::: ::::.:::::::
query   3' AGCCGUUCAGUAGGAACCGAC 5'
```

>Potri.009G052900.1
#size=1848

130	1	0.166666666666667	0	4	
317	1	0.142857142857143	0	4	
598	1	0.125	0	4	
622	1	0.125	0	4	
669	1	0.142857142857143	0	4	
807	1	0.125	0	4	
978	1	0.125	0	4	
1051	1	0.125	0	4	
1057	1	0.125	0	4	
1062	1	0.1	0	4	
1310	1	0.125	0	4	
1444	8	1	0	2	<<<
1506	2	0.25	0	2	
1532	1	0.125	0	4	
1585	2	0.25	0	2	
1586	12	1.5	0	0	
1587	4	0.5	0	2	
1588	4	0.5	0	2	
1589	1	0.125	0	4	
1595	1	0.125	0	4	
1599	1	0.125	0	4	
1603	1	0.125	0	4	
1608	1	0.125	0	4	
1615	1	0.125	0	4	
1650	1	0.125	0	4	
1668	1	0.125	0	4	
1669	2	0.25	0	2	
1696	1	0.125	0	4	

category=0, cleavage_site=1262
query=ptc-miR169a-c, target=Potri.009G060600.1,
score=4, range=1250-1271, strand=1

```
target  5' UCAGGCAAUUCAuUCUUGGCUU 3'
            :: ::::: ::::.:::::::: 
query   3' AG-CCGUUCAGUAGGAACCGAC 5'
```

>Potri.009G060600.1
#size=1930

92	1	0.2	0	4	
122	1	0.2	0	4	
144	1	0.2	0	4	
226	1	0.1	0	4	
239	1	0.1	0	4	
241	1	0.1	0	4	
275	1	0.166666666666667	0	4	
280	2	0.333333333333333	0	2	
292	1	0.166666666666667	0	4	
383	1	0.166666666666667	0	4	
435	1	0.166666666666667	0	4	
449	1	0.166666666666667	0	4	
454	1	0.166666666666667	0	4	
477	1	0.166666666666667	0	4	
508	1	0.166666666666667	0	4	
585	1	0.1	0	4	
620	1	0.166666666666667	0	4	
636	1	0.166666666666667	0	4	
704	1	0.166666666666667	0	4	
731	1	0.1	0	4	
769	1	0.1	0	4	
772	1	0.1	0	4	
775	1	0.1	0	4	
801	1	0.166666666666667	0	4	
826	1	0.1	0	4	
877	1	0.1	0	4	
1186	1	0.166666666666667	0	4	
1205	1	0.166666666666667	0	4	
1215	1	0.166666666666667	0	4	
1222	1	0.166666666666667	0	4	
1262	5	0.833333333333333	0	0	<<<
1263	1	0.166666666666667	0	4	
1274	1	0.166666666666667	0	4	
1296	1	0.166666666666667	0	4	
1314	1	0.166666666666667	0	4	
1317	1	0.166666666666667	0	4	
1322	1	0.1	0	4	
1342	3	0.5	0	2	
1343	2	0.333333333333333	0	2	
1348	1	0.166666666666667	0	4	
1353	1	0.166666666666667	0	4	
1361	2	0.333333333333333	0	2	
1376	1	0.166666666666667	0	4	
1379	1	0.166666666666667	0	4	
1547	1	0.166666666666667	0	4	

category=0, cleavage_site=1278
query=ptc-miR169a-c, target=Potri.018G064700.1,
score=3, range=1266-1287, strand=1

```
target  5' UCAGGCAAUUCAuCCUUGGCUU 3'
           :: ::::: ::::::::::::
query   3' AG-CCGUUCAGUAGGAACCGAC 5'
```

>Potri.018G064700.1
#size=1544

25	1	0.166666666666667	0	4	
407	1	0.5	0	4	
413	1	0.166666666666667	0	4	
538	1	0.333333333333333	0	4	
674	1	0.333333333333333	0	4	
714	1	0.111111111111111	0	4	
796	1	0.333333333333333	0	4	
812	1	0.111111111111111	0	4	
837	1	0.111111111111111	0	4	
841	1	0.111111111111111	0	4	
846	1	0.111111111111111	0	4	
900	1	0.111111111111111	0	4	
974	1	0.333333333333333	0	4	
1003	1	0.333333333333333	0	4	
1152	1	0.333333333333333	0	4	
1166	1	0.333333333333333	0	4	
1171	1	0.333333333333333	0	4	
1213	2	0.666666666666667	0	2	
1216	1	0.333333333333333	0	4	
1243	1	0.333333333333333	0	4	
1267	1	0.333333333333333	0	4	
1273	1	0.333333333333333	0	4	
1278	25	8.33333333333333	0	0	<<<
1298	1	0.333333333333333	0	4	
1301	3	1	0	2	
1308	1	0.333333333333333	0	4	
1310	1	0.333333333333333	0	4	
1311	2	0.666666666666667	0	2	
1312	7	1.88888888888889	0	2	
1313	2	0.666666666666667	0	2	
1314	1	0.333333333333333	0	4	
1316	2	0.666666666666667	0	2	
1319	5	1.66666666666667	0	2	
1320	2	0.666666666666667	0	2	
1322	1	0.111111111111111	0	4	
1327	1	0.333333333333333	0	4	
1423	1	0.333333333333333	0	4	
1460	2	0.222222222222222	0	3	
1465	1	0.333333333333333	0	4	
1468	1	0.333333333333333	0	4	

ptc-miR169d-h

category=0, cleavage_site=1450
query=ptc-miR169d-h, target=Potri.001G257600.1,
score=4, range=1439-1459, strand=1

```
target  5' GCGGCAAAUCAuUCUUGGCUU 3'
            :::::: ::::.:::::::
query   3' GGCCGUUCAGUAGGAACCGAC 5'
```

>Potri.001G257600.1

#size=2034

119	1	0.5	0	4	
136	1	0.5	0	4	
493	1	0.5	0	4	
498	1	0.5	0	4	
558	1	0.5	0	4	
573	2	1	0	2	
634	1	0.125	0	4	
1074	1	0.1	0	4	
1450	3	1.5	0	0	<<<
1491	1	0.5	0	4	

---

category=4, cleavage_site=1214
query=ptc-miR169d-h, target=Potri.001G266000.1,
score=4, range=1203-1223, strand=1

```
target  5' CAGGCAAUUCAuUCUUGGCUU 3'
             : ::::: ::::.:::::::
query   3' GGCCGUUCAGUAGGAACCGAC 5'
```

>Potri.001G266000.1
#size=1681

197	1	0.1	0	4	
244	1	0.2	0	4	
347	1	0.2	0	4	
385	1	0.2	0	4	
401	1	0.2	0	4	
540	1	0.1	0	4	
580	1	0.2	0	4	
686	1	0.1	0	4	
724	1	0.1	0	4	
727	1	0.1	0	4	
730	1	0.1	0	4	
781	2	0.2	0	2	
808	1	0.2	0	4	
978	1	0.2	0	4	
1214	1	0.2	0	4	<<<
1280	1	0.1	0	4	
1339	6	1.2	0	0	
1370	1	0.2	0	4	

---

category=0, cleavage_site=1390
query=ptc-miR169d-h, target=Potri.006G145100.1,
score=3, range=1379-1399, strand=1

```
target  5' CAGGCAAUUCAuCCUUGGCUU 3'
             : ::::: ::::::::::::
query   3' GGCCGUUCAGUAGGAACCGAC 5'
```

>Potri.006G145100.1
#size=1643

85	1	0.166666666666667	0	4
187	1	0.5	0	4
373	1	0.25	0	4
410	1	0.25	0	4
454	1	0.25	0	4
509	1	0.166666666666667	0	4
536	1	0.25	0	4
582	1	0.25	0	4
617	1	0.2	0	4
619	1	0.2	0	4
621	1	0.2	0	4

631	1	0.2	0	4	
636	1	0.2	0	4	
674	1	0.2	0	4	
691	1	0.2	0	4	
734	1	0.2	0	4	
795	1	0.166666666666667	0	4	
816	1	0.111111111111111	0	4	
875	2	0.333333333333333	0	2	
890	1	0.166666666666667	0	4	
914	1	0.111111111111111	0	4	
917	1	0.166666666666667	0	4	
931	1	0.166666666666667	0	4	
939	1	0.111111111111111	0	4	
943	1	0.111111111111111	0	4	
948	1	0.111111111111111	0	4	
1002	1	0.111111111111111	0	4	
1064	1	0.166666666666667	0	4	
1065	2	0.333333333333333	0	2	
1068	2	0.333333333333333	0	2	
1081	1	0.166666666666667	0	4	
1083	1	0.166666666666667	0	4	
1144	1	0.166666666666667	0	4	
1156	1	0.166666666666667	0	4	
1166	1	0.166666666666667	0	4	
1172	1	0.166666666666667	0	4	
1179	1	0.166666666666667	0	4	
1184	1	0.166666666666667	0	4	
1204	1	0.166666666666667	0	4	
1205	1	0.166666666666667	0	4	
1231	1	0.166666666666667	0	4	
1236	1	0.166666666666667	0	4	
1239	2	0.333333333333333	0	2	
1246	1	0.166666666666667	0	4	
1390	19	3.16666666666667	0	0	<<<
1391	1	0.166666666666667	0	4	
1392	1	0.166666666666667	0	4	
1419	2	0.333333333333333	0	2	
1421	2	0.333333333333333	0	2	
1422	4	0.666666666666667	0	2	
1423	2	0.222222222222222	0	2	
1425	1	0.166666666666667	0	4	
1427	1	0.166666666666667	0	4	
1429	3	0.5	0	2	
1430	6	1	0	2	
1431	1	0.166666666666667	0	4	
1433	1	0.111111111111111	0	4	
1434	5	0.833333333333333	0	2	
1452	1	0.166666666666667	0	4	
1453	1	0.166666666666667	0	4	
1463	1	0.166666666666667	0	4	
1469	1	0.166666666666667	0	4	
1538	1	0.166666666666667	0	4	
1541	1	0.166666666666667	0	4	
1564	2	0.222222222222222	0	2	
1604	1	1	1	4	

category=2, cleavage_site=1444
query=ptc-miR169d-h, target=Potri.009G052900.1,
score=4, range=1433-1453, strand=1

```
target  5' GCGGCAAAUCAuUCUUGGCUU 3'
             :::::: ::::.::::::::
query   3' GGCCGUUCAGUAGGAACCGAC 5'
```

>Potri.009G052900.1
#size=1848

130	1	0.166666666666667	0	4	
317	1	0.142857142857143	0	4	
598	1	0.125	0	4	
622	1	0.125	0	4	
669	1	0.142857142857143	0	4	
807	1	0.125	0	4	
978	1	0.125	0	4	
1051	1	0.125	0	4	
1057	1	0.125	0	4	
1062	1	0.1	0	4	
1310	1	0.125	0	4	
1444	8	1	0	2	<<<
1506	2	0.25	0	2	
1532	1	0.125	0	4	
1585	2	0.25	0	2	
1586	12	1.5	0	0	
1587	4	0.5	0	2	
1588	4	0.5	0	2	
1589	1	0.125	0	4	
1595	1	0.125	0	4	
1599	1	0.125	0	4	
1603	1	0.125	0	4	
1608	1	0.125	0	4	
1615	1	0.125	0	4	
1650	1	0.125	0	4	
1668	1	0.125	0	4	
1669	2	0.25	0	2	
1696	1	0.125	0	4	

category=0, cleavage_site=1262
query=ptc-miR169d-h, target=Potri.009G060600.1,
score=4, range=1251-1271, strand=1

```
target  5' CAGGCAAUUCAuUCUUGGCUU 3'
            : ::::: ::::.::::::::
query   3' GGCCGUUCAGUAGGAACCGAC 5'
```

>Potri.009G060600.1
#size=1930

92	1	0.2	0	4
122	1	0.2	0	4
144	1	0.2	0	4
226	1	0.1	0	4
239	1	0.1	0	4
241	1	0.1	0	4
275	1	0.166666666666667	0	4
280	2	0.333333333333333	0	2
292	1	0.166666666666667	0	4
383	1	0.166666666666667	0	4
435	1	0.166666666666667	0	4
449	1	0.166666666666667	0	4

454	1	0.166666666666667	0	4	
477	1	0.166666666666667	0	4	
508	1	0.166666666666667	0	4	
585	1	0.1	0	4	
620	1	0.166666666666667	0	4	
636	1	0.166666666666667	0	4	
704	1	0.166666666666667	0	4	
731	1	0.1	0	4	
769	1	0.1	0	4	
772	1	0.1	0	4	
775	1	0.1	0	4	
801	1	0.166666666666667	0	4	
826	1	0.1	0	4	
877	1	0.1	0	4	
1186	1	0.166666666666667	0	4	
1205	1	0.166666666666667	0	4	
1215	1	0.166666666666667	0	4	
1222	1	0.166666666666667	0	4	
1262	5	0.833333333333333	0	0	<<<
1263	1	0.166666666666667	0	4	
1274	1	0.166666666666667	0	4	
1296	1	0.166666666666667	0	4	
1314	1	0.166666666666667	0	4	
1317	1	0.166666666666667	0	4	
1322	1	0.1	0	4	
1342	3	0.5	0	2	
1343	2	0.333333333333333	0	2	
1348	1	0.166666666666667	0	4	
1353	1	0.166666666666667	0	4	
1361	2	0.333333333333333	0	2	
1376	1	0.166666666666667	0	4	
1379	1	0.166666666666667	0	4	
1547	1	0.166666666666667	0	4	

category=0, cleavage_site=1278
query=ptc-miR169d-h, target=Potri.018G064700.1,
score=3, range=1267-1287, strand=1

```
target  5' CAGGCAAUUCAuCCUUGGCUU 3'
             : ::::: :::::::::::
query   3' GGCCGUUCAGUAGGAACCGAC 5'
```

>Potri.018G064700.1
#size=1544

25	1	0.166666666666667	0	4
407	1	0.5	0	4
413	1	0.166666666666667	0	4
538	1	0.333333333333333	0	4
674	1	0.333333333333333	0	4
714	1	0.111111111111111	0	4
796	1	0.333333333333333	0	4
812	1	0.111111111111111	0	4
837	1	0.111111111111111	0	4
841	1	0.111111111111111	0	4
846	1	0.111111111111111	0	4
900	1	0.111111111111111	0	4
974	1	0.333333333333333	0	4
1003	1	0.333333333333333	0	4
1152	1	0.333333333333333	0	4
1166	1	0.333333333333333	0	4

1171	1	0.333333333333333	0	4	
1213	2	0.666666666666667	0	2	
1216	1	0.333333333333333	0	4	
1243	1	0.333333333333333	0	4	
1267	1	0.333333333333333	0	4	
1273	1	0.333333333333333	0	4	
1278	25	8.33333333333333	0	0	<<<
1298	1	0.333333333333333	0	4	
1301	3	1	0	2	
1308	1	0.333333333333333	0	4	
1310	1	0.333333333333333	0	4	
1311	2	0.666666666666667	0	2	
1312	7	1.88888888888889	0	2	
1313	2	0.666666666666667	0	2	
1314	1	0.333333333333333	0	4	
1316	2	0.666666666666667	0	2	
1319	5	1.66666666666667	0	2	
1320	2	0.666666666666667	0	2	
1322	1	0.111111111111111	0	4	
1327	1	0.333333333333333	0	4	
1423	1	0.333333333333333	0	4	
1460	2	0.222222222222222	0	3	
1465	1	0.333333333333333	0	4	
1468	1	0.333333333333333	0	4	

ptc-miR169q

category=0, cleavage_site=1390
query=ptc-miR169q, target=Potri.006G145100.1,
score=4, range=1379-1399, strand=1

```
target  5' CAGGCAAUUCAuCCUUGGCUU 3'
           ::::::: :: :::::::::
query   3' GUCCGUUCAGCAGGAACCGAU 5'
```

>Potri.006G145100.1
#size=1643

85	1	0.166666666666667	0	4
187	1	0.5	0	4
373	1	0.25	0	4
410	1	0.25	0	4
454	1	0.25	0	4
509	1	0.166666666666667	0	4
536	1	0.25	0	4
582	1	0.25	0	4
617	1	0.2	0	4
619	1	0.2	0	4
621	1	0.2	0	4
631	1	0.2	0	4
636	1	0.2	0	4
674	1	0.2	0	4
691	1	0.2	0	4
734	1	0.2	0	4
795	1	0.166666666666667	0	4
816	1	0.111111111111111	0	4
875	2	0.333333333333333	0	2
890	1	0.166666666666667	0	4
914	1	0.111111111111111	0	4
917	1	0.166666666666667	0	4
931	1	0.166666666666667	0	4

939 1 0.111111111111111 0 4
943 1 0.111111111111111 0 4
948 1 0.111111111111111 0 4
1002 1 0.111111111111111 0 4
1064 1 0.166666666666667 0 4
1065 2 0.333333333333333 0 2
1068 2 0.333333333333333 0 2
1081 1 0.166666666666667 0 4
1083 1 0.166666666666667 0 4
1144 1 0.166666666666667 0 4
1156 1 0.166666666666667 0 4
1166 1 0.166666666666667 0 4
1172 1 0.166666666666667 0 4
1179 1 0.166666666666667 0 4
1184 1 0.166666666666667 0 4
1204 1 0.166666666666667 0 4
1205 1 0.166666666666667 0 4
1231 1 0.166666666666667 0 4
1236 1 0.166666666666667 0 4
1239 2 0.333333333333333 0 2
1246 1 0.166666666666667 0 4
1390 19 3.16666666666667 0 0 <<<
1391 1 0.166666666666667 0 4
1392 1 0.166666666666667 0 4
1419 2 0.333333333333333 0 2
1421 2 0.333333333333333 0 2
1422 4 0.666666666666667 0 2
1423 2 0.222222222222222 0 2
1425 1 0.166666666666667 0 4
1427 1 0.166666666666667 0 4
1429 3 0.5 0 2
1430 6 1 0 2
1431 1 0.166666666666667 0 4
1433 1 0.111111111111111 0 4
1434 5 0.833333333333333 0 2
1452 1 0.166666666666667 0 4
1453 1 0.166666666666667 0 4
1463 1 0.166666666666667 0 4
1469 1 0.166666666666667 0 4
1538 1 0.166666666666667 0 4
1541 1 0.166666666666667 0 4
1564 2 0.222222222222222 0 2
1604 1 1 1 4

---

category=0, cleavage_site=1278
query=ptc-miR169q, target=Potri.018G064700.1,
score=4, range=1267-1287, strand=1

```
target  5' CAGGCAAUUCAuCCUUGGCUU 3'
           ::::::: :: :::::::::
query   3' GUCCGUUCAGCAGGAACCGAU 5'
```

>Potri.018G064700.1
#size=1544
25 1 0.166666666666667 0 4
407 1 0.5 0 4
413 1 0.166666666666667 0 4
538 1 0.333333333333333 0 4
674 1 0.333333333333333 0 4
714 1 0.111111111111111 0 4

796	1	0.333333333333333	0	4	
812	1	0.111111111111111	0	4	
837	1	0.111111111111111	0	4	
841	1	0.111111111111111	0	4	
846	1	0.111111111111111	0	4	
900	1	0.111111111111111	0	4	
974	1	0.333333333333333	0	4	
1003	1	0.333333333333333	0	4	
1152	1	0.333333333333333	0	4	
1166	1	0.333333333333333	0	4	
1171	1	0.333333333333333	0	4	
1213	2	0.666666666666667	0	2	
1216	1	0.333333333333333	0	4	
1243	1	0.333333333333333	0	4	
1267	1	0.333333333333333	0	4	
1273	1	0.333333333333333	0	4	
1278	25	8.33333333333333	0	0	<<<
1298	1	0.333333333333333	0	4	
1301	3	1	0	2	
1308	1	0.333333333333333	0	4	
1310	1	0.333333333333333	0	4	
1311	2	0.666666666666667	0	2	
1312	7	1.88888888888889	0	2	
1313	2	0.666666666666667	0	2	
1314	1	0.333333333333333	0	4	
1316	2	0.666666666666667	0	2	
1319	5	1.66666666666667	0	2	
1320	2	0.666666666666667	0	2	
1322	1	0.111111111111111	0	4	
1327	1	0.333333333333333	0	4	
1423	1	0.333333333333333	0	4	
1460	2	0.222222222222222	0	3	
1465	1	0.333333333333333	0	4	
1468	1	0.333333333333333	0	4	

ptc-miR169t

category=0, cleavage_site=1450
query=ptc-miR169t, target=Potri.001G257600.1,
score=2.5, range=1439-1459, strand=1

```
target  5' GCGGCAAAUCAuUCUUGGCUU 3'
           :::::: :::::::::::.
query   3' GGCCGUUCAGUAAGAACCGAG 5'
```

>Potri.001G257600.1
#size=2034

119	1	0.5	0	4	
136	1	0.5	0	4	
493	1	0.5	0	4	
498	1	0.5	0	4	
558	1	0.5	0	4	
573	2	1	0	2	
634	1	0.125	0	4	
1074	1	0.1	0	4	
1450	3	1.5	0	0	<<<
1491	1	0.5	0	4	

category=4, cleavage_site=1214
query=ptc-miR169t, target=Potri.001G266000.1,
score=2.5, range=1203-1223, strand=1

```
target  5' CAGGCAAUUCAuUCUUGGCUU 3'
           : ::::: ::::::::::::.
query   3' GGCCGUUCAGUAAGAACCGAG 5'
```

>Potri.001G266000.1
#size=1681

197	1	0.1	0	4	
244	1	0.2	0	4	
347	1	0.2	0	4	
385	1	0.2	0	4	
401	1	0.2	0	4	
540	1	0.1	0	4	
580	1	0.2	0	4	
686	1	0.1	0	4	
724	1	0.1	0	4	
727	1	0.1	0	4	
730	1	0.1	0	4	
781	2	0.2	0	2	
808	1	0.2	0	4	
978	1	0.2	0	4	
1214	1	0.2	0	4	<<<
1280	1	0.1	0	4	
1339	6	1.2	0	0	
1370	1	0.2	0	4	

category=2, cleavage_site=1444
query=ptc-miR169t, target=Potri.009G052900.1,
score=2.5, range=1433-1453, strand=1

```
target  5' GCGGCAAAUCAuUCUUGGCUU 3'
            :::::: ::::::::::::.
query   3' GGCCGUUCAGUAAGAACCGAG 5'
```

>Potri.009G052900.1
#size=1848

130	1	0.166666666666667	0	4	
317	1	0.142857142857143	0	4	
598	1	0.125	0	4	
622	1	0.125	0	4	
669	1	0.142857142857143	0	4	
807	1	0.125	0	4	
978	1	0.125	0	4	
1051	1	0.125	0	4	
1057	1	0.125	0	4	
1062	1	0.1	0	4	
1310	1	0.125	0	4	
1444	8	1	0	2	<<<
1506	2	0.25	0	2	
1532	1	0.125	0	4	
1585	2	0.25	0	2	
1586	12	1.5	0	0	
1587	4	0.5	0	2	
1588	4	0.5	0	2	
1589	1	0.125	0	4	
1595	1	0.125	0	4	
1599	1	0.125	0	4	
1603	1	0.125	0	4	
1608	1	0.125	0	4	

1615	1	0.125	0	4
1650	1	0.125	0	4
1668	1	0.125	0	4
1669	2	0.25	0	2
1696	1	0.125	0	4

category=0, cleavage_site=1262
query=ptc-miR169t, target=Potri.009G060600.1,
score=2.5, range=1251-1271, strand=1

```
target  5' CAGGCAAUUCAuUCUUGGCUU 3'
           : ::::: :::::::::::.
query   3' GGCCGUUCAGUAAGAACCGAG 5'
```

>Potri.009G060600.1
#size=1930

92	1	0.2	0	4	
122	1	0.2	0	4	
144	1	0.2	0	4	
226	1	0.1	0	4	
239	1	0.1	0	4	
241	1	0.1	0	4	
275	1	0.166666666666667	0	4	
280	2	0.333333333333333	0	2	
292	1	0.166666666666667	0	4	
383	1	0.166666666666667	0	4	
435	1	0.166666666666667	0	4	
449	1	0.166666666666667	0	4	
454	1	0.166666666666667	0	4	
477	1	0.166666666666667	0	4	
508	1	0.166666666666667	0	4	
585	1	0.1	0	4	
620	1	0.166666666666667	0	4	
636	1	0.166666666666667	0	4	
704	1	0.166666666666667	0	4	
731	1	0.1	0	4	
769	1	0.1	0	4	
772	1	0.1	0	4	
775	1	0.1	0	4	
801	1	0.166666666666667	0	4	
826	1	0.1	0	4	
877	1	0.1	0	4	
1186	1	0.166666666666667	0	4	
1205	1	0.166666666666667	0	4	
1215	1	0.166666666666667	0	4	
1222	1	0.166666666666667	0	4	
1262	5	0.833333333333333	0	0	<<<
1263	1	0.166666666666667	0	4	
1274	1	0.166666666666667	0	4	
1296	1	0.166666666666667	0	4	
1314	1	0.166666666666667	0	4	
1317	1	0.166666666666667	0	4	
1322	1	0.1	0	4	
1342	3	0.5	0	2	
1343	2	0.333333333333333	0	2	
1348	1	0.166666666666667	0	4	
1353	1	0.166666666666667	0	4	
1361	2	0.333333333333333	0	2	
1376	1	0.166666666666667	0	4	
1379	1	0.166666666666667	0	4	

1547	1	0.166666666666667	0	4	

ptc-miR171a,b

category=4, cleavage_site=1764
query=ptc-miR171a,b, target=Potri.001G122800.1,
score=3, range=1753-1773, strand=1

```
target  5' AGGGAUAUUGGcGCGGCUCAA 3'
             : ::::::::.::::::::
query   3' GCACUAUAACCGUGCCGAGUU 5'
```

>Potri.001G122800.1
#size=2999

543	1	0.111111111111111	0	4	
569	1	0.333333333333333	0	4	
651	1	0.333333333333333	0	4	
1169	1	0.333333333333333	0	4	
1250	1	0.333333333333333	0	4	
1264	1	0.333333333333333	0	4	
1504	1	0.333333333333333	0	4	
1602	1	0.25	0	4	
1604	1	0.333333333333333	0	4	
1646	1	0.1	0	4	
1750	1	0.1	0	4	
1764	1	0.333333333333333	0	4	<<<
1767	8	2.66666666666667	0	0	
2045	1	0.25	0	4	
2074	1	0.333333333333333	0	4	
2149	1	0.333333333333333	0	4	
2184	1	0.25	0	4	
2292	1	0.25	0	4	
2298	1	0.25	0	4	
2326	1	0.25	0	4	
2339	1	0.25	0	4	
2353	1	0.25	0	4	
2407	1	0.333333333333333	0	4	
2412	1	0.333333333333333	0	4	
2423	1	0.333333333333333	0	4	
2495	1	0.25	0	4	
2496	2	0.5	0	2	
2500	1	0.333333333333333	0	4	
2503	1	0.25	0	4	
2505	1	0.25	0	4	
2511	1	0.25	0	4	
2523	1	0.25	0	4	
2533	1	0.25	0	4	
2535	1	0.25	0	4	
2536	1	0.25	0	4	
2549	1	0.333333333333333	0	4	
2563	1	0.333333333333333	0	4	
2564	1	0.333333333333333	0	4	
2566	1	0.333333333333333	0	4	
2567	2	0.666666666666667	0	2	
2571	1	0.333333333333333	0	4	
2589	1	0.25	0	4	
2596	1	0.333333333333333	0	4	
2607	1	0.333333333333333	0	4	
2629	1	0.25	0	4	
2630	1	0.25	0	4	

2646	1	0.25	0	4
2650	2	0.5	0	2
2651	1	0.25	0	4
2652	1	0.25	0	4
2661	1	0.25	0	4
2676	1	0.25	0	4
2678	1	0.25	0	4
2699	1	0.333333333333333	0	4
2700	1	0.333333333333333	0	4
2744	1	0.25	0	4
2755	1	0.333333333333333	0	4
2766	1	0.333333333333333	0	4
2778	1	0.333333333333333	0	4
2781	1	0.5	0	4
2842	1	0.5	0	4
2896	1	0.5	0	4
2902	1	1	1	4

---

category=2, cleavage_site=1767
query=ptc-miR171a,b, target=Potri.002G144200.1,
score=3, range=1756-1776, strand=1

```
target  5' AGGGAUAUUGGcGCGGCUCAA 3'
            : ::::::::.::::::::
query   3' GCACUAUAACCGUGCCGAGUU 5'
```

>Potri.002G144200.1
#size=3548

43	2	0.666666666666667	0	2	
174	1	0.333333333333333	0	4	
216	1	0.333333333333333	0	4	
264	1	0.333333333333333	0	4	
565	1	0.2	0	4	
828	1	0.333333333333333	0	4	
1040	1	0.25	0	4	
1134	1	0.5	0	4	
1320	1	0.166666666666667	0	4	
1432	1	0.125	0	4	
1699	1	0.111111111111111	0	4	
1740	1	0.111111111111111	0	4	
1753	1	0.1	0	4	
1755	1	0.111111111111111	0	4	
1766	1	0.111111111111111	0	4	
1767	13	1.57142857142857	0	2	<<<
1770	21	2.58730158730159	0	0	
1771	1	0.111111111111111	0	4	
1773	1	0.25	0	4	
1776	1	0.111111111111111	0	4	
1778	1	0.25	0	4	
1930	1	0.142857142857143	0	4	
2014	1	0.25	0	4	
2060	1	0.125	0	4	
2088	1	0.111111111111111	0	4	
2099	1	0.125	0	4	
2128	1	0.333333333333333	0	4	
2275	1	0.333333333333333	0	4	
2291	1	0.333333333333333	0	4	
2556	1	0.333333333333333	0	4	
2557	1	0.333333333333333	0	4	
2675	1	0.333333333333333	0	4	

2697	1	0.333333333333333	0	4
2765	2	0.666666666666667	0	2
2768	1	0.333333333333333	0	4
2856	1	0.333333333333333	0	4
2857	1	0.333333333333333	0	4
2901	1	0.333333333333333	0	4
2915	1	0.333333333333333	0	4
2949	1	0.333333333333333	0	4
2963	1	0.333333333333333	0	4
2977	1	0.333333333333333	0	4
3147	1	0.333333333333333	0	4
3337	1	0.333333333333333	0	4
3391	1	0.333333333333333	0	4

---

category=2, cleavage_site=1926
query=ptc-miR171a,b, target=Potri.002G144700.1,
score=3, range=1915-1935, strand=1

```
target  5' AGGGAUAUUGGcGCGGCUCAA 3'
             : :::::::::.::::::::
query   3' GCACUAUAACCGUGCCGAGUU 5'
```

>Potri.002G144700.1
#size=3311

1011	1	0.166666666666667	0	4	
1316	1	0.25	0	4	
1468	1	0.166666666666667	0	4	
1485	1	0.166666666666667	0	4	
1597	1	0.125	0	4	
1807	1	0.166666666666667	0	4	
1858	1	0.111111111111111	0	4	
1868	1	0.166666666666667	0	4	
1899	1	0.111111111111111	0	4	
1912	1	0.1	0	4	
1914	1	0.111111111111111	0	4	
1925	1	0.111111111111111	0	4	
1926	13	1.57142857142857	0	2	<<<
1929	21	2.58730158730159	0	0	
1930	1	0.111111111111111	0	4	
1932	1	0.25	0	4	
1935	1	0.111111111111111	0	4	
1937	1	0.25	0	4	
2021	1	1	1	4	
2089	1	0.142857142857143	0	4	
2173	1	0.25	0	4	
2247	1	0.111111111111111	0	4	
2613	1	0.25	0	4	
2802	1	1	1	4	
2811	1	1	1	4	
2813	1	0.333333333333333	0	4	
2839	1	0.166666666666667	0	4	
2845	1	0.25	0	4	
2858	1	0.25	0	4	
2920	1	1	1	4	
2927	1	1	1	4	
2938	1	1	1	4	
2942	1	1	1	4	
3102	1	0.25	0	4	
3111	2	2	2	2	

category=2, cleavage_site=1469
query=ptc-miR171a,b, target=Potri.014G060200.1,
score=3, range=1458-1478, strand=1

```
target  5' AGGGAUAUUGGcGCGGCUCAA 3'
             : :::::::::.::::::::
query   3' GCACUAUAACCGUGCCGAGUU 5'
```

>Potri.014G060200.1
#size=2475

42	1	0.5	0	4	
70	1	0.5	0	4	
101	1	0.5	0	4	
105	1	0.5	0	4	
114	1	0.5	0	4	
128	1	0.25	0	4	
130	1	0.5	0	4	
162	1	0.5	0	4	
390	1	0.2	0	4	
424	1	0.2	0	4	
557	1	0.166666666666667	0	4	
662	1	0.5	0	4	
704	1	0.2	0	4	
705	1	0.2	0	4	
756	1	0.2	0	4	
795	1	0.5	0	4	
865	1	0.2	0	4	
993	1	0.166666666666667	0	4	
1035	1	0.5	0	4	
1043	1	0.5	0	4	
1049	1	0.5	0	4	
1122	1	0.125	0	4	
1128	1	0.25	0	4	
1318	1	0.5	0	4	
1350	1	0.166666666666667	0	4	
1401	1	0.111111111111111	0	4	
1411	1	0.166666666666667	0	4	
1442	1	0.111111111111111	0	4	
1455	1	0.1	0	4	
1457	1	0.111111111111111	0	4	
1467	2	1	0	2	
1468	1	0.111111111111111	0	4	
1469	9	1	0	2	<<<
1472	13	1.44444444444444	0	0	
1473	1	0.111111111111111	0	4	
1478	1	0.111111111111111	0	4	
1612	1	0.2	0	4	
1762	1	0.125	0	4	
1790	1	0.111111111111111	0	4	
1801	1	0.125	0	4	
1918	1	0.2	0	4	
2035	1	0.5	0	4	
2050	1	0.5	0	4	
2104	1	0.5	0	4	
2155	1	0.2	0	4	
2161	1	0.5	0	4	
2269	1	0.5	0	4	
2301	1	0.5	0	4	
2338	1	0.2	0	4	

2341	1	0.2	0	4	
2347	1	0.5	0	4	
2348	1	0.2	0	4	
2353	1	0.5	0	4	
2356	1	0.333333333333333	0	4	
2382	1	0.166666666666667	0	4	
2387	1	0.5	0	4	
2400	2	1	0	2	
2414	1	0.2	0	4	
2418	1	0.5	0	4	
2423	1	0.5	0	4	

---

category=2, cleavage_site=1499
query=ptc-miR171a,b, target=Potri.014G060500.1,
score=3, range=1488-1508, strand=1

```
target  5' AGGGAUAUUGGcGCGGCUCAA 3'
              : :::::::::.::::::::
query   3' GCACUAUAACCGUGCCGAGUU 5'
```

>Potri.014G060500.1
#size=2923

11	1	0.5	0	4	
63	1	0.5	0	4	
86	1	0.5	0	4	
122	1	0.5	0	4	
136	2	1	0	2	
142	1	0.25	0	4	
145	1	0.5	0	4	
149	1	0.5	0	4	
150	1	0.5	0	4	
162	1	0.5	0	4	
442	1	0.2	0	4	
575	1	0.166666666666667	0	4	
703	1	0.333333333333333	0	4	
722	1	0.2	0	4	
723	1	0.2	0	4	
774	1	0.2	0	4	
868	1	0.333333333333333	0	4	
883	1	0.2	0	4	
895	1	0.333333333333333	0	4	
907	1	0.333333333333333	0	4	
1026	1	0.166666666666667	0	4	
1043	1	0.166666666666667	0	4	
1155	1	0.125	0	4	
1161	1	0.25	0	4	
1380	1	0.166666666666667	0	4	
1431	1	0.111111111111111	0	4	
1441	1	0.166666666666667	0	4	
1472	1	0.111111111111111	0	4	
1485	1	0.1	0	4	
1487	1	0.111111111111111	0	4	
1498	1	0.111111111111111	0	4	
1499	13	1.57142857142857	0	2	<<<
1502	21	2.58730158730159	0	0	
1503	1	0.111111111111111	0	4	
1508	1	0.111111111111111	0	4	
1642	1	0.2	0	4	
1662	1	0.142857142857143	0	4	
1792	1	0.125	0	4	

1820	1	0.111111111111111	0	4
1831	1	0.125	0	4
1948	1	0.2	0	4
1978	1	0.333333333333333	0	4
1990	1	0.333333333333333	0	4
2057	2	0.666666666666667	0	2
2161	1	0.333333333333333	0	4
2167	1	0.333333333333333	0	4
2179	1	0.333333333333333	0	4
2185	1	0.2	0	4
2186	1	0.25	0	4
2266	1	0.333333333333333	0	4
2267	1	0.333333333333333	0	4
2281	1	0.333333333333333	0	4
2368	1	0.2	0	4
2371	1	0.2	0	4
2378	1	0.2	0	4
2406	1	0.333333333333333	0	4
2412	1	0.166666666666667	0	4
2418	1	0.25	0	4
2431	1	0.25	0	4
2444	1	0.2	0	4
2507	2	0.666666666666667	0	2
2513	1	0.333333333333333	0	4
2559	1	0.333333333333333	0	4
2569	1	0.333333333333333	0	4
2574	1	0.333333333333333	0	4
2587	1	0.333333333333333	0	4
2593	1	0.333333333333333	0	4
2600	1	0.333333333333333	0	4
2606	1	0.333333333333333	0	4
2607	1	0.333333333333333	0	4
2609	1	0.333333333333333	0	4
2611	1	0.333333333333333	0	4
2622	1	0.333333333333333	0	4
2634	1	0.333333333333333	0	4
2638	1	0.333333333333333	0	4
2642	1	0.333333333333333	0	4
2650	2	0.666666666666667	0	2
2654	1	0.333333333333333	0	4
2663	1	0.25	0	4
2672	1	0.333333333333333	0	4
2731	1	0.333333333333333	0	4

ptc-miR171c,d

category=0, cleavage_site=1767
query=ptc-miR171c,d, target=Potri.001G122800.1,
score=1, range=1756-1776, strand=1

```
target  5' GAUAUUGGCGCgGCUCAAUCA 3'
            :::::::::::::::::::: 
query   3' CUAUAACCGCGCCGAGUUAGA 5'
```

>Potri.001G122800.1
#size=2999

543	1	0.111111111111111	0	4
569	1	0.333333333333333	0	4
651	1	0.333333333333333	0	4
1169	1	0.333333333333333	0	4

1250	1	0.333333333333333	0	4	
1264	1	0.333333333333333	0	4	
1504	1	0.333333333333333	0	4	
1602	1	0.25	0	4	
1604	1	0.333333333333333	0	4	
1646	1	0.1	0	4	
1750	1	0.1	0	4	
1764	1	0.333333333333333	0	4	
1767	8	2.66666666666667	0	0	<<<
2045	1	0.25	0	4	
2074	1	0.333333333333333	0	4	
2149	1	0.333333333333333	0	4	
2184	1	0.25	0	4	
2292	1	0.25	0	4	
2298	1	0.25	0	4	
2326	1	0.25	0	4	
2339	1	0.25	0	4	
2353	1	0.25	0	4	
2407	1	0.333333333333333	0	4	
2412	1	0.333333333333333	0	4	
2423	1	0.333333333333333	0	4	
2495	1	0.25	0	4	
2496	2	0.5	0	2	
2500	1	0.333333333333333	0	4	
2503	1	0.25	0	4	
2505	1	0.25	0	4	
2511	1	0.25	0	4	
2523	1	0.25	0	4	
2533	1	0.25	0	4	
2535	1	0.25	0	4	
2536	1	0.25	0	4	
2549	1	0.333333333333333	0	4	
2563	1	0.333333333333333	0	4	
2564	1	0.333333333333333	0	4	
2566	1	0.333333333333333	0	4	
2567	2	0.666666666666667	0	2	
2571	1	0.333333333333333	0	4	
2589	1	0.25	0	4	
2596	1	0.333333333333333	0	4	
2607	1	0.333333333333333	0	4	
2629	1	0.25	0	4	
2630	1	0.25	0	4	
2646	1	0.25	0	4	
2650	2	0.5	0	2	
2651	1	0.25	0	4	
2652	1	0.25	0	4	
2661	1	0.25	0	4	
2676	1	0.25	0	4	
2678	1	0.25	0	4	
2699	1	0.333333333333333	0	4	
2700	1	0.333333333333333	0	4	
2744	1	0.25	0	4	
2755	1	0.333333333333333	0	4	
2766	1	0.333333333333333	0	4	
2778	1	0.333333333333333	0	4	
2781	1	0.5	0	4	
2842	1	0.5	0	4	
2896	1	0.5	0	4	

2902 1 1 1 4

---

category=0, cleavage_site=1770
query=ptc-miR171c,d, target=Potri.002G144200.1,
score=1, range=1759-1779, strand=1

```
target  5' GAUAUUGGCGCgGCUCAAUCA 3'
            ::::::::::::::::::::
query   3' CUAUAACCGCGCCGAGUUAGA 5'
```

>Potri.002G144200.1
#size=3548

43	2	0.666666666666667	0	2	
174	1	0.333333333333333	0	4	
216	1	0.333333333333333	0	4	
264	1	0.333333333333333	0	4	
565	1	0.2	0	4	
828	1	0.333333333333333	0	4	
1040	1	0.25	0	4	
1134	1	0.5	0	4	
1320	1	0.166666666666667	0	4	
1432	1	0.125	0	4	
1699	1	0.111111111111111	0	4	
1740	1	0.111111111111111	0	4	
1753	1	0.1	0	4	
1755	1	0.111111111111111	0	4	
1766	1	0.111111111111111	0	4	
1767	13	1.57142857142857	0	2	
1770	21	2.58730158730159	0	0	<<<
1771	1	0.111111111111111	0	4	
1773	1	0.25	0	4	
1776	1	0.111111111111111	0	4	
1778	1	0.25	0	4	
1930	1	0.142857142857143	0	4	
2014	1	0.25	0	4	
2060	1	0.125	0	4	
2088	1	0.111111111111111	0	4	
2099	1	0.125	0	4	
2128	1	0.333333333333333	0	4	
2275	1	0.333333333333333	0	4	
2291	1	0.333333333333333	0	4	
2556	1	0.333333333333333	0	4	
2557	1	0.333333333333333	0	4	
2675	1	0.333333333333333	0	4	
2697	1	0.333333333333333	0	4	
2765	2	0.666666666666667	0	2	
2768	1	0.333333333333333	0	4	
2856	1	0.333333333333333	0	4	
2857	1	0.333333333333333	0	4	
2901	1	0.333333333333333	0	4	
2915	1	0.333333333333333	0	4	
2949	1	0.333333333333333	0	4	
2963	1	0.333333333333333	0	4	
2977	1	0.333333333333333	0	4	
3147	1	0.333333333333333	0	4	
3337	1	0.333333333333333	0	4	
3391	1	0.333333333333333	0	4	

category=0, cleavage_site=1929
query=ptc-miR171c,d, target=Potri.002G144700.1,
score=1, range=1918-1938, strand=1

```
target  5' GAUAUUGGCGCgGCUCAAUCA 3'
            ::::::::::::::::::::
query   3' CUAUAACCGCGCCGAGUUAGA 5'
```

>Potri.002G144700.1
#size=3311

1011	1	0.166666666666667	0	4	
1316	1	0.25	0	4	
1468	1	0.166666666666667	0	4	
1485	1	0.166666666666667	0	4	
1597	1	0.125	0	4	
1807	1	0.166666666666667	0	4	
1858	1	0.111111111111111	0	4	
1868	1	0.166666666666667	0	4	
1899	1	0.111111111111111	0	4	
1912	1	0.1	0	4	
1914	1	0.111111111111111	0	4	
1925	1	0.111111111111111	0	4	
1926	13	1.57142857142857	0	2	
1929	21	2.58730158730159	0	0	<<<
1930	1	0.111111111111111	0	4	
1932	1	0.25	0	4	
1935	1	0.111111111111111	0	4	
1937	1	0.25	0	4	
2021	1	1	1	4	
2089	1	0.142857142857143	0	4	
2173	1	0.25	0	4	
2247	1	0.111111111111111	0	4	
2613	1	0.25	0	4	
2802	1	1	1	4	
2811	1	1	1	4	
2813	1	0.333333333333333	0	4	
2839	1	0.166666666666667	0	4	
2845	1	0.25	0	4	
2858	1	0.25	0	4	
2920	1	1	1	4	
2927	1	1	1	4	
2938	1	1	1	4	
2942	1	1	1	4	
3102	1	0.25	0	4	
3111	2	2	2	2	

category=0, cleavage_site=1472
query=ptc-miR171c,d, target=Potri.014G060200.1,
score=1, range=1461-1481, strand=1

```
target  5' GAUAUUGGCGCgGCUCAAUCA 3'
          ::::::::::::::::::::
query   3' CUAUAACCGCGCCGAGUUAGA 5'
```

>Potri.014G060200.1
#size=2475

42	1	0.5	0	4
70	1	0.5	0	4
101	1	0.5	0	4
105	1	0.5	0	4
114	1	0.5	0	4

128	1	0.25	0	4	
130	1	0.5	0	4	
162	1	0.5	0	4	
390	1	0.2	0	4	
424	1	0.2	0	4	
557	1	0.166666666666667	0	4	
662	1	0.5	0	4	
704	1	0.2	0	4	
705	1	0.2	0	4	
756	1	0.2	0	4	
795	1	0.5	0	4	
865	1	0.2	0	4	
993	1	0.166666666666667	0	4	
1035	1	0.5	0	4	
1043	1	0.5	0	4	
1049	1	0.5	0	4	
1122	1	0.125	0	4	
1128	1	0.25	0	4	
1318	1	0.5	0	4	
1350	1	0.166666666666667	0	4	
1401	1	0.111111111111111	0	4	
1411	1	0.166666666666667	0	4	
1442	1	0.111111111111111	0	4	
1455	1	0.1	0	4	
1457	1	0.111111111111111	0	4	
1467	2	1	0	2	
1468	1	0.111111111111111	0	4	
1469	9	1	0	2	
1472	13	1.44444444444444	0	0	<<<
1473	1	0.111111111111111	0	4	
1478	1	0.111111111111111	0	4	
1612	1	0.2	0	4	
1762	1	0.125	0	4	
1790	1	0.111111111111111	0	4	
1801	1	0.125	0	4	
1918	1	0.2	0	4	
2035	1	0.5	0	4	
2050	1	0.5	0	4	
2104	1	0.5	0	4	
2155	1	0.2	0	4	
2161	1	0.5	0	4	
2269	1	0.5	0	4	
2301	1	0.5	0	4	
2338	1	0.2	0	4	
2341	1	0.2	0	4	
2347	1	0.5	0	4	
2348	1	0.2	0	4	
2353	1	0.5	0	4	
2356	1	0.333333333333333	0	4	
2382	1	0.166666666666667	0	4	
2387	1	0.5	0	4	
2400	2	1	0	2	
2414	1	0.2	0	4	
2418	1	0.5	0	4	
2423	1	0.5	0	4	

category=0, cleavage_site=1502
query=ptc-miR171c,d, target=Potri.014G060500.1,
score=1, range=1491-1511, strand=1

```
target  5' GAUAUUGGCGCgGCUCAAUCA 3'
           :::::::::::::::::::::
query   3' CUAUAACCGCGCCGAGUUAGA 5'
```

>Potri.014G060500.1
#size=2923

11	1	0.5	0	4	
63	1	0.5	0	4	
86	1	0.5	0	4	
122	1	0.5	0	4	
136	2	1	0	2	
142	1	0.25	0	4	
145	1	0.5	0	4	
149	1	0.5	0	4	
150	1	0.5	0	4	
162	1	0.5	0	4	
442	1	0.2	0	4	
575	1	0.166666666666667	0	4	
703	1	0.333333333333333	0	4	
722	1	0.2	0	4	
723	1	0.2	0	4	
774	1	0.2	0	4	
868	1	0.333333333333333	0	4	
883	1	0.2	0	4	
895	1	0.333333333333333	0	4	
907	1	0.333333333333333	0	4	
1026	1	0.166666666666667	0	4	
1043	1	0.166666666666667	0	4	
1155	1	0.125	0	4	
1161	1	0.25	0	4	
1380	1	0.166666666666667	0	4	
1431	1	0.111111111111111	0	4	
1441	1	0.166666666666667	0	4	
1472	1	0.111111111111111	0	4	
1485	1	0.1	0	4	
1487	1	0.111111111111111	0	4	
1498	1	0.111111111111111	0	4	
1499	13	1.57142857142857	0	2	
1502	21	2.58730158730159	0	0	<<<
1503	1	0.111111111111111	0	4	
1508	1	0.111111111111111	0	4	
1642	1	0.2	0	4	
1662	1	0.142857142857143	0	4	
1792	1	0.125	0	4	
1820	1	0.111111111111111	0	4	
1831	1	0.125	0	4	
1948	1	0.2	0	4	
1978	1	0.333333333333333	0	4	
1990	1	0.333333333333333	0	4	
2057	2	0.666666666666667	0	2	
2161	1	0.333333333333333	0	4	
2167	1	0.333333333333333	0	4	
2179	1	0.333333333333333	0	4	
2185	1	0.2	0	4	
2186	1	0.25	0	4	

2266	1	0.333333333333333	0	4
2267	1	0.333333333333333	0	4
2281	1	0.333333333333333	0	4
2368	1	0.2	0	4
2371	1	0.2	0	4
2378	1	0.2	0	4
2406	1	0.333333333333333	0	4
2412	1	0.166666666666667	0	4
2418	1	0.25	0	4
2431	1	0.25	0	4
2444	1	0.2	0	4
2507	2	0.666666666666667	0	2
2513	1	0.333333333333333	0	4
2559	1	0.333333333333333	0	4
2569	1	0.333333333333333	0	4
2574	1	0.333333333333333	0	4
2587	1	0.333333333333333	0	4
2593	1	0.333333333333333	0	4
2600	1	0.333333333333333	0	4
2606	1	0.333333333333333	0	4
2607	1	0.333333333333333	0	4
2609	1	0.333333333333333	0	4
2611	1	0.333333333333333	0	4
2622	1	0.333333333333333	0	4
2634	1	0.333333333333333	0	4
2638	1	0.333333333333333	0	4
2642	1	0.333333333333333	0	4
2650	2	0.666666666666667	0	2
2654	1	0.333333333333333	0	4
2663	1	0.25	0	4
2672	1	0.333333333333333	0	4
2731	1	0.333333333333333	0	4

ptc-miR171e-i

category=0, cleavage_site=1767
query=ptc-miR171e-i, target=Potri.001G122800.1,
score=1, range=1756-1776, strand=1

```
target  5' GAUAUUGGCGCgGCUCAAUCA 3'
           :::::::::.:::::::::::
query   3' CUAUAACCGUGCCGAGUUAGU 5'
```

>Potri.001G122800.1
#size=2999
543	1	0.111111111111111	0	4
569	1	0.333333333333333	0	4
651	1	0.333333333333333	0	4
1169	1	0.333333333333333	0	4
1250	1	0.333333333333333	0	4
1264	1	0.333333333333333	0	4
1504	1	0.333333333333333	0	4
1602	1	0.25	0	4
1604	1	0.333333333333333	0	4
1646	1	0.1	0	4
1750	1	0.1	0	4
1764	1	0.333333333333333	0	4
1767	8	2.66666666666667	0	0	<<<
2045	1	0.25	0	4
2074	1	0.333333333333333	0	4

2149	1	0.333333333333333	0	4
2184	1	0.25	0	4
2292	1	0.25	0	4
2298	1	0.25	0	4
2326	1	0.25	0	4
2339	1	0.25	0	4
2353	1	0.25	0	4
2407	1	0.333333333333333	0	4
2412	1	0.333333333333333	0	4
2423	1	0.333333333333333	0	4
2495	1	0.25	0	4
2496	2	0.5	0	2
2500	1	0.333333333333333	0	4
2503	1	0.25	0	4
2505	1	0.25	0	4
2511	1	0.25	0	4
2523	1	0.25	0	4
2533	1	0.25	0	4
2535	1	0.25	0	4
2536	1	0.25	0	4
2549	1	0.333333333333333	0	4
2563	1	0.333333333333333	0	4
2564	1	0.333333333333333	0	4
2566	1	0.333333333333333	0	4
2567	2	0.666666666666667	0	2
2571	1	0.333333333333333	0	4
2589	1	0.25	0	4
2596	1	0.333333333333333	0	4
2607	1	0.333333333333333	0	4
2629	1	0.25	0	4
2630	1	0.25	0	4
2646	1	0.25	0	4
2650	2	0.5	0	2
2651	1	0.25	0	4
2652	1	0.25	0	4
2661	1	0.25	0	4
2676	1	0.25	0	4
2678	1	0.25	0	4
2699	1	0.333333333333333	0	4
2700	1	0.333333333333333	0	4
2744	1	0.25	0	4
2755	1	0.333333333333333	0	4
2766	1	0.333333333333333	0	4
2778	1	0.333333333333333	0	4
2781	1	0.5	0	4
2842	1	0.5	0	4
2896	1	0.5	0	4
2902	1	1	1	4

category=0, cleavage_site=1770
query=ptc-miR171e-i, target=Potri.002G144200.1,
score=1, range=1759-1779, strand=1

```
target  5' GAUAUUGGCGCgGCUCAAUCA 3'
           :::::::::.:::::::::::
query   3' CUAUAACCGUGCCGAGUUAGU 5'
```

>Potri.002G144200.1
#size=3548

43	2	0.666666666666667	0	2

174 1 0.333333333333333 0 4
216 1 0.333333333333333 0 4
264 1 0.333333333333333 0 4
565 1 0.2 0 4
828 1 0.333333333333333 0 4
1040 1 0.25 0 4
1134 1 0.5 0 4
1320 1 0.166666666666667 0 4
1432 1 0.125 0 4
1699 1 0.111111111111111 0 4
1740 1 0.111111111111111 0 4
1753 1 0.1 0 4
1755 1 0.111111111111111 0 4
1766 1 0.111111111111111 0 4
1767 13 1.57142857142857 0 2
1770 21 2.58730158730159 0 0 <<<
1771 1 0.111111111111111 0 4
1773 1 0.25 0 4
1776 1 0.111111111111111 0 4
1778 1 0.25 0 4
1930 1 0.142857142857143 0 4
2014 1 0.25 0 4
2060 1 0.125 0 4
2088 1 0.111111111111111 0 4
2099 1 0.125 0 4
2128 1 0.333333333333333 0 4
2275 1 0.333333333333333 0 4
2291 1 0.333333333333333 0 4
2556 1 0.333333333333333 0 4
2557 1 0.333333333333333 0 4
2675 1 0.333333333333333 0 4
2697 1 0.333333333333333 0 4
2765 2 0.666666666666667 0 2
2768 1 0.333333333333333 0 4
2856 1 0.333333333333333 0 4
2857 1 0.333333333333333 0 4
2901 1 0.333333333333333 0 4
2915 1 0.333333333333333 0 4
2949 1 0.333333333333333 0 4
2963 1 0.333333333333333 0 4
2977 1 0.333333333333333 0 4
3147 1 0.333333333333333 0 4
3337 1 0.333333333333333 0 4
3391 1 0.333333333333333 0 4

---

category=0, cleavage_site=1929
query=ptc-miR171e-i, target=Potri.002G144700.1,
score=1, range=1918-1938, strand=1

```
target  5' GAUAUUGGCGCgGCUCAAUCA 3'
           :::::::::.::::::::::::
query   3' CUAUAACCGUGCCGAGUUAGU 5'
```

>Potri.002G144700.1
#size=3311
1011 1 0.166666666666667 0 4
1316 1 0.25 0 4
1468 1 0.166666666666667 0 4
1485 1 0.166666666666667 0 4
1597 1 0.125 0 4

1807	1	0.166666666666667	0	4	
1858	1	0.111111111111111	0	4	
1868	1	0.166666666666667	0	4	
1899	1	0.111111111111111	0	4	
1912	1	0.1	0	4	
1914	1	0.111111111111111	0	4	
1925	1	0.111111111111111	0	4	
1926	13	1.57142857142857	0	2	
1929	21	2.58730158730159	0	0	<<<
1930	1	0.111111111111111	0	4	
1932	1	0.25	0	4	
1935	1	0.111111111111111	0	4	
1937	1	0.25	0	4	
2021	1	1	1	4	
2089	1	0.142857142857143	0	4	
2173	1	0.25	0	4	
2247	1	0.111111111111111	0	4	
2613	1	0.25	0	4	
2802	1	1	1	4	
2811	1	1	1	4	
2813	1	0.333333333333333	0	4	
2839	1	0.166666666666667	0	4	
2845	1	0.25	0	4	
2858	1	0.25	0	4	
2920	1	1	1	4	
2927	1	1	1	4	
2938	1	1	1	4	
2942	1	1	1	4	
3102	1	0.25	0	4	
3111	2	2	2	2	

---

category=0, cleavage_site=1472
query=ptc-miR171e-i, target=Potri.014G060200.1,
score=1, range=1461-1481, strand=1

```
target  5' GAUAUUGGCGCgGCUCAAUCA 3'
           :::::::::.:::::::::::
query   3' CUAUAACCGUGCCGAGUUAGU 5'
```

>Potri.014G060200.1
#size=2475

42	1	0.5	0	4
70	1	0.5	0	4
101	1	0.5	0	4
105	1	0.5	0	4
114	1	0.5	0	4
128	1	0.25	0	4
130	1	0.5	0	4
162	1	0.5	0	4
390	1	0.2	0	4
424	1	0.2	0	4
557	1	0.166666666666667	0	4
662	1	0.5	0	4
704	1	0.2	0	4
705	1	0.2	0	4
756	1	0.2	0	4
795	1	0.5	0	4

865	1	0.2	0	4	
993	1	0.166666666666667	0	4	
1035	1	0.5	0	4	
1043	1	0.5	0	4	
1049	1	0.5	0	4	
1122	1	0.125	0	4	
1128	1	0.25	0	4	
1318	1	0.5	0	4	
1350	1	0.166666666666667	0	4	
1401	1	0.111111111111111	0	4	
1411	1	0.166666666666667	0	4	
1442	1	0.111111111111111	0	4	
1455	1	0.1	0	4	
1457	1	0.111111111111111	0	4	
1467	2	1	0	2	
1468	1	0.111111111111111	0	4	
1469	9	1	0	2	
1472	13	1.44444444444444	0	0	<<<
1473	1	0.111111111111111	0	4	
1478	1	0.111111111111111	0	4	
1612	1	0.2	0	4	
1762	1	0.125	0	4	
1790	1	0.111111111111111	0	4	
1801	1	0.125	0	4	
1918	1	0.2	0	4	
2035	1	0.5	0	4	
2050	1	0.5	0	4	
2104	1	0.5	0	4	
2155	1	0.2	0	4	
2161	1	0.5	0	4	
2269	1	0.5	0	4	
2301	1	0.5	0	4	
2338	1	0.2	0	4	
2341	1	0.2	0	4	
2347	1	0.5	0	4	
2348	1	0.2	0	4	
2353	1	0.5	0	4	
2356	1	0.333333333333333	0	4	
2382	1	0.166666666666667	0	4	
2387	1	0.5	0	4	
2400	2	1	0	2	
2414	1	0.2	0	4	
2418	1	0.5	0	4	
2423	1	0.5	0	4	

---

category=0, cleavage_site=1502
query=ptc-miR171e-i, target=Potri.014G060500.1,
score=1, range=1491-1511, strand=1

```
target  5' GAUAUUGGCGCgGCUCAAUCA 3'
           :::::::::.::::::::::: 
query   3' CUAUAACCGUGCCGAGUUAGU 5'
```

>Potri.014G060500.1
#size=2923

11	1	0.5	0	4
63	1	0.5	0	4
86	1	0.5	0	4
122	1	0.5	0	4
136	2	1	0	2

142	1	0.25	0	4	
145	1	0.5	0	4	
149	1	0.5	0	4	
150	1	0.5	0	4	
162	1	0.5	0	4	
442	1	0.2	0	4	
575	1	0.166666666666667	0	4	
703	1	0.333333333333333	0	4	
722	1	0.2	0	4	
723	1	0.2	0	4	
774	1	0.2	0	4	
868	1	0.333333333333333	0	4	
883	1	0.2	0	4	
895	1	0.333333333333333	0	4	
907	1	0.333333333333333	0	4	
1026	1	0.166666666666667	0	4	
1043	1	0.166666666666667	0	4	
1155	1	0.125	0	4	
1161	1	0.25	0	4	
1380	1	0.166666666666667	0	4	
1431	1	0.111111111111111	0	4	
1441	1	0.166666666666667	0	4	
1472	1	0.111111111111111	0	4	
1485	1	0.1	0	4	
1487	1	0.111111111111111	0	4	
1498	1	0.111111111111111	0	4	
1499	13	1.57142857142857	0	2	
1502	21	2.58730158730159	0	0	<<<
1503	1	0.111111111111111	0	4	
1508	1	0.111111111111111	0	4	
1642	1	0.2	0	4	
1662	1	0.142857142857143	0	4	
1792	1	0.125	0	4	
1820	1	0.111111111111111	0	4	
1831	1	0.125	0	4	
1948	1	0.2	0	4	
1978	1	0.333333333333333	0	4	
1990	1	0.333333333333333	0	4	
2057	2	0.666666666666667	0	2	
2161	1	0.333333333333333	0	4	
2167	1	0.333333333333333	0	4	
2179	1	0.333333333333333	0	4	
2185	1	0.2	0	4	
2186	1	0.25	0	4	
2266	1	0.333333333333333	0	4	
2267	1	0.333333333333333	0	4	
2281	1	0.333333333333333	0	4	
2368	1	0.2	0	4	
2371	1	0.2	0	4	
2378	1	0.2	0	4	
2406	1	0.333333333333333	0	4	
2412	1	0.166666666666667	0	4	
2418	1	0.25	0	4	
2431	1	0.25	0	4	
2444	1	0.2	0	4	
2507	2	0.666666666666667	0	2	
2513	1	0.333333333333333	0	4	
2559	1	0.333333333333333	0	4	

2569	1	0.333333333333333	0	4
2574	1	0.333333333333333	0	4
2587	1	0.333333333333333	0	4
2593	1	0.333333333333333	0	4
2600	1	0.333333333333333	0	4
2606	1	0.333333333333333	0	4
2607	1	0.333333333333333	0	4
2609	1	0.333333333333333	0	4
2611	1	0.333333333333333	0	4
2622	1	0.333333333333333	0	4
2634	1	0.333333333333333	0	4
2638	1	0.333333333333333	0	4
2642	1	0.333333333333333	0	4
2650	2	0.666666666666667	0	2
2654	1	0.333333333333333	0	4
2663	1	0.25	0	4
2672	1	0.333333333333333	0	4
2731	1	0.333333333333333	0	4

ptc-miR172a-c,f

category=0, cleavage_site=1755
query=ptc-miR172a-c,f, target=Potri.005G140700.1,
score=2, range=1744-1764, strand=1

```
target  5' CUGCAGCAUCAuCAGGAUUCU 3'
           :::::::::::.:::::::
query   3' UACGUCGUAGUAGUUCUAAGA 5'
```

>Potri.005G140700.1
#size=2188

196	1	0.25	0	4
231	1	0.142857142857143	0	4
247	1	0.142857142857143	0	4
304	1	0.142857142857143	0	4
476	2	0.285714285714286	0	2
516	1	0.25	0	4
536	1	0.142857142857143	0	4
677	1	0.142857142857143	0	4
694	1	0.142857142857143	0	4
695	1	0.142857142857143	0	4
701	1	0.25	0	4
702	1	0.142857142857143	0	4
788	2	0.2	0	2
812	1	0.142857142857143	0	4
819	1	0.142857142857143	0	4
821	1	0.142857142857143	0	4
834	1	0.142857142857143	0	4
835	1	0.142857142857143	0	4
852	1	0.142857142857143	0	4
855	1	0.142857142857143	0	4
867	1	0.142857142857143	0	4
876	1	0.142857142857143	0	4
881	1	0.142857142857143	0	4
900	1	0.25	0	4
907	1	0.25	0	4
928	1	0.25	0	4
955	1	0.142857142857143	0	4
974	1	0.25	0	4
976	1	0.142857142857143	0	4

979 1 0.142857142857143 0 4
987 2 0.285714285714286 0 2
999 1 0.142857142857143 0 4
1000 3 0.428571428571429 0 2
1001 2 0.285714285714286 0 2
1012 1 0.142857142857143 0 4
1038 1 0.25 0 4
1042 1 0.142857142857143 0 4
1045 1 0.142857142857143 0 4
1101 1 0.142857142857143 0 4
1122 1 0.142857142857143 0 4
1156 1 0.142857142857143 0 4
1164 1 0.142857142857143 0 4
1337 1 0.25 0 4
1426 1 0.142857142857143 0 4
1440 1 0.142857142857143 0 4
1454 1 0.142857142857143 0 4
1473 1 0.142857142857143 0 4
1475 1 0.142857142857143 0 4
1522 1 0.25 0 4
1558 1 0.25 0 4
1572 1 0.25 0 4
1583 1 0.25 0 4
1701 1 0.142857142857143 0 4
1713 1 0.142857142857143 0 4
1755 32 5.33333333333333 0 0 <<<
1756 1 0.166666666666667 0 4
1759 1 0.166666666666667 0 4
1760 2 0.333333333333333 0 2
1761 1 0.166666666666667 0 4
1762 1 0.166666666666667 0 4
1763 1 0.166666666666667 0 4
1768 3 0.5 0 2
1770 2 0.333333333333333 0 2
1772 4 0.666666666666667 0 2
1775 1 0.166666666666667 0 4
1784 1 0.166666666666667 0 4
1831 2 0.666666666666667 0 2
1833 1 0.333333333333333 0 4
1841 1 0.333333333333333 0 4
1874 1 0.333333333333333 0 4
1888 1 0.333333333333333 0 4
1894 1 0.333333333333333 0 4
1950 1 0.333333333333333 0 4
1952 1 0.333333333333333 0 4
2068 1 0.333333333333333 0 4
2085 1 0.166666666666667 0 4
2092 1 0.333333333333333 0 4

---

category=2, cleavage_site=1479
query=ptc-miR172a-c,f, target=Potri.006G132400.1,
score=3, range=1468-1488, strand=1

```
target  5' CUGCAGCAUCAuCAGGAUUCG 3'
           :::::::::::.:::::
query   3' UACGUCGUAGUAGUUCUAAGA 5'
```

>Potri.006G132400.1
#size=2157
11 1 0.5 0 4

27	2	1	0	2
66	1	0.5	0	4
87	1	0.5	0	4
151	1	0.5	0	4
156	1	0.5	0	4
209	1	0.25	0	4
217	1	0.25	0	4
221	1	0.25	0	4
234	1	0.5	0	4
240	1	0.5	0	4
268	1	0.25	0	4
282	1	0.5	0	4
296	1	0.5	0	4
309	1	0.5	0	4
332	1	0.5	0	4
336	1	0.5	0	4
339	1	0.5	0	4
341	1	0.5	0	4
344	1	0.5	0	4
347	1	0.5	0	4
351	1	0.5	0	4
382	1	0.5	0	4
387	2	1	0	2
388	1	0.5	0	4
390	1	0.5	0	4
394	4	2	0	2
398	1	0.5	0	4
399	2	1	0	2
405	1	0.5	0	4
408	1	0.5	0	4
413	2	0.5	0	2
414	3	0.75	0	2
415	2	1	0	2
416	9	4.25	0	2
417	10	4.75	0	2
418	3	1.5	0	2
419	1	0.5	0	4
423	2	1	0	2
424	1	0.5	0	4
426	1	0.5	0	4
427	1	0.5	0	4
428	1	0.5	0	4
429	1	0.5	0	4
430	1	0.5	0	4
431	1	0.5	0	4
434	1	0.5	0	4
435	2	1	0	2
436	2	1	0	2
439	1	0.5	0	4
442	1	0.5	0	4
444	1	0.5	0	4
445	3	1.5	0	2
446	2	1	0	2
449	1	0.5	0	4
451	1	0.5	0	4
456	1	0.5	0	4
457	1	0.5	0	4
458	3	1.5	0	2

472	2	1	0	2
474	1	0.5	0	4
476	1	0.5	0	4
485	1	0.5	0	4
494	3	1.5	0	2
496	1	0.5	0	4
498	2	1	0	2
500	2	1	0	2
502	1	0.5	0	4
504	1	0.5	0	4
508	1	0.5	0	4
513	1	0.5	0	4
517	1	0.5	0	4
538	2	1	0	2
544	1	0.5	0	4
545	3	1.5	0	2
546	6	3	0	2
547	4	2	0	2
548	22	11	0	0
549	4	2	0	2
550	4	2	0	2
551	1	0.5	0	4
553	3	1.5	0	2
554	2	1	0	2
556	4	2	0	2
558	2	1	0	2
567	1	0.25	0	4
576	1	0.25	0	4
577	1	0.25	0	4
583	1	0.25	0	4
591	1	0.25	0	4
592	2	0.5	0	2
599	2	0.5	0	2
608	2	0.5	0	2
612	1	0.25	0	4
613	1	0.25	0	4
614	2	0.5	0	2
621	1	0.25	0	4
624	1	0.25	0	4
628	2	0.5	0	2
638	3	0.75	0	2
641	1	0.25	0	4
642	1	0.25	0	4
648	2	0.5	0	2
651	1	0.25	0	4
660	1	0.25	0	4
661	1	0.25	0	4
663	1	0.25	0	4
673	2	0.5	0	2
677	2	0.5	0	2
679	1	0.25	0	4
681	1	0.25	0	4
684	1	0.25	0	4
691	1	0.25	0	4
696	1	0.25	0	4
739	1	0.5	0	4
743	1	0.5	0	4
776	1	0.25	0	4

786	1	0.5	0	4
789	1	0.5	0	4
794	1	0.5	0	4
820	1	0.5	0	4
822	1	0.5	0	4
823	1	0.5	0	4
825	1	0.5	0	4
827	2	1	0	2
835	1	0.5	0	4
836	1	0.5	0	4
848	1	0.5	0	4
862	1	0.25	0	4
864	1	0.25	0	4
865	2	0.5	0	2
873	1	0.5	0	4
892	1	0.5	0	4
900	1	0.5	0	4
905	1	0.5	0	4
913	1	0.25	0	4
927	1	0.333333333333333	0	4
933	1	0.333333333333333	0	4
936	1	0.333333333333333	0	4
949	1	0.333333333333333	0	4
971	1	0.333333333333333	0	4
976	1	0.333333333333333	0	4
991	1	0.333333333333333	0	4
1016	1	0.25	0	4
1034	1	0.25	0	4
1037	1	0.25	0	4
1056	1	0.5	0	4
1066	1	0.5	0	4
1070	1	0.5	0	4
1100	1	0.25	0	4
1103	1	0.25	0	4
1106	1	0.25	0	4
1109	1	0.25	0	4
1113	1	0.5	0	4
1121	1	0.5	0	4
1126	1	0.5	0	4
1128	1	0.5	0	4
1131	1	0.5	0	4
1169	1	0.333333333333333	0	4
1170	1	0.333333333333333	0	4
1192	1	1	1	4
1264	1	0.5	0	4
1273	1	0.5	0	4
1293	1	0.5	0	4
1342	1	0.25	0	4
1349	1	0.25	0	4
1355	1	0.25	0	4
1368	1	0.25	0	4
1378	1	0.25	0	4
1379	1	0.25	0	4
1381	1	0.25	0	4
1382	1	0.25	0	4
1414	1	0.25	0	4
1415	1	0.25	0	4
1424	1	0.25	0	4

1449	1	0.25	0	4	
1452	1	0.25	0	4	
1458	1	0.25	0	4	
1479	17	4.25	0	2	<<<
1480	2	0.5	0	2	
1504	1	0.5	0	4	
1506	1	0.5	0	4	
1508	1	0.25	0	4	
1509	4	1.25	0	2	
1515	1	0.5	0	4	
1533	1	0.5	0	4	
1540	1	0.5	0	4	
1542	1	0.5	0	4	
1543	2	1	0	2	
1544	1	0.5	0	4	
1545	1	0.5	0	4	
1552	2	1	0	2	
1555	2	1	0	2	
1556	1	0.5	0	4	
1560	1	0.5	0	4	
1563	1	0.5	0	4	
1575	1	0.25	0	4	
1580	1	0.25	0	4	
1596	1	0.25	0	4	
1605	1	0.5	0	4	
1612	1	0.25	0	4	
1613	1	0.25	0	4	
1617	1	0.25	0	4	
1636	1	0.5	0	4	
1644	1	0.5	0	4	
1652	3	1.5	0	2	
1711	1	0.5	0	4	
1717	1	0.5	0	4	
1734	1	0.25	0	4	
1737	1	0.5	0	4	
1784	1	0.5	0	4	
1806	2	1	0	2	
1847	1	0.5	0	4	
1866	1	0.5	0	4	
1872	1	0.5	0	4	
1914	1	0.5	0	4	
1923	1	0.5	0	4	

---

category=0, cleavage_site=2149
query=ptc-miR172a-c,f, target=Potri.007G046200.1,
score=3, range=2138-2158, strand=1

```
target  5' CUGCAGCAUCAuCAGGAUUCC 3'
             ::::::::::::.:::::
query   3' UACGUCGUAGUAGUUCUAAGA 5'
```

>Potri.007G046200.1
#size=2909

266	1	0.333333333333333	0	4
366	1	0.333333333333333	0	4
414	2	0.666666666666667	0	2
420	1	0.333333333333333	0	4
459	1	0.333333333333333	0	4
546	1	0.333333333333333	0	4
597	1	0.333333333333333	0	4

599	1	0.333333333333333	0	4
631	1	0.142857142857143	0	4
647	1	0.142857142857143	0	4
696	1	0.333333333333333	0	4
704	1	0.142857142857143	0	4
834	1	0.333333333333333	0	4
853	1	0.333333333333333	0	4
876	2	0.285714285714286	0	3
936	1	0.142857142857143	0	4
986	1	0.333333333333333	0	4
1059	1	0.333333333333333	0	4
1063	1	0.333333333333333	0	4
1089	1	0.142857142857143	0	4
1106	1	0.142857142857143	0	4
1107	1	0.142857142857143	0	4
1114	1	0.142857142857143	0	4
1200	2	0.2 0 3		
1224	1	0.142857142857143	0	4
1231	1	0.142857142857143	0	4
1233	1	0.142857142857143	0	4
1246	1	0.142857142857143	0	4
1247	1	0.142857142857143	0	4
1264	1	0.142857142857143	0	4
1267	1	0.142857142857143	0	4
1279	1	0.142857142857143	0	4
1288	1	0.142857142857143	0	4
1293	1	0.142857142857143	0	4
1352	1	0.333333333333333	0	4
1353	1	0.333333333333333	0	4
1367	1	0.142857142857143	0	4
1388	1	0.142857142857143	0	4
1391	1	0.142857142857143	0	4
1399	2	0.285714285714286	0	3
1411	1	0.142857142857143	0	4
1412	3	0.428571428571429	0	2
1413	2	0.285714285714286	0	3
1424	1	0.142857142857143	0	4
1454	1	0.142857142857143	0	4
1457	1	0.142857142857143	0	4
1475	1	0.333333333333333	0	4
1513	1	0.142857142857143	0	4
1526	1	0.333333333333333	0	4
1534	1	0.142857142857143	0	4
1568	1	0.142857142857143	0	4
1576	1	0.142857142857143	0	4
1597	1	0.5 0 4		
1686	1	0.333333333333333	0	4
1713	2	0.666666666666667	0	2
1718	1	0.333333333333333	0	4
1719	1	0.333333333333333	0	4
1729	1	0.333333333333333	0	4
1771	2	0.666666666666667	0	2
1789	1	0.333333333333333	0	4
1794	1	0.333333333333333	0	4
1807	1	0.333333333333333	0	4
1808	3	1 0 2		
1812	1	0.333333333333333	0	4
1820	1	0.142857142857143	0	4

1834	1	0.142857142857143	0	4	
1848	1	0.142857142857143	0	4	
1867	1	0.142857142857143	0	4	
1869	1	0.142857142857143	0	4	
1919	1	0.333333333333333	0	4	
1941	1	0.333333333333333	0	4	
1956	1	0.333333333333333	0	4	
1979	1	0.333333333333333	0	4	
1990	1	0.5	0	4	
2030	1	0.333333333333333	0	4	
2075	1	0.333333333333333	0	4	
2095	1	0.142857142857143	0	4	
2107	1	0.142857142857143	0	4	
2149	32	5.33333333333333	0	0	<<<
2150	1	0.166666666666667	0	4	
2153	1	0.166666666666667	0	4	
2154	3	0.666666666666667	0	2	
2155	1	0.166666666666667	0	4	
2156	1	0.166666666666667	0	4	
2157	1	0.166666666666667	0	4	
2162	3	0.5	0	2	
2164	2	0.333333333333333	0	2	
2166	4	0.666666666666667	0	2	
2169	1	0.166666666666667	0	4	
2172	1	0.333333333333333	0	4	
2174	1	0.333333333333333	0	4	
2178	1	0.166666666666667	0	4	
2179	1	0.333333333333333	0	4	
2182	1	0.333333333333333	0	4	
2184	1	0.333333333333333	0	4	
2192	1	0.333333333333333	0	4	
2193	1	0.333333333333333	0	4	
2198	1	0.333333333333333	0	4	
2201	2	0.666666666666667	0	2	
2202	1	0.333333333333333	0	4	
2206	1	0.333333333333333	0	4	
2210	1	0.333333333333333	0	4	
2211	2	0.666666666666667	0	2	
2216	1	0.333333333333333	0	4	
2226	1	0.333333333333333	0	4	
2231	1	0.333333333333333	0	4	
2235	1	0.333333333333333	0	4	
2242	3	1	0	2	
2243	3	1	0	2	
2247	1	0.333333333333333	0	4	
2301	1	0.333333333333333	0	4	
2304	1	0.333333333333333	0	4	
2305	3	1	0	2	
2306	1	0.333333333333333	0	4	
2310	2	0.666666666666667	0	2	
2311	2	0.666666666666667	0	2	
2313	2	0.666666666666667	0	2	
2317	1	0.333333333333333	0	4	
2319	1	0.333333333333333	0	4	
2320	2	0.666666666666667	0	2	
2323	1	0.333333333333333	0	4	
2326	1	0.333333333333333	0	4	
2328	1	0.333333333333333	0	4	

2330	1	0.333333333333333	0	4
2333	1	0.333333333333333	0	4
2352	1	0.333333333333333	0	4
2359	1	0.333333333333333	0	4
2380	1	0.333333333333333	0	4
2381	1	0.333333333333333	0	4
2452	1	0.333333333333333	0	4
2470	1	0.333333333333333	0	4
2502	1	0.166666666666667	0	4
2631	1	0.333333333333333	0	4
2833	2	0.666666666666667	0	2

---

category=0, cleavage_site=1958
query=ptc-miR172a-c,f, target=Potri.008G045300.1,
score=2, range=1947-1967, strand=1

```
target  5' UUGCAGCAUCAuCAGGAUUCU 3'
              :::::::::::.::::::
query   3' UACGUCGUAGUAGUUCUAAGA 5'
```

>Potri.008G045300.1
#size=2324

490	1	0.2	0	4
494	1	0.2	0	4
568	2	0.4	0	2
578	2	0.25	0	2
579	1	0.125	0	4
616	1	0.2	0	4
638	1	0.2	0	4
667	1	0.2	0	4
699	1	0.2	0	4
717	1	0.1	0	4
800	1	0.1	0	4
870	1	0.2	0	4
935	1	0.2	0	4
937	1	0.2	0	4
940	1	0.2	0	4
942	1	0.2	0	4
945	1	0.2	0	4
949	1	0.2	0	4
954	1	0.2	0	4
964	1	0.1	0	4
966	1	0.1	0	4
968	1	0.1	0	4
969	1	0.1	0	4
995	1	0.2	0	4
1002	1	0.2	0	4
1018	1	0.2	0	4
1024	1	0.1	0	4
1025	1	0.1	0	4
1029	1	0.1	0	4
1030	1	0.1	0	4
1035	1	0.1	0	4
1036	1	0.1	0	4
1045	1	0.1	0	4
1048	1	0.1	0	4
1049	1	0.1	0	4
1050	1	0.1	0	4
1051	1	0.1	0	4
1065	1	0.1	0	4

1068	1	0.1	0	4	
1071	1	0.1	0	4	
1073	1	0.1	0	4	
1074	1	0.1	0	4	
1075	1	0.1	0	4	
1076	1	0.1	0	4	
1078	1	0.1	0	4	
1080	2	0.2	0	2	
1085	1	0.1	0	4	
1086	2	0.2	0	2	
1090	1	0.1	0	4	
1092	2	0.3	0	2	
1095	1	0.1	0	4	
1097	1	0.1	0	4	
1098	1	0.1	0	4	
1150	1	0.111111111111111	0	4	
1164	1	0.2	0	4	
1169	1	0.2	0	4	
1170	1	0.2	0	4	
1199	1	0.2	0	4	
1201	1	0.111111111111111	0	4	
1222	1	0.2	0	4	
1233	1	0.2	0	4	
1268	1	0.111111111111111	0	4	
1287	1	0.111111111111111	0	4	
1325	1	0.111111111111111	0	4	
1339	1	0.111111111111111	0	4	
1340	1	0.111111111111111	0	4	
1354	1	0.111111111111111	0	4	
1370	1	0.111111111111111	0	4	
1372	1	0.111111111111111	0	4	
1394	2	0.222222222222222	0	2	
1533	1	0.25	0	4	
1544	1	0.25	0	4	
1570	1	0.25	0	4	
1586	1	0.125	0	4	
1627	1	0.25	0	4	
1644	1	0.25	0	4	
1816	1	0.25	0	4	
1901	2	0.222222222222222	0	2	
1929	1	0.111111111111111	0	4	
1958	32	8	0	0	<<<
1969	1	0.25	0	4	
1982	1	0.25	0	4	
1986	1	0.25	0	4	
1989	2	0.222222222222222	0	2	
1992	1	0.25	0	4	
1994	3	0.75	0	2	
1998	1	0.25	0	4	
2001	1	0.25	0	4	
2009	1	0.25	0	4	
2010	1	0.25	0	4	
2015	1	0.25	0	4	
2017	1	0.25	0	4	
2020	1	0.25	0	4	
2021	1	0.25	0	4	
2024	1	0.25	0	4	
2041	1	0.25	0	4	

2045	1	0.25	0	4
2052	1	0.25	0	4
2055	1	0.25	0	4
2056	1	0.25	0	4
2058	1	0.25	0	4
2060	1	0.25	0	4
2061	1	0.25	0	4
2066	1	0.25	0	4
2068	2	0.5	0	2
2198	1	0.25	0	4
2224	1	0.25	0	4
2233	1	0.25	0	4

---

category=0, cleavage_site=1779
query=ptc-miR172a-c,f, target=Potri.010G216200.1,
score=2, range=1768-1788, strand=1

```
target  5' UUGCAGCAUCAuCAGGAUUCU 3'
             ::::::::::::.:::::::
query   3' UACGUCGUAGUAGUUCUAAGA 5'
```

>Potri.010G216200.1
#size=2200

185	1	0.333333333333333	0	4
227	1	0.333333333333333	0	4
287	1	0.333333333333333	0	4
306	1	0.333333333333333	0	4
356	1	0.333333333333333	0	4
402	2	0.25	0	2
403	1	0.125	0	4
496	1	0.2	0	4
526	1	0.2	0	4
538	1	0.1	0	4
580	1	0.2	0	4
621	1	0.1	0	4
684	1	0.2	0	4
689	1	0.2	0	4
691	2	0.4	0	2
711	1	0.2	0	4
729	1	0.2	0	4
734	1	0.2	0	4
755	1	0.2	0	4
763	1	0.2	0	4
769	2	0.4	0	2
770	1	0.2	0	4
776	1	0.2	0	4
785	1	0.1	0	4
787	1	0.1	0	4
789	1	0.1	0	4
790	1	0.1	0	4
845	1	0.1	0	4
846	1	0.1	0	4
850	1	0.1	0	4
851	1	0.1	0	4
856	1	0.1	0	4
857	1	0.1	0	4
866	1	0.1	0	4
869	1	0.1	0	4
870	1	0.1	0	4
871	1	0.1	0	4

872	1	0.1	0	4		
886	1	0.1	0	4		
889	1	0.1	0	4		
892	1	0.1	0	4		
894	1	0.1	0	4		
895	1	0.1	0	4		
896	1	0.1	0	4		
897	1	0.1	0	4		
899	1	0.1	0	4		
901	2	0.2	0	2		
906	1	0.1	0	4		
907	2	0.2	0	2		
911	1	0.1	0	4		
913	1	0.1	0	4		
916	1	0.1	0	4		
918	1	0.1	0	4		
919	1	0.1	0	4		
971	1	0.111111111111111			0	4
1022	1	0.111111111111111			0	4
1029	1	0.25	0	4		
1037	1	0.25	0	4		
1038	1	0.25	0	4		
1039	1	0.25	0	4		
1040	2	0.5	0	2		
1041	1	0.25	0	4		
1042	1	0.25	0	4		
1044	1	0.25	0	4		
1047	1	0.25	0	4		
1051	1	0.25	0	4		
1054	1	0.25	0	4		
1055	1	0.25	0	4		
1058	1	0.25	0	4		
1064	2	0.5	0	2		
1071	2	0.5	0	2		
1073	1	0.25	0	4		
1089	1	0.111111111111111			0	4
1108	1	0.111111111111111			0	4
1119	1	0.25	0	4		
1122	1	0.25	0	4		
1135	1	0.25	0	4		
1146	1	0.111111111111111			0	4
1160	1	0.111111111111111			0	4
1161	1	0.111111111111111			0	4
1175	1	0.111111111111111			0	4
1191	1	0.111111111111111			0	4
1193	1	0.111111111111111			0	4
1198	1	0.25	0	4		
1215	2	0.222222222222222			0	2
1304	1	0.25	0	4		
1318	1	0.25	0	4		
1323	1	0.25	0	4		
1334	1	0.25	0	4		
1407	1	0.125	0	4		
1423	1	0.25	0	4		
1528	2	0.666666666666667			0	2
1591	1	0.2	0	4		
1607	1	0.2	0	4		
1639	1	0.2	0	4		

1663	1	0.2	0	4	
1677	2	0.4	0	2	
1680	2	0.4	0	2	
1705	1	0.2	0	4	
1722	2	0.222222222222222	0	2	
1750	1	0.111111111111111	0	4	
1777	1	0.2	0	4	
1779	37	7.4	0	0	<<<
1786	1	0.2	0	4	
1798	2	0.4	0	2	
1799	1	0.2	0	4	
1800	1	0.2	0	4	
1801	1	0.2	0	4	
1806	2	0.4	0	2	
1807	3	0.422222222222222	0	2	
1809	1	0.2	0	4	
1810	1	0.2	0	4	
1816	1	0.2	0	4	
1827	1	0.2	0	4	
1830	1	0.2	0	4	
1873	1	0.2	0	4	
1880	3	0.6	0	2	
1882	1	0.2	0	4	
1890	1	0.2	0	4	
1895	1	0.2	0	4	
1906	1	0.2	0	4	
1913	2	0.4	0	2	
1923	1	0.2	0	4	
1924	1	0.2	0	4	
1944	1	0.2	0	4	
1946	1	0.2	0	4	
2001	1	0.2	0	4	
2068	1	0.2	0	4	
2076	1	0.2	0	4	
2077	1	0.2	0	4	

---

category=0, cleavage_site=1759
query=ptc-miR172a-c,f, target=Potri.016G084500.1,
score=3, range=1748-1768, strand=1

```
target  5' CUGCAGCAUCAuCAGGAUUCG 3'
           ::::::::::::.:::::
query   3' UACGUCGUAGUAGUUCUAAGA 5'
```

>Potri.016G084500.1
#size=2370

62	1	0.25	0	4
195	1	0.5	0	4
418	1	0.5	0	4
484	1	0.5	0	4
495	1	0.25	0	4
503	1	0.25	0	4
507	1	0.25	0	4
513	1	0.5	0	4
554	1	0.25	0	4
569	1	0.5	0	4
583	1	0.5	0	4
655	1	0.5	0	4
676	1	0.5	0	4
685	1	0.5	0	4

690	3	1	0	2
691	3	0.75	0	2
693	3	1.25	0	2
694	1	0.25	0	4
695	1	0.5	0	4
696	1	0.5	0	4
704	1	0.5	0	4
710	1	0.5	0	4
720	1	0.5	0	4
724	1	0.5	0	4
728	3	1.5	0	2
730	1	0.5	0	4
733	1	0.5	0	4
734	2	1	0	2
735	1	0.5	0	4
738	1	0.5	0	4
740	2	1	0	2
750	1	0.5	0	4
766	1	0.5	0	4
774	1	0.5	0	4
817	1	0.5	0	4
824	1	0.5	0	4
833	1	0.5	0	4
834	1	0.5	0	4
847	1	0.25	0	4
856	1	0.25	0	4
857	1	0.25	0	4
863	1	0.25	0	4
871	1	0.25	0	4
872	2	0.5	0	2
879	2	0.5	0	2
888	2	0.5	0	2
892	1	0.25	0	4
893	1	0.25	0	4
894	2	0.5	0	2
901	1	0.25	0	4
904	1	0.25	0	4
908	2	0.5	0	2
918	3	0.75	0	2
921	1	0.25	0	4
922	1	0.25	0	4
928	2	0.5	0	2
931	1	0.25	0	4
940	1	0.25	0	4
941	1	0.25	0	4
943	1	0.25	0	4
953	2	0.5	0	2
957	2	0.5	0	2
959	1	0.25	0	4
961	1	0.25	0	4
964	1	0.25	0	4
971	1	0.25	0	4
976	1	0.25	0	4
1010	1	0.5	0	4
1056	1	0.25	0	4
1118	1	0.5	0	4
1142	1	0.25	0	4
1144	1	0.25	0	4

1145	2	0.5	0	2		
1193	1	0.25	0	4		
1207	1	0.333333333333333			0	4
1213	1	0.333333333333333			0	4
1216	1	0.333333333333333			0	4
1229	1	0.333333333333333			0	4
1251	1	0.333333333333333			0	4
1256	1	0.333333333333333			0	4
1271	1	0.333333333333333			0	4
1296	1	0.25	0	4		
1314	1	0.25	0	4		
1317	1	0.25	0	4		
1380	1	0.25	0	4		
1383	1	0.25	0	4		
1386	1	0.25	0	4		
1389	1	0.25	0	4		
1438	1	0.5	0	4		
1449	1	0.333333333333333			0	4
1450	1	0.333333333333333			0	4
1480	1	0.5	0	4		
1535	1	0.5	0	4		
1538	1	0.5	0	4		
1541	1	0.5	0	4		
1622	1	0.25	0	4		
1629	1	0.25	0	4		
1635	1	0.25	0	4		
1648	1	0.25	0	4		
1658	1	0.25	0	4		
1659	1	0.25	0	4		
1661	1	0.25	0	4		
1662	1	0.25	0	4		
1683	1	0.5	0	4		
1694	1	0.25	0	4		
1695	1	0.25	0	4		
1697	1	0.5	0	4		
1704	1	0.25	0	4		
1729	1	0.25	0	4		
1732	1	0.25	0	4		
1738	1	0.25	0	4		
1759	17	4.25	0	0	<<<	
1760	2	0.5	0	2		
1788	1	0.25	0	4		
1789	3	0.75	0	2		
1803	2	1	0	2		
1816	1	0.5	0	4		
1861	1	0.25	0	4		
1866	2	0.75	0	2		
1875	1	0.5	0	4		
1879	1	0.5	0	4		
1882	1	0.25	0	4		
1898	1	0.25	0	4		
1899	1	0.25	0	4		
1900	1	0.5	0	4		
1903	1	0.25	0	4		
1909	1	0.5	0	4		
1910	1	0.5	0	4		
1924	1	0.5	0	4		
1926	1	0.5	0	4		

1934	1	0.5	0	4
1936	1	0.5	0	4
1937	1	0.5	0	4
2016	2	0.75	0	2
2027	1	0.5	0	4
2283	1	0.5	0	4

ptc-miR172d,e

category=0, cleavage_site=1755
query=ptc-miR172d,e, target=Potri.005G140700.1,
score=2.5, range=1744-1764, strand=1

```
target  5' CUGCAGCAUCAuCAGGAUUCU 3'
              :::::::::::.:::::.
query   3' UACGUCGUAGUAGUUCUAAGG 5'
```

>Potri.005G140700.1
#size=2188

196	1	0.25	0	4
231	1	0.142857142857143	0	4
247	1	0.142857142857143	0	4
304	1	0.142857142857143	0	4
476	2	0.285714285714286	0	2
516	1	0.25	0	4
536	1	0.142857142857143	0	4
677	1	0.142857142857143	0	4
694	1	0.142857142857143	0	4
695	1	0.142857142857143	0	4
701	1	0.25	0	4
702	1	0.142857142857143	0	4
788	2	0.2	0	2
812	1	0.142857142857143	0	4
819	1	0.142857142857143	0	4
821	1	0.142857142857143	0	4
834	1	0.142857142857143	0	4
835	1	0.142857142857143	0	4
852	1	0.142857142857143	0	4
855	1	0.142857142857143	0	4
867	1	0.142857142857143	0	4
876	1	0.142857142857143	0	4
881	1	0.142857142857143	0	4
900	1	0.25	0	4
907	1	0.25	0	4
928	1	0.25	0	4
955	1	0.142857142857143	0	4
974	1	0.25	0	4
976	1	0.142857142857143	0	4
979	1	0.142857142857143	0	4
987	2	0.285714285714286	0	2
999	1	0.142857142857143	0	4
1000	3	0.428571428571429	0	2
1001	2	0.285714285714286	0	2
1012	1	0.142857142857143	0	4
1038	1	0.25	0	4
1042	1	0.142857142857143	0	4
1045	1	0.142857142857143	0	4
1101	1	0.142857142857143	0	4
1122	1	0.142857142857143	0	4
1156	1	0.142857142857143	0	4

1164	1	0.142857142857143	0	4	
1337	1	0.25	0	4	
1426	1	0.142857142857143	0	4	
1440	1	0.142857142857143	0	4	
1454	1	0.142857142857143	0	4	
1473	1	0.142857142857143	0	4	
1475	1	0.142857142857143	0	4	
1522	1	0.25	0	4	
1558	1	0.25	0	4	
1572	1	0.25	0	4	
1583	1	0.25	0	4	
1701	1	0.142857142857143	0	4	
1713	1	0.142857142857143	0	4	
1755	32	5.33333333333333	0	0	<<<
1756	1	0.166666666666667	0	4	
1759	1	0.166666666666667	0	4	
1760	2	0.333333333333333	0	2	
1761	1	0.166666666666667	0	4	
1762	1	0.166666666666667	0	4	
1763	1	0.166666666666667	0	4	
1768	3	0.5	0	2	
1770	2	0.333333333333333	0	2	
1772	4	0.666666666666667	0	2	
1775	1	0.166666666666667	0	4	
1784	1	0.166666666666667	0	4	
1831	2	0.666666666666667	0	2	
1833	1	0.333333333333333	0	4	
1841	1	0.333333333333333	0	4	
1874	1	0.333333333333333	0	4	
1888	1	0.333333333333333	0	4	
1894	1	0.333333333333333	0	4	
1950	1	0.333333333333333	0	4	
1952	1	0.333333333333333	0	4	
2068	1	0.333333333333333	0	4	
2085	1	0.166666666666667	0	4	
2092	1	0.333333333333333	0	4	

---

category=2, cleavage_site=1479
query=ptc-miR172d,e, target=Potri.006G132400.1,
score=3, range=1468-1488, strand=1

```
target  5' CUGCAGCAUCAuCAGGAUUCG 3'
           ::::::::::::.:::::
query   3' UACGUCGUAGUAGUUCUAAGG 5'
```

>Potri.006G132400.1
#size=2157

11	1	0.5	0	4
27	2	1	0	2
66	1	0.5	0	4
87	1	0.5	0	4
151	1	0.5	0	4
156	1	0.5	0	4
209	1	0.25	0	4
217	1	0.25	0	4
221	1	0.25	0	4
234	1	0.5	0	4
240	1	0.5	0	4
268	1	0.25	0	4
282	1	0.5	0	4

296	1	0.5	0	4
309	1	0.5	0	4
332	1	0.5	0	4
336	1	0.5	0	4
339	1	0.5	0	4
341	1	0.5	0	4
344	1	0.5	0	4
347	1	0.5	0	4
351	1	0.5	0	4
382	1	0.5	0	4
387	2	1	0	2
388	1	0.5	0	4
390	1	0.5	0	4
394	4	2	0	2
398	1	0.5	0	4
399	2	1	0	2
405	1	0.5	0	4
408	1	0.5	0	4
413	2	0.5	0	2
414	3	0.75	0	2
415	2	1	0	2
416	9	4.25	0	2
417	10	4.75	0	2
418	3	1.5	0	2
419	1	0.5	0	4
423	2	1	0	2
424	1	0.5	0	4
426	1	0.5	0	4
427	1	0.5	0	4
428	1	0.5	0	4
429	1	0.5	0	4
430	1	0.5	0	4
431	1	0.5	0	4
434	1	0.5	0	4
435	2	1	0	2
436	2	1	0	2
439	1	0.5	0	4
442	1	0.5	0	4
444	1	0.5	0	4
445	3	1.5	0	2
446	2	1	0	2
449	1	0.5	0	4
451	1	0.5	0	4
456	1	0.5	0	4
457	1	0.5	0	4
458	3	1.5	0	2
472	2	1	0	2
474	1	0.5	0	4
476	1	0.5	0	4
485	1	0.5	0	4
494	3	1.5	0	2
496	1	0.5	0	4
498	2	1	0	2
500	2	1	0	2
502	1	0.5	0	4
504	1	0.5	0	4
508	1	0.5	0	4
513	1	0.5	0	4

517	1	0.5	0	4
538	2	1	0	2
544	1	0.5	0	4
545	3	1.5	0	2
546	6	3	0	2
547	4	2	0	2
548	22	11	0	0
549	4	2	0	2
550	4	2	0	2
551	1	0.5	0	4
553	3	1.5	0	2
554	2	1	0	2
556	4	2	0	2
558	2	1	0	2
567	1	0.25	0	4
576	1	0.25	0	4
577	1	0.25	0	4
583	1	0.25	0	4
591	1	0.25	0	4
592	2	0.5	0	2
599	2	0.5	0	2
608	2	0.5	0	2
612	1	0.25	0	4
613	1	0.25	0	4
614	2	0.5	0	2
621	1	0.25	0	4
624	1	0.25	0	4
628	2	0.5	0	2
638	3	0.75	0	2
641	1	0.25	0	4
642	1	0.25	0	4
648	2	0.5	0	2
651	1	0.25	0	4
660	1	0.25	0	4
661	1	0.25	0	4
663	1	0.25	0	4
673	2	0.5	0	2
677	2	0.5	0	2
679	1	0.25	0	4
681	1	0.25	0	4
684	1	0.25	0	4
691	1	0.25	0	4
696	1	0.25	0	4
739	1	0.5	0	4
743	1	0.5	0	4
776	1	0.25	0	4
786	1	0.5	0	4
789	1	0.5	0	4
794	1	0.5	0	4
820	1	0.5	0	4
822	1	0.5	0	4
823	1	0.5	0	4
825	1	0.5	0	4
827	2	1	0	2
835	1	0.5	0	4
836	1	0.5	0	4
848	1	0.5	0	4
862	1	0.25	0	4

864	1	0.25	0	4	
865	2	0.5	0	2	
873	1	0.5	0	4	
892	1	0.5	0	4	
900	1	0.5	0	4	
905	1	0.5	0	4	
913	1	0.25	0	4	
927	1	0.333333333333333	0	4	
933	1	0.333333333333333	0	4	
936	1	0.333333333333333	0	4	
949	1	0.333333333333333	0	4	
971	1	0.333333333333333	0	4	
976	1	0.333333333333333	0	4	
991	1	0.333333333333333	0	4	
1016	1	0.25	0	4	
1034	1	0.25	0	4	
1037	1	0.25	0	4	
1056	1	0.5	0	4	
1066	1	0.5	0	4	
1070	1	0.5	0	4	
1100	1	0.25	0	4	
1103	1	0.25	0	4	
1106	1	0.25	0	4	
1109	1	0.25	0	4	
1113	1	0.5	0	4	
1121	1	0.5	0	4	
1126	1	0.5	0	4	
1128	1	0.5	0	4	
1131	1	0.5	0	4	
1169	1	0.333333333333333	0	4	
1170	1	0.333333333333333	0	4	
1192	1	1	1	4	
1264	1	0.5	0	4	
1273	1	0.5	0	4	
1293	1	0.5	0	4	
1342	1	0.25	0	4	
1349	1	0.25	0	4	
1355	1	0.25	0	4	
1368	1	0.25	0	4	
1378	1	0.25	0	4	
1379	1	0.25	0	4	
1381	1	0.25	0	4	
1382	1	0.25	0	4	
1414	1	0.25	0	4	
1415	1	0.25	0	4	
1424	1	0.25	0	4	
1449	1	0.25	0	4	
1452	1	0.25	0	4	
1458	1	0.25	0	4	
1479	17	4.25	0	2	<<<
1480	2	0.5	0	2	
1504	1	0.5	0	4	
1506	1	0.5	0	4	
1508	1	0.25	0	4	
1509	4	1.25	0	2	
1515	1	0.5	0	4	
1533	1	0.5	0	4	
1540	1	0.5	0	4	

1542	1	0.5	0	4
1543	2	1	0	2
1544	1	0.5	0	4
1545	1	0.5	0	4
1552	2	1	0	2
1555	2	1	0	2
1556	1	0.5	0	4
1560	1	0.5	0	4
1563	1	0.5	0	4
1575	1	0.25	0	4
1580	1	0.25	0	4
1596	1	0.25	0	4
1605	1	0.5	0	4
1612	1	0.25	0	4
1613	1	0.25	0	4
1617	1	0.25	0	4
1636	1	0.5	0	4
1644	1	0.5	0	4
1652	3	1.5	0	2
1711	1	0.5	0	4
1717	1	0.5	0	4
1734	1	0.25	0	4
1737	1	0.5	0	4
1784	1	0.5	0	4
1806	2	1	0	2
1847	1	0.5	0	4
1866	1	0.5	0	4
1872	1	0.5	0	4
1914	1	0.5	0	4
1923	1	0.5	0	4

category=0, cleavage_site=2149
query=ptc-miR172d,e, target=Potri.007G046200.1,
score=2, range=2138-2158, strand=1

```
target  5' CUGCAGCAUCAuCAGGAUUCC 3'
             :::::::::::.::::::
query   3' UACGUCGUAGUAGUUCUAAGG 5'
```

>Potri.007G046200.1
#size=2909

266	1	0.333333333333333	0	4
366	1	0.333333333333333	0	4
414	2	0.666666666666667	0	2
420	1	0.333333333333333	0	4
459	1	0.333333333333333	0	4
546	1	0.333333333333333	0	4
597	1	0.333333333333333	0	4
599	1	0.333333333333333	0	4
631	1	0.142857142857143	0	4
647	1	0.142857142857143	0	4
696	1	0.333333333333333	0	4
704	1	0.142857142857143	0	4
834	1	0.333333333333333	0	4
853	1	0.333333333333333	0	4
876	2	0.285714285714286	0	3
936	1	0.142857142857143	0	4
986	1	0.333333333333333	0	4
1059	1	0.333333333333333	0	4
1063	1	0.333333333333333	0	4

1089	1	0.142857142857143	0	4
1106	1	0.142857142857143	0	4
1107	1	0.142857142857143	0	4
1114	1	0.142857142857143	0	4
1200	2	0.2	0	3
1224	1	0.142857142857143	0	4
1231	1	0.142857142857143	0	4
1233	1	0.142857142857143	0	4
1246	1	0.142857142857143	0	4
1247	1	0.142857142857143	0	4
1264	1	0.142857142857143	0	4
1267	1	0.142857142857143	0	4
1279	1	0.142857142857143	0	4
1288	1	0.142857142857143	0	4
1293	1	0.142857142857143	0	4
1352	1	0.333333333333333	0	4
1353	1	0.333333333333333	0	4
1367	1	0.142857142857143	0	4
1388	1	0.142857142857143	0	4
1391	1	0.142857142857143	0	4
1399	2	0.285714285714286	0	3
1411	1	0.142857142857143	0	4
1412	3	0.428571428571429	0	2
1413	2	0.285714285714286	0	3
1424	1	0.142857142857143	0	4
1454	1	0.142857142857143	0	4
1457	1	0.142857142857143	0	4
1475	1	0.333333333333333	0	4
1513	1	0.142857142857143	0	4
1526	1	0.333333333333333	0	4
1534	1	0.142857142857143	0	4
1568	1	0.142857142857143	0	4
1576	1	0.142857142857143	0	4
1597	1	0.5	0	4
1686	1	0.333333333333333	0	4
1713	2	0.666666666666667	0	2
1718	1	0.333333333333333	0	4
1719	1	0.333333333333333	0	4
1729	1	0.333333333333333	0	4
1771	2	0.666666666666667	0	2
1789	1	0.333333333333333	0	4
1794	1	0.333333333333333	0	4
1807	1	0.333333333333333	0	4
1808	3	1	0	2
1812	1	0.333333333333333	0	4
1820	1	0.142857142857143	0	4
1834	1	0.142857142857143	0	4
1848	1	0.142857142857143	0	4
1867	1	0.142857142857143	0	4
1869	1	0.142857142857143	0	4
1919	1	0.333333333333333	0	4
1941	1	0.333333333333333	0	4
1956	1	0.333333333333333	0	4
1979	1	0.333333333333333	0	4
1990	1	0.5	0	4
2030	1	0.333333333333333	0	4
2075	1	0.333333333333333	0	4
2095	1	0.142857142857143	0	4

2107	1	0.142857142857143	0	4	
2149	32	5.33333333333333	0	0	<<<
2150	1	0.166666666666667	0	4	
2153	1	0.166666666666667	0	4	
2154	3	0.666666666666667	0	2	
2155	1	0.166666666666667	0	4	
2156	1	0.166666666666667	0	4	
2157	1	0.166666666666667	0	4	
2162	3	0.5	0	2	
2164	2	0.333333333333333	0	2	
2166	4	0.666666666666667	0	2	
2169	1	0.166666666666667	0	4	
2172	1	0.333333333333333	0	4	
2174	1	0.333333333333333	0	4	
2178	1	0.166666666666667	0	4	
2179	1	0.333333333333333	0	4	
2182	1	0.333333333333333	0	4	
2184	1	0.333333333333333	0	4	
2192	1	0.333333333333333	0	4	
2193	1	0.333333333333333	0	4	
2198	1	0.333333333333333	0	4	
2201	2	0.666666666666667	0	2	
2202	1	0.333333333333333	0	4	
2206	1	0.333333333333333	0	4	
2210	1	0.333333333333333	0	4	
2211	2	0.666666666666667	0	2	
2216	1	0.333333333333333	0	4	
2226	1	0.333333333333333	0	4	
2231	1	0.333333333333333	0	4	
2235	1	0.333333333333333	0	4	
2242	3	1	0	2	
2243	3	1	0	2	
2247	1	0.333333333333333	0	4	
2301	1	0.333333333333333	0	4	
2304	1	0.333333333333333	0	4	
2305	3	1	0	2	
2306	1	0.333333333333333	0	4	
2310	2	0.666666666666667	0	2	
2311	2	0.666666666666667	0	2	
2313	2	0.666666666666667	0	2	
2317	1	0.333333333333333	0	4	
2319	1	0.333333333333333	0	4	
2320	2	0.666666666666667	0	2	
2323	1	0.333333333333333	0	4	
2326	1	0.333333333333333	0	4	
2328	1	0.333333333333333	0	4	
2330	1	0.333333333333333	0	4	
2333	1	0.333333333333333	0	4	
2352	1	0.333333333333333	0	4	
2359	1	0.333333333333333	0	4	
2380	1	0.333333333333333	0	4	
2381	1	0.333333333333333	0	4	
2452	1	0.333333333333333	0	4	
2470	1	0.333333333333333	0	4	
2502	1	0.166666666666667	0	4	
2631	1	0.333333333333333	0	4	
2833	2	0.666666666666667	0	2	

category=0, cleavage_site=1958
query=ptc-miR172d,e, target=Potri.008G045300.1,
score=2.5, range=1947-1967, strand=1

```
target  5' UUGCAGCAUCAuCAGGAUUCU 3'
             ::::::::::::.:::::.
query   3' UACGUCGUAGUAGUUCUAAGG 5'
```

>Potri.008G045300.1
#size=2324

490	1	0.2	0	4
494	1	0.2	0	4
568	2	0.4	0	2
578	2	0.25	0	2
579	1	0.125	0	4
616	1	0.2	0	4
638	1	0.2	0	4
667	1	0.2	0	4
699	1	0.2	0	4
717	1	0.1	0	4
800	1	0.1	0	4
870	1	0.2	0	4
935	1	0.2	0	4
937	1	0.2	0	4
940	1	0.2	0	4
942	1	0.2	0	4
945	1	0.2	0	4
949	1	0.2	0	4
954	1	0.2	0	4
964	1	0.1	0	4
966	1	0.1	0	4
968	1	0.1	0	4
969	1	0.1	0	4
995	1	0.2	0	4
1002	1	0.2	0	4
1018	1	0.2	0	4
1024	1	0.1	0	4
1025	1	0.1	0	4
1029	1	0.1	0	4
1030	1	0.1	0	4
1035	1	0.1	0	4
1036	1	0.1	0	4
1045	1	0.1	0	4
1048	1	0.1	0	4
1049	1	0.1	0	4
1050	1	0.1	0	4
1051	1	0.1	0	4
1065	1	0.1	0	4
1068	1	0.1	0	4
1071	1	0.1	0	4
1073	1	0.1	0	4
1074	1	0.1	0	4
1075	1	0.1	0	4
1076	1	0.1	0	4
1078	1	0.1	0	4
1080	2	0.2	0	2
1085	1	0.1	0	4
1086	2	0.2	0	2
1090	1	0.1	0	4

1092	2	0.3	0	2		
1095	1	0.1	0	4		
1097	1	0.1	0	4		
1098	1	0.1	0	4		
1150	1	0.111111111111111			0	4
1164	1	0.2	0	4		
1169	1	0.2	0	4		
1170	1	0.2	0	4		
1199	1	0.2	0	4		
1201	1	0.111111111111111			0	4
1222	1	0.2	0	4		
1233	1	0.2	0	4		
1268	1	0.111111111111111			0	4
1287	1	0.111111111111111			0	4
1325	1	0.111111111111111			0	4
1339	1	0.111111111111111			0	4
1340	1	0.111111111111111			0	4
1354	1	0.111111111111111			0	4
1370	1	0.111111111111111			0	4
1372	1	0.111111111111111			0	4
1394	2	0.222222222222222			0	2
1533	1	0.25	0	4		
1544	1	0.25	0	4		
1570	1	0.25	0	4		
1586	1	0.125	0	4		
1627	1	0.25	0	4		
1644	1	0.25	0	4		
1816	1	0.25	0	4		
1901	2	0.222222222222222			0	2
1929	1	0.111111111111111			0	4
1958	32	8	0	0	<<<	
1969	1	0.25	0	4		
1982	1	0.25	0	4		
1986	1	0.25	0	4		
1989	2	0.222222222222222			0	2
1992	1	0.25	0	4		
1994	3	0.75	0	2		
1998	1	0.25	0	4		
2001	1	0.25	0	4		
2009	1	0.25	0	4		
2010	1	0.25	0	4		
2015	1	0.25	0	4		
2017	1	0.25	0	4		
2020	1	0.25	0	4		
2021	1	0.25	0	4		
2024	1	0.25	0	4		
2041	1	0.25	0	4		
2045	1	0.25	0	4		
2052	1	0.25	0	4		
2055	1	0.25	0	4		
2056	1	0.25	0	4		
2058	1	0.25	0	4		
2060	1	0.25	0	4		
2061	1	0.25	0	4		
2066	1	0.25	0	4		
2068	2	0.5	0	2		
2198	1	0.25	0	4		
2224	1	0.25	0	4		

2233	1	0.25	0	4

category=0, cleavage_site=1779
query=ptc-miR172d,e, target=Potri.010G216200.1,
score=2.5, range=1768-1788, strand=1

```
target  5' UUGCAGCAUCAuCAGGAUUCU 3'
            :::::::::::::.:::::.
query   3' UACGUCGUAGUAGUUCUAAGG 5'
```

>Potri.010G216200.1
#size=2200

185	1	0.333333333333333	0	4
227	1	0.333333333333333	0	4
287	1	0.333333333333333	0	4
306	1	0.333333333333333	0	4
356	1	0.333333333333333	0	4
402	2	0.25	0	2
403	1	0.125	0	4
496	1	0.2	0	4
526	1	0.2	0	4
538	1	0.1	0	4
580	1	0.2	0	4
621	1	0.1	0	4
684	1	0.2	0	4
689	1	0.2	0	4
691	2	0.4	0	2
711	1	0.2	0	4
729	1	0.2	0	4
734	1	0.2	0	4
755	1	0.2	0	4
763	1	0.2	0	4
769	2	0.4	0	2
770	1	0.2	0	4
776	1	0.2	0	4
785	1	0.1	0	4
787	1	0.1	0	4
789	1	0.1	0	4
790	1	0.1	0	4
845	1	0.1	0	4
846	1	0.1	0	4
850	1	0.1	0	4
851	1	0.1	0	4
856	1	0.1	0	4
857	1	0.1	0	4
866	1	0.1	0	4
869	1	0.1	0	4
870	1	0.1	0	4
871	1	0.1	0	4
872	1	0.1	0	4
886	1	0.1	0	4
889	1	0.1	0	4
892	1	0.1	0	4
894	1	0.1	0	4
895	1	0.1	0	4
896	1	0.1	0	4
897	1	0.1	0	4
899	1	0.1	0	4
901	2	0.2	0	2
906	1	0.1	0	4

907	2	0.2	0	2	
911	1	0.1	0	4	
913	1	0.1	0	4	
916	1	0.1	0	4	
918	1	0.1	0	4	
919	1	0.1	0	4	
971	1	0.111111111111111	0	4	
1022	1	0.111111111111111	0	4	
1029	1	0.25	0	4	
1037	1	0.25	0	4	
1038	1	0.25	0	4	
1039	1	0.25	0	4	
1040	2	0.5	0	2	
1041	1	0.25	0	4	
1042	1	0.25	0	4	
1044	1	0.25	0	4	
1047	1	0.25	0	4	
1051	1	0.25	0	4	
1054	1	0.25	0	4	
1055	1	0.25	0	4	
1058	1	0.25	0	4	
1064	2	0.5	0	2	
1071	2	0.5	0	2	
1073	1	0.25	0	4	
1089	1	0.111111111111111	0	4	
1108	1	0.111111111111111	0	4	
1119	1	0.25	0	4	
1122	1	0.25	0	4	
1135	1	0.25	0	4	
1146	1	0.111111111111111	0	4	
1160	1	0.111111111111111	0	4	
1161	1	0.111111111111111	0	4	
1175	1	0.111111111111111	0	4	
1191	1	0.111111111111111	0	4	
1193	1	0.111111111111111	0	4	
1198	1	0.25	0	4	
1215	2	0.222222222222222	0	2	
1304	1	0.25	0	4	
1318	1	0.25	0	4	
1323	1	0.25	0	4	
1334	1	0.25	0	4	
1407	1	0.125	0	4	
1423	1	0.25	0	4	
1528	2	0.666666666666667	0	2	
1591	1	0.2	0	4	
1607	1	0.2	0	4	
1639	1	0.2	0	4	
1663	1	0.2	0	4	
1677	2	0.4	0	2	
1680	2	0.4	0	2	
1705	1	0.2	0	4	
1722	2	0.222222222222222	0	2	
1750	1	0.111111111111111	0	4	
1777	1	0.2	0	4	
1779	37	7.4	0	0	<<<
1786	1	0.2	0	4	
1798	2	0.4	0	2	
1799	1	0.2	0	4	

1800	1	0.2	0	4
1801	1	0.2	0	4
1806	2	0.4	0	2
1807	3	0.422222222222222	0	2
1809	1	0.2	0	4
1810	1	0.2	0	4
1816	1	0.2	0	4
1827	1	0.2	0	4
1830	1	0.2	0	4
1873	1	0.2	0	4
1880	3	0.6	0	2
1882	1	0.2	0	4
1890	1	0.2	0	4
1895	1	0.2	0	4
1906	1	0.2	0	4
1913	2	0.4	0	2
1923	1	0.2	0	4
1924	1	0.2	0	4
1944	1	0.2	0	4
1946	1	0.2	0	4
2001	1	0.2	0	4
2068	1	0.2	0	4
2076	1	0.2	0	4
2077	1	0.2	0	4

category=0, cleavage_site=1759
query=ptc-miR172d,e, target=Potri.016G084500.1,
score=3, range=1748-1768, strand=1

```
target  5' CUGCAGCAUCAuCAGGAUUCG 3'
            :::::::::::::.:::::
query   3' UACGUCGUAGUAGUUCUAAGG 5'
```

>Potri.016G084500.1
#size=2370

62	1	0.25	0	4
195	1	0.5	0	4
418	1	0.5	0	4
484	1	0.5	0	4
495	1	0.25	0	4
503	1	0.25	0	4
507	1	0.25	0	4
513	1	0.5	0	4
554	1	0.25	0	4
569	1	0.5	0	4
583	1	0.5	0	4
655	1	0.5	0	4
676	1	0.5	0	4
685	1	0.5	0	4
690	3	1	0	2
691	3	0.75	0	2
693	3	1.25	0	2
694	1	0.25	0	4
695	1	0.5	0	4
696	1	0.5	0	4
704	1	0.5	0	4
710	1	0.5	0	4
720	1	0.5	0	4
724	1	0.5	0	4
728	3	1.5	0	2

730	1	0.5	0	4
733	1	0.5	0	4
734	2	1	0	2
735	1	0.5	0	4
738	1	0.5	0	4
740	2	1	0	2
750	1	0.5	0	4
766	1	0.5	0	4
774	1	0.5	0	4
817	1	0.5	0	4
824	1	0.5	0	4
833	1	0.5	0	4
834	1	0.5	0	4
847	1	0.25	0	4
856	1	0.25	0	4
857	1	0.25	0	4
863	1	0.25	0	4
871	1	0.25	0	4
872	2	0.5	0	2
879	2	0.5	0	2
888	2	0.5	0	2
892	1	0.25	0	4
893	1	0.25	0	4
894	2	0.5	0	2
901	1	0.25	0	4
904	1	0.25	0	4
908	2	0.5	0	2
918	3	0.75	0	2
921	1	0.25	0	4
922	1	0.25	0	4
928	2	0.5	0	2
931	1	0.25	0	4
940	1	0.25	0	4
941	1	0.25	0	4
943	1	0.25	0	4
953	2	0.5	0	2
957	2	0.5	0	2
959	1	0.25	0	4
961	1	0.25	0	4
964	1	0.25	0	4
971	1	0.25	0	4
976	1	0.25	0	4
1010	1	0.5	0	4
1056	1	0.25	0	4
1118	1	0.5	0	4
1142	1	0.25	0	4
1144	1	0.25	0	4
1145	2	0.5	0	2
1193	1	0.25	0	4
1207	1	0.333333333333333	0	4
1213	1	0.333333333333333	0	4
1216	1	0.333333333333333	0	4
1229	1	0.333333333333333	0	4
1251	1	0.333333333333333	0	4
1256	1	0.333333333333333	0	4
1271	1	0.333333333333333	0	4
1296	1	0.25	0	4
1314	1	0.25	0	4

1317	1	0.25	0	4		
1380	1	0.25	0	4		
1383	1	0.25	0	4		
1386	1	0.25	0	4		
1389	1	0.25	0	4		
1438	1	0.5	0	4		
1449	1	0.333333333333333			0	4
1450	1	0.333333333333333			0	4
1480	1	0.5	0	4		
1535	1	0.5	0	4		
1538	1	0.5	0	4		
1541	1	0.5	0	4		
1622	1	0.25	0	4		
1629	1	0.25	0	4		
1635	1	0.25	0	4		
1648	1	0.25	0	4		
1658	1	0.25	0	4		
1659	1	0.25	0	4		
1661	1	0.25	0	4		
1662	1	0.25	0	4		
1683	1	0.5	0	4		
1694	1	0.25	0	4		
1695	1	0.25	0	4		
1697	1	0.5	0	4		
1704	1	0.25	0	4		
1729	1	0.25	0	4		
1732	1	0.25	0	4		
1738	1	0.25	0	4		
1759	17	4.25	0	0	<<<	
1760	2	0.5	0	2		
1788	1	0.25	0	4		
1789	3	0.75	0	2		
1803	2	1	0	2		
1816	1	0.5	0	4		
1861	1	0.25	0	4		
1866	2	0.75	0	2		
1875	1	0.5	0	4		
1879	1	0.5	0	4		
1882	1	0.25	0	4		
1898	1	0.25	0	4		
1899	1	0.25	0	4		
1900	1	0.5	0	4		
1903	1	0.25	0	4		
1909	1	0.5	0	4		
1910	1	0.5	0	4		
1924	1	0.5	0	4		
1926	1	0.5	0	4		
1934	1	0.5	0	4		
1936	1	0.5	0	4		
1937	1	0.5	0	4		
2016	2	0.75	0	2		
2027	1	0.5	0	4		
2283	1	0.5	0	4		

ptc-miR172g,h

category=0, cleavage_site=1755
query=ptc-miR172g,h, target=Potri.005G140700.1,
score=1.5, range=1744-1764, strand=1

```
target  5' CUGCAGCAUCAuCAGGAUUCU 3'
            ::::::::::::::.:::::.
query   3' GACGUCGUAGUAGUUCUAAGG 5'
```

>Potri.005G140700.1
#size=2188

196	1	0.25	0	4
231	1	0.142857142857143	0	4
247	1	0.142857142857143	0	4
304	1	0.142857142857143	0	4
476	2	0.285714285714286	0	2
516	1	0.25	0	4
536	1	0.142857142857143	0	4
677	1	0.142857142857143	0	4
694	1	0.142857142857143	0	4
695	1	0.142857142857143	0	4
701	1	0.25	0	4
702	1	0.142857142857143	0	4
788	2	0.2	0	2
812	1	0.142857142857143	0	4
819	1	0.142857142857143	0	4
821	1	0.142857142857143	0	4
834	1	0.142857142857143	0	4
835	1	0.142857142857143	0	4
852	1	0.142857142857143	0	4
855	1	0.142857142857143	0	4
867	1	0.142857142857143	0	4
876	1	0.142857142857143	0	4
881	1	0.142857142857143	0	4
900	1	0.25	0	4
907	1	0.25	0	4
928	1	0.25	0	4
955	1	0.142857142857143	0	4
974	1	0.25	0	4
976	1	0.142857142857143	0	4
979	1	0.142857142857143	0	4
987	2	0.285714285714286	0	2
999	1	0.142857142857143	0	4
1000	3	0.428571428571429	0	2
1001	2	0.285714285714286	0	2
1012	1	0.142857142857143	0	4
1038	1	0.25	0	4
1042	1	0.142857142857143	0	4
1045	1	0.142857142857143	0	4
1101	1	0.142857142857143	0	4
1122	1	0.142857142857143	0	4
1156	1	0.142857142857143	0	4
1164	1	0.142857142857143	0	4
1337	1	0.25	0	4
1426	1	0.142857142857143	0	4
1440	1	0.142857142857143	0	4
1454	1	0.142857142857143	0	4
1473	1	0.142857142857143	0	4
1475	1	0.142857142857143	0	4

1522	1	0.25	0	4	
1558	1	0.25	0	4	
1572	1	0.25	0	4	
1583	1	0.25	0	4	
1701	1	0.142857142857143	0	4	
1713	1	0.142857142857143	0	4	
1755	32	5.33333333333333	0	0	<<<
1756	1	0.166666666666667	0	4	
1759	1	0.166666666666667	0	4	
1760	2	0.333333333333333	0	2	
1761	1	0.166666666666667	0	4	
1762	1	0.166666666666667	0	4	
1763	1	0.166666666666667	0	4	
1768	3	0.5	0	2	
1770	2	0.333333333333333	0	2	
1772	4	0.666666666666667	0	2	
1775	1	0.166666666666667	0	4	
1784	1	0.166666666666667	0	4	
1831	2	0.666666666666667	0	2	
1833	1	0.333333333333333	0	4	
1841	1	0.333333333333333	0	4	
1874	1	0.333333333333333	0	4	
1888	1	0.333333333333333	0	4	
1894	1	0.333333333333333	0	4	
1950	1	0.333333333333333	0	4	
1952	1	0.333333333333333	0	4	
2068	1	0.333333333333333	0	4	
2085	1	0.166666666666667	0	4	
2092	1	0.333333333333333	0	4	

---

category=2, cleavage_site=1479
query=ptc-miR172g,h, target=Potri.006G132400.1,
score=2, range=1468-1488, strand=1

```
target  5' CUGCAGCAUCAuCAGGAUUCG 3'
           ::::::::::::.:::::
query   3' GACGUCGUAGUAGUUCUAAGG 5'
```

>Potri.006G132400.1
#size=2157

11	1	0.5	0	4
27	2	1	0	2
66	1	0.5	0	4
87	1	0.5	0	4
151	1	0.5	0	4
156	1	0.5	0	4
209	1	0.25	0	4
217	1	0.25	0	4
221	1	0.25	0	4
234	1	0.5	0	4
240	1	0.5	0	4
268	1	0.25	0	4
282	1	0.5	0	4
296	1	0.5	0	4
309	1	0.5	0	4
332	1	0.5	0	4
336	1	0.5	0	4
339	1	0.5	0	4
341	1	0.5	0	4
344	1	0.5	0	4

347	1	0.5	0	4
351	1	0.5	0	4
382	1	0.5	0	4
387	2	1	0	2
388	1	0.5	0	4
390	1	0.5	0	4
394	4	2	0	2
398	1	0.5	0	4
399	2	1	0	2
405	1	0.5	0	4
408	1	0.5	0	4
413	2	0.5	0	2
414	3	0.75	0	2
415	2	1	0	2
416	9	4.25	0	2
417	10	4.75	0	2
418	3	1.5	0	2
419	1	0.5	0	4
423	2	1	0	2
424	1	0.5	0	4
426	1	0.5	0	4
427	1	0.5	0	4
428	1	0.5	0	4
429	1	0.5	0	4
430	1	0.5	0	4
431	1	0.5	0	4
434	1	0.5	0	4
435	2	1	0	2
436	2	1	0	2
439	1	0.5	0	4
442	1	0.5	0	4
444	1	0.5	0	4
445	3	1.5	0	2
446	2	1	0	2
449	1	0.5	0	4
451	1	0.5	0	4
456	1	0.5	0	4
457	1	0.5	0	4
458	3	1.5	0	2
472	2	1	0	2
474	1	0.5	0	4
476	1	0.5	0	4
485	1	0.5	0	4
494	3	1.5	0	2
496	1	0.5	0	4
498	2	1	0	2
500	2	1	0	2
502	1	0.5	0	4
504	1	0.5	0	4
508	1	0.5	0	4
513	1	0.5	0	4
517	1	0.5	0	4
538	2	1	0	2
544	1	0.5	0	4
545	3	1.5	0	2
546	6	3	0	2
547	4	2	0	2
548	22	11	0	0

549	4	2	0	2
550	4	2	0	2
551	1	0.5	0	4
553	3	1.5	0	2
554	2	1	0	2
556	4	2	0	2
558	2	1	0	2
567	1	0.25	0	4
576	1	0.25	0	4
577	1	0.25	0	4
583	1	0.25	0	4
591	1	0.25	0	4
592	2	0.5	0	2
599	2	0.5	0	2
608	2	0.5	0	2
612	1	0.25	0	4
613	1	0.25	0	4
614	2	0.5	0	2
621	1	0.25	0	4
624	1	0.25	0	4
628	2	0.5	0	2
638	3	0.75	0	2
641	1	0.25	0	4
642	1	0.25	0	4
648	2	0.5	0	2
651	1	0.25	0	4
660	1	0.25	0	4
661	1	0.25	0	4
663	1	0.25	0	4
673	2	0.5	0	2
677	2	0.5	0	2
679	1	0.25	0	4
681	1	0.25	0	4
684	1	0.25	0	4
691	1	0.25	0	4
696	1	0.25	0	4
739	1	0.5	0	4
743	1	0.5	0	4
776	1	0.25	0	4
786	1	0.5	0	4
789	1	0.5	0	4
794	1	0.5	0	4
820	1	0.5	0	4
822	1	0.5	0	4
823	1	0.5	0	4
825	1	0.5	0	4
827	2	1	0	2
835	1	0.5	0	4
836	1	0.5	0	4
848	1	0.5	0	4
862	1	0.25	0	4
864	1	0.25	0	4
865	2	0.5	0	2
873	1	0.5	0	4
892	1	0.5	0	4
900	1	0.5	0	4
905	1	0.5	0	4
913	1	0.25	0	4

927	1	0.333333333333333	0	4	
933	1	0.333333333333333	0	4	
936	1	0.333333333333333	0	4	
949	1	0.333333333333333	0	4	
971	1	0.333333333333333	0	4	
976	1	0.333333333333333	0	4	
991	1	0.333333333333333	0	4	
1016	1	0.25	0	4	
1034	1	0.25	0	4	
1037	1	0.25	0	4	
1056	1	0.5	0	4	
1066	1	0.5	0	4	
1070	1	0.5	0	4	
1100	1	0.25	0	4	
1103	1	0.25	0	4	
1106	1	0.25	0	4	
1109	1	0.25	0	4	
1113	1	0.5	0	4	
1121	1	0.5	0	4	
1126	1	0.5	0	4	
1128	1	0.5	0	4	
1131	1	0.5	0	4	
1169	1	0.333333333333333	0	4	
1170	1	0.333333333333333	0	4	
1192	1	1	1	4	
1264	1	0.5	0	4	
1273	1	0.5	0	4	
1293	1	0.5	0	4	
1342	1	0.25	0	4	
1349	1	0.25	0	4	
1355	1	0.25	0	4	
1368	1	0.25	0	4	
1378	1	0.25	0	4	
1379	1	0.25	0	4	
1381	1	0.25	0	4	
1382	1	0.25	0	4	
1414	1	0.25	0	4	
1415	1	0.25	0	4	
1424	1	0.25	0	4	
1449	1	0.25	0	4	
1452	1	0.25	0	4	
1458	1	0.25	0	4	
1479	17	4.25	0	2	<<<
1480	2	0.5	0	2	
1504	1	0.5	0	4	
1506	1	0.5	0	4	
1508	1	0.25	0	4	
1509	4	1.25	0	2	
1515	1	0.5	0	4	
1533	1	0.5	0	4	
1540	1	0.5	0	4	
1542	1	0.5	0	4	
1543	2	1	0	2	
1544	1	0.5	0	4	
1545	1	0.5	0	4	
1552	2	1	0	2	
1555	2	1	0	2	
1556	1	0.5	0	4	

1560	1	0.5	0	4
1563	1	0.5	0	4
1575	1	0.25	0	4
1580	1	0.25	0	4
1596	1	0.25	0	4
1605	1	0.5	0	4
1612	1	0.25	0	4
1613	1	0.25	0	4
1617	1	0.25	0	4
1636	1	0.5	0	4
1644	1	0.5	0	4
1652	3	1.5	0	2
1711	1	0.5	0	4
1717	1	0.5	0	4
1734	1	0.25	0	4
1737	1	0.5	0	4
1784	1	0.5	0	4
1806	2	1	0	2
1847	1	0.5	0	4
1866	1	0.5	0	4
1872	1	0.5	0	4
1914	1	0.5	0	4
1923	1	0.5	0	4

category=0, cleavage_site=2149
query=ptc-miR172g,h, target=Potri.007G046200.1,
score=1, range=2138-2158, strand=1

```
target  5' CUGCAGCAUCAuCAGGAUUCC 3'
           ::::::::::::.::::::
query   3' GACGUCGUAGUAGUUCUAAGG 5'
```

>Potri.007G046200.1
#size=2909

266	1	0.333333333333333	0	4
366	1	0.333333333333333	0	4
414	2	0.666666666666667	0	2
420	1	0.333333333333333	0	4
459	1	0.333333333333333	0	4
546	1	0.333333333333333	0	4
597	1	0.333333333333333	0	4
599	1	0.333333333333333	0	4
631	1	0.142857142857143	0	4
647	1	0.142857142857143	0	4
696	1	0.333333333333333	0	4
704	1	0.142857142857143	0	4
834	1	0.333333333333333	0	4
853	1	0.333333333333333	0	4
876	2	0.285714285714286	0	3
936	1	0.142857142857143	0	4
986	1	0.333333333333333	0	4
1059	1	0.333333333333333	0	4
1063	1	0.333333333333333	0	4
1089	1	0.142857142857143	0	4
1106	1	0.142857142857143	0	4
1107	1	0.142857142857143	0	4
1114	1	0.142857142857143	0	4
1200	2	0.2	0	3
1224	1	0.142857142857143	0	4
1231	1	0.142857142857143	0	4

1233	1	0.142857142857143	0	4	
1246	1	0.142857142857143	0	4	
1247	1	0.142857142857143	0	4	
1264	1	0.142857142857143	0	4	
1267	1	0.142857142857143	0	4	
1279	1	0.142857142857143	0	4	
1288	1	0.142857142857143	0	4	
1293	1	0.142857142857143	0	4	
1352	1	0.333333333333333	0	4	
1353	1	0.333333333333333	0	4	
1367	1	0.142857142857143	0	4	
1388	1	0.142857142857143	0	4	
1391	1	0.142857142857143	0	4	
1399	2	0.285714285714286	0	3	
1411	1	0.142857142857143	0	4	
1412	3	0.428571428571429	0	2	
1413	2	0.285714285714286	0	3	
1424	1	0.142857142857143	0	4	
1454	1	0.142857142857143	0	4	
1457	1	0.142857142857143	0	4	
1475	1	0.333333333333333	0	4	
1513	1	0.142857142857143	0	4	
1526	1	0.333333333333333	0	4	
1534	1	0.142857142857143	0	4	
1568	1	0.142857142857143	0	4	
1576	1	0.142857142857143	0	4	
1597	1	0.5	0	4	
1686	1	0.333333333333333	0	4	
1713	2	0.666666666666667	0	2	
1718	1	0.333333333333333	0	4	
1719	1	0.333333333333333	0	4	
1729	1	0.333333333333333	0	4	
1771	2	0.666666666666667	0	2	
1789	1	0.333333333333333	0	4	
1794	1	0.333333333333333	0	4	
1807	1	0.333333333333333	0	4	
1808	3	1	0	2	
1812	1	0.333333333333333	0	4	
1820	1	0.142857142857143	0	4	
1834	1	0.142857142857143	0	4	
1848	1	0.142857142857143	0	4	
1867	1	0.142857142857143	0	4	
1869	1	0.142857142857143	0	4	
1919	1	0.333333333333333	0	4	
1941	1	0.333333333333333	0	4	
1956	1	0.333333333333333	0	4	
1979	1	0.333333333333333	0	4	
1990	1	0.5	0	4	
2030	1	0.333333333333333	0	4	
2075	1	0.333333333333333	0	4	
2095	1	0.142857142857143	0	4	
2107	1	0.142857142857143	0	4	
2149	32	5.33333333333333	0	0	<<<
2150	1	0.166666666666667	0	4	
2153	1	0.166666666666667	0	4	
2154	3	0.666666666666667	0	2	
2155	1	0.166666666666667	0	4	
2156	1	0.166666666666667	0	4	

2157	1	0.166666666666667	0	4
2162	3	0.5	0	2
2164	2	0.333333333333333	0	2
2166	4	0.666666666666667	0	2
2169	1	0.166666666666667	0	4
2172	1	0.333333333333333	0	4
2174	1	0.333333333333333	0	4
2178	1	0.166666666666667	0	4
2179	1	0.333333333333333	0	4
2182	1	0.333333333333333	0	4
2184	1	0.333333333333333	0	4
2192	1	0.333333333333333	0	4
2193	1	0.333333333333333	0	4
2198	1	0.333333333333333	0	4
2201	2	0.666666666666667	0	2
2202	1	0.333333333333333	0	4
2206	1	0.333333333333333	0	4
2210	1	0.333333333333333	0	4
2211	2	0.666666666666667	0	2
2216	1	0.333333333333333	0	4
2226	1	0.333333333333333	0	4
2231	1	0.333333333333333	0	4
2235	1	0.333333333333333	0	4
2242	3	1	0	2
2243	3	1	0	2
2247	1	0.333333333333333	0	4
2301	1	0.333333333333333	0	4
2304	1	0.333333333333333	0	4
2305	3	1	0	2
2306	1	0.333333333333333	0	4
2310	2	0.666666666666667	0	2
2311	2	0.666666666666667	0	2
2313	2	0.666666666666667	0	2
2317	1	0.333333333333333	0	4
2319	1	0.333333333333333	0	4
2320	2	0.666666666666667	0	2
2323	1	0.333333333333333	0	4
2326	1	0.333333333333333	0	4
2328	1	0.333333333333333	0	4
2330	1	0.333333333333333	0	4
2333	1	0.333333333333333	0	4
2352	1	0.333333333333333	0	4
2359	1	0.333333333333333	0	4
2380	1	0.333333333333333	0	4
2381	1	0.333333333333333	0	4
2452	1	0.333333333333333	0	4
2470	1	0.333333333333333	0	4
2502	1	0.166666666666667	0	4
2631	1	0.333333333333333	0	4
2833	2	0.666666666666667	0	2

---

category=0, cleavage_site=1958
query=ptc-miR172g,h, target=Potri.008G045300.1,
score=2, range=1947-1967, strand=1

```
target  5' UUGCAGCAUCAuCAGGAUUCU 3'
           .:::::::::::::.:::::.
query   3' GACGUCGUAGUAGUUCUAAGG 5'
```

>Potri.008G045300.1

#size=2324

490	1	0.2	0	4
494	1	0.2	0	4
568	2	0.4	0	2
578	2	0.25	0	2
579	1	0.125	0	4
616	1	0.2	0	4
638	1	0.2	0	4
667	1	0.2	0	4
699	1	0.2	0	4
717	1	0.1	0	4
800	1	0.1	0	4
870	1	0.2	0	4
935	1	0.2	0	4
937	1	0.2	0	4
940	1	0.2	0	4
942	1	0.2	0	4
945	1	0.2	0	4
949	1	0.2	0	4
954	1	0.2	0	4
964	1	0.1	0	4
966	1	0.1	0	4
968	1	0.1	0	4
969	1	0.1	0	4
995	1	0.2	0	4
1002	1	0.2	0	4
1018	1	0.2	0	4
1024	1	0.1	0	4
1025	1	0.1	0	4
1029	1	0.1	0	4
1030	1	0.1	0	4
1035	1	0.1	0	4
1036	1	0.1	0	4
1045	1	0.1	0	4
1048	1	0.1	0	4
1049	1	0.1	0	4
1050	1	0.1	0	4
1051	1	0.1	0	4
1065	1	0.1	0	4
1068	1	0.1	0	4
1071	1	0.1	0	4
1073	1	0.1	0	4
1074	1	0.1	0	4
1075	1	0.1	0	4
1076	1	0.1	0	4
1078	1	0.1	0	4
1080	2	0.2	0	2
1085	1	0.1	0	4
1086	2	0.2	0	2
1090	1	0.1	0	4
1092	2	0.3	0	2
1095	1	0.1	0	4
1097	1	0.1	0	4
1098	1	0.1	0	4
1150	1	0.111111111111111	0	4
1164	1	0.2	0	4
1169	1	0.2	0	4
1170	1	0.2	0	4

1199	1	0.2	0	4	
1201	1	0.111111111111111	0	4	
1222	1	0.2	0	4	
1233	1	0.2	0	4	
1268	1	0.111111111111111	0	4	
1287	1	0.111111111111111	0	4	
1325	1	0.111111111111111	0	4	
1339	1	0.111111111111111	0	4	
1340	1	0.111111111111111	0	4	
1354	1	0.111111111111111	0	4	
1370	1	0.111111111111111	0	4	
1372	1	0.111111111111111	0	4	
1394	2	0.222222222222222	0	2	
1533	1	0.25	0	4	
1544	1	0.25	0	4	
1570	1	0.25	0	4	
1586	1	0.125	0	4	
1627	1	0.25	0	4	
1644	1	0.25	0	4	
1816	1	0.25	0	4	
1901	2	0.222222222222222	0	2	
1929	1	0.111111111111111	0	4	
1958	32	8	0	0	<<<
1969	1	0.25	0	4	
1982	1	0.25	0	4	
1986	1	0.25	0	4	
1989	2	0.222222222222222	0	2	
1992	1	0.25	0	4	
1994	3	0.75	0	2	
1998	1	0.25	0	4	
2001	1	0.25	0	4	
2009	1	0.25	0	4	
2010	1	0.25	0	4	
2015	1	0.25	0	4	
2017	1	0.25	0	4	
2020	1	0.25	0	4	
2021	1	0.25	0	4	
2024	1	0.25	0	4	
2041	1	0.25	0	4	
2045	1	0.25	0	4	
2052	1	0.25	0	4	
2055	1	0.25	0	4	
2056	1	0.25	0	4	
2058	1	0.25	0	4	
2060	1	0.25	0	4	
2061	1	0.25	0	4	
2066	1	0.25	0	4	
2068	2	0.5	0	2	
2198	1	0.25	0	4	
2224	1	0.25	0	4	
2233	1	0.25	0	4	

---

category=0, cleavage_site=1779
query=ptc-miR172g,h, target=Potri.010G216200.1,
score=2, range=1768-1788, strand=1

```
target  5' UUGCAGCAUCAuCAGGAUUCU 3'
           .:::::::::::::.:::::.
query   3' GACGUCGUAGUAGUUCUAAGG 5'
```

>Potri.010G216200.1
#size=2200

185	1	0.333333333333333	0	4
227	1	0.333333333333333	0	4
287	1	0.333333333333333	0	4
306	1	0.333333333333333	0	4
356	1	0.333333333333333	0	4
402	2	0.25	0	2
403	1	0.125	0	4
496	1	0.2	0	4
526	1	0.2	0	4
538	1	0.1	0	4
580	1	0.2	0	4
621	1	0.1	0	4
684	1	0.2	0	4
689	1	0.2	0	4
691	2	0.4	0	2
711	1	0.2	0	4
729	1	0.2	0	4
734	1	0.2	0	4
755	1	0.2	0	4
763	1	0.2	0	4
769	2	0.4	0	2
770	1	0.2	0	4
776	1	0.2	0	4
785	1	0.1	0	4
787	1	0.1	0	4
789	1	0.1	0	4
790	1	0.1	0	4
845	1	0.1	0	4
846	1	0.1	0	4
850	1	0.1	0	4
851	1	0.1	0	4
856	1	0.1	0	4
857	1	0.1	0	4
866	1	0.1	0	4
869	1	0.1	0	4
870	1	0.1	0	4
871	1	0.1	0	4
872	1	0.1	0	4
886	1	0.1	0	4
889	1	0.1	0	4
892	1	0.1	0	4
894	1	0.1	0	4
895	1	0.1	0	4
896	1	0.1	0	4
897	1	0.1	0	4
899	1	0.1	0	4
901	2	0.2	0	2
906	1	0.1	0	4
907	2	0.2	0	2
911	1	0.1	0	4
913	1	0.1	0	4
916	1	0.1	0	4
918	1	0.1	0	4
919	1	0.1	0	4
971	1	0.111111111111111	0	4
1022	1	0.111111111111111	0	4

1029	1	0.25	0	4	
1037	1	0.25	0	4	
1038	1	0.25	0	4	
1039	1	0.25	0	4	
1040	2	0.5	0	2	
1041	1	0.25	0	4	
1042	1	0.25	0	4	
1044	1	0.25	0	4	
1047	1	0.25	0	4	
1051	1	0.25	0	4	
1054	1	0.25	0	4	
1055	1	0.25	0	4	
1058	1	0.25	0	4	
1064	2	0.5	0	2	
1071	2	0.5	0	2	
1073	1	0.25	0	4	
1089	1	0.111111111111111	0	4	
1108	1	0.111111111111111	0	4	
1119	1	0.25	0	4	
1122	1	0.25	0	4	
1135	1	0.25	0	4	
1146	1	0.111111111111111	0	4	
1160	1	0.111111111111111	0	4	
1161	1	0.111111111111111	0	4	
1175	1	0.111111111111111	0	4	
1191	1	0.111111111111111	0	4	
1193	1	0.111111111111111	0	4	
1198	1	0.25	0	4	
1215	2	0.222222222222222	0	2	
1304	1	0.25	0	4	
1318	1	0.25	0	4	
1323	1	0.25	0	4	
1334	1	0.25	0	4	
1407	1	0.125	0	4	
1423	1	0.25	0	4	
1528	2	0.666666666666667	0	2	
1591	1	0.2	0	4	
1607	1	0.2	0	4	
1639	1	0.2	0	4	
1663	1	0.2	0	4	
1677	2	0.4	0	2	
1680	2	0.4	0	2	
1705	1	0.2	0	4	
1722	2	0.222222222222222	0	2	
1750	1	0.111111111111111	0	4	
1777	1	0.2	0	4	
1779	37	7.4	0	0	<<<
1786	1	0.2	0	4	
1798	2	0.4	0	2	
1799	1	0.2	0	4	
1800	1	0.2	0	4	
1801	1	0.2	0	4	
1806	2	0.4	0	2	
1807	3	0.422222222222222	0	2	
1809	1	0.2	0	4	
1810	1	0.2	0	4	
1816	1	0.2	0	4	
1827	1	0.2	0	4	

1830	1	0.2	0	4
1873	1	0.2	0	4
1880	3	0.6	0	2
1882	1	0.2	0	4
1890	1	0.2	0	4
1895	1	0.2	0	4
1906	1	0.2	0	4
1913	2	0.4	0	2
1923	1	0.2	0	4
1924	1	0.2	0	4
1944	1	0.2	0	4
1946	1	0.2	0	4
2001	1	0.2	0	4
2068	1	0.2	0	4
2076	1	0.2	0	4
2077	1	0.2	0	4

---

category=0, cleavage_site=1759
query=ptc-miR172g,h, target=Potri.016G084500.1,
score=2, range=1748-1768, strand=1

```
target  5' CUGCAGCAUCAuCAGGAUUCG 3'
           :::::::::::.:::::
query   3' GACGUCGUAGUAGUUCUAAGG 5'
```

>Potri.016G084500.1
#size=2370

62	1	0.25	0	4
195	1	0.5	0	4
418	1	0.5	0	4
484	1	0.5	0	4
495	1	0.25	0	4
503	1	0.25	0	4
507	1	0.25	0	4
513	1	0.5	0	4
554	1	0.25	0	4
569	1	0.5	0	4
583	1	0.5	0	4
655	1	0.5	0	4
676	1	0.5	0	4
685	1	0.5	0	4
690	3	1	0	2
691	3	0.75	0	2
693	3	1.25	0	2
694	1	0.25	0	4
695	1	0.5	0	4
696	1	0.5	0	4
704	1	0.5	0	4
710	1	0.5	0	4
720	1	0.5	0	4
724	1	0.5	0	4
728	3	1.5	0	2
730	1	0.5	0	4
733	1	0.5	0	4
734	2	1	0	2
735	1	0.5	0	4
738	1	0.5	0	4
740	2	1	0	2
750	1	0.5	0	4
766	1	0.5	0	4

774	1	0.5	0	4
817	1	0.5	0	4
824	1	0.5	0	4
833	1	0.5	0	4
834	1	0.5	0	4
847	1	0.25	0	4
856	1	0.25	0	4
857	1	0.25	0	4
863	1	0.25	0	4
871	1	0.25	0	4
872	2	0.5	0	2
879	2	0.5	0	2
888	2	0.5	0	2
892	1	0.25	0	4
893	1	0.25	0	4
894	2	0.5	0	2
901	1	0.25	0	4
904	1	0.25	0	4
908	2	0.5	0	2
918	3	0.75	0	2
921	1	0.25	0	4
922	1	0.25	0	4
928	2	0.5	0	2
931	1	0.25	0	4
940	1	0.25	0	4
941	1	0.25	0	4
943	1	0.25	0	4
953	2	0.5	0	2
957	2	0.5	0	2
959	1	0.25	0	4
961	1	0.25	0	4
964	1	0.25	0	4
971	1	0.25	0	4
976	1	0.25	0	4
1010	1	0.5	0	4
1056	1	0.25	0	4
1118	1	0.5	0	4
1142	1	0.25	0	4
1144	1	0.25	0	4
1145	2	0.5	0	2
1193	1	0.25	0	4
1207	1	0.333333333333333	0	4
1213	1	0.333333333333333	0	4
1216	1	0.333333333333333	0	4
1229	1	0.333333333333333	0	4
1251	1	0.333333333333333	0	4
1256	1	0.333333333333333	0	4
1271	1	0.333333333333333	0	4
1296	1	0.25	0	4
1314	1	0.25	0	4
1317	1	0.25	0	4
1380	1	0.25	0	4
1383	1	0.25	0	4
1386	1	0.25	0	4
1389	1	0.25	0	4
1438	1	0.5	0	4
1449	1	0.333333333333333	0	4
1450	1	0.333333333333333	0	4

1480	1	0.5	0	4	
1535	1	0.5	0	4	
1538	1	0.5	0	4	
1541	1	0.5	0	4	
1622	1	0.25	0	4	
1629	1	0.25	0	4	
1635	1	0.25	0	4	
1648	1	0.25	0	4	
1658	1	0.25	0	4	
1659	1	0.25	0	4	
1661	1	0.25	0	4	
1662	1	0.25	0	4	
1683	1	0.5	0	4	
1694	1	0.25	0	4	
1695	1	0.25	0	4	
1697	1	0.5	0	4	
1704	1	0.25	0	4	
1729	1	0.25	0	4	
1732	1	0.25	0	4	
1738	1	0.25	0	4	
1759	17	4.25	0	0	<<<
1760	2	0.5	0	2	
1788	1	0.25	0	4	
1789	3	0.75	0	2	
1803	2	1	0	2	
1816	1	0.5	0	4	
1861	1	0.25	0	4	
1866	2	0.75	0	2	
1875	1	0.5	0	4	
1879	1	0.5	0	4	
1882	1	0.25	0	4	
1898	1	0.25	0	4	
1899	1	0.25	0	4	
1900	1	0.5	0	4	
1903	1	0.25	0	4	
1909	1	0.5	0	4	
1910	1	0.5	0	4	
1924	1	0.5	0	4	
1926	1	0.5	0	4	
1934	1	0.5	0	4	
1936	1	0.5	0	4	
1937	1	0.5	0	4	
2016	2	0.75	0	2	
2027	1	0.5	0	4	
2283	1	0.5	0	4	

ptc-miR319a-d

category=0, cleavage_site=2736
query=ptc-miR319a-d, target=Potri.004G065800.1,
score=3.5, range=2726-2745, strand=1

```
target  5' AGGGGGACCCuUCAGUCCAA 3'
              ::.:  ::::::::::::
query   3' CCCUCGAGGGAAGUCAGGUU 5'
```

>Potri.004G065800.1
#size=3440

38	1	0.111111111111111	0	4
1286	1	0.111111111111111	0	4

1368	1	0.111111111111111	0	4	
1373	1	0.111111111111111	0	4	
1407	1	0.111111111111111	0	4	
1412	1	0.111111111111111	0	4	
1414	1	0.111111111111111	0	4	
1447	1	0.111111111111111	0	4	
1497	1	0.111111111111111	0	4	
1500	1	0.111111111111111	0	4	
1565	1	0.111111111111111	0	4	
1587	1	0.111111111111111	0	4	
1623	1	0.111111111111111	0	4	
1656	1	0.111111111111111	0	4	
1832	1	0.111111111111111	0	4	
2047	1	0.111111111111111	0	4	
2191	1	0.111111111111111	0	4	
2269	2	0.2	0	2	
2270	1	0.1	0	4	
2300	1	0.1	0	4	
2427	1	0.1	0	4	
2470	1	0.111111111111111	0	4	
2482	1	0.1	0	4	
2498	1	0.1	0	4	
2536	1	0.1	0	4	
2542	1	0.1	0	4	
2608	1	0.1	0	4	
2613	1	0.1	0	4	
2626	1	0.1	0	4	
2627	1	0.1	0	4	
2686	1	0.1	0	4	
2719	1	0.111111111111111	0	4	
2736	13	1.44444444444444	0	0	<<<
2740	2	0.222222222222222	0	2	
2745	1	0.111111111111111	0	4	
2752	1	0.111111111111111	0	4	
2771	2	0.2	0	2	
2876	1	0.111111111111111	0	4	
2885	1	0.1	0	4	
2891	1	0.1	0	4	
2898	1	0.1	0	4	
2901	1	0.1	0	4	
2903	1	0.1	0	4	
2922	1	0.1	0	4	
2968	1	0.111111111111111	0	4	
2970	1	0.166666666666667	0	4	
2971	1	0.166666666666667	0	4	
2972	3	0.5	0	2	
2973	1	0.166666666666667	0	4	
2979	1	0.166666666666667	0	4	
2985	1	0.166666666666667	0	4	
2993	1	0.166666666666667	0	4	
2999	1	0.166666666666667	0	4	
3015	1	0.125	0	4	
3019	1	0.125	0	4	
3048	1	0.1	0	4	
3056	1	0.1	0	4	
3115	1	0.125	0	4	
3118	1	0.125	0	4	
3119	1	0.125	0	4	

3132	1	0.125	0	4	
3158	1	0.1	0	4	
3182	1	0.1	0	4	
3187	1	0.125	0	4	

---

category=4, cleavage_site=1902
query=ptc-miR319a-d, target=Potri.011G083100.3,
score=3.5, range=1892-1911, strand=1

```
target  5' AGGGGGACCCuUCAGUCCAA 3'
             ::.:  ::::::::::::
query   3' CCCUCGAGGGAAGUCAGGUU 5'
```

>Potri.011G083100.3
#size=2463

348	1	0.25	0	4	
1902	1	0.1	0	4	<<<
1940	1	0.1	0	4	
2079	1	0.1	0	4	

---

category=4, cleavage_site=1562
query=ptc-miR319a-d, target=Potri.011G096600.1,
score=4, range=1552-1571, strand=1

```
target  5' AGGGGAACCCuUCAGUCCAG 3'
             ::.:  :::::::::::.
query   3' CCCUCGAGGGAAGUCAGGUU 5'
```

>Potri.011G096600.1
#size=2502

180	1	0.5	0	4	
181	1	0.5	0	4	
1562	1	0.5	0	4	<<<
1577	1	0.5	0	4	
1747	1	0.333333333333333	0	4	
1802	1	1	1	4	
1817	1	0.5	0	4	
1894	1	0.5	0	4	
1929	1	0.5	0	4	
1940	1	0.5	0	4	
1950	1	0.5	0	4	
1973	1	0.5	0	4	
1974	1	0.5	0	4	
2003	1	0.5	0	4	
2016	1	0.5	0	4	
2021	1	0.5	0	4	
2038	1	0.5	0	4	
2082	1	0.5	0	4	
2094	1	0.5	0	4	

---

category=4, cleavage_site=1743
query=ptc-miR319a-d, target=Potri.013G119400.1,
score=4, range=1733-1752, strand=1

```
target  5' AGGGGAACCCuUCAGUCCAG 3'
             ::.:  :::::::::::.
query   3' CCCUCGAGGGAAGUCAGGUU 5'
```

>Potri.013G119400.1
#size=2339

892	1	1	1	4	
1705	1	1	1	4	
1743	1	1	1	4	<<<

ptc-miR393a-d

category=0, cleavage_site=2012
query=ptc-miR393a-d, target=Potri.001G323100.1,
score=2, range=2001-2021, strand=1

```
target  5' AAACAAUGCGAuCCCUUUGGA 3'
             : :::::::::::::::::
query   3' CUAGUUACGCUAGGGAAACCU 5'
```

>Potri.001G323100.1
#size=2828

20	1	0.333333333333333	0	4
28	1	0.333333333333333	0	4
99	1	0.333333333333333	0	4
209	1	0.333333333333333	0	4
219	2	0.666666666666667	0	2
255	1	0.333333333333333	0	4
340	1	0.333333333333333	0	4
374	1	0.333333333333333	0	4
495	1	0.25	0	4
511	1	0.25	0	4
513	2	0.5	0	2
518	1	0.25	0	4
524	1	0.25	0	4
525	1	0.25	0	4
544	1	0.25	0	4
620	1	0.25	0	4
659	2	0.666666666666667	0	2
675	2	0.666666666666667	0	2
738	1	0.25	0	4
750	1	0.25	0	4
768	1	0.25	0	4
769	1	0.25	0	4
772	1	0.25	0	4
773	1	0.25	0	4
775	1	0.25	0	4
778	2	0.5	0	2
793	1	0.25	0	4
796	1	0.25	0	4
804	1	0.25	0	4
808	1	0.25	0	4
810	1	0.25	0	4
812	3	0.75	0	2
814	1	0.25	0	4
822	1	0.25	0	4
825	1	0.25	0	4
826	1	0.25	0	4
839	1	0.25	0	4
847	1	0.25	0	4
894	1	0.25	0	4
903	1	0.25	0	4
904	1	0.25	0	4
910	1	0.25	0	4
940	1	0.25	0	4
965	1	0.2	0	4
981	2	0.45	0	2
982	1	0.25	0	4
986	1	0.2	0	4
995	1	0.25	0	4

997	1	0.2	0	4
1085	1	0.25	0	4
1099	1	0.2	0	4
1116	1	0.2	0	4
1127	1	0.2	0	4
1188	1	0.2	0	4
1204	1	0.25	0	4
1205	1	0.25	0	4
1229	1	0.2	0	4
1255	1	0.25	0	4
1267	1	0.2	0	4
1276	1	0.2	0	4
1354	1	0.25	0	4
1358	1	0.25	0	4
1360	2	0.4	0	2
1372	1	0.25	0	4
1379	1	0.2	0	4
1399	1	0.25	0	4
1448	1	0.2	0	4
1497	1	0.2	0	4
1498	1	0.2	0	4
1501	1	0.2	0	4
1504	1	0.2	0	4
1507	1	0.2	0	4
1511	1	0.25	0	4
1512	1	0.25	0	4
1522	1	0.25	0	4
1523	3	0.75	0	2
1535	1	0.25	0	4
1540	2	0.5	0	2
1551	2	0.4	0	2
1566	1	0.25	0	4
1595	1	0.25	0	4
1605	1	0.2	0	4
1612	1	0.25	0	4
1632	1	0.333333333333333	0	4
1650	1	0.333333333333333	0	4
1686	1	0.5	0	4
1690	1	0.333333333333333	0	4
1700	1	0.5	0	4
1712	1	0.5	0	4
1741	1	0.333333333333333	0	4
1759	1	0.333333333333333	0	4
1773	1	0.5	0	4
1795	1	0.333333333333333	0	4
1829	1	0.25	0	4
1836	1	0.25	0	4
1883	1	0.25	0	4
1886	1	0.25	0	4
1888	1	0.25	0	4
1889	1	0.25	0	4
1897	1	0.333333333333333	0	4
1898	1	0.25	0	4
1899	2	0.5	0	2
1900	1	0.25	0	4
1904	1	0.25	0	4
1905	1	0.25	0	4
1906	1	0.2	0	4

1911	1	0.25	0	4	
1912	1	0.25	0	4	
1913	3	0.75	0	2	
1914	1	0.25	0	4	
1917	1	0.25	0	4	
1921	1	0.25	0	4	
1923	1	0.25	0	4	
1925	1	0.25	0	4	
1926	1	0.25	0	4	
1929	1	0.2	0	4	
1934	1	0.2	0	4	
1935	1	0.2	0	4	
1938	1	0.2	0	4	
1940	1	0.1	0	4	
1942	2	0.2	0	3	
1943	2	0.2	0	3	
1944	2	0.4	0	2	
1945	1	0.2	0	4	
1947	3	0.6	0	2	
1948	5	1	0	2	
1949	1	0.2	0	4	
1954	2	0.4	0	2	
1958	1	0.2	0	4	
1959	2	0.4	0	2	
1960	1	0.2	0	4	
1961	2	0.4	0	2	
1962	1	0.2	0	4	
1965	1	0.2	0	4	
1967	1	0.2	0	4	
1976	3	0.6	0	2	
1977	1	0.2	0	4	
1982	1	0.2	0	4	
1984	1	0.2	0	4	
1985	1	0.2	0	4	
1987	1	0.2	0	4	
1989	1	0.2	0	4	
1991	1	0.2	0	4	
1993	1	0.2	0	4	
2010	1	0.2	0	4	
2011	1	0.25	0	4	
2012	103	24.8	0	0	<<<
2017	1	0.2	0	4	
2018	1	0.25	0	4	
2021	1	0.25	0	4	
2026	1	0.25	0	4	
2027	1	0.2	0	4	
2029	3	0.7	0	2	
2030	1	0.2	0	4	
2031	1	0.2	0	4	
2033	1	0.2	0	4	
2043	1	0.2	0	4	
2047	1	0.2	0	4	
2055	1	0.2	0	4	
2056	1	0.2	0	4	
2057	2	0.4	0	2	
2058	2	0.4	0	2	
2059	1	0.2	0	4	
2060	1	0.2	0	4	

2061	2	0.4	0	2
2062	3	0.6	0	2
2063	6	1.2	0	2
2070	2	0.45	0	2
2077	1	0.25	0	4
2080	1	0.25	0	4
2081	1	0.25	0	4
2083	1	0.25	0	4
2084	1	0.25	0	4
2085	1	0.25	0	4
2087	1	0.25	0	4
2092	1	0.25	0	4
2093	1	0.25	0	4
2094	1	0.25	0	4
2095	1	0.25	0	4
2130	1	0.25	0	4
2139	2	0.5	0	2
2143	1	0.25	0	4
2144	2	0.5	0	2
2145	1	0.25	0	4
2147	1	0.25	0	4
2148	1	0.25	0	4
2150	1	0.25	0	4
2153	2	0.5	0	2
2154	2	0.5	0	2
2155	1	0.25	0	4
2156	1	0.25	0	4
2157	1	0.25	0	4
2158	1	0.25	0	4
2162	1	0.25	0	4
2163	3	0.75	0	2
2165	1	0.25	0	4
2166	2	0.45	0	2
2169	2	0.5	0	2
2170	1	0.2	0	4
2171	2	0.5	0	2
2172	2	0.5	0	2
2175	1	0.25	0	4
2176	2	0.4	0	2
2178	1	0.2	0	4
2179	2	0.4	0	2
2180	1	0.2	0	4
2181	3	0.6	0	2
2182	1	0.2	0	4
2184	3	0.6	0	2
2185	1	0.2	0	4
2186	2	0.4	0	2
2187	1	0.2	0	4
2188	1	0.2	0	4
2190	2	0.4	0	2
2191	1	0.25	0	4
2192	1	0.25	0	4
2195	2	0.5	0	2
2197	1	0.25	0	4
2198	1	0.25	0	4
2212	1	0.25	0	4
2215	2	0.5	0	2
2218	1	0.25	0	4

2234	1	0.25	0	4
2261	3	0.75	0	2
2266	1	0.25	0	4
2270	1	0.25	0	4
2326	1	0.25	0	4
2451	1	0.25	0	4
2616	1	0.25	0	4

---

category=0, cleavage_site=1823
query=ptc-miR393a-d, target=Potri.002G207800.1,
score=1, range=1813-1832, strand=1

```
target  5' GA-CAAUGCGAuCCCUUUGGA 3'
           :: :::::::::::::::::
query   3' CUAGUUACGCUAGGGAAACCU 5'
```

>Potri.002G207800.1
#size=2616

188	1	1	1	4	
297	2	2	2	2	
309	1	1	1	4	
322	1	1	1	4	
378	1	0.5	0	4	
387	1	1	1	4	
397	1	1	1	4	
411	1	1	1	4	
414	1	1	1	4	
415	2	2	2	2	
428	1	1	1	4	
429	1	0.5	0	4	
431	1	0.5	0	4	
471	1	0.5	0	4	
558	1	1	1	4	
698	1	0.5	0	4	
753	1	1	1	4	
978	1	1	1	4	
1294	1	0.5	0	4	
1361	1	0.5	0	4	
1431	1	0.5	0	4	
1441	1	0.5	0	4	
1444	1	0.5	0	4	
1611	1	0.5	0	4	
1707	1	1	1	4	
1710	1	1	1	4	
1721	1	1	1	4	
1739	1	1	1	4	
1757	1	1	1	4	
1781	1	0.5	0	4	
1822	1	1	1	4	
1823	14	13.5	13	0	<<<
1849	1	1	1	4	
1866	1	0.5	0	4	
1871	2	1	0	2	
1874	1	0.5	0	4	
1875	1	0.5	0	4	
1893	1	0.5	0	4	
1905	1	0.5	0	4	
1918	2	2	2	2	
1923	1	1	1	4	
1930	1	1	1	4	

1939	1	0.5	0	4
1975	1	1	1	4
1987	1	1	1	4
1991	1	1	1	4
1993	1	1	1	4
2005	1	0.5	0	4
2019	1	1	1	4
2023	1	1	1	4
2027	1	1	1	4
2033	1	1	1	4
2034	1	1	1	4
2047	1	1	1	4
2081	1	1	1	4
2082	1	1	1	4
2126	1	1	1	4
2157	1	1	1	4
2188	1	1	1	4
2190	2	2	2	2
2197	1	1	1	4
2201	1	1	1	4
2202	2	2	2	2
2206	1	1	1	4
2209	1	1	1	4
2217	2	2	2	2
2218	1	1	1	4
2220	1	1	1	4
2227	1	1	1	4
2252	1	1	1	4
2275	1	1	1	4
2283	1	1	1	4
2284	1	1	1	4
2303	2	2	2	2

---

category=2, cleavage_site=606
query=ptc-miR393a-d, target=Potri.002G235400.1,
score=4, range=595-615, strand=1

```
target  5' GAUCAGAGCGAuCCCUUUGAG 3'
           :::::. :::::::::::: .
query   3' CUAGUUACGCUAGGGAAACCU 5'
```

>Potri.002G235400.1
#size=2576

4	1	0.333333333333333	0	4	
137	1	0.333333333333333	0	4	
161	1	0.333333333333333	0	4	
473	2	0.666666666666667	0	2	
492	1	0.333333333333333	0	4	
535	2	2	2	2	
544	1	1	1	4	
550	1	0.333333333333333	0	4	
583	1	0.333333333333333	0	4	
589	1	0.333333333333333	0	4	
593	1	0.333333333333333	0	4	
606	3	1	0	2	<<<
632	1	1	1	4	
650	1	1	1	4	
675	1	1	1	4	
678	1	1	1	4	
716	4	1.33333333333333	0	2	

732	1	1	1	4		
764	1	1	1	4		
865	1	0.333333333333333			0	4
875	1	1	1	4		
880	1	1	1	4		
883	1	1	1	4		
887	2	2	2	2		
891	1	1	1	4		
940	1	1	1	4		
942	1	1	1	4		
1045	1	1	1	4		
1049	1	0.333333333333333			0	4
1052	1	0.333333333333333			0	4
1057	2	0.666666666666667			0	2
1063	1	0.333333333333333			0	4
1064	1	0.333333333333333			0	4
1067	2	0.666666666666667			0	2
1069	1	0.333333333333333			0	4
1071	1	0.333333333333333			0	4
1072	1	0.333333333333333			0	4
1073	1	0.333333333333333			0	4
1078	1	0.333333333333333			0	4
1100	1	0.333333333333333			0	4
1104	1	0.333333333333333			0	4
1112	1	0.333333333333333			0	4
1120	1	0.333333333333333			0	4
1129	1	0.333333333333333			0	4
1133	1	1	1	4		
1137	1	0.333333333333333			0	4
1142	2	1.33333333333333			1	2
1166	2	2	2	2		
1184	1	1	1	4		
1205	1	1	1	4		
1206	1	1	1	4		
1212	1	0.333333333333333			0	4
1213	3	3	3	1		
1214	1	0.333333333333333			0	4
1222	1	1	1	4		
1232	1	1	1	4		
1245	1	1	1	4		
1258	1	1	1	4		
1271	1	0.333333333333333			0	4
1275	2	0.666666666666667			0	2
1278	2	0.666666666666667			0	2
1280	1	0.333333333333333			0	4
1281	1	0.333333333333333			0	4
1283	1	0.333333333333333			0	4
1286	1	0.333333333333333			0	4
1292	1	0.333333333333333			0	4
1298	1	0.333333333333333			0	4
1300	1	0.333333333333333			0	4
1302	3	1	0	2		
1303	1	0.333333333333333			0	4
1304	1	0.333333333333333			0	4
1313	1	0.333333333333333			0	4
1315	1	0.333333333333333			0	4
1316	1	0.333333333333333			0	4
1318	1	0.333333333333333			0	4

1321	2	0.666666666666667	0	2
1322	2	1.33333333333333	1	2
1354	1	0.333333333333333	0	4
1355	1	0.333333333333333	0	4
1357	2	0.666666666666667	0	2
1362	1	0.333333333333333	0	4
1371	1	0.333333333333333	0	4
1386	3	3	3	1
1410	1	1	1	4
1411	1	1	1	4
1412	1	1	1	4
1421	1	1	1	4
1429	1	1	1	4
1432	1	1	1	4
1438	1	1	1	4
1440	1	1	1	4
1441	1	1	1	4
1460	1	1	1	4
1466	1	1	1	4
1508	1	0.333333333333333	0	4
1513	1	0.333333333333333	0	4
1522	1	0.333333333333333	0	4
1523	1	0.333333333333333	0	4
1527	1	0.333333333333333	0	4
1533	1	0.333333333333333	0	4
1543	1	0.333333333333333	0	4
1565	1	0.333333333333333	0	4
1572	1	0.333333333333333	0	4
1589	2	0.4	0	3
1632	2	0.666666666666667	0	2
1640	1	0.333333333333333	0	4
1650	1	0.333333333333333	0	4
1652	1	0.333333333333333	0	4
1682	1	0.333333333333333	0	4
1697	1	0.333333333333333	0	4
1706	1	0.333333333333333	0	4
1726	1	1	1	4
1740	1	1	1	4
1746	1	1	1	4
1750	1	1	1	4
1763	1	1	1	4
1779	1	0.333333333333333	0	4
1800	1	0.333333333333333	0	4
1810	1	0.333333333333333	0	4
1916	1	1	1	4
1918	1	1	1	4
1922	1	1	1	4
1923	1	1	1	4
1968	1	1	1	4
1983	1	1	1	4
1996	1	1	1	4
2039	2	0.666666666666667	0	2
2040	1	0.333333333333333	0	4
2045	1	0.333333333333333	0	4
2046	1	0.333333333333333	0	4
2047	2	0.666666666666667	0	2
2050	2	0.666666666666667	0	2
2055	1	0.333333333333333	0	4

2056	1	0.333333333333333	0	4
2060	1	1	1	4
2074	1	1	1	4
2075	1	1	1	4
2188	1	0.333333333333333	0	4
2191	1	0.333333333333333	0	4
2192	2	0.666666666666667	0	2
2197	1	1	1	4
2203	1	1	1	4
2231	1	1	1	4
2288	1	1	1	4
2367	1	1	1	4
2381	1	1	1	4

---

category=4, cleavage_site=1607
query=ptc-miR393a-d, target=Potri.014G134800.1,
score=1, range=1597-1616, strand=1

```
target  5' GA-CAAUGCGAuCCCUUUGGA 3'
           :: ::::::::::::::::::
query   3' CUAGUUACGCUAGGGAAACCU 5'
```

>Potri.014G134800.1
#size=2138

162	1	0.5	0	4	
213	1	0.5	0	4	
215	1	0.5	0	4	
255	1	0.5	0	4	
482	1	0.5	0	4	
1078	1	0.5	0	4	
1145	1	0.5	0	4	
1215	1	0.5	0	4	
1225	1	0.5	0	4	
1228	1	0.5	0	4	
1395	1	0.5	0	4	
1565	1	0.5	0	4	
1607	1	0.5	0	4	<<<
1650	1	0.5	0	4	
1655	2	1	0	2	
1658	1	0.5	0	4	
1659	1	0.5	0	4	
1677	1	0.5	0	4	
1689	1	0.5	0	4	
1723	1	0.5	0	4	
1789	1	0.5	0	4	
1808	1	1	1	4	
1841	1	1	1	4	
1859	1	1	1	4	
1868	1	1	1	4	
1876	2	2	2	0	
2012	1	1	1	4	
2014	1	1	1	4	
2069	1	1	1	4	
2075	1	1	1	4	
2080	1	1	1	4	

category=2, cleavage_site=624
query=ptc-miR393a-d, target=Potri.014G148900.1,
score=4, range=613-633, strand=1

```
target  5' GAUCAGAGCGAuCCCUUUGAG 3'
           :::::. ::::::::::: .
query   3' CUAGUUACGCUAGGGAAACCU 5'
```

>Potri.014G148900.1
#size=2594

16	1	0.333333333333333	0	4	
76	1	0.5	0	4	
87	1	0.5	0	4	
157	1	0.333333333333333	0	4	
181	1	0.333333333333333	0	4	
472	1	0.5	0	4	
491	2	0.666666666666667	0	2	
510	1	0.333333333333333	0	4	
539	1	0.5	0	4	
550	1	0.5	0	4	
562	1	0.5	0	4	
568	1	0.333333333333333	0	4	
601	1	0.333333333333333	0	4	
607	1	0.333333333333333	0	4	
611	1	0.333333333333333	0	4	
624	3	1	0	2	<<<
647	2	1	0	2	
740	4	1.33333333333333	0	2	
750	1	0.5	0	4	
794	1	0.5	0	4	
889	1	0.333333333333333	0	4	
926	1	0.5	0	4	
931	1	0.5	0	4	
945	1	0.5	0	4	
957	1	0.5	0	4	
979	1	0.5	0	4	
980	1	0.5	0	4	
999	1	0.5	0	4	
1067	1	0.333333333333333	0	4	
1070	1	0.333333333333333	0	4	
1075	2	0.666666666666667	0	2	
1081	1	0.333333333333333	0	4	
1082	1	0.333333333333333	0	4	
1085	2	0.666666666666667	0	2	
1087	1	0.333333333333333	0	4	
1089	1	0.333333333333333	0	4	
1090	1	0.333333333333333	0	4	
1091	1	0.333333333333333	0	4	
1096	1	0.333333333333333	0	4	
1118	1	0.333333333333333	0	4	
1122	1	0.333333333333333	0	4	
1130	1	0.333333333333333	0	4	
1138	1	0.333333333333333	0	4	
1147	1	0.333333333333333	0	4	
1155	1	0.333333333333333	0	4	
1160	1	0.333333333333333	0	4	
1198	1	0.5	0	4	
1230	1	0.333333333333333	0	4	
1232	1	0.333333333333333	0	4	
1253	1	0.5	0	4	

1263	1	0.5	0	4
1278	1	0.5	0	4
1279	1	0.5	0	4
1283	2	1	0	2
1284	1	0.5	0	4
1289	1	0.333333333333333	0	4
1293	2	0.666666666666667	0	2
1296	2	0.666666666666667	0	2
1297	1	0.5	0	4
1298	1	0.333333333333333	0	4
1299	1	0.333333333333333	0	4
1301	1	0.333333333333333	0	4
1304	2	0.833333333333333	0	2
1310	1	0.333333333333333	0	4
1316	1	0.333333333333333	0	4
1318	1	0.333333333333333	0	4
1320	3	1	0	2
1321	1	0.333333333333333	0	4
1322	1	0.333333333333333	0	4
1331	1	0.333333333333333	0	4
1333	1	0.333333333333333	0	4
1334	1	0.333333333333333	0	4
1336	1	0.333333333333333	0	4
1339	2	0.666666666666667	0	2
1340	1	0.333333333333333	0	4
1343	1	0.5	0	4
1344	1	0.5	0	4
1345	1	0.5	0	4
1352	3	1.5	0	2
1353	2	1	0	2
1354	1	0.5	0	4
1356	1	0.5	0	4
1357	1	0.5	0	4
1361	1	0.5	0	4
1363	1	0.5	0	4
1365	1	0.5	0	4
1372	1	0.333333333333333	0	4
1373	1	0.333333333333333	0	4
1375	2	0.666666666666667	0	2
1380	1	0.333333333333333	0	4
1389	1	0.333333333333333	0	4
1395	1	0.5	0	4
1401	2	1	0	2
1404	1	0.5	0	4
1406	1	0.5	0	4
1408	1	0.5	0	4
1412	1	0.5	0	4
1413	2	1	0	2
1417	1	0.5	0	4
1421	1	0.5	0	4
1425	2	1	0	2
1426	1	0.5	0	4
1430	1	0.5	0	4
1432	3	1.5	0	2
1433	1	0.5	0	4
1442	1	0.5	0	4
1444	2	1	0	2
1450	1	0.5	0	4

1455	1	0.5	0	4
1459	1	0.5	0	4
1475	1	0.5	0	4
1485	1	0.5	0	4
1488	1	0.5	0	4
1497	1	0.5	0	4
1504	1	0.5	0	4
1526	1	0.333333333333333	0	4
1531	1	0.333333333333333	0	4
1540	1	0.333333333333333	0	4
1541	1	0.333333333333333	0	4
1545	1	0.333333333333333	0	4
1551	1	0.333333333333333	0	4
1561	1	0.333333333333333	0	4
1583	1	0.333333333333333	0	4
1590	1	0.333333333333333	0	4
1600	1	0.333333333333333	0	4
1629	1	0.5	0	4
1650	2	0.666666666666667	0	2
1658	1	0.333333333333333	0	4
1668	1	0.333333333333333	0	4
1670	1	0.333333333333333	0	4
1700	1	0.333333333333333	0	4
1708	1	0.5	0	4
1715	2	0.833333333333333	0	2
1724	1	0.333333333333333	0	4
1797	1	0.333333333333333	0	4
1818	1	0.333333333333333	0	4
1828	1	0.333333333333333	0	4
1901	1	0.5	0	4
1902	1	0.5	0	4
1960	1	0.5	0	4
1963	1	0.5	0	4
1975	1	0.5	0	4
2057	1	0.5	0	4
2068	1	0.5	0	4
2070	1	0.5	0	4
2079	2	0.666666666666667	0	2
2080	1	0.333333333333333	0	4
2085	1	0.333333333333333	0	4
2086	1	0.333333333333333	0	4
2087	2	0.666666666666667	0	2
2090	2	0.666666666666667	0	2
2095	1	0.333333333333333	0	4
2096	1	0.333333333333333	0	4
2100	1	0.5	0	4
2105	1	0.5	0	4
2107	1	0.5	0	4
2108	3	1.5	0	2
2109	3	1.5	0	2
2110	5	2.5	0	1
2111	2	1	0	2
2112	5	2.5	0	1
2114	5	2.5	0	1
2115	1	0.5	0	4
2116	1	0.5	0	4
2118	1	0.5	0	4
2119	2	1	0	2

2123	2	1	0	2
2126	1	0.5	0	4
2127	2	1	0	2
2128	1	0.5	0	4
2134	1	0.5	0	4
2154	1	0.5	0	4
2169	1	0.5	0	4
2176	1	0.5	0	4
2177	1	0.5	0	4
2179	2	1	0	2
2184	1	0.5	0	4
2185	1	0.5	0	4
2192	1	0.5	0	4
2225	1	0.333333333333333	0	4
2228	1	0.333333333333333	0	4
2229	2	0.666666666666667	0	2
2244	3	1.5	0	2
2308	1	0.5	0	4
2309	1	0.5	0	4
2318	2	1	0	2
2364	2	1	0	2
2375	1	0.5	0	4
2376	1	0.5	0	4
2381	2	1	0	2
2398	1	0.5	0	4
2402	1	0.5	0	4
2418	1	0.5	0	4

ptc-miR394a,b-5p

category=0, cleavage_site=1482
query=ptc-miR394a,b-5p, target=Potri.001G057100.1,
score=1, range=1472-1491, strand=1

```
target  5' GGAGGUUGACaGAAUGCCAA 3'
           :::::: ::::::::::::
query   3' CCUCCACCUGUCUUACGGUU 5'
```

>Potri.001G057100.1
#size=1943

296	1	0.333333333333333	0	4
304	1	0.333333333333333	0	4
367	1	0.333333333333333	0	4
471	2	0.5	0	2
474	1	0.2	0	4
475	2	0.333333333333333	0	2
484	1	0.166666666666667	0	4
493	1	0.166666666666667	0	4
509	1	0.166666666666667	0	4
510	2	0.333333333333333	0	2
513	3	0.5	0	2
518	1	0.166666666666667	0	4
532	1	0.333333333333333	0	4
540	1	0.333333333333333	0	4
558	1	0.333333333333333	0	4
567	1	0.333333333333333	0	4
679	1	0.166666666666667	0	4
682	1	0.333333333333333	0	4
706	1	0.166666666666667	0	4
751	2	0.333333333333333	0	2

755	1	0.166666666666667	0	4
758	2	0.333333333333333	0	2
790	1	0.166666666666667	0	4
800	1	0.166666666666667	0	4
850	1	0.166666666666667	0	4
856	1	0.166666666666667	0	4
861	1	0.166666666666667	0	4
864	1	0.166666666666667	0	4
886	1	0.333333333333333	0	4
942	1	0.333333333333333	0	4
962	1	0.166666666666667	0	4
969	1	0.166666666666667	0	4
970	1	0.166666666666667	0	4
977	1	0.166666666666667	0	4
998	1	0.166666666666667	0	4
1000	1	0.166666666666667	0	4
1023	1	0.166666666666667	0	4
1029	1	0.166666666666667	0	4
1032	1	0.333333333333333	0	4
1035	1	0.333333333333333	0	4
1052	1	0.166666666666667	0	4
1053	2	0.5 0 2		
1079	1	0.333333333333333	0	4
1081	1	0.333333333333333	0	4
1085	1	0.333333333333333	0	4
1091	2	0.333333333333333	0	2
1099	1	0.166666666666667	0	4
1100	1	0.166666666666667	0	4
1108	1	0.166666666666667	0	4
1117	1	0.166666666666667	0	4
1118	1	0.166666666666667	0	4
1124	1	0.166666666666667	0	4
1127	1	0.333333333333333	0	4
1138	1	0.166666666666667	0	4
1142	1	0.166666666666667	0	4
1187	1	0.166666666666667	0	4
1188	1	0.166666666666667	0	4
1197	1	0.166666666666667	0	4
1200	1	0.166666666666667	0	4
1212	1	0.166666666666667	0	4
1236	5	0.833333333333333	0	2
1246	2	0.333333333333333	0	2
1248	1	0.166666666666667	0	4
1253	1	0.166666666666667	0	4
1254	1	0.166666666666667	0	4
1255	1	0.166666666666667	0	4
1258	1	0.166666666666667	0	4
1259	2	0.5 0 2		
1265	2	0.333333333333333	0	2
1266	2	0.333333333333333	0	2
1268	1	0.333333333333333	0	4
1271	1	0.333333333333333	0	4
1273	2	0.666666666666667	0	2
1278	2	0.333333333333333	0	2
1280	1	0.333333333333333	0	4
1290	1	0.166666666666667	0	4
1298	1	0.166666666666667	0	4
1304	1	0.333333333333333	0	4

1317	1	0.333333333333333	0	4	
1351	1	0.333333333333333	0	4	
1365	1	0.333333333333333	0	4	
1371	1	0.333333333333333	0	4	
1382	1	0.166666666666667	0	4	
1387	1	0.166666666666667	0	4	
1388	2	0.666666666666667	0	2	
1398	2	0.333333333333333	0	2	
1412	4	0.666666666666667	0	2	
1415	1	0.166666666666667	0	4	
1417	1	0.166666666666667	0	4	
1428	1	0.166666666666667	0	4	
1435	1	0.166666666666667	0	4	
1436	1	0.166666666666667	0	4	
1448	1	0.166666666666667	0	4	
1449	1	0.166666666666667	0	4	
1452	1	0.166666666666667	0	4	
1458	1	0.166666666666667	0	4	
1459	2	0.333333333333333	0	2	
1462	1	0.166666666666667	0	4	
1470	2	0.333333333333333	0	2	
1472	1	0.166666666666667	0	4	
1474	1	0.166666666666667	0	4	
1475	1	0.166666666666667	0	4	
1476	1	0.166666666666667	0	4	
1477	1	0.166666666666667	0	4	
1478	1	0.166666666666667	0	4	
1480	1	0.166666666666667	0	4	
1481	1	0.166666666666667	0	4	
1482	59	9.83333333333333	0	0	<<<
1483	7	1.16666666666667	0	2	
1484	3	0.5	0	2	
1485	2	0.333333333333333	0	2	
1486	1	0.166666666666667	0	4	
1488	2	0.333333333333333	0	2	
1492	3	0.5	0	2	
1493	1	0.166666666666667	0	4	
1495	5	0.833333333333333	0	2	
1497	1	0.166666666666667	0	4	
1498	1	0.166666666666667	0	4	
1502	2	0.333333333333333	0	2	
1507	4	0.666666666666667	0	2	
1509	2	0.333333333333333	0	2	
1511	2	0.333333333333333	0	2	
1512	1	0.166666666666667	0	4	
1514	2	0.333333333333333	0	2	
1519	1	0.166666666666667	0	4	
1523	1	0.166666666666667	0	4	
1536	1	0.333333333333333	0	4	
1559	1	0.166666666666667	0	4	
1565	1	0.333333333333333	0	4	
1587	1	0.333333333333333	0	4	
1598	2	0.333333333333333	0	2	
1604	1	0.166666666666667	0	4	
1607	1	0.166666666666667	0	4	
1609	1	0.166666666666667	0	4	
1612	1	0.166666666666667	0	4	
1629	1	0.166666666666667	0	4	

1644	1	0.166666666666667	0	4
1649	1	0.166666666666667	0	4
1657	1	0.166666666666667	0	4
1658	1	0.166666666666667	0	4
1660	1	0.166666666666667	0	4
1669	1	0.333333333333333	0	4
1674	1	0.166666666666667	0	4
1690	1	0.333333333333333	0	4
1702	1	0.333333333333333	0	4
1721	1	0.333333333333333	0	4
1799	1	0.333333333333333	0	4
1805	1	0.333333333333333	0	4
1861	1	0.333333333333333	0	4

---

category=0, cleavage_site=1401
query=ptc-miR394a,b-5p, target=Potri.003G171300.1,
score=1, range=1391-1410, strand=1

```
target  5' GGAGGUUGACaGAAUGCCAA 3'
            :::::: ::::::::::::: 
query   3' CCUCCACCUGUCUUACGGUU 5'
```

>Potri.003G171300.1
#size=1891

81	1	0.5	0	4
82	2	0.666666666666667	0	2
84	1	0.333333333333333	0	4
93	1	0.333333333333333	0	4
155	1	0.333333333333333	0	4
175	1	0.333333333333333	0	4
178	1	0.333333333333333	0	4
195	1	0.333333333333333	0	4
384	3	1.5	0	2
386	1	0.5	0	4
387	2	1	0	2
390	2	0.5	0	2
393	1	0.2	0	4
394	2	0.333333333333333	0	2
403	1	0.166666666666667	0	4
412	1	0.166666666666667	0	4
428	1	0.166666666666667	0	4
429	2	0.333333333333333	0	2
432	3	0.5	0	2
437	1	0.166666666666667	0	4
468	1	0.333333333333333	0	4
577	1	0.333333333333333	0	4
598	1	0.166666666666667	0	4
625	1	0.166666666666667	0	4
670	2	0.333333333333333	0	2
674	1	0.166666666666667	0	4
677	2	0.333333333333333	0	2
709	1	0.166666666666667	0	4
719	1	0.166666666666667	0	4
769	1	0.166666666666667	0	4
775	1	0.166666666666667	0	4
780	1	0.166666666666667	0	4
783	1	0.166666666666667	0	4
881	1	0.166666666666667	0	4
888	1	0.166666666666667	0	4
889	1	0.166666666666667	0	4

896	1	0.166666666666667	0	4
917	1	0.166666666666667	0	4
919	1	0.166666666666667	0	4
942	1	0.166666666666667	0	4
947	1	0.333333333333333	0	4
948	1	0.166666666666667	0	4
971	1	0.166666666666667	0	4
972	1	0.166666666666667	0	4
1010	2	0.333333333333333	0	2
1018	1	0.166666666666667	0	4
1019	1	0.166666666666667	0	4
1027	1	0.166666666666667	0	4
1036	1	0.166666666666667	0	4
1037	1	0.166666666666667	0	4
1043	1	0.166666666666667	0	4
1057	1	0.166666666666667	0	4
1061	1	0.166666666666667	0	4
1106	1	0.166666666666667	0	4
1107	1	0.166666666666667	0	4
1116	1	0.166666666666667	0	4
1119	1	0.166666666666667	0	4
1131	1	0.166666666666667	0	4
1155	5	0.833333333333333	0	2
1165	2	0.333333333333333	0	2
1167	1	0.166666666666667	0	4
1172	1	0.166666666666667	0	4
1173	1	0.166666666666667	0	4
1174	1	0.166666666666667	0	4
1177	1	0.166666666666667	0	4
1178	1	0.166666666666667	0	4
1184	2	0.333333333333333	0	2
1185	2	0.333333333333333	0	2
1186	2	0.666666666666667	0	2
1190	1	0.333333333333333	0	4
1191	1	0.333333333333333	0	4
1192	1	0.333333333333333	0	4
1197	2	0.333333333333333	0	2
1209	1	0.166666666666667	0	4
1217	1	0.166666666666667	0	4
1224	1	0.333333333333333	0	4
1231	1	0.333333333333333	0	4
1236	1	0.333333333333333	0	4
1246	1	0.333333333333333	0	4
1248	1	0.333333333333333	0	4
1265	2	0.666666666666667	0	2
1273	1	0.333333333333333	0	4
1285	1	0.333333333333333	0	4
1301	1	0.166666666666667	0	4
1306	1	0.166666666666667	0	4
1317	2	0.333333333333333	0	2
1331	4	0.666666666666667	0	2
1334	1	0.166666666666667	0	4
1336	1	0.166666666666667	0	4
1347	1	0.166666666666667	0	4
1354	1	0.166666666666667	0	4
1355	1	0.166666666666667	0	4
1367	1	0.166666666666667	0	4
1368	1	0.166666666666667	0	4

1371	1	0.166666666666667	0	4	
1377	1	0.166666666666667	0	4	
1378	2	0.333333333333333	0	2	
1381	1	0.166666666666667	0	4	
1389	2	0.333333333333333	0	2	
1391	1	0.166666666666667	0	4	
1393	1	0.166666666666667	0	4	
1394	1	0.166666666666667	0	4	
1395	1	0.166666666666667	0	4	
1396	1	0.166666666666667	0	4	
1397	1	0.166666666666667	0	4	
1399	1	0.166666666666667	0	4	
1400	1	0.166666666666667	0	4	
1401	59	9.83333333333333	0	0	<<<
1402	7	1.16666666666667	0	2	
1403	3	0.5	0	2	
1404	2	0.333333333333333	0	2	
1405	1	0.166666666666667	0	4	
1407	2	0.333333333333333	0	2	
1411	3	0.5	0	2	
1412	1	0.166666666666667	0	4	
1414	5	0.833333333333333	0	2	
1416	1	0.166666666666667	0	4	
1417	1	0.166666666666667	0	4	
1421	2	0.333333333333333	0	2	
1426	4	0.666666666666667	0	2	
1428	2	0.333333333333333	0	2	
1430	2	0.333333333333333	0	2	
1431	1	0.166666666666667	0	4	
1433	2	0.333333333333333	0	2	
1438	1	0.166666666666667	0	4	
1442	1	0.166666666666667	0	4	
1478	1	0.166666666666667	0	4	
1485	1	0.333333333333333	0	4	
1517	2	0.333333333333333	0	2	
1523	1	0.166666666666667	0	4	
1526	1	0.166666666666667	0	4	
1528	1	0.166666666666667	0	4	
1531	1	0.166666666666667	0	4	
1548	1	0.166666666666667	0	4	
1563	1	0.166666666666667	0	4	
1568	1	0.166666666666667	0	4	
1576	1	0.166666666666667	0	4	
1577	1	0.166666666666667	0	4	
1579	1	0.166666666666667	0	4	
1593	1	0.166666666666667	0	4	
1680	1	0.333333333333333	0	4	
1694	1	0.333333333333333	0	4	
1695	1	0.333333333333333	0	4	
1697	1	0.333333333333333	0	4	
1792	1	0.333333333333333	0	4	
1800	1	0.333333333333333	0	4	

ptc-miR396a,b

category=4, cleavage_site=817
query=ptc-miR396a,b, target=Potri.001G082700.1,
score=3, range=806-827, strand=1

```
target  5' CCGUUCAAGAAaGCCUGUGGAA 3'
           : ::::::::::: ::::::::
query   3' GUCAAGUUCUUUC-GACACCUU 5'
```

>Potri.001G082700.1
#size=1956

817	1	1	1	4	<<<
1562	1	1	1	4	

category=0, cleavage_site=681
query=ptc-miR396a,b, target=Potri.001G114000.1,
score=4, range=670-691, strand=1

```
target  5' GCGUUCAAGAAaGCUUGUGGAA 3'
             :::::::::::: :::::::
query   3' GUCAAGUUCUUUCG-ACACCUU 5'
```

>Potri.001G114000.1
#size=1087

109	1	0.333333333333333	0	4	
357	1	0.333333333333333	0	4	
465	2	0.5	0	2	
530	1	0.5	0	4	
587	1	0.5	0	4	
673	1	0.5	0	4	
681	50	25	0	0	<<<
682	1	0.5	0	4	
697	1	0.5	0	4	
708	1	0.5	0	4	
715	1	0.5	0	4	
723	1	0.5	0	4	
755	1	0.5	0	4	

category=2, cleavage_site=581
query=ptc-miR396a,b, target=Potri.001G132600.1,
score=3, range=570-591, strand=1

```
target  5' CCGUUCAAGAAaGCCUGUGGAA 3'
           : ::::::::::: ::::::::
query   3' GUCAAGUUCUUUC-GACACCUU 5'
```

>Potri.001G132600.1
#size=1587

231	1	0.2	0	4	
424	1	0.2	0	4	
581	7	1.4	0	2	<<<
756	1	0.2	0	4	
759	2	0.4	0	3	
763	1	0.2	0	4	
781	1	0.2	0	4	
785	1	0.2	0	4	
813	1	0.2	0	4	
827	1	0.2	0	4	
899	1	0.2	0	4	
985	1	1	1	4	
1171	1	1	1	4	
1212	1	1	1	4	
1215	1	1	1	4	

1216	2	2	2	2
1217	1	0.5	0	4
1220	4	4	4	0
1237	1	1	1	4
1281	2	2	2	2
1288	1	1	1	4
1319	1	1	1	4
1341	2	2	2	2
1347	1	1	1	4
1354	1	1	1	4
1366	1	1	1	4
1368	1	1	1	4
1378	1	1	1	4
1416	1	1	1	4
1425	1	1	1	4

---

category=0, cleavage_site=770
query=ptc-miR396a,b, target=Potri.002G115100.1,
score=3.5, range=759-780, strand=1

```
target  5' UCGUUCAAGAAaGCCUGUGGAA 3'
           . ::::::::::: ::::::::
query   3' GUCAAGUUCUUUC-GACACCUU 5'
```

>Potri.002G115100.1
#size=1404

770	2	1	0	0	<<<
1233	1	0.5	0	4	

---

category=0, p-value=0.0513594494699684, cleavage_site=411
query=ptc-miR396a,b, target=Potri.003G100800.1,
score=3, range=400-421, strand=1

```
target  5' CCGUUCAAGAAaGCCUGUGGAA 3'
           : ::::::::::: ::::::::
query   3' GUCAAGUUCUUUC-GACACCUU 5'
```

>Potri.003G100800.1
#size=1449

61	1	0.2	0	4	
254	1	0.2	0	4	
411	9	1.9	0	0	<<<
439	1	0.25	0	4	
565	1	0.2	0	4	
568	2	0.4	0	2	
572	1	0.2	0	4	
590	1	0.2	0	4	
594	1	0.2	0	4	
622	1	0.2	0	4	
636	1	0.2	0	4	
708	1	0.2	0	4	
1203	1	0.25	0	4	
1302	1	0.25	0	4	
1305	1	0.25	0	4	

---

category=0, cleavage_site=1114
query=ptc-miR396a,b, target=Potri.007G007100.1,
score=3.5, range=1103-1124, strand=1

```
target  5' UCGUUCAAGAAaGCCUGUGGAA 3'
           . ::::::::::: ::::::::
query   3' GUCAAGUUCUUUC-GACACCUU 5'
```

>Potri.007G007100.1

#size=2403

1114	4	2	0	0	<<<
1130	1	0.5	0	4	
1294	1	1	1	4	
1314	1	1	1	4	
1471	1	0.5	0	4	
1825	1	0.5	0	4	
1834	1	0.5	0	4	
1847	1	0.5	0	4	
1902	1	1	1	4	
1985	1	1	1	4	

---

category=4, cleavage_site=1052
query=ptc-miR396a,b, target=Potri.013G077500.1,
score=3, range=1041-1062, strand=1

```
target  5' CCGUUCAAGAAaGCCUGUGGAA 3'
            : ::::::::::: ::::::::
query   3' GUCAAGUUCUUUC-GACACCUU 5'
```

>Potri.013G077500.1
#size=2145

4	1	1	1	4	
1052	1	0.5	0	4	<<<
1226	1	1	1	4	
1429	1	0.5	0	4	
1594	1	1	1	4	
1628	1	1	1	4	
1845	3	3	3	0	
1846	1	1	1	4	

---

category=0, cleavage_site=1087
query=ptc-miR396a,b, target=Potri.014G007200.1,
score=3.5, range=1076-1097, strand=1

```
target  5' UCGUUCAAGAAaGCCUGUGGAA 3'
            . ::::::::::: ::::::::
query   3' GUCAAGUUCUUUC-GACACCUU 5'
```

>Potri.014G007200.1
#size=2360

383	1	1	1	4	
1087	4	2	0	0	<<<
1103	1	0.5	0	4	
1444	1	0.5	0	4	
1798	1	0.5	0	4	
1807	1	0.5	0	4	
1820	1	0.5	0	4	

---

category=1, cleavage_site=1045
query=ptc-miR396a,b, target=Potri.014G012800.1,
score=3.5, range=1034-1055, strand=1

```
target  5' UCGUUCAAGAAaGCCUGUGGAA 3'
            . ::::::::::: ::::::::
query   3' GUCAAGUUCUUUC-GACACCUU 5'
```

>Potri.014G012800.1
#size=2421

384	1	1	1	4	
1045	2	1	0	1	<<<
1155	1	1	1	4	
1505	1	0.5	0	4	
2127	1	1	1	4	

2148	1	1	1	4
2157	1	1	1	4

category=0, cleavage_site=587
query=ptc-miR396a,b, target=Potri.014G071800.1,
score=4, range=576-597, strand=1

```
target  5' ACGUUCAAGAAaGCUUGUGGAA 3'
             :::::::::::: :::::::
query   3' GUCAAGUUCUUUCG-ACACCUU 5'
```

>Potri.014G071800.1
#size=1776

587	5	5	5	0	<<<
665	1	1	1	4	
700	1	1	1	4	
721	1	1	1	4	
809	2	2	2	2	
830	1	1	1	4	
833	1	1	1	4	
837	1	1	1	4	
942	1	1	1	4	
1002	1	1	1	4	
1078	1	1	1	4	
1310	1	1	1	4	
1424	1	1	1	4	
1527	1	1	1	4	
1544	1	1	1	4	
1575	1	1	1	4	
1577	1	1	1	4	

category=4, cleavage_site=398
query=ptc-miR396a,b, target=Potri.015G006200.1,
score=3, range=387-408, strand=1

```
target  5' CCGUUCAAGAAaGCAUGUGGAA 3'
           : :::::::::::: :::::::
query   3' GUCAAGUUCUUUCG-ACACCUU 5'
```

>Potri.015G006200.1
#size=1554

398	1	0.2	0	4	<<<
1337	1	0.2	0	4	

ptc-miR396c-e

category=4, cleavage_site=817
query=ptc-miR396c-e, target=Potri.001G082700.1,
score=4, range=806-827, strand=1

```
target  5' CCGUUCAAGAAaGCCUGUGGAA 3'
             ::::::::::: ::::::::
query   3' UUCAAGUUCUUUC-GACACCUU 5'
```

>Potri.001G082700.1
#size=1956

817	1	1	1	4	<<<
1562	1	1	1	4	

category=0, cleavage_site=681
query=ptc-miR396c-e, target=Potri.001G114000.1,
score=3.5, range=670-691, strand=1

```
target  5' GCGUUCAAGAAaGCUUGUGGAA 3'
            . :::::::::::: :::::::
query   3' UUCAAGUUCUUUCG-ACACCUU 5'
```

>Potri.001G114000.1
#size=1087

109	1	0.333333333333333	0	4	
357	1	0.333333333333333	0	4	
465	2	0.5	0	2	
530	1	0.5	0	4	
587	1	0.5	0	4	
673	1	0.5	0	4	
681	50	25	0	0	<<<
682	1	0.5	0	4	
697	1	0.5	0	4	
708	1	0.5	0	4	
715	1	0.5	0	4	
723	1	0.5	0	4	
755	1	0.5	0	4	

category=2, cleavage_site=581
query=ptc-miR396c-e, target=Potri.001G132600.1,
score=4, range=570-591, strand=1

```
target  5' CCGUUCAAGAAaGCCUGUGGAA 3'
              ::::::::::: :::::::
query   3' UUCAAGUUCUUUC-GACACCUU 5'
```

>Potri.001G132600.1
#size=1587

231	1	0.2	0	4	
424	1	0.2	0	4	
581	7	1.4	0	2	<<<
756	1	0.2	0	4	
759	2	0.4	0	3	
763	1	0.2	0	4	
781	1	0.2	0	4	
785	1	0.2	0	4	
813	1	0.2	0	4	
827	1	0.2	0	4	
899	1	0.2	0	4	
985	1	1	1	4	
1171	1	1	1	4	
1212	1	1	1	4	
1215	1	1	1	4	
1216	2	2	2	2	
1217	1	0.5	0	4	
1220	4	4	4	0	
1237	1	1	1	4	
1281	2	2	2	2	
1288	1	1	1	4	
1319	1	1	1	4	
1341	2	2	2	2	
1347	1	1	1	4	
1354	1	1	1	4	
1366	1	1	1	4	
1368	1	1	1	4	

1378	1	1	1	4	
1416	1	1	1	4	
1425	1	1	1	4	

---

category=0, cleavage_site=770
query=ptc-miR396c-e, target=Potri.002G115100.1,
score=4, range=759-780, strand=1

```
target  5' UCGUUCAAGAAaGCCUGUGGAA 3'
              ::::::::::: ::::::::
query   3' UUCAAGUUCUUUC-GACACCUU 5'
```

>Potri.002G115100.1
#size=1404

770	2	1	0	0	<<<
1233	1	0.5	0	4	

---

category=0, cleavage_site=411
query=ptc-miR396c-e, target=Potri.003G100800.1,
score=4, range=400-421, strand=1

```
target  5' CCGUUCAAGAAaGCCUGUGGAA 3'
              ::::::::::: ::::::::
query   3' UUCAAGUUCUUUC-GACACCUU 5'
```

>Potri.003G100800.1
#size=1449

61	1	0.2	0	4	
254	1	0.2	0	4	
411	9	1.9	0	0	<<<
439	1	0.25	0	4	
565	1	0.2	0	4	
568	2	0.4	0	2	
572	1	0.2	0	4	
590	1	0.2	0	4	
594	1	0.2	0	4	
622	1	0.2	0	4	
636	1	0.2	0	4	
708	1	0.2	0	4	
1203	1	0.25	0	4	
1302	1	0.25	0	4	
1305	1	0.25	0	4	

---

category=0, cleavage_site=1114
query=ptc-miR396c-e, target=Potri.007G007100.1,
score=4, range=1103-1124, strand=1

```
target  5' UCGUUCAAGAAaGCCUGUGGAA 3'
              ::::::::::: ::::::::
query   3' UUCAAGUUCUUUC-GACACCUU 5'
```

>Potri.007G007100.1
#size=2403

1114	4	2	0	0	<<<
1130	1	0.5	0	4	
1294	1	1	1	4	
1314	1	1	1	4	
1471	1	0.5	0	4	
1825	1	0.5	0	4	
1834	1	0.5	0	4	
1847	1	0.5	0	4	
1902	1	1	1	4	
1985	1	1	1	4	

category=0, cleavage_site=1087
query=ptc-miR396c-e, target=Potri.014G007200.1,
score=4, range=1076-1097, strand=1

```
target  5' UCGUUCAAGAAaGCCUGUGGAA 3'
             ::::::::::: ::::::::
query   3' UUCAAGUUCUUUC-GACACCUU 5'
```

>Potri.014G007200.1
#size=2360

383	1	1	1	4	
1087	4	2	0	0	<<<
1103	1	0.5	0	4	
1444	1	0.5	0	4	
1798	1	0.5	0	4	
1807	1	0.5	0	4	
1820	1	0.5	0	4	

category=1, cleavage_site=1045
query=ptc-miR396c-e, target=Potri.014G012800.1,
score=4, range=1034-1055, strand=1

```
target  5' UCGUUCAAGAAaGCCUGUGGAA 3'
             ::::::::::: ::::::::
query   3' UUCAAGUUCUUUC-GACACCUU 5'
```

>Potri.014G012800.1
#size=2421

384	1	1	1	4	
1045	2	1	0	1	<<<
1155	1	1	1	4	
1505	1	0.5	0	4	
2127	1	1	1	4	
2148	1	1	1	4	
2157	1	1	1	4	

category=0, cleavage_site=587
query=ptc-miR396c-e, target=Potri.014G071800.1,
score=3, range=576-597, strand=1

```
target  5' ACGUUCAAGAAaGCUUGUGGAA 3'
           : :::::::::::: :::::::
query   3' UUCAAGUUCUUUCG-ACACCUU 5'
```

>Potri.014G071800.1
#size=1776

587	5	5	5	0	<<<
665	1	1	1	4	
700	1	1	1	4	
721	1	1	1	4	
809	2	2	2	2	
830	1	1	1	4	
833	1	1	1	4	
837	1	1	1	4	
942	1	1	1	4	
1002	1	1	1	4	
1078	1	1	1	4	
1310	1	1	1	4	
1424	1	1	1	4	
1527	1	1	1	4	
1544	1	1	1	4	
1575	1	1	1	4	
1577	1	1	1	4	

category=4, cleavage_site=398
query=ptc-miR396c-e, target=Potri.015G006200.1,
score=4, range=387-408, strand=1

```
target  5' CCGUUCAAGAAaGCAUGUGGAA 3'
              ::::::::::: :::::::
query   3' UUCAAGUUCUUUCG-ACACCUU 5'
```

>Potri.015G006200.1
#size=1554

398	1	0.2	0	4	<<<
1337	1	0.2	0	4	

ptc-miR396f,g

category=4, cleavage_site=817
query=ptc-miR396f,g, target=Potri.001G082700.1,
score=3, range=806-827, strand=1

```
target  5' CCGUUCAAGAAaGCCUGUGGAA 3'
             : :::::::::::: ::::::
query   3' GUCAAGUUCUUUCGG-CACCUU 5'
```

>Potri.001G082700.1
#size=1956

817	1	1	1	4	<<<
1562	1	1	1	4	

category=2, cleavage_site=581
query=ptc-miR396f,g, target=Potri.001G132600.1,
score=3, range=570-591, strand=1

```
target  5' CCGUUCAAGAAaGCCUGUGGAA 3'
             : :::::::::::: ::::::
query   3' GUCAAGUUCUUUCGG-CACCUU 5'
```

>Potri.001G132600.1
#size=1587

231	1	0.2	0	4	
424	1	0.2	0	4	
581	7	1.4	0	2	<<<
756	1	0.2	0	4	
759	2	0.4	0	3	
763	1	0.2	0	4	
781	1	0.2	0	4	
785	1	0.2	0	4	
813	1	0.2	0	4	
827	1	0.2	0	4	
899	1	0.2	0	4	
985	1	1	1	4	
1171	1	1	1	4	
1212	1	1	1	4	
1215	1	1	1	4	
1216	2	2	2	2	
1217	1	0.5	0	4	
1220	4	4	4	0	
1237	1	1	1	4	
1281	2	2	2	2	
1288	1	1	1	4	
1319	1	1	1	4	
1341	2	2	2	2	
1347	1	1	1	4	
1354	1	1	1	4	

1366	1	1	1	4
1368	1	1	1	4
1378	1	1	1	4
1416	1	1	1	4
1425	1	1	1	4

---

category=0, cleavage_site=770
query=ptc-miR396f,g, target=Potri.002G115100.1,
score=3.5, range=759-780, strand=1

```
target  5' UCGUUCAAGAAaGCCUGUGGAA 3'
            . ::::::::::::: ::::::
query   3' GUCAAGUUCUUUCGG-CACCUU 5'
```

>Potri.002G115100.1
#size=1404

770	2	1	0	0	<<<
1233	1	0.5	0	4	

---

category=0, cleavage_site=411
query=ptc-miR396f,g, target=Potri.003G100800.1,
score=3, range=400-421, strand=1

```
target  5' CCGUUCAAGAAaGCCUGUGGAA 3'
            : ::::::::::::: ::::::
query   3' GUCAAGUUCUUUCGG-CACCUU 5'
```

>Potri.003G100800.1
#size=1449

61	1	0.2	0	4	
254	1	0.2	0	4	
411	9	1.9	0	0	<<<
439	1	0.25	0	4	
565	1	0.2	0	4	
568	2	0.4	0	2	
572	1	0.2	0	4	
590	1	0.2	0	4	
594	1	0.2	0	4	
622	1	0.2	0	4	
636	1	0.2	0	4	
708	1	0.2	0	4	
1203	1	0.25	0	4	
1302	1	0.25	0	4	
1305	1	0.25	0	4	

---

category=4, cleavage_site=674
query=ptc-miR396f,g, target=Potri.006G143200.1,
score=3, range=663-684, strand=1

```
target  5' CCGUUCAAGAAaGCCUGUGGAA 3'
            : ::::::::::::: ::::::
query   3' GUCAAGUUCUUUCGG-CACCUU 5'
```

>Potri.006G143200.1
#size=1981

635	1	0.5	0	4	
674	1	1	1	4	<<<

---

category=0, cleavage_site=1114
query=ptc-miR396f,g, target=Potri.007G007100.1,
score=3.5, range=1103-1124, strand=1

```
target  5' UCGUUCAAGAAaGCCUGUGGAA 3'
            . ::::::::::::: ::::::
query   3' GUCAAGUUCUUUCGG-CACCUU 5'
```

>Potri.007G007100.1
#size=2403

1114	4	2	0	0	<<<
1130	1	0.5	0	4	
1294	1	1	1	4	
1314	1	1	1	4	
1471	1	0.5	0	4	
1825	1	0.5	0	4	
1834	1	0.5	0	4	
1847	1	0.5	0	4	
1902	1	1	1	4	
1985	1	1	1	4	

---

category=4, cleavage_site=1052
query=ptc-miR396f,g, target=Potri.013G077500.1,
score=3, range=1041-1062, strand=1

```
target  5' CCGUUCAAGAAaGCCUGUGGAA 3'
            : ::::::::::::: ::::::
query   3' GUCAAGUUCUUUCGG-CACCUU 5'
```

>Potri.013G077500.1
#size=2145

4	1	1	1	4	
1052	1	0.5	0	4	<<<
1226	1	1	1	4	
1429	1	0.5	0	4	
1594	1	1	1	4	
1628	1	1	1	4	
1845	3	3	3	0	
1846	1	1	1	4	

---

category=0, cleavage_site=1087
query=ptc-miR396f,g, target=Potri.014G007200.1,
score=3.5, range=1076-1097, strand=1

```
target  5' UCGUUCAAGAAaGCCUGUGGAA 3'
            . ::::::::::::: ::::::
query   3' GUCAAGUUCUUUCGG-CACCUU 5'
```

>Potri.014G007200.1
#size=2360

383	1	1	1	4	
1087	4	2	0	0	<<<
1103	1	0.5	0	4	
1444	1	0.5	0	4	
1798	1	0.5	0	4	
1807	1	0.5	0	4	
1820	1	0.5	0	4	

---

category=1, cleavage_site=1045
query=ptc-miR396f,g, target=Potri.014G012800.1,
score=3.5, range=1034-1055, strand=1

```
target  5' UCGUUCAAGAAaGCCUGUGGAA 3'
            . ::::::::::::: ::::::
query   3' GUCAAGUUCUUUCGG-CACCUU 5'
```

>Potri.014G012800.1
#size=2421

384	1	1	1	4	
1045	2	1	0	1	<<<
1155	1	1	1	4	
1505	1	0.5	0	4	

2127	1	1	1	4
2148	1	1	1	4
2157	1	1	1	4

---

category=4, cleavage_site=398
query=ptc-miR396f,g, target=Potri.015G006200.1,
score=4, range=387-408, strand=1

```
target  5' CCGUUCAAGAAaGCAUGUGGAA 3'
           : ::::::::::: .::::::
query   3' GUCAAGUUCUUUCG-GCACCUU 5'
```

>Potri.015G006200.1
#size=1554

398	1	0.2	0	4

ptc-miR397a

---

category=0, cleavage_site=1089
query=ptc-miR397a, target=Potri.001G180600.1,
score=3.5, range=1078-1098, strand=1

```
target  5' CAUCAAUUUUGuACUCAAUGA 3'
           ::::::. .::.::::::::
query   3' GUAGUUGCGACGUGAGUUACU 5'
```

>Potri.001G180600.1
#size=1161

513	1	0.333333333333333	0	4	
714	1	0.333333333333333	0	4	
718	4	1.33333333333333	0	2	
722	1	0.5	0	4	
765	1	0.333333333333333	0	4	
817	1	0.333333333333333	0	4	
825	1	0.333333333333333	0	4	
827	2	0.666666666666667	0	2	
936	2	1	0	2	
961	1	0.5	0	4	
1059	1	0.5	0	4	
1065	1	0.5	0	4	
1066	3	1.5	0	2	
1067	2	1	0	2	
1081	1	0.5	0	4	
1085	1	0.5	0	4	
1089	7	3.5	0	0	<<<
1090	2	1	0	2	
1091	3	1.5	0	2	
1093	1	0.5	0	4	
1096	1	0.5	0	4	
1097	4	2	0	2	
1098	1	0.5	0	4	
1099	2	1	0	2	
1102	1	0.5	0	4	
1108	1	0.5	0	4	

---

category=4, cleavage_site=695
query=ptc-miR397a, target=Potri.006G094100.1,
score=3, range=684-704, strand=1

```
target  5' AAUCAACGCUGcACUCAAUAA 3'
             ::::::::::::::::: :
query   3' GUAGUUGCGACGUGAGUUACU 5'
```

>Potri.006G094100.1

#size=1987

695	1	1	1	4	<<<
1510	1	1	1	4	
1598	1	1	1	4	

---

category=3, cleavage_site=772
query=ptc-miR397a, target=Potri.008G073700.1,
score=2.5, range=761-781, strand=1

```
target  5' CAUCAAUGCUGcACUCAAUCA 3'
           ::::::.::::::::::::: :
query   3' GUAGUUGCGACGUGAGUUACU 5'
```

>Potri.008G073700.1
#size=2090

101	1	1	1	4	
137	1	1	1	4	
154	1	1	1	4	
203	1	0.5	0	4	
220	1	1	1	4	
241	1	1	1	4	
333	1	0.5	0	4	
342	1	0.2	0	4	
415	1	0.333333333333333	0	4	
427	1	0.333333333333333	0	4	
433	1	1	1	4	
434	1	0.333333333333333	0	4	
445	1	0.333333333333333	0	4	
479	1	0.1	0	4	
482	1	0.142857142857143	0	4	
494	1	0.2	0	4	
495	1	0.2	0	4	
515	1	1	1	4	
523	1	1	1	4	
526	1	1	1	4	
595	1	1	1	4	
610	1	1	1	4	
650	1	0.333333333333333	0	4	
651	1	0.333333333333333	0	4	
655	1	0.2	0	4	
694	1	1	1	4	
717	1	0.333333333333333	0	4	
719	1	0.333333333333333	0	4	
725	1	0.333333333333333	0	4	
733	2	0.666666666666667	0	3	
735	1	0.333333333333333	0	4	
743	1	0.333333333333333	0	4	
744	1	0.333333333333333	0	4	
748	1	0.333333333333333	0	4	
754	1	0.333333333333333	0	4	
757	1	0.333333333333333	0	4	
766	1	0.333333333333333	0	4	
769	1	0.333333333333333	0	4	
772	2	0.666666666666667	0	3	<<<
858	1	1	1	4	
871	1	1	1	4	
876	1	1	1	4	
877	1	1	1	4	
883	1	0.333333333333333	0	4	
889	1	0.333333333333333	0	4	

941	1	1	1	4		
946	1	0.333333333333333			0	4
947	1	0.333333333333333			0	4
948	2	0.458333333333333			0	3
951	2	0.666666666666667			0	3
958	1	0.333333333333333			0	4
972	3	1	0	2		
974	2	0.666666666666667			0	3
977	2	0.533333333333333			0	3
978	1	0.1	0	4		
979	1	0.2	0	4		
980	1	0.2	0	4		
981	1	0.2	0	4		
982	3	1	0	2		
995	1	0.333333333333333			0	4
1009	1	1	1	4		
1065	1	1	1	4		
1068	1	1	1	4		
1126	1	1	1	4		
1149	2	2	2	1		
1175	1	1	1	4		
1209	1	0.333333333333333			0	4
1214	1	0.333333333333333			0	4
1216	2	0.666666666666667			0	3
1222	1	0.333333333333333			0	4
1256	1	1	1	4		
1259	1	1	1	4		
1260	1	1	1	4		
1280	1	1	1	4		
1302	1	1	1	4		
1345	2	2	2	1		
1354	1	1	1	4		
1356	1	1	1	4		
1373	1	1	1	4		
1387	1	0.333333333333333			0	4
1401	1	1	1	4		
1404	2	0.666666666666667			0	3
1406	1	0.333333333333333			0	4
1423	2	0.666666666666667			0	3
1426	1	0.333333333333333			0	4
1443	1	0.2	0	4		
1458	2	0.4	0	3		
1460	1	0.2	0	4		
1543	1	1	1	4		
1548	1	1	1	4		
1564	1	1	1	4		
1575	1	1	1	4		
1587	2	0.666666666666667			0	3
1597	1	1	1	4		
1610	1	1	1	4		
1615	2	2	2	1		
1621	1	1	1	4		
1626	2	2	2	1		
1627	1	1	1	4		
1644	1	0.333333333333333			0	4
1648	1	0.333333333333333			0	4
1653	1	0.333333333333333			0	4
1655	1	0.333333333333333			0	4

1656	1	0.333333333333333	0	4
1660	1	0.333333333333333	0	4
1662	1	0.2	0	4
1664	1	0.333333333333333	0	4
1666	4	1.06666666666667	0	2
1667	1	0.2	0	4
1668	1	0.2	0	4
1679	1	0.333333333333333	0	4
1688	2	0.666666666666667	0	3
1691	1	0.333333333333333	0	4
1702	1	1	1	4
1705	1	1	1	4
1709	1	1	1	4
1718	1	1	1	4
1730	1	1	1	4
1731	1	1	1	4
1740	1	1	1	4
1742	2	2	2	1
1748	2	2	2	1
1751	2	2	2	1
1753	1	1	1	4
1754	1	1	1	4
1756	1	1	1	4
1758	1	1	1	4
1764	1	1	1	4
1785	1	1	1	4
1792	1	1	1	4
1804	1	1	1	4
1810	1	1	1	4
1814	2	2	2	1
1815	1	1	1	4
1819	1	1	1	4
1820	2	2	2	1
1824	1	1	1	4
1825	1	1	1	4
1840	1	1	1	4
1843	1	1	1	4
1844	1	1	1	4
1845	1	1	1	4
1855	1	1	1	4
1878	1	1	1	4
1883	1	1	1	4
1887	1	1	1	4
1890	1	1	1	4
1904	1	1	1	4
1936	1	1	1	4

---

category=2, cleavage_site=762
query=ptc-miR397a, target=Potri.010G183600.1,
score=2.5, range=751-771, strand=1

```
target  5' CAUCAAUGCUGcACUCAAUCA 3'
           ::::::.::::::::::: :
query   3' GUAGUUGCGACGUGAGUUACU 5'
```

>Potri.010G183600.1
#size=2115

32	1	1	1	4
94	1	1	1	4
138	2	2	2	1

193	1	0.5	0	4	
230	1	1	1	4	
253	1	1	1	4	
323	1	0.5	0	4	
332	1	0.2	0	4	
361	1	0.5	0	4	
368	1	0.5	0	4	
370	1	0.5	0	4	
381	1	0.5	0	4	
401	1	0.5	0	4	
405	1	0.333333333333333	0	4	
417	1	0.333333333333333	0	4	
424	1	0.333333333333333	0	4	
435	1	0.333333333333333	0	4	
469	1	0.1	0	4	
472	1	0.142857142857143	0	4	
474	1	0.25	0	4	
484	1	0.2	0	4	
485	1	0.2	0	4	
505	1	0.5	0	4	
508	1	0.5	0	4	
509	1	0.5	0	4	
511	1	0.5	0	4	
512	1	0.5	0	4	
513	1	0.5	0	4	
516	1	0.5	0	4	
525	1	0.5	0	4	
538	1	0.5	0	4	
552	2	1	0	2	
561	1	0.5	0	4	
579	1	0.5	0	4	
583	1	0.5	0	4	
599	1	0.5	0	4	
612	1	0.5	0	4	
629	1	0.5	0	4	
636	1	0.5	0	4	
640	1	0.333333333333333	0	4	
641	1	0.333333333333333	0	4	
645	1	0.2	0	4	
650	1	0.5	0	4	
659	1	0.5	0	4	
669	1	0.5	0	4	
679	1	0.5	0	4	
707	1	0.333333333333333	0	4	
709	1	0.333333333333333	0	4	
715	1	0.333333333333333	0	4	
723	3	1.16666666666667	0	2	
725	1	0.333333333333333	0	4	
733	1	0.333333333333333	0	4	
734	1	0.333333333333333	0	4	
738	1	0.333333333333333	0	4	
744	1	0.333333333333333	0	4	
747	1	0.333333333333333	0	4	
756	1	0.333333333333333	0	4	
759	1	0.333333333333333	0	4	
762	2	0.666666666666667	0	2	<<<
772	1	0.5	0	4	
774	1	0.5	0	4	

842	1	0.5	0	4
863	1	0.5	0	4
873	1	0.333333333333333	0	4
877	1	0.5	0	4
879	1	0.333333333333333	0	4
882	1	0.5	0	4
888	1	0.5	0	4
889	1	0.5	0	4
897	1	0.5	0	4
901	1	0.5	0	4
903	1	0.5	0	4
924	2	1	0	2
927	1	0.5	0	4
936	1	0.333333333333333	0	4
937	1	0.333333333333333	0	4
938	1	0.333333333333333	0	4
941	2	0.666666666666667	0	2
948	1	0.333333333333333	0	4
962	3	1	0	2
964	1	0.333333333333333	0	4
967	2	0.533333333333333	0	2
968	1	0.1	0	4
969	1	0.2	0	4
970	1	0.2	0	4
971	1	0.2	0	4
972	3	1	0	2
978	1	0.5	0	4
985	1	0.333333333333333	0	4
1009	1	0.5	0	4
1015	1	0.5	0	4
1020	1	0.5	0	4
1027	1	0.5	0	4
1035	1	0.5	0	4
1037	1	0.5	0	4
1038	1	0.5	0	4
1043	1	0.5	0	4
1046	1	0.5	0	4
1050	1	0.5	0	4
1051	1	0.5	0	4
1053	1	0.5	0	4
1056	1	0.5	0	4
1061	1	0.5	0	4
1062	1	0.5	0	4
1095	1	0.5	0	4
1102	2	1	0	2
1115	1	0.5	0	4
1116	1	0.5	0	4
1144	1	0.5	0	4
1154	1	0.5	0	4
1156	1	0.5	0	4
1159	1	0.5	0	4
1166	1	0.5	0	4
1173	1	0.5	0	4
1199	1	0.333333333333333	0	4
1204	1	0.333333333333333	0	4
1206	2	0.666666666666667	0	2
1212	1	0.333333333333333	0	4
1231	1	0.5	0	4

1232	1	0.5	0	4
1235	1	0.5	0	4
1243	1	0.5	0	4
1259	1	0.5	0	4
1266	1	0.5	0	4
1268	1	0.5	0	4
1269	1	0.5	0	4
1270	1	0.5	0	4
1273	1	0.5	0	4
1275	1	0.5	0	4
1276	1	0.5	0	4
1277	1	0.5	0	4
1278	1	0.5	0	4
1282	1	0.5	0	4
1287	1	0.5	0	4
1289	2	1	0	2
1295	2	1	0	2
1353	1	0.5	0	4
1354	1	0.5	0	4
1356	1	0.142857142857143	0	4
1377	1	0.333333333333333	0	4
1383	1	0.5	0	4
1394	2	0.666666666666667	0	2
1396	1	0.333333333333333	0	4
1413	2	0.666666666666667	0	2
1416	1	0.333333333333333	0	4
1422	1	0.5	0	4
1427	1	0.5	0	4
1428	1	0.5	0	4
1431	1	0.5	0	4
1432	1	0.5	0	4
1433	1	0.2	0	4
1434	1	0.5	0	4
1448	2	0.4	0	3
1450	1	0.2	0	4
1459	1	0.111111111111111	0	4
1461	1	0.142857142857143	0	4
1464	1	0.111111111111111	0	4
1469	2	0.222222222222222	0	3
1470	1	0.111111111111111	0	4
1472	2	0.285714285714286	0	3
1482	2	0.222222222222222	0	3
1536	2	1	0	2
1538	1	0.5	0	4
1546	1	0.5	0	4
1548	1	0.5	0	4
1552	1	0.5	0	4
1560	1	0.5	0	4
1577	2	0.666666666666667	0	2
1578	1	0.5	0	4
1581	1	0.5	0	4
1582	1	0.5	0	4
1584	2	1	0	2
1585	2	1	0	2
1594	1	0.5	0	4
1599	1	0.5	0	4
1605	1	0.5	0	4
1607	1	0.5	0	4

1608	1	0.5	0	4
1609	4	2	0	1
1614	2	1	0	2
1616	1	0.25	0	4
1617	1	0.5	0	4
1625	1	0.5	0	4
1634	1	0.333333333333333	0	4
1638	1	0.333333333333333	0	4
1643	1	0.333333333333333	0	4
1645	1	0.333333333333333	0	4
1646	1	0.333333333333333	0	4
1650	1	0.333333333333333	0	4
1652	1	0.2	0	4
1654	1	0.333333333333333	0	4
1656	4	1.06666666666667	0	2
1657	1	0.2	0	4
1658	1	0.2	0	4
1669	1	0.333333333333333	0	4
1678	2	0.666666666666667	0	2
1681	1	0.333333333333333	0	4
1694	2	1	0	2
1695	1	0.5	0	4
1707	1	0.5	0	4
1708	1	0.5	0	4
1710	1	0.5	0	4
1712	1	0.5	0	4
1714	1	0.5	0	4
1715	2	1	0	2
1716	1	0.5	0	4
1718	2	1	0	2
1723	2	1	0	2
1724	2	1	0	2
1725	3	1.5	0	2
1727	2	1	0	2
1728	1	0.5	0	4
1730	1	0.5	0	4
1731	3	1.5	0	2
1733	1	0.5	0	4
1735	1	0.5	0	4
1736	3	1.5	0	2
1737	1	0.5	0	4
1738	1	0.5	0	4
1741	1	0.5	0	4
1742	1	0.5	0	4
1743	1	0.5	0	4
1744	1	0.5	0	4
1747	2	1	0	2
1748	1	0.5	0	4
1750	2	1	0	2
1755	2	2	2	1
1761	1	1	1	4
1769	1	1	1	4
1772	1	1	1	4
1819	1	1	1	4
1824	1	1	1	4
1831	1	1	1	4
1834	1	1	1	4
1890	1	1	1	4

1927	1	1	1	4
1975	1	1	1	4
2019	1	1	1	4
2023	1	1	1	4

ptc-miR472b

category=0, cleavage_site=842
query=ptc-miR472b, target=Potri.001G028700.1,
score=3, range=830-851, strand=1

```
target  5' GGGUUUGGUGGAgUUGGGAAGA 3'
           ::: : ::::::::::::::.:
query   3' CCCUACCCACCUCAACCCUUUU 5'
```

>Potri.001G028700.1
#size=2611

481	1	1	1	4	
578	1	1	1	4	
840	2	2	2	2	
842	3	3	3	0	<<<
873	1	1	1	4	
1290	1	1	1	4	
1406	1	1	1	4	
1527	1	1	1	4	
1657	1	1	1	4	
2013	1	1	1	4	
2293	1	1	1	4	
2533	1	1	1	4	

category=0, cleavage_site=676
query=ptc-miR472b, target=Potri.001G363400.1,
score=3, range=664-685, strand=1

```
target  5' GGAAUUGGUGGAgUUGGGAAGA 3'
           :: :: ::::::::::::::.:
query   3' CCCUACCCACCUCAACCCUUUU 5'
```

>Potri.001G363400.1
#size=1899

35	1	0.1	0	4
42	1	0.1	0	4
43	1	0.1	0	4
46	1	0.1	0	4
60	1	0.1	0	4
100	1	0.166666666666667	0	4
105	2	0.333333333333333	0	2
117	1	0.142857142857143	0	4
142	1	0.1	0	4
149	1	0.1	0	4
152	1	0.1	0	4
154	1	0.1	0	4
155	1	0.1	0	4
156	1	0.333333333333333	0	4
158	2	0.333333333333333	0	2
159	1	0.1	0	4
166	1	0.1	0	4
167	1	0.1	0	4
171	1	0.1	0	4
172	1	0.1	0	4
180	1	0.1	0	4
204	1	0.1	0	4

210	1	0.1	0	4	
214	1	0.1	0	4	
225	1	0.1	0	4	
241	1	0.1	0	4	
246	1	0.1	0	4	
255	2	0.2	0	2	
256	1	0.1	0	4	
378	2	0.2	0	2	
379	1	0.1	0	4	
381	1	0.1	0	4	
408	2	0.2	0	2	
461	1	0.1	0	4	
464	1	0.1	0	4	
468	1	0.1	0	4	
476	1	0.1	0	4	
479	1	0.1	0	4	
494	1	0.1	0	4	
496	1	0.1	0	4	
500	1	0.1	0	4	
518	1	0.142857142857143	0	4	
598	1	0.1	0	4	
648	1	0.111111111111111	0	4	
673	1	0.125	0	4	
674	2	0.2	0	2	
675	7	0.7	0	2	
676	10	1.125	0	0	<<<
727	1	0.111111111111111	0	4	
807	1	0.333333333333333	0	4	
810	1	0.333333333333333	0	4	
814	1	0.333333333333333	0	4	
820	1	0.1	0	4	
831	2	0.225	0	2	
854	2	0.25	0	2	
861	3	0.75	0	2	
863	2	0.5	0	2	
928	1	0.111111111111111	0	4	
929	2	0.222222222222222	0	2	
960	1	0.111111111111111	0	4	
1003	2	0.5	0	2	
1019	1	0.1	0	4	
1175	1	0.1	0	4	
1177	1	0.1	0	4	
1188	1	0.1	0	4	
1199	1	0.166666666666667	0	4	
1227	1	0.1	0	4	
1228	1	0.1	0	4	
1229	1	0.1	0	4	
1257	1	0.1	0	4	
1291	2	0.2	0	2	
1387	1	0.1	0	4	
1407	1	0.1	0	4	
1455	1	0.1	0	4	
1458	1	0.1	0	4	
1466	1	0.1	0	4	
1477	1	0.1	0	4	
1491	1	0.1	0	4	
1498	1	0.1	0	4	
1541	1	0.1	0	4	

1542	2	0.2	0	2
1556	1	0.1	0	4
1586	1	0.125	0	4
1712	1	0.166666666666667	0	4
1714	1	0.166666666666667	0	4

---

category=2, cleavage_site=472
query=ptc-miR472b, target=Potri.011G008600.1,
score=3, range=460-481, strand=1

```
target  5' GGAAUUGGUGGAgUUGGGAAGA 3'
           :: :: ::::::::::::::.:
query   3' CCCUACCCACCUCAACCCUUUU 5'
```

>Potri.011G008600.1
#size=3210

52	1	0.1	0	4	
55	1	0.1	0	4	
69	1	0.1	0	4	
86	1	0.1	0	4	
88	1	0.1	0	4	
93	1	0.1	0	4	
99	1	0.1	0	4	
101	1	0.1	0	4	
107	1	0.1	0	4	
169	1	0.1	0	4	
174	2	0.2	0	2	
183	1	0.1	0	4	
204	2	0.2	0	2	
232	1	0.1	0	4	
245	1	0.1	0	4	
249	1	0.1	0	4	
250	1	0.1	0	4	
257	1	0.1	0	4	
260	1	0.1	0	4	
264	1	0.1	0	4	
272	1	0.1	0	4	
314	1	0.142857142857143	0	4	
394	1	0.1	0	4	
469	1	0.125	0	4	
470	2	0.2	0	2	
471	7	0.7	0	2	
472	10	1.125	0	2	<<<
523	1	0.111111111111111	0	4	
567	2	0.222222222222222	0	2	
571	1	0.1	0	4	
579	1	0.125	0	4	
585	1	0.125	0	4	
587	1	0.125	0	4	
613	1	0.125	0	4	
619	1	0.1	0	4	
630	2	0.225	0	2	
646	1	0.125	0	4	
659	6	0.666666666666667	0	2	
660	4	0.444444444444444	0	2	
661	3	0.333333333333333	0	2	
662	82	9.11111111111109	0	0	
663	3	0.333333333333333	0	2	
670	1	0.1	0	4	
671	1	0.111111111111111	0	4	

679	1	0.1	0	4
680	1	0.125	0	4
693	1	0.1	0	4
699	1	0.111111111111111	0	4
727	1	0.111111111111111	0	4
728	2	0.222222222222222	0	2
736	1	0.1	0	4
751	1	0.125	0	4
809	1	0.333333333333333	0	4
818	1	0.1	0	4
849	1	0.1	0	4
893	1	0.1	0	4
927	1	0.142857142857143	0	4
932	1	0.1	0	4
974	1	0.1	0	4
987	1	0.1	0	4
1056	1	0.1	0	4
1125	1	0.166666666666667	0	4
1236	1	0.1	0	4
1254	1	0.1	0	4
1257	1	0.1	0	4
1265	1	0.1	0	4
1276	1	0.1	0	4
1290	1	0.1	0	4
1340	1	0.1	0	4
1341	2	0.2	0	2
1385	1	0.125	0	4
1604	1	0.166666666666667	0	4
1606	1	0.166666666666667	0	4
1685	1	0.111111111111111	0	4
1702	1	0.1	0	4
1751	2	0.242857142857143	0	2
1899	1	0.1	0	4
2242	1	0.1	0	4
2243	1	0.1	0	4
2507	1	0.125	0	4
2512	2	0.4	0	2
2627	1	0.111111111111111	0	4
2655	1	0.1	0	4
2747	1	1	1	4
2852	1	1	1	4
2884	1	1	1	4
2888	1	1	1	4
2892	1	1	1	4
2893	1	1	1	4
2894	2	1	0	2
2960	3	0.375	0	2
2967	2	0.225	0	2
2969	3	0.333333333333333	0	2
2970	2	0.236111111111111	0	2
2984	1	0.2	0	4
2987	2	0.2	0	2
2991	2	0.2	0	2
2992	1	0.1	0	4
2995	1	0.1	0	4
2996	1	0.1	0	4
2998	1	0.1	0	4
2999	1	0.1	0	4

3039	1	0.1	0	4	
3083	1	0.125	0	4	
3084	2	0.222222222222222	0	2	

---

category=0, cleavage_site=676
query=ptc-miR472b, target=Potri.011G009400.1,
score=3, range=664-685, strand=1

```
target  5' GGAAUUGGUGGAgUUGGGAAGA 3'
            :: :: ::::::::::::::.:
query   3' CCCUACCCACCUCAACCCUUUU 5'
```

>Potri.011G009400.1
#size=1560

241	2	0.2	0	2	
411	1	0.1	0	4	
598	1	0.1	0	4	
673	1	0.125	0	4	
674	2	0.2	0	2	
675	7	0.7	0	2	
676	10	1.125	0	0	<<<
786	1	0.111111111111111	0	4	
883	1	0.111111111111111	0	4	
1341	1	0.166666666666667	0	4	

---

category=2, cleavage_site=676
query=ptc-miR472b, target=Potri.011G060600.1,
score=3, range=664-685, strand=1

```
target  5' GGAAUUGGUGGAgUUGGGAAGA 3'
            :: :: ::::::::::::::.:
query   3' CCCUACCCACCUCAACCCUUUU 5'
```

>Potri.011G060600.1
#size=2748

46	1	0.1	0	4	
56	1	0.1	0	4	
117	1	0.142857142857143	0	4	
154	1	0.1	0	4	
167	1	0.1	0	4	
203	1	0.1	0	4	
204	2	0.2	0	2	
209	2	0.2	0	2	
248	1	0.1	0	4	
254	1	0.125	0	4	
259	2	0.2	0	2	
269	1	0.2	0	4	
402	2	0.285714285714286	0	2	
407	1	0.2	0	4	
409	2	1.1	1	2	
411	1	0.1	0	4	
436	1	0.1	0	4	
449	1	0.1	0	4	
454	1	0.1	0	4	
479	1	0.1	0	4	
518	1	0.142857142857143	0	4	
598	1	0.1	0	4	
648	1	0.111111111111111	0	4	
673	1	0.125	0	4	
674	2	0.2	0	2	
675	7	0.7	0	2	
676	10	1.125	0	2	<<<

727	1	0.111111111111111	0	4
783	1	0.125	0	4
789	1	0.125	0	4
791	1	0.125	0	4
817	1	0.125	0	4
823	1	0.1	0	4
834	2	0.225	0	2
850	1	0.125	0	4
863	4	0.444444444444444	0	2
864	6	0.666666666666667	0	2
865	3	0.333333333333333	0	2
866	84	9.33333333333332	0	0
867	4	0.444444444444444	0	2
872	1	0.111111111111111	0	4
874	3	0.336111111111111	0	2
883	1	0.1	0	4
884	1	0.125	0	4
897	1	0.1	0	4
903	1	0.111111111111111	0	4
931	1	0.111111111111111	0	4
932	2	0.222222222222222	0	2
955	1	0.125	0	4
1022	1	0.1	0	4
1053	1	0.1	0	4
1097	1	0.1	0	4
1131	1	0.142857142857143	0	4
1136	1	0.1	0	4
1178	1	0.1	0	4
1191	1	0.1	0	4
1329	1	0.166666666666667	0	4
1390	1	0.1	0	4
1440	1	0.1	0	4
1494	1	0.1	0	4
1501	1	0.1	0	4
1507	1	0.1	0	4
1517	1	0.1	0	4
1589	1	0.125	0	4
1753	1	0.1	0	4
1808	1	0.166666666666667	0	4
1810	1	0.166666666666667	0	4
1889	1	0.111111111111111	0	4
1955	1	0.142857142857143	0	4
2128	1	0.1	0	4
2189	1	0.1	0	4
2263	1	0.1	0	4
2425	1	0.1	0	4
2431	1	0.1	0	4
2443	1	0.1	0	4
2444	1	0.1	0	4
2445	1	0.1	0	4
2450	1	0.1	0	4
2455	1	0.1	0	4
2514	1	0.1	0	4
2686	1	0.5	0	4

category=2, cleavage_site=556
query=ptc-miR472b, target=Potri.019G002800.1,
score=4, range=544-565, strand=1

```
target  5' GGAAUGGGAGGAgUUGGUAAAA 3'
           :: ::::: :::::::: ::::
query   3' CCCUACCCACCUCAACCCUUUU 5'
```

>Potri.019G002800.1
#size=3247

127	1	0.1	0	4	
155	1	0.1	0	4	
175	1	0.125	0	4	
536	1	0.1	0	4	
547	1	0.1	0	4	
556	6	0.6	0	2	<<<
769	1	0.1	0	4	
770	1	0.1	0	4	
919	1	0.1	0	4	
1014	1	0.1	0	4	
1027	1	0.5	0	4	
1106	1	0.142857142857143	0	4	
1116	1	0.333333333333333	0	4	
1118	2	0.666666666666667	0	2	
1137	1	0.1	0	4	
1142	4	0.4	0	2	
1149	1	0.1	0	4	
1206	1	0.1	0	4	
1217	1	0.5	0	4	
1225	1	0.1	0	4	
1230	1	0.1	0	4	
1269	1	0.5	0	4	
1560	1	0.125	0	4	
1633	1	0.333333333333333	0	4	
1725	1	0.1	0	4	
1987	1	0.5	0	4	
2026	1	0.1	0	4	
2045	1	0.5	0	4	
2126	1	0.5	0	4	
2242	1	0.5	0	4	
2294	1	0.5	0	4	
2392	1	0.1	0	4	
2397	2	0.366666666666667	0	2	
2399	1	0.5	0	4	
2473	1	0.5	0	4	
2851	1	0.125	0	4	
2856	1	0.5	0	4	
2988	1	1	1	4	

category=4, cleavage_site=355
query=ptc-miR472b, target=Potri.T012900.1,
score=2, range=343-364, strand=1

```
target  5' GGGAUGGGAGGAgUUGGGAAGA 3'
           :::::::: :::::::::::.:
query   3' CCCUACCCACCUCAACCCUUUU 5'
```

>Potri.T012900.1
#size=3114

50	1	1	1	4	
355	1	0.5	0	4	<<<

634	1	0.1	0	4
740	1	0.1	0	4
743	1	0.1	0	4
745	2	0.25	0	2
747	1	0.1	0	4
792	1	0.1	0	4
793	1	0.1	0	4
959	1	1	1	4
1073	1	1	1	4
1621	1	0.1	0	4
1628	2	0.2	0	3
1804	1	0.1	0	4
1913	1	0.1	0	4
1988	1	0.5	0	4
2052	1	1	1	4
2092	1	1	1	4
2336	2	2	2	0
2477	1	1	1	4
2986	1	1	1	4

---

category=0, cleavage_site=271
query=ptc-miR472b, target=Potri.T013200.1,
score=4, range=259-280, strand=1

```
target  5' GGAAUGGGGGGAgUUGGUAAAA 3'
           :: ::::: :::::::: ::::
query   3' CCCUACCCACCUCAACCCUUUU 5'
```

>Potri.T013200.1
#size=1358

116	1	0.2	0	4	
138	1	0.5	0	4	
230	1	0.166666666666667	0	4	
270	1	0.333333333333333	0	4	
271	8	2.66666666666667	0	0	<<<
299	1	0.25	0	4	
442	1	1	1	4	
445	1	1	1	4	
487	1	0.1	0	4	
488	1	0.1	0	4	
491	1	0.111111111111111	0	4	
492	3	0.385714285714286	0	2	
527	1	0.166666666666667	0	4	
575	1	0.166666666666667	0	4	
605	1	0.25	0	4	
617	1	0.142857142857143	0	4	
618	1	0.142857142857143	0	4	
620	1	0.142857142857143	0	4	
626	1	0.142857142857143	0	4	
640	1	0.333333333333333	0	4	
642	1	0.333333333333333	0	4	
650	1	0.333333333333333	0	4	
680	2	0.666666666666667	0	2	
681	2	0.666666666666667	0	2	
682	2	0.666666666666667	0	2	
689	2	0.4	0	2	
691	2	0.666666666666667	0	2	
699	1	0.125	0	4	
711	1	0.1	0	4	
717	2	0.2	0	2	

725	1	0.1	0	4	
747	1	0.142857142857143	0	4	
749	1	0.142857142857143	0	4	
763	2	2	2	2	
790	1	0.1	0	4	
800	1	0.1	0	4	
817	1	0.2	0	4	
819	1	0.125	0	4	
820	1	0.125	0	4	
821	1	0.125	0	4	
823	1	0.125	0	4	
825	1	0.125	0	4	
826	1	0.125	0	4	
834	1	0.125	0	4	
866	1	0.125	0	4	
908	1	1	1	4	
974	1	1	1	4	
976	1	0.333333333333333	0	4	
1105	1	0.111111111111111	0	4	
1124	1	0.333333333333333	0	4	
1164	1	0.333333333333333	0	4	
1165	2	0.666666666666667	0	2	
1178	1	1	1	4	
1191	2	2	2	2	

---

category=0, cleavage_site=1110
query=ptc-miR472b, target=Potri.T024900.1,
score=3, range=1098-1119, strand=1

```
target  5' GGGAUGGGGGGAgUUGGUAAAA 3'
           :::::::: :::::::: ::::
query   3' CCCUACCCACCUCAACCCUUUU 5'
```

>Potri.T024900.1
#size=3263

178	1	0.333333333333333	0	4	
293	1	0.25	0	4	
323	1	0.2	0	4	
329	1	0.142857142857143	0	4	
438	1	0.125	0	4	
466	1	0.142857142857143	0	4	
475	2	0.222222222222222	0	2	
477	1	0.1	0	4	
498	1	0.111111111111111	0	4	
685	1	0.111111111111111	0	4	
703	2	0.25	0	2	
857	1	0.111111111111111	0	4	
891	1	0.125	0	4	
938	1	0.1	0	4	
978	1	0.1	0	4	
980	1	0.1	0	4	
991	1	0.1	0	4	
1101	1	0.1	0	4	
1108	1	0.166666666666667	0	4	
1110	20	2.8	0	0	<<<
1119	3	0.3	0	2	
1126	1	0.333333333333333	0	4	
1140	1	0.1	0	4	
1177	1	0.1	0	4	
1182	1	0.1	0	4	

1268	1	0.333333333333333	0	4
1318	1	0.1	0	4
1331	3	0.3	0	2
1355	1	0.5	0	4
1427	1	0.1	0	4
1490	2	0.2	0	2
1500	1	0.1	0	4
1560	1	0.1	0	4
1646	1	0.1	0	4
1649	1	0.1	0	4
1650	1	0.1	0	4
1652	1	0.1	0	4
1657	1	0.1	0	4
1673	1	0.1	0	4
1686	1	0.1	0	4
1687	1	0.1	0	4
1699	1	0.1	0	4
1700	1	0.1	0	4
1703	1	0.1	0	4
1704	1	0.1	0	4
1716	1	0.1	0	4
1728	1	0.111111111111111	0	4
1803	1	0.1	0	4
1804	1	0.111111111111111	0	4
1807	1	0.1	0	4
1844	1	0.1	0	4
1856	1	0.166666666666667	0	4
1862	1	0.111111111111111	0	4
1866	1	0.1	0	4
1869	1	0.1	0	4
1879	1	0.1	0	4
1880	1	0.1	0	4
1882	1	0.1	0	4
1885	1	0.1	0	4
1941	1	0.166666666666667	0	4
1942	1	0.166666666666667	0	4
1947	1	0.166666666666667	0	4
1961	1	0.1	0	4
1972	1	0.142857142857143	0	4
1990	1	0.142857142857143	0	4
1997	1	0.1	0	4
2008	2	0.333333333333333	0	2
2035	1	0.1	0	4
2323	1	0.166666666666667	0	4
2333	1	0.1	0	4
2463	1	0.1	0	4
2464	1	0.1	0	4
2466	1	0.1	0	4
2492	1	0.1	0	4
2616	2	0.2	0	2
2621	2	0.2	0	2
2822	2	0.2	0	2
2905	3	0.3	0	2
2906	1	0.1	0	4
2907	3	0.3	0	2
2908	1	0.1	0	4
2910	3	0.3	0	2
2911	2	0.2	0	2

2913	1	0.1	0	4
2916	2	0.2	0	2
2917	1	0.1	0	4
2918	1	0.1	0	4
2921	1	0.1	0	4
2927	1	0.1	0	4
2943	2	0.2	0	2
2944	1	0.1	0	4
2945	1	0.1	0	4
3033	1	0.1	0	4
3041	1	0.1	0	4
3081	1	0.1	0	4
3091	2	0.2	0	2
3094	1	0.1	0	4
3095	1	0.1	0	4
3097	2	0.2	0	2
3098	1	0.1	0	4
3099	1	0.1	0	4
3100	3	0.3	0	2
3101	1	0.1	0	4
3109	1	0.1	0	4
3110	1	0.1	0	4
3112	1	0.1	0	4
3196	1	0.1	0	4
3199	1	0.1	0	4
3200	1	0.1	0	4

---

category=4, cleavage_site=46
query=ptc-miR472b, target=Potri.T025300.1,
score=3, range=34-55, strand=1

```
target  5' GGGAUGGGAGGAgUUGGUAAAA 3'
           :::::::: :::::::: ::::
query   3' CCCUACCCACCUCAACCCUUUU 5'
```

>Potri.T025300.1
#size=2469

26	1	0.1	0	4	
37	1	0.1	0	4	
46	1	0.1	0	4	<<<
76	1	0.1	0	4	
118	2	0.2	0	2	
160	1	0.2	0	4	
218	1	0.125	0	4	
224	1	0.25	0	4	
232	1	0.1	0	4	
245	2	0.2	0	2	
246	1	0.1	0	4	
251	1	0.1	0	4	
252	1	0.1	0	4	
311	1	0.1	0	4	
326	1	0.2	0	4	
389	1	1	1	4	
412	1	0.1	0	4	
417	1	0.166666666666667	0	4	
426	1	0.1	0	4	
436	2	0.25	0	2	
437	1	0.142857142857143	0	4	
440	1	0.111111111111111	0	4	
455	1	0.25	0	4	

532	1	0.333333333333333	0	4
589	1	0.1	0	4
590	1	0.1	0	4
602	1	0.1	0	4
603	1	0.1	0	4
606	1	0.1	0	4
607	1	0.1	0	4
619	1	0.1	0	4
627	1	0.1	0	4
631	1	0.111111111111111	0	4
706	1	0.1	0	4
737	1	0.2	0	4
759	1	0.166666666666667	0	4
760	1	0.142857142857143	0	4
765	1	0.111111111111111	0	4
769	1	0.1	0	4
772	1	0.1	0	4
781	1	0.1	0	4
791	1	0.1	0	4
796	2	0.2	0	2
800	1	0.1	0	4
925	1	0.1	0	4
1161	1	0.1	0	4
1166	1	0.1	0	4
1170	1	0.1	0	4
1233	1	0.333333333333333	0	4
1282	1	0.1	0	4
1289	1	0.1	0	4
1343	1	0.1	0	4
1346	1	0.1	0	4
1354	1	0.1	0	4
1355	1	0.1	0	4
1357	1	0.1	0	4
1450	1	0.1	0	4
1451	1	0.1	0	4
1452	1	0.1	0	4
1453	1	0.1	0	4
1456	1	0.1	0	4
1637	1	0.2	0	4
1640	1	0.1	0	4
1779	1	0.1	0	4
1783	1	0.1	0	4
1825	2	0.2	0	2
1831	1	0.1	0	4
1869	1	0.1	0	4
1872	1	0.142857142857143	0	4
1882	1	0.333333333333333	0	4
1946	1	0.1	0	4
1957	1	0.1	0	4
1963	1	0.125	0	4
2002	1	0.125	0	4
2005	1	0.1	0	4
2015	2	0.2	0	2
2019	1	0.1	0	4
2021	2	0.2	0	2
2022	1	0.1	0	4
2023	1	0.1	0	4
2024	4	0.4	0	2

2025	1	0.1	0	4	
2033	1	0.1	0	4	
2034	1	0.1	0	4	
2036	1	0.1	0	4	
2197	1	0.111111111111111	0	4	
2209	1	0.1	0	4	
2309	1	0.1	0	4	

category=0, cleavage_site=106
query=ptc-miR472b, target=Potri.T025800.1,
score=4, range=94-115, strand=1

```
target  5' GGGAUGGGGGGAgUUGGUAAAU 3'
            :::::::: :::::::: :::
query   3' CCCUACCCACCUCAACCCUUUU 5'
```

>Potri.T025800.1
#size=3056

83	1	0.142857142857143	0	4	
104	1	0.333333333333333	0	4	
105	1	0.1	0	4	
106	4	2.2	2	0	<<<
136	1	0.1	0	4	
199	1	0.333333333333333	0	4	
274	2	2	2	2	
278	1	0.125	0	4	
292	1	0.1	0	4	
305	2	0.2	0	2	
309	1	0.1	0	4	
311	1	0.1	0	4	
312	1	0.1	0	4	
326	1	0.1	0	4	
327	3	0.3	0	2	
423	1	0.1	0	4	
472	1	0.1	0	4	
500	1	0.1	0	4	
567	1	0.1	0	4	
583	1	0.1	0	4	
646	1	0.1	0	4	
648	1	0.1	0	4	
652	1	0.1	0	4	
657	1	0.1	0	4	
661	1	0.166666666666667	0	4	
663	1	0.5	0	4	
664	1	0.2	0	4	
668	2	0.242857142857143	0	2	
669	1	0.1	0	4	
671	1	0.125	0	4	
680	1	0.125	0	4	
683	2	0.285714285714286	0	2	
706	1	0.1	0	4	
718	1	0.1	0	4	
726	1	0.1	0	4	
730	1	0.111111111111111	0	4	
759	1	0.1	0	4	
836	1	0.2	0	4	
875	2	0.2	0	2	
880	1	0.1	0	4	
881	1	0.1	0	4	
882	1	0.1	0	4	

884	2	0.433333333333333	0	2
887	1	0.1	0	4
890	1	0.1	0	4
895	2	0.2	0	2
899	1	0.1	0	4
915	2	0.5	0	2
963	1	0.1	0	4
964	1	0.1	0	4
965	1	0.1	0	4
1031	1	0.1	0	4
1037	1	0.1	0	4
1454	2	0.2	0	2
1470	1	0.1	0	4
1621	1	0.1	0	4
1622	1	0.1	0	4
1623	1	0.1	0	4
1624	1	0.1	0	4
1627	1	0.1	0	4
1659	1	0.111111111111111	0	4
1705	1	0.1	0	4
1708	1	0.1	0	4
1710	1	0.1	0	4
1787	1	0.2	0	4
1790	2	0.225	0	2
1866	1	0.1	0	4
1892	1	0.333333333333333	0	4
1896	2	0.2	0	2
1898	1	0.1	0	4
1909	1	0.1	0	4
1929	1	0.1	0	4
1931	1	0.5	0	4
1933	1	0.1	0	4
1936	1	0.142857142857143	0	4
1963	1	0.333333333333333	0	4
1975	3	0.3	0	2
1981	1	0.1	0	4
1983	1	0.1	0	4
1984	4	0.4	0	2
1987	1	0.1	0	4
1990	2	0.2	0	2
1991	1	0.1	0	4
1992	1	0.1	0	4
1995	1	0.1	0	4
2009	1	1	1	4
2018	1	0.25	0	4
2020	2	0.4	0	2
2091	1	0.1	0	4
2174	1	0.2	0	4
2177	2	0.225	0	2
2285	1	0.1	0	4
2301	1	0.1	0	4
2362	3	0.3	0	2
2368	1	0.1	0	4
2552	2	0.2	0	2
2560	2	0.2	0	2
2561	2	0.2	0	2
2570	1	0.1	0	4
2571	1	0.1	0	4

2573	1	0.1	0	4	
2581	2	0.25	0	2	
2593	1	0.1	0	4	
2604	1	0.111111111111111	0	4	
2609	1	0.125	0	4	
2612	1	0.166666666666667	0	4	
2651	1	0.1	0	4	
2763	1	0.1	0	4	
2767	1	0.1	0	4	
2779	1	0.142857142857143	0	4	
2791	1	1	1	4	
2846	1	0.1	0	4	
2852	2	0.2	0	2	
2854	1	0.1	0	4	
2861	1	0.2	0	4	
2891	1	0.5	0	4	

---

category=0, cleavage_site=1000
query=ptc-miR472b, target=Potri.T025900.1,
score=4, range=988-1009, strand=1

```
target  5' GGGACGGGGGGAgUUGGUAAAA 3'
           :::: ::: :::::::: ::::
query   3' CCCUACCCACCUCAACCCUUUU 5'
```

>Potri.T025900.1
#size=2025

39	1	0.111111111111111	0	4	
52	1	0.111111111111111	0	4	
54	1	0.1	0	4	
69	1	0.125	0	4	
97	1	0.142857142857143	0	4	
154	1	0.1	0	4	
173	1	0.1	0	4	
316	1	0.111111111111111	0	4	
488	1	0.111111111111111	0	4	
518	1	0.142857142857143	0	4	
522	1	0.125	0	4	
541	1	0.25	0	4	
569	1	0.1	0	4	
583	1	0.1	0	4	
611	1	0.1	0	4	
725	2	1	0	2	
831	1	0.111111111111111	0	4	
850	1	0.1	0	4	
999	2	0.433333333333333	0	2	
1000	12	3.36666666666667	0	0	<<<
1009	1	0.1	0	4	
1093	1	0.333333333333333	0	4	
1202	1	0.2	0	4	
1316	1	0.111111111111111	0	4	
1331	1	0.166666666666667	0	4	
1333	1	0.166666666666667	0	4	
1334	1	0.166666666666667	0	4	
1336	1	0.166666666666667	0	4	
1345	1	0.1	0	4	
1372	1	0.1	0	4	
1448	1	0.5	0	4	
1468	1	0.1	0	4	
1498	1	0.1	0	4	

1499	1	0.1	0	4	
1564	1	0.1	0	4	
1565	1	0.111111111111111	0	4	
1605	1	0.1	0	4	
1627	1	0.1	0	4	
1640	1	0.1	0	4	
1700	2	0.4	0	2	

category=0, cleavage_site=988
query=ptc-miR472b, target=Potri.T026200.1,
score=3, range=976-997, strand=1

```
target  5' GGGAUGGGAGGAgUUGGUAAAA 3'
           :::::::: :::::::: ::::
query   3' CCCUACCCACCUCAACCCUUUU 5'
```

>Potri.T026200.1
#size=3348

217	1	0.5	0	4	
221	1	0.5	0	4	
223	1	0.5	0	4	
235	1	0.142857142857143	0	4	
238	1	0.25	0	4	
247	2	0.311111111111111	0	2	
263	1	0.111111111111111	0	4	
275	1	0.5	0	4	
323	2	0.222222222222222	0	2	
327	1	0.111111111111111	0	4	
328	1	0.111111111111111	0	4	
330	1	0.111111111111111	0	4	
334	1	0.111111111111111	0	4	
345	1	0.142857142857143	0	4	
348	1	0.142857142857143	0	4	
351	1	0.333333333333333	0	4	
365	1	0.142857142857143	0	4	
396	2	0.222222222222222	0	2	
403	1	1	1	4	
416	1	0.125	0	4	
426	2	0.236111111111111	0	2	
439	3	0.420634920634921	0	2	
455	1	0.125	0	4	
516	1	0.166666666666667	0	4	
544	1	0.166666666666667	0	4	
549	1	0.125	0	4	
562	1	0.125	0	4	
673	1	0.142857142857143	0	4	
838	1	0.1	0	4	
968	1	0.1	0	4	
986	1	0.5	0	4	
988	4	2	0	0	<<<
1055	1	0.1	0	4	
1060	2	0.2	0	2	
1160	1	0.125	0	4	
1166	1	0.25	0	4	
1174	1	0.1	0	4	
1333	1	0.142857142857143	0	4	
1342	1	0.166666666666667	0	4	
1343	1	0.166666666666667	0	4	
1352	1	0.1	0	4	
1359	1	0.166666666666667	0	4	

1725	1	0.1	0	4
1747	1	0.1	0	4
1774	1	0.1	0	4
1836	1	0.1	0	4
1839	1	0.1	0	4
1907	1	0.1	0	4
1912	1	0.1	0	4
1913	1	0.1	0	4
1915	1	0.1	0	4
2276	1	0.1	0	4
2346	1	0.1	0	4
2370	1	0.1	0	4
2803	1	0.1	0	4
2806	1	0.1	0	4
2807	3	0.3	0	2
2808	1	0.1	0	4
2809	3	0.3	0	2
2810	1	0.1	0	4
2812	3	0.3	0	2
2813	1	0.1	0	4
2815	1	0.1	0	4
2818	2	0.2	0	2
2820	1	0.1	0	4
2823	1	0.1	0	4
2829	1	0.1	0	4
2837	1	0.142857142857143	0	4
2844	1	0.25	0	4
2845	2	0.2	0	2
2846	2	0.35	0	2
2847	1	0.1	0	4
2848	2	0.4	0	2
2850	1	0.142857142857143	0	4
2928	1	0.1	0	4
2943	1	0.1	0	4
2992	1	0.1	0	4
3001	2	0.2	0	2
3002	3	0.3	0	2
3003	1	0.1	0	4
3011	1	0.1	0	4
3012	1	0.1	0	4
3014	1	0.1	0	4
3199	1	0.1	0	4
3204	1	0.1	0	4

---

category=0, cleavage_site=490
query=ptc-miR472b, target=Potri.T026800.1,
score=3, range=478-499, strand=1

```
target  5' GGGAUGGGAGGAgUUGGUAAAA 3'
           :::::::: :::::::: ::::
query   3' CCCUACCCACCUCAACCCUUUU 5'
```

>Potri.T026800.1
#size=2859

8	1	0.142857142857143	0	4
59	1	0.1	0	4
73	1	0.1	0	4
101	1	0.1	0	4
121	2	0.225	0	2
129	1	0.25	0	4

133	1	0.2	0	4	
134	2	0.4	0	2	
136	2	0.666666666666667	0	2	
186	1	0.333333333333333	0	4	
215	2	1	0	2	
246	1	0.1	0	4	
268	1	0.25	0	4	
274	1	1	1	4	
315	1	0.111111111111111	0	4	
358	1	0.1	0	4	
360	1	0.1	0	4	
371	2	0.2	0	2	
470	1	0.1	0	4	
490	17	4.1	1	0	<<<
520	1	0.1	0	4	
557	1	0.1	0	4	
562	2	0.2	0	2	
662	1	0.125	0	4	
668	1	0.25	0	4	
676	1	0.1	0	4	
689	2	0.2	0	2	
690	1	0.1	0	4	
844	1	0.166666666666667	0	4	
845	1	0.166666666666667	0	4	
854	1	0.1	0	4	
884	1	0.1	0	4	
898	1	0.142857142857143	0	4	
913	1	0.1	0	4	
1077	1	0.25	0	4	
1133	1	0.166666666666667	0	4	
1143	1	0.1	0	4	
1217	1	0.1	0	4	
1230	1	0.1	0	4	
1252	1	0.1	0	4	
1255	1	0.1	0	4	
1264	1	0.1	0	4	
1274	1	0.1	0	4	
1279	2	0.2	0	2	
1283	1	0.1	0	4	
1347	2	0.2	0	2	
1415	1	0.1	0	4	
1420	1	0.1	0	4	
1421	1	0.1	0	4	
1423	1	0.1	0	4	
1435	1	0.111111111111111	0	4	
1444	1	0.1	0	4	
1525	1	0.142857142857143	0	4	
1585	1	0.1	0	4	
1609	1	0.1	0	4	
1612	2	0.2	0	2	
1681	1	0.2	0	4	
1709	1	0.166666666666667	0	4	
1777	1	0.1	0	4	
1838	1	0.1	0	4	
1849	1	0.1	0	4	
1852	1	0.1	0	4	
1854	1	0.1	0	4	
1878	1	0.1	0	4	

1890	1	1	1	4		
1945	1	0.1	0	4		
1946	1	0.1	0	4		
1947	1	0.1	0	4		
1948	1	0.1	0	4		
1951	1	0.1	0	4		
1990	1	0.111111111111111			0	4
1991	2	0.2	0	2		
1997	1	0.142857142857143			0	4
2011	2	0.211111111111111			0	2
2014	2	0.2	0	2		
2019	2	0.2	0	2		
2020	1	0.1	0	4		
2025	1	0.1	0	4		
2027	1	0.1	0	4		
2029	1	0.1	0	4		
2032	1	0.1	0	4		
2034	1	0.1	0	4		
2176	1	0.1	0	4		
2178	1	0.1	0	4		
2187	2	0.3	0	2		
2190	1	0.1	0	4		
2201	1	1	1	4		
2216	1	0.333333333333333			0	4
2220	2	0.2	0	2		
2222	1	0.1	0	4		
2233	1	0.1	0	4		
2253	1	0.1	0	4		
2260	1	0.142857142857143			0	4
2287	1	0.333333333333333			0	4
2299	1	0.1	0	4		
2319	1	0.1	0	4		
2325	1	0.1	0	4		
2340	1	0.25	0	4		
2344	1	0.5	0	4		
2402	1	0.333333333333333			0	4
2415	2	0.433333333333333			0	2
2416	1	0.166666666666667			0	4
2417	1	0.166666666666667			0	4
2419	1	0.333333333333333			0	4
2424	1	0.1	0	4		
2431	1	0.1	0	4		
2436	1	0.25	0	4		
2437	1	0.125	0	4		
2439	1	0.1	0	4		
2488	1	0.1	0	4		
2492	1	0.1	0	4		
2498	1	0.1	0	4		
2530	1	0.1	0	4		
2541	1	0.111111111111111			0	4
2546	1	0.125	0	4		
2549	1	0.166666666666667			0	4
2622	1	0.166666666666667			0	4
2675	1	0.333333333333333			0	4
2676	1	0.333333333333333			0	4
2679	1	0.333333333333333			0	4
2680	1	0.333333333333333			0	4
2709	1	0.1	0	4		

category=0, cleavage_site=1306
query=ptc-miR472b, target=Potri.T026900.1,
score=3, range=1294-1315, strand=1

```
target  5' GGGAUGGGAGGAgUUGGUAAAA 3'
            :::::::: :::::::: ::::
query   3' CCCUACCCACCUCAACCCUUUU 5'
```

>Potri.T026900.1
#size=3639

13	1	0.1	0	4
284	1	0.166666666666667	0	4
339	1	0.111111111111111	0	4
383	1	0.2	0	4
399	1	0.2	0	4
459	2	0.45	0	2
460	1	0.2	0	4
462	3	0.733333333333333	0	2
470	1	0.333333333333333	0	4
471	2	0.583333333333333	0	2
473	1	0.2	0	4
487	1	0.166666666666667	0	4
490	1	0.111111111111111	0	4
566	2	0.222222222222222	0	2
570	1	0.111111111111111	0	4
571	1	0.111111111111111	0	4
573	1	0.111111111111111	0	4
577	1	0.111111111111111	0	4
585	1	0.111111111111111	0	4
591	1	0.111111111111111	0	4
609	1	0.111111111111111	0	4
611	1	0.111111111111111	0	4
619	1	0.111111111111111	0	4
620	1	0.111111111111111	0	4
625	1	0.111111111111111	0	4
631	1	0.166666666666667	0	4
639	2	0.222222222222222	0	2
643	1	0.2	0	4
646	1	0.166666666666667	0	4
659	1	0.125	0	4
662	1	0.142857142857143	0	4
665	1	0.2	0	4
669	2	0.236111111111111	0	2
675	1	0.166666666666667	0	4
676	4	0.676190476190476	0	2
677	2	0.342857142857143	0	2
682	3	0.420634920634921	0	2
687	1	0.333333333333333	0	4
698	1	0.125	0	4
713	2	0.625	0	2
726	2	0.222222222222222	0	2
732	1	0.142857142857143	0	4
829	1	0.166666666666667	0	4
857	1	0.166666666666667	0	4
862	1	0.125	0	4
875	1	0.125	0	4
890	1	1	1	4
901	1	0.25	0	4
988	1	0.142857142857143	0	4

1137	1	0.111111111111111	0	4	
1156	1	0.1	0	4	
1174	1	0.1	0	4	
1176	1	0.1	0	4	
1187	2	0.2	0	2	
1286	1	0.1	0	4	
1297	1	0.1	0	4	
1306	14	2.8	0	0	<<<
1315	1	0.1	0	4	
1317	1	0.5	0	4	
1326	1	0.5	0	4	
1336	1	0.1	0	4	
1451	1	0.166666666666667	0	4	
1571	1	0.1	0	4	
1586	1	0.2	0	4	
1727	1	0.1	0	4	
1732	1	0.142857142857143	0	4	
1756	1	0.1	0	4	
1767	1	0.1	0	4	
1810	1	0.1	0	4	
1820	1	0.1	0	4	
1832	1	0.5	0	4	
1837	1	1	1	4	
1845	1	0.1	0	4	
1846	1	0.1	0	4	
1848	1	0.1	0	4	
1852	1	0.1	0	4	
1857	1	0.1	0	4	
1861	1	0.166666666666667	0	4	
1863	1	0.5	0	4	
1868	1	0.142857142857143	0	4	
1869	1	0.1	0	4	
1871	1	0.125	0	4	
1876	1	0.5	0	4	
1882	1	0.5	0	4	
1901	1	0.1	0	4	
1902	1	0.1	0	4	
1905	1	0.1	0	4	
1906	2	0.2	0	2	
1918	1	0.1	0	4	
1926	1	0.1	0	4	
1930	1	0.111111111111111	0	4	
1949	1	0.166666666666667	0	4	
2010	1	0.1	0	4	
2017	1	0.1	0	4	
2020	3	0.3	0	2	
2022	1	0.166666666666667	0	4	
2023	1	0.166666666666667	0	4	
2033	1	0.333333333333333	0	4	
2036	2	0.342857142857143	0	2	
2043	1	0.25	0	4	
2046	1	0.1	0	4	
2059	1	0.142857142857143	0	4	
2061	1	0.1	0	4	
2064	1	0.111111111111111	0	4	
2068	1	0.1	0	4	
2071	1	0.1	0	4	
2080	1	0.1	0	4	

2087	1	0.1	0	4
2088	1	0.333333333333333	0	4
2090	1	0.1	0	4
2095	2	0.2	0	2
2099	1	0.1	0	4
2109	1	0.5	0	4
2115	2	0.5	0	2
2122	1	1	1	4
2126	2	0.666666666666667	0	2
2135	1	0.25	0	4
2143	1	0.166666666666667	0	4
2144	1	0.166666666666667	0	4
2149	1	0.166666666666667	0	4
2163	1	0.1	0	4
2165	1	0.1	0	4
2231	1	0.1	0	4
2236	1	0.1	0	4
2237	1	0.1	0	4
2239	1	0.1	0	4
2299	1	0.2	0	4
2316	1	0.125	0	4
2328	2	0.2	0	2
2334	1	0.1	0	4
2341	1	0.142857142857143	0	4
2472	1	0.1	0	4
2477	1	0.1	0	4
2600	1	0.1	0	4
2642	1	0.1	0	4
2666	1	0.1	0	4
2754	1	0.1	0	4
2930	1	0.1	0	4
2992	1	0.1	0	4
3036	2	0.2	0	2
3038	1	0.1	0	4
3049	1	0.1	0	4
3069	1	0.1	0	4
3073	1	0.1	0	4
3076	1	0.142857142857143	0	4
3115	1	0.1	0	4
3119	2	0.2	0	2
3121	2	0.2	0	2
3124	3	0.3	0	2
3132	2	0.266666666666667	0	2
3216	1	0.1	0	4
3217	1	0.166666666666667	0	4
3218	1	0.166666666666667	0	4
3225	1	0.1	0	4
3277	1	0.125	0	4
3280	1	0.1	0	4
3290	2	0.2	0	2
3294	1	0.1	0	4
3296	1	0.1	0	4
3297	1	0.1	0	4
3298	1	0.1	0	4
3299	3	0.3	0	2
3300	1	0.1	0	4
3308	1	0.1	0	4
3309	1	0.1	0	4

3311 1 0.1 0 4
3319 2 0.25 0 2
3331 1 0.1 0 4
3342 1 0.111111111111111 0 4
3347 1 0.125 0 4
3350 1 0.166666666666667 0 4
3351 1 0.5 0 4
3484 2 0.2 0 2
3501 1 0.1 0 4
3513 1 0.2 0 4
3525 1 1 1 4
3528 1 1 1 4
3540 1 0.5 0 4
3587 1 0.125 0 4

---

category=0, cleavage_site=1051
query=ptc-miR472b, target=Potri.T027200.1,
score=3, range=1039-1060, strand=1

```
target  5' GGGAUGGGGGGAgUUGGUAAAA 3'
           :::::::: :::::::: ::::
query   3' CCCUACCCACCUCAACCCUUUU 5'
```

>Potri.T027200.1
#size=3393
11 1 0.2 0 4
17 1 0.142857142857143 0 4
109 1 0.111111111111111 0 4
126 1 0.125 0 4
154 1 0.142857142857143 0 4
211 1 0.1 0 4
536 1 0.111111111111111 0 4
659 1 0.1 0 4
679 2 0.225 0 2
687 1 0.25 0 4
691 1 0.2 0 4
692 2 0.4 0 2
694 2 0.666666666666667 0 2
847 1 0.125 0 4
953 1 0.1 0 4
955 1 0.1 0 4
1010 1 0.166666666666667 0 4
1027 1 1 1 4
1042 1 0.1 0 4
1051 32 23 22 0 <<<
1060 3 0.3 0 2
1118 1 0.1 0 4
1123 2 0.2 0 2
1187 1 1 1 4
1241 2 1 0 2
1331 1 0.2 0 4
1472 1 0.1 0 4
1474 1 0.1 0 4
1477 1 0.142857142857143 0 4
1501 1 0.1 0 4
1537 1 0.333333333333333 0 4
1552 1 0.333333333333333 0 4
1559 2 0.5 0 2
1577 3 0.3 0 2
1578 1 0.1 0 4

1580	1	0.1	0	4
1587	2	1.1	1	2
1596	1	1	1	4
1598	1	0.1	0	4
1628	1	0.1	0	4
1695	1	0.1	0	4
1797	1	0.166666666666667	0	4
1807	1	0.1	0	4
1829	1	0.1	0	4
1834	2	0.2	0	2
1838	1	0.1	0	4
1848	1	0.5	0	4
1854	2	0.5	0	2
1865	2	0.666666666666667	0	2
1874	1	0.25	0	4
1882	1	0.166666666666667	0	4
1883	1	0.166666666666667	0	4
1888	1	0.166666666666667	0	4
1902	1	0.1	0	4
1904	1	0.1	0	4
1913	1	0.142857142857143	0	4
1931	1	0.142857142857143	0	4
1938	1	0.1	0	4
1949	2	0.333333333333333	0	2
1990	1	0.111111111111111	0	4
2208	1	0.5	0	4
2339	1	0.1	0	4
2381	1	0.1	0	4
2393	1	0.1	0	4
2405	1	0.1	0	4
2433	1	0.1	0	4
2846	2	0.2	0	2
2847	1	0.1	0	4
2848	3	0.3	0	2
2849	1	0.1	0	4
2850	1	0.1	0	4
2851	3	0.3	0	2
2852	1	0.1	0	4
2854	1	0.1	0	4
2857	2	0.2	0	2
2858	1	0.1	0	4
2859	1	0.1	0	4
2862	1	0.1	0	4
2868	1	0.1	0	4
2884	2	0.2	0	2
2885	1	0.1	0	4
2886	1	0.1	0	4
2925	1	0.166666666666667	0	4
2936	1	0.333333333333333	0	4
2958	1	0.1	0	4
2959	1	0.166666666666667	0	4
2960	1	0.166666666666667	0	4
2967	1	0.1	0	4
3007	1	0.333333333333333	0	4
3008	1	0.333333333333333	0	4
3009	1	0.333333333333333	0	4
3031	1	0.1	0	4
3035	1	0.1	0	4

3036	1	0.1	0	4
3038	2	0.2	0	2
3039	1	0.1	0	4
3041	1	0.1	0	4
3135	1	0.142857142857143	0	4
3151	1	0.1	0	4
3155	1	0.1	0	4
3165	1	0.166666666666667	0	4
3226	2	0.2	0	2
3269	1	1	1	4
3298	1	0.125	0	4
3332	2	0.2	0	2
3334	1	0.1	0	4
3341	1	0.2	0	4

---

category=2, cleavage_site=589
query=ptc-miR472b, target=Potri.T027500.1,
score=3, range=577-598, strand=1

```
target  5' GGGAUGGGGGGAgUUGGUAAAA 3'
           :::::::: :::::::: ::::
query   3' CCCUACCCACCUCAACCCUUUU 5'
```

>Potri.T027500.1
#size=2996

6	1	0.125	0	4	
21	1	0.125	0	4	
32	1	0.166666666666667	0	4	
34	2	0.222222222222222	0	2	
72	1	0.1	0	4	
115	1	0.166666666666667	0	4	
143	1	0.166666666666667	0	4	
148	1	0.125	0	4	
161	1	0.125	0	4	
274	1	0.142857142857143	0	4	
345	1	0.1	0	4	
420	1	0.111111111111111	0	4	
439	1	0.1	0	4	
470	2	0.2	0	2	
580	1	0.1	0	4	
587	1	0.166666666666667	0	4	
588	1	0.1	0	4	
589	20	2.8	0	2	<<<
598	3	0.3	0	2	
661	1	0.1	0	4	
795	1	0.1	0	4	
841	1	0.25	0	4	
854	1	0.1	0	4	
879	1	0.5	0	4	
906	1	0.1	0	4	
955	1	0.1	0	4	
997	1	0.142857142857143	0	4	
1005	1	0.166666666666667	0	4	
1010	1	0.1	0	4	
1012	1	0.1	0	4	
1015	1	0.142857142857143	0	4	
1103	1	0.1	0	4	
1129	1	0.1	0	4	
1131	1	0.1	0	4	
1135	1	0.1	0	4	

1140	1	0.1	0	4
1152	1	0.1	0	4
1165	1	0.1	0	4
1166	1	0.1	0	4
1178	1	0.1	0	4
1179	1	0.1	0	4
1195	1	0.1	0	4
1203	1	0.1	0	4
1207	1	0.111111111111111	0	4
1226	1	0.166666666666667	0	4
1313	1	0.2	0	4
1338	1	0.1	0	4
1364	1	0.1	0	4
1440	1	0.1	0	4
1450	1	0.333333333333333	0	4
1508	1	0.1	0	4
1513	1	0.1	0	4
1514	1	0.1	0	4
1516	1	0.1	0	4
1537	1	0.1	0	4
1593	1	0.125	0	4
1595	1	0.1	0	4
1605	1	0.1	0	4
1611	1	0.1	0	4
1618	1	0.142857142857143	0	4
1678	1	0.1	0	4
1702	1	0.1	0	4
1705	2	0.2	0	2
1774	1	0.2	0	4
1812	1	0.1	0	4
1917	1	0.1	0	4
1947	1	0.1	0	4
2034	1	0.1	0	4
2037	1	0.111111111111111	0	4
2041	1	0.1	0	4
2042	1	0.1	0	4
2043	1	0.1	0	4
2044	1	0.1	0	4
2047	1	0.1	0	4
2121	1	0.1	0	4
2125	1	0.1	0	4
2130	1	0.1	0	4
2191	3	3	3	0
2201	1	0.2	0	4
2204	2	0.225	0	2
2323	1	0.1	0	4
2328	1	0.1	0	4
2332	1	0.1	0	4
2389	1	0.1	0	4
2392	1	0.1	0	4
2430	1	0.25	0	4
2431	2	0.2	0	2
2432	1	0.1	0	4
2433	1	0.1	0	4
2506	1	0.166666666666667	0	4
2507	2	1.16666666666667	1	2
2514	1	0.1	0	4
2578	1	0.1	0	4

2583	1	0.1	0	4
2701	1	0.1	0	4
2770	1	0.111111111111111	0	4

---

category=0, cleavage_site=46
query=ptc-miR472b, target=Potri.T028700.1,
score=3, range=34-55, strand=1

```
target  5' GGGAUGGGGGGAgUUGGUAAAA 3'
           :::::::: :::::::: ::::
query   3' CCCUACCCACCUCAACCCUUUU 5'
```

>Potri.T028700.1
#size=2492

37	1	0.1	0	4	
44	1	0.166666666666667	0	4	
45	1	0.1	0	4	
46	20	2.8	0	0	<<<
55	3	0.3	0	2	
76	1	0.1	0	4	
326	1	0.2	0	4	
391	1	0.142857142857143	0	4	
400	1	0.166666666666667	0	4	
401	1	0.166666666666667	0	4	
410	1	0.1	0	4	
426	1	0.1	0	4	
440	1	0.1	0	4	
563	3	0.3	0	2	
564	1	0.1	0	4	
573	1	0.1	0	4	
588	1	0.1	0	4	
613	1	0.1	0	4	
614	1	0.1	0	4	
698	1	0.1	0	4	
699	1	0.1	0	4	
742	1	0.1	0	4	
745	3	0.3	0	2	
758	1	0.1	0	4	
771	1	0.1	0	4	
786	1	0.1	0	4	
793	2	0.2	0	2	
797	1	0.1	0	4	
800	1	0.1	0	4	
883	1	0.333333333333333	0	4	
941	1	0.1	0	4	
947	1	0.1	0	4	
1038	2	0.2	0	2	
1044	1	0.1	0	4	
1051	1	0.142857142857143	0	4	
1099	1	0.333333333333333	0	4	
1135	1	0.1	0	4	
1138	1	0.1	0	4	
1182	1	0.1	0	4	
1187	1	0.1	0	4	
1191	1	0.1	0	4	
1364	2	0.2	0	2	
1367	1	0.1	0	4	
1375	1	0.1	0	4	
1378	1	0.1	0	4	
1516	1	0.111111111111111	0	4	

1558	1	0.1	0	4	
1560	1	0.1	0	4	
1702	1	0.1	0	4	
1704	1	0.1	0	4	
1713	2	0.3	0	2	
1716	1	0.1	0	4	
1884	1	0.1	0	4	
1894	1	0.111111111111111	0	4	
1991	1	0.5	0	4	
1994	1	0.166666666666667	0	4	
2002	1	0.125	0	4	
2005	1	0.1	0	4	
2019	1	0.1	0	4	
2021	2	0.2	0	2	
2022	1	0.1	0	4	
2024	2	0.2	0	2	
2120	1	0.1	0	4	
2134	2	0.2	0	2	
2138	1	0.1	0	4	
2148	1	0.166666666666667	0	4	
2235	1	0.1	0	4	
2309	1	0.1	0	4	
2312	1	0.125	0	4	

---

category=0, cleavage_site=1261
query=ptc-miR472b, target=Potri.T029000.1,
score=3, range=1249-1270, strand=1

```
target  5' GGGAUGGGGGGAgUUGGUAAAA 3'
            :::::::: :::::::: ::::
query   3' CCCUACCCACCUCAACCCUUUU 5'
```

>Potri.T029000.1
#size=4001

325	2	0.222222222222222	0	2	
327	1	0.1	0	4	
373	1	0.1	0	4	
577	1	0.111111111111111	0	4	
595	2	0.25	0	2	
749	1	0.111111111111111	0	4	
779	1	0.142857142857143	0	4	
783	1	0.125	0	4	
830	1	0.1	0	4	
844	1	0.1	0	4	
872	1	0.1	0	4	
892	2	0.225	0	2	
957	1	0.333333333333333	0	4	
1017	1	0.1	0	4	
1057	1	0.125	0	4	
1163	1	0.1	0	4	
1165	1	0.1	0	4	
1259	1	0.166666666666667	0	4	
1261	10	1	0	0	<<<
1270	3	0.3	0	2	
1328	1	0.1	0	4	
1333	1	0.1	0	4	
1433	1	0.125	0	4	
1447	1	0.1	0	4	
1460	2	0.2	0	2	
1461	1	0.1	0	4	

1466	1	0.1	0	4
1481	1	0.1	0	4
1482	4	0.4	0	2
1578	1	0.1	0	4
1606	1	0.142857142857143	0	4
1615	1	0.166666666666667	0	4
1616	1	0.166666666666667	0	4
1627	1	0.1	0	4
1651	2	0.25	0	2
1652	1	0.142857142857143	0	4
1655	1	0.111111111111111	0	4
1670	1	0.166666666666667	0	4
1672	1	0.166666666666667	0	4
1673	1	0.166666666666667	0	4
1675	1	0.166666666666667	0	4
1738	1	0.1	0	4
1773	1	0.125	0	4
1775	1	0.1	0	4
1777	2	0.222222222222222	0	2
1787	3	0.3	0	2
1788	1	0.1	0	4
1804	1	0.25	0	4
1808	1	0.1	0	4
1812	1	0.1	0	4
1814	1	0.142857142857143	0	4
1823	1	0.1	0	4
1848	1	0.25	0	4
1914	1	0.1	0	4
1928	2	0.35	0	2
1929	1	0.1	0	4
1933	1	0.1	0	4
1936	2	0.5	0	2
1938	1	0.1	0	4
1988	1	0.1	0	4
2021	1	0.1	0	4
2027	1	0.1	0	4
2030	2	0.2	0	2
2036	1	0.1	0	4
2037	1	0.1	0	4
2039	2	0.433333333333333	0	2
2042	1	0.1	0	4
2050	2	0.2	0	2
2096	2	0.4	0	2
2283	1	0.1	0	4
2289	1	0.1	0	4
2436	1	0.1	0	4
2499	1	0.333333333333333	0	4
2597	1	0.1	0	4
2599	1	0.1	0	4
2734	1	0.1	0	4
2735	1	0.1	0	4
2737	1	0.1	0	4
2749	1	0.1	0	4
2798	1	0.1	0	4
2946	1	0.333333333333333	0	4
3103	1	0.1	0	4
3134	1	0.2	0	4
3146	1	0.111111111111111	0	4

3147	1	0.1	0	4	
3149	1	0.1	0	4	
3278	1	0.1	0	4	
3287	2	0.2	0	2	
3288	1	0.1	0	4	
3364	1	0.333333333333333	0	4	
3387	1	0.1	0	4	
3391	1	0.1	0	4	
3522	1	0.142857142857143	0	4	
3540	1	0.333333333333333	0	4	

---

category=2, cleavage_site=763
query=ptc-miR472b, target=Potri.T037600.1,
score=3, range=751-772, strand=1

```
target  5' GGAAUUGGUGGAgUUGGGAAGA 3'
           :: :: ::::::::::::::.:
query   3' CCCUACCCACCUCAACCCUUUU 5'
```

>Potri.T037600.1
#size=2623

328	2	0.2	0	2	
475	1	0.142857142857143	0	4	
496	1	0.1	0	4	
498	1	0.1	0	4	
685	1	0.1	0	4	
761	2	0.2	0	2	
762	1	0.1	0	4	
763	5	0.5	0	2	<<<
873	2	0.222222222222222	0	2	
970	1	0.111111111111111	0	4	
1136	1	0.1	0	4	
1428	1	0.166666666666667	0	4	
1674	1	0.1	0	4	
1970	1	0.111111111111111	0	4	
1987	1	0.1	0	4	
2090	1	1	1	4	
2183	1	0.1	0	4	
2189	1	0.1	0	4	
2285	1	0.1	0	4	
2423	1	0.1	0	4	
2491	1	0.1	0	4	
2511	1	0.1	0	4	

---

category=2, cleavage_site=673
query=ptc-miR472b, target=Potri.T037900.1,
score=3, range=661-682, strand=1

```
target  5' GGAAUUGGUGGAgUUGGGAAGA 3'
           :: :: ::::::::::::::.:
query   3' CCCUACCCACCUCAACCCUUUU 5'
```

>Potri.T037900.1
#size=1686

238	2	0.2	0	2	
385	1	0.142857142857143	0	4	
406	1	0.1	0	4	
408	1	0.1	0	4	
595	1	0.1	0	4	
671	2	0.2	0	2	
672	6	0.6	0	2	
673	5	0.5	0	2	<<<

783	1	0.111111111111111	0	4
880	1	0.111111111111111	0	4
1046	1	0.1	0	4
1338	1	0.166666666666667	0	4
1454	1	0.111111111111111	0	4
1626	2	2	2	0
1634	1	1	1	4

category=0, cleavage_site=745
query=ptc-miR472b, target=Potri.T038300.1,
score=3, range=733-754, strand=1

```
target  5' GGAAUUGGUGGAgUUGGGAAGA 3'
             :: :: ::::::::::::::.:
query   3' CCCUACCCACCUCAACCCUUUU 5'
```

>Potri.T038300.1
#size=2502

7	2	0.25	0	2
8	1	0.1	0	4
16	2	0.2	0	2
84	1	0.1	0	4
85	1	0.1	0	4
88	1	0.1	0	4
104	1	0.1	0	4
107	1	0.1	0	4
111	1	0.1	0	4
112	1	0.1	0	4
116	1	0.1	0	4
117	1	0.1	0	4
143	1	0.1	0	4
156	1	0.1	0	4
163	1	0.1	0	4
211	1	0.1	0	4
218	1	0.1	0	4
221	1	0.1	0	4
223	1	0.1	0	4
224	1	0.1	0	4
228	1	0.1	0	4
315	1	0.1	0	4
322	1	0.1	0	4
324	2	0.2	0	2
325	1	0.1	0	4
328	1	0.1	0	4
374	1	0.1	0	4
380	1	0.1	0	4
518	1	0.1	0	4
522	1	0.1	0	4
523	1	0.1	0	4
530	1	0.1	0	4
533	1	0.1	0	4
537	1	0.1	0	4
545	1	0.1	0	4
548	1	0.1	0	4
563	1	0.1	0	4
565	1	0.1	0	4
569	1	0.1	0	4
587	1	0.142857142857143	0	4
667	1	0.1	0	4
717	1	0.111111111111111	0	4

742	1	0.125	0	4	
743	2	0.2	0	2	
744	7	0.7	0	2	
745	10	1.125	0	0	<<<
796	1	0.111111111111111	0	4	
840	2	0.222222222222222	0	2	
844	1	0.1	0	4	
852	1	0.125	0	4	
858	1	0.125	0	4	
860	1	0.125	0	4	
886	1	0.125	0	4	
892	1	0.1	0	4	
903	2	0.225	0	2	
933	4	0.444444444444444	0	2	
934	2	0.222222222222222	0	2	
940	1	0.111111111111111	0	4	
941	1	0.111111111111111	0	4	
953	1	0.125	0	4	
1166	1	0.1	0	4	
1205	2	0.2	0	2	
1215	1	0.1	0	4	
1496	1	0.1	0	4	
1497	1	0.1	0	4	
1530	1	0.1	0	4	
1538	1	0.1	0	4	
1549	1	0.1	0	4	
1563	1	0.1	0	4	
1586	1	0.1	0	4	
1613	1	0.1	0	4	
1630	1	0.1	0	4	
1658	1	0.125	0	4	

category=2, cleavage_site=499
query=ptc-miR472b, target=Potri.T039300.1,
score=3, range=487-508, strand=1

```
target  5' GGAAUUGGUGGAgUUGGGAAGA 3'
           :: :: ::::::::::::::.:
query   3' CCCUACCCACCUCAACCCUUUU 5'
```

>Potri.T039300.1
#size=2784

71	1	0.1	0	4
77	1	0.125	0	4
82	2	0.2	0	2
103	1	0.25	0	4
104	2	0.5	0	2
107	1	0.25	0	4
118	1	0.125	0	4
119	1	0.125	0	4
121	1	0.111111111111111	0	4
160	1	0.1	0	4
176	1	0.1	0	4
181	1	0.1	0	4
197	2	0.2	0	2
201	2	0.2	0	2
204	1	0.1	0	4
208	1	0.125	0	4
225	2	0.285714285714286	0	2
230	1	0.2	0	4

259	1	0.1	0	4		
272	1	0.1	0	4		
277	1	0.1	0	4		
302	1	0.1	0	4		
311	2	0.2	0	2		
318	1	0.1	0	4		
319	1	0.1	0	4		
320	1	0.1	0	4		
341	1	0.142857142857143			0	4
421	1	0.1	0	4		
471	1	0.111111111111111			0	4
496	1	0.125	0	4		
497	2	0.2	0	2		
498	7	0.7	0	2		
499	10	1.125	0	2	<<<	
594	2	0.222222222222222			0	2
598	1	0.1	0	4		
606	1	0.125	0	4		
612	1	0.125	0	4		
614	1	0.125	0	4		
640	1	0.125	0	4		
646	1	0.1	0	4		
657	2	0.225	0	2		
673	1	0.125	0	4		
686	5	0.544444444444444			0	2
687	2	0.222222222222222			0	2
688	4	0.444444444444444			0	2
689	87	9.66666666666665			0	0
690	4	0.444444444444444			0	2
697	1	0.1	0	4		
698	1	0.111111111111111			0	4
707	1	0.125	0	4		
720	1	0.1	0	4		
722	1	0.1	0	4		
726	1	0.111111111111111			0	4
813	1	0.1	0	4		
819	1	0.1	0	4		
821	1	0.1	0	4		
833	1	0.1	0	4		
884	1	0.1	0	4		
897	1	0.1	0	4		
898	1	0.1	0	4		
899	1	0.1	0	4		
927	1	0.1	0	4		
961	2	0.2	0	2		
1057	1	0.1	0	4		
1128	1	0.1	0	4		
1161	1	0.1	0	4		
1168	1	0.1	0	4		
1433	1	0.1	0	4		
1437	1	0.1	0	4		
1464	1	0.1	0	4		
1573	1	0.1	0	4		
1621	1	0.1	0	4		
1622	2	0.242857142857143			0	2
1795	1	0.1	0	4		
1835	1	0.1	0	4		
1930	1	0.1	0	4		

2018	1	0.1	0	4
2110	1	0.1	0	4
2111	1	0.1	0	4
2181	1	0.1	0	4
2214	1	1	1	4
2327	1	0.1	0	4
2378	1	0.125	0	4
2383	2	0.4	0	2
2526	1	0.1	0	4

category=0, cleavage_site=699
query=ptc-miR472b, target=Potri.T039900.1,
score=3, range=687-708, strand=1

```
target  5' GGAAUUGGUGGAgUUGGGAAGA 3'
           :: :: ::::::::::::::.:
query   3' CCCUACCCACCUCAACCCUUUU 5'
```

>Potri.T039900.1
#size=1700

61	1	0.1	0	4
65	1	0.1	0	4
69	1	0.1	0	4
79	1	0.1	0	4
97	1	0.1	0	4
122	1	0.2	0	4
123	1	0.2	0	4
126	1	0.2	0	4
165	1	0.1	0	4
172	1	0.1	0	4
175	1	0.1	0	4
177	1	0.1	0	4
178	1	0.1	0	4
179	1	0.333333333333333	0	4
181	2	0.333333333333333	0	2
182	1	0.1	0	4
189	1	0.1	0	4
190	1	0.1	0	4
194	1	0.1	0	4
195	1	0.1	0	4
203	1	0.1	0	4
226	1	0.1	0	4
227	1	0.1	0	4
232	1	0.1	0	4
233	1	0.1	0	4
237	1	0.1	0	4
248	1	0.1	0	4
264	1	0.1	0	4
269	1	0.1	0	4
276	1	0.1	0	4
278	2	0.2	0	2
279	1	0.1	0	4
296	1	0.1	0	4
301	1	0.1	0	4
310	1	0.1	0	4
315	1	0.1	0	4
320	1	0.1	0	4
326	1	0.1	0	4
328	1	0.1	0	4
334	1	0.1	0	4

431	1	0.1	0	4	
491	1	0.1	0	4	
499	1	0.1	0	4	
511	1	0.1	0	4	
517	1	0.1	0	4	
520	1	0.1	0	4	
531	1	0.1	0	4	
532	1	0.1	0	4	
541	1	0.142857142857143	0	4	
621	1	0.1	0	4	
671	1	0.111111111111111	0	4	
696	1	0.125	0	4	
697	2	0.2	0	2	
698	7	0.7	0	2	
699	10	1.125	0	0	<<<
750	1	0.111111111111111	0	4	
840	1	0.125	0	4	
846	1	0.1	0	4	
857	2	0.225	0	2	
894	1	0.111111111111111	0	4	
895	1	0.111111111111111	0	4	
907	1	0.125	0	4	
955	1	0.111111111111111	0	4	
1036	1	0.333333333333333	0	4	
1045	1	0.1	0	4	
1120	1	0.1	0	4	
1159	1	0.1	0	4	
1201	1	0.1	0	4	
1214	1	0.1	0	4	
1240	1	0.1	0	4	
1253	1	0.1	0	4	
1254	1	0.1	0	4	
1255	1	0.1	0	4	
1283	1	0.1	0	4	
1317	1	0.1	0	4	
1413	1	0.1	0	4	
1484	1	0.1	0	4	
1495	1	0.1	0	4	
1520	1	0.1	0	4	
1533	1	0.1	0	4	
1543	1	0.1	0	4	
1570	1	0.1	0	4	
1571	2	0.2	0	2	
1585	1	0.1	0	4	
1606	1	0.1	0	4	
1619	1	0.1	0	4	

---

category=0, cleavage_site=406
query=ptc-miR472b, target=Potri.T044500.1,
score=4, range=394-415, strand=1

```
target  5' GGAAUGGGGGGAgUUGGUAAAA 3'
             :: ::::: :::::::: ::::
query   3' CCCUACCCACCUCAACCCUUUU 5'
```

>Potri.T044500.1
#size=1267

38	1	0.25	0	4	
91	1	0.166666666666667	0	4	
99	1	0.1	0	4	

251	1	0.2	0	4	
273	1	0.5	0	4	
308	1	0.1	0	4	
310	1	0.1	0	4	
405	1	0.333333333333333	0	4	
406	8	2.66666666666667	0	0	<<<
434	1	0.25	0	4	
605	1	0.1	0	4	
647	1	0.142857142857143	0	4	
648	1	0.142857142857143	0	4	
650	1	0.142857142857143	0	4	
656	1	0.142857142857143	0	4	
729	1	0.125	0	4	
741	1	0.1	0	4	
747	1	0.1	0	4	
755	1	0.1	0	4	
777	1	0.142857142857143	0	4	
779	1	0.142857142857143	0	4	
830	1	0.1	0	4	
849	1	0.125	0	4	
850	1	0.125	0	4	
851	1	0.125	0	4	
853	1	0.125	0	4	
855	1	0.125	0	4	
856	1	0.125	0	4	
864	1	0.125	0	4	
896	1	0.125	0	4	
912	1	0.5	0	4	
914	1	0.5	0	4	
916	1	0.5	0	4	
917	1	0.5	0	4	
974	1	1	1	4	
1027	1	0.25	0	4	
1031	2	0.4	0	2	
1032	1	0.2	0	4	
1035	1	0.2	0	4	
1036	1	0.2	0	4	
1040	2	0.45	0	2	
1045	2	0.4	0	2	
1051	2	0.4	0	2	
1052	1	0.2	0	4	
1058	1	0.2	0	4	
1071	1	1	1	4	
1104	1	0.142857142857143	0	4	
1135	1	0.111111111111111	0	4	
1137	1	0.1	0	4	
1154	1	0.333333333333333	0	4	
1167	1	0.5	0	4	
1194	1	0.333333333333333	0	4	
1195	2	0.666666666666667	0	2	

---

category=0, cleavage_site=1261
query=ptc-miR472b, target=Potri.T052000.1,
score=3, range=1249-1270, strand=1

```
target  5' GGGAUGGGAGGAgUUGGUAAAA 3'
           :::::::: :::::::: ::::
query   3' CCCUACCCACCUCAACCCUUUU 5'
```

>Potri.T052000.1

#size=3480

325	1	0.111111111111111	0	4	
327	1	0.1	0	4	
373	1	0.1	0	4	
577	1	0.111111111111111	0	4	
595	2	0.25	0	2	
749	1	0.111111111111111	0	4	
779	1	0.142857142857143	0	4	
783	1	0.125	0	4	
830	1	0.1	0	4	
844	1	0.1	0	4	
872	1	0.1	0	4	
892	2	0.225	0	2	
957	1	0.333333333333333	0	4	
1017	1	0.1	0	4	
1057	1	0.125	0	4	
1163	1	0.1	0	4	
1165	1	0.1	0	4	
1241	1	0.1	0	4	
1252	1	0.1	0	4	
1259	1	0.166666666666667	0	4	
1261	9	0.9	0	0	<<<
1270	3	0.3	0	2	
1328	1	0.1	0	4	
1333	2	0.2	0	2	
1433	1	0.125	0	4	
1439	1	0.25	0	4	
1447	1	0.1	0	4	
1460	2	0.2	0	2	
1461	1	0.1	0	4	
1466	1	0.1	0	4	
1481	1	0.1	0	4	
1482	3	0.3	0	2	
1578	1	0.1	0	4	
1606	1	0.142857142857143	0	4	
1615	1	0.166666666666667	0	4	
1616	1	0.166666666666667	0	4	
1625	1	0.1	0	4	
1651	2	0.25	0	2	
1652	1	0.142857142857143	0	4	
1655	1	0.111111111111111	0	4	
1670	1	0.166666666666667	0	4	
1672	1	0.166666666666667	0	4	
1673	1	0.166666666666667	0	4	
1675	1	0.166666666666667	0	4	
1738	1	0.1	0	4	
1765	1	0.1	0	4	
1773	1	0.125	0	4	
1775	1	0.1	0	4	
1777	2	0.222222222222222	0	2	
1787	3	0.3	0	2	
1788	1	0.1	0	4	
1790	1	0.1	0	4	
1804	1	0.25	0	4	
1808	2	0.2	0	2	
1812	1	0.1	0	4	
1814	1	0.142857142857143	0	4	
1823	1	0.1	0	4	

1848	1	0.25	0	4
1914	1	0.1	0	4
1928	2	0.35	0	2
1929	1	0.1	0	4
1933	1	0.1	0	4
1936	2	0.5	0	2
1938	1	0.1	0	4
1988	1	0.1	0	4
2021	1	0.1	0	4
2027	1	0.1	0	4
2030	2	0.2	0	2
2035	1	0.1	0	4
2036	1	0.1	0	4
2037	1	0.1	0	4
2039	2	0.433333333333333	0	2
2042	1	0.1	0	4
2050	1	0.1	0	4
2096	2	0.4	0	2
2186	1	0.1	0	4
2192	1	0.1	0	4
2194	1	0.1	0	4
2283	2	0.2	0	2
2289	1	0.1	0	4
2432	1	0.1	0	4
2436	1	0.1	0	4
2499	1	0.333333333333333	0	4
2597	1	0.1	0	4
2599	1	0.1	0	4
2609	1	0.1	0	4
2734	1	0.1	0	4
2735	1	0.1	0	4
2737	1	0.1	0	4
2749	1	0.1	0	4
2798	1	0.1	0	4
2946	1	0.333333333333333	0	4
3167	3	0.3	0	2
3168	2	0.2	0	2
3244	1	0.333333333333333	0	4
3267	1	0.1	0	4
3402	1	0.142857142857143	0	4
3420	1	0.333333333333333	0	4

---

category=2, cleavage_site=711
query=ptc-miR472b, target=Potri.T112200.1,
score=3, range=699-720, strand=1

```
target  5' GGAAUUGGUGGAgUUGGGAAGA 3'
            :: :: ::::::::::::::.:
query   3' CCCUACCCACCUCAACCCUUUU 5'
```

>Potri.T112200.1
#size=2489

21	1	0.125	0	4
51	1	0.1	0	4
95	1	0.1	0	4
109	1	0.1	0	4
189	1	0.1	0	4
190	1	0.1	0	4
193	2	0.333333333333333	0	2
206	1	0.1	0	4

207	1	0.1	0	4		
215	1	0.1	0	4		
238	1	0.1	0	4		
239	1	0.1	0	4		
281	1	0.1	0	4		
288	1	0.1	0	4		
290	2	0.2	0	2		
291	1	0.1	0	4		
308	1	0.1	0	4		
313	1	0.1	0	4		
322	1	0.1	0	4		
327	1	0.1	0	4		
332	1	0.1	0	4		
338	1	0.1	0	4		
340	1	0.1	0	4		
346	1	0.1	0	4		
413	1	0.1	0	4		
414	1	0.1	0	4		
416	1	0.1	0	4		
422	1	0.1	0	4		
443	2	0.2	0	2		
496	1	0.1	0	4		
499	1	0.1	0	4		
503	1	0.1	0	4		
511	1	0.1	0	4		
514	1	0.1	0	4		
531	1	0.1	0	4		
532	1	0.1	0	4		
553	1	0.142857142857143			0	4
633	1	0.1	0	4		
683	1	0.111111111111111			0	4
708	1	0.125	0	4		
709	2	0.2	0	2		
710	7	0.7	0	2		
711	10	1.125	0	2	<<<	
762	1	0.111111111111111			0	4
806	2	0.222222222222222			0	2
810	1	0.1	0	4		
818	1	0.125	0	4		
858	1	0.1	0	4		
869	2	0.225	0	2		
885	1	0.125	0	4		
898	4	0.444444444444444			0	2
899	1	0.111111111111111			0	4
900	4	0.444444444444444			0	2
901	90	9.99999999999998			0	0
902	3	0.333333333333333			0	2
907	1	0.111111111111111			0	4
910	1	0.111111111111111			0	4
918	1	0.1	0	4		
934	1	0.1	0	4		
938	1	0.111111111111111			0	4
1051	1	0.333333333333333			0	4
1060	1	0.1	0	4		
1255	1	0.1	0	4		
1268	1	0.1	0	4		
1269	1	0.1	0	4		
1270	1	0.1	0	4		

1475	1	0.1	0	4
1493	1	0.1	0	4
1496	1	0.1	0	4
1504	1	0.1	0	4
1515	1	0.1	0	4
1529	1	0.1	0	4
1536	1	0.1	0	4
1542	1	0.1	0	4
1552	1	0.1	0	4
1596	1	0.1	0	4
1746	1	0.1	0	4
1842	1	0.1	0	4
1882	1	0.1	0	4
1903	1	0.1	0	4
1918	1	0.1	0	4
1977	1	0.1	0	4
2045	1	0.1	0	4
2065	1	0.1	0	4
2157	1	0.1	0	4
2158	1	0.1	0	4
2397	1	0.5	0	4
2429	2	0.4	0	2

ptc-miR1447

category=0, cleavage_site=906
query=ptc-miR1447, target=Potri.019G105700.1,
score=4, range=895-915, strand=1

```
target  5' GACCGAGGCACuGCGAUUCUA 3'
           .: :.:::::::::.:::::
query   3' UUAGUUCCGUGACGUUAAGAC 5'
```

>Potri.019G105700.1
#size=1305

66	1	0.1	0	4
70	2	0.2	0	2
73	1	0.1	0	4
74	1	0.166666666666667	0	4
82	1	0.142857142857143	0	4
83	2	0.285714285714286	0	2
85	1	0.111111111111111	0	4
125	1	0.142857142857143	0	4
126	1	0.142857142857143	0	4
129	1	0.125	0	4
132	2	0.266666666666667	0	2
136	1	0.333333333333333	0	4
138	1	0.25	0	4
144	1	0.25	0	4
148	1	0.142857142857143	0	4
158	1	0.125	0	4
254	1	0.125	0	4
264	1	0.125	0	4
299	1	0.1	0	4
303	1	0.111111111111111	0	4
344	1	0.2	0	4
351	1	0.1	0	4
377	1	0.1	0	4
378	1	0.1	0	4
379	1	0.1	0	4

398	1	0.1	0	4	
400	1	0.1	0	4	
401	1	0.1	0	4	
450	1	0.333333333333333	0	4	
485	1	0.2	0	4	
494	1	0.166666666666667	0	4	
495	1	0.166666666666667	0	4	
500	4	0.4	0	2	
501	1	0.1	0	4	
502	1	0.1	0	4	
506	1	0.1	0	4	
508	1	0.1	0	4	
510	1	0.1	0	4	
511	1	0.1	0	4	
513	1	0.1	0	4	
514	1	0.1	0	4	
515	1	0.1	0	4	
518	1	0.1	0	4	
699	2	0.285714285714286	0	2	
702	1	0.142857142857143	0	4	
705	1	0.2	0	4	
712	1	0.142857142857143	0	4	
810	1	0.1	0	4	
840	1	0.1	0	4	
845	1	0.1	0	4	
846	1	0.1	0	4	
872	1	0.125	0	4	
894	1	0.142857142857143	0	4	
896	2	0.25	0	2	
899	1	0.1	0	4	
906	7	0.7	0	0	<<<
932	2	0.4	0	2	
933	1	0.1	0	4	
937	1	0.333333333333333	0	4	
940	1	0.25	0	4	
968	1	0.2	0	4	
1122	1	0.25	0	4	
1130	1	0.5	0	4	

---

category=2, cleavage_site=1482
query=ptc-miR1447, target=Potri.019G105900.1,
score=4, range=1471-1491, strand=1

```
target  5' GACCGAGGCACuGCGAUUCUA 3'
             .: :.:::::::::.:::::
query   3' UUAGUUCCGUGACGUUAAGAC 5'
```

>Potri.019G105900.1
#size=1833

75	1	0.333333333333333	0	4
247	2	0.30952380952381	0	2
274	1	0.125	0	4
276	1	0.1	0	4
281	2	0.222222222222222	0	2
318	1	0.142857142857143	0	4
366	1	0.166666666666667	0	4
414	1	0.142857142857143	0	4
419	1	0.142857142857143	0	4
428	1	0.1	0	4
438	1	0.1	0	4

439	2	0.2	0	2
443	1	0.1	0	4
444	1	0.1	0	4
445	1	0.1	0	4
454	1	0.1	0	4
457	1	0.1	0	4
459	1	0.1	0	4
460	2	0.2	0	2
462	1	0.1	0	4
463	2	0.2	0	2
464	1	0.1	0	4
465	1	0.1	0	4
489	1	0.142857142857143	0	4
513	1	0.1	0	4
526	1	0.1	0	4
540	1	0.1	0	4
548	1	0.1	0	4
557	1	0.1	0	4
560	1	0.1	0	4
652	2	0.2	0	2
653	1	0.1	0	4
656	1	0.1	0	4
657	1	0.1	0	4
675	1	0.125	0	4
723	1	0.1	0	4
739	1	0.142857142857143	0	4
749	1	0.125	0	4
760	2	0.285714285714286	0	2
761	1	0.142857142857143	0	4
768	1	0.166666666666667	0	4
837	1	0.333333333333333	0	4
848	1	0.125	0	4
858	1	0.125	0	4
893	1	0.1	0	4
897	1	0.111111111111111	0	4
903	1	0.166666666666667	0	4
905	1	0.25	0	4
938	1	0.2	0	4
943	1	0.111111111111111	0	4
945	1	0.1	0	4
949	1	0.1	0	4
950	1	0.1	0	4
951	1	0.1	0	4
952	2	0.2	0	2
968	1	0.111111111111111	0	4
973	1	0.1	0	4
974	1	0.1	0	4
980	1	0.1	0	4
987	1	0.1	0	4
992	1	0.1	0	4
994	1	0.1	0	4
995	1	0.1	0	4
999	1	0.1	0	4
1004	1	0.142857142857143	0	4
1044	1	0.333333333333333	0	4
1085	1	0.2	0	4
1094	3	0.3	0	2
1095	1	0.1	0	4

1096	1	0.1	0	4	
1100	1	0.1	0	4	
1102	1	0.1	0	4	
1104	1	0.1	0	4	
1105	1	0.1	0	4	
1107	1	0.1	0	4	
1108	1	0.1	0	4	
1109	1	0.1	0	4	
1112	1	0.1	0	4	
1115	2	0.333333333333333	0	2	
1214	1	0.166666666666667	0	4	
1216	1	0.166666666666667	0	4	
1362	1	0.125	0	4	
1386	1	0.1	0	4	
1416	1	0.1	0	4	
1421	1	0.1	0	4	
1422	1	0.1	0	4	
1425	1	0.1	0	4	
1428	1	0.1	0	4	
1441	1	0.125	0	4	
1448	1	0.125	0	4	
1456	1	0.142857142857143	0	4	
1468	1	0.166666666666667	0	4	
1470	1	0.142857142857143	0	4	
1472	2	0.25	0	2	
1475	1	0.1	0	4	
1482	7	0.7	0	2	<<<
1509	1	0.1	0	4	
1549	1	0.2	0	4	
1649	2	1	0	0	
1652	1	0.142857142857143	0	4	
1656	1	0.125	0	4	
1665	2	0.25	0	2	
1703	1	0.111111111111111	0	4	
1723	1	0.333333333333333	0	4	

---

category=2, cleavage_site=1437
query=ptc-miR1447, target=Potri.019G106000.1,
score=2, range=1426-1446, strand=1

```
target  5' AAUGAAGGCACuGCAAUUCUU 3'
            ::: :::::::::::::::
query   3' UUAGUUCCGUGACGUUAAGAC 5'
```

>Potri.019G106000.1
#size=1851

29	1	0.166666666666667	0	4
57	1	0.25	0	4
99	1	0.111111111111111	0	4
104	1	0.1	0	4
109	1	0.1	0	4
129	2	0.211111111111111	0	2
137	1	0.125	0	4
140	1	0.142857142857143	0	4
142	1	0.142857142857143	0	4
152	1	0.142857142857143	0	4
153	2	0.285714285714286	0	2
158	2	0.285714285714286	0	2
161	1	0.166666666666667	0	4
170	1	0.142857142857143	0	4

173	1	0.142857142857143	0	4
177	1	0.142857142857143	0	4
184	3	0.428571428571429	0	2
188	1	0.125	0	4
189	1	0.2	0	4
192	1	0.2	0	4
198	1	0.125	0	4
199	1	0.125	0	4
201	1	0.1	0	4
205	1	0.1	0	4
206	2	0.222222222222222	0	2
210	2	0.25	0	2
216	1	0.25	0	4
245	1	0.125	0	4
322	1	0.1	0	4
324	1	0.1	0	4
327	1	0.1	0	4
328	1	0.111111111111111	0	4
329	2	0.211111111111111	0	2
332	1	0.1	0	4
333	1	0.111111111111111	0	4
336	6	0.6	0	2
337	1	0.1	0	4
338	1	0.1	0	4
339	1	0.1	0	4
340	1	0.1	0	4
343	1	0.1	0	4
344	1	0.125	0	4
345	1	0.142857142857143	0	4
350	1	0.125	0	4
351	3	0.3	0	2
352	1	0.111111111111111	0	4
353	2	0.2	0	2
355	1	0.125	0	4
356	2	0.3	0	2
357	1	0.111111111111111	0	4
359	1	0.1	0	4
361	1	0.1	0	4
362	1	0.1	0	4
363	1	0.1	0	4
364	1	0.1	0	4
368	1	0.1	0	4
369	1	0.1	0	4
370	1	0.1	0	4
376	1	0.1	0	4
379	1	0.1	0	4
382	1	0.1	0	4
384	1	0.1	0	4
385	2	0.2	0	2
387	1	0.1	0	4
388	1	0.1	0	4
389	1	0.1	0	4
465	1	0.1	0	4
470	1	0.1	0	4
471	1	0.1	0	4
472	1	0.1	0	4
473	1	0.1	0	4
474	1	0.1	0	4

476	1	0.1	0	4
482	2	0.2	0	2
485	1	0.1	0	4
575	1	0.1	0	4
577	1	0.1	0	4
579	1	0.1	0	4
583	2	0.2	0	2
586	1	0.1	0	4
598	1	0.111111111111111	0	4
638	1	0.142857142857143	0	4
639	1	0.142857142857143	0	4
642	1	0.125	0	4
645	2	0.266666666666667	0	2
646	2	0.45	0	2
676	1	0.333333333333333	0	4
857	1	0.2	0	4
862	1	0.111111111111111	0	4
864	1	0.1	0	4
868	1	0.1	0	4
869	1	0.1	0	4
870	1	0.1	0	4
871	2	0.2	0	2
875	1	0.2	0	4
877	1	0.142857142857143	0	4
887	1	0.111111111111111	0	4
890	1	0.1	0	4
891	1	0.1	0	4
892	1	0.1	0	4
893	1	0.1	0	4
899	1	0.1	0	4
906	1	0.1	0	4
911	1	0.1	0	4
913	1	0.1	0	4
914	1	0.1	0	4
918	1	0.1	0	4
923	1	0.142857142857143	0	4
971	1	0.333333333333333	0	4
976	1	0.333333333333333	0	4
978	1	0.333333333333333	0	4
979	1	0.333333333333333	0	4
980	2	0.666666666666667	0	2
984	1	0.333333333333333	0	4
987	1	0.333333333333333	0	4
988	1	0.333333333333333	0	4
989	2	0.666666666666667	0	2
990	1	0.333333333333333	0	4
992	1	0.333333333333333	0	4
995	2	0.666666666666667	0	2
998	2	0.533333333333333	0	2
1000	3	1	0	1
1002	1	0.333333333333333	0	4
1005	1	0.333333333333333	0	4
1007	1	0.166666666666667	0	4
1008	1	0.166666666666667	0	4
1034	2	0.333333333333333	0	2
1042	1	0.25	0	4
1043	1	0.25	0	4
1047	1	0.25	0	4

1136	1	0.166666666666667	0	4	
1138	1	0.166666666666667	0	4	
1211	1	1	1	4	
1221	1	0.5	0	4	
1305	1	0.125	0	4	
1313	1	0.5	0	4	
1326	1	0.5	0	4	
1329	1	0.1	0	4	
1359	1	0.1	0	4	
1437	6	0.6	0	2	<<<
1630	1	0.142857142857143	0	4	

---

category=2, cleavage_site=1507
query=ptc-miR1447, target=Potri.019G107700.1,
score=3, range=1496-1516, strand=1

```
target  5' GACCGAGGCACuGCAAUUCUA 3'
            .: :.:::::::::::::::
query   3' UUAGUUCCGUGACGUUAAGAC 5'
```

>Potri.019G107700.1
#size=1901

144	1	0.166666666666667	0	4
172	1	0.25	0	4
214	1	0.111111111111111	0	4
219	1	0.1	0	4
224	1	0.1	0	4
244	2	0.211111111111111	0	2
252	1	0.125	0	4
255	1	0.142857142857143	0	4
257	1	0.142857142857143	0	4
267	1	0.142857142857143	0	4
268	2	0.285714285714286	0	2
273	2	0.285714285714286	0	2
276	1	0.166666666666667	0	4
285	1	0.142857142857143	0	4
288	1	0.142857142857143	0	4
292	1	0.142857142857143	0	4
299	3	0.428571428571429	0	2
303	1	0.125	0	4
304	1	0.2	0	4
307	1	0.2	0	4
313	1	0.125	0	4
314	1	0.125	0	4
316	1	0.1	0	4
320	1	0.1	0	4
321	2	0.222222222222222	0	2
325	2	0.25	0	2
346	1	0.25	0	4
358	1	0.142857142857143	0	4
406	1	0.166666666666667	0	4
430	1	0.25	0	4
454	1	0.142857142857143	0	4
459	1	0.142857142857143	0	4
479	2	0.2	0	2
491	1	0.1	0	4
505	1	0.1	0	4
553	1	0.1	0	4
578	1	0.1	0	4
580	1	0.1	0	4

588	1	0.1	0	4
591	1	0.1	0	4
657	1	0.111111111111111	0	4
661	1	0.125	0	4
680	1	0.1	0	4
681	1	0.1	0	4
683	1	0.1	0	4
684	1	0.1	0	4
689	2	0.2	0	2
692	1	0.1	0	4
693	1	0.166666666666667	0	4
736	1	1	1	4
744	1	0.142857142857143	0	4
745	1	0.142857142857143	0	4
748	1	0.125	0	4
751	2	0.266666666666667	0	2
752	2	0.45	0	2
755	1	0.333333333333333	0	4
757	1	0.25	0	4
763	1	0.25	0	4
767	1	0.142857142857143	0	4
777	1	0.125	0	4
788	2	0.285714285714286	0	2
789	1	0.142857142857143	0	4
796	1	0.166666666666667	0	4
873	1	0.125	0	4
877	1	0.25	0	4
883	1	0.125	0	4
918	1	0.1	0	4
928	1	0.25	0	4
968	1	0.111111111111111	0	4
970	1	0.1	0	4
974	1	0.1	0	4
975	1	0.1	0	4
976	1	0.1	0	4
977	2	0.2	0	2
981	1	0.2	0	4
983	1	0.142857142857143	0	4
993	1	0.111111111111111	0	4
996	1	0.1	0	4
997	1	0.1	0	4
998	1	0.1	0	4
999	1	0.1	0	4
1005	1	0.1	0	4
1012	1	0.1	0	4
1017	1	0.1	0	4
1019	1	0.1	0	4
1020	1	0.1	0	4
1024	1	0.1	0	4
1029	1	0.142857142857143	0	4
1110	1	0.2	0	4
1119	4	0.4	0	2
1120	1	0.1	0	4
1121	1	0.1	0	4
1125	1	0.1	0	4
1127	1	0.1	0	4
1129	1	0.1	0	4
1130	1	0.1	0	4

1132	1	0.1	0	4	
1133	1	0.1	0	4	
1134	1	0.1	0	4	
1137	1	0.1	0	4	
1230	1	0.166666666666667	0	4	
1232	1	0.166666666666667	0	4	
1285	2	0.285714285714286	0	2	
1288	1	0.142857142857143	0	4	
1291	1	0.2	0	4	
1293	1	0.2	0	4	
1295	1	0.333333333333333	0	4	
1296	1	0.142857142857143	0	4	
1297	1	0.142857142857143	0	4	
1298	1	0.142857142857143	0	4	
1304	1	0.166666666666667	0	4	
1341	1	0.5	0	4	
1387	1	0.125	0	4	
1411	1	0.1	0	4	
1441	1	0.1	0	4	
1446	1	0.1	0	4	
1447	1	0.1	0	4	
1450	1	0.1	0	4	
1453	1	0.1	0	4	
1466	1	0.125	0	4	
1473	1	0.125	0	4	
1481	1	0.142857142857143	0	4	
1488	1	0.333333333333333	0	4	
1493	1	0.166666666666667	0	4	
1495	1	0.142857142857143	0	4	
1497	2	0.25	0	2	
1500	1	0.1	0	4	
1507	7	0.7	0	2	<<<
1533	2	0.4	0	2	
1534	1	0.1	0	4	
1538	1	0.333333333333333	0	4	
1541	1	0.25	0	4	
1574	1	0.2	0	4	
1677	1	0.142857142857143	0	4	
1678	1	0.166666666666667	0	4	
1681	1	0.125	0	4	
1690	2	0.25	0	2	
1694	1	0.166666666666667	0	4	
1700	1	0.142857142857143	0	4	
1728	1	0.111111111111111	0	4	

---

category=2, cleavage_site=1374
query=ptc-miR1447, target=Potri.019G107800.1,
score=4, range=1363-1383, strand=1

```
target  5' GACCGAGGCACuGCGAUUCUA 3'
           .: :.:::::::::.:::::
query   3' UUAGUUCCGUGACGUUAAGAC 5'
```

>Potri.019G107800.1
#size=1725

139	2	0.30952380952381	0	2
166	1	0.125	0	4
168	1	0.1	0	4
173	2	0.222222222222222	0	2
210	1	0.142857142857143	0	4

258	1	0.166666666666667	0	4
306	1	0.142857142857143	0	4
311	1	0.142857142857143	0	4
320	1	0.1	0	4
323	1	0.1	0	4
330	1	0.1	0	4
331	2	0.2	0	2
335	1	0.1	0	4
336	1	0.1	0	4
337	1	0.1	0	4
343	1	0.1	0	4
346	1	0.1	0	4
349	1	0.1	0	4
351	1	0.1	0	4
352	1	0.1	0	4
354	1	0.1	0	4
355	2	0.2	0	2
356	1	0.1	0	4
357	1	0.1	0	4
381	1	0.142857142857143	0	4
405	1	0.1	0	4
418	1	0.1	0	4
432	1	0.1	0	4
440	1	0.1	0	4
449	1	0.1	0	4
452	1	0.1	0	4
544	2	0.2	0	2
545	1	0.1	0	4
548	1	0.1	0	4
549	1	0.1	0	4
567	1	0.125	0	4
615	1	0.1	0	4
631	1	0.142857142857143	0	4
641	1	0.125	0	4
652	2	0.285714285714286	0	2
653	1	0.142857142857143	0	4
660	1	0.166666666666667	0	4
729	1	0.333333333333333	0	4
740	1	0.125	0	4
750	1	0.125	0	4
785	1	0.1	0	4
789	1	0.111111111111111	0	4
795	1	0.166666666666667	0	4
797	1	0.25	0	4
830	1	0.2	0	4
835	1	0.111111111111111	0	4
837	1	0.1	0	4
841	1	0.1	0	4
842	1	0.1	0	4
843	1	0.1	0	4
844	2	0.2	0	2
860	1	0.111111111111111	0	4
864	1	0.1	0	4
866	1	0.1	0	4
872	1	0.1	0	4
879	1	0.1	0	4
884	1	0.1	0	4
886	1	0.1	0	4

887	1	0.1	0	4	
891	1	0.1	0	4	
896	1	0.142857142857143	0	4	
936	1	0.333333333333333	0	4	
977	1	0.2	0	4	
986	4	0.4	0	2	
987	1	0.1	0	4	
988	1	0.1	0	4	
992	1	0.1	0	4	
994	1	0.1	0	4	
996	1	0.1	0	4	
997	1	0.1	0	4	
999	1	0.1	0	4	
1000	1	0.1	0	4	
1001	1	0.1	0	4	
1004	1	0.1	0	4	
1007	2	0.333333333333333	0	2	
1106	1	0.166666666666667	0	4	
1108	1	0.166666666666667	0	4	
1254	1	0.125	0	4	
1278	1	0.1	0	4	
1308	1	0.1	0	4	
1313	1	0.1	0	4	
1314	1	0.1	0	4	
1317	1	0.1	0	4	
1320	1	0.1	0	4	
1333	1	0.125	0	4	
1340	1	0.125	0	4	
1348	1	0.142857142857143	0	4	
1360	1	0.166666666666667	0	4	
1362	1	0.142857142857143	0	4	
1364	2	0.25	0	2	
1367	1	0.1	0	4	
1374	7	0.7	0	2	<<<
1401	1	0.1	0	4	
1441	1	0.2	0	4	
1541	2	1	0	0	
1544	1	0.142857142857143	0	4	
1548	1	0.125	0	4	
1557	2	0.25	0	2	
1595	1	0.111111111111111	0	4	
1615	1	0.333333333333333	0	4	

category=4, cleavage_site=1557
query=ptc-miR1447, target=Potri.019G108000.1,
score=3, range=1546-1566, strand=1

```
target  5' AAUGAAGGCACuGCAAUUUUA 3'
            ::: ::::::::::::::.:
query   3' UUAGUUCCGUGACGUUAAGAC 5'
```

>Potri.019G108000.1
#size=1908

131	1	0.5	0	4
177	1	0.5	0	4
178	1	0.5	0	4
242	1	0.111111111111111	0	4
247	1	0.1	0	4
252	1	0.1	0	4
341	1	0.125	0	4

342	1	0.125	0	4		
344	1	0.1	0	4		
349	2	0.222222222222222			0	2
353	2	0.25	0	2		
386	1	0.142857142857143			0	4
482	1	0.142857142857143			0	4
487	1	0.142857142857143			0	4
507	2	0.2	0	2		
519	1	0.1	0	4		
533	2	0.2	0	2		
688	1	1	1	4		
724	1	0.1	0	4		
733	1	0.142857142857143			0	4
734	2	0.285714285714286			0	2
735	1	0.125	0	4		
736	1	0.111111111111111			0	4
753	1	1	1	4		
776	1	0.142857142857143			0	4
777	1	0.142857142857143			0	4
780	1	0.125	0	4		
783	2	0.266666666666667			0	2
784	2	0.45	0	2		
814	1	0.333333333333333			0	4
911	1	0.125	0	4		
915	1	0.25	0	4		
956	1	0.1	0	4		
960	1	0.111111111111111			0	4
980	1	0.333333333333333			0	4
1006	1	0.111111111111111			0	4
1008	1	0.1	0	4		
1012	1	0.1	0	4		
1013	1	0.1	0	4		
1014	1	0.1	0	4		
1015	2	0.2	0	2		
1034	1	0.1	0	4		
1050	1	0.1	0	4		
1055	1	0.1	0	4		
1057	1	0.1	0	4		
1058	1	0.1	0	4		
1062	1	0.1	0	4		
1067	1	0.142857142857143			0	4
1148	1	0.2	0	4		
1157	4	0.4	0	2		
1158	1	0.1	0	4		
1159	1	0.1	0	4		
1163	1	0.1	0	4		
1165	1	0.1	0	4		
1167	1	0.1	0	4		
1168	1	0.1	0	4		
1335	2	0.285714285714286			0	2
1336	2	2	2	0		
1338	1	0.142857142857143			0	4
1343	1	0.2	0	4		
1346	1	0.142857142857143			0	4
1347	1	0.142857142857143			0	4
1348	1	0.142857142857143			0	4
1354	1	0.166666666666667			0	4
1491	1	0.1	0	4		

1557	1	0.1	0	4	<<<
1583	2	0.4	0	2	
1584	1	0.1	0	4	
1619	1	0.2	0	4	
1728	1	0.166666666666667	0	4	
1731	1	0.125	0	4	
1740	2	0.25	0	2	
1744	1	0.166666666666667	0	4	
1750	1	0.142857142857143	0	4	

category=0, cleavage_site=1440
query=ptc-miR1447, target=Potri.019G108200.1,
score=2, range=1429-1449, strand=1

```
target  5' AAUGAAGGCACuGCAAUUCUU 3'
              ::: ::::::::::::::::
query   3' UUAGUUCCGUGACGUUAAGAC 5'
```

>Potri.019G108200.1
#size=1803

147	2	0.2	0	2
293	1	0.1	0	4
407	1	0.1	0	4
413	4	0.4	0	2
417	8	0.8	0	2
490	1	0.1	0	4
497	1	0.1	0	4
498	1	0.125	0	4
499	4	0.5	0	2
500	2	0.25	0	2
501	2	0.2	0	2
511	1	0.1	0	4
512	6	0.6	0	2
513	2	0.2	0	2
514	1	0.1	0	4
517	1	0.1	0	4
524	1	0.1	0	4
527	11	1.1	0	2
528	3	0.3	0	2
530	1	0.1	0	4
544	1	0.333333333333333	0	4
577	1	0.125	0	4
581	1	0.1	0	4
583	1	0.333333333333333	0	4
584	2	0.222222222222222	0	2
592	1	0.142857142857143	0	4
599	1	0.111111111111111	0	4
600	1	0.1	0	4
606	1	0.1	0	4
609	1	0.1	0	4
611	3	0.3	0	2
614	1	0.1	0	4
617	1	0.1	0	4
621	1	0.1	0	4
627	1	0.1	0	4
660	1	0.5	0	4
661	2	0.25	0	2
662	2	0.333333333333333	0	2
663	2	0.6	0	2
664	2	0.625	0	2

665	1	0.111111111111111	0	4	
668	2	0.267857142857143	0	2	
669	3	0.428571428571429	0	2	
682	1	0.1	0	4	
745	1	0.142857142857143	0	4	
793	1	0.1	0	4	
997	1	0.25	0	4	
1007	3	0.75	0	2	
1186	1	0.142857142857143	0	4	
1200	1	0.125	0	4	
1205	1	0.111111111111111	0	4	
1210	1	0.125	0	4	
1222	1	0.25	0	4	
1223	1	0.2	0	4	
1226	4	1.7	0	2	
1227	1	0.5	0	4	
1228	1	0.25	0	4	
1342	1	0.1	0	4	
1349	1	0.1	0	4	
1378	1	0.1	0	4	
1390	4	0.4	0	2	
1395	6	0.611111111111111	0	2	
1434	1	0.1	0	4	
1438	1	0.1	0	4	
1439	2	0.2	0	2	
1440	53	6.54563492063492	0	0	<<<
1470	4	0.4	0	2	
1471	1	0.1	0	4	
1472	1	0.1	0	4	
1473	3	0.3	0	2	
1475	4	0.533333333333333	0	2	
1476	3	0.533333333333333	0	2	
1477	16	1.75833333333333	0	2	
1478	7	0.966666666666667	0	2	
1479	12	1.2	0	2	
1480	10	1	0	2	
1481	4	0.425	0	2	
1482	5	0.5	0	2	
1483	4	0.4	0	2	
1484	2	0.2	0	2	
1485	1	0.1	0	4	
1488	1	0.142857142857143	0	4	
1492	2	0.366666666666667	0	2	
1493	3	0.5	0	2	
1509	1	0.111111111111111	0	4	
1511	1	0.1	0	4	
1561	1	0.333333333333333	0	4	
1573	3	1	0	2	
1612	2	0.2	0	2	
1622	1	0.1	0	4	
1639	3	0.3	0	2	
1658	1	0.166666666666667	0	4	
1659	4	0.5	0	2	
1679	2	0.2	0	2	
1688	1	0.1	0	4	
1748	1	0.142857142857143	0	4	

ptc-miR1450

category=4, cleavage_site=511
query=ptc-miR1450, target=Potri.004G215900.1,
score=2, range=498-520, strand=1

```
target  5' GUAGUCUGACCCGaGCCAUUGAA 3'
            ::::.:::: ::::::::::::
query   3' CAUUGGACU-GGCUCGGUAACUU 5'
```

>Potri.004G215900.1
#size=1031

33	2	2	2	2	
39	1	1	1	4	
68	1	1	1	4	
343	1	0.166666666666667	0	4	
359	1	0.166666666666667	0	4	
510	1	0.142857142857143	0	4	
511	1	0.142857142857143	0	4	<<<
523	18	2.57142857142857	0	2	
557	1	0.142857142857143	0	4	
572	1	0.142857142857143	0	4	
575	1	0.142857142857143	0	4	
576	1	0.142857142857143	0	4	
577	44	6.28571428571429	0	0	
587	14	2	0	2	
593	1	1	1	4	
594	3	3	3	2	
595	1	1	1	4	
738	1	0.2	0	4	
740	4	0.8	0	2	
743	1	0.25	0	4	
744	1	0.25	0	4	
745	3	0.75	0	2	
746	4	1	0	2	
748	1	0.25	0	4	
749	1	0.25	0	4	
750	1	0.25	0	4	
751	3	0.75	0	2	
752	1	0.25	0	4	
753	2	0.5	0	2	
755	2	0.5	0	2	
756	2	0.5	0	2	
757	1	0.25	0	4	
758	3	0.75	0	2	
760	1	0.25	0	4	
827	1	0.25	0	4	
828	8	2	0	2	
829	3	0.75	0	2	
830	2	0.5	0	2	
831	7	1.75	0	2	
832	5	1.25	0	2	
835	1	0.25	0	4	
836	2	0.5	0	2	
838	1	0.25	0	4	
847	1	0.25	0	4	
848	1	0.25	0	4	
851	2	0.5	0	2	
852	1	0.25	0	4	
914	1	1	1	4	

917	1	1	1	4
918	1	1	1	4
921	1	1	1	4
923	1	1	1	4
979	1	1	1	4

---

category=4, cleavage_site=391
query=ptc-miR1450, target=Potri.006G030400.1,
score=4, range=379-400, strand=1

```
target  5' GUAGCCUGGACGaGCCAUUGAU 3'
           :::.::::. :::::::::::
query   3' CAUUGGACUGGCUCGGUAACUU 5'
```

>Potri.006G030400.1
#size=540
1	1	0.2	0	4
146	1	0.5	0	4
385	1	0.333333333333333	0	4
391	1	0.333333333333333	0	4	<<<

ptc-miR475a,b

---

category=0, cleavage_site=521
query=ptc-miR475a,b, target=Potri.006G242200.1,
score=2, range=510-530, strand=1

```
target  5' CUUAAUCAAUGgGCUCUGUAA 3'
           :::::::::::::: ::::::
query   3' GAAUUAGUUACCCGUGACAUU 5'
```

>Potri.006G242200.1
#size=1773
223	1	0.125	0	4
237	1	0.1	0	4
483	1	1	1	4
521	13	2.125	0	0	<<<
594	1	0.333333333333333	0	4
614	1	0.125	0	4
663	1	0.142857142857143	0	4
953	1	0.125	0	4
1038	3	0.666666666666667	0	2
1091	1	0.166666666666667	0	4
1102	1	0.2	0	4
1103	1	0.25	0	4
1104	2	0.325	0	2
1118	1	0.111111111111111	0	4
1141	1	1	1	4
1165	1	0.111111111111111	0	4
1258	1	0.142857142857143	0	4
1359	1	0.142857142857143	0	4
1365	1	0.166666666666667	0	4
1385	1	0.25	0	4
1393	1	0.2	0	4
1515	1	0.125	0	4
1518	2	0.5	0	2
1551	1	1	1	4
1577	1	0.1	0	4
1583	1	0.1	0	4
1589	2	0.2	0	2
1619	1	0.25	0	4
1648	1	0.111111111111111	0	4

1701	1	1	1	4	

category=0, cleavage_site=790
query=ptc-miR475a,b, target=Potri.006G257300.1,
score=2, range=779-799, strand=1

```
target  5' CUUAAUCAAUGgGCUCUGUAA 3'
           ::::::::::::: ::::::
query   3' GAAUUAGUUACCCGUGACAUU 5'
```

>Potri.006G257300.1
#size=2403

23	1	0.333333333333333	0	4	
44	1	0.5	0	4	
99	1	0.5	0	4	
149	1	0.25	0	4	
191	1	0.25	0	4	
212	1	0.5	0	4	
492	1	0.125	0	4	
506	1	0.1	0	4	
790	15	3.125	0	0	<<<
1090	2	0.4	0	2	
1094	1	0.25	0	4	
1264	1	0.142857142857143	0	4	
1302	1	0.5	0	4	
1334	1	0.111111111111111	0	4	
1342	1	0.142857142857143	0	4	
1344	1	0.1	0	4	
1360	1	0.166666666666667	0	4	
1371	1	0.2	0	4	
1372	1	0.25	0	4	
1373	2	0.325	0	2	
1384	1	0.2	0	4	
1387	1	0.111111111111111	0	4	
1407	1	0.166666666666667	0	4	
1434	1	0.111111111111111	0	4	
1470	1	0.111111111111111	0	4	
1527	1	0.142857142857143	0	4	
1688	1	0.166666666666667	0	4	
1781	1	0.142857142857143	0	4	
1784	1	0.125	0	4	
1787	2	0.5	0	2	
1790	1	0.166666666666667	0	4	
1828	1	0.1	0	4	
1846	1	0.1	0	4	
1848	1	0.1	0	4	
1852	1	0.1	0	4	
1888	1	0.25	0	4	
1917	1	0.111111111111111	0	4	
1929	1	0.1	0	4	
2005	1	0.25	0	4	
2057	1	0.5	0	4	
2060	1	0.5	0	4	
2067	1	0.5	0	4	
2069	4	2	0	2	
2071	1	0.5	0	4	
2073	1	0.5	0	4	
2076	1	0.5	0	4	
2077	3	1.5	0	2	
2084	1	0.5	0	4	

2161	1	0.2	0	4
2176	2	0.7	0	2
2180	1	0.5	0	4
2182	2	0.7	0	2
2184	8	2.1	0	2
2188	1	0.166666666666667	0	4
2189	2	0.333333333333333	0	2
2190	2	0.333333333333333	0	2
2196	1	0.166666666666667	0	4
2198	1	0.166666666666667	0	4
2200	1	0.166666666666667	0	4
2201	1	0.166666666666667	0	4
2220	1	0.2	0	4
2226	1	0.2	0	4
2227	1	0.2	0	4
2234	1	0.2	0	4
2235	2	0.4	0	2
2244	1	0.2	0	4
2253	1	0.2	0	4
2255	1	0.2	0	4
2341	1	0.2	0	4
2344	1	0.2	0	4
2366	1	0.2	0	4

---

category=0, cleavage_site=400
query=ptc-miR475a,b, target=Potri.006G271200.1,
score=2, range=389-409, strand=1

```
target  5' CUUAAUCAAUGgGCUCUGUAA 3'
           ::::::::::::: ::::::
query   3' GAAUUAGUUACCCGUGACAUU 5'
```

>Potri.006G271200.1
#size=1798

119	1	0.5	0	4	
189	1	1	1	4	
223	1	0.25	0	4	
400	17	3.29166666666667	0	0	<<<
436	1	0.125	0	4	
473	1	0.333333333333333	0	4	
542	1	0.142857142857143	0	4	
700	2	0.4	0	2	
704	1	0.25	0	4	
832	1	0.125	0	4	
874	1	0.142857142857143	0	4	
944	1	0.111111111111111	0	4	
952	1	0.142857142857143	0	4	
954	1	0.1	0	4	
994	1	0.2	0	4	
997	1	0.111111111111111	0	4	
1044	1	0.111111111111111	0	4	
1110	1	0.25	0	4	
1137	1	0.142857142857143	0	4	
1192	1	1	1	4	
1238	1	0.142857142857143	0	4	
1244	1	0.166666666666667	0	4	
1264	1	0.25	0	4	
1272	1	0.2	0	4	
1391	1	0.142857142857143	0	4	
1394	1	0.125	0	4	

1397 2 0.5 0 2
1400 1 0.166666666666667 0 4
1456 1 0.1 0 4
1458 1 0.1 0 4
1462 1 0.1 0 4
1468 2 0.2 0 2
1473 1 0.142857142857143 0 4
1623 1 1 1 4
1631 1 1 1 4

---

category=0, cleavage_site=515
query=ptc-miR475a,b, target=Potri.006G271400.1,
score=2, range=504-524, strand=1

```
target  5' CUUAAUCAAUGgGCUCUGUAA 3'
           ::::::::::::: ::::::
query   3' GAAUUAGUUACCCGUGACAUU 5'
```

>Potri.006G271400.1
#size=1888
3 1 0.25 0 4
217 1 0.125 0 4
231 1 0.1 0 4
515 17 3.29166666666667 0 0 <<<
551 1 0.125 0 4
608 1 0.125 0 4
624 1 0.333333333333333 0 4
657 1 0.142857142857143 0 4
947 1 0.125 0 4
989 1 0.142857142857143 0 4
1032 2 0.333333333333333 0 2
1067 1 0.142857142857143 0 4
1069 1 0.1 0 4
1132 1 0.166666666666667 0 4
1195 1 0.111111111111111 0 4
1225 1 0.25 0 4
1227 1 0.333333333333333 0 4
1353 1 0.142857142857143 0 4
1359 1 0.166666666666667 0 4
1379 1 0.25 0 4
1387 1 0.2 0 4
1413 1 0.166666666666667 0 4
1506 1 0.142857142857143 0 4
1553 1 0.1 0 4
1571 1 0.1 0 4
1573 1 0.1 0 4
1577 1 0.1 0 4
1583 2 0.2 0 2
1624 1 0.2 0 4
1642 1 0.111111111111111 0 4
1722 1 1 1 4
1739 1 1 1 4

---

category=4, cleavage_site=1059
query=ptc-miR475a,b, target=Potri.011G057900.1,
score=3.5, range=1048-1068, strand=1

```
target  5' GUUAAUUAAUGgGCUCUGUAA 3'
             :::::.::::::: ::::::
query   3' GAAUUAGUUACCCGUGACAUU 5'
```

>Potri.011G057900.1
#size=1914

759	1	1	1	4	
851	1	1	1	4	
1059	1	1	1	4	<<<
1432	1	1	1	4	
1574	1	1	1	4	

---

category=4, cleavage_site=1140
query=ptc-miR475a,b, target=Potri.013G034400.1,
score=2, range=1129-1149, strand=1

```
target  5' ACUAAUCAAUGgGCACUGUAA 3'
             :::::::::::::::::::
query   3' GAAUUAGUUACCCGUGACAUU 5'
```

>Potri.013G034400.1
#size=2062

153	1	1	1	4	
524	1	0.5	0	4	
910	1	0.142857142857143	0	4	
1053	1	1	1	4	
1140	1	1	1	4	<<<
1534	1	0.25	0	4	

---

category=2, cleavage_site=639
query=ptc-miR475a,b, target=Potri.019G021200.1,
score=2, range=628-648, strand=1

```
target  5' CUUAAUCAAUGgGCUCUGUAA 3'
           :::::::::::::: ::::::
query   3' GAAUUAGUUACCCGUGACAUU 5'
```

>Potri.019G021200.1
#size=2286

46	1	0.5	0	4	
114	1	0.5	0	4	
341	1	0.125	0	4	
355	1	0.1	0	4	
639	9	1.625	0	2	<<<
675	1	0.125	0	4	
732	1	0.125	0	4	
860	1	0.5	0	4	
931	1	0.5	0	4	
933	1	0.5	0	4	
942	1	0.5	0	4	
1071	1	0.125	0	4	
1113	1	0.142857142857143	0	4	
1183	1	0.111111111111111	0	4	
1191	1	0.142857142857143	0	4	
1193	1	0.1	0	4	
1256	1	0.166666666666667	0	4	
1263	1	0.333333333333333	0	4	
1283	1	0.111111111111111	0	4	
1319	1	0.111111111111111	0	4	
1477	1	0.142857142857143	0	4	
1483	1	0.166666666666667	0	4	
1630	1	0.142857142857143	0	4	
1633	1	0.125	0	4	
1677	1	0.1	0	4	
1695	1	0.1	0	4	
1697	1	0.1	0	4	

1701	1	0.1	0	4	
1707	2	0.2	0	2	
1712	1	0.142857142857143	0	4	
1766	1	0.111111111111111	0	4	
1778	1	0.1	0	4	
1854	1	0.25	0	4	
1855	1	0.166666666666667	0	4	
1866	1	0.333333333333333	0	4	
1941	2	2	2	0	
1950	1	1	1	4	
2129	1	1	1	4	
2238	1	1	1	4	

ptc-miR482.1

category=2, cleavage_site=548
query=ptc-miR482.1, target=Potri.001G420000.1,
score=3, range=541-557, strand=1

```
target  5' GGCAUGGgAGGGGUGGG 3'
            :: :::::::.::.::
query   3' CCUUACCCUCCUCAUCC 5'
```

>Potri.001G420000.1
#size=4005

474	1	0.111111111111111	0	4	
532	1	0.1	0	4	
533	1	0.111111111111111	0	4	
534	1	0.1	0	4	
535	1	0.1	0	4	
537	1	0.1	0	4	
539	1	0.1	0	4	
541	1	0.1	0	4	
542	1	0.1	0	4	
543	1	0.1	0	4	
545	1	0.1	0	4	
547	1	0.1	0	4	
548	2	0.2	0	2	<<<
549	1	0.1	0	4	
579	1	0.25	0	4	
593	1	0.1	0	4	
596	1	0.1	0	4	
601	1	0.166666666666667	0	4	
627	1	0.2	0	4	
639	1	0.1	0	4	
644	1	0.25	0	4	
692	1	0.333333333333333	0	4	
695	1	0.25	0	4	
703	1	0.1	0	4	
704	1	0.1	0	4	
754	1	0.142857142857143	0	4	
764	1	0.1	0	4	
822	1	0.1	0	4	
883	1	0.25	0	4	
1650	1	0.1	0	4	
2258	1	1	1	4	
2864	1	0.1	0	4	
3165	1	0.1	0	4	
3168	1	0.1	0	4	
3171	2	0.2	0	2	

3457	1	0.1	0	4		
3533	1	0.1	0	4		
3539	1	0.1	0	4		

category=4, cleavage_site=685
query=ptc-miR482.1, target=Potri.001G422800.5,
score=3, range=678-694, strand=1

```
target  5' GGCAUGGgAGGGGUGGG 3'
           :: :::::::::.::.::
query   3' CCUUACCCUCCUCAUCC 5'
```

>Potri.001G422800.5
#size=4286

42	1	0.1	0	4		
158	1	0.1	0	4		
309	1	0.111111111111111			0	4
458	1	0.1	0	4		
459	1	0.1	0	4		
569	1	0.1	0	4		
577	1	0.1	0	4		
582	1	0.111111111111111			0	4
598	1	0.111111111111111			0	4
601	1	0.111111111111111			0	4
616	1	0.111111111111111			0	4
623	1	0.111111111111111			0	4
657	1	0.1	0	4		
658	1	0.1	0	4		
661	1	0.111111111111111			0	4
685	1	0.1	0	4	<<<	
705	1	0.1	0	4		
714	1	0.1	0	4		
717	1	0.1	0	4		
719	1	0.1	0	4		
750	1	0.1	0	4		
758	1	0.1	0	4		
765	1	0.1	0	4		
767	1	0.1	0	4		
833	1	0.1	0	4		
1454	1	0.1	0	4		
1607	1	0.1	0	4		
1657	1	0.1	0	4		
1720	1	0.1	0	4		
1820	1	0.1	0	4		
1958	1	0.1	0	4		
1961	1	0.1	0	4		
1974	1	0.1	0	4		
1995	1	0.1	0	4		
2000	1	0.1	0	4		
2013	1	0.1	0	4		
2022	1	0.1	0	4		
2025	1	0.1	0	4		
2026	1	0.1	0	4		
2031	1	0.1	0	4		
2045	1	0.1	0	4		
2054	1	0.1	0	4		
2068	1	0.1	0	4		
2365	1	0.1	0	4		
2425	1	0.1	0	4		
2436	2	0.2	0	1		

2477	1	0.1	0	4
2621	1	0.1	0	4
2783	1	0.1	0	4
2784	1	0.1	0	4
2795	1	0.1	0	4
2912	1	0.1	0	4
2916	1	0.1	0	4
2919	1	0.1	0	4
3034	1	0.1	0	4
3037	2	0.2	0	1
3491	1	0.1	0	4
3808	1	0.1	0	4
3816	1	0.1	0	4
3825	2	0.2	0	1
3827	1	0.1	0	4
3880	1	0.1	0	4
3933	1	0.1	0	4
3939	1	0.1	0	4
3975	1	0.1	0	4
3991	1	0.1	0	4
3994	1	0.1	0	4
4013	1	0.1	0	4
4021	1	0.1	0	4
4029	1	0.1	0	4
4044	1	0.1	0	4
4052	1	0.1	0	4
4125	2	0.2	0	1
4135	1	0.1	0	4

---

category=4, cleavage_site=548
query=ptc-miR482.1, target=Potri.001G426500.3,
score=3, range=541-557, strand=1

```
target  5' GGCAUGGgAGGGGUGGG 3'
           :: ::::::::.::.::
query   3' CCUUACCCUCCUCAUCC 5'
```

>Potri.001G426500.3
#size=4011

57	1	0.1	0	4
64	1	0.111111111111111	0	4
170	1	0.111111111111111	0	4
177	1	0.111111111111111	0	4
198	1	0.111111111111111	0	4
199	1	0.111111111111111	0	4
264	1	0.111111111111111	0	4
283	1	0.1	0	4
404	1	0.111111111111111	0	4
419	1	0.1	0	4
440	1	0.1	0	4
441	1	1	1	4
445	1	0.111111111111111	0	4
466	1	0.166666666666667	0	4
480	1	0.166666666666667	0	4
485	1	0.125	0	4
494	2	0.222222222222222	0	2
499	1	0.111111111111111	0	4
502	1	0.111111111111111	0	4
503	1	0.111111111111111	0	4
504	1	0.111111111111111	0	4

505	1	0.111111111111111			0	4
506	1	0.111111111111111			0	4
509	1	0.111111111111111			0	4
511	2	0.2	0	2		
513	2	0.2	0	2		
516	3	0.3	0	2		
518	4	0.4	0	2		
519	1	0.1	0	4		
520	1	0.1	0	4		
521	2	0.2	0	2		
522	2	0.2	0	2		
537	1	0.111111111111111			0	4
545	1	0.1	0	4		
548	1	0.1	0	4	<<<	
572	1	0.1	0	4		
576	1	0.111111111111111			0	4
628	1	0.1	0	4		
658	3	0.3	0	2		
668	1	0.1	0	4		
696	1	0.333333333333333			0	4
704	1	0.1	0	4		
962	1	0.1	0	4		
1654	1	0.1	0	4		
1715	1	0.1	0	4		
1791	1	0.333333333333333			0	4
1801	1	0.333333333333333			0	4
1833	1	0.1	0	4		
1837	1	0.1	0	4		
1868	1	0.1	0	4		
1893	1	0.1	0	4		
1908	1	0.1	0	4		
1912	1	0.1	0	4		
1918	1	0.1	0	4		
1922	1	0.1	0	4		
1924	1	0.1	0	4		
1990	1	0.1	0	4		
2382	1	0.1	0	4		
3005	1	0.111111111111111			0	4
3021	1	0.1	0	4		
3028	1	0.1	0	4		
3096	1	0.1	0	4		
3172	1	0.1	0	4		
3179	1	0.1	0	4		
3183	1	0.1	0	4		
3187	1	0.1	0	4		
3195	1	0.2	0	4		
3231	1	0.1	0	4		
3293	1	0.1	0	4		
3304	1	0.1	0	4		
3547	1	0.1	0	4		
3550	1	0.1	0	4		
3554	1	0.2	0	4		
3564	1	0.25	0	4		
3599	1	0.1	0	4		
3629	1	0.1	0	4		
3635	1	0.1	0	4		
3702	1	0.1	0	4		
3712	1	0.1	0	4		

3720	1	0.1	0	4
3761	1	0.1	0	4
3765	1	0.111111111111111	0	4
3773	1	0.1	0	4
3777	1	0.111111111111111	0	4
3780	2	0.211111111111111	0	2
3790	1	0.111111111111111	0	4
3850	1	0.1	0	4
3858	1	0.1	0	4
3876	1	0.1	0	4
3880	1	0.1	0	4
3891	2	0.222222222222222	0	2

category=2, cleavage_site=806
query=ptc-miR482.1, target=Potri.001G428100.1,
score=3, range=799-815, strand=1

```
target  5' GGCAUGGgAGGGGUGGG 3'
           :: :::::::::.::.::
query   3' CCUUACCCUCCUCAUCC 5'
```

>Potri.001G428100.1
#size=3262

107	1	0.1	0	4
113	1	0.1	0	4
118	1	0.1	0	4
280	1	0.1	0	4
326	1	0.1	0	4
427	2	0.2	0	2
429	1	0.1	0	4
430	1	0.111111111111111	0	4
433	2	0.2	0	2
434	1	0.1	0	4
440	1	0.1	0	4
464	1	0.1	0	4
497	1	0.1	0	4
505	1	0.1	0	4
539	1	0.111111111111111	0	4
548	1	0.111111111111111	0	4
605	1	0.111111111111111	0	4
612	1	0.111111111111111	0	4
614	1	0.111111111111111	0	4
615	1	0.111111111111111	0	4
648	1	0.5	0	4
656	2	0.666666666666667	0	0
677	1	0.1	0	4
690	1	0.1	0	4
703	1	0.111111111111111	0	4
719	1	0.111111111111111	0	4
722	1	0.111111111111111	0	4
737	1	0.111111111111111	0	4
743	1	0.125	0	4
744	1	0.111111111111111	0	4
745	1	0.333333333333333	0	4
748	2	0.222222222222222	0	2
750	1	0.111111111111111	0	4
751	2	0.222222222222222	0	2
752	1	0.111111111111111	0	4
753	1	0.111111111111111	0	4
757	1	0.111111111111111	0	4

760	1	0.111111111111111	0	4	
761	2	0.222222222222222	0	2	
762	1	0.111111111111111	0	4	
763	1	0.111111111111111	0	4	
764	1	0.111111111111111	0	4	
767	1	0.111111111111111	0	4	
769	2	0.2	0	2	
771	2	0.2	0	2	
774	4	0.4	0	2	
776	4	0.4	0	2	
777	1	0.1	0	4	
779	1	0.1	0	4	
780	1	0.1	0	4	
793	1	0.1	0	4	
795	1	0.111111111111111	0	4	
797	1	0.1	0	4	
799	1	0.1	0	4	
801	1	0.1	0	4	
805	1	0.1	0	4	
806	2	0.2	0	2	<<<
807	1	0.1	0	4	
897	1	0.1	0	4	
976	1	0.1	0	4	
990	1	0.1	0	4	
1007	1	0.1	0	4	
1217	1	0.1	0	4	
1287	1	0.1	0	4	
1341	1	0.166666666666667	0	4	
1401	1	0.1	0	4	
1909	1	0.1	0	4	
1925	1	0.1	0	4	
2004	1	0.1	0	4	
2085	1	0.1	0	4	
2097	1	0.1	0	4	
2105	1	0.1	0	4	
2158	1	0.1	0	4	
2176	1	0.1	0	4	
2190	1	0.1	0	4	
2262	1	0.1	0	4	
2272	1	0.1	0	4	
2283	1	0.1	0	4	
2288	1	0.1	0	4	
2429	1	0.5	0	4	
2445	1	0.166666666666667	0	4	
2450	1	0.111111111111111	0	4	
2457	1	0.1	0	4	
2458	1	0.1	0	4	
2464	1	0.1	0	4	
2465	1	0.1	0	4	
2474	1	0.1	0	4	
2574	1	0.1	0	4	
2615	1	0.1	0	4	
2619	1	0.1	0	4	
2649	1	0.1	0	4	
2723	1	0.1	0	4	
2725	2	0.2	0	2	
2730	1	0.1	0	4	
2731	1	0.1	0	4	

2735	1	0.1	0	4
2738	1	0.1	0	4
2928	1	0.1	0	4
2943	2	0.2	0	2
2945	1	0.1	0	4
3025	1	0.1	0	4
3033	1	0.1	0	4
3039	1	0.1	0	4
3047	1	0.1	0	4
3062	1	0.1	0	4
3076	1	0.1	0	4
3077	1	0.1	0	4
3083	1	0.1	0	4
3085	1	0.1	0	4
3091	2	0.2	0	2
3114	1	0.1	0	4
3162	2	0.2	0	2
3163	1	0.1	0	4
3164	1	0.1	0	4
3165	2	0.2	0	2
3168	1	0.1	0	4
3169	1	0.111111111111111	0	4
3170	1	0.1	0	4
3173	1	0.1	0	4
3175	1	0.1	0	4
3185	1	0.1	0	4
3189	1	0.111111111111111	0	4
3192	2	0.211111111111111	0	2
3203	1	0.2	0	4

---

category=2, cleavage_site=959
query=ptc-miR482.1, target=Potri.001G435100.1,
score=3, range=952-968, strand=1

```
target  5' GGCAUGGgAGGGGUGGG 3'
           :: ::::::::.::.::
query   3' CCUUACCCUCCUCAUCC 5'
```

>Potri.001G435100.1
#size=1719

58	1	0.5	0	4
95	2	2	2	0
203	1	0.333333333333333	0	4
230	1	0.2	0	4
244	1	0.333333333333333	0	4
287	1	0.2	0	4
289	1	0.166666666666667	0	4
334	1	0.1	0	4
609	1	0.111111111111111	0	4
610	1	0.111111111111111	0	4
644	1	0.1	0	4
735	1	0.111111111111111	0	4
742	1	0.111111111111111	0	4
913	2	0.222222222222222	0	2
914	1	0.111111111111111	0	4
915	1	0.111111111111111	0	4
916	1	0.111111111111111	0	4
917	1	0.111111111111111	0	4
920	1	0.111111111111111	0	4
922	3	0.3	0	2

924	2	0.2	0	2	
927	3	0.3	0	2	
929	4	0.4	0	2	
930	1	0.1	0	4	
932	1	0.1	0	4	
933	2	0.2	0	2	
935	1	0.111111111111111	0	4	
943	1	0.1	0	4	
945	1	0.1	0	4	
946	1	0.1	0	4	
948	1	0.111111111111111	0	4	
950	1	0.1	0	4	
952	1	0.1	0	4	
954	1	0.1	0	4	
958	1	0.1	0	4	
959	2	0.2	0	2	<<<
960	1	0.1	0	4	
1004	1	0.1	0	4	
1069	3	0.3	0	2	
1079	1	0.1	0	4	
1115	1	0.1	0	4	
1373	1	0.1	0	4	
1443	1	0.1	0	4	
1497	1	0.166666666666667	0	4	

---

category=2, cleavage_site=488
query=ptc-miR482.1, target=Potri.001G443700.1,
score=3, range=481-497, strand=1

```
target  5' GGCAUGGgAGGGGUGGG 3'
           :: ::::::::.::.::
query   3' CCUUACCCUCCUCAUCC 5'
```

>Potri.001G443700.1
#size=2628

456	1	0.1	0	4	
458	1	0.1	0	4	
462	2	0.2	0	2	
472	1	0.1	0	4	
473	1	0.111111111111111	0	4	
474	1	0.1	0	4	
475	1	0.1	0	4	
477	2	0.211111111111111	0	0	
479	1	0.1	0	4	
483	1	0.1	0	4	
485	1	0.1	0	4	
487	1	0.1	0	4	
488	2	0.2	0	2	<<<
489	1	0.1	0	4	
508	1	0.111111111111111	0	4	
511	1	0.166666666666667	0	4	
516	1	0.111111111111111	0	4	
795	1	0.1	0	4	
1256	1	0.1	0	4	
1324	1	0.1	0	4	
1408	1	0.111111111111111	0	4	
2043	1	0.1	0	4	
2046	1	0.1	0	4	
2174	1	0.1	0	4	
2327	1	0.1	0	4	

category=0, cleavage_site=597
query=ptc-miR482.1, target=Potri.001G444100.1,
score=3, range=590-606, strand=1

```
target  5' GGCAUGGgAGGGGUGGG 3'
           :: :::::::.::.::
query   3' CCUUACCCUCCUCAUCC 5'
```

>Potri.001G444100.1
#size=4231

76	1	0.1	0	4	
121	1	0.1	0	4	
541	1	0.111111111111111	0	4	
542	2	0.222222222222222	0	2	
544	1	0.111111111111111	0	4	
552	1	0.1	0	4	
570	1	0.1	0	4	
580	2	0.211111111111111	0	2	
582	1	0.1	0	4	
584	1	0.1	0	4	
585	1	0.111111111111111	0	4	
590	1	0.1	0	4	
591	1	0.1	0	4	
592	1	0.1	0	4	
596	1	0.1	0	4	
597	3	0.3	0	0	<<<
598	2	0.2	0	2	
600	2	0.2	0	2	
615	1	0.1	0	4	
756	1	0.2	0	4	
759	1	0.2	0	4	
953	1	0.111111111111111	0	4	
1186	1	0.125	0	4	
1880	2	0.2	0	2	
1887	1	0.1	0	4	
2029	1	0.1	0	4	
2283	1	0.1	0	4	
2917	1	0.166666666666667	0	4	
3158	1	0.1	0	4	
3168	1	0.1	0	4	
3233	1	0.1	0	4	
3282	1	0.1	0	4	
3662	1	0.1	0	4	
3672	1	0.1	0	4	
3751	1	0.1	0	4	
3786	1	0.1	0	4	
3901	1	0.111111111111111	0	4	
3919	1	0.1	0	4	
3957	2	0.2	0	2	
4038	1	0.2	0	4	

category=2, cleavage_site=548
query=ptc-miR482.1, target=Potri.001G445000.1,
score=3, range=541-557, strand=1

```
target  5' GGCAUGGgAGGGGUGGG 3'
           :: :::::::.::.::
query   3' CCUUACCCUCCUCAUCC 5'
```

>Potri.001G445000.1
#size=3099

493	1	0.111111111111111	0	4	
495	1	0.111111111111111	0	4	
518	2	0.211111111111111	0	2	
519	1	0.1	0	4	
521	1	0.1	0	4	
522	2	0.2	0	2	
532	1	0.1	0	4	
533	1	0.111111111111111	0	4	
534	1	0.1	0	4	
535	1	0.1	0	4	
537	1	0.1	0	4	
539	1	0.1	0	4	
542	1	0.1	0	4	
543	1	0.1	0	4	
545	1	0.1	0	4	
547	1	0.1	0	4	
548	2	0.2	0	2	<<<
549	1	0.1	0	4	
568	1	0.111111111111111	0	4	
571	1	0.166666666666667	0	4	
743	1	0.1	0	4	
749	1	0.1	0	4	
855	1	0.1	0	4	
1358	1	0.1	0	4	
1426	1	0.1	0	4	
1510	1	0.111111111111111	0	4	
2184	1	0.1	0	4	
2187	1	0.1	0	4	
2468	1	0.1	0	4	
2894	1	0.1	0	4	
2896	1	0.1	0	4	
2955	1	0.25	0	4	

---

category=4, cleavage_site=915
query=ptc-miR482a.1, target=Potri.001G445100.1,
score=2, range=908-924, strand=1

```
target  5' GGAAUGGgAGGAGGAGG 3'
           ::::::::::::: :::
query   3' CCUUACCCUCCUCAUCC 5'
```

>Potri.001G445100.1
#size=1001

440	1	1	1	4	
828	1	0.125	0	4	
867	1	0.166666666666667	0	4	
868	2	0.333333333333333	0	2	
876	1	0.125	0	4	
915	1	0.166666666666667	0	4	<<<
916	2	0.333333333333333	0	2	
919	1	0.5	0	4	
923	1	0.5	0	4	

---

category=2, cleavage_site=814
query=ptc-miR482.1, target=Potri.001G445900.1,
score=3, range=807-823, strand=1

```
target  5' GGCAUGGgAGGGGUGGG 3'
           :: :::::::::.::.::
query   3' CCUUACCCUCCUCAUCC 5'
```

>Potri.001G445900.1
#size=3296

130	1	0.333333333333333	0	4
168	1	0.142857142857143	0	4
187	1	0.166666666666667	0	4
293	1	0.1	0	4
338	1	0.1	0	4
758	1	0.111111111111111	0	4
759	1	0.111111111111111	0	4
761	1	0.111111111111111	0	4
769	1	0.1	0	4
787	1	0.1	0	4
788	1	0.1	0	4
799	1	0.1	0	4
801	1	0.1	0	4
802	1	0.111111111111111	0	4
807	1	0.1	0	4
808	1	0.1	0	4
809	1	0.1	0	4
813	2	0.2	0	2
814	3	0.3	0	2 <<<
815	1	0.1	0	4
817	2	0.2	0	2
832	1	0.1	0	4
973	1	0.2	0	4
976	1	0.2	0	4
1170	1	0.111111111111111	0	4
1403	1	0.125	0	4
2056	1	0.333333333333333	0	4
2107	1	0.1	0	4
2244	1	0.1	0	4
2468	1	0.1	0	4
2471	1	0.1	0	4
2599	1	0.111111111111111	0	4
2722	1	0.1	0	4
2726	1	0.1	0	4
2779	1	0.1	0	4
2883	1	0.2	0	4

---

category=4, cleavage_site=2410
query=ptc-miR482.1, target=Potri.005G083300.1,
score=3.5, range=2403-2419, strand=1

```
target  5' GAGAUGGgAGGAGUGGC 3'
             : .:::::::::::.:
query   3' CCUUACCCUCCUCAUCC 5'
```

>Potri.005G083300.1
#size=2818

273	1	1	1	4
416	1	1	1	4
567	1	0.333333333333333	0	4
778	1	0.333333333333333	0	4
800	1	0.333333333333333	0	4
967	1	0.333333333333333	0	4
998	1	0.25	0	4
1062	1	0.333333333333333	0	4
1242	1	1	1	4
1259	2	1	0	2
1288	2	2	2	1

1484	1	0.333333333333333	0	4	
1584	1	0.333333333333333	0	4	
1587	1	0.333333333333333	0	4	
1607	1	0.333333333333333	0	4	
1622	1	0.333333333333333	0	4	
1652	1	1	1	4	
1656	2	2	2	1	
1671	1	0.333333333333333	0	4	
1672	1	0.333333333333333	0	4	
1692	1	0.333333333333333	0	4	
1719	1	0.333333333333333	0	4	
1748	1	0.333333333333333	0	4	
1771	1	0.2	0	4	
1774	1	0.2	0	4	
1780	1	0.2	0	4	
1834	2	2	2	1	
1839	1	1	1	4	
1892	1	0.333333333333333	0	4	
1938	1	0.25	0	4	
1967	1	0.333333333333333	0	4	
2021	1	0.333333333333333	0	4	
2033	1	0.333333333333333	0	4	
2113	1	0.333333333333333	0	4	
2160	1	1	1	4	
2171	1	0.333333333333333	0	4	
2179	1	0.333333333333333	0	4	
2214	1	1	1	4	
2269	1	1	1	4	
2304	1	0.333333333333333	0	4	
2351	1	0.333333333333333	0	4	
2352	1	0.333333333333333	0	4	
2355	1	0.2	0	4	
2357	3	1	0	2	
2358	4	1.33333333333333	0	2	
2363	1	0.333333333333333	0	4	
2368	1	0.2	0	4	
2370	1	0.25	0	4	
2375	1	0.2	0	4	
2382	1	0.25	0	4	
2386	1	0.333333333333333	0	4	
2387	1	0.25	0	4	
2390	1	0.166666666666667	0	4	
2397	1	0.333333333333333	0	4	
2399	1	0.333333333333333	0	4	
2410	1	0.333333333333333	0	4	<<<
2417	1	1	1	4	
2420	1	1	1	4	
2432	2	2	2	1	
2492	1	0.333333333333333	0	4	
2534	1	0.333333333333333	0	4	
2550	1	1	1	4	
2555	1	1	1	4	
2566	2	2	2	1	
2609	1	0.333333333333333	0	4	
2632	1	1	1	4	
2648	2	2	2	1	
2659	1	0.333333333333333	0	4	
2682	1	1	1	4	

category=1, cleavage_site=1221
query=ptc-miR482.1, target=Potri.006G005800.1,
score=4, range=1214-1230, strand=1

```
target  5' AGAAGGGgAGGAGUGGG 3'
            ::: :::::::::.::
query   3' CCUUACCCUCCUCAUCC 5'
```

>Potri.006G005800.1
#size=1768

90	1	1	1	4	
383	1	1	1	4	
393	1	1	1	4	
633	1	1	1	4	
658	1	1	1	4	
679	1	1	1	4	
682	1	1	1	4	
685	1	1	1	4	
705	1	0.5	0	4	
734	1	1	1	4	
746	1	0.5	0	4	
775	1	0.5	0	4	
783	1	0.5	0	4	
809	2	1	0	1	
835	1	0.5	0	4	
864	1	0.5	0	4	
1209	1	0.5	0	4	
1221	2	1	0	1	<<<
1226	1	0.5	0	4	
1256	1	0.5	0	4	
1261	1	0.5	0	4	
1341	1	1	1	4	

category=4, cleavage_site=369
query=ptc-miR482.1, target=Potri.006G252300.1,
score=3, range=362-378, strand=1

```
target  5' GGAAUGGgAGGAGAAGA 3'
           :::::::::::: ::
query   3' CCUUACCCUCCUCAUCC 5'
```

>Potri.006G252300.1
#size=2149

244	1	1	1	4	
327	3	3	3	2	
334	2	2	2	2	
365	4	4	4	2	
366	1	1	1	4	
369	1	1	1	4	<<<
376	10	10	10	2	
378	3	3	3	2	
381	2	2	2	2	
415	1	1	1	4	
454	1	1	1	4	
463	1	1	1	4	
511	1	1	1	4	
1404	1	1	1	4	
1530	6	6	6	2	
1589	1	1	1	4	
1682	1	1	1	4	
1880	1	1	1	4	

1935	1	1	1	4
1949	7	7	7	2
1952	2	2	2	2
1953	1	1	1	4
1960	3	3	3	2
1962	1	1	1	4
1963	2	2	2	2
1964	1	1	1	4
1967	1	1	1	4
1968	2	2	2	2
1970	6	6	6	2
1971	10	10	10	2
1972	11	11	11	0
1973	4	4	4	2
1974	1	1	1	4
1994	1	1	1	4
2000	1	1	1	4
2021	1	1	1	4
2022	1	1	1	4
2024	1	1	1	4
2027	3	3	3	2
2031	2	2	2	2

---

category=2, cleavage_site=1897
query=ptc-miR482.1, target=Potri.008G066400.1,
score=4, range=1890-1905, strand=1

```
target  5' GGAUUGGgAGG-GUGGG 3'
           ::: ::::::: ::.::
query   3' CCUUACCCUCCUCAUCC 5'
```

>Potri.008G066400.1
#size=2588

207	1	0.333333333333333	0	4
214	1	0.2	0	4
428	1	0.333333333333333	0	4
456	1	0.333333333333333	0	4
680	2	0.666666666666667	0	0
725	1	0.166666666666667	0	4
786	1	0.166666666666667	0	4
897	1	0.166666666666667	0	4
952	1	0.166666666666667	0	4
1155	1	0.111111111111111	0	4
1307	1	0.1	0	4
1318	1	0.1	0	4
1383	1	0.166666666666667	0	4
1434	2	0.333333333333333	0	2
1544	1	0.166666666666667	0	4
1580	1	0.166666666666667	0	4
1598	1	0.333333333333333	0	4
1615	1	0.333333333333333	0	4
1655	1	0.166666666666667	0	4
1724	1	0.166666666666667	0	4
1745	1	0.166666666666667	0	4
1807	1	0.2	0	4
1818	1	0.2	0	4
1829	1	0.2	0	4
1834	1	0.2	0	4
1849	1	0.333333333333333	0	4
1850	1	0.333333333333333	0	4

1862	1	0.2	0	4	
1863	1	0.2	0	4	
1872	2	0.4	0	2	
1874	1	0.2	0	4	
1883	1	0.2	0	4	
1885	2	0.4	0	2	
1897	2	0.4	0	2	<<<
1910	1	0.2	0	4	
1911	1	0.2	0	4	
1925	1	0.333333333333333	0	4	
1959	1	0.333333333333333	0	4	
1971	1	0.333333333333333	0	4	
1973	1	0.333333333333333	0	4	

---

category=2, cleavage_site=628
query=ptc-miR482.1, target=Potri.009G095800.1,
score=3, range=621-637, strand=1

```
target  5' GGACUUGgAGGAGUAGG 3'
               ::: : ::::::::::
query   3' CCUUACCCUCCUCAUCC 5'
```

>Potri.009G095800.1
#size=1453

8	4	1.33333333333333	0	2
12	3	1	0	2
14	1	0.333333333333333	0	4
19	1	0.333333333333333	0	4
25	1	0.333333333333333	0	4
37	1	0.333333333333333	0	4
56	1	0.333333333333333	0	4
57	1	0.333333333333333	0	4
82	1	0.333333333333333	0	4
87	3	1	0	2
97	1	0.333333333333333	0	4
101	1	0.333333333333333	0	4
102	1	0.333333333333333	0	4
103	1	0.333333333333333	0	4
108	1	0.333333333333333	0	4
110	1	0.333333333333333	0	4
125	2	0.666666666666667	0	2
135	1	0.333333333333333	0	4
141	1	0.333333333333333	0	4
144	1	0.333333333333333	0	4
152	1	0.5	0	4
162	1	0.5	0	4
166	1	0.5	0	4
173	2	1	0	2
180	1	0.5	0	4
183	1	0.5	0	4
184	1	0.5	0	4
206	1	0.5	0	4
208	1	0.5	0	4
209	1	0.5	0	4
210	1	0.5	0	4
220	2	1	0	2
225	2	1	0	2
232	1	0.5	0	4
242	2	1	0	2
261	1	0.333333333333333	0	4

266	1	0.333333333333333	0	4
268	1	0.333333333333333	0	4
280	1	0.333333333333333	0	4
288	1	0.333333333333333	0	4
294	1	0.333333333333333	0	4
295	1	0.333333333333333	0	4
298	3	1	0	2
299	1	0.333333333333333	0	4
309	1	0.333333333333333	0	4
311	3	1	0	2
315	1	0.333333333333333	0	4
316	1	0.333333333333333	0	4
318	2	0.666666666666667	0	2
322	1	0.333333333333333	0	4
351	1	0.333333333333333	0	4
361	1	0.333333333333333	0	4
369	1	0.333333333333333	0	4
379	1	0.333333333333333	0	4
397	1	0.333333333333333	0	4
403	1	0.333333333333333	0	4
410	1	0.333333333333333	0	4
416	2	0.666666666666667	0	2
427	3	1	0	2
441	1	0.333333333333333	0	4
446	1	0.333333333333333	0	4
452	1	0.333333333333333	0	4
455	1	0.333333333333333	0	4
461	1	0.5	0	4
462	3	1.5	0	2
472	1	0.5	0	4
477	1	0.5	0	4
480	1	0.5	0	4
482	1	0.5	0	4
486	1	0.5	0	4
487	1	0.5	0	4
491	1	0.333333333333333	0	4
493	1	0.333333333333333	0	4
500	1	0.333333333333333	0	4
504	1	0.333333333333333	0	4
514	2	0.666666666666667	0	2
515	1	0.333333333333333	0	4
520	1	0.333333333333333	0	4
522	1	0.333333333333333	0	4
523	2	0.666666666666667	0	2
525	1	0.333333333333333	0	4
526	1	0.333333333333333	0	4
527	1	0.333333333333333	0	4
528	2	0.666666666666667	0	2
530	1	0.333333333333333	0	4
532	5	1.66666666666667	0	2
533	1	0.333333333333333	0	4
534	1	0.333333333333333	0	4
536	1	0.333333333333333	0	4
540	3	1	0	2
543	2	0.5	0	2
544	1	0.25	0	4
547	1	0.25	0	4
550	3	0.75	0	2

554	2	0.583333333333333	0	2	
559	1	0.25	0	4	
560	2	0.5	0	2	
562	1	0.25	0	4	
563	1	0.25	0	4	
564	1	0.25	0	4	
566	2	0.5	0	2	
570	2	0.5	0	2	
571	1	0.25	0	4	
572	1	0.25	0	4	
579	2	0.666666666666667	0	2	
580	1	0.333333333333333	0	4	
581	2	0.666666666666667	0	2	
582	3	1	0	2	
583	1	0.333333333333333	0	4	
585	2	0.666666666666667	0	2	
586	3	1	0	2	
587	1	0.333333333333333	0	4	
588	2	0.666666666666667	0	2	
589	1	0.25	0	4	
590	1	0.25	0	4	
591	1	0.25	0	4	
592	1	0.25	0	4	
593	2	0.5	0	2	
594	1	0.25	0	4	
595	2	0.5	0	2	
596	1	0.25	0	4	
597	1	0.25	0	4	
599	2	0.5	0	2	
602	1	0.25	0	4	
610	3	1	0	2	
612	2	0.666666666666667	0	2	
614	2	0.666666666666667	0	2	
615	2	0.666666666666667	0	2	
616	1	0.333333333333333	0	4	
618	3	1	0	2	
619	1	0.333333333333333	0	4	
621	1	0.333333333333333	0	4	
628	2	0.666666666666667	0	2	<<<
629	1	0.333333333333333	0	4	
630	1	0.333333333333333	0	4	
639	1	0.333333333333333	0	4	
641	1	0.25	0	4	
653	1	0.25	0	4	
662	1	0.25	0	4	
663	1	0.25	0	4	
666	1	0.25	0	4	
677	1	0.25	0	4	
679	1	0.25	0	4	
680	1	0.25	0	4	
690	1	0.333333333333333	0	4	
692	2	0.666666666666667	0	2	
693	1	0.333333333333333	0	4	
694	1	0.333333333333333	0	4	
699	1	0.333333333333333	0	4	
701	1	0.333333333333333	0	4	
702	1	0.25	0	4	
703	1	0.25	0	4	

705	1	0.333333333333333	0	4
715	1	0.333333333333333	0	4
717	1	0.333333333333333	0	4
718	1	0.333333333333333	0	4
723	1	0.333333333333333	0	4
724	1	0.333333333333333	0	4
727	1	0.333333333333333	0	4
739	1	0.333333333333333	0	4
740	1	0.333333333333333	0	4
742	1	0.333333333333333	0	4
744	1	0.333333333333333	0	4
747	1	0.333333333333333	0	4
750	1	0.333333333333333	0	4
753	1	0.333333333333333	0	4
760	4	1.33333333333333	0	2
766	1	0.333333333333333	0	4
770	3	1	0	2
772	1	0.333333333333333	0	4
776	1	0.333333333333333	0	4
779	1	0.333333333333333	0	4
780	4	1.33333333333333	0	2
781	1	0.333333333333333	0	4
783	2	0.666666666666667	0	2
786	1	0.333333333333333	0	4
788	2	0.666666666666667	0	2
789	1	0.333333333333333	0	4
791	1	0.333333333333333	0	4
795	1	0.333333333333333	0	4
796	3	1	0	2
798	3	1	0	2
800	1	0.333333333333333	0	4
801	1	0.333333333333333	0	4
803	2	0.666666666666667	0	2
804	1	0.333333333333333	0	4
805	3	1	0	2
807	2	0.666666666666667	0	2
809	1	0.333333333333333	0	4
810	2	0.666666666666667	0	2
812	1	0.333333333333333	0	4
815	2	0.666666666666667	0	2
816	6	2	0	2
817	1	0.333333333333333	0	4
820	1	0.333333333333333	0	4
828	1	0.333333333333333	0	4
830	1	0.333333333333333	0	4
831	1	0.333333333333333	0	4
832	3	1	0	2
833	2	0.666666666666667	0	2
834	3	1	0	2
836	2	0.666666666666667	0	2
838	1	0.333333333333333	0	4
839	1	0.333333333333333	0	4
840	1	0.333333333333333	0	4
844	2	0.666666666666667	0	2
848	3	1	0	2
853	2	0.583333333333333	0	2
858	2	0.5	0	2
861	2	0.666666666666667	0	2

865	1	0.333333333333333	0	4
866	2	0.666666666666667	0	2
867	1	0.333333333333333	0	4
868	5	1.58333333333333	0	2
869	5	1.66666666666667	0	2
870	1	0.333333333333333	0	4
871	2	0.5	0	2
872	3	0.75	0	2
874	4	1.08333333333333	0	2
876	2	0.583333333333333	0	2
879	1	0.333333333333333	0	4
880	1	0.333333333333333	0	4
881	1	0.333333333333333	0	4
882	1	0.333333333333333	0	4
887	1	0.25	0	4
889	4	1.16666666666667	0	2
890	2	0.666666666666667	0	2
892	3	1	0	2
895	3	0.75	0	2
896	1	0.333333333333333	0	4
897	1	0.333333333333333	0	4
898	2	0.666666666666667	0	2
899	1	0.333333333333333	0	4
900	1	0.333333333333333	0	4
901	2	0.666666666666667	0	2
902	3	0.833333333333333	0	2
903	1	0.333333333333333	0	4
904	7	2.33333333333333	0	0
905	2	0.666666666666667	0	2
907	3	1	0	2
908	1	0.333333333333333	0	4
909	5	1.66666666666667	0	2
910	2	0.666666666666667	0	2
911	1	0.333333333333333	0	4
912	1	0.333333333333333	0	4
913	1	0.333333333333333	0	4
914	4	1.33333333333333	0	2
915	2	0.666666666666667	0	2
916	2	0.666666666666667	0	2
918	2	0.583333333333333	0	2
919	2	0.666666666666667	0	2
921	2	0.583333333333333	0	2
922	3	1	0	2
924	3	1	0	2
925	3	1	0	2
926	2	0.666666666666667	0	2
927	7	2.16666666666667	0	2
928	3	0.916666666666667	0	2
929	1	0.25	0	4
930	1	0.25	0	4
931	5	1.25	0	2
932	2	0.5	0	2
933	9	2.25	0	2
934	1	0.25	0	4
935	3	0.75	0	2
936	1	0.25	0	4
937	5	1.25	0	2
938	3	0.75	0	2

939	3	0.75	0	2
940	4	1.16666666666667	0	2
941	2	0.5	0	2
942	4	1	0	2
943	2	0.5	0	2
944	3	0.75	0	2
945	3	0.833333333333333	0	2
947	1	0.25	0	4
948	1	0.25	0	4
949	1	0.25	0	4
950	1	0.25	0	4
951	2	0.5	0	2
952	1	0.25	0	4
953	2	0.5	0	2
956	2	0.5	0	2
957	1	0.25	0	4
958	2	0.5	0	2
959	1	0.25	0	4
960	1	0.25	0	4
961	2	0.5	0	2
964	3	0.75	0	2
965	1	0.25	0	4
967	1	0.333333333333333	0	4
968	1	0.25	0	4
969	1	0.25	0	4
970	1	0.25	0	4
971	5	1.58333333333333	0	2
972	2	0.666666666666667	0	2
973	2	0.583333333333333	0	2
974	3	0.916666666666667	0	2
975	2	0.5	0	2
976	2	0.5	0	2
977	2	0.5	0	2
978	6	1.5	0	2
980	2	0.5	0	2
981	2	0.583333333333333	0	2
983	2	0.666666666666667	0	2
984	2	0.666666666666667	0	2
990	1	0.333333333333333	0	4
993	1	0.333333333333333	0	4
995	1	0.333333333333333	0	4
998	1	0.333333333333333	0	4
999	2	0.666666666666667	0	2
1000	2	0.666666666666667	0	2
1001	3	0.75	0	2
1002	1	0.25	0	4
1003	4	1	0	2
1004	2	0.5	0	2
1005	1	0.25	0	4
1006	3	0.75	0	2
1007	3	0.75	0	2
1008	2	0.5	0	2
1009	4	1.08333333333333	0	2
1011	4	1	0	2
1012	1	0.25	0	4
1013	3	0.833333333333333	0	2
1014	4	1	0	2
1015	3	0.75	0	2

1016	1	0.25	0	4
1017	2	0.5	0	2
1018	5	1.25	0	2
1019	1	0.25	0	4
1020	1	0.25	0	4
1021	1	0.25	0	4
1022	4	1	0	2
1023	3	0.75	0	2
1024	2	0.5	0	2
1025	2	0.5	0	2
1026	1	0.25	0	4
1027	2	0.5	0	2
1028	1	0.25	0	4
1030	1	0.25	0	4
1032	1	0.25	0	4
1033	2	0.5	0	2
1034	3	0.75	0	2
1038	1	0.333333333333333	0	4
1040	1	0.333333333333333	0	4
1041	1	0.333333333333333	0	4
1043	4	1.33333333333333	0	2
1045	2	0.666666666666667	0	2
1047	1	0.333333333333333	0	4
1050	1	0.333333333333333	0	4
1052	1	0.333333333333333	0	4
1057	2	0.666666666666667	0	2
1058	1	0.25	0	4
1062	1	0.333333333333333	0	4
1064	1	0.333333333333333	0	4
1065	1	0.333333333333333	0	4
1067	1	0.333333333333333	0	4
1068	1	0.333333333333333	0	4
1069	1	0.333333333333333	0	4
1073	1	0.333333333333333	0	4
1075	2	0.666666666666667	0	2
1076	2	0.666666666666667	0	2
1077	1	0.333333333333333	0	4
1080	1	0.333333333333333	0	4
1081	2	0.666666666666667	0	2
1083	1	0.333333333333333	0	4
1089	1	0.333333333333333	0	4
1091	1	0.333333333333333	0	4
1094	1	0.333333333333333	0	4
1095	1	0.333333333333333	0	4
1096	1	0.333333333333333	0	4
1097	1	0.333333333333333	0	4
1098	1	0.333333333333333	0	4
1099	1	0.333333333333333	0	4
1102	1	0.333333333333333	0	4
1105	1	0.333333333333333	0	4
1110	1	0.333333333333333	0	4
1118	1	0.333333333333333	0	4
1132	1	0.333333333333333	0	4
1169	1	0.333333333333333	0	4
1171	1	0.333333333333333	0	4
1184	2	0.666666666666667	0	2
1188	2	0.666666666666667	0	2
1193	1	0.333333333333333	0	4

1199 1 0.333333333333333 0 4

---

category=2, cleavage_site=2062
query=ptc-miR482.1, target=Potri.010G191000.1,
score=4, range=2055-2070, strand=1

```
target  5' GGAUUGGgAGG-GUGGG 3'
           ::: ::::::: ::.::
query   3' CCUUACCCUCCUCAUCC 5'
```

>Potri.010G191000.1
#size=2863
723 1 0.333333333333333 0 4
835 1 0.333333333333333 0 4
890 1 0.166666666666667 0 4
951 1 0.166666666666667 0 4
1055 1 0.1 0 4
1062 1 0.166666666666667 0 4
1076 1 0.1 0 4
1117 1 0.166666666666667 0 4
1138 1 0.333333333333333 0 4
1472 1 0.1 0 4
1483 1 0.1 0 4
1487 1 0.142857142857143 0 4
1548 1 0.166666666666667 0 4
1599 2 0.333333333333333 0 2
1686 2 0.666666666666667 0 0
1709 1 0.166666666666667 0 4
1745 1 0.166666666666667 0 4
1820 1 0.166666666666667 0 4
1889 1 0.166666666666667 0 4
1910 1 0.166666666666667 0 4
1972 1 0.2 0 4
1981 1 0.5 0 4
1983 1 0.2 0 4
1994 1 0.2 0 4
1999 1 0.2 0 4
2008 1 0.5 0 4
2027 1 0.2 0 4
2028 1 0.2 0 4
2037 2 0.4 0 2
2039 1 0.2 0 4
2048 1 0.2 0 4
2050 2 0.4 0 2
2062 2 0.4 0 2 <<<
2075 1 0.2 0 4
2076 1 0.2 0 4
2089 1 0.5 0 4
2090 1 0.5 0 4
2094 1 0.5 0 4
2186 1 0.5 0 4
2192 1 0.5 0 4

---

category=2, cleavage_site=510
query=ptc-miR482a.1, target=Potri.015G115500.1,
score=4, range=503-519, strand=1

```
target  5' GGAAGGGgAGGAUUAGG 3'
          :::: ::::::: ::::
query   3' CCUUACCCUCCUCAUCC 5'
```

>Potri.015G115500.1

#size=886

112	1	0.2	0	4		
117	1	0.142857142857143			0	4
118	1	0.1	0	4		
120	1	0.1	0	4		
121	1	0.1	0	4		
128	1	0.1	0	4		
130	1	0.1	0	4		
136	1	0.333333333333333			0	4
137	1	0.333333333333333			0	4
139	8	2.66666666666667			0	2
140	3	1	0	2		
178	1	0.25	0	4		
213	1	0.25	0	4		
226	1	0.25	0	4		
245	1	0.5	0	4		
248	1	0.5	0	4		
270	1	0.25	0	4		
285	1	0.25	0	4		
308	2	0.5	0	2		
310	1	0.25	0	4		
316	1	0.25	0	4		
317	1	0.25	0	4		
318	1	0.25	0	4		
322	1	0.25	0	4		
323	1	0.25	0	4		
326	2	0.5	0	2		
327	1	0.25	0	4		
328	1	0.25	0	4		
337	1	0.25	0	4		
342	1	0.25	0	4		
346	1	0.25	0	4		
354	1	0.25	0	4		
355	1	0.25	0	4		
362	9	2.33333333333333			0	2
367	1	0.25	0	4		
372	2	0.5	0	2		
380	2	0.5	0	2		
382	1	0.25	0	4		
383	1	0.25	0	4		
387	5	1.5	0	2		
392	1	0.25	0	4		
393	2	0.75	0	2		
394	6	1.5	0	2		
395	1	0.25	0	4		
396	2	0.5	0	2		
398	1	0.25	0	4		
399	1	0.25	0	4		
401	2	0.5	0	2		
404	2	1	0	2		
406	1	0.25	0	4		
408	4	1	0	2		
409	1	0.25	0	4		
411	1	0.25	0	4		
415	1	0.25	0	4		
417	1	0.25	0	4		
429	1	0.25	0	4		
431	2	0.5	0	2		

432	1	0.25	0	4	
435	1	0.25	0	4	
455	1	0.25	0	4	
457	1	0.25	0	4	
458	1	0.25	0	4	
459	2	0.583333333333333	0	2	
461	1	0.333333333333333	0	4	
463	1	0.25	0	4	
465	1	0.25	0	4	
466	2	0.5	0	2	
468	1	0.25	0	4	
469	2	0.5	0	2	
470	1	0.25	0	4	
472	2	0.666666666666667	0	2	
473	1	0.333333333333333	0	4	
475	1	0.333333333333333	0	4	
480	1	0.25	0	4	
481	1	0.25	0	4	
491	1	0.333333333333333	0	4	
492	2	0.666666666666667	0	2	
496	1	0.333333333333333	0	4	
497	1	1	1	4	
498	3	1.66666666666667	1	2	
500	2	1.33333333333333	1	2	
501	2	1.33333333333333	1	2	
503	1	0.333333333333333	0	4	
505	1	0.333333333333333	0	4	
506	2	0.666666666666667	0	2	
508	2	0.5	0	2	
510	4	1	0	2	<<<
511	6	1.5	0	2	
513	8	2	0	2	
514	7	1.75	0	2	
515	4	1	0	2	
516	3	0.75	0	2	
525	1	0.25	0	4	
526	2	0.5	0	2	
527	2	0.5	0	2	
528	1	0.25	0	4	
531	5	1.25	0	2	
536	1	0.25	0	4	
537	26	6.5	0	2	
538	5	1.25	0	2	
539	2	0.5	0	2	
540	7	1.75	0	2	
541	12	3	0	2	
542	10	2.5	0	2	
543	9	2.25	0	2	
544	1	0.333333333333333	0	4	
546	4	1	0	2	
547	10	2.58333333333333	0	2	
548	10	2.75	0	2	
549	4	1	0	2	
550	3	0.75	0	2	
551	4	1	0	2	
553	1	0.5	0	4	
554	1	0.25	0	4	
555	1	0.25	0	4	

556	1	0.25	0	4	
557	1	0.333333333333333	0	4	
558	1	0.25	0	4	
559	2	0.5	0	2	
560	1	0.25	0	4	
561	2	0.75	0	2	
562	7	2.33333333333333	0	2	
563	2	0.666666666666667	0	2	
564	5	1.83333333333333	0	2	
565	5	1.25	0	2	
566	4	1.33333333333333	0	2	
567	3	1	0	2	
568	3	1.66666666666667	1	2	
569	1	0.333333333333333	0	4	
570	8	8	8	2	
571	5	5	5	2	
572	5	5	5	2	
579	1	1	1	4	
580	1	1	1	4	
581	7	7	7	2	
582	8	8	8	2	
583	3	3	3	2	
584	7	2.33333333333333	0	2	
585	2	0.666666666666667	0	2	
586	3	1	0	2	
587	1	0.333333333333333	0	4	
588	5	1.66666666666667	0	2	
589	2	0.666666666666667	0	2	
590	14	4.66666666666667	0	2	
591	9	3	0	2	
593	3	1	0	2	
594	3	1	0	2	
595	1	0.333333333333333	0	4	
601	1	0.333333333333333	0	4	
605	4	1.33333333333333	0	2	
606	4	1.33333333333333	0	2	
607	2	0.666666666666667	0	2	
608	25	8.33333333333333	0	0	
609	3	1	0	2	
610	2	0.666666666666667	0	2	
611	4	1.33333333333333	0	2	
612	1	0.333333333333333	0	4	
613	2	0.666666666666667	0	2	
614	1	0.333333333333333	0	4	
615	3	1	0	2	
616	2	0.666666666666667	0	2	
619	1	0.333333333333333	0	4	
620	1	0.333333333333333	0	4	
621	1	0.333333333333333	0	4	

---

category=2, cleavage_site=2162
query=ptc-miR482a.1, target=Potri.018G015200.1,
score=4, range=2155-2171, strand=1

```
target  5' UGAAUGGgAGAAGUGGG 3'
             :::::::: :::.::
query   3' CCUUACCCUCCUCAUCC 5'
```

>Potri.018G015200.1
#size=2650

312	1	0.166666666666667	0	4	
330	2	0.333333333333333	0	3	
339	1	0.166666666666667	0	4	
340	1	0.166666666666667	0	4	
342	1	0.166666666666667	0	4	
356	4	0.666666666666667	0	2	
357	3	0.5	0	2	
447	1	0.166666666666667	0	4	
1098	1	0.333333333333333	0	4	
1116	1	0.166666666666667	0	4	
1118	1	0.166666666666667	0	4	
1150	2	0.666666666666667	0	2	
1151	4	1.33333333333333	0	2	
1152	3	1	0	2	
1153	1	0.333333333333333	0	4	
1162	1	0.166666666666667	0	4	
1217	1	0.166666666666667	0	4	
2002	1	0.333333333333333	0	4	
2078	1	0.25	0	4	
2081	2	0.5	0	2	
2082	2	0.416666666666667	0	3	
2083	1	0.25	0	4	
2085	2	0.5	0	2	
2087	2	0.5	0	2	
2091	1	0.333333333333333	0	4	
2092	2	0.666666666666667	0	2	
2094	2	0.666666666666667	0	2	
2096	1	0.333333333333333	0	4	
2102	1	0.333333333333333	0	4	
2104	1	0.333333333333333	0	4	
2105	2	0.5	0	2	
2106	1	0.25	0	4	
2114	1	0.25	0	4	
2116	2	0.5	0	2	
2117	4	1	0	2	
2119	4	1	0	2	
2120	2	0.5	0	2	
2122	6	1.5	0	2	
2131	2	0.5	0	2	
2132	1	0.25	0	4	
2133	2	0.5	0	2	
2134	5	1.25	0	2	
2138	3	0.75	0	2	
2140	3	0.833333333333333	0	2	
2141	2	0.666666666666667	0	2	
2143	4	1.33333333333333	0	2	
2144	18	6	0	1	
2145	6	1.91666666666667	0	2	
2146	8	2.66666666666667	0	2	
2147	8	2.66666666666667	0	2	
2149	10	2.75	0	2	
2150	13	4.33333333333333	0	2	
2151	16	5.33333333333333	0	2	
2153	3	1	0	2	
2157	1	0.333333333333333	0	4	
2158	8	2.66666666666667	0	2	
2159	18	6	0	1	
2162	11	3.66666666666667	0	2	<<<

2163	2	0.666666666666667	0	2
2164	1	0.333333333333333	0	4
2165	1	0.333333333333333	0	4
2167	2	0.666666666666667	0	2
2168	2	0.666666666666667	0	2
2171	4	1.33333333333333	0	2
2172	2	0.666666666666667	0	2
2175	1	0.333333333333333	0	4
2176	1	0.333333333333333	0	4
2177	3	1	0	2
2179	6	2	0	2
2180	4	1	0	2
2186	4	1.33333333333333	0	2
2201	1	0.333333333333333	0	4
2202	1	0.333333333333333	0	4
2204	3	1	0	2
2205	2	0.666666666666667	0	2
2210	4	1.33333333333333	0	2
2212	1	0.333333333333333	0	4
2213	3	1	0	2
2214	2	0.666666666666667	0	2
2215	2	0.666666666666667	0	2
2216	2	0.666666666666667	0	2
2217	3	1	0	2
2218	1	0.333333333333333	0	4
2219	4	1.33333333333333	0	2
2220	10	2.91666666666667	0	2
2221	5	1.58333333333333	0	2
2222	12	3.66666666666667	0	2
2223	9	2.25	0	2
2224	2	0.5	0	2
2225	2	0.5	0	2
2226	1	0.25	0	4
2232	1	0.25	0	4
2233	3	0.75	0	2
2243	1	0.25	0	4
2246	1	0.25	0	4
2268	1	0.2	0	4
2274	2	0.4	0	3
2276	1	0.2	0	4
2278	2	0.4	0	3
2279	1	0.2	0	4
2280	3	0.733333333333333	0	2
2283	1	0.333333333333333	0	4
2284	2	0.4	0	3
2286	3	1	0	2
2287	1	0.333333333333333	0	4
2288	1	0.333333333333333	0	4
2292	1	0.2	0	4
2294	1	0.333333333333333	0	4
2295	1	0.333333333333333	0	4
2296	1	0.333333333333333	0	4
2298	1	0.333333333333333	0	4
2299	1	0.333333333333333	0	4
2316	1	0.333333333333333	0	4
2317	4	1.33333333333333	0	2
2318	6	2	0	2
2320	2	0.666666666666667	0	2

2321	4	1.33333333333333	0	2
2322	3	1	0	2
2323	1	0.333333333333333	0	4
2324	1	0.333333333333333	0	4
2326	1	0.333333333333333	0	4
2329	1	0.333333333333333	0	4
2330	1	0.333333333333333	0	4
2331	1	0.333333333333333	0	4
2333	3	1	0	2
2347	1	0.333333333333333	0	4
2348	3	1	0	2
2349	3	1	0	2
2358	1	0.2	0	4
2359	4	0.933333333333333	0	2
2360	1	0.333333333333333	0	4
2361	1	0.333333333333333	0	4
2363	1	0.333333333333333	0	4
2367	1	0.333333333333333	0	4
2369	1	0.333333333333333	0	4
2370	1	0.333333333333333	0	4
2374	1	0.333333333333333	0	4
2376	1	0.333333333333333	0	4
2379	2	0.666666666666667	0	2
2380	2	0.45	0	3
2399	1	0.2	0	4
2410	1	0.2	0	4
2427	1	0.333333333333333	0	4
2442	1	0.2	0	4
2454	1	0.333333333333333	0	4
2461	1	0.333333333333333	0	4
2462	1	0.333333333333333	0	4
2463	2	0.666666666666667	0	2
2464	1	0.333333333333333	0	4
2466	2	0.666666666666667	0	2
2467	4	1.33333333333333	0	2
2468	6	2	0	2
2470	2	0.666666666666667	0	2
2472	1	0.333333333333333	0	4
2473	1	0.333333333333333	0	4
2474	2	0.666666666666667	0	2
2491	1	0.333333333333333	0	4
2492	2	0.666666666666667	0	2
2493	2	0.666666666666667	0	2
2494	2	0.666666666666667	0	2
2495	1	0.333333333333333	0	4
2496	1	0.333333333333333	0	4
2497	8	2.66666666666667	0	2
2498	3	1	0	2
2499	1	0.333333333333333	0	4
2500	5	1.66666666666667	0	2
2501	2	0.666666666666667	0	2
2503	1	0.333333333333333	0	4
2504	2	0.666666666666667	0	2
2505	1	0.333333333333333	0	4
2506	1	0.333333333333333	0	4
2507	1	0.333333333333333	0	4
2508	1	0.333333333333333	0	4
2510	7	2.33333333333333	0	2

2511	4	1.33333333333333	0	2
2513	1	0.333333333333333	0	4
2514	2	0.666666666666667	0	2
2516	1	0.333333333333333	0	4
2517	2	0.666666666666667	0	2
2519	1	0.333333333333333	0	4
2521	2	0.666666666666667	0	2
2539	4	1.33333333333333	0	2
2542	1	0.333333333333333	0	4
2543	5	1.66666666666667	0	2
2544	2	0.666666666666667	0	2
2547	1	0.2	0	4
2548	6	1.2	0	2
2549	16	3.86666666666667	0	2
2550	16	3.2	0	2
2551	12	3.06666666666667	0	2
2552	7	2.33333333333333	0	2
2553	8	2.66666666666667	0	2
2554	5	1.66666666666667	0	2
2555	4	1.33333333333333	0	2
2557	2	0.666666666666667	0	2
2558	4	1.33333333333333	0	2
2560	1	0.333333333333333	0	4
2561	1	0.333333333333333	0	4
2571	5	1.66666666666667	0	2
2622	1	0.333333333333333	0	4
2623	2	0.666666666666667	0	2

---

category=4, cleavage_site=798
query=ptc-miR482.1, target=Potri.T087900.1,
score=3, range=791-807, strand=1

```
target  5' GGCAUGGgAGGGGUGGG 3'
            :: :::::::::.::.::
query   3' CCUUACCCUCCUCAUCC 5'
```

>Potri.T087900.1
#size=4812

53	1	0.25	0	4	
242	1	0.166666666666667	0	4	
766	1	0.1	0	4	
768	2	0.211111111111111	0	2	
769	1	0.1	0	4	
772	2	0.2	0	2	
773	1	0.1	0	4	
782	1	0.1	0	4	
784	1	0.1	0	4	
785	1	0.1	0	4	
787	1	0.1	0	4	
789	1	0.1	0	4	
792	1	0.1	0	4	
795	1	0.1	0	4	
798	1	0.1	0	4	<<<
846	1	0.1	0	4	
1495	1	0.25	0	4	
1980	1	0.1	0	4	
2122	1	0.1	0	4	
2184	1	0.1	0	4	
2233	1	0.1	0	4	
2365	1	0.1	0	4	

2520	1	1	1	4	
2525	1	1	1	4	
2814	1	0.1	0	4	
2815	1	0.1	0	4	
3364	1	0.1	0	4	
3374	1	0.1	0	4	
3488	1	0.1	0	4	
3697	1	0.1	0	4	
3868	1	0.1	0	4	
3878	1	0.1	0	4	
4125	1	0.1	0	4	
4343	1	0.1	0	4	
4455	1	0.25	0	4	
4463	1	0.166666666666667	0	4	
4471	1	0.125	0	4	
4480	1	0.125	0	4	
4585	1	0.2	0	4	
4659	1	0.1	0	4	
4734	1	0.1	0	4	

category=4, cleavage_site=548
query=ptc-miR482.1, target=Potri.T162100.1,
score=3, range=541-557, strand=1

```
target  5' GGCAUGGgAGGGGUGGG 3'
           :: :::::::.::.::
query   3' CCUUACCCUCCUCAUCC 5'
```

>Potri.T162100.1
#size=4520

27	1	0.1	0	4	
72	1	0.1	0	4	
490	1	0.111111111111111	0	4	
492	1	0.111111111111111	0	4	
493	1	0.111111111111111	0	4	
503	1	0.1	0	4	
520	1	0.1	0	4	
521	1	0.1	0	4	
524	2	0.2	0	2	
531	2	0.211111111111111	0	1	
533	2	0.2	0	2	
536	1	0.111111111111111	0	4	
542	1	0.1	0	4	
547	1	0.1	0	4	
548	1	0.1	0	4	<<<
549	2	0.211111111111111	0	1	
551	2	0.2	0	2	
566	1	0.1	0	4	
567	1	0.1	0	4	
628	1	0.1	0	4	
904	1	0.111111111111111	0	4	
1048	1	0.1	0	4	
1137	1	0.125	0	4	
1798	1	0.2	0	4	
2468	2	0.211111111111111	0	1	
2469	1	0.1	0	4	
2478	1	0.1	0	4	
2928	2	0.2	0	2	
3029	1	0.1	0	4	
3033	1	0.1	0	4	

3068	1	0.1	0	4
3239	2	0.2	0	2
3320	1	0.2	0	4
3660	1	0.1	0	4
3751	1	0.1	0	4
3971	2	0.2	0	2
4110	1	0.1	0	4
4293	1	0.2	0	4
4392	1	0.1	0	4

---

category=4, cleavage_site=548
query=ptc-miR482.1, target=Potri.T176000.1,
score=3, range=541-557, strand=1

```
target  5' GGCAUGGgAGGGGUGGG 3'
           :: ::::::::.::.::
query   3' CCUUACCCUCCUCAUCC 5'
```

>Potri.T176000.1
#size=1341

170	1	0.111111111111111	0	4	
199	1	0.111111111111111	0	4	
311	1	0.1	0	4	
348	1	0.111111111111111	0	4	
521	1	0.1	0	4	
522	1	0.1	0	4	
523	1	0.1	0	4	
533	1	0.1	0	4	
543	1	0.1	0	4	
545	1	0.1	0	4	
547	1	0.1	0	4	
548	1	0.1	0	4	<<<
621	1	0.1	0	4	
628	1	0.1	0	4	
1054	1	0.1	0	4	
1100	1	0.125	0	4	
1152	1	0.1	0	4	
1182	1	0.1	0	4	
1290	1	0.142857142857143	0	4	

ptc-miR482.2

---

category=0, cleavage_site=187
query=ptc-miR482.2, target=Potri.008G030000.1,
score=1.5, range=177-196, strand=1

```
target  5' GAUGAGAGGAgUAGGCAAGA 3'
           .::: :::::::::::::::
query   3' UUACCCUCCUCAUCCGUUCU 5'
```

>Potri.008G030000.1
#size=828

187	67	67	67	0	<<<
188	3	3	3	2	
225	1	1	1	4	
226	1	1	1	4	

category=0, cleavage_site=394
query=ptc-miR482.2, target=Potri.010G231200.1,
score=2, range=384-403, strand=1

```
target  5' AAUGGAAGGAgUGGGCAAGA 3'
           ::::: ::::::.:::::::
query   3' UUACCCUCCUCAUCCGUUCU 5'
```

>Potri.010G231200.1
#size=2058

169	1	0.5	0	4	
250	1	0.5	0	4	
253	2	1	0	2	
254	1	0.5	0	4	
257	2	1	0	2	
307	1	0.5	0	4	
374	1	0.5	0	4	
394	18	9	0	0	<<<
448	1	0.5	0	4	
536	1	0.5	0	4	
569	1	0.5	0	4	
571	1	0.5	0	4	
576	1	0.5	0	4	
587	1	0.5	0	4	
603	1	0.5	0	4	
697	1	0.5	0	4	
698	3	1.5	0	2	
732	1	0.5	0	4	
734	1	0.5	0	4	
746	1	0.5	0	4	
754	1	0.5	0	4	
758	1	0.5	0	4	
779	1	0.5	0	4	
781	1	0.5	0	4	
784	1	0.5	0	4	
785	1	0.5	0	4	
786	1	0.5	0	4	
788	2	1	0	2	
797	1	0.5	0	4	
808	1	0.5	0	4	
817	1	0.5	0	4	
877	1	0.5	0	4	
910	1	0.5	0	4	
911	1	0.5	0	4	
956	1	0.5	0	4	
1022	1	0.5	0	4	
1058	1	0.5	0	4	
1060	2	1	0	2	
1087	1	0.5	0	4	
1100	1	0.5	0	4	
1129	1	0.5	0	4	
1141	1	0.5	0	4	
1155	1	0.5	0	4	
1171	1	0.5	0	4	
1210	1	0.5	0	4	
1240	1	0.5	0	4	
1414	1	0.5	0	4	
1419	1	0.5	0	4	
1438	1	0.5	0	4	
1527	1	0.5	0	4	

1529	1	0.5	0	4	
1540	1	0.5	0	4	
1552	2	1	0	2	
1554	1	0.5	0	4	
1605	1	0.5	0	4	
1606	1	0.333333333333333	0	4	
1633	1	0.5	0	4	
1656	1	0.5	0	4	
1670	1	0.5	0	4	
1675	1	0.5	0	4	
1744	2	1	0	2	
1758	1	0.5	0	4	
1761	1	0.5	0	4	
1776	2	1	0	2	
1777	1	0.5	0	4	
1799	1	0.5	0	4	
1803	2	1	0	2	

---

category=0, cleavage_site=733
query=ptc-miR482.2, target=Potri.019G002600.1,
score=4, range=723-742, strand=1

```
target  5' AAUUGGCGGAaUAGGCAAGA 3'
           ::: :: ::: :::::::::
query   3' UUACCCUCCUCAUCCGUUCU 5'
```

>Potri.019G002600.1
#size=2937

70	1	1	1	4	
409	1	1	1	4	
733	2	2	2	0	<<<
871	1	1	1	4	
1013	1	1	1	4	
1547	1	1	1	4	
2530	1	1	1	4	
2586	1	1	1	4	

---

category=0, cleavage_site=259
query=ptc-miR482.2, target=Potri.T003600.1,
score=3.5, range=249-268, strand=1

```
target  5' GAUGGGCGGAgUGGGUAAGA 3'
           .::::: :::::.::.::::
query   3' UUACCCUCCUCAUCCGUUCU 5'
```

>Potri.T003600.1
#size=2666

61	1	0.125	0	4	
77	1	1	1	4	
180	2	0.666666666666667	0	2	
259	8	8	8	0	<<<
280	1	0.142857142857143	0	4	
326	1	0.125	0	4	
380	1	1	1	4	
452	1	0.125	0	4	
464	2	0.333333333333333	0	2	
473	1	0.142857142857143	0	4	
571	1	1	1	4	
674	1	0.333333333333333	0	4	
687	1	0.125	0	4	
699	1	0.1	0	4	
705	2	0.2	0	2	

713	1	0.1	0	4
748	1	0.111111111111111	0	4
767	2	0.5	0	2
773	1	0.1	0	4
775	2	0.222222222222222	0	2
785	3	0.3	0	2
786	1	0.1	0	4
788	1	0.1	0	4
795	1	0.1	0	4
806	1	0.1	0	4
812	1	0.142857142857143	0	4
822	1	0.333333333333333	0	4
824	2	0.666666666666667	0	2
835	1	1	1	4
848	1	0.1	0	4
894	1	0.142857142857143	0	4
906	1	0.125	0	4
926	1	0.166666666666667	0	4
934	1	0.1	0	4
939	1	0.1	0	4
977	1	1	1	4
1022	1	0.1	0	4
1028	1	0.1	0	4
1031	1	0.1	0	4
1038	1	0.1	0	4
1057	2	1	0	2
1225	1	1	1	4
1305	1	1	1	4
1488	1	0.5	0	4
1649	2	2	2	2
1698	1	0.111111111111111	0	4
1708	1	0.1	0	4
1709	1	0.1	0	4
1749	1	0.2	0	4
1766	1	0.166666666666667	0	4
1768	1	0.166666666666667	0	4
1849	1	1	1	4
1900	1	1	1	4
1967	1	1	1	4
1985	1	1	1	4
1992	1	0.1	0	4
2039	1	1	1	4
2065	1	0.1	0	4
2088	1	1	1	4
2092	1	1	1	4
2111	1	0.111111111111111	0	4
2112	1	0.1	0	4
2114	1	0.1	0	4
2125	1	1	1	4
2126	1	1	1	4
2329	1	0.333333333333333	0	4
2330	1	1	1	4
2331	1	1	1	4
2366	1	0.1	0	4
2367	2	0.211111111111111	0	2
2368	1	0.111111111111111	0	4
2485	1	1	1	4
2505	1	1	1	4

category=0, cleavage_site=799
query=ptc-miR482.2, target=Potri.T004200.1,
score=3.5, range=789-808, strand=1

```
target  5' GAUGGGCGGAgUGGGUAAGA 3'
            .::::: :::::.::.::::
query   3' UUACCCUCCUCAUCCGUUCU 5'
```

>Potri.T004200.1
#size=2913

182	4	0.5	0	2	
183	1	0.125	0	4	
185	1	0.125	0	4	
197	1	0.166666666666667	0	4	
198	1	0.166666666666667	0	4	
559	2	0.2	0	2	
567	2	0.2	0	2	
568	3	0.3	0	2	
571	2	0.2	0	2	
572	4	0.4	0	2	
574	8	0.85	0	2	
576	3	0.325	0	2	
595	3	0.6	0	2	
773	2	0.285714285714286	0	2	
786	2	0.285714285714286	0	2	
790	1	0.25	0	4	
799	21	5.4	0	0	<<<
865	3	0.5	0	2	
917	6	0.666666666666667	0	2	
919	1	0.111111111111111	0	4	
928	7	0.766666666666667	0	2	
929	1	0.1	0	4	
936	1	0.1	0	4	
937	4	0.4	0	2	
938	4	0.411111111111111	0	2	
939	1	0.1	0	4	
941	6	0.644444444444444	0	2	
948	3	0.3	0	2	
949	2	0.333333333333333	0	2	
950	3	0.3	0	2	
952	3	0.428571428571429	0	2	
953	3	0.619047619047619	0	2	
1315	1	0.1	0	4	
1440	1	0.1	0	4	
1471	1	0.1	0	4	
1473	1	0.1	0	4	
1479	2	0.3	0	2	
1483	1	0.1	0	4	
1490	1	0.1	0	4	
1493	1	0.1	0	4	
1495	3	0.3	0	2	
1496	1	0.1	0	4	
1498	1	0.1	0	4	
1850	1	0.333333333333333	0	4	
2244	1	0.2	0	4	
2284	3	0.333333333333333	0	2	
2665	1	0.125	0	4	
2666	1	0.125	0	4	
2730	1	0.1	0	4	

2732 1 0.1 0 4

ptc-miR530a

category=0, cleavage_site=307
query=ptc-miR530a, target=Potri.006G180500.1,
score=4, range=297-316, strand=1

```
target  5' GGGGUGCGGGuGCUAAUGCA 3'
              ..:::::.::::: ::::::
query   3' UUCCACGUCCACGUUUACGU 5'
```

>Potri.006G180500.1
#size=2120

151	1	0.333333333333333	0	4	
155	1	0.333333333333333	0	4	
180	1	0.5	0	4	
203	1	0.5	0	4	
204	2	0.833333333333333	0	2	
207	1	0.333333333333333	0	4	
246	1	0.5	0	4	
271	1	0.5	0	4	
283	1	0.333333333333333	0	4	
307	33	16.5	0	0	<<<
314	1	0.5	0	4	
327	1	0.5	0	4	
329	1	0.5	0	4	
337	1	0.333333333333333	0	4	
341	1	0.333333333333333	0	4	
343	3	1	0	2	
359	1	0.333333333333333	0	4	
380	1	0.333333333333333	0	4	
383	1	0.333333333333333	0	4	
387	1	0.333333333333333	0	4	
392	1	0.333333333333333	0	4	
398	1	0.333333333333333	0	4	
409	1	0.5	0	4	
446	1	0.5	0	4	
459	1	0.5	0	4	
460	1	0.5	0	4	
467	1	0.5	0	4	
474	1	0.5	0	4	
540	1	0.5	0	4	
571	2	1	0	2	
616	1	0.333333333333333	0	4	
621	2	0.666666666666667	0	2	
623	2	0.666666666666667	0	2	
627	1	0.333333333333333	0	4	
635	2	0.666666666666667	0	2	
642	2	0.666666666666667	0	2	
650	1	0.333333333333333	0	4	
659	1	0.333333333333333	0	4	
662	1	0.333333333333333	0	4	
667	1	0.333333333333333	0	4	
678	2	0.666666666666667	0	2	
733	1	0.5	0	4	
746	1	0.5	0	4	
761	1	0.333333333333333	0	4	
765	1	0.333333333333333	0	4	
768	1	0.333333333333333	0	4	

770	1	0.5	0	4
772	1	0.333333333333333	0	4
775	1	0.333333333333333	0	4
777	1	0.333333333333333	0	4
782	1	0.333333333333333	0	4
790	1	0.333333333333333	0	4
791	1	0.333333333333333	0	4
848	1	0.333333333333333	0	4
853	1	0.333333333333333	0	4
874	1	0.333333333333333	0	4
902	1	0.5	0	4
909	1	0.5	0	4
910	1	0.5	0	4
932	1	0.5	0	4
947	1	0.5	0	4
972	1	0.333333333333333	0	4
996	1	0.333333333333333	0	4
1002	1	0.333333333333333	0	4
1004	1	0.333333333333333	0	4
1012	1	0.333333333333333	0	4
1039	1	0.333333333333333	0	4
1047	1	0.333333333333333	0	4
1055	1	0.333333333333333	0	4
1064	1	0.333333333333333	0	4
1152	1	0.333333333333333	0	4
1167	1	0.333333333333333	0	4
1173	1	0.333333333333333	0	4
1181	2	0.666666666666667	0	2
1196	2	0.833333333333333	0	2
1197	3	1.5	0	2
1207	1	0.333333333333333	0	4
1208	1	0.333333333333333	0	4
1213	1	0.5	0	4
1224	1	0.5	0	4
1234	1	0.333333333333333	0	4
1294	1	0.333333333333333	0	4
1315	1	0.333333333333333	0	4
1335	1	0.333333333333333	0	4
1434	1	0.5	0	4
1463	1	0.5	0	4
1478	1	0.333333333333333	0	4
1489	1	0.333333333333333	0	4
1552	1	0.5	0	4
1595	2	2	2	2
1598	2	2	2	2
1599	1	1	1	4
1600	1	1	1	4
1604	1	1	1	4
1606	1	1	1	4
1611	1	1	1	4
1624	1	1	1	4
1632	1	1	1	4
1653	1	1	1	4
1662	1	0.5	0	4
1669	1	0.5	0	4
1704	2	0.666666666666667	0	2
1705	1	0.333333333333333	0	4
1708	1	0.333333333333333	0	4

1709	1	0.333333333333333	0	4
1710	1	0.5	0	4
1720	1	0.5	0	4
1721	2	1	0	2
1725	1	0.5	0	4
1726	1	0.5	0	4
1727	2	1	0	2
1728	2	1	0	2
1729	1	0.5	0	4
1731	1	0.5	0	4
1732	1	0.5	0	4
1733	1	0.333333333333333	0	4
1735	1	0.333333333333333	0	4
1737	2	0.666666666666667	0	2
1738	1	0.333333333333333	0	4
1741	1	0.5	0	4
1745	1	0.5	0	4
1748	1	0.333333333333333	0	4
1751	1	0.333333333333333	0	4
1754	1	0.333333333333333	0	4
1755	1	0.333333333333333	0	4
1756	1	0.333333333333333	0	4
1815	1	0.5	0	4
1817	1	0.5	0	4
1821	2	1	0	2
1822	1	0.5	0	4
1824	1	0.5	0	4
1833	3	1.5	0	2
1834	2	1	0	2
1840	1	0.5	0	4
1849	1	0.5	0	4
1858	1	0.5	0	4
1861	1	0.5	0	4
1862	1	0.5	0	4
1871	1	0.5	0	4
1875	1	0.5	0	4
1917	1	0.5	0	4
1937	1	0.5	0	4
2000	1	0.5	0	4
2019	1	0.5	0	4
2029	1	0.5	0	4
2031	1	0.5	0	4

---

category=0, cleavage_site=647
query=ptc-miR530a, target=Potri.008G162500.1,
score=1, range=637-656, strand=1

```
target  5' CAGGUGCAGGuGCAAAUGCA 3'
           ::::::::::::::::::::
query   3' UUCCACGUCCACGUUUACGU 5'
```

>Potri.008G162500.1
#size=4196

629	1	0.1	0	4	
647	15	3	0	0	<<<
648	1	0.2	0	4	
652	1	0.2	0	4	
655	1	0.2	0	4	
710	1	0.1	0	4	
717	2	0.2	0	2	

720	1	0.2	0	4		
794	1	0.2	0	4		
883	1	0.2	0	4		
885	1	0.2	0	4		
994	1	0.5	0	4		
1004	1	0.2	0	4		
1005	1	0.2	0	4		
1044	1	0.2	0	4		
1129	2	0.4	0	2		
1133	1	0.2	0	4		
1142	2	0.4	0	2		
1154	1	0.2	0	4		
1161	1	0.2	0	4		
1165	1	0.2	0	4		
1177	1	0.2	0	4		
1190	1	0.2	0	4		
1191	1	0.2	0	4		
1198	2	0.4	0	2		
1213	1	0.2	0	4		
1215	1	0.2	0	4		
1219	1	0.2	0	4		
1289	1	0.2	0	4		
1347	1	0.125	0	4		
1364	1	0.2	0	4		
1437	1	0.166666666666667			0	4
1464	1	0.166666666666667			0	4
1471	1	0.1	0	4		
1580	1	0.166666666666667			0	4
1606	2	0.333333333333333			0	2
1644	1	0.166666666666667			0	4
1650	1	0.166666666666667			0	4
1692	1	0.166666666666667			0	4
1702	1	0.1	0	4		
1782	1	0.1	0	4		
1850	1	0.1	0	4		
1856	1	0.1	0	4		
1914	1	0.166666666666667			0	4
1919	1	0.142857142857143			0	4
1925	1	0.142857142857143			0	4
1928	1	0.142857142857143			0	4
1947	1	0.142857142857143			0	4
1952	2	0.285714285714286			0	2
1954	1	0.142857142857143			0	4
1966	1	0.142857142857143			0	4
1999	1	0.2	0	4		
2034	1	0.1	0	4		
2052	1	0.142857142857143			0	4
2060	1	0.142857142857143			0	4
2096	2	0.4	0	2		
2112	1	0.142857142857143			0	4
2121	1	0.1	0	4		
2153	1	0.166666666666667			0	4
2171	2	0.2	0	2		
2249	1	0.166666666666667			0	4
2302	1	0.142857142857143			0	4
2357	2	0.285714285714286			0	2
2401	1	0.142857142857143			0	4
2410	1	0.142857142857143			0	4

2416	1	0.142857142857143	0	4
2484	1	0.142857142857143	0	4
2486	1	0.142857142857143	0	4
2494	1	0.142857142857143	0	4
2611	1	0.142857142857143	0	4
2637	1	0.142857142857143	0	4
2650	1	0.142857142857143	0	4
2672	1	0.142857142857143	0	4
2746	1	0.142857142857143	0	4
2773	1	0.142857142857143	0	4
2781	1	0.1	0	4
2802	1	0.1	0	4
2832	1	0.1	0	4
2896	1	0.1	0	4
2912	1	0.1	0	4
2914	1	0.1	0	4
2952	2	0.285714285714286	0	2
2961	1	0.142857142857143	0	4
3029	1	0.1	0	4
3069	1	0.1	0	4
3130	1	0.142857142857143	0	4
3166	1	0.142857142857143	0	4
3177	1	0.142857142857143	0	4
3180	1	0.142857142857143	0	4
3181	1	0.142857142857143	0	4
3184	1	0.142857142857143	0	4
3186	2	0.285714285714286	0	2
3188	1	0.142857142857143	0	4
3193	1	0.142857142857143	0	4
3194	1	0.142857142857143	0	4
3202	2	0.285714285714286	0	2
3206	1	0.142857142857143	0	4
3214	1	0.142857142857143	0	4
3215	1	0.142857142857143	0	4
3219	1	0.142857142857143	0	4
3284	1	0.142857142857143	0	4
3287	1	0.142857142857143	0	4
3290	1	0.142857142857143	0	4
3324	1	0.1	0	4
3362	1	0.142857142857143	0	4
3366	1	0.142857142857143	0	4
3373	1	0.142857142857143	0	4
3375	1	0.142857142857143	0	4
3376	1	0.142857142857143	0	4
3387	1	0.1	0	4
3394	1	0.142857142857143	0	4
3403	1	0.142857142857143	0	4
3437	1	0.166666666666667	0	4
3442	2	0.333333333333333	0	2
3446	1	0.166666666666667	0	4
3463	1	0.2	0	4
3505	1	0.166666666666667	0	4
3534	1	0.2	0	4
3555	2	0.333333333333333	0	2
3561	1	0.166666666666667	0	4
3586	1	0.2	0	4
3590	1	0.2	0	4
3593	1	0.2	0	4

3609	1	0.2	0	4
3610	4	0.8	0	2
3640	1	0.2	0	4
3677	1	0.2	0	4
3701	1	0.2	0	4
3703	1	0.2	0	4
3720	1	0.2	0	4
3721	1	0.2	0	4
3733	1	0.2	0	4
3734	1	0.2	0	4
3735	1	0.2	0	4
3738	1	0.2	0	4
3750	1	0.2	0	4
3822	1	0.2	0	4
3826	1	0.2	0	4
3831	1	0.2	0	4
3879	3	0.6	0	2
3889	1	0.2	0	4
3892	1	0.2	0	4
3893	1	0.2	0	4
3896	1	0.2	0	4
3899	1	0.2	0	4
3903	1	0.2	0	4
3909	1	0.2	0	4
3991	2	0.4	0	2
3993	1	0.2	0	4
3995	1	0.2	0	4
3997	1	0.2	0	4
4105	1	0.2	0	4

---

category=4, cleavage_site=1870
query=ptc-miR530a, target=Potri.010G062000.1,
score=4, range=1860-1879, strand=1

```
target  5' AAAUUGCAUGuGCAAAUGCA 3'
           ::  :::: :::::::::::
query   3' UUCCACGUCCACGUUUACGU 5'
```

>Potri.010G062000.1
#size=3133

21	1	1	1	4	
77	1	1	1	4	
128	1	1	1	4	
402	1	0.25	0	4	
403	1	0.25	0	4	
450	2	0.333333333333333	0	2	
452	1	0.166666666666667	0	4	
875	1	0.333333333333333	0	4	
949	1	0.333333333333333	0	4	
1417	1	0.166666666666667	0	4	
1442	1	0.333333333333333	0	4	
1659	1	0.166666666666667	0	4	
1700	1	0.166666666666667	0	4	
1763	1	0.166666666666667	0	4	
1797	1	0.166666666666667	0	4	
1848	1	0.166666666666667	0	4	
1870	1	0.333333333333333	0	4	<<<
1907	1	0.166666666666667	0	4	
1935	1	0.166666666666667	0	4	
1955	1	0.333333333333333	0	4	

1998	1	0.333333333333333	0	4
2012	1	0.166666666666667	0	4
2040	1	0.333333333333333	0	4
2052	1	0.333333333333333	0	4
2108	1	0.333333333333333	0	4
2156	1	0.166666666666667	0	4
2312	1	0.166666666666667	0	4
2483	1	0.166666666666667	0	4
2526	1	0.333333333333333	0	4
2564	1	0.333333333333333	0	4
2602	1	0.166666666666667	0	4
2615	1	0.166666666666667	0	4
2622	1	0.166666666666667	0	4
2666	1	0.333333333333333	0	4
2674	1	0.333333333333333	0	4
2677	1	0.333333333333333	0	4
2846	1	0.333333333333333	0	4
2977	1	0.333333333333333	0	4

---

category=2, cleavage_site=81
query=ptc-miR530a, target=Potri.010G076700.1,
score=1, range=71-90, strand=1

```
target  5' CAGGUGCAGGuGCAAAUGCA 3'
           ::::::::::::::::::::
query   3' UUCCACGUCCACGUUUACGU 5'
```

>Potri.010G076700.1
#size=3189

63	1	0.1	0	4	
81	3	0.6	0	2	<<<
144	1	0.1	0	4	
150	1	0.2	0	4	
151	2	0.2	0	2	
297	1	0.2	0	4	
327	1	0.2	0	4	
434	1	0.1	0	4	
535	1	0.2	0	4	
581	1	0.2	0	4	
710	1	0.1	0	4	
790	1	0.1	0	4	
858	1	0.1	0	4	
864	1	0.1	0	4	
957	1	0.2	0	4	
1020	1	0.2	0	4	
1042	1	0.1	0	4	
1085	2	0.4	0	2	
1097	1	0.2	0	4	
1129	1	0.1	0	4	
1179	2	0.2	0	2	
1297	1	0.2	0	4	
1449	1	0.2	0	4	
1794	1	0.2	0	4	
1798	1	0.2	0	4	
1822	1	0.1	0	4	
1852	1	0.1	0	4	
1853	2	0.4	0	2	
1861	1	0.2	0	4	
1864	1	0.2	0	4	
1916	1	0.1	0	4	

1921	1	0.2	0	4		
1932	1	0.1	0	4		
1934	1	0.1	0	4		
1986	1	0.2	0	4		
2003	1	0.2	0	4		
2021	1	0.142857142857143			0	4
2046	1	0.2	0	4		
2056	1	0.2	0	4		
2089	1	0.1	0	4		
2191	1	0.2	0	4		
2372	1	0.2	0	4		
2373	2	0.4	0	2		
2375	1	0.2	0	4		
2395	1	0.2	0	4		
2448	1	0.2	0	4		
2491	1	1	1	4		
2540	3	3	3	2		
2541	1	1	1	4		
2558	1	1	1	4		
2598	1	1	1	4		
2601	1	1	1	4		
2616	1	1	1	4		
2638	1	1	1	4		
2652	2	2	2	2		
2660	1	1	1	4		
2691	1	1	1	4		
2697	1	1	1	4		
2700	1	1	1	4		
2718	1	1	1	4		
2732	1	1	1	4		
2742	1	1	1	4		
2858	1	1	1	4		
2861	1	1	1	4		
2875	1	1	1	4		
2969	2	2	2	2		
2974	1	1	1	4		
3032	1	1	1	4		
3033	1	1	1	4		
3035	12	12	12	0		

---

category=0, cleavage_site=1945
query=ptc-miR530a, target=Potri.014G099700.1,
score=4, range=1935-1954, strand=1

```
target  5' CAAAAGCAGGuGCAAAUGCA 3'
             :   ::::::::::::::
query   3' UUCCACGUCCACGUUUACGU 5'
```

>Potri.014G099700.1
#size=3179

219	1	0.5	0	4		
228	1	0.25	0	4		
234	1	0.142857142857143			0	4
311	1	1	1	4		
387	1	1	1	4		
396	1	1	1	4		
425	1	1	1	4		
443	2	2	2	2		
446	1	1	1	4		
451	1	1	1	4		

480	1	1	1	4
530	1	1	1	4
533	1	1	1	4
544	1	1	1	4
564	2	2	2	2
582	1	1	1	4
636	1	1	1	4
713	1	1	1	4
783	1	1	1	4
784	1	1	1	4
943	1	0.5	0	4
955	1	1	1	4
970	1	1	1	4
972	1	1	1	4
973	1	1	1	4
1018	2	1	0	2
1050	1	0.5	0	4
1122	1	0.5	0	4
1156	1	0.5	0	4
1233	1	1	1	4
1244	1	1	1	4
1249	1	1	1	4
1369	1	1	1	4
1371	1	1	1	4
1395	1	0.5	0	4
1421	1	1	1	4
1449	1	0.5	0	4
1454	1	0.5	0	4
1458	1	0.5	0	4
1462	1	0.5	0	4
1487	1	1	1	4
1496	2	2	2	2
1517	2	2	2	2
1557	1	1	1	4
1579	1	1	1	4
1586	1	1	1	4
1608	2	2	2	2
1627	1	1	1	4
1713	1	1	1	4
1719	1	1	1	4
1720	1	1	1	4
1722	1	1	1	4
1776	1	1	1	4
1801	1	1	1	4
1807	1	1	1	4
1812	1	1	1	4
1832	1	1	1	4
1857	1	0.5	0	4
1862	1	0.5	0	4
1869	3	1.5	0	2
1871	1	0.5	0	4
1875	2	1	0	2
1876	1	0.5	0	4
1882	3	1.5	0	2
1883	1	0.5	0	4
1892	1	1	1	4
1896	2	2	2	2
1898	1	1	1	4

1901	1	1	1	4	
1902	1	1	1	4	
1906	1	1	1	4	
1910	1	1	1	4	
1921	2	2	2	2	
1935	1	0.5	0	4	
1939	1	0.5	0	4	
1944	2	2	2	2	
1945	14	14	14	0	<<<
1955	1	0.5	0	4	
1957	1	1	1	4	
1958	2	2	2	2	
1962	2	1	0	2	
1969	1	1	1	4	
1972	1	1	1	4	
1973	1	1	1	4	
1975	2	1.5	1	2	
1977	1	1	1	4	
1978	1	1	1	4	
1983	1	0.5	0	4	
1984	1	1	1	4	
1985	1	0.5	0	4	
1987	1	0.5	0	4	
1992	1	1	1	4	
2000	1	0.5	0	4	
2001	1	0.5	0	4	
2005	1	0.5	0	4	
2009	1	0.5	0	4	
2011	1	0.5	0	4	
2014	1	0.5	0	4	
2021	1	1	1	4	
2028	2	2	2	2	
2031	1	1	1	4	
2043	1	1	1	4	
2051	1	1	1	4	
2064	2	2	2	2	
2075	1	1	1	4	
2078	1	1	1	4	
2079	1	1	1	4	
2084	1	0.5	0	4	
2097	1	0.5	0	4	
2098	1	0.5	0	4	
2099	1	0.5	0	4	
2103	1	0.5	0	4	
2104	1	0.5	0	4	
2105	1	1	1	4	
2106	1	0.5	0	4	
2107	2	1	0	2	
2108	2	2	2	2	
2109	4	2	0	2	
2111	2	1	0	2	
2115	2	1	0	2	
2117	1	0.5	0	4	
2118	3	1.5	0	2	
2120	1	0.5	0	4	
2122	2	1	0	2	
2123	2	1	0	2	
2124	4	2	0	2	

2126	1	0.5	0	4
2128	2	1	0	2
2130	1	0.5	0	4
2137	2	1	0	2
2138	2	1	0	2
2144	1	0.5	0	4
2147	1	0.5	0	4
2149	1	0.5	0	4
2170	1	0.5	0	4
2181	1	0.5	0	4
2182	1	0.5	0	4
2185	1	0.5	0	4
2191	2	1	0	2
2193	1	0.5	0	4
2201	1	0.5	0	4
2203	1	0.5	0	4
2207	1	0.5	0	4
2213	1	0.5	0	4
2233	1	0.5	0	4
2238	2	1	0	2
2239	1	0.5	0	4
2279	1	1	1	4
2281	1	1	1	4
2283	2	2	2	2
2301	1	1	1	4
2317	1	1	1	4
2323	1	1	1	4
2389	1	0.5	0	4
2403	2	1	0	2
2406	2	1	0	2
2446	1	0.5	0	4
2451	1	1	1	4
2454	1	1	1	4
2455	1	0.5	0	4
2467	1	1	1	4
2472	1	1	1	4
2524	2	2	2	2
2529	2	1	0	2
2534	1	0.5	0	4
2537	1	0.5	0	4
2538	1	0.5	0	4
2547	1	0.5	0	4
2548	1	0.5	0	4
2549	1	0.5	0	4
2552	2	1	0	2
2553	2	1	0	2
2555	1	0.5	0	4
2568	1	1	1	4
2569	1	1	1	4
2572	1	1	1	4
2579	1	1	1	4
2599	1	1	1	4
2602	1	1	1	4
2605	1	1	1	4
2608	2	2	2	2
2612	1	1	1	4
2613	1	1	1	4
2621	1	1	1	4

2623	2	2	2	2
2629	2	2	2	2
2634	1	1	1	4
2641	1	1	1	4
2648	1	1	1	4
2657	1	1	1	4
2662	1	1	1	4
2663	1	1	1	4
2667	2	2	2	2
2669	2	2	2	2
2670	2	2	2	2
2672	2	2	2	2
2673	1	1	1	4
2677	2	2	2	2
2683	1	1	1	4
2688	2	2	2	2
2709	1	1	1	4
2713	1	1	1	4
2730	1	1	1	4
2739	1	1	1	4
2756	1	1	1	4
2784	1	1	1	4
2814	1	1	1	4
3104	1	1	1	4

category=0, cleavage_site=327
query=ptc-miR530a, target=Potri.018G102800.1,
score=4, range=317-336, strand=1

```
target  5' GGGGUGCGGGuGCUAAUGCA 3'
             ..:::::.::::: ::::::
query   3' UUCCACGUCCACGUUUACGU 5'
```

>Potri.018G102800.1
#size=2215

127	1	1	1	4	
165	1	0.333333333333333	0	4	
169	1	0.333333333333333	0	4	
205	1	1	1	4	
224	1	0.333333333333333	0	4	
227	1	0.333333333333333	0	4	
303	2	1.33333333333333	1	2	
306	2	2	2	2	
308	1	1	1	4	
314	1	1	1	4	
317	1	1	1	4	
322	2	2	2	2	
327	66	66	66	0	<<<
328	1	1	1	4	
330	1	1	1	4	
333	1	1	1	4	
339	1	1	1	4	
349	1	1	1	4	
357	1	0.333333333333333	0	4	
361	1	0.333333333333333	0	4	
363	3	1	0	2	
379	1	0.333333333333333	0	4	
400	1	0.333333333333333	0	4	
403	1	0.333333333333333	0	4	
407	1	0.333333333333333	0	4	

412	1	0.333333333333333			0	4
418	1	0.333333333333333			0	4
432	1	1	1	4		
445	1	1	1	4		
448	1	1	1	4		
452	2	2	2	2		
456	1	1	1	4		
460	1	1	1	4		
461	1	1	1	4		
462	2	2	2	2		
463	1	1	1	4		
471	1	1	1	4		
484	1	1	1	4		
496	1	1	1	4		
497	1	1	1	4		
515	1	1	1	4		
524	1	1	1	4		
573	1	1	1	4		
605	1	1	1	4		
639	1	0.333333333333333			0	4
644	2	0.666666666666667			0	3
646	2	0.666666666666667			0	3
650	1	0.333333333333333			0	4
658	2	0.666666666666667			0	3
665	2	0.666666666666667			0	3
673	1	0.333333333333333			0	4
682	1	0.333333333333333			0	4
685	1	0.333333333333333			0	4
690	1	0.333333333333333			0	4
701	2	0.666666666666667			0	3
738	2	2	2	2		
742	1	1	1	4		
759	1	1	1	4		
773	1	1	1	4		
784	1	0.333333333333333			0	4
788	1	0.333333333333333			0	4
791	1	0.333333333333333			0	4
795	1	0.333333333333333			0	4
798	1	0.333333333333333			0	4
800	1	0.333333333333333			0	4
805	1	0.333333333333333			0	4
813	1	0.333333333333333			0	4
814	1	0.333333333333333			0	4
871	1	0.333333333333333			0	4
876	1	0.333333333333333			0	4
897	1	0.333333333333333			0	4
906	1	1	1	4		
922	1	1	1	4		
995	1	0.333333333333333			0	4
1019	1	0.333333333333333			0	4
1025	1	0.333333333333333			0	4
1027	1	0.333333333333333			0	4
1035	1	0.333333333333333			0	4
1062	1	0.333333333333333			0	4
1070	1	0.333333333333333			0	4
1078	1	0.333333333333333			0	4
1087	1	0.333333333333333			0	4
1092	1	1	1	4		

1136	1	1	1	4		
1139	1	1	1	4		
1159	1	1	1	4		
1175	1	0.333333333333333			0	4
1190	1	0.333333333333333			0	4
1196	1	0.333333333333333			0	4
1204	2	0.666666666666667			0	3
1214	1	1	1	4		
1219	1	0.333333333333333			0	4
1230	1	0.333333333333333			0	4
1231	1	0.333333333333333			0	4
1236	1	1	1	4		
1239	1	1	1	4		
1257	1	0.333333333333333			0	4
1270	1	1	1	4		
1317	1	0.333333333333333			0	4
1338	1	0.333333333333333			0	4
1358	1	0.333333333333333			0	4
1501	1	0.333333333333333			0	4
1512	1	0.333333333333333			0	4
1548	1	1	1	4		
1612	1	1	1	4		
1623	1	1	1	4		
1636	1	1	1	4		
1638	1	1	1	4		
1645	1	1	1	4		
1667	2	2	2	2		
1674	1	1	1	4		
1701	1	1	1	4		
1704	1	1	1	4		
1708	1	1	1	4		
1727	2	0.666666666666667			0	3
1728	1	0.333333333333333			0	4
1731	1	0.333333333333333			0	4
1732	1	0.333333333333333			0	4
1745	1	1	1	4		
1748	1	1	1	4		
1749	1	1	1	4		
1756	1	0.333333333333333			0	4
1758	1	0.333333333333333			0	4
1760	2	0.666666666666667			0	3
1761	1	0.333333333333333			0	4
1771	1	0.333333333333333			0	4
1772	1	1	1	4		
1774	1	0.333333333333333			0	4
1777	1	0.333333333333333			0	4
1778	1	0.333333333333333			0	4
1779	1	0.333333333333333			0	4
1782	1	1	1	4		
1786	1	1	1	4		
1787	1	1	1	4		
1791	2	2	2	2		
1792	2	2	2	2		
1803	1	1	1	4		
1808	1	1	1	4		
1818	1	1	1	4		
1821	1	1	1	4		
1822	1	1	1	4		

1823	1	1	1	4
1825	2	2	2	2
1827	1	1	1	4
1829	1	1	1	4
1843	1	1	1	4
1844	1	1	1	4
1846	1	1	1	4
1851	1	1	1	4
1855	2	2	2	2
1859	1	1	1	4
1866	1	1	1	4
1869	1	1	1	4
1883	4	4	4	2
2029	1	1	1	4
2032	1	1	1	4
2043	1	1	1	4
2109	1	1	1	4

novel miRNAs

pto-miR001a,b

category=2, cleavage_site=2608
query=pto-miR001a,b, target=Potri.006G079300.1,
score=4, range=2598-2617, strand=1

```
target  5' UUGUGC-AUUUuGUCCUUUGU 3'
            :::::: ::::::::::.: :
query   3' AACACGUUAAAACAGGAGAAA 5'
```

>Potri.006G079300.1
#size=2710

258	1	0.142857142857143	0	4
291	1	0.142857142857143	0	4
457	1	0.142857142857143	0	4
469	1	0.5	0	4
610	1	0.125	0	4
613	1	0.125	0	4
647	1	0.125	0	4
759	1	0.125	0	4
764	2	0.222222222222222	0	3
772	3	0.333333333333333	0	3
783	3	0.375	0	3
813	4	0.5	0	2
864	2	0.2	0	3
871	1	0.1	0	4
890	1	0.1	0	4
937	5	0.5	0	2
950	3	0.3	0	3
951	4	0.4	0	3
957	1	0.1	0	4
1014	1	0.1	0	4
1262	2	0.2	0	3
1267	1	0.1	0	4
1313	1	0.1	0	4
1523	2	0.2	0	3
1880	1	0.125	0	4
1899	1	0.111111111111111	0	4
1945	1	0.111111111111111	0	4
1977	1	0.5	0	4
1980	1	0.5	0	4
2252	1	0.111111111111111	0	4
2276	1	0.111111111111111	0	4
2307	1	0.111111111111111	0	4
2310	1	0.111111111111111	0	4
2321	1	0.111111111111111	0	4
2322	1	0.111111111111111	0	4
2326	1	0.111111111111111	0	4
2327	1	0.111111111111111	0	4
2334	1	0.111111111111111	0	4
2369	1	0.5	0	4
2398	1	0.5	0	4
2414	1	0.5	0	4
2415	1	0.5	0	4
2417	1	0.5	0	4
2423	1	0.5	0	4
2432	1	0.5	0	4

2439	1	0.5	0	4	
2453	1	0.5	0	4	
2459	1	0.5	0	4	
2485	1	0.5	0	4	
2487	1	0.5	0	4	
2497	1	0.5	0	4	
2498	2	1	0	2	
2558	8	4	0	2	
2582	3	1.5	0	2	
2583	9	4.5	0	2	
2584	7	3.5	0	2	
2585	8	4	0	2	
2589	10	5	0	2	
2590	10	5	0	2	
2591	14	7	0	2	
2592	13	6.5	0	2	
2593	37	18.5	0	0	
2594	5	2.5	0	2	
2595	1	0.5	0	4	
2598	7	3.5	0	2	
2599	13	6.5	0	2	
2600	2	1	0	2	
2603	5	2.5	0	2	
2604	3	1.5	0	2	
2605	2	1	0	2	
2606	2	1	0	2	
2607	8	4	0	2	
2608	6	3	0	2	<<<
2609	19	9.5	0	2	
2610	5	2.5	0	2	
2611	4	2	0	2	
2612	3	1.5	0	2	
2613	10	5	0	2	
2614	8	4	0	2	
2615	22	11	0	2	
2616	16	8	0	2	
2617	1	0.5	0	4	
2618	1	0.5	0	4	
2620	2	1	0	2	
2621	15	7.5	0	2	
2622	4	2	0	2	
2623	1	0.5	0	4	
2624	2	1	0	2	
2625	5	2.5	0	2	
2627	6	3	0	2	
2628	13	6.5	0	2	
2629	4	2	0	2	
2630	2	1	0	2	
2631	1	0.5	0	4	
2632	1	0.5	0	4	
2633	4	2	0	2	
2638	1	0.5	0	4	
2639	2	1	0	2	
2642	1	0.5	0	4	
2643	1	0.5	0	4	
2647	2	1	0	2	
2655	5	2.5	0	2	
2656	1	0.5	0	4	

2662	2	1	0	2	
2665	1	0.5	0	4	
2668	1	0.5	0	4	
2669	1	0.5	0	4	
2672	1	0.5	0	4	
2682	3	1.5	0	2	

category=2, cleavage_site=1109
query=pto-miR001a,b, target=Potri.019G125000.1,
score=4, range=1099-1118, strand=1

```
target  5' UUGUUC-AUGUuGUCCUCUUU 3'
            :::: : :: :::::::::::
query   3' AACACGUUAAAACAGGAGAAA 5'
```

>Potri.019G125000.1
#size=1501

245	1	0.5	0	4	
248	4	2	0	2	
252	1	0.5	0	4	
277	1	0.333333333333333	0	4	
297	1	0.333333333333333	0	4	
298	1	0.333333333333333	0	4	
322	1	0.333333333333333	0	4	
330	4	1.33333333333333	0	2	
335	2	0.666666666666667	0	2	
337	1	0.142857142857143	0	4	
529	1	0.333333333333333	0	4	
534	2	0.4	0	3	
558	1	0.2	0	4	
560	1	0.2	0	4	
567	1	0.333333333333333	0	4	
643	3	0.6	0	3	
652	1	0.1	0	4	
660	1	0.142857142857143	0	4	
668	1	0.2	0	4	
670	1	0.2	0	4	
690	1	0.2	0	4	
782	100	20	0	2	
783	1	0.2	0	4	
784	1	0.2	0	4	
785	1	0.2	0	4	
790	1	0.2	0	4	
791	1	0.2	0	4	
801	1	0.2	0	4	
807	1	0.333333333333333	0	4	
812	5	1	0	2	
813	1	0.2	0	4	
822	3	1	0	2	
829	1	0.333333333333333	0	4	
841	1	0.2	0	4	
843	1	0.2	0	4	
844	3	0.733333333333333	0	2	
845	2	0.4	0	3	
846	1	0.333333333333333	0	4	
849	2	0.4	0	3	
851	1	0.2	0	4	
853	3	0.6	0	3	
854	4	0.8	0	2	
855	1	0.2	0	4	

856	3	0.6	0	3
858	3	0.6	0	3
859	6	1.2	0	2
860	4	0.8	0	2
862	2	0.4	0	3
864	1	0.2	0	4
867	1	0.2	0	4
868	4	0.8	0	2
869	3	0.6	0	3
870	2	0.4	0	3
871	3	0.6	0	3
872	1	0.2	0	4
873	1	0.2	0	4
876	3	0.6	0	3
879	5	1	0	2
880	2	0.4	0	3
881	12	2.4	0	2
883	2	0.666666666666667	0	2
887	3	1	0	2
892	2	0.666666666666667	0	2
898	1	0.333333333333333	0	4
902	2	0.666666666666667	0	2
906	1	0.333333333333333	0	4
907	16	3.2	0	2
908	2	0.4	0	3
909	2	0.4	0	3
910	2	0.4	0	3
911	5	1	0	2
912	3	0.6	0	3
913	3	0.6	0	3
914	1	0.2	0	4
915	12	2.4	0	2
916	9	1.8	0	2
917	3	0.6	0	3
918	15	3	0	2
919	3	0.6	0	3
920	16	3.2	0	2
921	15	3	0	2
922	6	1.2	0	2
923	9	1.8	0	2
927	3	0.6	0	3
928	1	0.2	0	4
929	1	0.2	0	4
930	6	1.2	0	2
932	2	0.4	0	3
933	6	1.2	0	2
943	1	0.333333333333333	0	4
950	1	0.333333333333333	0	4
951	1	0.333333333333333	0	4
952	1	0.333333333333333	0	4
953	1	0.333333333333333	0	4
964	2	0.666666666666667	0	2
965	3	1	0	2
970	1	0.333333333333333	0	4
976	1	0.333333333333333	0	4
981	1	0.333333333333333	0	4
984	1	0.333333333333333	0	4
987	2	0.666666666666667	0	2

991	7	2.2	0	2	
992	12	3.6	0	2	
993	31	7	0	2	
994	7	1.4	0	2	
995	4	0.8	0	2	
996	2	0.666666666666667	0	2	
998	2	0.666666666666667	0	2	
999	2	0.666666666666667	0	2	
1000	12	4	0	2	
1001	3	1	0	2	
1002	8	2.66666666666667	0	2	
1003	2	0.666666666666667	0	2	
1004	1	0.333333333333333	0	4	
1005	3	1	0	2	
1006	4	1.33333333333333	0	2	
1008	6	2	0	2	
1012	2	0.666666666666667	0	2	
1014	6	2	0	2	
1015	1	0.333333333333333	0	4	
1016	2	0.666666666666667	0	2	
1023	2	0.666666666666667	0	2	
1024	1	0.333333333333333	0	4	
1025	2	0.666666666666667	0	2	
1028	2	0.666666666666667	0	2	
1029	1	0.333333333333333	0	4	
1030	2	0.666666666666667	0	2	
1033	1	0.333333333333333	0	4	
1034	2	0.666666666666667	0	2	
1035	1	0.333333333333333	0	4	
1070	1	0.333333333333333	0	4	
1077	3	0.6	0	3	
1078	6	1.2	0	2	
1079	4	0.8	0	2	
1080	18	3.6	0	2	
1083	13	2.6	0	2	
1084	10	2	0	2	
1085	7	1.4	0	2	
1086	11	2.2	0	2	
1087	3	0.6	0	3	
1088	2	0.4	0	3	
1089	6	1.2	0	2	
1091	3	0.866666666666667	0	2	
1092	3	0.866666666666667	0	2	
1093	4	0.8	0	2	
1094	4	1.2	0	2	
1095	3	0.733333333333333	0	2	
1096	3	1	0	2	
1097	4	1.33333333333333	0	2	
1098	3	0.866666666666667	0	2	
1099	1	0.2	0	4	
1100	2	0.4	0	3	
1102	1	0.2	0	4	
1103	1	0.2	0	4	
1106	1	0.333333333333333	0	4	
1107	6	2	0	2	
1108	1	0.333333333333333	0	4	
1109	3	1	0	2	<<<
1114	1	0.333333333333333	0	4	

1115	1	0.333333333333333	0	4
1116	1	0.333333333333333	0	4
1117	4	1.33333333333333	0	2
1120	1	0.333333333333333	0	4
1122	2	0.666666666666667	0	2
1123	3	0.833333333333333	0	2
1124	5	1.66666666666667	0	2
1125	6	2	0	2
1126	4	1.33333333333333	0	2
1127	5	1.66666666666667	0	2
1128	3	1	0	2
1129	3	1	0	2
1130	9	3	0	2
1131	1	0.333333333333333	0	4
1132	2	0.666666666666667	0	2
1134	1	0.333333333333333	0	4
1135	7	2.33333333333333	0	2
1136	3	1	0	2
1137	23	7.66666666666666	0	2
1138	2	0.666666666666667	0	2
1139	11	3.66666666666667	0	2
1140	14	4.66666666666667	0	2
1141	10	3.33333333333333	0	2
1142	23	7.66666666666666	0	2
1143	14	4.66666666666667	0	2
1144	44	14.6666666666667	0	2
1145	100	33.3333333333333	0	2
1146	105	35	0	0
1147	29	9.66666666666667	0	2
1148	36	12	0	2
1149	33	11	0	2
1150	22	7.33333333333333	0	2
1151	31	10.3333333333333	0	2
1152	14	4.66666666666667	0	2
1153	5	1.66666666666667	0	2
1154	3	1	0	2
1155	2	0.666666666666667	0	2
1156	3	1	0	2
1157	4	1.33333333333333	0	2
1159	17	4.2	0	2
1160	9	2.33333333333333	0	2
1161	4	1.06666666666667	0	2
1162	6	1.33333333333333	0	2
1163	2	0.4	0	3
1165	2	0.4	0	3
1166	8	1.6	0	2
1167	2	0.4	0	3
1168	3	0.733333333333333	0	2
1169	5	1.53333333333333	0	2
1170	4	1.33333333333333	0	2
1171	1	0.333333333333333	0	4
1186	1	0.333333333333333	0	4
1250	1	0.333333333333333	0	4
1282	1	0.333333333333333	0	4
1325	2	0.666666666666667	0	2

pto-miR005a-c

category=4, cleavage_site=268
query=pto-miR005a-c, target=Potri.005G120500.1,
score=4, range=257-276, strand=1

```
target  5' CCUUACUAUGCaAA-UGGCUU 3'
            :::: :: :::::: ::::::
query   3' GGAACGAGACGUUUAACCGAA 5'
```

>Potri.005G120500.1
#size=1790

2	1	0.333333333333333	0	4	
268	1	0.333333333333333	0	4	<<<
286	1	0.333333333333333	0	4	
301	1	0.333333333333333	0	4	
379	1	0.333333333333333	0	4	
506	1	0.333333333333333	0	4	
688	1	0.333333333333333	0	4	
689	1	0.333333333333333	0	4	
694	2	0.666666666666667	0	2	
701	1	0.333333333333333	0	4	
707	1	0.333333333333333	0	4	
720	1	0.333333333333333	0	4	
721	1	0.333333333333333	0	4	
725	1	0.333333333333333	0	4	
757	1	0.333333333333333	0	4	
791	1	0.333333333333333	0	4	
812	1	0.333333333333333	0	4	
827	1	0.333333333333333	0	4	
879	1	0.333333333333333	0	4	
882	1	0.333333333333333	0	4	
941	1	0.333333333333333	0	4	
950	1	0.333333333333333	0	4	
1060	2	0.666666666666667	0	2	
1061	1	0.333333333333333	0	4	
1064	1	0.333333333333333	0	4	
1067	2	1.33333333333333	1	1	
1073	2	0.666666666666667	0	2	
1080	1	0.333333333333333	0	4	
1093	1	0.333333333333333	0	4	
1099	1	0.333333333333333	0	4	
1101	1	0.333333333333333	0	4	
1104	1	0.333333333333333	0	4	
1112	1	0.333333333333333	0	4	
1121	1	0.333333333333333	0	4	
1123	1	0.333333333333333	0	4	
1127	1	0.333333333333333	0	4	
1128	1	0.333333333333333	0	4	
1132	1	0.333333333333333	0	4	
1134	1	0.333333333333333	0	4	
1135	1	0.333333333333333	0	4	
1177	1	0.333333333333333	0	4	
1192	1	0.333333333333333	0	4	
1193	1	0.333333333333333	0	4	
1194	1	0.333333333333333	0	4	
1204	1	0.333333333333333	0	4	
1293	1	0.333333333333333	0	4	
1294	1	0.333333333333333	0	4	

1339	4	1.33333333333333	0	1	

---

category=4, cleavage_site=451
query=pto-miR005a-c, target=Potri.007G022000.1,
score=4, range=440-459, strand=1

```
target  5' CCUUACUAUGCaAA-UGGCUU 3'
            :::: :: :::::: ::::::
query   3' GGAACGAGACGUUUAACCGAA 5'
```

>Potri.007G022000.1
#size=2511

325	1	0.5	0	4	
451	1	0.333333333333333	0	4	<<<
469	1	0.333333333333333	0	4	
484	1	0.333333333333333	0	4	
562	1	0.333333333333333	0	4	
588	1	0.5	0	4	
657	2	1	0	2	
689	1	0.333333333333333	0	4	
704	1	0.5	0	4	
716	1	0.5	0	4	
723	1	0.5	0	4	
727	1	0.5	0	4	
776	1	0.5	0	4	
871	1	0.333333333333333	0	4	
872	1	0.333333333333333	0	4	
877	2	0.666666666666667	0	2	
884	1	0.333333333333333	0	4	
890	1	0.333333333333333	0	4	
903	1	0.333333333333333	0	4	
904	1	0.333333333333333	0	4	
908	1	0.333333333333333	0	4	
940	1	0.333333333333333	0	4	
974	1	0.333333333333333	0	4	
993	1	0.5	0	4	
995	1	0.333333333333333	0	4	
1001	1	0.5	0	4	
1008	1	0.5	0	4	
1010	1	0.333333333333333	0	4	
1023	1	0.5	0	4	
1040	1	0.5	0	4	
1042	2	1	0	2	
1062	1	0.333333333333333	0	4	
1065	1	0.333333333333333	0	4	
1092	1	0.5	0	4	
1100	1	0.5	0	4	
1115	1	0.5	0	4	
1124	1	0.333333333333333	0	4	
1133	1	0.333333333333333	0	4	
1148	2	1	0	2	
1154	1	0.5	0	4	
1155	1	0.5	0	4	
1156	1	0.5	0	4	
1157	1	0.5	0	4	
1159	1	0.5	0	4	
1165	1	0.5	0	4	
1167	1	0.5	0	4	
1170	1	0.5	0	4	
1171	1	0.5	0	4	

1174	1	0.5	0	4
1186	1	0.5	0	4
1199	1	0.5	0	4
1200	1	0.5	0	4
1202	1	0.5	0	4
1210	1	0.5	0	4
1214	1	0.5	0	4
1215	3	1.5	0	2
1221	1	0.5	0	4
1226	1	0.5	0	4
1231	2	1	0	2
1243	2	0.666666666666667	0	2
1244	1	0.333333333333333	0	4
1247	1	0.333333333333333	0	4
1250	1	0.333333333333333	0	4
1251	1	0.5	0	4
1256	2	0.666666666666667	0	2
1257	1	0.5	0	4
1263	1	0.333333333333333	0	4
1276	2	0.833333333333333	0	2
1282	1	0.333333333333333	0	4
1284	1	0.333333333333333	0	4
1287	1	0.333333333333333	0	4
1295	1	0.333333333333333	0	4
1304	1	0.333333333333333	0	4
1306	1	0.333333333333333	0	4
1310	1	0.333333333333333	0	4
1311	1	0.333333333333333	0	4
1315	1	0.333333333333333	0	4
1317	1	0.333333333333333	0	4
1318	1	0.333333333333333	0	4
1336	1	0.5	0	4
1340	1	0.5	0	4
1341	1	0.5	0	4
1346	1	0.5	0	4
1352	1	0.5	0	4
1356	1	0.5	0	4
1357	2	1	0	2
1360	1	0.333333333333333	0	4
1362	2	1	0	2
1363	1	0.5	0	4
1365	2	1	0	2
1366	1	0.5	0	4
1368	1	0.5	0	4
1369	1	0.5	0	4
1375	1	0.333333333333333	0	4
1376	1	0.333333333333333	0	4
1377	1	0.333333333333333	0	4
1387	1	0.333333333333333	0	4
1393	1	0.5	0	4
1398	2	1	0	2
1402	3	1.5	0	2
1403	1	0.5	0	4
1405	5	2.5	0	0
1408	1	0.5	0	4
1412	1	0.5	0	4
1417	1	0.5	0	4
1420	1	0.5	0	4

1424	1	0.5	0	4
1430	1	0.5	0	4
1434	1	0.5	0	4
1436	1	0.5	0	4
1443	1	0.5	0	4
1449	1	0.5	0	4
1476	1	0.333333333333333	0	4
1477	1	0.333333333333333	0	4
1503	1	0.5	0	4
1510	1	0.5	0	4
1522	4	1.33333333333333	0	2
1524	1	0.5	0	4
1530	1	0.5	0	4
1540	1	0.5	0	4
1553	1	0.5	0	4
1556	1	0.5	0	4
1559	2	1	0	2
1566	1	0.5	0	4
1568	1	0.5	0	4
1569	1	0.5	0	4
1572	1	0.5	0	4
1573	1	0.5	0	4
1577	1	0.5	0	4
1581	1	0.5	0	4
1582	1	0.5	0	4
1585	1	0.5	0	4
1589	1	0.5	0	4
1590	1	0.5	0	4
1591	1	0.5	0	4
1593	1	0.5	0	4
1596	2	1	0	2
1597	1	0.5	0	4
1599	1	0.5	0	4
1600	1	0.5	0	4
1601	1	0.5	0	4
1602	3	1.5	0	2
1603	1	0.5	0	4
1604	2	1	0	2
1608	2	1	0	2
1617	1	0.5	0	4
1619	1	0.5	0	4
1623	1	0.5	0	4
1626	1	0.5	0	4
1656	2	1	0	2
1671	1	0.5	0	4
1676	1	0.5	0	4
1677	1	0.5	0	4
1680	1	0.5	0	4
1681	1	0.5	0	4
1687	1	0.5	0	4
1693	1	0.5	0	4
1696	1	0.5	0	4
1697	1	0.5	0	4
1717	1	0.5	0	4
1793	1	0.5	0	4

category=4, cleavage_site=182
query=pto-miR005a-c, target=Potri.008G203800.1,
score=4, range=172-191, strand=1

```
target  5' CUCACUCUGCaGAUUGGUUU 3'
           ::  :::::::.:::::.::
query   3' GAACGAGACGUUUAACCGAA 5'
```

>Potri.008G203800.1
#size=736

14	1	1	1	4	
19	2	2	2	2	
21	1	1	1	4	
32	1	1	1	4	
121	1	1	1	4	
125	1	1	1	4	
141	2	2	2	2	
145	1	1	1	4	
148	1	1	1	4	
149	1	1	1	4	
156	1	1	1	4	
160	1	1	1	4	
170	1	1	1	4	
174	2	2	2	2	
182	1	1	1	4	<<<
183	1	1	1	4	
186	1	1	1	4	
214	1	1	1	4	
221	1	1	1	4	
223	1	1	1	4	
225	1	1	1	4	
229	1	1	1	4	
230	1	1	1	4	
257	1	1	1	4	
273	2	2	2	2	
275	1	1	1	4	
283	1	1	1	4	
288	1	1	1	4	
291	2	2	2	2	
295	1	1	1	4	
300	1	1	1	4	
303	1	1	1	4	
307	2	2	2	2	
308	2	2	2	2	
309	1	1	1	4	
310	2	2	2	2	
311	2	2	2	2	
312	1	1	1	4	
313	1	1	1	4	
314	1	1	1	4	
318	1	1	1	4	
320	1	1	1	4	
323	2	2	2	2	
324	1	1	1	4	
325	1	1	1	4	
327	3	3	3	2	
332	2	2	2	2	
333	7	7	7	0	
335	2	2	2	2	
336	1	1	1	4	

337	1	1	1	4
340	1	1	1	4
341	1	1	1	4
342	1	1	1	4
343	1	1	1	4
360	1	1	1	4
363	1	1	1	4
366	1	1	1	4
369	1	1	1	4
417	1	1	1	4
422	1	1	1	4
427	1	1	1	4
430	2	2	2	2
434	1	1	1	4
435	1	1	1	4
444	1	1	1	4
449	1	1	1	4
456	2	2	2	2
459	1	1	1	4
460	1	1	1	4
461	1	1	1	4
492	1	1	1	4
499	1	1	1	4
519	1	1	1	4
526	1	1	1	4
566	1	1	1	4
592	1	1	1	4
610	1	1	1	4
624	1	1	1	4

pto-miR009a,b

category=4, cleavage_site=1483
query=pto-miR009a,b, target=Potri.008G076300.1,
score=4, range=1473-1491, strand=1

```
target  5' CCAUGAAAUCuUUA-UUUCU 3'
           :::: : ::::::: :::::
query   3' GGUAGUAUAGAAAUCAAAGA 5'
```

>Potri.008G076300.1
#size=1823

528	1	0.125	0	4
535	1	0.125	0	4
768	1	0.333333333333333	0	4
952	2	0.666666666666667	0	3
1060	2	0.666666666666667	0	3
1097	1	0.333333333333333	0	4
1145	1	0.333333333333333	0	4
1149	1	0.333333333333333	0	4
1168	5	2.5	0	2
1200	2	1	0	2
1212	2	1	0	2
1248	1	0.5	0	4
1251	1	0.5	0	4
1254	1	0.5	0	4
1256	1	0.5	0	4
1257	3	1.5	0	2
1264	2	1	0	2

1271	5	2.5	0	2	
1284	2	2	2	2	
1294	1	1	1	4	
1296	3	3	3	2	
1297	1	1	1	4	
1298	2	2	2	2	
1309	1	1	1	4	
1314	1	1	1	4	
1321	1	1	1	4	
1419	1	1	1	4	
1458	1	1	1	4	
1466	1	1	1	4	
1474	1	1	1	4	
1483	1	1	1	4	<<<
1484	2	2	2	2	
1485	1	1	1	4	
1486	1	1	1	4	
1487	3	3	3	2	
1491	2	2	2	2	
1493	5	5	5	2	
1494	4	4	4	2	
1499	1	1	1	4	
1500	1	1	1	4	
1501	6	6	6	0	
1502	3	3	3	2	
1506	2	2	2	2	
1507	1	1	1	4	
1549	1	1	1	4	
1590	2	2	2	2	
1591	2	2	2	2	
1593	1	1	1	4	
1641	1	1	1	4	

category=4, cleavage_site=276
query=pto-miR009a,b, target=Potri.013G144000.1,
score=3.5, range=267-285, strand=1

```
target  5' UUAUU-UAUUuUUAGUUUCU 3'
           ..::. :::.::::::::::
query   3' GGUAGUAUAGAAAUCAAAGA 5'
```

>Potri.013G144000.1
#size=1918

4	1	1	1	4	
59	1	1	1	4	
143	1	1	1	4	
276	1	1	1	4	<<<
440	1	1	1	4	
443	1	1	1	4	
478	2	2	2	2	
480	2	2	2	2	
486	1	1	1	4	
493	1	1	1	4	
503	1	1	1	4	
543	1	0.5	0	4	
545	1	0.5	0	4	
548	2	1	0	2	
549	1	1	1	4	
552	1	1	1	4	
553	1	1	1	4	

557	1	1	1	4
578	1	1	1	4
617	3	3	3	2
628	1	1	1	4
630	1	1	1	4
656	1	0.5	0	4
663	1	1	1	4
664	1	0.5	0	4
666	1	0.5	0	4
675	1	0.5	0	4
682	1	0.5	0	4
685	3	2.5	2	2
686	2	1.5	1	2
687	1	1	1	4
688	1	0.5	0	4
696	1	1	1	4
699	2	2	2	2
701	1	0.5	0	4
704	1	0.5	0	4
705	1	0.5	0	4
713	1	0.5	0	4
719	1	0.5	0	4
721	1	0.5	0	4
724	1	0.5	0	4
726	2	1	0	2
731	1	0.5	0	4
732	1	0.5	0	4
734	1	0.5	0	4
739	1	1	1	4
742	1	1	1	4
744	1	1	1	4
748	1	1	1	4
760	1	1	1	4
761	1	1	1	4
762	1	1	1	4
770	1	1	1	4
777	1	1	1	4
778	1	1	1	4
783	1	1	1	4
797	1	1	1	4
798	1	1	1	4
809	1	1	1	4
819	1	0.5	0	4
826	1	0.5	0	4
829	2	1	0	2
837	1	0.5	0	4
839	1	0.5	0	4
850	1	1	1	4
855	1	0.5	0	4
858	1	1	1	4
863	1	1	1	4
864	1	1	1	4
865	1	1	1	4
866	4	4	4	0
867	1	1	1	4
870	1	1	1	4
878	1	1	1	4
881	1	1	1	4

884	2	2	2	2
888	2	2	2	2
898	1	1	1	4
899	1	1	1	4
905	1	1	1	4
907	2	2	2	2
909	2	2	2	2
915	1	1	1	4
933	1	1	1	4
942	1	1	1	4
954	2	2	2	2
964	1	1	1	4
967	1	1	1	4
984	1	1	1	4
988	1	1	1	4
995	1	1	1	4
1003	1	1	1	4
1025	1	0.5	0	4
1027	1	0.5	0	4
1038	1	0.5	0	4
1047	3	1.5	0	2
1048	2	1	0	2
1057	1	0.5	0	4
1066	1	0.5	0	4
1073	2	1.5	1	2
1075	2	2	2	2
1079	1	1	1	4
1081	1	0.5	0	4
1082	1	1	1	4
1113	1	1	1	4
1115	1	1	1	4
1119	1	0.5	0	4
1120	1	0.5	0	4
1122	1	0.5	0	4
1181	1	0.5	0	4
1183	1	0.5	0	4
1191	1	0.5	0	4
1214	1	1	1	4
1228	1	0.5	0	4
1229	1	0.5	0	4
1233	1	0.5	0	4
1235	1	0.5	0	4
1237	2	1	0	2
1243	1	0.5	0	4
1244	1	0.5	0	4
1247	1	0.5	0	4
1256	1	1	1	4
1257	1	1	1	4
1263	1	1	1	4
1274	2	2	2	2
1277	1	1	1	4
1293	1	0.5	0	4
1294	2	1	0	2
1298	1	0.5	0	4
1304	1	0.5	0	4
1314	1	0.5	0	4
1318	2	1	0	2
1321	2	1	0	2

1347	1	1	1	4
1350	1	1	1	4
1363	1	1	1	4
1367	1	1	1	4
1379	1	0.5	0	4
1390	1	0.5	0	4
1404	1	1	1	4
1408	2	1	0	2
1433	1	1	1	4
1441	1	1	1	4
1443	1	1	1	4
1444	1	1	1	4
1452	1	1	1	4
1455	2	2	2	2
1474	1	1	1	4
1476	1	1	1	4
1485	1	1	1	4
1620	1	1	1	4
1634	1	1	1	4
1636	1	1	1	4
1672	1	1	1	4
1680	1	1	1	4
1718	1	1	1	4
1752	1	1	1	4
1761	2	2	2	2
1767	1	1	1	4
1770	1	1	1	4
1773	1	1	1	4

pto-miR011a,b

category=4, cleavage_site=220
query=pto-miR011a,b, target=Potri.001G077200.1,
score=4, range=209-229, strand=1

```
target  5' AAGAGGAAACUaAUAUAAUCU 3'
           ::::::::::::. ::::::
query   3' CUCUCCUUUGAUUGCAUUAGA 5'
```

>Potri.001G077200.1
#size=453

21	1	1	1	4	
23	1	0.5	0	4	
54	2	0.5	0	2	
58	1	0.25	0	4	
66	1	0.25	0	4	
71	1	0.25	0	4	
96	1	0.25	0	4	
100	1	0.25	0	4	
101	3	0.75	0	2	
118	2	0.5	0	2	
162	1	0.25	0	4	
174	1	0.166666666666667	0	4	
190	2	0.277777777777778	0	2	
191	1	0.111111111111111	0	4	
208	1	0.166666666666667	0	4	
212	4	0.666666666666667	0	2	
220	1	0.166666666666667	0	4	<<<
227	1	0.166666666666667	0	4	

228	1	0.166666666666667	0	4	
229	2	0.5	0	2	
230	1	0.25	0	4	
236	1	0.1	0	4	
240	1	0.25	0	4	
290	2	1	0	1	
295	1	0.5	0	4	
343	1	0.25	0	4	

---

category=4, cleavage_site=217
query=pto-miR011a,b, target=Potri.T154400.1,
score=4, range=206-226, strand=1

```
target  5' AAGAGGAAACUaAUAUAAUCU 3'
            ::::::::::::. ::::::
query   3' CUCUCCUUUGAUUGCAUUAGA 5'
```

>Potri.T154400.1
#size=708

20	1	0.5	0	4	
51	2	0.5	0	2	
55	1	0.25	0	4	
63	1	0.25	0	4	
68	1	0.25	0	4	
93	1	0.25	0	4	
97	1	0.25	0	4	
98	3	0.75	0	0	
115	2	0.5	0	2	
159	1	0.25	0	4	
171	1	0.166666666666667	0	4	
187	2	0.277777777777778	0	2	
188	1	0.111111111111111	0	4	
205	1	0.166666666666667	0	4	
209	4	0.666666666666667	0	2	
217	1	0.166666666666667	0	4	<<<
224	1	0.166666666666667	0	4	
225	1	0.166666666666667	0	4	
237	1	0.25	0	4	
340	1	0.25	0	4	
376	1	0.5	0	4	
392	1	0.5	0	4	

pto-miR016a

---

category=4, cleavage_site=3180
query=pto-miR016a, target=Potri.004G169800.1,
score=1, range=3169-3189, strand=1

```
target  5' UCAAUCCAACAcCUCAACUUG 3'
           ::: :::::::::::::::::
query   3' AGUAAGGUUGUGGAGUUGAAC 5'
```

>Potri.004G169800.1
#size=3558

8	1	0.142857142857143	0	4
10	1	0.125	0	4
15	1	0.1	0	4
46	1	0.2	0	4
67	1	0.1	0	4
80	1	0.1	0	4
114	2	0.5	0	2

146	1	0.142857142857143	0	4
163	1	0.166666666666667	0	4
183	1	0.142857142857143	0	4
278	1	0.111111111111111	0	4
279	1	0.111111111111111	0	4
280	1	0.111111111111111	0	4
284	1	0.111111111111111	0	4
285	1	0.111111111111111	0	4
292	2	0.833333333333333	0	0
303	1	0.5	0	4
306	2	0.7	0	2
317	1	0.333333333333333	0	4
404	1	0.1	0	4
412	1	0.1	0	4
431	1	0.1	0	4
451	1	0.142857142857143	0	4
457	1	0.111111111111111	0	4
460	1	0.1	0	4
465	1	0.142857142857143	0	4
482	1	0.1	0	4
493	1	0.142857142857143	0	4
522	1	0.1	0	4
534	1	0.1	0	4
555	1	0.1	0	4
586	1	0.1	0	4
588	1	0.1	0	4
683	2	0.2	0	2
752	1	0.125	0	4
784	2	0.2	0	2
790	1	0.1	0	4
804	1	0.1	0	4
811	1	0.1	0	4
825	1	0.1	0	4
839	1	0.1	0	4
840	1	0.1	0	4
854	1	0.1	0	4
855	1	0.1	0	4
951	1	0.166666666666667	0	4
953	1	0.166666666666667	0	4
997	1	0.125	0	4
1011	1	0.25	0	4
1020	1	0.333333333333333	0	4
1031	1	0.111111111111111	0	4
1041	1	0.1	0	4
1058	1	0.1	0	4
1094	1	0.1	0	4
1097	1	0.1	0	4
1215	1	0.1	0	4
1280	1	0.1	0	4
1283	1	0.1	0	4
1390	1	0.1	0	4
1423	1	0.1	0	4
1448	1	0.1	0	4
1461	1	0.1	0	4
1509	1	0.1	0	4
1536	1	0.1	0	4
1545	1	0.1	0	4
1604	1	0.1	0	4

1615	1	0.1	0	4
1621	2	0.2	0	2
1626	1	0.1	0	4
1630	1	0.1	0	4
1632	2	0.2	0	2
1649	1	0.25	0	4
1661	1	0.25	0	4
1675	1	0.1	0	4
1677	1	0.1	0	4
1775	2	0.225	0	2
1837	1	0.1	0	4
1841	1	0.1	0	4
1844	1	0.1	0	4
1883	1	0.1	0	4
1885	1	0.1	0	4
1886	2	0.2	0	2
1890	1	0.1	0	4
1893	1	0.1	0	4
1894	2	0.2	0	2
1898	1	0.1	0	4
1900	2	0.2	0	2
1903	1	0.1	0	4
1904	1	0.1	0	4
1907	1	0.1	0	4
1914	1	0.1	0	4
1916	1	0.1	0	4
1917	1	0.1	0	4
1919	1	0.1	0	4
1930	1	0.1	0	4
1960	2	0.5	0	2
1964	1	0.2	0	4
1990	1	0.1	0	4
2118	1	0.1	0	4
2132	1	0.111111111111111	0	4
2200	1	0.1	0	4
2221	1	0.1	0	4
2223	1	0.1	0	4
2243	1	0.1	0	4
2244	1	0.1	0	4
2321	1	0.1	0	4
2339	2	0.2	0	2
2343	1	0.1	0	4
2377	1	0.1	0	4
2385	1	0.1	0	4
2425	1	0.1	0	4
2509	1	0.1	0	4
2553	1	0.1	0	4
2567	1	0.1	0	4
2628	1	0.1	0	4
2629	1	0.1	0	4
2635	1	0.1	0	4
2637	1	0.1	0	4
2655	1	0.333333333333333	0	4
2720	1	0.166666666666667	0	4
2791	1	0.1	0	4
2909	1	0.1	0	4
2967	1	0.1	0	4
2974	2	0.2	0	2

3060 1 0.1 0 4
3114 1 0.125 0 4
3120 2 0.2 0 2
3133 1 0.1 0 4
3138 2 0.25 0 2
3140 1 0.1 0 4
3143 1 0.1 0 4
3154 1 0.1 0 4
3171 1 0.1 0 4
3173 1 0.1 0 4
3180 1 0.1 0 4 <<<
3290 1 0.333333333333333 0 4
3298 1 0.1 0 4
3315 1 0.1 0 4
3318 1 0.1 0 4
3320 1 0.111111111111111 0 4
3321 1 0.1 0 4
3324 2 0.2 0 2
3380 1 0.1 0 4
3389 1 0.1 0 4
3392 1 0.1 0 4
3396 2 0.2 0 2
3397 1 0.1 0 4
3398 1 0.1 0 4
3399 2 0.333333333333333 0 2
3401 1 0.125 0 4
3402 2 0.2 0 2
3403 3 0.311111111111111 0 2
3404 4 0.4 0 2
3405 4 0.55 0 2
3406 3 0.3 0 2
3410 1 0.25 0 4
3463 1 0.1 0 4
3473 1 0.1 0 4
3482 1 0.1 0 4
3487 1 0.1 0 4
3488 3 0.325 0 2

---

category=4, cleavage_site=3132
query=pto-miR016a, target=Potri.004G170200.1,
score=0, range=3121-3141, strand=1

```
target  5' UCAUUCCAACACcCUCAACUUG 3'
           :::::::::::::::::::::
query   3' AGUAAGGUUGUGGAGUUGAAC 5'
```

>Potri.004G170200.1
#size=3744
17 1 0.1 0 4
174 1 0.1 0 4
183 1 0.1 0 4
481 1 0.1 0 4
488 1 0.111111111111111 0 4
529 1 0.1 0 4
547 1 0.2 0 4
643 1 0.1 0 4
652 1 0.1 0 4
662 1 0.1 0 4
679 1 0.1 0 4
766 1 0.1 0 4

794	1	0.111111111111111	0	4
813	1	0.1	0	4
824	1	0.1	0	4
827	1	0.1	0	4
926	1	0.1	0	4
932	1	0.111111111111111	0	4
937	1	0.1	0	4
942	1	0.1	0	4
947	1	0.1	0	4
955	1	0.1	0	4
957	1	0.1	0	4
959	1	0.1	0	4
964	1	0.1	0	4
1018	1	0.1	0	4
1054	1	0.1	0	4
1056	1	0.2	0	4
1058	2	0.2	0	2
1062	1	0.125	0	4
1064	2	0.25	0	2
1147	1	0.1	0	4
1159	1	0.1	0	4
1184	1	0.1	0	4
1213	1	0.1	0	4
1291	1	0.1	0	4
1527	1	0.1	0	4
1531	1	0.1	0	4
1533	1	0.1	0	4
1738	1	0.1	0	4
1745	1	0.1	0	4
1787	1	0.1	0	4
1818	1	0.1	0	4
1820	2	0.2	0	2
1831	1	0.1	0	4
1851	1	0.1	0	4
1865	1	0.2	0	4
1876	1	0.1	0	4
1881	1	0.333333333333333	0	4
1884	1	0.1	0	4
1893	1	0.1	0	4
1898	1	0.1	0	4
1900	1	0.1	0	4
1955	1	0.1	0	4
1959	1	0.1	0	4
1960	1	0.1	0	4
1968	1	0.1	0	4
2057	1	0.125	0	4
2069	2	0.225	0	2
2075	1	0.1	0	4
2122	1	0.1	0	4
2151	1	0.111111111111111	0	4
2162	1	0.1	0	4
2174	1	0.1	0	4
2175	1	0.111111111111111	0	4
2185	1	0.142857142857143	0	4
2197	1	0.142857142857143	0	4
2201	1	0.142857142857143	0	4
2240	2	0.2	0	2
2244	1	0.1	0	4

2306	1	0.142857142857143	0	4	
2308	1	0.1	0	4	
2310	1	0.125	0	4	
2322	1	0.1	0	4	
2474	1	0.1	0	4	
2492	2	0.2	0	2	
2496	1	0.1	0	4	
2558	1	0.142857142857143	0	4	
2560	1	0.1	0	4	
2562	1	0.125	0	4	
2574	1	0.1	0	4	
2665	1	0.1	0	4	
2717	1	0.125	0	4	
2719	2	0.25	0	2	
2723	2	0.2	0	2	
2728	1	0.5	0	4	
2729	1	0.25	0	4	
2732	1	0.333333333333333	0	4	
2748	2	2	2	0	
2932	1	0.1	0	4	
2952	1	0.25	0	4	
3001	2	0.2	0	2	
3004	1	0.125	0	4	
3088	1	0.125	0	4	
3106	1	0.1	0	4	
3132	1	0.1	0	4	<<<
3171	2	0.2	0	2	
3175	1	0.1	0	4	
3181	1	0.1	0	4	
3183	2	0.2	0	2	
3188	1	0.1	0	4	
3195	1	0.1	0	4	
3244	1	0.2	0	4	
3328	1	0.1	0	4	
3329	1	0.1	0	4	
3333	1	0.2	0	4	
3344	1	0.1	0	4	
3345	1	0.1	0	4	
3348	2	0.2	0	2	
3349	1	0.1	0	4	
3350	1	0.1	0	4	
3351	2	0.333333333333333	0	2	
3353	1	0.125	0	4	
3354	2	0.2	0	2	
3355	2	0.2	0	2	
3356	4	0.4	0	2	
3357	1	0.1	0	4	
3358	3	0.3	0	2	
3381	1	0.5	0	4	
3393	1	0.5	0	4	
3511	1	0.125	0	4	

category=4, cleavage_site=3174
query=pto-miR016a, target=Potri.004G170700.1,
score=0, range=3163-3183, strand=1

```
target  5' UCAUUCCAACAcCUCAACUUG 3'
           :::::::::::::::::::::
query   3' AGUAAGGUUGUGGAGUUGAAC 5'
```

>Potri.004G170700.1
#size=3534

47	1	0.1	0	4		
232	1	0.1	0	4		
237	1	0.1	0	4		
247	1	0.1	0	4		
251	1	0.1	0	4		
256	1	0.1	0	4		
273	1	0.1	0	4		
278	1	0.1	0	4		
562	1	0.2	0	4		
658	1	0.1	0	4		
667	1	0.1	0	4		
677	1	0.1	0	4		
678	2	0.2	0	2		
689	1	0.142857142857143			0	4
809	1	0.111111111111111			0	4
828	1	0.1	0	4		
839	1	0.1	0	4		
842	1	0.1	0	4		
918	1	0.125	0	4		
1124	1	0.1	0	4		
1140	1	0.2	0	4		
1142	1	0.1	0	4		
1146	1	0.125	0	4		
1148	2	0.25	0	2		
1187	1	0.1	0	4		
1205	2	0.333333333333333			0	2
1237	1	0.1	0	4		
1331	1	0.25	0	4		
1350	1	0.1	0	4		
1353	1	0.1	0	4		
1359	2	0.2	0	2		
1433	1	0.1	0	4		
1437	1	0.1	0	4		
1452	1	0.1	0	4		
1521	1	0.1	0	4		
1606	2	0.2	0	2		
1611	1	0.1	0	4		
1615	1	0.1	0	4		
1617	2	0.2	0	2		
1670	1	0.1	0	4		
1693	1	0.1	0	4		
1751	1	0.1	0	4		
1776	1	0.1	0	4		
1779	1	0.1	0	4		
1781	1	0.1	0	4		
1783	2	0.2	0	2		
1785	1	0.1	0	4		
1786	1	0.1	0	4		
1789	2	0.2	0	2		
1790	1	0.1	0	4		
1794	1	0.1	0	4		
1802	2	0.2	0	2		
1803	1	0.1	0	4		
1808	1	0.1	0	4		
1810	1	0.1	0	4		
1811	1	0.1	0	4		

1865	2	0.2	0	2	
1868	2	0.2	0	2	
1870	1	0.1	0	4	
1888	1	0.1	0	4	
1892	1	0.1	0	4	
1894	1	0.1	0	4	
1899	1	0.1	0	4	
1903	1	0.1	0	4	
1912	1	0.1	0	4	
1916	1	0.1	0	4	
1918	1	0.1	0	4	
1922	1	0.1	0	4	
1928	2	0.2	0	2	
1929	1	0.1	0	4	
1953	2	0.2	0	2	
1960	1	0.1	0	4	
1968	1	0.1	0	4	
1977	1	0.1	0	4	
1978	1	0.1	0	4	
2026	1	0.1	0	4	
2028	1	0.1	0	4	
2039	1	0.1	0	4	
2043	3	0.3	0	2	
2044	1	0.1	0	4	
2047	1	0.1	0	4	
2077	1	0.111111111111111	0	4	
2081	1	0.1	0	4	
2082	1	0.1	0	4	
2103	1	0.1	0	4	
2117	1	0.111111111111111	0	4	
2141	1	0.125	0	4	
2153	2	0.225	0	2	
2159	1	0.1	0	4	
2333	1	0.1	0	4	
2352	1	0.1	0	4	
2383	1	0.25	0	4	
2396	2	0.476190476190476	0	2	
2398	1	0.1	0	4	
2400	1	0.125	0	4	
2555	1	0.125	0	4	
2557	2	0.25	0	2	
2561	2	0.2	0	2	
2566	1	0.5	0	4	
2567	1	0.25	0	4	
2570	1	0.333333333333333	0	4	
2616	1	0.333333333333333	0	4	
2751	1	0.333333333333333	0	4	
2974	1	0.1	0	4	
2994	1	0.25	0	4	
3043	2	0.2	0	2	
3046	1	0.125	0	4	
3062	1	0.1	0	4	
3101	1	0.333333333333333	0	4	
3130	1	0.125	0	4	
3137	1	0.1	0	4	
3167	1	0.1	0	4	
3174	1	0.1	0	4	<<<
3184	1	0.25	0	4	

3185	1	0.25	0	4
3198	2	0.5	0	1
3212	1	0.1	0	4
3218	1	0.1	0	4
3286	1	0.2	0	4
3370	1	0.1	0	4
3371	1	0.1	0	4
3375	1	0.2	0	4
3386	1	0.1	0	4
3387	1	0.1	0	4
3390	2	0.2	0	2
3391	1	0.1	0	4
3392	1	0.1	0	4
3393	2	0.333333333333333	0	2
3395	1	0.125	0	4
3396	2	0.2	0	2
3397	2	0.2	0	2
3398	4	0.4	0	2
3399	1	0.1	0	4
3400	3	0.3	0	2
3423	1	0.5	0	4
3435	1	0.5	0	4

category=4, cleavage_site=3504
query=pto-miR016a, target=Potri.014G002200.1,
score=1, range=3493-3513, strand=1

```
target  5' UCAAUCCAACAcCUCAACUUG 3'
            ::: :::::::::::::::::
query   3' AGUAAGGUUGUGGAGUUGAAC 5'
```

>Potri.014G002200.1
#size=3894

146	1	0.142857142857143	0	4
163	1	0.166666666666667	0	4
183	1	0.142857142857143	0	4
273	1	0.1	0	4
329	1	0.1	0	4
353	1	0.1	0	4
407	1	0.1	0	4
410	2	0.2	0	2
415	1	0.1	0	4
422	2	0.2	0	2
426	1	0.125	0	4
427	1	0.166666666666667	0	4
432	1	0.111111111111111	0	4
434	1	0.1	0	4
440	1	0.166666666666667	0	4
443	1	0.125	0	4
558	1	0.1	0	4
589	1	0.1	0	4
591	1	0.1	0	4
686	3	0.3	0	2
697	3	0.3	0	2
700	1	0.1	0	4
764	1	0.1	0	4
767	1	0.1	0	4
768	1	0.142857142857143	0	4
799	2	0.222222222222222	0	2
807	1	0.1	0	4

815	2	0.2	0	2
822	1	0.1	0	4
842	1	0.1	0	4
843	1	0.1	0	4
857	1	0.1	0	4
858	1	0.1	0	4
868	1	0.1	0	4
897	1	0.2	0	4
1000	1	0.125	0	4
1014	1	0.25	0	4
1087	1	0.5	0	4
1088	1	0.5	0	4
1089	1	0.5	0	4
1114	1	0.1	0	4
1120	1	0.1	0	4
1188	1	0.166666666666667	0	4
1249	1	0.1	0	4
1261	3	0.342857142857143	0	2
1264	1	0.1	0	4
1411	1	0.1	0	4
1470	2	0.2	0	2
1607	1	0.1	0	4
1618	1	0.1	0	4
1646	1	1	1	4
1688	1	0.1	0	4
1730	1	0.1	0	4
1790	1	0.166666666666667	0	4
1791	2	0.333333333333333	0	2
1802	1	0.25	0	4
1812	1	0.1	0	4
1820	3	0.3	0	2
1821	1	0.1	0	4
1823	2	0.2	0	2
1826	1	0.1	0	4
1828	1	0.1	0	4
1836	1	0.1	0	4
1837	1	0.1	0	4
1862	1	0.1	0	4
1919	1	0.166666666666667	0	4
1933	1	0.1	0	4
2061	2	0.2	0	2
2065	1	0.1	0	4
2070	1	0.1	0	4
2200	1	0.1	0	4
2310	1	0.1	0	4
2327	1	0.1	0	4
2328	1	0.1	0	4
2384	1	0.1	0	4
2391	1	0.1	0	4
2431	1	0.1	0	4
2840	1	0.1	0	4
2898	1	0.1	0	4
2905	2	0.2	0	2
3045	1	0.125	0	4
3051	1	0.1	0	4
3052	1	0.125	0	4
3064	1	0.1	0	4
3133	1	0.5	0	4

3150	1	0.1	0	4	
3154	1	0.1	0	4	
3160	1	0.1	0	4	
3162	1	0.1	0	4	
3167	2	0.2	0	2	
3255	2	0.2	0	2	
3464	1	0.1	0	4	
3495	1	0.1	0	4	
3497	1	0.1	0	4	
3504	1	0.1	0	4	<<<
3516	2	0.5	0	2	
3558	1	0.5	0	4	
3606	1	0.1	0	4	
3623	1	0.111111111111111	0	4	
3671	1	0.166666666666667	0	4	
3673	1	0.166666666666667	0	4	
3679	1	0.1	0	4	
3680	1	0.1	0	4	
3683	1	0.1	0	4	
3692	1	0.1	0	4	
3695	1	0.1	0	4	
3696	1	0.1	0	4	
3699	2	0.2	0	2	
3700	1	0.1	0	4	
3701	1	0.1	0	4	
3705	1	0.1	0	4	
3776	1	0.1	0	4	
3785	1	0.1	0	4	
3789	1	0.1	0	4	
3790	1	0.1	0	4	
3791	3	0.325	0	2	
3802	1	0.5	0	4	

---

category=4, cleavage_site=2807
query=pto-miR016a, target=Potri.014G002300.1,
score=0, range=2796-2816, strand=1

```
target  5' UCAUUCCAACAcCUCAACUUG 3'
           :::::::::::::::::::::
query   3' AGUAAGGUUGUGGAGUUGAAC 5'
```

>Potri.014G002300.1
#size=3254

19	1	0.2	0	4
60	1	0.1	0	4
63	1	0.1	0	4
203	2	0.2	0	2
238	1	0.1	0	4
241	2	0.2	0	2
246	1	0.1	0	4
248	1	0.1	0	4
253	2	0.2	0	2
257	1	0.125	0	4
258	1	0.166666666666667	0	4
263	1	0.111111111111111	0	4
265	1	0.1	0	4
271	1	0.166666666666667	0	4
274	1	0.125	0	4
285	1	0.142857142857143	0	4
291	1	0.111111111111111	0	4

294	1	0.1	0	4		
356	1	0.1	0	4		
368	1	0.1	0	4		
422	1	0.1	0	4		
501	1	0.1	0	4		
515	1	0.1	0	4		
517	1	0.1	0	4		
522	1	0.1	0	4		
530	1	0.1	0	4		
543	1	0.1	0	4		
550	1	0.1	0	4		
551	1	0.1	0	4		
747	1	0.1	0	4		
752	1	0.1	0	4		
757	1	0.1	0	4		
765	2	0.433333333333333			0	2
767	1	0.1	0	4		
769	1	0.1	0	4		
774	1	0.1	0	4		
779	1	0.125	0	4		
783	1	0.166666666666667			0	4
788	1	0.1	0	4		
789	1	0.125	0	4		
810	1	0.25	0	4		
822	1	0.1	0	4		
846	1	0.1	0	4		
847	1	0.1	0	4		
850	1	0.1	0	4		
861	1	0.1	0	4		
864	1	0.1	0	4		
868	2	0.2	0	2		
914	1	1	1	4		
928	1	0.1	0	4		
929	1	0.1	0	4		
960	1	0.1	0	4		
975	1	0.1	0	4		
994	1	0.1	0	4		
1026	1	0.1	0	4		
1163	1	0.1	0	4		
1172	1	0.1	0	4		
1178	1	0.1	0	4		
1307	1	0.1	0	4		
1315	1	0.1	0	4		
1381	1	0.1	0	4		
1386	1	0.1	0	4		
1396	1	0.1	0	4		
1419	1	0.1	0	4		
1434	1	0.1	0	4		
1438	2	0.2	0	2		
1460	1	0.1	0	4		
1462	1	0.1	0	4		
1466	3	0.3	0	2		
1535	1	0.1	0	4		
1555	1	0.1	0	4		
1562	1	0.1	0	4		
1594	1	0.1	0	4		
1596	1	0.1	0	4		
1597	2	0.2	0	2		

1604	2	0.2	0	2	
1605	3	0.3	0	2	
1609	1	0.1	0	4	
1610	1	0.1	0	4	
1611	1	0.1	0	4	
1614	1	0.1	0	4	
1615	1	0.1	0	4	
1618	1	0.1	0	4	
1620	1	0.1	0	4	
1625	1	0.1	0	4	
1630	2	0.2	0	2	
1679	2	0.2	0	2	
1680	1	0.5	0	4	
1686	1	0.1	0	4	
1687	1	0.5	0	4	
1733	1	0.1	0	4	
1754	1	0.1	0	4	
1981	1	0.1	0	4	
2282	1	0.166666666666667	0	4	
2333	1	0.1	0	4	
2334	1	0.1	0	4	
2340	1	0.1	0	4	
2342	1	0.1	0	4	
2496	1	0.1	0	4	
2561	1	0.5	0	4	
2763	1	0.125	0	4	
2765	1	0.125	0	4	
2807	1	0.1	0	4	<<<
2856	1	0.1	0	4	
2858	2	0.2	0	2	
2863	2	0.2	0	2	
2870	1	0.1	0	4	
2930	1	0.1	0	4	
3020	1	0.1	0	4	
3030	3	0.311111111111111	0	2	
3031	4	0.4	0	2	
3032	4	0.55	0	2	
3033	2	0.2	0	2	
3037	1	0.25	0	4	

---

category=4, cleavage_site=3057
query=pto-miR016a, target=Potri.014G007900.1,
score=1, range=3046-3066, strand=1

```
target  5' UCAAUCCAACAcCUCAACUUG 3'
             ::: ::::::::::::::::
query   3' AGUAAGGUUGUGGAGUUGAAC 5'
```

>Potri.014G007900.1
#size=3502

17	1	0.1	0	4
45	1	0.142857142857143	0	4
202	1	0.1	0	4
207	1	0.1	0	4
217	1	0.1	0	4
299	1	0.1	0	4
323	1	0.1	0	4
523	1	0.1	0	4
645	1	0.1	0	4
656	6	0.6	0	2

657	1	0.1	0	4
668	2	0.242857142857143	0	2
670	1	0.1	0	4
673	1	0.1	0	4
684	1	0.1	0	4
687	1	0.166666666666667	0	4
690	1	0.166666666666667	0	4
725	1	0.125	0	4
766	2	0.285714285714286	0	2
785	2	0.2	0	2
827	1	0.1	0	4
828	1	0.1	0	4
838	1	0.1	0	4
849	1	0.1	0	4
854	1	0.1	0	4
887	1	0.1	0	4
1027	1	0.1	0	4
1029	1	0.1	0	4
1090	1	0.1	0	4
1109	1	0.1	0	4
1112	1	0.1	0	4
1123	1	0.1	0	4
1253	1	0.1	0	4
1256	1	0.1	0	4
1285	1	0.1	0	4
1306	1	0.1	0	4
1327	1	0.1	0	4
1341	1	0.1	0	4
1381	1	0.1	0	4
1396	1	0.1	0	4
1577	1	0.1	0	4
1588	1	0.1	0	4
1681	1	0.1	0	4
1701	1	0.1	0	4
1710	1	0.1	0	4
1769	1	0.1	0	4
1782	1	0.1	0	4
1790	3	0.3	0	2
1791	1	0.1	0	4
1793	2	0.2	0	2
1796	1	0.1	0	4
1856	1	0.1	0	4
1858	1	0.1	0	4
1859	2	0.2	0	2
1906	1	0.1	0	4
1916	2	0.2	0	2
1917	1	0.1	0	4
1923	1	0.1	0	4
1935	1	0.333333333333333	0	4
1941	2	0.2	0	2
1948	1	0.1	0	4
1953	1	0.333333333333333	0	4
1956	1	0.1	0	4
1965	1	0.1	0	4
1966	1	0.1	0	4
1970	1	0.1	0	4
1972	1	0.1	0	4
1973	1	0.1	0	4

1975	2	0.2	0	2		
1976	1	0.1	0	4		
1977	1	0.1	0	4		
1983	1	0.1	0	4		
1986	1	0.1	0	4		
1989	3	0.3	0	2		
1990	1	0.1	0	4		
1992	1	0.1	0	4		
1995	1	0.1	0	4		
1997	2	0.2	0	2		
1998	1	0.1	0	4		
2000	1	0.1	0	4		
2001	2	0.2	0	2		
2003	1	0.1	0	4		
2027	1	0.1	0	4		
2031	3	0.3	0	2		
2032	1	0.1	0	4		
2035	1	0.1	0	4		
2040	1	0.1	0	4		
2060	1	0.1	0	4		
2065	1	0.111111111111111			0	4
2069	1	0.1	0	4		
2070	1	0.1	0	4		
2071	1	0.142857142857143			0	4
2144	1	0.1	0	4		
2213	1	0.1	0	4		
2214	1	0.1	0	4		
2220	1	0.111111111111111			0	4
2231	1	0.1	0	4		
2243	1	0.1	0	4		
2244	1	0.111111111111111			0	4
2294	1	0.1	0	4		
2437	1	0.1	0	4		
2450	1	0.1	0	4		
2479	1	0.1	0	4		
2547	1	0.166666666666667			0	4
2741	1	0.125	0	4		
2743	1	0.2	0	4		
2997	2	0.2	0	2		
2998	1	0.125	0	4		
3015	1	0.125	0	4		
3017	1	0.1	0	4		
3020	1	0.1	0	4		
3048	1	0.1	0	4		
3050	1	0.1	0	4		
3057	1	0.1	0	4	<<<	
3069	2	0.5	0	2		
3095	1	0.1	0	4		
3101	1	0.1	0	4		
3169	1	0.2	0	4		
3172	1	0.142857142857143			0	4
3175	1	0.1	0	4		
3178	1	0.1	0	4		
3181	1	0.111111111111111			0	4
3184	2	0.2	0	2		
3195	1	0.1	0	4		
3198	1	0.1	0	4		
3280	2	0.2	0	2		

3281	4	0.4	0	2
3282	1	0.1	0	4
3283	3	0.3	0	2
3335	1	0.142857142857143	0	4
3339	3	0.676190476190476	0	2
3353	1	0.1	0	4
3355	1	0.1	0	4
3366	1	0.1	0	4
3368	1	0.1	0	4
3422	1	1	1	4

category=4, cleavage_site=3161
query=pto-miR016a, target=Potri.014G009300.1,
score=0, range=3150-3170, strand=1

```
target  5' UCAUUCCAACAcCUCAACUUG 3'
           :::::::::::::::::::::
query   3' AGUAAGGUUGUGGAGUUGAAC 5'
```

>Potri.014G009300.1
#size=3729

55	1	0.1	0	4
83	1	0.142857142857143	0	4
212	1	0.1	0	4
221	1	0.1	0	4
255	1	0.1	0	4
259	1	0.1	0	4
264	2	0.2	0	2
268	1	0.1	0	4
275	1	0.1	0	4
280	1	0.1	0	4
337	1	0.1	0	4
361	1	0.1	0	4
561	1	0.1	0	4
683	1	0.1	0	4
694	6	0.6	0	2
695	3	0.3	0	2
706	2	0.242857142857143	0	2
708	1	0.1	0	4
711	1	0.1	0	4
722	1	0.1	0	4
725	1	0.166666666666667	0	4
728	1	0.166666666666667	0	4
763	1	0.125	0	4
804	2	0.285714285714286	0	2
865	1	0.1	0	4
866	1	0.1	0	4
876	1	0.1	0	4
887	1	0.1	0	4
892	1	0.1	0	4
925	1	0.1	0	4
1008	1	0.125	0	4
1014	2	0.2	0	2
1023	1	0.1	0	4
1065	1	0.1	0	4
1067	1	0.1	0	4
1128	1	0.1	0	4
1164	1	0.1	0	4
1172	1	0.125	0	4
1174	2	0.25	0	2

1213	1	0.1	0	4
1226	1	0.1	0	4
1263	1	0.1	0	4
1344	1	0.1	0	4
1379	4	0.4	0	2
1401	1	0.1	0	4
1553	1	0.1	0	4
1559	1	0.1	0	4
1604	1	0.1	0	4
1623	1	0.1	0	4
1731	1	0.1	0	4
1736	1	0.1	0	4
1798	1	0.1	0	4
1821	2	0.2	0	2
1822	1	0.1	0	4
1825	4	0.4	0	2
1828	1	0.1	0	4
1832	2	0.2	0	2
1833	2	0.2	0	2
1836	1	0.1	0	4
1843	1	0.1	0	4
1845	1	0.1	0	4
1849	2	0.2	0	2
1852	1	0.1	0	4
1865	1	0.1	0	4
1867	1	0.1	0	4
1873	2	0.2	0	2
1874	1	0.1	0	4
1875	1	0.1	0	4
1876	1	0.1	0	4
1879	2	0.2	0	2
1882	1	0.1	0	4
1883	1	0.1	0	4
1886	2	0.2	0	2
1888	2	0.2	0	2
1890	1	0.1	0	4
1891	2	0.2	0	2
1893	2	0.2	0	2
1924	1	0.166666666666667	0	4
1938	1	0.1	0	4
1939	1	0.1	0	4
1952	1	0.1	0	4
1958	1	0.1	0	4
1970	1	0.333333333333333	0	4
1976	2	0.2	0	2
1983	1	0.1	0	4
1988	1	0.333333333333333	0	4
1991	1	0.1	0	4
2000	1	0.1	0	4
2001	1	0.1	0	4
2005	1	0.1	0	4
2007	1	0.1	0	4
2008	1	0.1	0	4
2010	2	0.2	0	2
2011	1	0.1	0	4
2012	1	0.1	0	4
2018	1	0.1	0	4
2021	1	0.1	0	4

2024	3	0.3	0	2		
2025	1	0.1	0	4		
2027	1	0.1	0	4		
2030	2	0.2	0	2		
2032	3	0.325	0	2		
2033	1	0.1	0	4		
2035	1	0.1	0	4		
2036	2	0.2	0	2		
2038	1	0.1	0	4		
2049	1	0.1	0	4		
2229	1	0.1	0	4		
2231	1	0.1	0	4		
2298	1	0.142857142857143			0	4
2310	1	0.142857142857143			0	4
2314	1	0.142857142857143			0	4
2335	1	0.1	0	4		
2392	1	0.1	0	4		
2406	1	0.25	0	4		
2421	1	0.1	0	4		
2423	1	0.125	0	4		
2537	1	0.1	0	4		
2612	1	0.1	0	4		
2613	1	0.1	0	4		
2619	1	0.1	0	4		
2621	1	0.1	0	4		
2769	1	1	1	4		
2890	1	0.1	0	4		
2948	1	0.1	0	4		
2955	2	0.2	0	2		
3101	2	0.2	0	2		
3102	1	0.125	0	4		
3114	1	0.1	0	4		
3117	1	0.125	0	4		
3161	1	0.1	0	4	<<<	
3224	1	0.1	0	4		
3279	1	0.1	0	4		
3282	1	0.1	0	4		
3285	1	0.111111111111111			0	4
3288	2	0.2	0	2		
3296	1	0.1	0	4		
3299	1	0.1	0	4		
3302	1	0.1	0	4		
3305	2	0.2	0	2		
3351	1	0.166666666666667			0	4
3370	1	0.1	0	4		
3373	1	0.1	0	4		
3377	2	0.2	0	2		
3378	1	0.1	0	4		
3379	1	0.1	0	4		
3380	2	0.333333333333333			0	2
3382	1	0.125	0	4		
3383	2	0.2	0	2		
3384	2	0.2	0	2		
3385	1	0.1	0	4		
3386	1	0.1	0	4		
3436	1	0.142857142857143			0	4
3440	3	0.676190476190476			0	2
3454	1	0.1	0	4		

3456	1	0.1	0	4
3467	1	0.1	0	4
3468	1	0.1	0	4
3469	1	0.1	0	4

---

category=4, cleavage_site=3171
query=pto-miR016a, target=Potri.014G009600.1,
score=0, range=3160-3180, strand=1

```
target  5' UCAUUCCAACAcCUCAACUUG 3'
           :::::::::::::::::::::
query   3' AGUAAGGUUGUGGAGUUGAAC 5'
```

>Potri.014G009600.1
#size=3658

81	1	0.333333333333333	0	4
198	1	0.1	0	4
266	1	0.1	0	4
272	1	0.1	0	4
652	1	0.5	0	4
772	2	0.285714285714286	0	2
775	2	0.222222222222222	0	2
969	1	1	1	4
1059	1	0.1	0	4
1060	1	0.125	0	4
1076	1	0.1	0	4
1079	1	0.1	0	4
1127	1	0.5	0	4
1149	1	1	1	4
1240	1	0.1	0	4
1243	1	0.1	0	4
1390	1	0.1	0	4
1405	1	0.1	0	4
1430	1	0.1	0	4
1434	1	0.1	0	4
1449	1	0.1	0	4
1491	1	0.1	0	4
1533	1	0.1	0	4
1608	1	0.1	0	4
1612	1	0.1	0	4
1614	1	0.1	0	4
1652	1	0.1	0	4
1659	1	0.1	0	4
1791	1	0.1	0	4
1795	1	0.1	0	4
1799	4	0.4	0	2
1802	2	0.2	0	2
1865	1	0.1	0	4
1867	1	0.1	0	4
1868	2	0.2	0	2
1871	1	0.1	0	4
1875	2	0.2	0	2
1876	2	0.2	0	2
1880	1	0.1	0	4
1881	1	0.1	0	4
1885	1	0.1	0	4
1886	1	0.1	0	4
1887	1	0.1	0	4
1891	1	0.1	0	4
1892	1	0.1	0	4

1895	1	0.1	0	4	
1896	1	0.1	0	4	
1898	1	0.1	0	4	
1901	2	0.2	0	2	
2025	1	0.1	0	4	
2052	1	0.1	0	4	
2150	1	0.1	0	4	
2153	1	0.25	0	4	
2205	1	0.1	0	4	
2292	1	0.1	0	4	
2310	1	0.1	0	4	
2330	2	0.2	0	2	
2349	1	0.1	0	4	
2541	1	0.1	0	4	
2711	1	1	1	4	
2745	1	0.333333333333333	0	4	
3162	1	0.1	0	4	
3164	1	0.1	0	4	
3171	1	0.1	0	4	<<<
3193	1	0.5	0	4	
3214	1	0.1	0	4	
3220	1	0.1	0	4	
3222	1	0.1	0	4	
3227	1	0.1	0	4	
3234	1	0.1	0	4	
3294	1	0.1	0	4	
3306	1	0.1	0	4	
3336	1	0.333333333333333	0	4	
3359	1	0.166666666666667	0	4	
3394	3	0.311111111111111	0	2	
3395	4	0.4	0	2	
3396	4	0.55	0	2	
3397	3	0.3	0	2	
3401	1	0.25	0	4	
3457	1	0.1	0	4	
3467	1	0.1	0	4	
3476	1	0.1	0	4	
3481	1	0.1	0	4	
3482	3	0.325	0	2	

category=4, cleavage_site=2997
query=pto-miR016a, target=Potri.014G010900.1,
score=1, range=2986-3006, strand=1

```
target  5' UCAAUCCAACAcCUCAACUUG 3'
              ::: :::::::::::::::::
query   3' AGUAAGGUUGUGGAGUUGAAC 5'
```

>Potri.014G010900.1
#size=3470

80	2	0.2	0	2
180	1	0.166666666666667	0	4
189	1	0.166666666666667	0	4
213	1	0.1	0	4
229	1	0.1	0	4
241	1	0.1	0	4
256	1	0.1	0	4
260	1	0.1	0	4
263	1	0.1	0	4
267	1	0.1	0	4

272	1	0.1	0	4
278	2	0.211111111111111	0	2
279	1	0.111111111111111	0	4
280	1	0.111111111111111	0	4
284	1	0.111111111111111	0	4
285	1	0.111111111111111	0	4
393	1	0.25	0	4
411	1	0.1	0	4
416	2	0.2	0	2
426	1	0.111111111111111	0	4
428	1	0.1	0	4
437	1	0.125	0	4
444	1	0.166666666666667	0	4
472	1	0.2	0	4
479	1	0.1	0	4
552	1	0.1	0	4
583	1	0.1	0	4
585	1	0.1	0	4
616	1	0.1	0	4
680	4	0.4	0	2
691	3	0.3	0	2
694	1	0.1	0	4
758	1	0.1	0	4
761	1	0.1	0	4
808	1	0.1	0	4
822	1	0.1	0	4
836	1	0.1	0	4
837	1	0.1	0	4
873	1	0.1	0	4
943	2	0.211111111111111	0	2
946	2	0.2	0	2
999	1	0.1	0	4
1062	1	0.2	0	4
1186	1	0.1	0	4
1187	1	0.1	0	4
1188	1	0.1	0	4
1190	1	0.1	0	4
1195	1	0.1	0	4
1199	1	0.1	0	4
1242	1	0.1	0	4
1245	1	0.1	0	4
1251	2	0.2	0	2
1410	1	0.1	0	4
1461	1	0.1	0	4
1469	1	0.1	0	4
1535	1	0.1	0	4
1540	1	0.1	0	4
1542	1	0.1	0	4
1592	2	0.2	0	2
1593	1	0.1	0	4
1602	1	0.1	0	4
1614	1	0.1	0	4
1640	1	0.1	0	4
1655	1	0.1	0	4
1677	1	0.1	0	4
1678	1	0.1	0	4
1682	3	0.3	0	2
1685	1	0.1	0	4

1688	1	0.1	0	4		
1689	1	0.1	0	4		
1804	1	0.1	0	4		
2141	1	0.1	0	4		
2144	1	0.1	0	4		
2276	1	0.1	0	4		
2373	1	0.1	0	4		
2387	2	0.2	0	2		
2393	1	0.25	0	4		
2457	1	0.1	0	4		
2540	1	0.166666666666667			0	4
2611	1	0.1	0	4		
2681	1	0.125	0	4		
2734	1	0.166666666666667			0	4
2784	1	0.1	0	4		
2885	1	0.1	0	4		
2913	1	0.5	0	4		
2931	1	0.125	0	4		
2937	1	0.1	0	4		
2957	1	0.1	0	4		
2988	1	0.1	0	4		
2990	1	0.1	0	4		
2997	1	0.1	0	4	<<<	
3007	1	0.25	0	4		
3008	1	0.25	0	4		
3009	2	0.5	0	2		
3021	2	0.5	0	2		
3036	2	0.2	0	2		
3040	1	0.1	0	4		
3046	1	0.1	0	4		
3048	2	0.2	0	2		
3053	2	0.2	0	2		
3072	1	0.5	0	4		
3115	1	0.1	0	4		
3190	1	0.333333333333333			0	4
3197	1	0.1	0	4		
3206	1	0.1	0	4		
3209	1	0.1	0	4		
3213	2	0.2	0	2		
3214	1	0.1	0	4		
3215	1	0.1	0	4		
3218	1	0.125	0	4		
3219	2	0.2	0	2		
3220	1	0.111111111111111			0	4
3222	2	0.2	0	2		
3271	1	1	1	4		
3275	1	0.142857142857143			0	4
3279	2	0.342857142857143			0	2
3293	1	0.1	0	4		
3302	1	0.1	0	4		
3306	1	0.1	0	4		
3307	1	0.1	0	4		
3308	3	0.325	0	2		

category=4, cleavage_site=2946
query=pto-miR016a, target=Potri.014G012000.1,
score=1, range=2935-2955, strand=1

```
target  5' UCAAUCCAACAcCUCAACUUG 3'
           ::: ::::::::::::::::
query   3' AGUAAGGUUGUGGAGUUGAAC 5'
```

>Potri.014G012000.1
#size=3416

12	1	0.1	0	4
62	1	0.1	0	4
64	1	0.1	0	4
229	1	0.1	0	4
234	1	0.1	0	4
248	1	0.1	0	4
252	1	0.1	0	4
260	1	0.1	0	4
270	1	0.1	0	4
274	1	0.25	0	4
305	1	0.2	0	4
502	1	0.1	0	4
509	1	0.111111111111111	0	4
664	1	0.1	0	4
670	1	0.125	0	4
672	1	0.1	0	4
673	1	0.1	0	4
680	1	0.2	0	4
683	12	1.2	0	0
684	3	0.3	0	2
697	1	0.1	0	4
700	1	0.1	0	4
711	1	0.1	0	4
732	1	0.166666666666667	0	4
735	1	0.25	0	4
787	1	0.1	0	4
815	1	0.111111111111111	0	4
834	1	0.1	0	4
845	1	0.1	0	4
848	1	0.1	0	4
924	1	0.125	0	4
938	1	0.166666666666667	0	4
991	2	0.211111111111111	0	2
994	2	0.2	0	2
1003	1	0.1	0	4
1016	1	0.1	0	4
1022	1	0.111111111111111	0	4
1027	1	0.1	0	4
1032	1	0.1	0	4
1037	1	0.1	0	4
1126	1	0.1	0	4
1127	1	0.1	0	4
1141	1	0.1	0	4
1144	1	0.1	0	4
1148	1	0.1	0	4
1205	1	0.1	0	4
1237	1	0.1	0	4
1249	3	0.342857142857143	0	2
1252	1	0.1	0	4
1300	1	0.1	0	4

1304	1	0.1	0	4
1305	1	0.1	0	4
1309	1	0.1	0	4
1313	1	0.1	0	4
1324	1	0.1	0	4
1359	4	0.4	0	2
1443	1	0.1	0	4
1537	1	0.2	0	4
1617	2	0.2	0	2
1621	1	0.1	0	4
1623	1	0.1	0	4
1661	2	0.2	0	2
1714	1	0.1	0	4
1742	1	0.1	0	4
1746	3	0.3	0	2
1766	2	0.225	0	2
1778	1	0.166666666666667	0	4
1779	2	0.333333333333333	0	2
1782	1	0.1	0	4
1790	1	0.25	0	4
1803	1	0.1	0	4
1804	1	0.1	0	4
1805	1	0.1	0	4
1808	2	0.2	0	2
1811	1	0.1	0	4
1814	2	0.2	0	2
1815	1	0.1	0	4
1816	2	0.2	0	2
1817	1	0.1	0	4
1819	1	0.1	0	4
1824	1	0.1	0	4
1825	1	0.1	0	4
1826	1	0.1	0	4
1828	1	0.1	0	4
1832	2	0.2	0	2
1850	1	0.1	0	4
1856	2	0.2	0	2
1857	1	0.1	0	4
1858	1	0.1	0	4
1859	2	0.2	0	2
1862	2	0.2	0	2
1865	1	0.1	0	4
1866	1	0.1	0	4
1869	2	0.2	0	2
1873	1	0.1	0	4
1874	1	0.1	0	4
1876	2	0.2	0	2
1907	1	0.166666666666667	0	4
1921	1	0.1	0	4
1928	1	0.1	0	4
1933	1	0.1	0	4
1934	2	0.2	0	2
1941	1	0.1	0	4
2061	1	0.1	0	4
2147	1	0.125	0	4
2159	2	0.225	0	2
2165	1	0.1	0	4
2212	1	0.1	0	4

2214	1	0.1	0	4	
2301	1	0.1	0	4	
2318	1	0.1	0	4	
2339	2	0.2	0	2	
2358	1	0.1	0	4	
2374	1	0.1	0	4	
2477	1	0.1	0	4	
2506	1	0.1	0	4	
2558	1	0.125	0	4	
2560	2	0.25	0	2	
2564	1	0.1	0	4	
2625	1	0.1	0	4	
2853	1	0.5	0	4	
2937	1	0.1	0	4	
2939	1	0.1	0	4	
2946	1	0.1	0	4	<<<
2956	1	0.25	0	4	
2957	1	0.25	0	4	
2958	2	0.5	0	2	
2970	2	0.5	0	2	
2985	2	0.2	0	2	
2989	1	0.1	0	4	
2997	2	0.2	0	2	
3002	2	0.2	0	2	
3058	1	0.2	0	4	
3061	1	0.142857142857143	0	4	
3064	1	0.1	0	4	
3067	1	0.1	0	4	
3070	1	0.111111111111111	0	4	
3073	2	0.2	0	2	
3084	1	0.1	0	4	
3087	1	0.1	0	4	
3170	3	0.3	0	2	
3172	3	0.3	0	2	
3224	1	0.142857142857143	0	4	
3228	3	0.676190476190476	0	2	
3268	1	0.5	0	4	

pto-miR017f

category=4, cleavage_site=1436
query=pto-miR017f, target=Potri.003G217900.2,
score=3, range=1425-1445, strand=1

```
target  5' UCUUAUUCCAUuUUUUUCCCU 3'
             :::::: :::::::: :::::
query   3' AGAAUACGGUAAAAACAGGGA 5'
```

>Potri.003G217900.2
#size=1638

134	1	0.166666666666667	0	4
137	5	0.833333333333333	0	2
144	1	0.166666666666667	0	4
146	1	0.166666666666667	0	4
147	1	0.166666666666667	0	4
148	3	0.5	0	2
154	1	0.2	0	4
557	1	0.111111111111111	0	4
559	5	0.777777777777778	0	2

632	1	0.1	0	4	
642	1	0.166666666666667	0	4	
708	1	0.166666666666667	0	4	
1270	1	0.2	0	4	
1293	2	0.333333333333333	0	3	
1299	1	0.166666666666667	0	4	
1346	1	0.2	0	4	
1362	1	0.2	0	4	
1366	1	0.2	0	4	
1393	2	0.5	0	2	
1394	1	0.25	0	4	
1402	2	0.5	0	2	
1407	1	0.25	0	4	
1413	1	0.25	0	4	
1416	1	0.25	0	4	
1419	2	0.5	0	2	
1432	1	0.25	0	4	
1433	2	0.5	0	2	
1434	1	0.25	0	4	
1436	1	0.25	0	4	<<<
1438	1	0.25	0	4	
1440	7	1.4	0	2	
1441	1	0.2	0	4	
1443	1	0.2	0	4	
1444	4	0.8	0	2	
1446	1	0.2	0	4	
1447	6	1.2	0	2	
1448	2	0.4	0	3	
1449	7	1.4	0	2	
1450	14	2.33333333333333	0	2	
1451	1	0.166666666666667	0	4	
1453	3	0.5	0	2	
1454	5	0.833333333333333	0	2	
1456	6	1.16666666666667	0	2	
1457	3	0.5	0	2	
1458	11	1.83333333333333	0	2	
1459	6	1.03333333333333	0	2	
1460	2	0.333333333333333	0	3	
1461	9	1.5	0	2	
1462	2	0.333333333333333	0	3	
1463	3	0.5	0	2	
1465	12	2	0	2	
1466	5	0.833333333333333	0	2	
1467	2	0.333333333333333	0	3	
1468	12	2	0	2	
1470	1	0.166666666666667	0	4	
1471	16	2.66666666666667	0	2	
1472	11	1.83333333333333	0	2	
1473	12	2	0	2	
1474	2	0.333333333333333	0	3	
1477	3	0.5	0	2	
1478	5	0.833333333333333	0	2	
1479	1	0.166666666666667	0	4	
1480	3	0.5	0	2	
1481	4	0.666666666666667	0	2	
1482	6	1	0	2	
1483	5	0.833333333333333	0	2	
1484	2	0.333333333333333	0	3	

1486	3	0.5	0	2
1488	3	0.5	0	2
1489	3	0.5	0	2
1490	3	0.5	0	2
1491	1	0.166666666666667	0	4
1492	8	1.33333333333333	0	2
1493	1	0.166666666666667	0	4
1494	8	1.33333333333333	0	2
1495	6	1	0	2
1497	4	0.666666666666667	0	2
1498	4	0.666666666666667	0	2
1500	1	0.2	0	4
1501	1	0.2	0	4
1503	10	2	0	2
1504	4	0.8	0	2
1505	3	0.6	0	2
1506	3	0.6	0	2
1507	1	0.2	0	4
1508	2	0.4	0	3
1509	1	0.2	0	4
1510	1	0.2	0	4
1514	1	0.2	0	4
1516	5	1	0	2
1517	4	0.8	0	2
1518	6	1.2	0	2
1519	11	2.2	0	2
1520	24	4.8	0	2
1521	17	3.4	0	2
1522	5	1	0	2
1523	3	0.6	0	2
1524	2	0.4	0	3
1525	1	0.2	0	4
1527	1	0.2	0	4
1528	1	0.2	0	4
1529	2	0.4	0	3
1530	2	0.4	0	3
1531	4	1	0	2
1532	4	1	0	2
1534	4	1	0	2
1535	5	1.25	0	2
1540	2	0.5	0	2
1606	6	6	6	0

pto-miR018a

category=2, cleavage_site=493
query=pto-miR018a, target=Potri.001G077600.1,
score=2, range=483-502, strand=1

```
target  5' CGAGGGACAAaAACGCAUAA 3'
           :::::::::::: ::::::
query   3' GCUCCCUGUUUUUACGUAUU 5'
```

>Potri.001G077600.1
#size=1001

282	1	0.25	0	4
283	2	0.5	0	2
284	1	0.25	0	4
296	1	0.25	0	4

297	1	0.25	0	4
314	1	0.25	0	4
315	1	0.25	0	4
316	1	0.25	0	4
318	1	0.25	0	4
321	2	0.5	0	2
322	1	0.25	0	4
324	2	0.5	0	2
327	1	0.25	0	4
328	1	0.25	0	4
331	1	0.25	0	4
332	1	0.25	0	4
335	1	0.25	0	4
342	2	0.5	0	2
344	1	0.25	0	4
346	1	0.25	0	4
394	1	1	1	4
408	1	0.333333333333333	0	4
409	2	0.666666666666667	0	2
410	2	0.5	0	2
411	4	1	0	2
412	1	0.25	0	4
413	1	0.25	0	4
415	1	0.25	0	4
418	2	0.5	0	2
420	2	0.5	0	2
421	1	0.25	0	4
422	1	0.25	0	4
423	2	0.5	0	2
424	1	0.25	0	4
426	4	1	0	2
428	2	0.5	0	2
429	4	1	0	2
430	1	0.25	0	4
431	2	0.5	0	2
432	1	0.25	0	4
433	1	0.25	0	4
434	2	0.5	0	2
435	3	0.75	0	2
436	2	0.5	0	2
437	2	0.5	0	2
438	3	0.75	0	2
439	2	0.5	0	2
440	2	0.5	0	2
441	1	0.25	0	4
442	2	0.5	0	2
445	1	0.25	0	4
448	1	0.25	0	4
449	2	0.5	0	2
450	4	1	0	2
451	1	0.25	0	4
452	2	0.5	0	2
455	4	1	0	2
456	2	0.5	0	2
462	1	0.142857142857143	0	4
463	2	0.285714285714286	0	3
464	1	0.2	0	4
465	1	0.2	0	4

466	5	0.85	0	2		
468	1	0.2	0	4		
470	3	0.375	0	3		
471	4	0.5	0	2		
472	1	0.142857142857143			0	4
473	2	0.4	0	3		
474	1	0.125	0	4		
475	1	0.125	0	4		
479	2	0.285714285714286			0	3
481	1	0.25	0	4		
484	2	0.5	0	2		
485	2	0.5	0	2		
487	1	0.25	0	4		
488	2	0.5	0	2		
490	6	1.5	0	2		
492	5	1.25	0	2		
493	10	2.5	0	2	<<<	
494	12	3	0	0		
495	3	0.75	0	2		
496	1	0.25	0	4		
497	7	1.75	0	2		
500	2	0.5	0	2		
504	3	0.75	0	2		
505	1	0.25	0	4		
508	3	0.75	0	2		
509	2	0.5	0	2		
510	2	0.5	0	2		
511	2	0.5	0	2		
512	1	0.25	0	4		
513	2	0.5	0	2		
514	1	0.25	0	4		
516	2	0.5	0	2		
517	6	1.5	0	2		
518	3	0.75	0	2		
519	3	0.75	0	2		
520	2	0.5	0	2		
522	2	0.5	0	2		
523	1	0.25	0	4		
526	2	0.45	0	2		
527	2	0.5	0	2		
530	1	0.25	0	4		
531	3	0.75	0	2		
532	1	0.25	0	4		
533	2	0.5	0	2		
534	3	0.75	0	2		
536	2	0.5	0	2		
540	4	1	0	2		
541	4	1	0	2		
542	2	0.5	0	2		
543	4	1	0	2		
545	3	0.75	0	2		
546	2	0.5	0	2		
547	1	0.25	0	4		
549	3	0.75	0	2		
550	1	0.25	0	4		
551	3	0.75	0	2		
552	2	0.5	0	2		
553	2	0.5	0	2		

554	1	0.25	0	4
556	2	0.5	0	2
557	2	0.5	0	2
558	1	0.25	0	4
559	2	0.5	0	2
560	1	0.25	0	4
561	1	0.25	0	4
562	6	1.5	0	2
563	4	1	0	2
564	3	0.75	0	2
566	3	0.75	0	2
567	1	0.25	0	4
568	2	0.5	0	2
571	2	0.5	0	2
573	3	0.75	0	2
574	2	0.5	0	2
575	2	0.5	0	2
576	2	0.5	0	2
578	1	0.25	0	4
579	2	0.5	0	2
582	4	1	0	2
583	1	0.25	0	4
584	1	0.25	0	4
585	1	0.25	0	4
587	5	1.25	0	2
589	4	1	0	2
590	1	0.25	0	4
592	4	1	0	2
595	1	0.25	0	4
597	1	0.25	0	4
598	1	0.25	0	4
600	1	0.25	0	4
601	1	0.25	0	4
603	2	0.5	0	2
607	1	0.25	0	4
608	1	0.25	0	4
609	2	0.5	0	2
610	1	0.25	0	4
612	4	1	0	2
621	1	0.25	0	4
637	1	0.142857142857143	0	4
638	2	0.285714285714286	0	3
639	3	0.428571428571429	0	3
640	2	0.285714285714286	0	3
641	1	0.25	0	4
642	2	0.5	0	2
643	2	0.45	0	2
644	3	0.75	0	2
645	1	0.25	0	4
649	3	0.75	0	2
650	3	0.75	0	2
651	1	0.25	0	4
652	2	0.5	0	2
653	2	0.5	0	2
654	1	0.25	0	4
655	1	0.25	0	4
656	1	0.25	0	4
659	1	0.25	0	4

666	2	0.666666666666667	0	2
667	1	0.333333333333333	0	4
670	1	0.333333333333333	0	4
671	1	0.333333333333333	0	4
672	1	0.333333333333333	0	4
674	1	0.333333333333333	0	4
675	1	0.333333333333333	0	4
678	1	0.333333333333333	0	4
682	1	0.333333333333333	0	4
686	1	0.333333333333333	0	4
687	1	0.333333333333333	0	4
689	3	1	0	2
690	5	1.66666666666667	0	2
691	3	1	0	2
692	1	0.333333333333333	0	4
693	1	0.333333333333333	0	4
694	1	0.333333333333333	0	4
698	1	0.25	0	4
700	1	0.25	0	4
701	2	0.5	0	2
702	6	1.5	0	2
703	1	0.25	0	4
705	1	0.25	0	4

---

category=2, cleavage_site=307
query=pto-miR018a, target=Potri.001G078100.1,
score=3, range=297-316, strand=1

```
target  5' CGAGGGUCAAaAACGCAUAA 3'
           :::::: :::::: ::::::
query   3' GCUCCCUGUUUUUACGUAUU 5'
```

>Potri.001G078100.1
#size=789

120	1	1	1	4
121	1	1	1	4
123	2	2	2	3
125	1	1	1	4
127	2	2	2	3
129	1	0.5	0	4
130	1	0.5	0	4
134	1	0.5	0	4
138	1	0.5	0	4
140	1	0.5	0	4
141	1	0.5	0	4
143	2	1	0	3
144	1	0.5	0	4
145	2	1	0	3
146	5	2.5	0	3
147	3	1.5	0	3
148	2	1	0	3
149	2	1	0	3
150	1	0.5	0	4
151	1	0.5	0	4
152	6	3	0	3
153	10	5	0	2
154	3	1.5	0	3
158	2	1	0	3
160	1	0.5	0	4
244	2	1	0	3

245	3	1.5	0	3	
247	1	0.5	0	4	
249	4	2	0	3	
250	1	0.5	0	4	
253	1	0.5	0	4	
254	1	0.5	0	4	
255	7	3.5	0	3	
256	5	2.5	0	3	
257	5	2.25	0	3	
258	7	3.5	0	3	
259	5	2.5	0	3	
260	7	2.83333333333333	0	3	
261	18	7.33333333333333	0	2	
262	13	5.83333333333333	0	2	
263	18	8.66666666666667	0	2	
264	14	6.33333333333333	0	2	
265	10	5	0	2	
266	6	3	0	3	
267	9	5	1	2	
268	8	4	0	3	
269	9	4.5	0	3	
270	17	6.2	0	2	
271	12	4.66666666666667	0	3	
272	14	6.33333333333333	0	2	
273	9	3.83333333333333	0	3	
274	6	1.2	0	3	
275	3	0.9	0	3	
276	8	2.2	0	3	
277	14	3.1	0	3	
278	6	1.5	0	3	
279	11	2.5	0	3	
280	9	1.8	0	3	
281	10	2	0	3	
282	7	1.4	0	3	
283	11	2.5	0	3	
284	5	0.833333333333333	0	3	
285	7	1.11111111111111	0	3	
286	11	5.5	0	2	
287	19	8.47777777777778	0	2	
288	4	2	0	3	
289	7	3.5	0	3	
290	5	2.5	0	3	
291	4	2	0	3	
292	7	3.5	0	3	
293	13	6.5	0	2	
294	3	1.5	0	3	
296	7	4	1	3	
297	8	4	0	3	
298	14	7	0	2	
299	2	1	0	3	
300	4	2	0	3	
301	1	0.5	0	4	
302	12	6	0	2	
303	2	1	0	3	
304	11	5.5	0	2	
305	4	2	0	3	
306	6	3	0	3	
307	28	14	0	2	<<<

308	12	6	0	2		
309	9	9	9	2		
310	21	20.5	20	2		
311	17	17	17	2		
312	11	11	11	2		
313	5	5	5	2		
314	10	10	10	2		
315	6	6	6	2		
316	11	11	11	2		
317	7	7	7	2		
318	6	6	6	2		
319	8	8	8	2		
320	6	6	6	2		
321	10	10	10	2		
322	7	7	7	2		
323	10	10	10	2		
324	5	5	5	2		
325	10	10	10	2		
326	6	6	6	2		
327	6	6	6	2		
328	31	31	31	2		
329	2	2	2	3		
330	6	6	6	2		
331	18	18	18	2		
332	9	9	9	2		
333	7	7	7	2		
334	6	6	6	2		
335	6	6	6	2		
336	5	5	5	2		
337	2	2	2	3		
338	3	3	3	3		
339	3	3	3	3		
340	6	5.2	5	2		
341	4	2	0	3		
342	1	1	1	4		
343	8	8	8	2		
344	6	5.5	5	2		
345	6	3	0	3		
346	12	6.5	1	2		
347	6	3	0	3		
348	13	6.5	0	2		
349	15	7.5	0	2		
350	15	7.5	0	2		
351	21	10.5	0	2		
352	13	6.5	0	2		
353	12	6	0	2		
354	14	7	0	2		
355	6	3	0	3		
356	12	6	0	2		
357	18	9	0	2		
358	20	10	0	2		
359	23	11.5	0	2		
360	10	5	0	2		
361	34	16.2777777777778			0	2
362	23	11.1111111111111			0	2
363	5	2.11111111111111			0	3
364	19	10	1	2		
365	14	7	0	2		

366	13	13	13	2
367	9	9	9	2
368	11	10.5	10	2
369	13	13	13	2
370	77	77	77	0
371	7	7	7	2
372	8	8	8	2
373	7	7	7	2
374	19	19	19	2
375	11	11	11	2
376	11	11	11	2
377	10	10	10	2
378	16	8	0	2
379	19	9.5	0	2
380	8	4	0	3
381	13	6.5	0	2
382	42	21.5	1	2
383	29	15	1	2
384	16	9	2	2
385	14	7	0	2
386	11	5.5	0	2
387	6	3	0	3
388	12	6	0	2
389	1	0.5	0	4
390	2	1	0	3
391	15	7.5	0	2
392	4	2	0	3
393	4	1.33333333333333	0	3
394	9	3	0	3
395	6	2	0	3
396	2	0.666666666666667	0	3
397	2	0.5	0	3
398	5	1.5	0	3
399	4	1	0	3
400	3	0.833333333333333	0	3
401	2	0.25	0	3
402	4	0.5	0	3
403	4	0.5	0	3
404	6	0.75	0	3
405	5	0.625	0	3
406	7	0.875	0	3
407	5	0.6	0	3
408	2	0.25	0	3
409	4	0.5	0	3
410	3	0.375	0	3
411	2	0.25	0	3
412	4	1	0	3
413	1	0.25	0	4
414	1	0.25	0	4
415	4	1	0	3
416	2	0.5	0	3
417	3	0.75	0	3
418	4	1.33333333333333	0	3
419	3	1	0	3
420	4	1.33333333333333	0	3
421	10	10	10	2
422	17	17	17	2
423	5	4.5	4	3

424	4	4	4	3
425	10	10	10	2
427	3	3	3	3
428	9	9	9	2
429	4	4	4	3
430	4	4	4	3
431	8	8	8	2
432	12	12	12	2
433	8	8	8	2
434	7	7	7	2
435	8	8	8	2
436	13	13	13	2
437	10	10	10	2
438	12	12	12	2
439	9	9	9	2
440	5	5	5	2
441	15	15	15	2
442	13	13	13	2
443	14	14	14	2
444	4	4	4	3
445	5	5	5	2
446	2	2	2	3
447	7	7	7	2
448	3	3	3	3
449	11	11	11	2
450	16	16	16	2
451	12	12	12	2
452	7	7	7	2
453	5	5	5	2
454	11	11	11	2
455	11	11	11	2
456	5	5	5	2
457	11	10.2	10	2
458	10	10	10	2
459	9	9	9	2
460	7	7	7	2
461	8	8	8	2
462	5	5	5	2
463	13	13	13	2
464	10	10	10	2
465	13	13	13	2
466	46	46	46	2
467	9	9	9	2
468	20	20	20	2
469	9	9	9	2
470	20	20	20	2
471	11	11	11	2
472	46	46	46	2
473	11	11	11	2
474	11	11	11	2
475	14	14	14	2
476	8	8	8	2
477	8	8	8	2
478	10	10	10	2
479	19	19	19	2
480	9	9	9	2
481	7	7	7	2
482	9	9	9	2

483	2	2	2	3
484	7	7	7	2
485	2	2	2	3
486	2	2	2	3
487	15	15	15	2
488	3	3	3	3
489	4	4	4	3
490	7	7	7	2
491	10	10	10	2
492	11	11	11	2
493	17	17	17	2
494	8	8	8	2
495	12	12	12	2
496	14	14	14	2
497	12	12	12	2
498	13	13	13	2
499	12	12	12	2
500	10	10	10	2
501	16	16	16	2
502	14	14	14	2
503	29	29	29	2
504	23	23	23	2
505	1	1	1	4
609	3	3	3	3
610	3	3	3	3
611	2	2	2	3
612	3	3	3	3
613	2	2	2	3
614	1	1	1	4
615	1	1	1	4
616	4	4	4	3
617	2	2	2	3
618	1	1	1	4
620	2	2	2	3
621	1	1	1	4
622	1	1	1	4
624	1	1	1	4
625	1	1	1	4
626	3	3	3	3
627	2	2	2	3
628	1	1	1	4
664	2	2	2	3
672	1	1	1	4
675	1	1	1	4
681	1	1	1	4
683	1	1	1	4
689	1	1	1	4

---

category=2, cleavage_site=706
query=pto-miR018a, target=Potri.001G422300.1,
score=2, range=696-715, strand=1

```
target  5' CGAGGGACAAaAACGCAUAA 3'
           :::::::::::: ::::::
query   3' GCUCCCUGUUUUUACGUAUU 5'
```

>Potri.001G422300.1
#size=906

642	1	1	1	4
643	1	1	1	4

644	2	2	2	2	
646	1	0.333333333333333	0	4	
647	3	1	0	2	
648	1	0.333333333333333	0	4	
649	2	0.666666666666667	0	2	
650	2	2	2	2	
651	1	1	1	4	
653	2	2	2	2	
654	3	3	3	2	
655	1	1	1	4	
656	3	3	3	2	
657	1	1	1	4	
658	3	3	3	2	
659	2	2	2	2	
660	5	5	5	2	
661	8	8	8	2	
662	4	4	4	2	
663	9	9	9	0	
664	2	2	2	2	
665	7	7	7	2	
666	2	2	2	2	
668	3	3	3	2	
669	1	1	1	4	
670	1	1	1	4	
671	1	1	1	4	
672	3	3	3	2	
673	1	1	1	4	
674	5	5	5	2	
675	5	5	5	2	
676	3	3	3	2	
677	1	0.2	0	4	
678	1	0.2	0	4	
679	5	0.85	0	2	
681	1	0.2	0	4	
683	3	0.375	0	3	
684	4	0.5	0	3	
686	2	0.4	0	3	
687	1	0.125	0	4	
688	1	0.125	0	4	
690	1	1	1	4	
693	2	2	2	2	
694	1	1	1	4	
703	2	0.666666666666667	0	2	
705	1	0.333333333333333	0	4	
706	2	0.666666666666667	0	2	<<<
707	4	2	1	2	
709	3	1	0	2	
710	2	0.666666666666667	0	2	
711	1	0.333333333333333	0	4	
712	1	0.333333333333333	0	4	
716	3	1.4	1	2	
717	2	0.4	0	3	
719	1	0.2	0	4	
720	2	0.4	0	3	
723	1	0.2	0	4	
725	1	0.2	0	4	
726	2	0.4	0	3	
727	1	0.2	0	4	

728	2	0.4	0	3
729	2	0.4	0	3
730	1	0.2	0	4
734	1	0.333333333333333	0	4
735	2	0.533333333333333	0	3
736	1	0.333333333333333	0	4
737	1	0.333333333333333	0	4
738	1	0.333333333333333	0	4
739	3	1	0	2
740	1	0.333333333333333	0	4
742	2	0.666666666666667	0	2
745	1	0.333333333333333	0	4
746	2	0.666666666666667	0	2
750	3	1	0	2
751	1	0.333333333333333	0	4
752	5	1.66666666666667	0	2
753	3	1	0	2
754	1	0.333333333333333	0	4
763	1	0.333333333333333	0	4
764	1	1	1	4
766	1	1	1	4
768	2	2	2	2
771	1	0.333333333333333	0	4
774	1	0.333333333333333	0	4
803	1	0.333333333333333	0	4
806	1	0.333333333333333	0	4
817	1	0.333333333333333	0	4
819	1	0.333333333333333	0	4
822	2	0.4	0	3
835	1	0.333333333333333	0	4
839	1	0.333333333333333	0	4
850	1	1	1	4
851	2	0.666666666666667	0	2
854	2	0.666666666666667	0	2
855	1	0.333333333333333	0	4
860	1	0.333333333333333	0	4
864	3	1	0	2
865	1	0.333333333333333	0	4
866	1	0.333333333333333	0	4
867	3	1	0	2
869	1	0.333333333333333	0	4

---

category=4, cleavage_site=192
query=pto-miR018a, target=Potri.003G152000.1,
score=2, range=182-201, strand=1

```
target  5' CGAGGGACAAaAACGCAUAA 3'
           ::::::::::::: ::::::
query   3' GCUCCCUGUUUUUACGUAUU 5'
```

>Potri.003G152000.1
#size=398

117	1	0.333333333333333	0	4
136	1	0.333333333333333	0	4
139	2	1.33333333333333	1	2
140	1	0.333333333333333	0	4
142	2	0.5	0	2
152	1	0.25	0	4
155	1	0.2	0	4
159	6	1.2	0	2

160	2	0.4	0	2	
161	7	1.34285714285714	0	2	
162	15	2.88571428571429	0	0	
163	5	1	0	2	
164	10	2	0	2	
165	11	2.05	0	2	
166	10	2	0	2	
167	7	1.4	0	2	
169	3	0.375	0	2	
170	5	0.611111111111111	0	2	
171	1	0.142857142857143	0	4	
172	2	0.311111111111111	0	3	
173	1	0.125	0	4	
174	1	0.125	0	4	
178	2	0.285714285714286	0	3	
192	1	0.333333333333333	0	4	<<<
193	2	0.666666666666667	0	2	
194	1	0.333333333333333	0	4	
198	1	0.333333333333333	0	4	
207	1	0.333333333333333	0	4	
273	1	0.25	0	4	
295	1	0.25	0	4	
319	1	0.25	0	4	
322	1	0.333333333333333	0	4	
336	1	0.142857142857143	0	4	
337	2	0.285714285714286	0	3	
338	3	0.428571428571429	0	2	
339	2	0.285714285714286	0	3	
342	1	0.333333333333333	0	4	
344	1	0.333333333333333	0	4	
345	1	0.333333333333333	0	4	
352	1	0.333333333333333	0	4	

---

category=4, cleavage_site=323
query=pto-miR018a, target=Potri.003G152300.1,
score=2, range=313-332, strand=1

```
target  5' CGAGGGACAAaAACGCAUAA 3'
           :::::::::::: ::::::
query   3' GCUCCCUGUUUUUACGUAUU 5'
```

>Potri.003G152300.1
#size=668

144	1	1	1	4
147	1	1	1	4
150	1	1	1	4
152	1	1	1	4
164	1	0.5	0	4
167	1	0.5	0	4
170	1	0.5	0	4
174	2	1	0	2
183	2	1	0	2
212	1	0.5	0	4
248	1	0.333333333333333	0	4
267	1	0.333333333333333	0	4
270	1	0.333333333333333	0	4
273	1	0.25	0	4
283	1	0.25	0	4
286	1	0.2	0	4
290	6	1.2	0	2

291	2	0.4	0	2	
292	7	1.34285714285714	0	2	
293	15	2.88571428571429	0	0	
294	5	1	0	2	
295	10	2	0	2	
296	11	2.05	0	2	
297	10	2	0	2	
298	7	1.4	0	2	
300	3	0.375	0	2	
301	5	0.611111111111111	0	2	
302	1	0.142857142857143	0	4	
303	2	0.311111111111111	0	3	
304	1	0.125	0	4	
305	1	0.125	0	4	
309	2	0.285714285714286	0	3	
323	1	0.333333333333333	0	4	<<<
324	2	0.666666666666667	0	2	
325	1	0.333333333333333	0	4	
329	1	0.333333333333333	0	4	
338	1	0.333333333333333	0	4	
378	1	0.111111111111111	0	4	
379	1	0.111111111111111	0	4	
404	1	0.25	0	4	
426	1	0.25	0	4	
450	1	0.25	0	4	
453	1	0.333333333333333	0	4	
467	1	0.142857142857143	0	4	
468	2	0.285714285714286	0	3	
469	3	0.428571428571429	0	2	
470	2	0.285714285714286	0	3	
473	1	0.333333333333333	0	4	
475	1	0.333333333333333	0	4	
476	1	0.333333333333333	0	4	
483	1	0.333333333333333	0	4	
560	1	0.111111111111111	0	4	
561	1	0.111111111111111	0	4	
586	1	0.25	0	4	
608	1	0.25	0	4	
632	1	0.25	0	4	

---

category=2, cleavage_site=250
query=pto-miR018a, target=Potri.015G135100.1,
score=4, range=240-259, strand=1

```
target  5' CGAGGGUCAAaAACGCGUAA 3'
             :::::: :::::: ::.:::
query   3' GCUCCCUGUUUUUACGUAUU 5'
```

>Potri.015G135100.1
#size=471

72	1	0.5	0	4
73	1	0.5	0	4
77	1	0.5	0	4
78	1	1	1	4
79	4	4	4	2
80	3	3	3	2
81	4	3.5	3	2
82	2	2	2	3
83	1	0.5	0	4
84	6	5.5	5	2

85	2	2	2	3
86	12	11	10	2
87	3	2.5	2	3
88	2	1	0	3
89	5	2.5	0	3
90	3	1.5	0	3
91	2	1	0	3
92	2	1	0	3
93	1	0.5	0	4
94	1	0.5	0	4
95	6	3	0	2
96	11	6	1	2
97	3	1.5	0	3
101	2	1	0	3
103	1	0.5	0	4
187	2	1	0	3
188	3	1.5	0	3
190	5	4.5	4	2
191	5	5	5	2
192	9	7	5	2
193	5	4.5	4	2
194	8	8	8	2
195	6	6	6	2
196	4	3.5	3	2
197	5	4.5	4	2
198	7	3.5	0	2
199	5	2.5	0	3
200	7	2.75	0	3
201	8	4.5	1	2
202	6	2.83333333333333	0	2
203	8	3.83333333333333	1	2
204	20	8	0	2
205	13	5.83333333333333	0	2
206	18	8.66666666666667	0	2
207	14	6.33333333333333	0	2
208	10	5	0	2
209	7	4	1	2
210	9	4.25	0	2
211	8	4	0	2
212	12	7.5	3	2
213	17	6.2	0	2
214	12	4.66666666666667	0	2
215	15	7.33333333333333	1	2
216	9	3.83333333333333	0	2
217	6	1.2	0	3
218	3	0.9	0	3
219	8	2.2	0	3
220	14	3.1	0	2
221	6	1.5	0	3
222	11	2.5	0	3
223	9	1.8	0	3
224	10	2	0	3
225	7	1.4	0	3
226	11	2.5	0	3
227	5	0.833333333333333	0	3
228	7	1.11111111111111	0	3
229	11	5.5	0	2
230	19	8.47777777777778	0	2

231	4	2	0	3		
232	7	3.5	0	2		
233	5	2.5	0	3		
234	4	2	0	3		
235	7	3.5	0	2		
236	13	6.5	0	2		
237	3	1.5	0	3		
239	6	3	0	2		
240	8	4	0	2		
241	14	7	0	2		
242	2	1	0	3		
243	4	2	0	3		
244	1	0.5	0	4		
245	12	6	0	2		
246	2	1	0	3		
247	11	5.5	0	2		
248	4	2	0	3		
249	6	3	0	2		
250	28	14	0	2	<<<	
251	12	6	0	2		
253	1	0.5	0	4		
284	4	2	0	3		
287	1	0.5	0	4		
288	6	3	0	2		
289	11	5.5	0	2		
290	6	3	0	2		
291	13	6.5	0	2		
292	15	7.5	0	2		
293	15	7.5	0	2		
294	21	10.5	0	2		
295	13	6.5	0	2		
296	12	6	0	2		
297	14	7	0	2		
298	6	3	0	2		
299	12	6	0	2		
300	18	9	0	2		
301	20	10	0	2		
302	23	11.5	0	2		
303	10	5	0	2		
304	34	16.2777777777778			0	2
305	23	11.1111111111111			0	2
306	5	2.11111111111111			0	3
307	18	9	0	2		
308	14	7	0	2		
311	1	0.5	0	4		
321	16	8	0	2		
322	19	9.5	0	2		
323	8	4	0	2		
324	13	6.5	0	2		
325	41	20.5	0	0		
326	28	14	0	2		
327	14	7	0	2		
328	14	7	0	2		
329	11	5.5	0	2		
330	6	3	0	2		
331	12	6	0	2		
332	1	0.5	0	4		
333	2	1	0	3		

334	15	7.5	0	2
335	4	2	0	3
336	4	1.33333333333333	0	3
337	9	3	0	2
338	6	2	0	3
339	2	0.666666666666667	0	3
340	2	0.5	0	3
341	4	1	0	3
342	4	1	0	3
343	3	0.833333333333333	0	3
344	2	0.25	0	3
345	4	0.5	0	3
346	4	0.5	0	3
347	6	0.75	0	3
348	5	0.625	0	3
349	7	0.875	0	3
350	5	0.6	0	3
351	2	0.25	0	3
352	4	0.5	0	3
353	3	0.375	0	3
354	2	0.25	0	3
355	4	1	0	3
356	1	0.25	0	4
357	1	0.25	0	4
358	4	1	0	3
359	2	0.5	0	3
360	3	0.75	0	3
361	4	1.33333333333333	0	3
362	3	1	0	3
363	4	1.33333333333333	0	3
366	1	0.5	0	4

pto-miR018b

category=0, cleavage_site=494
query=pto-miR018b, target=Potri.001G077600.1,
score=3, range=483-504, strand=1

```
target  5' CGAGGGACAAAaACGCAUAAGA 3'
             : ::::::::::::::: :::
query   3' GGUCCCUGUUUUUGCGUA-UCU 5'
```

>Potri.001G077600.1
#size=1001

282	1	0.25	0	4
283	2	0.5	0	2
284	1	0.25	0	4
296	1	0.25	0	4
297	1	0.25	0	4
314	1	0.25	0	4
315	1	0.25	0	4
316	1	0.25	0	4
318	1	0.25	0	4
321	2	0.5	0	2
322	1	0.25	0	4
324	2	0.5	0	2
327	1	0.25	0	4
328	1	0.25	0	4
331	1	0.25	0	4

332	1	0.25	0	4
335	1	0.25	0	4
342	2	0.5	0	2
344	1	0.25	0	4
346	1	0.25	0	4
394	1	1	1	4
408	1	0.333333333333333	0	4
409	2	0.666666666666667	0	2
410	2	0.5	0	2
411	4	1	0	2
412	1	0.25	0	4
413	1	0.25	0	4
415	1	0.25	0	4
418	2	0.5	0	2
420	2	0.5	0	2
421	1	0.25	0	4
422	1	0.25	0	4
423	2	0.5	0	2
424	1	0.25	0	4
426	4	1	0	2
428	2	0.5	0	2
429	4	1	0	2
430	1	0.25	0	4
431	2	0.5	0	2
432	1	0.25	0	4
433	1	0.25	0	4
434	2	0.5	0	2
435	3	0.75	0	2
436	2	0.5	0	2
437	2	0.5	0	2
438	3	0.75	0	2
439	2	0.5	0	2
440	2	0.5	0	2
441	1	0.25	0	4
442	2	0.5	0	2
445	1	0.25	0	4
448	1	0.25	0	4
449	2	0.5	0	2
450	4	1	0	2
451	1	0.25	0	4
452	2	0.5	0	2
455	4	1	0	2
456	2	0.5	0	2
462	1	0.142857142857143	0	4
463	2	0.285714285714286	0	3
464	1	0.2	0	4
465	1	0.2	0	4
466	5	0.85	0	2
468	1	0.2	0	4
470	3	0.375	0	3
471	4	0.5	0	2
472	1	0.142857142857143	0	4
473	2	0.4	0	3
474	1	0.125	0	4
475	1	0.125	0	4
479	2	0.285714285714286	0	3
481	1	0.25	0	4
484	2	0.5	0	2

485	2	0.5	0	2	
487	1	0.25	0	4	
488	2	0.5	0	2	
490	6	1.5	0	2	
492	5	1.25	0	2	
493	10	2.5	0	2	
494	12	3	0	0	<<<
495	3	0.75	0	2	
496	1	0.25	0	4	
497	7	1.75	0	2	
500	2	0.5	0	2	
504	3	0.75	0	2	
505	1	0.25	0	4	
508	3	0.75	0	2	
509	2	0.5	0	2	
510	2	0.5	0	2	
511	2	0.5	0	2	
512	1	0.25	0	4	
513	2	0.5	0	2	
514	1	0.25	0	4	
516	2	0.5	0	2	
517	6	1.5	0	2	
518	3	0.75	0	2	
519	3	0.75	0	2	
520	2	0.5	0	2	
522	2	0.5	0	2	
523	1	0.25	0	4	
526	2	0.45	0	2	
527	2	0.5	0	2	
530	1	0.25	0	4	
531	3	0.75	0	2	
532	1	0.25	0	4	
533	2	0.5	0	2	
534	3	0.75	0	2	
536	2	0.5	0	2	
540	4	1	0	2	
541	4	1	0	2	
542	2	0.5	0	2	
543	4	1	0	2	
545	3	0.75	0	2	
546	2	0.5	0	2	
547	1	0.25	0	4	
549	3	0.75	0	2	
550	1	0.25	0	4	
551	3	0.75	0	2	
552	2	0.5	0	2	
553	2	0.5	0	2	
554	1	0.25	0	4	
556	2	0.5	0	2	
557	2	0.5	0	2	
558	1	0.25	0	4	
559	2	0.5	0	2	
560	1	0.25	0	4	
561	1	0.25	0	4	
562	6	1.5	0	2	
563	4	1	0	2	
564	3	0.75	0	2	
566	3	0.75	0	2	

567	1	0.25	0	4
568	2	0.5	0	2
571	2	0.5	0	2
573	3	0.75	0	2
574	2	0.5	0	2
575	2	0.5	0	2
576	2	0.5	0	2
578	1	0.25	0	4
579	2	0.5	0	2
582	4	1	0	2
583	1	0.25	0	4
584	1	0.25	0	4
585	1	0.25	0	4
587	5	1.25	0	2
589	4	1	0	2
590	1	0.25	0	4
592	4	1	0	2
595	1	0.25	0	4
597	1	0.25	0	4
598	1	0.25	0	4
600	1	0.25	0	4
601	1	0.25	0	4
603	2	0.5	0	2
607	1	0.25	0	4
608	1	0.25	0	4
609	2	0.5	0	2
610	1	0.25	0	4
612	4	1	0	2
621	1	0.25	0	4
637	1	0.142857142857143	0	4
638	2	0.285714285714286	0	3
639	3	0.428571428571429	0	3
640	2	0.285714285714286	0	3
641	1	0.25	0	4
642	2	0.5	0	2
643	2	0.45	0	2
644	3	0.75	0	2
645	1	0.25	0	4
649	3	0.75	0	2
650	3	0.75	0	2
651	1	0.25	0	4
652	2	0.5	0	2
653	2	0.5	0	2
654	1	0.25	0	4
655	1	0.25	0	4
656	1	0.25	0	4
659	1	0.25	0	4
666	2	0.666666666666667	0	2
667	1	0.333333333333333	0	4
670	1	0.333333333333333	0	4
671	1	0.333333333333333	0	4
672	1	0.333333333333333	0	4
674	1	0.333333333333333	0	4
675	1	0.333333333333333	0	4
678	1	0.333333333333333	0	4
682	1	0.333333333333333	0	4
686	1	0.333333333333333	0	4
687	1	0.333333333333333	0	4

689	3	1	0	2
690	5	1.66666666666667	0	2
691	3	1	0	2
692	1	0.333333333333333	0	4
693	1	0.333333333333333	0	4
694	1	0.333333333333333	0	4
698	1	0.25	0	4
700	1	0.25	0	4
701	2	0.5	0	2
702	6	1.5	0	2
703	1	0.25	0	4
705	1	0.25	0	4

category=2, cleavage_site=233
query=pto-miR018b, target=Potri.001G422000.1,
score=4, range=222-243, strand=1

```
target  5' UGAGGGGCAAAaACGCAUCAGA 3'
           . ::::.::::::::::: :::
query   3' GGUCCCUGUUUUUGCGUA-UCU 5'
```

>Potri.001G422000.1
#size=549

13	1	0.5	0	4
42	2	1	0	2
43	1	0.5	0	4
47	1	0.5	0	4
49	1	0.5	0	4
51	1	0.5	0	4
56	1	0.5	0	4
62	1	0.5	0	4
63	2	1	0	2
64	3	1.5	0	2
65	1	0.5	0	4
66	1	0.5	0	4
86	1	0.5	0	4
88	1	0.5	0	4
90	2	1	0	2
91	1	0.5	0	4
93	1	0.5	0	4
95	1	0.5	0	4
99	1	0.5	0	4
100	1	0.5	0	4
101	1	0.5	0	4
106	2	1	0	2
107	1	0.5	0	4
108	1	0.5	0	4
113	1	0.5	0	4
116	1	0.5	0	4
118	1	0.5	0	4
121	2	1	0	2
122	1	0.5	0	4
140	1	0.5	0	4
161	1	0.5	0	4
164	1	0.5	0	4
172	1	0.333333333333333	0	4
173	3	1	0	2
174	1	0.333333333333333	0	4
175	2	0.666666666666667	0	2
178	1	0.5	0	4

179	2	1	0	2	
182	1	0.5	0	4	
183	2	1	0	2	
184	1	0.5	0	4	
185	2	1	0	2	
186	5	2.5	0	0	
187	1	0.5	0	4	
192	3	1.5	0	2	
193	2	1	0	2	
194	3	1.5	0	2	
196	3	1.5	0	2	
203	1	0.5	0	4	
204	2	1	0	2	
210	1	0.5	0	4	
229	2	0.666666666666667	0	2	
231	1	0.333333333333333	0	4	
232	2	0.666666666666667	0	2	
233	3	1	0	2	<<<
235	3	1	0	2	
236	2	0.666666666666667	0	2	
237	1	0.333333333333333	0	4	
238	1	0.333333333333333	0	4	
242	2	0.4	0	3	
243	2	0.4	0	3	
245	1	0.2	0	4	
246	2	0.4	0	3	
249	1	0.2	0	4	
251	1	0.2	0	4	
252	2	0.4	0	3	
253	1	0.2	0	4	
254	2	0.4	0	3	
255	2	0.4	0	3	
256	1	0.2	0	4	
260	1	0.333333333333333	0	4	
261	2	0.533333333333333	0	2	
262	1	0.333333333333333	0	4	
263	1	0.333333333333333	0	4	
264	1	0.333333333333333	0	4	
265	3	1	0	2	
266	1	0.333333333333333	0	4	
268	2	0.666666666666667	0	2	
271	1	0.333333333333333	0	4	
272	2	0.666666666666667	0	2	
276	3	1	0	2	
277	1	0.333333333333333	0	4	
278	5	1.66666666666667	0	2	
279	3	1	0	2	
280	1	0.333333333333333	0	4	
289	1	0.333333333333333	0	4	
297	1	0.333333333333333	0	4	
323	1	0.5	0	4	
329	1	0.333333333333333	0	4	
332	1	0.333333333333333	0	4	
337	1	0.5	0	4	
341	1	0.5	0	4	
343	1	0.333333333333333	0	4	
345	1	0.333333333333333	0	4	
348	2	0.4	0	3	

361	1	0.333333333333333	0	4
365	1	0.333333333333333	0	4
377	2	0.666666666666667	0	2
380	2	0.666666666666667	0	2
381	1	0.333333333333333	0	4
386	1	0.333333333333333	0	4
390	3	1	0	2
391	1	0.333333333333333	0	4
392	1	0.333333333333333	0	4
393	3	1	0	2
395	1	0.333333333333333	0	4
401	3	1.5	0	2

---

category=2, cleavage_site=707
query=pto-miR018b, target=Potri.001G422300.1,
score=3, range=696-717, strand=1

```
target  5' CGAGGGACAAAaACGCAUAAGA 3'
             : ::::::::::::::: :::
query   3' GGUCCCUGUUUUUGCGUA-UCU 5'
```

>Potri.001G422300.1
#size=906

642	1	1	1	4
643	1	1	1	4
644	2	2	2	2
646	1	0.333333333333333	0	4
647	3	1	0	2
648	1	0.333333333333333	0	4
649	2	0.666666666666667	0	2
650	2	2	2	2
651	1	1	1	4
653	2	2	2	2
654	3	3	3	2
655	1	1	1	4
656	3	3	3	2
657	1	1	1	4
658	3	3	3	2
659	2	2	2	2
660	5	5	5	2
661	8	8	8	2
662	4	4	4	2
663	9	9	9	0
664	2	2	2	2
665	7	7	7	2
666	2	2	2	2
668	3	3	3	2
669	1	1	1	4
670	1	1	1	4
671	1	1	1	4
672	3	3	3	2
673	1	1	1	4
674	5	5	5	2
675	5	5	5	2
676	3	3	3	2
677	1	0.2	0	4
678	1	0.2	0	4
679	5	0.85	0	2
681	1	0.2	0	4
683	3	0.375	0	3

684	4	0.5	0	3	
686	2	0.4	0	3	
687	1	0.125	0	4	
688	1	0.125	0	4	
690	1	1	1	4	
693	2	2	2	2	
694	1	1	1	4	
703	2	0.666666666666667	0	2	
705	1	0.333333333333333	0	4	
706	2	0.666666666666667	0	2	
707	4	2	1	2	<<<
709	3	1	0	2	
710	2	0.666666666666667	0	2	
711	1	0.333333333333333	0	4	
712	1	0.333333333333333	0	4	
716	3	1.4	1	2	
717	2	0.4	0	3	
719	1	0.2	0	4	
720	2	0.4	0	3	
723	1	0.2	0	4	
725	1	0.2	0	4	
726	2	0.4	0	3	
727	1	0.2	0	4	
728	2	0.4	0	3	
729	2	0.4	0	3	
730	1	0.2	0	4	
734	1	0.333333333333333	0	4	
735	2	0.533333333333333	0	3	
736	1	0.333333333333333	0	4	
737	1	0.333333333333333	0	4	
738	1	0.333333333333333	0	4	
739	3	1	0	2	
740	1	0.333333333333333	0	4	
742	2	0.666666666666667	0	2	
745	1	0.333333333333333	0	4	
746	2	0.666666666666667	0	2	
750	3	1	0	2	
751	1	0.333333333333333	0	4	
752	5	1.66666666666667	0	2	
753	3	1	0	2	
754	1	0.333333333333333	0	4	
763	1	0.333333333333333	0	4	
764	1	1	1	4	
766	1	1	1	4	
768	2	2	2	2	
771	1	0.333333333333333	0	4	
774	1	0.333333333333333	0	4	
803	1	0.333333333333333	0	4	
806	1	0.333333333333333	0	4	
817	1	0.333333333333333	0	4	
819	1	0.333333333333333	0	4	
822	2	0.4	0	3	
835	1	0.333333333333333	0	4	
839	1	0.333333333333333	0	4	
850	1	1	1	4	
851	2	0.666666666666667	0	2	
854	2	0.666666666666667	0	2	
855	1	0.333333333333333	0	4	

860	1	0.333333333333333	0	4	
864	3	1	0	2	
865	1	0.333333333333333	0	4	
866	1	0.333333333333333	0	4	
867	3	1	0	2	
869	1	0.333333333333333	0	4	

---

category=2, cleavage_site=193
query=pto-miR018b, target=Potri.003G152000.1,
score=3, range=182-203, strand=1

```
target  5' CGAGGGACAAAaACGCAUAAGA 3'
           : :::::::::::::::: :::
query   3' GGUCCCUGUUUUUGCGUA-UCU 5'
```

>Potri.003G152000.1
#size=398

117	1	0.333333333333333	0	4	
136	1	0.333333333333333	0	4	
139	2	1.33333333333333	1	2	
140	1	0.333333333333333	0	4	
142	2	0.5	0	2	
152	1	0.25	0	4	
155	1	0.2	0	4	
159	6	1.2	0	2	
160	2	0.4	0	2	
161	7	1.34285714285714	0	2	
162	15	2.88571428571429	0	0	
163	5	1	0	2	
164	10	2	0	2	
165	11	2.05	0	2	
166	10	2	0	2	
167	7	1.4	0	2	
169	3	0.375	0	2	
170	5	0.611111111111111	0	2	
171	1	0.142857142857143	0	4	
172	2	0.311111111111111	0	3	
173	1	0.125	0	4	
174	1	0.125	0	4	
178	2	0.285714285714286	0	3	
192	1	0.333333333333333	0	4	
193	2	0.666666666666667	0	2	<<<
194	1	0.333333333333333	0	4	
198	1	0.333333333333333	0	4	
207	1	0.333333333333333	0	4	
273	1	0.25	0	4	
295	1	0.25	0	4	
319	1	0.25	0	4	
322	1	0.333333333333333	0	4	
336	1	0.142857142857143	0	4	
337	2	0.285714285714286	0	3	
338	3	0.428571428571429	0	2	
339	2	0.285714285714286	0	3	
342	1	0.333333333333333	0	4	
344	1	0.333333333333333	0	4	
345	1	0.333333333333333	0	4	
352	1	0.333333333333333	0	4	

category=2, cleavage_site=324
query=pto-miR018b, target=Potri.003G152300.1,
score=3, range=313-334, strand=1

```
target  5' CGAGGGACAAAaACGCAUAAGA 3'
            : :::::::::::::::: :::
query   3' GGUCCCUGUUUUUGCGUA-UCU 5'
```

>Potri.003G152300.1
#size=668

144	1	1	1	4	
147	1	1	1	4	
150	1	1	1	4	
152	1	1	1	4	
164	1	0.5	0	4	
167	1	0.5	0	4	
170	1	0.5	0	4	
174	2	1	0	2	
183	2	1	0	2	
212	1	0.5	0	4	
248	1	0.333333333333333	0	4	
267	1	0.333333333333333	0	4	
270	1	0.333333333333333	0	4	
273	1	0.25	0	4	
283	1	0.25	0	4	
286	1	0.2	0	4	
290	6	1.2	0	2	
291	2	0.4	0	2	
292	7	1.34285714285714	0	2	
293	15	2.88571428571429	0	0	
294	5	1	0	2	
295	10	2	0	2	
296	11	2.05	0	2	
297	10	2	0	2	
298	7	1.4	0	2	
300	3	0.375	0	2	
301	5	0.611111111111111	0	2	
302	1	0.142857142857143	0	4	
303	2	0.311111111111111	0	3	
304	1	0.125	0	4	
305	1	0.125	0	4	
309	2	0.285714285714286	0	3	
323	1	0.333333333333333	0	4	
324	2	0.666666666666667	0	2	<<<
325	1	0.333333333333333	0	4	
329	1	0.333333333333333	0	4	
338	1	0.333333333333333	0	4	
378	1	0.111111111111111	0	4	
379	1	0.111111111111111	0	4	
404	1	0.25	0	4	
426	1	0.25	0	4	
450	1	0.25	0	4	
453	1	0.333333333333333	0	4	
467	1	0.142857142857143	0	4	
468	2	0.285714285714286	0	3	
469	3	0.428571428571429	0	2	
470	2	0.285714285714286	0	3	
473	1	0.333333333333333	0	4	
475	1	0.333333333333333	0	4	

476	1	0.333333333333333	0	4
483	1	0.333333333333333	0	4
560	1	0.111111111111111	0	4
561	1	0.111111111111111	0	4
586	1	0.25	0	4
608	1	0.25	0	4
632	1	0.25	0	4

pto-miR030a,b

category=2, cleavage_site=276
query=pto-miR030a,b, target=Potri.008G221200.1,
score=4, range=265-285, strand=1

```
target  5' GGUGUUGAUGAcCAAACUGUC 3'
            .:. ::::::::::::: :::
query   3' UCGGAACUACUGGUUUGCCAG 5'
```

>Potri.008G221200.1
#size=871

18	1	0.5	0	4
19	1	1	1	4
22	3	2	1	2
31	1	0.5	0	4
32	1	0.5	0	4
43	1	0.5	0	4
44	1	0.5	0	4
45	2	1	0	2
47	1	0.5	0	4
49	4	2.5	1	2
50	1	0.5	0	4
51	1	0.5	0	4
52	1	0.5	0	4
55	1	0.5	0	4
56	1	0.5	0	4
57	1	0.5	0	4
64	9	4.5	0	2
65	2	1	0	2
67	2	1	0	2
68	2	1	0	2
69	1	0.5	0	4
70	1	0.5	0	4
71	1	0.5	0	4
72	3	1.5	0	2
73	1	0.5	0	4
78	1	0.25	0	4
79	2	1	0	2
80	3	0.75	0	3
81	6	1.5	0	2
82	7	1.75	0	2
83	7	1.75	0	2
84	7	1.75	0	2
85	4	1	0	2
86	6	1.5	0	2
87	7	1.16666666666667	0	2
88	9	1.58333333333333	0	2
89	5	0.833333333333333	0	3
90	5	0.833333333333333	0	3
91	10	1.75	0	2

93	5	0.833333333333333	0	3
94	4	0.666666666666667	0	3
95	5	1	0	3
96	4	0.666666666666667	0	3
97	4	0.666666666666667	0	3
98	1	0.166666666666667	0	4
99	2	0.333333333333333	0	3
100	5	1.25	0	2
101	7	1.75	0	2
102	25	6.25	0	2
103	11	2.75	0	2
104	4	1	0	2
105	12	3	0	2
106	2	0.5	0	3
107	2	0.5	0	3
108	3	0.75	0	3
109	4	1	0	2
110	1	0.25	0	4
111	6	1.5	0	2
112	2	0.5	0	3
113	2	0.5	0	3
114	2	0.5	0	3
115	10	2.5	0	2
116	4	1	0	3
117	8	2.08333333333333	0	2
118	19	3.16666666666667	0	2
119	8	2	0	2
120	6	1.08333333333333	0	2
123	1	0.5	0	4
124	1	0.5	0	4
126	1	0.5	0	4
127	1	0.5	0	4
128	1	0.5	0	4
129	1	0.5	0	4
130	1	0.5	0	4
132	2	1	0	2
137	1	0.5	0	4
139	4	1	0	2
140	2	0.5	0	3
141	2	0.5	0	3
142	5	1.25	0	2
143	4	1	0	2
144	5	1.25	0	2
145	8	2	0	2
146	3	0.75	0	3
147	3	0.75	0	3
148	6	1.5	0	2
149	4	1	0	2
150	6	1.5	0	2
151	10	2.5	0	2
152	8	2	0	2
153	3	0.75	0	3
154	1	0.25	0	4
155	5	1.25	0	2
156	8	2	0	2
157	8	2	0	2
158	23	5.75	0	2
159	6	1.5	0	2

160	1	0.25	0	4
161	4	1	0	2
162	8	2	0	2
163	3	0.75	0	3
164	6	1.5	0	2
165	3	0.75	0	3
166	1	0.25	0	4
167	2	0.5	0	3
168	3	0.75	0	3
170	6	1.5	0	2
171	14	3.5	0	2
172	13	2.16666666666667	0	2
173	4	1	0	2
174	4	1	0	2
175	8	2	0	2
176	6	1.5	0	2
177	5	1.25	0	2
179	3	0.75	0	3
180	6	1.5	0	2
181	6	3	0	2
182	6	3	0	2
183	7	3.5	0	2
184	5	2.5	0	2
185	2	1	0	2
186	13	6.5	0	2
187	6	2.75	0	2
188	10	5	0	2
189	2	1	0	2
190	1	0.5	0	4
191	4	2	0	2
192	2	1	0	2
193	1	0.5	0	4
195	3	1.5	0	2
196	2	1	0	2
197	2	1	0	2
198	2	1	0	2
199	1	0.5	0	4
200	2	1	0	2
201	2	1	0	2
203	5	2.5	0	2
204	3	1.5	0	2
205	3	1.5	0	2
206	4	2	0	2
207	3	1.5	0	2
208	1	0.5	0	4
209	1	0.5	0	4
210	1	0.5	0	4
213	1	0.5	0	4
214	1	0.5	0	4
215	1	0.5	0	4
216	1	0.5	0	4
217	3	1.5	0	2
218	1	0.5	0	4
220	1	0.5	0	4
221	1	0.5	0	4
222	2	1	0	2
223	2	1	0	2
224	1	0.5	0	4

227	2	1	0	2	
228	1	0.5	0	4	
231	1	0.5	0	4	
232	1	0.25	0	4	
233	1	0.25	0	4	
234	2	0.5	0	3	
235	1	0.25	0	4	
236	1	0.25	0	4	
239	1	0.5	0	4	
240	2	0.75	0	3	
241	1	0.25	0	4	
242	2	0.5	0	3	
243	1	0.25	0	4	
244	1	0.25	0	4	
248	2	0.5	0	3	
250	3	1.5	0	2	
251	2	1	0	2	
253	2	0.5	0	3	
254	2	0.75	0	3	
255	2	0.75	0	3	
256	2	1	0	2	
257	3	1.5	0	2	
258	2	1	0	2	
259	4	2	0	2	
260	3	1.5	0	2	
261	8	4	0	2	
262	7	3.25	0	2	
263	2	1	0	2	
264	1	0.5	0	4	
265	2	1	0	2	
266	3	1.5	0	2	
268	3	1.5	0	2	
269	1	0.5	0	4	
270	1	0.5	0	4	
271	3	1.5	0	2	
272	3	1.5	0	2	
274	3	1.5	0	2	
276	2	1	0	2	<<<
277	3	1.5	0	2	
278	1	0.5	0	4	
279	1	0.5	0	4	
280	1	0.5	0	4	
281	7	3.5	0	2	
282	3	1.5	0	2	
283	4	2	0	2	
285	6	3	0	2	
287	1	0.5	0	4	
288	1	0.5	0	4	
290	2	1	0	2	
291	1	0.5	0	4	
292	3	0.916666666666667	0	3	
293	1	0.333333333333333	0	4	
294	4	1.33333333333333	0	2	
295	6	2	0	2	
296	2	1	0	2	
297	2	1	0	2	
298	2	1	0	2	
299	2	0.833333333333333	0	3	

300	2	1	0	2
301	9	2.2	0	2
302	1	0.25	0	4
303	4	1.25	0	2
304	1	0.2	0	4
305	3	0.75	0	3
306	3	0.75	0	3
307	5	1.25	0	2
308	4	1	0	2
309	5	1.25	0	2
310	8	1.6	0	2
311	12	2.4	0	2
312	5	1	0	2
313	6	2	0	2
314	9	3	0	2
315	14	4	0	2
316	14	4.05	0	2
317	3	0.75	0	3
318	2	0.5	0	3
319	4	2	0	2
320	2	1	0	2
321	2	1	0	2
322	1	0.5	0	4
324	1	0.5	0	4
325	1	0.5	0	4
328	1	0.5	0	4
331	1	0.5	0	4
332	2	1	0	2
333	2	1	0	2
334	1	0.5	0	4
336	2	1	0	2
337	4	2	0	2
338	9	4.5	0	2
339	3	1.5	0	2
340	7	3.5	0	2
341	9	4.5	0	2
342	6	3	0	2
343	4	2	0	2
344	5	2.5	0	2
345	1	0.5	0	4
346	3	1.5	0	2
347	1	0.5	0	4
348	3	1.5	0	2
349	1	0.5	0	4
351	1	0.5	0	4
352	1	0.5	0	4
355	2	1	0	2
356	3	1.5	0	2
357	7	3.5	0	2
358	8	4	0	2
359	3	1.5	0	2
360	3	1.5	0	2
361	9	4.5	0	2
362	3	1.5	0	2
363	6	3	0	2
364	4	2	0	2
365	9	4.5	0	2
366	5	2.5	0	2

367	14	7	0	2
368	19	9.5	0	0
369	4	2	0	2
370	5	2.5	0	2
371	4	2	0	2
373	1	0.5	0	4
374	1	0.5	0	4
375	2	1	0	2
376	5	2.5	0	2
378	4	2	0	2
379	8	4	0	2
380	3	1.5	0	2
381	1	0.5	0	4
382	2	1	0	2
384	2	1	0	2
385	3	1.5	0	2
386	1	0.5	0	4
387	2	1	0	2
388	1	0.5	0	4
389	2	1	0	2
390	2	1	0	2
391	2	1	0	2
392	3	1.5	0	2
393	1	0.5	0	4
394	2	1	0	2
397	1	0.5	0	4
398	1	0.5	0	4
399	1	0.5	0	4
400	5	2.5	0	2
401	3	1.5	0	2
402	1	0.5	0	4
403	2	1	0	2
404	1	0.5	0	4
405	3	1.5	0	2
406	1	0.5	0	4
407	1	0.5	0	4
408	3	1.5	0	2
409	2	1	0	2
410	2	1	0	2
412	1	0.5	0	4
413	2	1	0	2
416	3	1.5	0	2
417	1	0.5	0	4
418	2	1	0	2
419	1	0.5	0	4
420	1	0.5	0	4
423	6	2.25	0	2
424	4	1.25	0	2
425	5	1.5	0	2
426	3	1	0	2
427	4	2	0	2
428	1	0.5	0	4
429	5	2.25	0	2
430	3	1.25	0	2
431	1	0.5	0	4
432	1	0.5	0	4
433	3	1.5	0	2
434	1	0.5	0	4

436	1	0.5	0	4
437	1	0.5	0	4
438	1	0.5	0	4
440	1	0.5	0	4
444	1	0.5	0	4
449	1	0.5	0	4
450	2	1	0	2
451	1	0.5	0	4
452	1	0.5	0	4
453	1	0.5	0	4
455	3	1.5	0	2
456	2	1	0	2
458	2	1	0	2
459	1	0.5	0	4
460	2	0.5	0	3
461	6	1.5	0	2
462	10	2.5	0	2
463	4	1	0	2
464	6	1.35	0	2
465	3	0.75	0	3
467	4	1	0	2
468	2	0.5	0	3
469	4	0.95	0	3
470	2	0.45	0	3
471	8	2	0	2
472	3	0.75	0	3
473	3	0.75	0	3
474	4	1	0	2
475	5	1.25	0	2
476	10	2.5	0	2
478	2	1	0	2
479	1	0.5	0	4
480	3	1.5	0	2
482	3	1.5	0	2
483	3	1.5	0	2
484	6	3	0	2
485	4	2	0	2
486	3	1.5	0	2
487	4	2	0	2
490	4	2	0	2
491	2	1	0	2
493	1	0.5	0	4
494	3	1.5	0	2
495	11	5.5	0	2
497	4	2	0	2
499	1	0.5	0	4
500	5	2.5	0	2
502	2	1	0	2
503	1	0.5	0	4
504	2	1	0	2
505	2	1	0	2
506	2	1	0	2
511	1	0.5	0	4
513	2	0.5	0	3
514	5	2	0	2
515	5	2	0	2
516	5	1.5	0	2
517	1	0.5	0	4

518	2	0.75	0	3
519	1	0.25	0	4
520	1	0.5	0	4
521	3	0.75	0	3
524	4	2	0	2
527	2	1	0	2
529	1	0.5	0	4
530	3	1.25	0	2
531	2	0.75	0	3
532	4	1.25	0	2
533	1	0.25	0	4
535	2	0.75	0	3
536	1	0.5	0	4
537	1	0.5	0	4
539	1	0.5	0	4
541	1	0.5	0	4
542	1	0.5	0	4
543	2	1	0	2
544	1	0.5	0	4
545	2	1	0	2
546	1	0.5	0	4
547	1	0.5	0	4
551	1	0.5	0	4
552	9	4.5	0	2
553	3	1.5	0	2
555	2	1	0	2
560	2	1	0	2
561	3	1.5	0	2
564	1	0.5	0	4
567	2	1	0	2
568	1	0.5	0	4
570	1	0.5	0	4
573	5	1.5	0	2
574	5	2	0	2
575	5	1.25	0	2
576	3	0.75	0	3
577	9	2.25	0	2
578	11	2.75	0	2
579	11	2.75	0	2
580	8	2	0	2
581	9	3.25	0	2
582	3	0.75	0	3
583	1	0.5	0	4
584	1	0.5	0	4
585	1	0.5	0	4
587	2	1	0	2
588	3	1.5	0	2
590	1	0.5	0	4
591	1	0.5	0	4
625	2	1	0	2
628	1	0.5	0	4
629	1	0.5	0	4
630	1	0.25	0	4
635	2	1	0	2
637	1	0.5	0	4
638	1	0.5	0	4
640	1	0.5	0	4
644	1	0.5	0	4

646	1	0.5	0	4
651	2	1	0	2
653	2	1	0	2
654	3	1.5	0	2
659	1	0.5	0	4
661	2	1	0	2
663	1	0.5	0	4
665	2	1	0	2
666	3	1.5	0	2
667	1	0.5	0	4
669	1	0.5	0	4
670	2	1	0	2
671	2	1	0	2
673	2	1	0	2
674	2	1	0	2
675	2	1	0	2
677	1	0.5	0	4
678	2	1	0	2
679	2	1	0	2
681	3	1.5	0	2
686	1	0.5	0	4
687	1	0.5	0	4
692	1	0.5	0	4
695	1	0.5	0	4
699	1	0.5	0	4
703	1	0.5	0	4
704	1	0.5	0	4
705	1	0.5	0	4
714	2	1	0	2
716	1	0.5	0	4
721	1	0.5	0	4
723	1	0.5	0	4
729	1	0.5	0	4
731	1	0.5	0	4
732	1	0.5	0	4
733	1	0.5	0	4
739	1	0.5	0	4
740	1	0.5	0	4
742	1	0.5	0	4
744	1	0.5	0	4
761	1	0.5	0	4
798	1	0.5	0	4

---

category=2, cleavage_site=511
query=pto-miR030a,b, target=Potri.008G226500.1,
score=4, range=500-520, strand=1

```
target  5' GGUGUUGAUGAcCAAACUGUC 3'
             .:. :::::::::::: :::
query   3' UCGGAACUACUGGUUUGCCAG 5'
```

>Potri.008G226500.1
#size=1101

253	1	0.5	0	4
257	2	1	0	2
266	1	0.5	0	4
267	1	0.5	0	4
278	1	0.5	0	4
279	1	0.5	0	4
280	2	1	0	2

282	1	0.5	0	4
284	3	1.5	0	2
285	1	0.5	0	4
286	1	0.5	0	4
287	1	0.5	0	4
290	1	0.5	0	4
291	1	0.5	0	4
292	1	0.5	0	4
299	9	4.5	0	2
300	2	1	0	2
302	2	1	0	2
303	2	1	0	2
304	1	0.5	0	4
305	1	0.5	0	4
306	1	0.5	0	4
307	3	1.5	0	2
308	1	0.5	0	4
313	1	0.25	0	4
314	2	1	0	2
315	3	0.75	0	3
316	6	1.5	0	2
317	7	1.75	0	2
318	7	1.75	0	2
319	7	1.75	0	2
320	4	1	0	2
321	6	1.5	0	2
322	7	1.16666666666667	0	2
323	9	1.58333333333333	0	2
324	5	0.833333333333333	0	3
325	5	0.833333333333333	0	3
326	10	1.75	0	2
328	5	0.833333333333333	0	3
329	4	0.666666666666667	0	3
330	5	1	0	3
331	4	0.666666666666667	0	3
332	4	0.666666666666667	0	3
333	1	0.166666666666667	0	4
334	2	0.333333333333333	0	3
335	5	1.25	0	2
336	7	1.75	0	2
337	25	6.25	0	2
338	11	2.75	0	2
339	4	1	0	2
340	12	3	0	2
341	2	0.5	0	3
342	2	0.5	0	3
343	3	0.75	0	3
344	4	1	0	2
345	1	0.25	0	4
346	6	1.5	0	2
347	2	0.5	0	3
348	2	0.5	0	3
349	2	0.5	0	3
350	10	2.5	0	2
351	4	1	0	3
352	8	2.08333333333333	0	2
353	19	3.16666666666667	0	2
354	8	2	0	2

355	6	1.08333333333333	0	2
358	1	0.5	0	4
359	1	0.5	0	4
361	1	0.5	0	4
362	1	0.5	0	4
363	1	0.5	0	4
364	1	0.5	0	4
365	1	0.5	0	4
367	2	1	0	2
372	1	0.5	0	4
374	4	1	0	2
375	2	0.5	0	3
376	2	0.5	0	3
377	5	1.25	0	2
378	4	1	0	2
379	5	1.25	0	2
380	8	2	0	2
381	3	0.75	0	3
382	3	0.75	0	3
383	6	1.5	0	2
384	4	1	0	2
385	6	1.5	0	2
386	10	2.5	0	2
387	8	2	0	2
388	3	0.75	0	3
389	1	0.25	0	4
390	5	1.25	0	2
391	8	2	0	2
392	8	2	0	2
393	23	5.75	0	2
394	6	1.5	0	2
395	1	0.25	0	4
396	4	1	0	2
397	8	2	0	2
398	3	0.75	0	3
399	6	1.5	0	2
400	3	0.75	0	3
401	1	0.25	0	4
402	2	0.5	0	3
403	3	0.75	0	3
405	6	1.5	0	2
406	14	3.5	0	2
407	13	2.16666666666667	0	2
408	4	1	0	2
409	4	1	0	2
410	8	2	0	2
411	6	1.5	0	2
412	5	1.25	0	2
414	3	0.75	0	3
415	6	1.5	0	2
416	6	3	0	2
417	6	3	0	2
418	7	3.5	0	2
419	5	2.5	0	2
420	2	1	0	2
421	13	6.5	0	2
422	6	2.75	0	2
423	10	5	0	2

424	2	1	0	2		
425	1	0.5	0	4		
426	4	2	0	2		
427	2	1	0	2		
428	1	0.5	0	4		
430	3	1.5	0	2		
431	2	1	0	2		
432	2	1	0	2		
433	2	1	0	2		
434	1	0.5	0	4		
435	2	1	0	2		
436	2	1	0	2		
438	5	2.5	0	2		
439	3	1.5	0	2		
440	3	1.5	0	2		
441	4	2	0	2		
442	3	1.5	0	2		
443	1	0.5	0	4		
444	1	0.5	0	4		
445	1	0.5	0	4		
448	1	0.5	0	4		
449	1	0.5	0	4		
450	1	0.5	0	4		
451	1	0.5	0	4		
452	3	1.5	0	2		
453	1	0.5	0	4		
455	1	0.5	0	4		
456	1	0.5	0	4		
457	2	1	0	2		
458	2	1	0	2		
459	1	0.5	0	4		
462	2	1	0	2		
463	1	0.5	0	4		
466	1	0.5	0	4		
467	1	0.25	0	4		
468	1	0.25	0	4		
469	2	0.5	0	3		
470	1	0.25	0	4		
471	1	0.25	0	4		
474	1	0.5	0	4		
475	2	0.75	0	3		
476	6	1.91666666666667			0	2
477	7	2.16666666666667			0	2
478	4	1.91666666666667			1	2
479	2	0.583333333333333			0	3
483	2	0.5	0	3		
485	3	1.5	0	2		
486	2	1	0	2		
488	2	0.5	0	3		
489	2	0.75	0	3		
490	2	0.75	0	3		
491	2	1	0	2		
492	3	1.5	0	2		
493	2	1	0	2		
494	4	2	0	2		
495	4	1.83333333333333			0	2
496	9	4.33333333333333			0	2
497	7	3.25	0	2		

498	2	1	0	2		
499	1	0.5	0	4		
500	2	1	0	2		
501	3	1.5	0	2		
503	3	1.5	0	2		
504	1	0.5	0	4		
505	1	0.5	0	4		
506	3	1.5	0	2		
507	3	1.5	0	2		
509	3	1.5	0	2		
511	2	1	0	2	<<<	
512	3	1.5	0	2		
513	1	0.5	0	4		
514	1	0.5	0	4		
515	1	0.5	0	4		
516	7	3.5	0	2		
517	3	1.5	0	2		
518	4	2	0	2		
520	6	3	0	2		
522	1	0.5	0	4		
523	1	0.5	0	4		
525	2	1	0	2		
526	1	0.5	0	4		
527	3	0.916666666666667			0	3
528	1	0.333333333333333			0	4
529	4	1.33333333333333			0	2
530	6	2	0	2		
531	2	1	0	2		
532	2	1	0	2		
533	2	1	0	2		
534	2	0.833333333333333			0	3
535	2	1	0	2		
536	9	2.2	0	2		
537	1	0.25	0	4		
538	4	1.25	0	2		
539	1	0.2	0	4		
540	3	0.75	0	3		
541	3	0.75	0	3		
542	5	1.25	0	2		
543	4	1	0	2		
544	5	1.25	0	2		
545	8	1.6	0	2		
546	12	2.4	0	2		
547	5	1	0	2		
548	6	2	0	2		
549	9	3	0	2		
550	14	4	0	2		
551	14	4.05	0	2		
552	3	0.75	0	3		
553	2	0.5	0	3		
554	4	2	0	2		
555	2	1	0	2		
556	2	1	0	2		
557	1	0.5	0	4		
559	1	0.5	0	4		
560	1	0.5	0	4		
563	1	0.5	0	4		
566	1	0.5	0	4		

567	2	1	0	2
568	2	1	0	2
569	1	0.5	0	4
571	2	1	0	2
572	4	2	0	2
573	9	4.5	0	2
574	3	1.5	0	2
575	7	3.5	0	2
576	9	4.5	0	2
577	6	3	0	2
578	4	2	0	2
579	5	2.5	0	2
580	1	0.5	0	4
581	3	1.5	0	2
582	1	0.5	0	4
583	3	1.5	0	2
584	1	0.5	0	4
586	1	0.5	0	4
587	1	0.5	0	4
590	2	1	0	2
591	3	1.5	0	2
592	7	3.5	0	2
593	8	4	0	2
594	3	1.5	0	2
595	3	1.5	0	2
596	9	4.5	0	2
597	3	1.5	0	2
598	6	3	0	2
599	4	2	0	2
600	9	4.5	0	2
601	5	2.5	0	2
602	14	7	0	2
603	19	9.5	0	0
604	4	2	0	2
605	5	2.5	0	2
606	4	2	0	2
608	1	0.5	0	4
609	1	0.5	0	4
610	2	1	0	2
611	5	2.5	0	2
613	4	2	0	2
614	8	4	0	2
615	3	1.5	0	2
616	1	0.5	0	4
617	2	1	0	2
619	2	1	0	2
620	3	1.5	0	2
621	1	0.5	0	4
622	2	1	0	2
623	1	0.5	0	4
624	2	1	0	2
625	2	1	0	2
626	2	1	0	2
627	3	1.5	0	2
628	1	0.5	0	4
629	2	1	0	2
632	1	0.5	0	4
633	1	0.5	0	4

634	1	0.5	0	4
635	5	2.5	0	2
636	3	1.5	0	2
637	1	0.5	0	4
638	2	1	0	2
639	1	0.5	0	4
640	3	1.5	0	2
641	1	0.5	0	4
642	1	0.5	0	4
643	3	1.5	0	2
644	2	1	0	2
645	2	1	0	2
647	1	0.5	0	4
648	2	1	0	2
651	3	1.5	0	2
652	1	0.5	0	4
653	2	1	0	2
654	1	0.5	0	4
655	1	0.5	0	4
658	6	2.25	0	2
659	4	1.25	0	2
660	5	1.5	0	2
661	3	1	0	2
662	4	2	0	2
663	1	0.5	0	4
664	5	2.25	0	2
665	3	1.25	0	2
666	1	0.5	0	4
667	1	0.5	0	4
668	3	1.5	0	2
669	1	0.5	0	4
671	1	0.5	0	4
672	1	0.5	0	4
673	1	0.5	0	4
675	1	0.5	0	4
679	1	0.5	0	4
684	1	0.5	0	4
685	2	1	0	2
686	1	0.5	0	4
687	1	0.5	0	4
688	1	0.5	0	4
690	3	1.5	0	2
691	2	1	0	2
693	2	1	0	2
694	1	0.5	0	4
695	2	0.5	0	3
696	6	1.5	0	2
697	10	2.5	0	2
698	4	1	0	2
699	6	1.35	0	2
700	3	0.75	0	3
702	4	1	0	2
703	2	0.5	0	3
704	4	0.95	0	3
705	2	0.45	0	3
706	8	2	0	2
707	3	0.75	0	3
708	3	0.75	0	3

709	4	1	0	2
710	5	1.25	0	2
711	10	2.5	0	2
713	2	1	0	2
714	1	0.5	0	4
715	3	1.5	0	2
717	3	1.5	0	2
718	3	1.5	0	2
719	6	3	0	2
720	4	2	0	2
721	3	1.5	0	2
722	4	2	0	2
725	4	2	0	2
726	2	1	0	2
728	1	0.5	0	4
729	3	1.5	0	2
730	11	5.5	0	2
732	4	2	0	2
734	1	0.5	0	4
735	5	2.5	0	2
737	2	1	0	2
738	1	0.5	0	4
739	2	1	0	2
740	2	1	0	2
741	2	1	0	2
746	1	0.5	0	4
748	2	0.5	0	3
749	5	2	0	2
750	5	2	0	2
751	5	1.5	0	2
752	1	0.5	0	4
753	2	0.75	0	3
754	1	0.25	0	4
755	1	0.5	0	4
756	3	0.75	0	3
759	4	2	0	2
762	2	1	0	2
764	1	0.5	0	4
765	3	1.25	0	2
766	2	0.75	0	3
767	4	1.25	0	2
768	1	0.25	0	4
770	2	0.75	0	3
771	1	0.5	0	4
772	1	0.5	0	4
774	1	0.5	0	4
776	1	0.5	0	4
777	1	0.5	0	4
778	2	1	0	2
779	1	0.5	0	4
780	2	1	0	2
781	1	0.5	0	4
782	1	0.5	0	4
786	1	0.5	0	4
787	9	4.5	0	2
788	3	1.5	0	2
790	2	1	0	2
795	2	1	0	2

796	3	1.5	0	2
799	1	0.5	0	4
802	2	1	0	2
803	1	0.5	0	4
805	1	0.5	0	4
808	5	1.5	0	2
809	5	2	0	2
810	5	1.25	0	2
811	3	0.75	0	3
812	9	2.25	0	2
813	11	2.75	0	2
814	11	2.75	0	2
815	8	2	0	2
816	9	3.25	0	2
817	3	0.75	0	3
818	1	0.5	0	4
819	1	0.5	0	4
820	1	0.5	0	4
822	2	1	0	2
823	3	1.5	0	2
825	1	0.5	0	4
826	1	0.5	0	4
860	2	1	0	2
863	1	0.5	0	4
864	1	0.5	0	4
865	1	0.25	0	4
870	2	1	0	2
872	1	0.5	0	4
873	1	0.5	0	4
875	1	0.5	0	4
879	1	0.5	0	4
881	1	0.5	0	4
886	2	1	0	2
888	2	1	0	2
889	3	1.5	0	2
894	1	0.5	0	4
896	2	1	0	2
898	1	0.5	0	4
900	2	1	0	2
901	3	1.5	0	2
902	1	0.5	0	4
904	1	0.5	0	4
905	2	1	0	2
906	2	1	0	2
908	2	1	0	2
909	2	1	0	2
910	2	1	0	2
912	1	0.5	0	4
913	2	1	0	2
914	2	1	0	2
916	3	1.5	0	2
921	1	0.5	0	4
922	1	0.5	0	4
927	1	0.5	0	4
930	1	0.5	0	4
934	1	0.5	0	4
938	1	0.5	0	4
939	1	0.5	0	4

940	1	0.5	0	4
949	2	1	0	2
951	1	0.5	0	4
956	1	0.5	0	4
958	1	0.5	0	4
964	1	0.5	0	4
966	1	0.5	0	4
967	1	0.5	0	4
968	1	0.5	0	4
974	1	0.5	0	4
975	1	0.5	0	4
977	1	0.5	0	4
979	1	0.5	0	4
996	1	0.5	0	4
1033	1	0.5	0	4

pto-miR037a,b

category=2, cleavage_site=683
query=pto-miR037a,b, target=Potri.015G125100.1,
score=4, range=672-692, strand=1

```
target  5' UGGGCACUGAUcAAGCUGUUA 3'
           ::: :: :::::::: :::::
query   3' ACCGGUAACUAGUUCUACAAU 5'
```

>Potri.015G125100.1
#size=1187

119	1	1	1	4
121	1	1	1	4
127	1	1	1	4
177	1	1	1	4
183	1	1	1	4
186	1	1	1	4
221	1	1	1	4
266	2	2	2	2
358	1	1	1	4
377	1	1	1	4
392	1	1	1	4
395	1	1	1	4
400	1	1	1	4
403	1	1	1	4
413	1	1	1	4
421	1	1	1	4
430	1	1	1	4
452	1	1	1	4
456	1	1	1	4
459	1	1	1	4
469	1	1	1	4
477	1	1	1	4
483	2	2	2	2
489	1	1	1	4
493	1	1	1	4
509	1	1	1	4
511	1	1	1	4
535	1	1	1	4
545	1	1	1	4
556	1	1	1	4
565	1	1	1	4

575	2	2	2	2	
591	1	1	1	4	
594	1	1	1	4	
602	1	1	1	4	
606	2	2	2	2	
608	1	1	1	4	
614	1	1	1	4	
615	1	1	1	4	
617	1	1	1	4	
626	1	1	1	4	
632	1	1	1	4	
635	2	2	2	2	
637	1	1	1	4	
639	3	3	3	2	
641	1	1	1	4	
644	2	2	2	2	
645	2	2	2	2	
646	1	1	1	4	
648	1	1	1	4	
649	2	2	2	2	
651	5	5	5	0	
653	3	3	3	2	
654	1	1	1	4	
655	1	1	1	4	
659	1	1	1	4	
660	3	3	3	2	
665	1	1	1	4	
669	1	1	1	4	
673	1	1	1	4	
676	1	1	1	4	
679	1	1	1	4	
680	1	1	1	4	
683	2	2	2	2	<<<
686	1	1	1	4	
701	1	1	1	4	
704	1	1	1	4	
707	2	2	2	2	
711	1	1	1	4	
732	1	1	1	4	
747	1	1	1	4	
755	1	1	1	4	
778	1	1	1	4	
821	1	1	1	4	
829	1	1	1	4	

category=2, cleavage_site=1379
query=pto-miR037a,b, target=Potri.001G240900.1,
score=3.5, range=1369-1388, strand=1

```
target  5' UAUAGCA-CUUgAUCAAUGGC 3'
           ::::.:: :::.::::::::
query   3' AUAUUGUAGAAUUAGUUACCC 5'
```

>Potri.001G240900.1
#size=1625

413	1	0.25	0	4
474	1	0.333333333333333	0	4
533	1	0.333333333333333	0	4
585	1	0.333333333333333	0	4
591	1	0.333333333333333	0	4

597	1	0.333333333333333	0	4
599	1	0.333333333333333	0	4
612	1	0.142857142857143	0	4
620	1	0.333333333333333	0	4
622	1	0.333333333333333	0	4
625	1	0.333333333333333	0	4
634	1	0.333333333333333	0	4
643	2	1	0	2
647	1	0.25	0	4
656	3	0.733333333333333	0	3
661	1	0.25	0	4
670	1	0.333333333333333	0	4
675	1	0.333333333333333	0	4
680	1	0.333333333333333	0	4
682	1	0.333333333333333	0	4
683	2	0.666666666666667	0	3
686	1	0.333333333333333	0	4
692	1	0.333333333333333	0	4
694	1	0.333333333333333	0	4
754	1	0.25	0	4
755	1	0.2	0	4
759	1	0.2	0	4
763	2	0.666666666666667	0	3
768	1	0.2	0	4
769	4	0.666666666666667	0	3
770	1	0.125	0	4
772	3	0.375	0	3
773	2	0.4	0	3
774	3	0.6	0	3
775	1	0.2	0	4
780	2	0.666666666666667	0	3
788	1	0.333333333333333	0	4
789	2	0.5	0	3
792	1	0.25	0	4
794	1	0.25	0	4
808	1	0.25	0	4
810	1	0.25	0	4
813	1	0.25	0	4
814	3	0.75	0	3
816	1	0.25	0	4
824	1	0.25	0	4
825	2	0.5	0	3
826	1	0.25	0	4
828	3	2	1	2
829	1	0.25	0	4
830	1	0.25	0	4
834	1	0.5	0	4
835	1	0.25	0	4
838	3	0.916666666666667	0	3
841	1	0.25	0	4
842	2	0.416666666666667	0	3
843	2	0.333333333333333	0	3
844	4	0.666666666666667	0	3
845	4	0.783333333333333	0	3
847	5	1.01666666666667	0	2
848	1	0.166666666666667	0	4
850	1	0.2	0	4
854	5	0.9	0	3

855	2	0.366666666666667	0	3
858	2	0.366666666666667	0	3
859	1	0.2	0	4
860	4	1.06666666666667	0	2
862	1	0.166666666666667	0	4
864	2	0.4	0	3
866	2	0.366666666666667	0	3
867	4	0.666666666666667	0	3
872	1	0.2	0	4
879	1	0.5	0	4
881	1	0.5	0	4
884	1	0.5	0	4
885	1	0.5	0	4
886	1	0.5	0	4
896	1	1	1	4
897	3	3	3	2
898	3	3	3	2
905	1	1	1	4
917	3	1.5	0	2
918	1	0.5	0	4
919	1	0.5	0	4
923	1	0.5	0	4
924	2	1	0	2
925	1	0.5	0	4
929	1	0.5	0	4
930	3	1.5	0	2
938	3	2	1	2
940	1	0.5	0	4
941	1	0.5	0	4
944	1	0.333333333333333	0	4
947	1	0.333333333333333	0	4
950	2	0.666666666666667	0	3
952	2	0.666666666666667	0	3
956	1	0.333333333333333	0	4
957	1	0.333333333333333	0	4
958	6	2	0	2
959	12	4	0	2
960	1	0.333333333333333	0	4
961	3	1	0	2
963	1	0.333333333333333	0	4
964	1	0.333333333333333	0	4
965	1	0.333333333333333	0	4
966	1	0.333333333333333	0	4
967	1	0.333333333333333	0	4
968	8	2.66666666666667	0	2
969	9	3	0	2
970	1	0.333333333333333	0	4
972	2	0.666666666666667	0	3
976	1	0.333333333333333	0	4
977	1	0.333333333333333	0	4
979	3	1	0	2
982	2	0.666666666666667	0	3
986	3	1	0	2
989	2	0.666666666666667	0	3
990	4	1.33333333333333	0	2
991	6	2	0	2
994	2	1.33333333333333	1	2
995	1	0.333333333333333	0	4

996	1	0.333333333333333	0	4
997	1	0.333333333333333	0	4
998	3	1	0	2
999	3	1	0	2
1000	10	3.33333333333333	0	2
1001	3	1	0	2
1002	3	1	0	2
1003	3	1	0	2
1004	1	0.333333333333333	0	4
1005	1	0.333333333333333	0	4
1007	3	1	0	2
1008	28	17.3333333333333	12	2
1009	1	1	1	4
1010	2	2	2	2
1011	1	1	1	4
1015	2	2	2	2
1017	7	7	7	2
1018	5	5	5	2
1019	7	7	7	2
1027	2	2	2	2
1028	4	4	4	2
1029	1	1	1	4
1031	1	1	1	4
1034	2	2	2	2
1100	1	1	1	4
1101	1	1	1	4
1115	1	1	1	4
1132	1	1	1	4
1143	1	1	1	4
1161	1	1	1	4
1191	1	1	1	4
1193	1	1	1	4
1211	2	0.666666666666667	0	3
1213	1	1	1	4
1215	1	1	1	4
1236	1	1	1	4
1237	2	2	2	2
1239	1	1	1	4
1241	1	1	1	4
1242	1	1	1	4
1243	7	7	7	2
1244	11	11	11	2
1245	5	5	5	2
1246	2	2	2	2
1248	5	5	5	2
1249	7	7	7	2
1250	1	1	1	4
1251	1	1	1	4
1253	7	7	7	2
1254	1	1	1	4
1255	1	1	1	4
1257	2	2	2	2
1258	2	2	2	2
1259	3	3	3	2
1260	3	1.66666666666667	1	2
1261	1	0.333333333333333	0	4
1266	2	0.666666666666667	0	3
1267	2	0.666666666666667	0	3

1268	6	2	0	2		
1269	10	4	1	2		
1271	2	2	2	2		
1272	1	1	1	4		
1273	1	1	1	4		
1276	3	3	3	2		
1277	26	26	26	2		
1278	9	9	9	2		
1279	6	6	6	2		
1309	1	1	1	4		
1310	2	2	2	2		
1327	1	1	1	4		
1328	5	4.33333333333333			4	2
1329	6	4.66666666666667			4	2
1330	7	3.66666666666667			2	2
1331	14	11.3333333333333			10	2
1332	19	13.6666666666667			11	2
1333	1	1	1	4		
1334	2	1.33333333333333			1	2
1335	7	6.33333333333333			6	2
1336	15	13.6666666666667			13	2
1337	11	7.66666666666667			6	2
1338	5	1.66666666666667			0	2
1339	7	2.33333333333333			0	2
1340	5	1.66666666666667			0	2
1341	9	3	0	2		
1342	8	3.33333333333333			1	2
1343	6	6	6	2		
1344	5	4.33333333333333			4	2
1345	13	10.3333333333333			9	2
1346	17	15	14	2		
1347	8	2.66666666666667			0	2
1348	2	0.666666666666667			0	3
1349	14	4.66666666666667			0	2
1350	24	8	0	2		
1351	14	4.66666666666667			0	2
1352	43	15	1	2		
1353	64	64	64	2		
1354	16	16	16	2		
1355	71	71	71	2		
1356	70	70	70	2		
1357	20	20	20	2		
1358	41	41	41	2		
1359	68	68	68	2		
1360	39	39	39	2		
1361	38	38	38	2		
1362	11	11	11	2		
1363	30	30	30	2		
1364	80	80	80	2		
1365	49	49	49	2		
1366	24	24	24	2		
1367	125	125	125	2		
1368	128	128	128	2		
1369	158	158	158	0		
1370	104	104	104	2		
1371	23	23	23	2		
1372	2	2	2	2		
1373	5	5	5	2		

1374	7	7	7	2	
1375	3	3	3	2	
1376	6	6	6	2	
1377	21	21	21	2	
1378	23	23	23	2	
1379	12	12	12	2	<<<
1380	4	4	4	2	
1381	4	4	4	2	
1385	3	3	3	2	
1388	1	1	1	4	
1389	1	1	1	4	
1393	1	1	1	4	
1395	1	1	1	4	
1397	1	1	1	4	
1398	1	1	1	4	
1401	4	4	4	2	
1405	1	1	1	4	
1408	4	4	4	2	
1409	2	2	2	2	
1410	1	1	1	4	
1413	2	2	2	2	
1414	2	2	2	2	
1415	3	3	3	2	
1451	1	1	1	4	
1524	1	1	1	4	
1525	1	1	1	4	
1527	1	1	1	4	
1529	1	1	1	4	
1592	1	1	1	4	
1598	1	1	1	4	

pto-miR040a,b

category=4, cleavage_site=857
query=pto-miR040a,b, target=Potri.001G331500.1,
score=3, range=846-866, strand=1

```
target  5' AAAACCUCAGGaUACAGAUUA 3'
             ::::::::::: :::::::
query   3' GUUUGGAGUCCUACGUCUAAU 5'
```

>Potri.001G331500.1
#size=2936

286	1	1	1	4
357	2	1	0	2
363	1	0.5	0	4
565	1	1	1	4
567	1	1	1	4
581	1	0.333333333333333	0	4
604	3	0.6	0	2
615	1	0.2	0	4
634	1	0.25	0	4
640	1	0.2	0	4
647	1	0.25	0	4
651	1	0.25	0	4
658	1	0.2	0	4
664	1	0.25	0	4
673	1	0.25	0	4
706	1	0.25	0	4

840	1	0.25	0	4	
857	1	0.25	0	4	<<<
886	1	0.25	0	4	
905	1	0.2	0	4	
908	1	0.2	0	4	
934	1	0.25	0	4	
949	1	0.25	0	4	
950	1	0.25	0	4	
951	1	0.2	0	4	
983	1	0.25	0	4	
992	1	0.25	0	4	
1011	1	0.25	0	4	
1015	2	0.5	0	2	
1039	1	0.2	0	4	
1043	1	0.25	0	4	
1063	2	0.5	0	2	
1071	2	0.5	0	2	
1083	1	0.25	0	4	
1116	1	0.25	0	4	
1139	1	0.2	0	4	
1143	1	0.2	0	4	
1148	1	0.2	0	4	
1156	1	0.2	0	4	
1170	1	0.25	0	4	
1191	2	0.5	0	2	
1267	1	0.25	0	4	
1276	1	0.2	0	4	
1278	2	0.4	0	2	
1292	2	0.4	0	2	
1319	2	0.5	0	2	
1368	1	0.2	0	4	
1390	1	0.25	0	4	
1400	2	0.5	0	2	
1425	2	0.4	0	2	
1444	1	0.25	0	4	
1448	1	0.25	0	4	
1449	1	0.25	0	4	
1450	1	0.25	0	4	
1451	1	0.25	0	4	
1462	1	0.25	0	4	
1465	1	0.25	0	4	
1468	1	0.25	0	4	
1469	1	0.25	0	4	
1471	4	1	0	2	
1476	1	0.25	0	4	
1481	2	0.5	0	2	
1489	2	0.5	0	2	
1500	2	0.4	0	2	
1506	1	0.2	0	4	
1526	1	0.2	0	4	
1534	1	0.2	0	4	
1543	1	0.2	0	4	
1545	1	0.2	0	4	
1555	1	0.2	0	4	
1560	1	0.2	0	4	
1586	1	0.2	0	4	
1588	1	0.2	0	4	
1599	1	0.25	0	4	

1609	1	0.25	0	4
1622	1	0.25	0	4
1639	1	0.25	0	4
1641	1	0.25	0	4
1648	1	0.2	0	4
1670	1	0.2	0	4
1686	1	0.25	0	4
1691	3	0.6	0	2
1704	2	0.4	0	2
1711	1	0.2	0	4
1729	1	0.2	0	4
1733	2	0.4	0	2
1739	1	0.25	0	4
1745	1	0.25	0	4
1782	1	0.25	0	4
1809	1	0.25	0	4
1816	1	0.25	0	4
1823	2	0.5	0	2
1834	1	0.2	0	4
1841	1	0.2	0	4
1845	1	0.2	0	4
1859	1	0.2	0	4
1865	2	0.4	0	2
1875	1	0.2	0	4
1880	1	0.2	0	4
1886	1	0.2	0	4
1887	1	0.2	0	4
1903	2	0.4	0	2
1905	1	0.2	0	4
1927	1	0.2	0	4
1930	1	0.2	0	4
1934	1	0.25	0	4
1935	1	0.25	0	4
1943	1	0.25	0	4
1949	1	0.25	0	4
1950	1	0.25	0	4
1951	1	0.25	0	4
1952	3	0.75	0	2
1954	1	0.25	0	4
1963	1	0.25	0	4
1968	2	0.4	0	2
1979	1	0.2	0	4
1986	2	0.4	0	2
1996	1	0.25	0	4
1997	2	0.5	0	2
2000	1	0.25	0	4
2017	1	0.25	0	4
2053	1	0.25	0	4
2058	1	0.2	0	4
2063	1	0.25	0	4
2085	1	0.25	0	4
2102	1	0.25	0	4
2105	1	0.25	0	4
2106	1	0.25	0	4
2107	1	0.25	0	4
2110	1	0.25	0	4
2111	5	1.25	0	0
2116	1	0.25	0	4

2117	1	0.25	0	4
2118	2	0.5	0	2
2120	1	0.25	0	4
2122	1	0.25	0	4
2148	1	0.25	0	4
2192	1	0.25	0	4
2287	1	0.25	0	4
2686	1	0.25	0	4
2687	1	0.25	0	4
2801	1	0.25	0	4

pto-miR042a,b

category=2, cleavage_site=2304
query=pto-miR042a,b, target=Potri.007G026400.1,
score=3, range=2294-2313, strand=1

```
target  5' GUAAGUGAAUgCUGAUUCAU 3'
           ..::::::::::::::.:::
query   3' UGUUCACUUACGACUGAGUC 5'
```

>Potri.007G026400.1
#size=2371

138	1	0.2	0	4
158	1	0.2	0	4
169	1	0.2	0	4
178	1	0.2	0	4
186	1	0.2	0	4
201	1	0.2	0	4
203	1	0.2	0	4
209	2	0.4	0	2
221	1	0.2	0	4
223	1	0.2	0	4
245	1	0.2	0	4
252	1	0.2	0	4
257	1	0.2	0	4
282	1	0.2	0	4
287	1	0.2	0	4
289	1	0.2	0	4
295	1	0.166666666666667	0	4
296	3	0.5	0	2
297	1	0.166666666666667	0	4
309	1	0.125	0	4
310	1	0.125	0	4
312	1	0.125	0	4
314	4	0.5	0	2
315	2	0.25	0	2
316	1	0.125	0	4
317	1	0.125	0	4
318	7	0.875	0	2
320	2	0.25	0	2
321	1	0.125	0	4
322	1	0.125	0	4
323	1	0.125	0	4
326	2	0.25	0	2
327	1	0.125	0	4
328	2	0.25	0	2
330	1	0.166666666666667	0	4
332	5	0.625	0	2

333	1	0.125	0	4
335	2	0.25	0	2
337	2	0.333333333333333	0	2
342	1	0.166666666666667	0	4
345	1	0.166666666666667	0	4
350	2	0.25	0	2
357	1	0.125	0	4
363	1	0.125	0	4
370	1	0.125	0	4
375	1	0.125	0	4
379	2	0.25	0	2
380	1	0.125	0	4
382	1	0.125	0	4
385	1	0.125	0	4
394	2	0.25	0	2
428	1	0.166666666666667	0	4
435	2	0.333333333333333	0	2
436	1	0.125	0	4
441	1	0.125	0	4
445	1	0.125	0	4
448	1	0.125	0	4
451	1	0.125	0	4
453	1	0.125	0	4
465	1	0.166666666666667	0	4
470	1	0.166666666666667	0	4
478	1	0.166666666666667	0	4
484	1	0.125	0	4
489	1	0.166666666666667	0	4
490	1	0.166666666666667	0	4
492	1	0.166666666666667	0	4
496	1	0.166666666666667	0	4
500	1	0.125	0	4
501	1	0.125	0	4
502	1	0.125	0	4
503	1	0.125	0	4
505	2	0.25	0	2
507	1	0.125	0	4
512	1	0.125	0	4
517	1	0.125	0	4
519	1	0.125	0	4
521	1	0.125	0	4
522	1	0.125	0	4
525	1	0.125	0	4
539	1	0.125	0	4
545	1	0.125	0	4
557	1	0.125	0	4
561	2	0.25	0	2
569	1	0.125	0	4
570	1	0.125	0	4
571	2	0.25	0	2
575	1	0.125	0	4
585	1	0.125	0	4
597	1	0.166666666666667	0	4
599	1	0.166666666666667	0	4
603	1	0.166666666666667	0	4
617	1	0.166666666666667	0	4
621	1	0.125	0	4
629	1	0.125	0	4

637	1	0.125	0	4
639	1	0.125	0	4
643	2	0.25	0	2
648	1	0.125	0	4
649	3	0.375	0	2
652	1	0.125	0	4
655	1	0.125	0	4
658	1	0.125	0	4
659	1	0.166666666666667	0	4
670	2	0.333333333333333	0	2
680	1	0.166666666666667	0	4
682	1	0.125	0	4
683	1	0.166666666666667	0	4
688	1	0.125	0	4
691	1	0.125	0	4
695	1	0.125	0	4
699	3	0.375	0	2
704	1	0.166666666666667	0	4
707	1	0.166666666666667	0	4
708	1	0.166666666666667	0	4
713	1	0.125	0	4
733	1	0.125	0	4
735	1	0.125	0	4
751	1	0.125	0	4
757	1	0.166666666666667	0	4
759	1	0.166666666666667	0	4
762	1	0.166666666666667	0	4
764	1	0.166666666666667	0	4
767	2	0.333333333333333	0	2
783	2	0.333333333333333	0	2
787	1	0.166666666666667	0	4
791	1	0.166666666666667	0	4
797	2	0.333333333333333	0	2
808	1	0.166666666666667	0	4
810	1	0.125	0	4
811	1	0.166666666666667	0	4
813	1	0.125	0	4
818	1	0.166666666666667	0	4
820	1	0.125	0	4
823	1	0.166666666666667	0	4
826	2	0.25	0	2
834	1	0.125	0	4
849	1	0.125	0	4
850	2	0.25	0	2
859	1	0.125	0	4
865	2	0.25	0	2
867	1	0.125	0	4
869	1	0.166666666666667	0	4
870	1	0.166666666666667	0	4
878	1	0.166666666666667	0	4
883	2	0.333333333333333	0	2
899	2	0.333333333333333	0	2
911	1	0.166666666666667	0	4
919	1	0.166666666666667	0	4
920	1	0.166666666666667	0	4
923	1	0.166666666666667	0	4
927	1	0.166666666666667	0	4
929	1	0.166666666666667	0	4

938	1	0.166666666666667	0	4
940	1	0.125	0	4
943	1	0.125	0	4
944	1	0.125	0	4
945	1	0.125	0	4
948	1	0.125	0	4
949	1	0.125	0	4
951	2	0.25	0	2
952	1	0.125	0	4
955	1	0.125	0	4
956	1	0.125	0	4
957	1	0.125	0	4
960	1	0.125	0	4
961	1	0.125	0	4
964	1	0.125	0	4
973	1	0.166666666666667	0	4
977	1	0.125	0	4
984	1	0.125	0	4
990	1	0.166666666666667	0	4
1005	1	0.166666666666667	0	4
1010	1	0.125	0	4
1013	1	0.125	0	4
1014	2	0.25	0	2
1024	1	0.125	0	4
1026	1	0.166666666666667	0	4
1030	1	0.166666666666667	0	4
1040	1	0.166666666666667	0	4
1049	1	0.166666666666667	0	4
1056	1	0.166666666666667	0	4
1060	1	0.166666666666667	0	4
1063	1	0.125	0	4
1066	3	0.375	0	2
1067	1	0.166666666666667	0	4
1070	2	0.333333333333333	0	2
1073	1	0.125	0	4
1076	1	0.125	0	4
1083	2	0.333333333333333	0	2
1085	1	0.166666666666667	0	4
1087	2	0.333333333333333	0	2
1088	4	0.666666666666667	0	2
1089	1	0.166666666666667	0	4
1092	2	0.333333333333333	0	2
1097	1	0.166666666666667	0	4
1099	1	0.166666666666667	0	4
1100	2	0.333333333333333	0	2
1103	1	0.125	0	4
1104	1	0.125	0	4
1105	1	0.125	0	4
1106	1	0.125	0	4
1107	1	0.125	0	4
1108	1	0.125	0	4
1112	1	0.125	0	4
1114	1	0.125	0	4
1116	1	0.125	0	4
1118	2	0.25	0	2
1119	1	0.125	0	4
1122	1	0.125	0	4
1130	2	0.25	0	2

1131	1	0.125	0	4
1133	1	0.125	0	4
1134	1	0.125	0	4
1135	2	0.25	0	2
1138	1	0.125	0	4
1141	1	0.125	0	4
1146	1	0.125	0	4
1153	1	0.125	0	4
1163	2	0.25	0	2
1176	1	0.125	0	4
1177	1	0.125	0	4
1181	1	0.125	0	4
1190	1	0.125	0	4
1192	2	0.25	0	2
1195	1	0.125	0	4
1196	1	0.125	0	4
1202	5	0.625	0	2
1206	1	0.125	0	4
1211	3	0.5	0	2
1213	2	0.333333333333333	0	2
1214	1	0.125	0	4
1215	1	0.125	0	4
1216	1	0.125	0	4
1217	1	0.125	0	4
1219	3	0.375	0	2
1220	2	0.25	0	2
1224	1	0.125	0	4
1225	1	0.125	0	4
1226	1	0.125	0	4
1229	1	0.125	0	4
1232	2	0.25	0	2
1233	1	0.125	0	4
1234	2	0.25	0	2
1235	1	0.125	0	4
1238	3	0.375	0	2
1239	1	0.125	0	4
1241	2	0.25	0	2
1243	1	0.125	0	4
1248	2	0.25	0	2
1251	1	0.125	0	4
1253	2	0.25	0	2
1256	1	0.125	0	4
1257	1	0.125	0	4
1259	1	0.125	0	4
1262	1	0.125	0	4
1263	2	0.25	0	2
1266	1	0.125	0	4
1286	2	0.25	0	2
1299	1	0.125	0	4
1310	1	0.125	0	4
1314	1	0.125	0	4
1336	1	0.166666666666667	0	4
1337	1	0.166666666666667	0	4
1338	1	0.166666666666667	0	4
1343	1	0.166666666666667	0	4
1345	1	0.166666666666667	0	4
1346	1	0.166666666666667	0	4
1348	2	0.333333333333333	0	2

1351	1	0.166666666666667	0	4
1357	1	0.166666666666667	0	4
1360	1	0.166666666666667	0	4
1361	1	0.166666666666667	0	4
1366	1	0.166666666666667	0	4
1370	1	0.166666666666667	0	4
1373	1	0.166666666666667	0	4
1375	1	0.166666666666667	0	4
1376	1	0.166666666666667	0	4
1378	1	0.166666666666667	0	4
1384	1	0.166666666666667	0	4
1391	1	0.166666666666667	0	4
1392	1	0.166666666666667	0	4
1397	1	0.125	0	4
1400	1	0.166666666666667	0	4
1401	1	0.166666666666667	0	4
1403	2	0.333333333333333	0	2
1406	1	0.166666666666667	0	4
1408	1	0.166666666666667	0	4
1411	1	0.166666666666667	0	4
1412	1	0.166666666666667	0	4
1414	2	0.333333333333333	0	2
1418	1	0.166666666666667	0	4
1420	1	0.166666666666667	0	4
1426	1	0.166666666666667	0	4
1429	1	0.166666666666667	0	4
1430	2	0.333333333333333	0	2
1432	1	0.166666666666667	0	4
1436	1	0.166666666666667	0	4
1438	1	0.166666666666667	0	4
1442	1	0.166666666666667	0	4
1444	1	0.166666666666667	0	4
1446	1	0.166666666666667	0	4
1448	1	0.166666666666667	0	4
1450	1	0.166666666666667	0	4
1455	1	0.125	0	4
1479	1	0.125	0	4
1491	1	0.125	0	4
1495	1	0.125	0	4
1500	1	0.125	0	4
1511	1	0.125	0	4
1513	1	0.125	0	4
1514	1	0.125	0	4
1515	3	0.375	0	2
1516	1	0.125	0	4
1518	1	0.125	0	4
1523	1	0.125	0	4
1525	3	0.375	0	2
1526	1	0.125	0	4
1527	2	0.25	0	2
1528	1	0.125	0	4
1529	1	0.125	0	4
1530	1	0.125	0	4
1533	2	0.25	0	2
1534	1	0.125	0	4
1537	2	0.25	0	2
1539	3	0.416666666666667	0	2
1541	1	0.166666666666667	0	4

1545	1	0.166666666666667	0	4
1546	2	0.333333333333333	0	2
1547	1	0.166666666666667	0	4
1548	2	0.333333333333333	0	2
1550	1	0.166666666666667	0	4
1551	1	0.166666666666667	0	4
1554	3	0.5	0	2
1558	2	0.333333333333333	0	2
1560	1	0.2	0	4
1561	2	0.4	0	2
1563	1	0.2	0	4
1565	2	0.4	0	2
1566	1	0.2	0	4
1567	2	0.4	0	2
1568	1	0.2	0	4
1569	3	0.6	0	2
1570	3	0.6	0	2
1572	1	0.2	0	4
1573	2	0.4	0	2
1574	1	0.2	0	4
1575	2	0.333333333333333	0	2
1576	1	0.166666666666667	0	4
1577	2	0.333333333333333	0	2
1578	3	0.5	0	2
1579	1	0.166666666666667	0	4
1580	2	0.333333333333333	0	2
1581	2	0.333333333333333	0	2
1582	5	0.833333333333333	0	2
1587	2	0.333333333333333	0	2
1589	1	0.166666666666667	0	4
1590	1	0.166666666666667	0	4
1592	1	0.166666666666667	0	4
1594	1	0.142857142857143	0	4
1595	1	0.2	0	4
1597	2	0.285714285714286	0	2
1599	2	0.291666666666667	0	2
1600	3	0.375	0	2
1601	1	0.166666666666667	0	4
1603	3	0.375	0	2
1605	1	0.166666666666667	0	4
1606	1	0.166666666666667	0	4
1607	1	0.166666666666667	0	4
1609	3	0.5	0	2
1610	1	0.166666666666667	0	4
1611	6	1	0	2
1612	2	0.333333333333333	0	2
1614	1	0.166666666666667	0	4
1616	2	0.333333333333333	0	2
1617	2	0.333333333333333	0	2
1618	5	0.833333333333333	0	2
1619	4	0.666666666666667	0	2
1620	6	1	0	2
1621	3	0.5	0	2
1622	3	0.5	0	2
1623	3	0.5	0	2
1624	3	0.5	0	2
1625	1	0.125	0	4
1626	2	0.25	0	2

1627	2	0.333333333333333	0	2
1628	6	0.916666666666667	0	2
1629	6	0.875	0	2
1630	4	0.5	0	2
1631	1	0.125	0	4
1632	3	0.375	0	2
1636	1	0.125	0	4
1640	2	0.25	0	2
1642	1	0.125	0	4
1643	2	0.25	0	2
1647	1	0.125	0	4
1648	1	0.125	0	4
1649	2	0.25	0	2
1650	1	0.125	0	4
1651	1	0.125	0	4
1652	3	0.5	0	2
1653	2	0.333333333333333	0	2
1654	2	0.333333333333333	0	2
1655	4	0.625	0	2
1658	1	0.166666666666667	0	4
1661	2	0.333333333333333	0	2
1662	1	0.166666666666667	0	4
1663	4	0.666666666666667	0	2
1664	1	0.166666666666667	0	4
1666	1	0.166666666666667	0	4
1667	1	0.166666666666667	0	4
1668	1	0.166666666666667	0	4
1673	1	0.166666666666667	0	4
1674	1	0.125	0	4
1676	1	0.166666666666667	0	4
1677	5	0.666666666666667	0	2
1678	1	0.125	0	4
1680	4	0.5	0	2
1681	2	0.25	0	2
1682	3	0.375	0	2
1683	5	0.625	0	2
1684	3	0.5	0	2
1685	1	0.166666666666667	0	4
1686	1	0.166666666666667	0	4
1689	2	0.25	0	2
1690	1	0.125	0	4
1692	4	0.5	0	2
1693	3	0.375	0	2
1694	2	0.333333333333333	0	2
1696	2	0.333333333333333	0	2
1697	1	0.166666666666667	0	4
1698	2	0.333333333333333	0	2
1701	1	0.166666666666667	0	4
1702	5	0.833333333333333	0	2
1703	1	0.166666666666667	0	4
1704	2	0.333333333333333	0	2
1705	3	0.5	0	2
1707	2	0.333333333333333	0	2
1708	1	0.166666666666667	0	4
1709	3	0.5	0	2
1710	2	0.333333333333333	0	2
1711	2	0.333333333333333	0	2
1712	2	0.333333333333333	0	2

1713	1	0.166666666666667	0	4
1714	4	0.666666666666667	0	2
1715	5	0.833333333333333	0	2
1716	1	0.166666666666667	0	4
1717	1	0.166666666666667	0	4
1718	8	1.33333333333333	0	2
1720	3	0.5	0	2
1721	2	0.333333333333333	0	2
1722	5	0.833333333333333	0	2
1723	1	0.166666666666667	0	4
1724	3	0.5	0	2
1725	4	0.666666666666667	0	2
1726	9	1.5	0	2
1727	6	1	0	2
1728	8	1.33333333333333	0	2
1729	5	0.833333333333333	0	2
1730	12	2	0	2
1731	3	0.5	0	2
1732	10	1.66666666666667	0	2
1733	5	0.833333333333333	0	2
1734	3	0.5	0	2
1735	7	1.16666666666667	0	2
1736	1	0.166666666666667	0	4
1737	5	0.625	0	2
1738	3	0.458333333333333	0	2
1739	7	1.04166666666667	0	2
1740	12	1.54166666666667	0	2
1741	21	2.95833333333333	0	2
1742	10	1.25	0	2
1743	13	1.625	0	2
1744	8	1	0	2
1745	4	0.541666666666667	0	2
1746	6	0.791666666666667	0	2
1747	6	0.875	0	2
1748	6	0.791666666666667	0	2
1749	13	1.66666666666667	0	2
1750	13	1.79166666666667	0	2
1751	12	1.625	0	2
1752	12	1.66666666666667	0	2
1753	2	0.291666666666667	0	2
1754	8	1.08333333333333	0	2
1755	12	1.58333333333333	0	2
1756	14	2	0	2
1757	6	0.875	0	2
1758	12	1.875	0	2
1759	8	1.125	0	2
1760	8	1	0	2
1761	15	2.08333333333333	0	2
1762	15	2	0	2
1763	11	1.375	0	2
1764	14	1.75	0	2
1765	18	2.29166666666667	0	2
1766	19	2.375	0	2
1767	22	2.75	0	2
1768	13	1.625	0	2
1769	15	1.875	0	2
1770	7	0.875	0	2
1771	12	1.5	0	2

1772	19	2.375	0	2
1773	12	1.5	0	2
1774	24	3	0	2
1775	19	2.375	0	2
1776	15	1.91666666666667	0	2
1777	16	2	0	2
1778	21	2.625	0	2
1779	20	2.5	0	2
1780	23	2.875	0	2
1781	9	1.125	0	2
1782	12	1.5	0	2
1783	34	4.25	0	0
1784	21	2.66666666666667	0	2
1785	15	1.875	0	2
1786	15	1.91666666666667	0	2
1787	24	3	0	2
1788	26	3.25	0	2
1789	29	3.625	0	2
1790	25	3.125	0	2
1791	25	3.125	0	2
1792	22	2.75	0	2
1793	23	2.875	0	2
1794	26	3.25	0	2
1795	11	1.375	0	2
1796	8	1	0	2
1797	14	1.75	0	2
1798	13	1.625	0	2
1799	18	2.25	0	2
1800	21	2.625	0	2
1801	14	1.75	0	2
1802	27	3.375	0	2
1803	22	2.75	0	2
1804	11	1.375	0	2
1805	9	1.125	0	2
1806	13	1.625	0	2
1807	8	1	0	2
1808	16	2	0	2
1809	9	1.125	0	2
1810	15	1.875	0	2
1811	7	0.875	0	2
1812	4	0.5	0	2
1813	5	0.625	0	2
1814	5	0.625	0	2
1815	7	0.875	0	2
1816	7	0.875	0	2
1817	7	1.16666666666667	0	2
1818	4	0.666666666666667	0	2
1819	6	1	0	2
1820	3	0.5	0	2
1821	2	0.333333333333333	0	2
1822	1	0.166666666666667	0	4
1823	3	0.5	0	2
1824	1	0.166666666666667	0	4
1826	1	0.166666666666667	0	4
1827	3	0.5	0	2
1828	3	0.5	0	2
1829	5	0.833333333333333	0	2
1830	5	0.833333333333333	0	2

1831	5	0.833333333333333	0	2
1832	2	0.333333333333333	0	2
1833	3	0.5	0	2
1834	1	0.166666666666667	0	4
1835	4	0.666666666666667	0	2
1836	1	0.166666666666667	0	4
1837	3	0.5	0	2
1838	6	1	0	2
1839	6	1	0	2
1843	2	0.333333333333333	0	2
1844	2	0.333333333333333	0	2
1845	5	0.833333333333333	0	2
1846	2	0.333333333333333	0	2
1847	2	0.333333333333333	0	2
1848	3	0.5	0	2
1849	2	0.333333333333333	0	2
1850	4	0.666666666666667	0	2
1851	7	1.16666666666667	0	2
1852	5	0.833333333333333	0	2
1853	1	0.166666666666667	0	4
1854	1	0.125	0	4
1855	2	0.25	0	2
1857	2	0.25	0	2
1858	3	0.375	0	2
1859	2	0.25	0	2
1860	1	0.125	0	4
1861	1	0.125	0	4
1862	1	0.125	0	4
1863	2	0.25	0	2
1864	1	0.125	0	4
1865	1	0.125	0	4
1866	1	0.125	0	4
1867	2	0.25	0	2
1868	5	0.625	0	2
1869	2	0.25	0	2
1870	2	0.25	0	2
1871	3	0.375	0	2
1873	1	0.125	0	4
1874	2	0.25	0	2
1875	2	0.25	0	2
1876	1	0.125	0	4
1877	2	0.25	0	2
1878	1	0.125	0	4
1879	1	0.125	0	4
1880	1	0.125	0	4
1882	2	0.25	0	2
1883	2	0.25	0	2
1884	1	0.125	0	4
1885	3	0.375	0	2
1886	2	0.25	0	2
1887	1	0.125	0	4
1888	7	0.875	0	2
1889	2	0.25	0	2
1890	4	0.5	0	2
1891	3	0.375	0	2
1892	2	0.25	0	2
1894	1	0.125	0	4
1895	2	0.25	0	2

1896	5	0.625	0	2
1897	2	0.25	0	2
1898	3	0.375	0	2
1899	5	0.625	0	2
1900	2	0.25	0	2
1901	3	0.416666666666667	0	2
1902	1	0.125	0	4
1904	2	0.25	0	2
1905	4	0.5	0	2
1906	5	0.666666666666667	0	2
1907	3	0.375	0	2
1908	9	1.125	0	2
1909	10	1.29166666666667	0	2
1910	15	2.45833333333333	0	2
1911	18	2.29166666666667	0	2
1912	16	2	0	2
1913	5	0.708333333333333	0	2
1914	10	1.5	0	2
1915	8	1.20833333333333	0	2
1916	3	0.5	0	2
1917	4	0.666666666666667	0	2
1918	1	0.166666666666667	0	4
1919	3	0.5	0	2
1920	1	0.166666666666667	0	4
1921	2	0.366666666666667	0	2
1922	2	0.4	0	2
1923	3	0.6	0	2
1924	1	0.2	0	4
1925	1	0.142857142857143	0	4
1926	1	0.142857142857143	0	4
1927	1	0.142857142857143	0	4
1930	4	0.571428571428571	0	2
1931	13	2.08571428571429	0	2
1932	3	0.428571428571429	0	2
1933	4	0.571428571428571	0	2
1935	1	0.2	0	4
1936	3	0.6	0	2
1937	4	0.8	0	2
1938	6	1.2	0	2
1939	2	0.4	0	2
1940	1	0.2	0	4
1941	2	0.4	0	2
1942	4	0.8	0	2
1943	3	0.6	0	2
1944	4	0.8	0	2
1945	2	0.4	0	2
1946	3	0.6	0	2
1947	4	0.8	0	2
1948	2	0.4	0	2
1949	2	0.342857142857143	0	2
1950	1	0.2	0	4
1951	1	0.142857142857143	0	4
1952	2	0.285714285714286	0	2
1953	4	0.571428571428571	0	2
1954	2	0.25	0	2
1958	2	0.25	0	2
1959	1	0.125	0	4
1960	1	0.125	0	4

1961	4	0.5	0	2
1962	4	0.5	0	2
1963	1	0.125	0	4
1966	2	0.25	0	2
1969	2	0.333333333333333	0	2
1970	1	0.125	0	4
1971	1	0.125	0	4
1973	1	0.125	0	4
1979	4	0.5	0	2
1981	5	0.625	0	2
1983	11	1.45833333333333	0	2
1984	2	0.25	0	2
1987	3	0.375	0	2
1988	1	0.125	0	4
1989	4	0.5	0	2
1990	1	0.125	0	4
1991	1	0.125	0	4
1992	2	0.25	0	2
1994	1	0.125	0	4
1995	2	0.25	0	2
1996	2	0.291666666666667	0	2
1997	2	0.25	0	2
1998	1	0.125	0	4
2003	1	0.125	0	4
2006	1	0.125	0	4
2011	1	0.125	0	4
2014	2	0.333333333333333	0	2
2032	1	0.166666666666667	0	4
2033	1	0.166666666666667	0	4
2034	1	0.166666666666667	0	4
2035	1	0.166666666666667	0	4
2036	1	0.166666666666667	0	4
2037	1	0.166666666666667	0	4
2038	1	0.166666666666667	0	4
2042	2	0.333333333333333	0	2
2046	1	0.166666666666667	0	4
2047	3	0.5	0	2
2058	1	0.166666666666667	0	4
2062	1	0.166666666666667	0	4
2063	1	0.166666666666667	0	4
2064	1	0.166666666666667	0	4
2065	1	0.166666666666667	0	4
2066	2	0.333333333333333	0	2
2067	1	0.166666666666667	0	4
2072	2	0.333333333333333	0	2
2079	1	0.166666666666667	0	4
2094	1	0.166666666666667	0	4
2096	1	0.166666666666667	0	4
2097	2	0.333333333333333	0	2
2100	1	0.166666666666667	0	4
2102	1	0.166666666666667	0	4
2105	1	0.166666666666667	0	4
2107	1	0.166666666666667	0	4
2109	2	0.333333333333333	0	2
2110	1	0.166666666666667	0	4
2112	1	0.166666666666667	0	4
2114	1	0.166666666666667	0	4
2115	2	0.333333333333333	0	2

2117	1	0.166666666666667	0	4	
2118	1	0.166666666666667	0	4	
2124	1	0.166666666666667	0	4	
2128	1	0.166666666666667	0	4	
2131	1	0.166666666666667	0	4	
2135	1	0.166666666666667	0	4	
2139	1	0.166666666666667	0	4	
2159	1	0.166666666666667	0	4	
2160	1	0.166666666666667	0	4	
2161	1	0.166666666666667	0	4	
2204	1	0.166666666666667	0	4	
2206	1	0.166666666666667	0	4	
2215	1	0.166666666666667	0	4	
2218	2	0.333333333333333	0	2	
2223	1	0.166666666666667	0	4	
2240	1	0.166666666666667	0	4	
2242	1	0.166666666666667	0	4	
2247	2	0.333333333333333	0	2	
2248	1	0.166666666666667	0	4	
2256	2	0.333333333333333	0	2	
2258	1	0.166666666666667	0	4	
2259	2	0.333333333333333	0	2	
2260	1	0.166666666666667	0	4	
2261	2	0.333333333333333	0	2	
2263	1	0.166666666666667	0	4	
2265	1	0.166666666666667	0	4	
2266	3	0.5	0	2	
2270	1	0.166666666666667	0	4	
2273	3	0.5	0	2	
2275	1	0.166666666666667	0	4	
2276	2	0.333333333333333	0	2	
2277	1	0.166666666666667	0	4	
2280	2	0.333333333333333	0	2	
2281	2	0.333333333333333	0	2	
2282	2	0.333333333333333	0	2	
2287	2	0.333333333333333	0	2	
2288	1	0.166666666666667	0	4	
2289	1	0.166666666666667	0	4	
2290	2	0.333333333333333	0	2	
2292	1	0.166666666666667	0	4	
2293	5	0.833333333333333	0	2	
2295	2	0.333333333333333	0	2	
2296	2	0.333333333333333	0	2	
2297	1	0.166666666666667	0	4	
2298	4	0.666666666666667	0	2	
2299	1	0.166666666666667	0	4	
2300	3	0.5	0	2	
2304	2	0.333333333333333	0	2	<<<
2308	2	0.333333333333333	0	2	

pto-miR042c,d

category=2, cleavage_site=2304
query=pto-miR042c,d, target=Potri.007G026400.1,
score=4, range=2292-2313, strand=1

```
target  5' CAGUAAGUGAAUgCUGAUUCAU 3'
           : ..:::::::::::::.:::
query   3' GAUGUUCACUUACGACUGAGUC 5'
```

>Potri.007G026400.1
#size=2371

138	1	0.2	0	4
158	1	0.2	0	4
169	1	0.2	0	4
178	1	0.2	0	4
186	1	0.2	0	4
201	1	0.2	0	4
203	1	0.2	0	4
209	2	0.4	0	2
221	1	0.2	0	4
223	1	0.2	0	4
245	1	0.2	0	4
252	1	0.2	0	4
257	1	0.2	0	4
282	1	0.2	0	4
287	1	0.2	0	4
289	1	0.2	0	4
295	1	0.166666666666667	0	4
296	3	0.5	0	2
297	1	0.166666666666667	0	4
309	1	0.125	0	4
310	1	0.125	0	4
312	1	0.125	0	4
314	4	0.5	0	2
315	2	0.25	0	2
316	1	0.125	0	4
317	1	0.125	0	4
318	7	0.875	0	2
320	2	0.25	0	2
321	1	0.125	0	4
322	1	0.125	0	4
323	1	0.125	0	4
326	2	0.25	0	2
327	1	0.125	0	4
328	2	0.25	0	2
330	1	0.166666666666667	0	4
332	5	0.625	0	2
333	1	0.125	0	4
335	2	0.25	0	2
337	2	0.333333333333333	0	2
342	1	0.166666666666667	0	4
345	1	0.166666666666667	0	4
350	2	0.25	0	2
357	1	0.125	0	4
363	1	0.125	0	4
370	1	0.125	0	4
375	1	0.125	0	4
379	2	0.25	0	2
380	1	0.125	0	4
382	1	0.125	0	4
385	1	0.125	0	4
394	2	0.25	0	2
428	1	0.166666666666667	0	4
435	2	0.333333333333333	0	2
436	1	0.125	0	4
441	1	0.125	0	4
445	1	0.125	0	4

448	1	0.125	0	4
451	1	0.125	0	4
453	1	0.125	0	4
465	1	0.166666666666667	0	4
470	1	0.166666666666667	0	4
478	1	0.166666666666667	0	4
484	1	0.125	0	4
489	1	0.166666666666667	0	4
490	1	0.166666666666667	0	4
492	1	0.166666666666667	0	4
496	1	0.166666666666667	0	4
500	1	0.125	0	4
501	1	0.125	0	4
502	1	0.125	0	4
503	1	0.125	0	4
505	2	0.25	0	2
507	1	0.125	0	4
512	1	0.125	0	4
517	1	0.125	0	4
519	1	0.125	0	4
521	1	0.125	0	4
522	1	0.125	0	4
525	1	0.125	0	4
539	1	0.125	0	4
545	1	0.125	0	4
557	1	0.125	0	4
561	2	0.25	0	2
569	1	0.125	0	4
570	1	0.125	0	4
571	2	0.25	0	2
575	1	0.125	0	4
585	1	0.125	0	4
597	1	0.166666666666667	0	4
599	1	0.166666666666667	0	4
603	1	0.166666666666667	0	4
617	1	0.166666666666667	0	4
621	1	0.125	0	4
629	1	0.125	0	4
637	1	0.125	0	4
639	1	0.125	0	4
643	2	0.25	0	2
648	1	0.125	0	4
649	3	0.375	0	2
652	1	0.125	0	4
655	1	0.125	0	4
658	1	0.125	0	4
659	1	0.166666666666667	0	4
670	2	0.333333333333333	0	2
680	1	0.166666666666667	0	4
682	1	0.125	0	4
683	1	0.166666666666667	0	4
688	1	0.125	0	4
691	1	0.125	0	4
695	1	0.125	0	4
699	3	0.375	0	2
704	1	0.166666666666667	0	4
707	1	0.166666666666667	0	4
708	1	0.166666666666667	0	4

713	1	0.125	0	4
733	1	0.125	0	4
735	1	0.125	0	4
751	1	0.125	0	4
757	1	0.166666666666667	0	4
759	1	0.166666666666667	0	4
762	1	0.166666666666667	0	4
764	1	0.166666666666667	0	4
767	2	0.333333333333333	0	2
783	2	0.333333333333333	0	2
787	1	0.166666666666667	0	4
791	1	0.166666666666667	0	4
797	2	0.333333333333333	0	2
808	1	0.166666666666667	0	4
810	1	0.125	0	4
811	1	0.166666666666667	0	4
813	1	0.125	0	4
818	1	0.166666666666667	0	4
820	1	0.125	0	4
823	1	0.166666666666667	0	4
826	2	0.25	0	2
834	1	0.125	0	4
849	1	0.125	0	4
850	2	0.25	0	2
859	1	0.125	0	4
865	2	0.25	0	2
867	1	0.125	0	4
869	1	0.166666666666667	0	4
870	1	0.166666666666667	0	4
878	1	0.166666666666667	0	4
883	2	0.333333333333333	0	2
899	2	0.333333333333333	0	2
911	1	0.166666666666667	0	4
919	1	0.166666666666667	0	4
920	1	0.166666666666667	0	4
923	1	0.166666666666667	0	4
927	1	0.166666666666667	0	4
929	1	0.166666666666667	0	4
938	1	0.166666666666667	0	4
940	1	0.125	0	4
943	1	0.125	0	4
944	1	0.125	0	4
945	1	0.125	0	4
948	1	0.125	0	4
949	1	0.125	0	4
951	2	0.25	0	2
952	1	0.125	0	4
955	1	0.125	0	4
956	1	0.125	0	4
957	1	0.125	0	4
960	1	0.125	0	4
961	1	0.125	0	4
964	1	0.125	0	4
973	1	0.166666666666667	0	4
977	1	0.125	0	4
984	1	0.125	0	4
990	1	0.166666666666667	0	4
1005	1	0.166666666666667	0	4

1010	1	0.125	0	4
1013	1	0.125	0	4
1014	2	0.25	0	2
1024	1	0.125	0	4
1026	1	0.166666666666667	0	4
1030	1	0.166666666666667	0	4
1040	1	0.166666666666667	0	4
1049	1	0.166666666666667	0	4
1056	1	0.166666666666667	0	4
1060	1	0.166666666666667	0	4
1063	1	0.125	0	4
1066	3	0.375	0	2
1067	1	0.166666666666667	0	4
1070	2	0.333333333333333	0	2
1073	1	0.125	0	4
1076	1	0.125	0	4
1083	2	0.333333333333333	0	2
1085	1	0.166666666666667	0	4
1087	2	0.333333333333333	0	2
1088	4	0.666666666666667	0	2
1089	1	0.166666666666667	0	4
1092	2	0.333333333333333	0	2
1097	1	0.166666666666667	0	4
1099	1	0.166666666666667	0	4
1100	2	0.333333333333333	0	2
1103	1	0.125	0	4
1104	1	0.125	0	4
1105	1	0.125	0	4
1106	1	0.125	0	4
1107	1	0.125	0	4
1108	1	0.125	0	4
1112	1	0.125	0	4
1114	1	0.125	0	4
1116	1	0.125	0	4
1118	2	0.25	0	2
1119	1	0.125	0	4
1122	1	0.125	0	4
1130	2	0.25	0	2
1131	1	0.125	0	4
1133	1	0.125	0	4
1134	1	0.125	0	4
1135	2	0.25	0	2
1138	1	0.125	0	4
1141	1	0.125	0	4
1146	1	0.125	0	4
1153	1	0.125	0	4
1163	2	0.25	0	2
1176	1	0.125	0	4
1177	1	0.125	0	4
1181	1	0.125	0	4
1190	1	0.125	0	4
1192	2	0.25	0	2
1195	1	0.125	0	4
1196	1	0.125	0	4
1202	5	0.625	0	2
1206	1	0.125	0	4
1211	3	0.5	0	2
1213	2	0.333333333333333	0	2

1214	1	0.125	0	4
1215	1	0.125	0	4
1216	1	0.125	0	4
1217	1	0.125	0	4
1219	3	0.375	0	2
1220	2	0.25	0	2
1224	1	0.125	0	4
1225	1	0.125	0	4
1226	1	0.125	0	4
1229	1	0.125	0	4
1232	2	0.25	0	2
1233	1	0.125	0	4
1234	2	0.25	0	2
1235	1	0.125	0	4
1238	3	0.375	0	2
1239	1	0.125	0	4
1241	2	0.25	0	2
1243	1	0.125	0	4
1248	2	0.25	0	2
1251	1	0.125	0	4
1253	2	0.25	0	2
1256	1	0.125	0	4
1257	1	0.125	0	4
1259	1	0.125	0	4
1262	1	0.125	0	4
1263	2	0.25	0	2
1266	1	0.125	0	4
1286	2	0.25	0	2
1299	1	0.125	0	4
1310	1	0.125	0	4
1314	1	0.125	0	4
1336	1	0.166666666666667	0	4
1337	1	0.166666666666667	0	4
1338	1	0.166666666666667	0	4
1343	1	0.166666666666667	0	4
1345	1	0.166666666666667	0	4
1346	1	0.166666666666667	0	4
1348	2	0.333333333333333	0	2
1351	1	0.166666666666667	0	4
1357	1	0.166666666666667	0	4
1360	1	0.166666666666667	0	4
1361	1	0.166666666666667	0	4
1366	1	0.166666666666667	0	4
1370	1	0.166666666666667	0	4
1373	1	0.166666666666667	0	4
1375	1	0.166666666666667	0	4
1376	1	0.166666666666667	0	4
1378	1	0.166666666666667	0	4
1384	1	0.166666666666667	0	4
1391	1	0.166666666666667	0	4
1392	1	0.166666666666667	0	4
1397	1	0.125	0	4
1400	1	0.166666666666667	0	4
1401	1	0.166666666666667	0	4
1403	2	0.333333333333333	0	2
1406	1	0.166666666666667	0	4
1408	1	0.166666666666667	0	4
1411	1	0.166666666666667	0	4

1412	1	0.166666666666667	0	4
1414	2	0.333333333333333	0	2
1418	1	0.166666666666667	0	4
1420	1	0.166666666666667	0	4
1426	1	0.166666666666667	0	4
1429	1	0.166666666666667	0	4
1430	2	0.333333333333333	0	2
1432	1	0.166666666666667	0	4
1436	1	0.166666666666667	0	4
1438	1	0.166666666666667	0	4
1442	1	0.166666666666667	0	4
1444	1	0.166666666666667	0	4
1446	1	0.166666666666667	0	4
1448	1	0.166666666666667	0	4
1450	1	0.166666666666667	0	4
1455	1	0.125	0	4
1479	1	0.125	0	4
1491	1	0.125	0	4
1495	1	0.125	0	4
1500	1	0.125	0	4
1511	1	0.125	0	4
1513	1	0.125	0	4
1514	1	0.125	0	4
1515	3	0.375	0	2
1516	1	0.125	0	4
1518	1	0.125	0	4
1523	1	0.125	0	4
1525	3	0.375	0	2
1526	1	0.125	0	4
1527	2	0.25	0	2
1528	1	0.125	0	4
1529	1	0.125	0	4
1530	1	0.125	0	4
1533	2	0.25	0	2
1534	1	0.125	0	4
1537	2	0.25	0	2
1539	3	0.416666666666667	0	2
1541	1	0.166666666666667	0	4
1545	1	0.166666666666667	0	4
1546	2	0.333333333333333	0	2
1547	1	0.166666666666667	0	4
1548	2	0.333333333333333	0	2
1550	1	0.166666666666667	0	4
1551	1	0.166666666666667	0	4
1554	3	0.5	0	2
1558	2	0.333333333333333	0	2
1560	1	0.2	0	4
1561	2	0.4	0	2
1563	1	0.2	0	4
1565	2	0.4	0	2
1566	1	0.2	0	4
1567	2	0.4	0	2
1568	1	0.2	0	4
1569	3	0.6	0	2
1570	3	0.6	0	2
1572	1	0.2	0	4
1573	2	0.4	0	2
1574	1	0.2	0	4

1575	2	0.333333333333333	0	2
1576	1	0.166666666666667	0	4
1577	2	0.333333333333333	0	2
1578	3	0.5	0	2
1579	1	0.166666666666667	0	4
1580	2	0.333333333333333	0	2
1581	2	0.333333333333333	0	2
1582	5	0.833333333333333	0	2
1587	2	0.333333333333333	0	2
1589	1	0.166666666666667	0	4
1590	1	0.166666666666667	0	4
1592	1	0.166666666666667	0	4
1594	1	0.142857142857143	0	4
1595	1	0.2	0	4
1597	2	0.285714285714286	0	2
1599	2	0.291666666666667	0	2
1600	3	0.375	0	2
1601	1	0.166666666666667	0	4
1603	3	0.375	0	2
1605	1	0.166666666666667	0	4
1606	1	0.166666666666667	0	4
1607	1	0.166666666666667	0	4
1609	3	0.5	0	2
1610	1	0.166666666666667	0	4
1611	6	1	0	2
1612	2	0.333333333333333	0	2
1614	1	0.166666666666667	0	4
1616	2	0.333333333333333	0	2
1617	2	0.333333333333333	0	2
1618	5	0.833333333333333	0	2
1619	4	0.666666666666667	0	2
1620	6	1	0	2
1621	3	0.5	0	2
1622	3	0.5	0	2
1623	3	0.5	0	2
1624	3	0.5	0	2
1625	1	0.125	0	4
1626	2	0.25	0	2
1627	2	0.333333333333333	0	2
1628	6	0.916666666666667	0	2
1629	6	0.875	0	2
1630	4	0.5	0	2
1631	1	0.125	0	4
1632	3	0.375	0	2
1636	1	0.125	0	4
1640	2	0.25	0	2
1642	1	0.125	0	4
1643	2	0.25	0	2
1647	1	0.125	0	4
1648	1	0.125	0	4
1649	2	0.25	0	2
1650	1	0.125	0	4
1651	1	0.125	0	4
1652	3	0.5	0	2
1653	2	0.333333333333333	0	2
1654	2	0.333333333333333	0	2
1655	4	0.625	0	2
1658	1	0.166666666666667	0	4

1661	2	0.333333333333333	0	2
1662	1	0.166666666666667	0	4
1663	4	0.666666666666667	0	2
1664	1	0.166666666666667	0	4
1666	1	0.166666666666667	0	4
1667	1	0.166666666666667	0	4
1668	1	0.166666666666667	0	4
1673	1	0.166666666666667	0	4
1674	1	0.125	0	4
1676	1	0.166666666666667	0	4
1677	5	0.666666666666667	0	2
1678	1	0.125	0	4
1680	4	0.5	0	2
1681	2	0.25	0	2
1682	3	0.375	0	2
1683	5	0.625	0	2
1684	3	0.5	0	2
1685	1	0.166666666666667	0	4
1686	1	0.166666666666667	0	4
1689	2	0.25	0	2
1690	1	0.125	0	4
1692	4	0.5	0	2
1693	3	0.375	0	2
1694	2	0.333333333333333	0	2
1696	2	0.333333333333333	0	2
1697	1	0.166666666666667	0	4
1698	2	0.333333333333333	0	2
1701	1	0.166666666666667	0	4
1702	5	0.833333333333333	0	2
1703	1	0.166666666666667	0	4
1704	2	0.333333333333333	0	2
1705	3	0.5	0	2
1707	2	0.333333333333333	0	2
1708	1	0.166666666666667	0	4
1709	3	0.5	0	2
1710	2	0.333333333333333	0	2
1711	2	0.333333333333333	0	2
1712	2	0.333333333333333	0	2
1713	1	0.166666666666667	0	4
1714	4	0.666666666666667	0	2
1715	5	0.833333333333333	0	2
1716	1	0.166666666666667	0	4
1717	1	0.166666666666667	0	4
1718	8	1.33333333333333	0	2
1720	3	0.5	0	2
1721	2	0.333333333333333	0	2
1722	5	0.833333333333333	0	2
1723	1	0.166666666666667	0	4
1724	3	0.5	0	2
1725	4	0.666666666666667	0	2
1726	9	1.5	0	2
1727	6	1	0	2
1728	8	1.33333333333333	0	2
1729	5	0.833333333333333	0	2
1730	12	2	0	2
1731	3	0.5	0	2
1732	10	1.66666666666667	0	2
1733	5	0.833333333333333	0	2

1734	3	0.5	0	2
1735	7	1.16666666666667	0	2
1736	1	0.166666666666667	0	4
1737	5	0.625	0	2
1738	3	0.458333333333333	0	2
1739	7	1.04166666666667	0	2
1740	12	1.54166666666667	0	2
1741	21	2.95833333333333	0	2
1742	10	1.25	0	2
1743	13	1.625	0	2
1744	8	1	0	2
1745	4	0.541666666666667	0	2
1746	6	0.791666666666667	0	2
1747	6	0.875	0	2
1748	6	0.791666666666667	0	2
1749	13	1.66666666666667	0	2
1750	13	1.79166666666667	0	2
1751	12	1.625	0	2
1752	12	1.66666666666667	0	2
1753	2	0.291666666666667	0	2
1754	8	1.08333333333333	0	2
1755	12	1.58333333333333	0	2
1756	14	2	0	2
1757	6	0.875	0	2
1758	12	1.875	0	2
1759	8	1.125	0	2
1760	8	1	0	2
1761	15	2.08333333333333	0	2
1762	15	2	0	2
1763	11	1.375	0	2
1764	14	1.75	0	2
1765	18	2.29166666666667	0	2
1766	19	2.375	0	2
1767	22	2.75	0	2
1768	13	1.625	0	2
1769	15	1.875	0	2
1770	7	0.875	0	2
1771	12	1.5	0	2
1772	19	2.375	0	2
1773	12	1.5	0	2
1774	24	3	0	2
1775	19	2.375	0	2
1776	15	1.91666666666667	0	2
1777	16	2	0	2
1778	21	2.625	0	2
1779	20	2.5	0	2
1780	23	2.875	0	2
1781	9	1.125	0	2
1782	12	1.5	0	2
1783	34	4.25	0	0
1784	21	2.66666666666667	0	2
1785	15	1.875	0	2
1786	15	1.91666666666667	0	2
1787	24	3	0	2
1788	26	3.25	0	2
1789	29	3.625	0	2
1790	25	3.125	0	2
1791	25	3.125	0	2

1792	22	2.75	0	2
1793	23	2.875	0	2
1794	26	3.25	0	2
1795	11	1.375	0	2
1796	8	1	0	2
1797	14	1.75	0	2
1798	13	1.625	0	2
1799	18	2.25	0	2
1800	21	2.625	0	2
1801	14	1.75	0	2
1802	27	3.375	0	2
1803	22	2.75	0	2
1804	11	1.375	0	2
1805	9	1.125	0	2
1806	13	1.625	0	2
1807	8	1	0	2
1808	16	2	0	2
1809	9	1.125	0	2
1810	15	1.875	0	2
1811	7	0.875	0	2
1812	4	0.5	0	2
1813	5	0.625	0	2
1814	5	0.625	0	2
1815	7	0.875	0	2
1816	7	0.875	0	2
1817	7	1.16666666666667	0	2
1818	4	0.666666666666667	0	2
1819	6	1	0	2
1820	3	0.5	0	2
1821	2	0.333333333333333	0	2
1822	1	0.166666666666667	0	4
1823	3	0.5	0	2
1824	1	0.166666666666667	0	4
1826	1	0.166666666666667	0	4
1827	3	0.5	0	2
1828	3	0.5	0	2
1829	5	0.833333333333333	0	2
1830	5	0.833333333333333	0	2
1831	5	0.833333333333333	0	2
1832	2	0.333333333333333	0	2
1833	3	0.5	0	2
1834	1	0.166666666666667	0	4
1835	4	0.666666666666667	0	2
1836	1	0.166666666666667	0	4
1837	3	0.5	0	2
1838	6	1	0	2
1839	6	1	0	2
1843	2	0.333333333333333	0	2
1844	2	0.333333333333333	0	2
1845	5	0.833333333333333	0	2
1846	2	0.333333333333333	0	2
1847	2	0.333333333333333	0	2
1848	3	0.5	0	2
1849	2	0.333333333333333	0	2
1850	4	0.666666666666667	0	2
1851	7	1.16666666666667	0	2
1852	5	0.833333333333333	0	2
1853	1	0.166666666666667	0	4

1854	1	0.125	0	4
1855	2	0.25	0	2
1857	2	0.25	0	2
1858	3	0.375	0	2
1859	2	0.25	0	2
1860	1	0.125	0	4
1861	1	0.125	0	4
1862	1	0.125	0	4
1863	2	0.25	0	2
1864	1	0.125	0	4
1865	1	0.125	0	4
1866	1	0.125	0	4
1867	2	0.25	0	2
1868	5	0.625	0	2
1869	2	0.25	0	2
1870	2	0.25	0	2
1871	3	0.375	0	2
1873	1	0.125	0	4
1874	2	0.25	0	2
1875	2	0.25	0	2
1876	1	0.125	0	4
1877	2	0.25	0	2
1878	1	0.125	0	4
1879	1	0.125	0	4
1880	1	0.125	0	4
1882	2	0.25	0	2
1883	2	0.25	0	2
1884	1	0.125	0	4
1885	3	0.375	0	2
1886	2	0.25	0	2
1887	1	0.125	0	4
1888	7	0.875	0	2
1889	2	0.25	0	2
1890	4	0.5	0	2
1891	3	0.375	0	2
1892	2	0.25	0	2
1894	1	0.125	0	4
1895	2	0.25	0	2
1896	5	0.625	0	2
1897	2	0.25	0	2
1898	3	0.375	0	2
1899	5	0.625	0	2
1900	2	0.25	0	2
1901	3	0.416666666666667	0	2
1902	1	0.125	0	4
1904	2	0.25	0	2
1905	4	0.5	0	2
1906	5	0.666666666666667	0	2
1907	3	0.375	0	2
1908	9	1.125	0	2
1909	10	1.29166666666667	0	2
1910	15	2.45833333333333	0	2
1911	18	2.29166666666667	0	2
1912	16	2	0	2
1913	5	0.708333333333333	0	2
1914	10	1.5	0	2
1915	8	1.20833333333333	0	2
1916	3	0.5	0	2

1917	4	0.666666666666667	0	2
1918	1	0.166666666666667	0	4
1919	3	0.5	0	2
1920	1	0.166666666666667	0	4
1921	2	0.366666666666667	0	2
1922	2	0.4	0	2
1923	3	0.6	0	2
1924	1	0.2	0	4
1925	1	0.142857142857143	0	4
1926	1	0.142857142857143	0	4
1927	1	0.142857142857143	0	4
1930	4	0.571428571428571	0	2
1931	13	2.08571428571429	0	2
1932	3	0.428571428571429	0	2
1933	4	0.571428571428571	0	2
1935	1	0.2	0	4
1936	3	0.6	0	2
1937	4	0.8	0	2
1938	6	1.2	0	2
1939	2	0.4	0	2
1940	1	0.2	0	4
1941	2	0.4	0	2
1942	4	0.8	0	2
1943	3	0.6	0	2
1944	4	0.8	0	2
1945	2	0.4	0	2
1946	3	0.6	0	2
1947	4	0.8	0	2
1948	2	0.4	0	2
1949	2	0.342857142857143	0	2
1950	1	0.2	0	4
1951	1	0.142857142857143	0	4
1952	2	0.285714285714286	0	2
1953	4	0.571428571428571	0	2
1954	2	0.25	0	2
1958	2	0.25	0	2
1959	1	0.125	0	4
1960	1	0.125	0	4
1961	4	0.5	0	2
1962	4	0.5	0	2
1963	1	0.125	0	4
1966	2	0.25	0	2
1969	2	0.333333333333333	0	2
1970	1	0.125	0	4
1971	1	0.125	0	4
1973	1	0.125	0	4
1979	4	0.5	0	2
1981	5	0.625	0	2
1983	11	1.45833333333333	0	2
1984	2	0.25	0	2
1987	3	0.375	0	2
1988	1	0.125	0	4
1989	4	0.5	0	2
1990	1	0.125	0	4
1991	1	0.125	0	4
1992	2	0.25	0	2
1994	1	0.125	0	4
1995	2	0.25	0	2

1996	2	0.291666666666667	0	2
1997	2	0.25	0	2
1998	1	0.125	0	4
2003	1	0.125	0	4
2006	1	0.125	0	4
2011	1	0.125	0	4
2014	2	0.333333333333333	0	2
2032	1	0.166666666666667	0	4
2033	1	0.166666666666667	0	4
2034	1	0.166666666666667	0	4
2035	1	0.166666666666667	0	4
2036	1	0.166666666666667	0	4
2037	1	0.166666666666667	0	4
2038	1	0.166666666666667	0	4
2042	2	0.333333333333333	0	2
2046	1	0.166666666666667	0	4
2047	3	0.5	0	2
2058	1	0.166666666666667	0	4
2062	1	0.166666666666667	0	4
2063	1	0.166666666666667	0	4
2064	1	0.166666666666667	0	4
2065	1	0.166666666666667	0	4
2066	2	0.333333333333333	0	2
2067	1	0.166666666666667	0	4
2072	2	0.333333333333333	0	2
2079	1	0.166666666666667	0	4
2094	1	0.166666666666667	0	4
2096	1	0.166666666666667	0	4
2097	2	0.333333333333333	0	2
2100	1	0.166666666666667	0	4
2102	1	0.166666666666667	0	4
2105	1	0.166666666666667	0	4
2107	1	0.166666666666667	0	4
2109	2	0.333333333333333	0	2
2110	1	0.166666666666667	0	4
2112	1	0.166666666666667	0	4
2114	1	0.166666666666667	0	4
2115	2	0.333333333333333	0	2
2117	1	0.166666666666667	0	4
2118	1	0.166666666666667	0	4
2124	1	0.166666666666667	0	4
2128	1	0.166666666666667	0	4
2131	1	0.166666666666667	0	4
2135	1	0.166666666666667	0	4
2139	1	0.166666666666667	0	4
2159	1	0.166666666666667	0	4
2160	1	0.166666666666667	0	4
2161	1	0.166666666666667	0	4
2204	1	0.166666666666667	0	4
2206	1	0.166666666666667	0	4
2215	1	0.166666666666667	0	4
2218	2	0.333333333333333	0	2
2223	1	0.166666666666667	0	4
2240	1	0.166666666666667	0	4
2242	1	0.166666666666667	0	4
2247	2	0.333333333333333	0	2
2248	1	0.166666666666667	0	4
2256	2	0.333333333333333	0	2

2258	1	0.166666666666667	0	4	
2259	2	0.333333333333333	0	2	
2260	1	0.166666666666667	0	4	
2261	2	0.333333333333333	0	2	
2263	1	0.166666666666667	0	4	
2265	1	0.166666666666667	0	4	
2266	3	0.5	0	2	
2270	1	0.166666666666667	0	4	
2273	3	0.5	0	2	
2275	1	0.166666666666667	0	4	
2276	2	0.333333333333333	0	2	
2277	1	0.166666666666667	0	4	
2280	2	0.333333333333333	0	2	
2281	2	0.333333333333333	0	2	
2282	2	0.333333333333333	0	2	
2287	2	0.333333333333333	0	2	
2288	1	0.166666666666667	0	4	
2289	1	0.166666666666667	0	4	
2290	2	0.333333333333333	0	2	
2292	1	0.166666666666667	0	4	
2293	5	0.833333333333333	0	2	
2295	2	0.333333333333333	0	2	
2296	2	0.333333333333333	0	2	
2297	1	0.166666666666667	0	4	
2298	4	0.666666666666667	0	2	
2299	1	0.166666666666667	0	4	
2300	3	0.5	0	2	
2304	2	0.333333333333333	0	2	<<<
2308	2	0.333333333333333	0	2	

pto-miR043a-c

---

category=4, cleavage_site=662
query=pto-miR043a-c, target=Potri.003G013300.1,
score=4, range=651-671, strand=1

```
target  5' AGUCCCAUGAAaAUCAACUGA 3'
              :.:  ::.:::::.:::::::
query   3' UUAUAGUGCUUUUGGUUGACU 5'
```

>Potri.003G013300.1
#size=1027

290	1	0.2	0	4	
339	1	0.166666666666667	0	4	
342	3	0.428571428571429	0	0	
347	1	0.142857142857143	0	4	
348	2	0.333333333333333	0	2	
356	1	0.142857142857143	0	4	
370	1	0.142857142857143	0	4	
392	1	0.111111111111111	0	4	
662	1	0.111111111111111	0	4	<<<
705	1	0.111111111111111	0	4	

---

category=4, cleavage_site=399
query=pto-miR043a-c, target=Potri.003G029100.1,
score=4, range=388-408, strand=1

```
target  5' AGUCCCAUGAAaAUCAACUGA 3'
              :.:  ::.:::::.:::::::
query   3' UUAUAGUGCUUUUGGUUGACU 5'
```

>Potri.003G029100.1
#size=575

399	1	0.111111111111111	0	4	<<<
442	1	0.111111111111111	0	4	

pto-miR047a,b

category=4, cleavage_site=232
query=pto-miR047a,b, target=Potri.015G064000.1,
score=4, range=221-241, strand=1

```
target  5' AACCAC-CUCUCuAUCUCUUCU 3'
           :: ::: :::::::.::::::
query   3' UUAGUGUGAGAGAUGGAGAAGU 5'
```

>Potri.015G064000.1
#size=1096

52	2	2	2	2	
92	2	2	2	2	
93	1	1	1	4	
97	1	1	1	4	
98	1	1	1	4	
99	1	1	1	4	
102	1	1	1	4	
103	2	2	2	2	
108	1	1	1	4	
149	1	1	1	4	
160	1	1	1	4	
170	1	1	1	4	
172	1	1	1	4	
174	2	2	2	2	
178	1	1	1	4	
193	1	1	1	4	
201	1	1	1	4	
203	1	1	1	4	
205	1	1	1	4	
206	1	1	1	4	
207	2	2	2	2	
209	1	1	1	4	
216	2	2	2	2	
225	1	1	1	4	
226	1	1	1	4	
227	1	1	1	4	
229	2	2	2	2	
232	1	1	1	4	<<<
254	1	1	1	4	
266	1	1	1	4	
268	1	1	1	4	
269	1	1	1	4	
270	1	1	1	4	
271	2	2	2	2	
273	1	1	1	4	
276	1	1	1	4	
277	2	2	2	2	
278	1	1	1	4	
281	2	2	2	2	
282	1	1	1	4	
283	1	1	1	4	
284	6	6	6	0	

285	1	1	1	4
287	1	1	1	4
289	1	1	1	4
290	2	2	2	2
291	1	1	1	4
292	3	3	3	2
294	1	1	1	4
296	1	1	1	4
297	2	2	2	2
298	1	1	1	4
299	1	1	1	4
300	1	1	1	4
301	1	1	1	4
302	1	1	1	4
307	1	1	1	4
317	1	1	1	4
319	1	1	1	4
320	1	1	1	4
322	1	1	1	4
323	2	2	2	2
325	4	4	4	2
329	1	1	1	4
330	1	1	1	4
333	1	1	1	4
334	1	1	1	4
336	2	2	2	2
342	1	1	1	4
344	1	1	1	4
346	1	1	1	4
348	1	1	1	4
350	2	2	2	2
355	1	1	1	4
357	1	1	1	4
358	2	2	2	2
362	1	1	1	4
364	1	1	1	4
367	1	1	1	4
368	1	1	1	4
379	1	1	1	4
384	1	1	1	4
386	2	2	2	2
388	1	1	1	4
391	1	1	1	4
395	1	1	1	4
399	1	0.166666666666667	0	4
403	1	1	1	4
417	1	1	1	4
429	1	1	1	4
430	1	1	1	4
431	1	1	1	4
433	1	1	1	4
436	1	1	1	4
438	1	1	1	4
441	1	1	1	4
443	2	2	2	2
444	1	1	1	4
445	1	1	1	4
448	1	1	1	4

454	1	1	1	4	
458	2	2	2	2	
463	2	2	2	2	
465	2	2	2	2	
466	1	0.142857142857143		0	4
467	3	0.428571428571429		0	3
468	3	1.28571428571429		1	2
472	1	0.25	0	4	
473	2	0.5	0	3	
476	2	0.5	0	3	
477	1	0.25	0	4	
478	5	1.16666666666667		0	2
479	1	0.25	0	4	
481	1	0.166666666666667		0	4
482	1	0.166666666666667		0	4
483	2	0.5	0	3	
485	3	0.5	0	3	
486	4	0.666666666666667		0	3
487	2	0.333333333333333		0	3
488	2	0.333333333333333		0	3
489	2	0.333333333333333		0	3
495	1	0.25	0	4	
500	3	3	3	2	
502	1	1	1	4	
505	1	1	1	4	
506	2	2	2	2	
507	1	1	1	4	
509	1	1	1	4	
510	1	1	1	4	
511	2	2	2	2	
513	1	1	1	4	
514	4	4	4	2	
515	1	1	1	4	
517	1	1	1	4	
519	1	1	1	4	
521	3	3	3	2	
522	2	2	2	2	
524	1	1	1	4	
529	2	2	2	2	
532	4	4	4	2	
535	2	2	2	2	
536	2	2	2	2	
537	2	2	2	2	
541	2	2	2	2	
544	1	1	1	4	
545	1	1	1	4	
546	2	2	2	2	
550	1	1	1	4	
551	1	1	1	4	
554	1	1	1	4	
557	1	1	1	4	

pto-miR056a,b

category=0, cleavage_site=103
query=pto-miR056a,b, target=Potri.002G181900.1,
score=3, range=91-112, strand=1

```
target  5' UUAUUUUAGGACgAGGAAUGAA 3'
           ..:::::::: :::::::::
query   3' GGUAAAAUCCUUCUCCUUACUU 5'
```

>Potri.002G181900.1
#size=1283

24	1	0.333333333333333	0	4	
103	10	3.33333333333333	0	0	<<<
202	1	0.333333333333333	0	4	
238	1	0.333333333333333	0	4	
570	1	0.333333333333333	0	4	
623	1	0.333333333333333	0	4	
736	1	0.25	0	4	
754	1	0.142857142857143	0	4	
844	1	0.25	0	4	
845	1	0.25	0	4	
859	1	0.142857142857143	0	4	
871	2	0.5	0	2	
949	1	0.25	0	4	
950	1	0.25	0	4	
964	1	0.142857142857143	0	4	
976	2	0.5	0	2	
1044	1	0.5	0	4	
1048	1	0.5	0	4	
1129	1	0.333333333333333	0	4	
1131	1	0.333333333333333	0	4	
1191	1	0.333333333333333	0	4	
1194	1	0.333333333333333	0	4	
1195	1	0.25	0	4	
1239	1	0.5	0	4	
1242	1	0.333333333333333	0	4	

category=0, cleavage_site=129
query=pto-miR056a,b, target=Potri.002G182400.1,
score=1, range=117-138, strand=1

```
target  5' CUGUUUUAGGAAgAGGAAUGAA 3'
           :..:::::::::::::::::::
query   3' GGUAAAAUCCUUCUCCUUACUU 5'
```

>Potri.002G182400.1
#size=2331

129	20	10	0	0	<<<
136	1	0.5	0	4	
153	1	0.5	0	4	
181	1	0.5	0	4	
265	3	1.5	0	2	
271	1	0.5	0	4	
272	1	0.5	0	4	
307	2	2	2	2	
378	1	0.5	0	4	
415	1	0.5	0	4	
494	1	0.5	0	4	
498	1	0.5	0	4	
548	1	0.5	0	4	

638	1	0.5	0	4
676	1	0.5	0	4
742	1	0.5	0	4
788	1	0.5	0	4
892	1	0.5	0	4
906	1	0.5	0	4
993	1	0.5	0	4
1265	1	0.5	0	4
1383	1	0.5	0	4
1469	3	1.5	0	2
1476	1	0.5	0	4
1477	1	0.5	0	4
1506	1	0.5	0	4
1507	1	0.5	0	4
1509	1	0.5	0	4
1511	1	0.5	0	4
1513	2	1	0	2
1524	1	0.5	0	4
1535	1	0.5	0	4
1543	1	0.5	0	4
1544	2	1	0	2
1737	1	0.5	0	4
1746	1	0.5	0	4
1747	1	0.5	0	4
1769	1	0.5	0	4
1837	1	0.5	0	4
1861	1	0.5	0	4
1864	1	0.5	0	4
1867	1	0.5	0	4
1868	1	0.5	0	4
1870	1	0.5	0	4
1871	1	0.5	0	4
1875	1	0.5	0	4
1882	2	1	0	2
1888	4	2	0	2
1889	2	1	0	2
1890	1	0.5	0	4
1892	1	0.5	0	4
1895	2	1	0	2
1896	4	2	0	2
1897	1	0.5	0	4
1898	3	1.5	0	2
1899	1	0.5	0	4
1901	1	0.5	0	4
1903	1	0.5	0	4
1904	2	1	0	2
1905	1	0.5	0	4
1907	3	1.5	0	2
1908	2	1	0	2
1909	1	0.5	0	4
1910	4	2	0	2
1912	1	0.5	0	4
1956	1	0.5	0	4
1958	1	0.5	0	4
1961	1	0.5	0	4
1967	3	1.5	0	2
1970	1	0.5	0	4
1977	1	0.5	0	4

1978	1	0.5	0	4
2000	1	0.5	0	4
2001	1	0.5	0	4
2053	2	1	0	2
2066	1	0.5	0	4
2076	1	0.5	0	4
2084	1	0.5	0	4
2094	1	0.5	0	4
2098	1	0.5	0	4
2100	1	0.5	0	4
2126	1	0.5	0	4
2130	1	0.5	0	4
2139	1	0.5	0	4
2143	2	1	0	2
2146	1	0.5	0	4
2147	1	0.5	0	4
2150	1	0.5	0	4
2151	1	0.5	0	4
2152	1	0.5	0	4
2153	2	1	0	2
2154	2	1	0	2
2156	2	1	0	2
2157	2	1	0	2
2158	2	1	0	2
2159	1	0.5	0	4
2161	1	0.5	0	4
2167	2	1	0	2
2180	1	0.5	0	4
2182	1	0.5	0	4
2184	1	0.5	0	4
2240	1	0.5	0	4
2248	1	0.5	0	4
2256	1	0.5	0	4

---

category=0, cleavage_site=450
query=pto-miR056a,b, target=Potri.013G079700.1,
score=1, range=438-459, strand=1

```
target  5' CUGUUUUAGGAAgAGGAAUGAA 3'
            :..::::::::::::::::::
query   3' GGUAAAAUCCUUCUCCUUACUU 5'
```

>Potri.013G079700.1
#size=1749

384	1	0.2	0	4	
437	1	0.2	0	4	
450	78	19.45	0	0	<<<
451	1	0.25	0	4	
460	1	0.25	0	4	
480	1	0.166666666666667	0	4	
481	3	0.5	0	2	
485	1	0.166666666666667	0	4	
486	2	0.333333333333333	0	2	
500	1	0.166666666666667	0	4	
501	9	1.5	0	2	
514	1	0.166666666666667	0	4	
517	1	0.166666666666667	0	4	
562	1	0.166666666666667	0	4	
563	2	0.333333333333333	0	2	
564	5	0.833333333333333	0	2	

567	1	0.166666666666667	0	4
568	1	0.166666666666667	0	4
586	3	0.5	0	2
587	1	0.166666666666667	0	4
588	1	0.166666666666667	0	4
596	1	0.166666666666667	0	4
597	1	0.166666666666667	0	4
598	1	0.166666666666667	0	4
607	1	0.166666666666667	0	4
608	2	0.333333333333333	0	2
614	2	0.333333333333333	0	2
618	1	0.166666666666667	0	4
619	1	0.166666666666667	0	4
623	1	0.166666666666667	0	4
624	1	0.166666666666667	0	4
625	1	0.166666666666667	0	4
626	3	0.5	0	2
627	4	0.666666666666667	0	2
628	1	0.166666666666667	0	4
629	4	0.666666666666667	0	2
632	1	0.166666666666667	0	4
635	1	0.166666666666667	0	4
637	2	0.333333333333333	0	2
638	1	0.166666666666667	0	4
639	1	0.166666666666667	0	4
641	6	1	0	2
642	3	0.5	0	2
644	1	0.166666666666667	0	4
647	3	0.5	0	2
648	2	0.333333333333333	0	2
650	2	0.333333333333333	0	2
651	1	0.166666666666667	0	4
652	1	0.166666666666667	0	4
654	1	0.166666666666667	0	4
657	1	0.166666666666667	0	4
658	2	0.333333333333333	0	2
660	1	0.166666666666667	0	4
670	1	0.125	0	4
673	2	0.25	0	2
674	1	0.125	0	4
675	1	0.125	0	4
688	1	0.125	0	4
707	1	0.125	0	4
724	1	0.125	0	4
730	2	0.225	0	2
748	1	0.125	0	4
757	1	0.125	0	4
761	1	0.125	0	4
764	2	0.25	0	2
765	1	0.125	0	4
767	1	0.125	0	4
770	3	0.375	0	2
773	2	0.25	0	2
774	35	4.375	0	2
775	13	1.625	0	2
776	1	0.125	0	4
777	2	0.25	0	2
778	3	0.375	0	2

779	2	0.25	0	2
780	2	0.25	0	2
782	1	0.125	0	4
783	2	0.25	0	2
784	3	0.375	0	2
786	2	0.25	0	2
787	2	0.25	0	2
788	1	0.125	0	4
790	2	0.25	0	2
791	2	0.25	0	2
796	6	0.75	0	2
797	1	0.125	0	4
811	1	0.125	0	4
814	1	0.125	0	4
817	3	0.375	0	2
818	3	0.375	0	2
820	2	0.25	0	2
824	1	0.125	0	4
827	1	0.125	0	4
828	2	0.25	0	2
847	1	0.125	0	4
851	1	0.125	0	4
852	1	0.125	0	4
854	1	0.125	0	4
859	1	0.125	0	4
867	6	0.75	0	2
871	1	0.125	0	4
872	2	0.25	0	2
874	1	0.125	0	4
882	1	0.125	0	4
883	1	0.125	0	4
900	1	0.142857142857143	0	4
902	1	0.142857142857143	0	4
914	1	0.142857142857143	0	4
917	1	0.142857142857143	0	4
918	1	0.142857142857143	0	4
921	1	0.142857142857143	0	4
922	1	0.142857142857143	0	4
925	1	0.142857142857143	0	4
929	1	0.142857142857143	0	4
931	1	0.142857142857143	0	4
942	2	0.285714285714286	0	2
943	1	0.142857142857143	0	4
944	2	0.285714285714286	0	2
945	1	0.142857142857143	0	4
948	1	0.142857142857143	0	4
953	2	0.285714285714286	0	2
955	1	0.142857142857143	0	4
960	1	0.142857142857143	0	4
969	1	0.142857142857143	0	4
970	1	0.142857142857143	0	4
975	1	0.142857142857143	0	4
976	1	0.142857142857143	0	4
978	1	0.142857142857143	0	4
987	1	0.142857142857143	0	4
998	1	0.142857142857143	0	4
1018	1	0.2	0	4
1042	1	0.2	0	4

1043	1	0.142857142857143	0	4
1047	1	0.142857142857143	0	4
1119	3	0.642857142857143	0	2
1121	1	0.25	0	4
1122	1	0.25	0	4
1125	1	0.25	0	4
1162	1	0.142857142857143	0	4
1177	1	0.142857142857143	0	4
1196	1	0.142857142857143	0	4
1200	1	0.142857142857143	0	4
1210	1	0.142857142857143	0	4
1218	1	0.142857142857143	0	4
1219	1	0.142857142857143	0	4
1231	1	0.142857142857143	0	4
1232	1	0.142857142857143	0	4
1233	3	0.428571428571429	0	2
1237	2	0.285714285714286	0	2
1241	1	0.142857142857143	0	4
1244	1	0.142857142857143	0	4
1247	1	0.142857142857143	0	4
1248	1	0.142857142857143	0	4
1250	1	0.142857142857143	0	4
1251	1	0.142857142857143	0	4
1255	1	0.142857142857143	0	4
1264	2	0.285714285714286	0	2
1266	2	0.285714285714286	0	2
1271	2	0.285714285714286	0	2
1273	1	0.142857142857143	0	4
1339	2	0.285714285714286	0	2
1343	1	0.142857142857143	0	4
1350	1	0.142857142857143	0	4
1353	1	0.142857142857143	0	4
1370	2	0.285714285714286	0	2
1372	1	0.142857142857143	0	4
1396	1	0.142857142857143	0	4
1402	1	0.142857142857143	0	4
1423	1	0.142857142857143	0	4
1474	1	0.142857142857143	0	4
1509	1	0.142857142857143	0	4
1512	1	0.142857142857143	0	4
1532	1	0.142857142857143	0	4
1548	1	0.142857142857143	0	4

category=0, cleavage_site=371
query=pto-miR056a,b, target=Potri.014G071000.1,
score=1, range=359-380, strand=1

```
target  5' CUGUUUUAGGAAgAGGAAUGAA 3'
            :..:::::::::::::::::::
query   3' GGUAAAAUCCUUCUCCUUACUU 5'
```

>Potri.014G071000.1
#size=509

274	1	1	1	4
292	1	1	1	4
298	1	1	1	4
330	1	1	1	4
332	1	1	1	4
338	2	2	2	2
354	1	1	1	4

356 1 1 1 4
363 3 3 3 2
370 1 1 1 4
371 391 391 391 0 <<<
372 3 3 3 2
403 2 2 2 2
465 1 1 1 4

---

category=0, cleavage_site=119
query=pto-miR056a,b, target=Potri.014G108100.1,
score=1.5, range=107-128, strand=1

```
target  5' CUCUUUUAGGAAgAGGAAUGAA 3'
           :. ::::::::::::::::::
query   3' GGUAAAAUCCUUCUCCUUACUU 5'
```

>Potri.014G108100.1
#size=1561
62 1 1 1 4
118 1 1 1 4
119 28 28 28 0 <<<
142 1 1 1 4
145 1 1 1 4
204 1 1 1 4
205 1 1 1 4
441 1 1 1 4
462 1 1 1 4
611 1 1 1 4
621 1 1 1 4
663 1 1 1 4
857 1 0.5 0 4

pto-miR070

---

category=1, cleavage_site=1017
query=pto-miR070, target=Potri.013G029100.1,
score=3, range=1008-1026, strand=1

```
target  5' ACCGUCCAG-cCAUGUUCGU 3'
           : ::::::: ::::::::::
query   3' UCGCAGGUCGGGUACAAGCA 5'
```

>Potri.013G029100.1
#size=2053
1017 2 0.666666666666667 0 1 <<<

pto-miR073

---

category=4, cleavage_site=997
query=pto-miR073, target=Potri.005G237900.1,
score=3.5, range=986-1006, strand=1

```
target  5' GUUCCUGCAACaGCUUCUUCA 3'
           .:: :.. :::::::::::::
query   3' UAAUGGUUUUGUCGAAGAAGU 5'
```

>Potri.005G237900.1
#size=1503
215 1 1 1 4
260 1 1 1 4
263 1 1 1 4
290 1 1 1 4
308 1 1 1 4

322	1	0.25	0	4
332	1	0.25	0	4
337	1	0.25	0	4
341	1	0.25	0	4
360	1	0.25	0	4
425	1	1	1	4
460	1	0.25	0	4
478	1	0.5	0	4
497	1	0.5	0	4
505	1	0.5	0	4
507	1	0.2	0	4
512	1	0.2	0	4
554	1	0.142857142857143	0	4
580	1	0.333333333333333	0	4
581	1	0.333333333333333	0	4
592	1	0.333333333333333	0	4
593	1	0.333333333333333	0	4
595	1	0.333333333333333	0	4
627	1	0.142857142857143	0	4
650	2	0.666666666666667	0	2
657	1	0.333333333333333	0	4
668	1	0.333333333333333	0	4
688	1	0.333333333333333	0	4
689	2	0.666666666666667	0	2
705	2	0.285714285714286	0	3
707	1	0.333333333333333	0	4
728	1	0.333333333333333	0	4
729	1	0.333333333333333	0	4
731	1	0.333333333333333	0	4
765	1	0.333333333333333	0	4
777	1	0.333333333333333	0	4
832	1	0.333333333333333	0	4
833	1	0.333333333333333	0	4
835	1	0.333333333333333	0	4
836	1	0.333333333333333	0	4
854	1	0.333333333333333	0	4
856	1	0.333333333333333	0	4
868	1	0.333333333333333	0	4
873	1	0.333333333333333	0	4
881	1	0.333333333333333	0	4
883	3	1	0	2
887	1	0.333333333333333	0	4
893	1	0.333333333333333	0	4
895	1	0.333333333333333	0	4
911	1	0.333333333333333	0	4
915	1	0.333333333333333	0	4
926	2	0.666666666666667	0	2
936	1	0.333333333333333	0	4
952	1	0.333333333333333	0	4
964	1	0.333333333333333	0	4
970	1	0.333333333333333	0	4
972	1	0.333333333333333	0	4
974	1	0.333333333333333	0	4
977	1	0.333333333333333	0	4
982	2	0.666666666666667	0	2
983	1	0.333333333333333	0	4
984	2	0.666666666666667	0	2
985	1	0.333333333333333	0	4

987	2	0.666666666666667	0	2	
988	2	0.666666666666667	0	2	
990	1	0.333333333333333	0	4	
992	1	0.333333333333333	0	4	
993	1	0.333333333333333	0	4	
997	1	0.333333333333333	0	4	<<<
998	1	0.333333333333333	0	4	
1005	1	0.333333333333333	0	4	
1008	1	0.333333333333333	0	4	
1010	1	0.333333333333333	0	4	
1012	1	0.333333333333333	0	4	
1015	1	0.333333333333333	0	4	
1016	1	0.333333333333333	0	4	
1017	2	0.666666666666667	0	2	
1018	3	1	0	2	
1019	6	2	0	0	
1021	2	0.666666666666667	0	2	
1024	1	0.333333333333333	0	4	
1029	1	0.333333333333333	0	4	
1034	1	0.142857142857143	0	4	
1036	1	0.142857142857143	0	4	
1038	1	0.142857142857143	0	4	
1039	1	0.142857142857143	0	4	
1042	1	0.142857142857143	0	4	
1044	1	0.142857142857143	0	4	
1047	1	0.142857142857143	0	4	
1052	1	0.142857142857143	0	4	
1053	2	0.285714285714286	0	3	
1055	3	1	0	2	
1056	2	0.666666666666667	0	2	
1060	1	0.142857142857143	0	4	
1062	3	1	0	2	
1063	2	0.666666666666667	0	2	
1064	2	0.666666666666667	0	2	
1065	2	0.666666666666667	0	2	
1066	1	0.333333333333333	0	4	
1067	1	0.333333333333333	0	4	
1068	3	1	0	2	
1069	2	0.666666666666667	0	2	
1071	1	0.333333333333333	0	4	
1072	2	0.666666666666667	0	2	
1073	1	0.333333333333333	0	4	
1074	2	0.666666666666667	0	2	
1075	1	0.333333333333333	0	4	
1076	3	1	0	2	
1077	3	1	0	2	
1078	1	0.333333333333333	0	4	
1079	1	0.333333333333333	0	4	
1080	1	0.333333333333333	0	4	
1081	2	0.666666666666667	0	2	
1083	1	0.333333333333333	0	4	
1084	2	0.666666666666667	0	2	
1085	2	0.666666666666667	0	2	
1086	2	0.666666666666667	0	2	
1087	1	0.333333333333333	0	4	
1089	1	0.333333333333333	0	4	
1092	1	0.333333333333333	0	4	
1110	1	0.333333333333333	0	4	

1115	1	0.333333333333333	0	4
1119	2	0.666666666666667	0	2
1124	2	0.666666666666667	0	2
1126	1	0.333333333333333	0	4
1127	1	0.333333333333333	0	4
1136	1	0.333333333333333	0	4
1139	1	0.333333333333333	0	4
1141	1	0.333333333333333	0	4
1143	1	0.333333333333333	0	4
1148	1	0.333333333333333	0	4
1153	1	0.333333333333333	0	4
1154	2	0.666666666666667	0	2
1155	1	0.333333333333333	0	4
1158	1	0.333333333333333	0	4
1161	1	0.333333333333333	0	4
1172	1	0.333333333333333	0	4
1179	2	0.285714285714286	0	3
1181	1	0.142857142857143	0	4
1183	2	0.285714285714286	0	3
1187	1	0.142857142857143	0	4
1188	1	0.142857142857143	0	4
1193	1	0.333333333333333	0	4
1194	3	1	0	2
1197	1	0.142857142857143	0	4
1198	1	0.333333333333333	0	4
1199	1	0.333333333333333	0	4
1205	1	0.333333333333333	0	4
1207	3	1	0	2
1212	1	0.333333333333333	0	4
1218	1	0.333333333333333	0	4
1227	1	0.333333333333333	0	4
1236	2	0.666666666666667	0	2
1265	1	0.333333333333333	0	4
1274	1	0.333333333333333	0	4
1279	1	0.333333333333333	0	4
1283	1	0.333333333333333	0	4
1292	1	0.333333333333333	0	4

---

category=4, cleavage_site=2394
query=pto-miR073, target=Potri.017G143500.1,
score=1.5, range=2383-2403, strand=1

```
target  5' UUUACCAGAACaGCUUCUUCA 3'
           ::::::.::::::::::::::
query   3' UAAUGGUUUUGUCGAAGAAGU 5'
```

>Potri.017G143500.1
#size=2629

92	1	1	1	4
141	1	0.166666666666667	0	4
167	1	0.1	0	4
260	1	0.111111111111111	0	4
262	1	0.111111111111111	0	4
281	1	0.111111111111111	0	4
320	1	0.1	0	4
335	1	0.1	0	4
343	1	0.25	0	4
353	1	0.25	0	4
370	1	0.25	0	4
380	1	0.142857142857143	0	4

445	1	0.166666666666667	0	4
548	1	0.25	0	4
689	1	0.1	0	4
699	1	0.1	0	4
836	1	0.1	0	4
849	1	0.1	0	4
861	1	0.111111111111111	0	4
862	2	0.222222222222222	0	2
950	3	0.3	0	2
951	4	0.4	0	2
957	1	0.1	0	4
960	1	0.1	0	4
961	1	0.1	0	4
978	1	0.1	0	4
979	1	0.1	0	4
998	1	0.1	0	4
1069	1	0.25	0	4
1081	1	0.1	0	4
1085	1	0.1	0	4
1086	1	0.1	0	4
1094	1	0.1	0	4
1095	1	0.1	0	4
1096	1	0.1	0	4
1097	1	0.1	0	4
1098	1	0.1	0	4
1109	1	0.1	0	4
1110	1	0.1	0	4
1183	1	0.1	0	4
1219	1	0.1	0	4
1226	1	0.1	0	4
1236	1	0.1	0	4
1238	1	0.1	0	4
1240	1	0.1	0	4
1245	1	0.1	0	4
1263	1	0.1	0	4
1264	2	0.2	0	2
1268	1	0.1	0	4
1283	1	0.1	0	4
1328	1	0.1	0	4
1337	1	0.111111111111111	0	4
1626	1	0.1	0	4
1637	1	0.1	0	4
1748	1	0.1	0	4
1775	1	0.1	0	4
1799	1	0.1	0	4
1807	1	0.1	0	4
1832	1	0.1	0	4
1888	1	0.166666666666667	0	4
1934	1	0.1	0	4
1949	1	0.1	0	4
1971	2	2	2	0
2000	1	0.1	0	4
2010	1	0.1	0	4
2226	1	0.2	0	4
2374	1	0.5	0	4
2378	1	0.5	0	4
2380	1	0.111111111111111	0	4
2385	1	0.1	0	4

2390	2	0.2	0	2	
2393	2	0.2	0	2	
2394	1	0.1	0	4	<<<
2492	1	0.1	0	4	
2498	1	0.333333333333333	0	4	
2499	1	0.166666666666667	0	4	
2500	1	0.5	0	4	
2502	1	0.166666666666667	0	4	

category=4, cleavage_site=2144
query=pto-miR073, target=Potri.017G143600.1,
score=2.5, range=2133-2153, strand=1

```
target  5' UUUACCAGAACaGUUUCUUCA 3'
             ::::::.::::.:::::::
query   3' UAAUGGUUUUGUCGAAGAAGU 5'
```

>Potri.017G143600.1
#size=2580

64	1	0.1	0	4
101	1	0.1	0	4
109	1	0.1	0	4
128	1	0.1	0	4
157	1	0.111111111111111	0	4
159	1	0.111111111111111	0	4
168	1	0.5	0	4
178	1	0.111111111111111	0	4
249	1	0.333333333333333	0	4
520	1	0.2	0	4
674	2	0.2	0	2
697	3	0.3	0	2
698	4	0.4	0	2
704	1	0.1	0	4
707	1	0.1	0	4
708	1	0.1	0	4
725	2	0.2	0	2
726	1	0.1	0	4
828	1	0.1	0	4
832	1	0.1	0	4
833	1	0.1	0	4
841	1	0.1	0	4
842	1	0.1	0	4
843	1	0.1	0	4
844	1	0.1	0	4
845	1	0.1	0	4
972	1	0.1	0	4
979	1	0.1	0	4
989	1	0.1	0	4
991	1	0.1	0	4
993	1	0.1	0	4
998	1	0.1	0	4
1017	2	0.2	0	2
1021	1	0.1	0	4
1036	1	0.1	0	4
1081	1	0.1	0	4
1226	1	0.1	0	4
1370	1	0.1	0	4
1449	1	0.1	0	4
1492	1	0.1	0	4
1519	1	0.1	0	4

1543	1	0.1	0	4	
1551	1	0.1	0	4	
1576	1	0.1	0	4	
1582	1	0.125	0	4	
1606	1	0.1	0	4	
1619	1	0.1	0	4	
1678	1	0.1	0	4	
1693	1	0.1	0	4	
1826	1	0.1	0	4	
1882	1	0.1	0	4	
1976	1	0.2	0	4	
2023	1	0.1	0	4	
2135	1	0.1	0	4	
2140	2	0.2	0	2	
2143	2	0.4	0	2	
2144	1	0.1	0	4	<<<
2202	1	0.111111111111111	0	4	
2219	1	0.1	0	4	
2224	1	0.1	0	4	
2229	1	0.1	0	4	
2242	1	0.1	0	4	
2249	1	0.166666666666667	0	4	
2250	1	0.333333333333333	0	4	
2252	1	0.166666666666667	0	4	
2278	1	0.2	0	4	
2281	1	0.2	0	4	
2311	1	0.142857142857143	0	4	
2318	1	0.25	0	4	
2336	1	0.25	0	4	
2339	1	0.25	0	4	
2445	1	0.142857142857143	0	4	
2463	1	0.2	0	4	
2494	1	0.25	0	4	

---

category=4, cleavage_site=2226
query=pto-miR073, target=Potri.017G144400.1,
score=3.5, range=2215-2235, strand=1

```
target  5' CUUACCAGAACaGCUUCUACA 3'
             ::::::.:::::::::: ::
query   3' UAAUGGUUUUGUCGAAGAAGU 5'
```

>Potri.017G144400.1
#size=2464

131	1	0.1	0	4
168	1	0.1	0	4
176	1	0.1	0	4
195	1	0.1	0	4
224	1	0.111111111111111	0	4
226	1	0.111111111111111	0	4
245	1	0.111111111111111	0	4
284	1	0.1	0	4
312	1	0.2	0	4
512	1	0.25	0	4
665	1	0.1	0	4
678	1	0.1	0	4
779	3	0.3	0	2
780	4	0.4	0	2
786	1	0.1	0	4
789	1	0.1	0	4

790	1	0.1	0	4	
807	2	0.2	0	2	
808	1	0.1	0	4	
898	1	0.25	0	4	
910	1	0.1	0	4	
914	1	0.1	0	4	
915	1	0.1	0	4	
920	2	2	2	0	
923	1	0.1	0	4	
926	1	0.1	0	4	
938	1	0.1	0	4	
939	1	0.1	0	4	
958	1	0.1	0	4	
959	1	0.1	0	4	
962	1	0.2	0	4	
1098	1	0.1	0	4	
1099	2	0.2	0	2	
1103	1	0.1	0	4	
1118	1	0.1	0	4	
1163	1	0.1	0	4	
1172	1	0.111111111111111	0	4	
1308	1	0.1	0	4	
1452	1	0.1	0	4	
1463	1	0.1	0	4	
1531	1	0.1	0	4	
1574	1	0.1	0	4	
1625	1	0.1	0	4	
1633	1	0.1	0	4	
1688	1	0.1	0	4	
1718	1	0.5	0	4	
1736	1	1	1	4	
1760	1	0.1	0	4	
1775	1	0.1	0	4	
1823	1	0.1	0	4	
1907	1	0.1	0	4	
1964	1	0.1	0	4	
2033	1	0.166666666666667	0	4	
2056	1	0.5	0	4	
2058	1	0.2	0	4	
2139	1	0.25	0	4	
2225	2	0.2	0	2	
2226	1	0.1	0	4	<<<
2228	1	0.1	0	4	
2232	1	0.111111111111111	0	4	
2324	1	0.1	0	4	
2331	1	0.166666666666667	0	4	
2332	1	0.333333333333333	0	4	
2334	1	0.166666666666667	0	4	
2344	1	0.5	0	4	

---

category=4, cleavage_site=700
query=pto-miR073, target=Potri.018G069900.2,
score=3, range=689-709, strand=1

```
target  5' AUUGGAAGAACaGCUUCUUCA 3'
            :::.  :.:::::::::::::
query   3' UAAUGGUUUUGUCGAAGAAGU 5'
```

>Potri.018G069900.2
#size=1214

100	1	0.166666666666667	0	4
101	1	0.166666666666667	0	4
129	1	0.166666666666667	0	4
142	1	0.166666666666667	0	4
145	1	0.166666666666667	0	4
147	1	0.166666666666667	0	4
150	1	0.166666666666667	0	4
152	1	0.166666666666667	0	4
158	1	0.166666666666667	0	4
162	1	0.166666666666667	0	4
167	1	0.166666666666667	0	4
170	2	0.333333333333333	0	2
179	1	0.166666666666667	0	4
189	1	0.2	0	4
193	1	0.2	0	4
194	2	0.4	0	2
208	1	0.2	0	4
220	1	0.166666666666667	0	4
221	1	0.166666666666667	0	4
222	2	0.333333333333333	0	2
228	1	0.166666666666667	0	4
229	2	0.333333333333333	0	2
232	1	0.166666666666667	0	4
236	1	0.166666666666667	0	4
237	1	0.166666666666667	0	4
239	1	0.166666666666667	0	4
242	1	0.166666666666667	0	4
243	1	0.166666666666667	0	4
246	2	0.333333333333333	0	2
252	2	0.333333333333333	0	2
254	1	0.166666666666667	0	4
257	1	0.166666666666667	0	4
258	2	0.2	0	2
259	1	0.1	0	4
260	1	0.1	0	4
261	1	0.1	0	4
262	1	0.1	0	4
263	1	0.1	0	4
266	1	0.1	0	4
270	2	0.2	0	2
274	1	0.1	0	4
307	1	0.166666666666667	0	4
312	1	0.166666666666667	0	4
313	2	0.2	0	2
314	1	0.1	0	4
317	1	0.1	0	4
318	1	0.1	0	4
320	1	0.1	0	4
325	2	0.2	0	2
327	2	0.2	0	2
329	2	0.2	0	2
331	2	0.2	0	2
333	1	0.1	0	4
335	1	0.1	0	4
336	1	0.1	0	4
337	2	0.2	0	2
339	2	0.2	0	2
340	1	0.166666666666667	0	4

346	2	0.333333333333333	0	2
352	1	0.166666666666667	0	4
353	1	0.166666666666667	0	4
354	1	0.166666666666667	0	4
355	2	0.333333333333333	0	2
357	2	0.333333333333333	0	2
358	1	0.166666666666667	0	4
360	2	0.333333333333333	0	2
364	1	0.166666666666667	0	4
365	4	0.666666666666667	0	2
367	1	0.166666666666667	0	4
371	2	0.333333333333333	0	2
373	2	0.333333333333333	0	2
376	1	0.1	0	4
377	1	0.1	0	4
378	1	0.1	0	4
379	2	0.2	0	2
384	1	0.1	0	4
391	1	0.1	0	4
395	1	0.166666666666667	0	4
397	1	0.166666666666667	0	4
399	1	0.166666666666667	0	4
403	1	0.166666666666667	0	4
404	1	0.166666666666667	0	4
405	2	0.333333333333333	0	2
406	1	0.166666666666667	0	4
407	3	0.5	0	2
408	1	0.166666666666667	0	4
409	1	0.166666666666667	0	4
410	4	0.666666666666667	0	2
413	1	0.166666666666667	0	4
415	1	0.166666666666667	0	4
416	2	0.333333333333333	0	2
418	1	0.166666666666667	0	4
423	6	0.6	0	2
424	2	0.2	0	2
426	2	0.2	0	2
427	1	0.1	0	4
428	2	0.2	0	2
429	1	0.1	0	4
430	1	0.1	0	4
431	2	0.2	0	2
432	2	0.2	0	2
434	2	0.2	0	2
435	1	0.1	0	4
437	1	0.1	0	4
438	4	0.4	0	2
440	1	0.1	0	4
441	3	0.3	0	2
442	2	0.2	0	2
443	1	0.1	0	4
444	4	0.4	0	2
445	7	0.7	0	2
446	5	0.5	0	2
447	1	0.1	0	4
448	7	0.7	0	2
449	9	0.9	0	2
450	15	1.5	0	2

451	3	0.3	0	2
452	41	4.1	0	0
453	14	1.4	0	2
454	10	1	0	2
455	7	0.7	0	2
456	13	1.3	0	2
457	16	1.6	0	2
458	13	1.3	0	2
459	32	3.2	0	2
460	28	2.8	0	2
461	7	0.7	0	2
462	6	0.6	0	2
463	6	0.6	0	2
464	10	1	0	2
465	4	0.4	0	2
466	10	1	0	2
467	14	1.4	0	2
468	7	0.7	0	2
469	5	0.5	0	2
470	5	0.5	0	2
471	6	0.6	0	2
472	4	0.4	0	2
473	8	0.8	0	2
474	5	0.5	0	2
475	2	0.2	0	2
476	1	0.1	0	4
477	5	0.5	0	2
478	5	0.5	0	2
479	2	0.2	0	2
480	10	1	0	2
481	2	0.2	0	2
482	6	0.6	0	2
484	2	0.2	0	2
485	5	0.5	0	2
486	4	0.4	0	2
487	3	0.3	0	2
488	6	0.6	0	2
489	3	0.3	0	2
490	3	0.433333333333333	0	2
491	9	0.9	0	2
492	5	0.5	0	2
493	7	0.766666666666667	0	2
494	5	0.5	0	2
495	3	0.3	0	2
496	2	0.266666666666667	0	2
497	2	0.2	0	2
498	1	0.1	0	4
500	3	0.5	0	2
502	2	0.333333333333333	0	2
504	1	0.166666666666667	0	4
505	2	0.333333333333333	0	2
506	3	0.5	0	2
508	3	0.5	0	2
509	3	0.5	0	2
510	1	0.166666666666667	0	4
511	2	0.2	0	2
512	4	0.4	0	2
513	1	0.1	0	4

514	2	0.2	0	2
515	3	0.3	0	2
516	6	0.6	0	2
517	4	0.4	0	2
519	3	0.3	0	2
520	1	0.1	0	4
521	3	0.3	0	2
522	1	0.1	0	4
523	1	0.1	0	4
524	3	0.3	0	2
525	6	0.6	0	2
527	1	0.1	0	4
528	4	0.4	0	2
530	3	0.3	0	2
531	2	0.2	0	2
532	2	0.2	0	2
533	1	0.1	0	4
534	2	0.2	0	2
535	2	0.2	0	2
536	1	0.1	0	4
537	3	0.3	0	2
539	2	0.2	0	2
540	1	0.1	0	4
541	1	0.1	0	4
542	1	0.1	0	4
543	2	0.2	0	2
544	4	0.4	0	2
545	4	0.4	0	2
546	2	0.2	0	2
548	3	0.3	0	2
549	1	0.1	0	4
551	3	0.3	0	2
552	1	0.1	0	4
553	1	0.1	0	4
555	4	0.4	0	2
556	3	0.3	0	2
558	1	0.1	0	4
559	1	0.1	0	4
560	2	0.2	0	2
561	2	0.2	0	2
563	1	0.1	0	4
564	3	0.3	0	2
565	1	0.1	0	4
566	2	0.2	0	2
567	1	0.1	0	4
569	1	0.16666666666666667	0	4
570	1	0.16666666666666667	0	4
571	3	0.43333333333333333	0	2
572	1	0.1	0	4
574	1	0.1	0	4
575	4	0.4	0	2
576	1	0.1	0	4
578	1	0.1	0	4
581	2	0.2	0	2
582	4	0.46666666666666667	0	2
583	2	0.2	0	2
584	1	0.1	0	4
585	1	0.1	0	4

587	4	0.4	0	2
588	1	0.1	0	4
589	1	0.1	0	4
590	1	0.1	0	4
591	2	0.2	0	2
592	2	0.2	0	2
594	1	0.1	0	4
595	4	0.4	0	2
596	5	0.5	0	2
597	1	0.1	0	4
598	1	0.1	0	4
599	5	0.5	0	2
601	1	0.1	0	4
602	1	0.1	0	4
603	2	0.2	0	2
604	6	0.6	0	2
605	1	0.1	0	4
606	2	0.2	0	2
610	2	0.2	0	2
611	1	0.1	0	4
613	1	0.1	0	4
614	2	0.266666666666667	0	2
615	2	0.266666666666667	0	2
616	2	0.2	0	2
617	1	0.1	0	4
618	1	0.1	0	4
619	1	0.1	0	4
621	1	0.1	0	4
622	5	0.5	0	2
623	1	0.1	0	4
624	5	0.566666666666667	0	2
625	2	0.2	0	2
626	1	0.1	0	4
627	2	0.2	0	2
628	2	0.2	0	2
658	1	0.1	0	4
660	3	0.3	0	2
661	1	0.1	0	4
662	2	0.2	0	2
663	3	0.45	0	2
664	2	0.35	0	2
665	1	0.1	0	4
666	1	0.25	0	4
668	3	0.75	0	2
669	3	0.6	0	2
670	4	0.4	0	2
671	2	0.35	0	2
672	2	0.5	0	2
673	1	0.1	0	4
674	4	1	0	2
675	1	0.25	0	4
676	2	0.35	0	2
677	1	0.25	0	4
679	1	0.25	0	4
680	2	0.35	0	2
682	3	0.3	0	2
683	1	0.1	0	4
684	1	0.1	0	4

686	1	0.1	0	4	
687	3	0.3	0	2	
688	1	0.1	0	4	
690	1	0.25	0	4	
691	1	0.1	0	4	
693	1	0.1	0	4	
694	2	0.2	0	2	
697	3	0.3	0	2	
698	1	0.25	0	4	
699	5	0.5	0	2	
700	1	0.1	0	4	<<<
703	2	0.2	0	2	
711	4	1	0	2	
715	1	0.25	0	4	
719	1	0.25	0	4	
721	2	0.4	0	2	
724	1	0.2	0	4	
725	1	0.2	0	4	
726	3	0.6	0	2	
733	2	0.2	0	2	
734	1	0.1	0	4	
736	2	0.2	0	2	
740	2	0.2	0	2	
741	1	0.1	0	4	
746	1	0.1	0	4	
747	1	0.1	0	4	
763	1	0.1	0	4	
770	1	0.1	0	4	
772	1	0.2	0	4	
778	1	0.2	0	4	
781	1	0.2	0	4	
783	1	0.2	0	4	
784	2	0.4	0	2	
785	3	0.6	0	2	
786	1	0.2	0	4	
787	2	0.4	0	2	
788	1	0.2	0	4	
789	4	0.8	0	2	
790	1	0.2	0	4	
792	1	0.2	0	4	
793	1	0.2	0	4	
796	1	0.2	0	4	
798	3	0.6	0	2	
802	1	0.2	0	4	
803	1	0.25	0	4	
810	1	0.25	0	4	
814	1	0.25	0	4	
815	1	0.25	0	4	
817	1	0.25	0	4	
819	4	1	0	2	
820	1	0.25	0	4	
823	3	0.75	0	2	
824	2	0.5	0	2	
825	2	0.5	0	2	
826	1	0.25	0	4	
827	1	0.25	0	4	
828	1	0.2	0	4	
829	1	0.2	0	4	

```
830	3	0.6	0	2
831	1	0.25	0	4
832	1	0.25	0	4
908	3	0.6	0	2
937	1	0.2	0	4
944	1	0.2	0	4
945	1	0.2	0	4
946	1	0.2	0	4
960	1	0.2	0	4
961	1	0.2	0	4
987	1	0.2	0	4
988	2	0.4	0	2
990	1	0.2	0	4
991	2	0.4	0	2
992	1	0.2	0	4
993	2	0.4	0	2
994	1	0.2	0	4
998	1	0.2	0	4
999	3	0.6	0	2
1005	1	0.2	0	4
1009	1	0.2	0	4
1015	1	0.2	0	4
1016	1	0.2	0	4
```

---

category=4, cleavage_site=2294
query=pto-miR073, target=Potri.T060300.1,
score=3.5, range=2283-2303, strand=1

```
target  5' UGUACCAGAGCaGCUUCUUCA 3'
             :::::.:.:::::::::::
query   3' UAAUGGUUUUGUCGAAGAAGU 5'
```

```
>Potri.T060300.1
#size=2532
38	1	0.166666666666667	0	4
102	1	0.5	0	4
106	1	0.5	0	4
181	1	0.25	0	4
196	1	0.25	0	4
245	1	0.2	0	4
277	1	0.142857142857143	0	4
320	1	0.166666666666667	0	4
333	1	0.125	0	4
586	1	0.1	0	4
673	1	0.2	0	4
746	1	0.1	0	4
755	1	0.166666666666667	0	4
758	1	0.111111111111111	0	4
759	2	0.222222222222222	0	2
847	1	0.1	0	4
848	3	0.3	0	2
876	1	0.1	0	4
895	1	0.1	0	4
1007	1	0.1	0	4
1020	1	0.1	0	4
1026	1	0.1	0	4
1027	1	0.1	0	4
1029	1	0.1	0	4
1129	1	0.1	0	4
1148	1	0.1	0	4
```

1166	1	0.1	0	4	
1167	2	0.2	0	2	
1186	1	0.1	0	4	
1240	1	0.111111111111111	0	4	
1520	1	0.1	0	4	
1531	1	0.1	0	4	
1573	1	0.333333333333333	0	4	
1585	1	0.1	0	4	
1599	1	0.1	0	4	
1669	1	0.1	0	4	
1693	1	0.1	0	4	
1701	1	0.1	0	4	
1726	1	0.1	0	4	
1732	1	0.125	0	4	
1756	1	0.1	0	4	
1769	1	0.1	0	4	
1773	1	0.1	0	4	
1828	1	0.1	0	4	
1843	1	0.1	0	4	
1891	1	0.1	0	4	
1901	1	0.1	0	4	
1975	1	0.1	0	4	
1976	1	0.1	0	4	
2032	1	0.1	0	4	
2101	1	0.166666666666667	0	4	
2124	1	0.5	0	4	
2173	1	0.1	0	4	
2179	1	0.333333333333333	0	4	
2189	1	1	1	4	
2207	1	0.25	0	4	
2285	1	0.1	0	4	
2290	2	0.2	0	2	
2293	4	0.6	0	2	
2294	1	0.1	0	4	<<<
2296	1	0.1	0	4	
2300	1	0.111111111111111	0	4	
2308	1	0.5	0	4	
2327	1	0.25	0	4	
2352	1	0.111111111111111	0	4	
2482	1	0.5	0	4	
2486	1	0.25	0	4	
2489	1	0.25	0	4	

---

category=4, cleavage_site=1714
query=pto-miR073, target=Potri.T060400.1,
score=2.5, range=1703-1723, strand=1

```
target  5' UUUACCAGAACaGUUUCUUCA 3'
           ::::::.::::.:::::::
query   3' UAAUGGUUUUGUCGAAGAAGU 5'
```

>Potri.T060400.1
#size=1952

2	1	0.25	0	4
12	1	0.25	0	4
29	1	0.25	0	4
39	1	0.142857142857143	0	4
82	1	0.166666666666667	0	4
95	1	0.125	0	4
348	1	0.1	0	4

358	1	0.1	0	4	
494	1	0.2	0	4	
495	1	0.1	0	4	
508	1	0.1	0	4	
517	1	0.166666666666667	0	4	
520	1	0.111111111111111	0	4	
521	2	0.222222222222222	0	2	
609	3	0.3	0	2	
610	4	0.4	0	1	
616	1	0.1	0	4	
619	1	0.1	0	4	
620	1	0.1	0	4	
637	2	0.2	0	2	
657	1	0.1	0	4	
740	1	0.1	0	4	
745	1	0.1	0	4	
753	1	0.1	0	4	
768	1	0.1	0	4	
769	1	0.1	0	4	
782	1	0.1	0	4	
788	1	0.1	0	4	
789	1	0.1	0	4	
791	1	0.1	0	4	
848	1	0.1	0	4	
884	1	0.1	0	4	
928	1	0.1	0	4	
929	2	0.2	0	2	
933	1	0.1	0	4	
1347	1	0.1	0	4	
1690	1	0.333333333333333	0	4	
1700	1	0.111111111111111	0	4	
1705	1	0.1	0	4	
1710	2	0.2	0	2	
1713	2	0.4	0	1	
1714	1	0.1	0	4	<<<
1742	1	0.2	0	4	
1799	1	0.1	0	4	

---

category=4, cleavage_site=353
query=pto-miR073, target=Potri.T060600.1,
score=2, range=342-362, strand=1

```
target  5' UUUACCGGAACaGCUUCUUCA 3'
              :::::..:::::::::::::
query   3' UAAUGGUUUUGUCGAAGAAGU 5'
```

>Potri.T060600.1
#size=831

26	1	0.1	0	4	
30	1	0.1	0	4	
85	1	0.1	0	4	
100	1	0.1	0	4	
148	1	0.1	0	4	
158	1	0.1	0	4	
339	1	0.111111111111111	0	4	
344	1	0.1	0	4	
349	2	0.2	0	2	
352	4	0.6	0	2	
353	1	0.1	0	4	<<<
355	1	0.1	0	4	

359	1	0.111111111111111	0	4
380	1	1	1	4
393	1	1	1	4
395	1	1	1	4
398	2	2	2	1
399	1	1	1	4
400	2	2	2	1
428	1	0.1	0	4
433	1	0.1	0	4
438	1	0.1	0	4
451	1	0.1	0	4
457	1	0.333333333333333	0	4
458	1	0.166666666666667	0	4
461	1	0.166666666666667	0	4
469	1	0.5	0	4
472	1	0.5	0	4
478	1	0.5	0	4
481	1	0.5	0	4
487	1	0.2	0	4
490	1	0.2	0	4
499	2	0.5	0	2
520	1	0.142857142857143	0	4
686	1	0.25	0	4

pto-miR080

category=2, cleavage_site=2503
query=pto-miR080, target=Potri.005G181700.1,
score=3, range=2492-2512, strand=1

```
target  5' AGGCUUACAUGgUCGUUAACU 3'
             ::: ::: ::::::.::::::
query   3' UCCAAAUAUACCAGUAAUUGA 5'
```

>Potri.005G181700.1
#size=2567

247	3	1	0	2
254	4	1.33333333333333	0	2
982	1	0.25	0	4
989	2	0.5	0	3
1009	1	0.25	0	4
1012	2	0.5	0	3
1013	1	0.25	0	4
1015	1	0.25	0	4
1023	1	0.25	0	4
1026	1	0.25	0	4
1027	1	0.25	0	4
1034	2	0.583333333333333	0	3
1037	4	1.33333333333333	0	2
1043	3	1	0	2
1044	3	1	0	2
1064	1	0.333333333333333	0	4
1132	3	0.75	0	3
1135	1	0.25	0	4
1339	1	0.25	0	4
1463	4	1.33333333333333	0	2
1481	3	0.75	0	3
1483	1	0.25	0	4
1487	1	0.25	0	4

1561	1	0.25	0	4
1618	1	0.333333333333333	0	4
1619	2	0.666666666666667	0	3
1621	1	0.25	0	4
1622	2	0.5	0	3
1624	4	1	0	2
1625	4	1	0	2
1626	2	0.5	0	3
1627	15	3.75	0	2
1630	2	0.5	0	3
1633	1	0.25	0	4
1634	1	0.25	0	4
1635	2	0.5	0	3
1636	7	1.75	0	2
1637	2	0.5	0	3
1638	1	0.25	0	4
1640	1	0.25	0	4
1641	1	0.25	0	4
1643	3	0.75	0	3
1644	1	0.25	0	4
1647	1	0.25	0	4
1648	2	0.5	0	3
1651	1	0.25	0	4
1654	1	0.25	0	4
1656	1	0.25	0	4
1797	1	0.25	0	4
1849	1	0.25	0	4
2386	1	0.5	0	4
2387	19	9	0	2
2391	1	0.5	0	4
2411	1	0.25	0	4
2413	1	0.166666666666667	0	4
2419	3	0.6	0	3
2424	1	0.2	0	4
2429	1	0.333333333333333	0	4
2435	4	4	4	2
2437	1	1	1	4
2467	5	0.714285714285714	0	3
2469	9	1.25	0	2
2470	9	1.17857142857143	0	2
2471	15	2.00952380952381	0	2
2472	8	1.01785714285714	0	2
2473	18	2.26785714285714	0	2
2474	6	1.64285714285714	1	2
2475	4	0.5	0	3
2476	2	0.267857142857143	0	3
2478	2	0.253968253968254	0	3
2479	7	0.974206349206349	0	3
2480	5	0.714285714285714	0	3
2481	7	0.982142857142857	0	2
2482	1	0.142857142857143	0	4
2484	2	0.285714285714286	0	3
2488	7	1.05714285714286	0	2
2489	3	0.428571428571429	0	3
2490	4	0.610714285714286	0	3
2491	9	1.5	0	2
2492	2	0.333333333333333	0	3
2494	2	0.333333333333333	0	3

2495	2	0.5	0	3	
2496	66	14.45	0	2	
2497	27	6.48333333333334	0	2	
2498	41	9.6	0	2	
2499	11	2.25	0	2	
2500	56	14.0333333333333	0	2	
2501	60	12.7166666666667	0	2	
2502	1	0.2	0	4	
2503	52	13.25	0	2	<<<
2504	50	12.95	0	2	
2505	18	4.45	0	2	
2506	31	7.98333333333333	0	2	
2507	26	8.26666666666666	0	2	
2508	17	5.16666666666667	0	2	
2509	1	0.333333333333333	0	4	
2510	8	2.66666666666667	0	2	
2511	11	2.75	0	2	
2512	25	11.5833333333333	6	2	
2513	29	10.8666666666667	5	2	
2514	10	3.83333333333333	0	2	
2515	13	9.75	8	2	
2516	3	1.5	0	2	
2517	4	2.5	1	2	
2518	16	10.5	5	2	
2519	146	134.833333333333	124	0	
2520	51	47	43	2	
2521	22	10.5333333333333	0	2	
2522	223	110.416666666667	0	2	
2523	89	43.75	0	2	
2524	17	5.83333333333333	0	2	
2525	1	0.5	0	4	
2526	6	2.08333333333333	0	2	
2527	87	29	0	2	
2528	169	80.8666666666667	0	2	
2529	228	112.866666666667	0	2	
2530	17	8	0	2	
2531	5	2.5	0	2	
2532	5	2.5	0	2	
2533	3	1.25	0	2	
2534	14	5.33333333333333	0	2	
2536	1	0.166666666666667	0	4	
2537	7	4.03333333333333	3	2	
2538	4	1.4	0	2	
2539	24	12.4333333333333	10	2	
2540	5	4.2	4	2	
2541	13	10.6666666666667	9	2	
2542	15	12.5	11	2	
2543	2	2	2	2	
2544	4	4	4	2	

---

category=2, cleavage_site=129
query=pto-miR080, target=Potri.012G129800.1,
score=3, range=118-138, strand=1

```
target  5' AGGCUUACAUGgUCGUUAACU 3'
           ::: ::: :::::.::::::
query   3' UCCAAAUAUACCAGUAAUUGA 5'
```

>Potri.012G129800.1
#size=1298

13	3	0.833333333333333	0	2	
14	2	0.666666666666667	0	2	
24	1	0.333333333333333	0	4	
27	1	0.166666666666667	0	4	
34	2	1	0	2	
35	1	0.5	0	4	
37	5	2.25	0	2	
39	1	0.166666666666667	0	4	
45	3	0.6	0	3	
50	1	0.2	0	4	
65	1	0.5	0	4	
93	5	0.714285714285714	0	2	
95	8	1	0	2	
96	9	1.17857142857143	0	2	
97	14	1.80952380952381	0	2	
98	8	1.01785714285714	0	2	
99	18	2.26785714285714	0	2	
100	5	0.642857142857143	0	3	
101	5	0.666666666666667	0	2	
102	2	0.267857142857143	0	3	
104	3	0.453968253968254	0	3	
105	14	2.08134920634921	0	2	
106	8	1.25	0	2	
107	8	1.125	0	2	
108	3	0.55952380952381	0	3	
109	3	0.467857142857143	0	3	
110	6	1.08571428571429	0	2	
111	8	1.2	0	2	
112	2	0.285714285714286	0	3	
113	2	0.476190476190476	0	3	
114	7	1.05714285714286	0	2	
115	3	0.428571428571429	0	3	
116	5	0.810714285714286	0	2	
117	9	1.5	0	2	
118	2	0.333333333333333	0	3	
120	2	0.333333333333333	0	3	
121	2	0.5	0	3	
122	66	14.45	0	2	
123	27	6.48333333333334	0	2	
124	41	9.6	0	2	
125	11	2.25	0	2	
126	57	14.5333333333333	0	0	
127	61	12.8833333333333	0	2	
128	1	0.2	0	4	
129	52	13.25	0	2	<<<
130	50	12.95	0	2	
131	18	4.45	0	2	
132	31	7.98333333333333	0	2	
133	31	9.93333333333333	0	2	
134	17	5.16666666666667	0	2	
136	8	2.66666666666667	0	2	
137	6	1.5	0	2	
138	7	1.83333333333333	0	2	
139	23	5.53333333333334	0	2	
140	8	2.83333333333333	0	2	
141	3	0.75	0	2	
916	1	0.25	0	4	
922	1	1	1	4	

982	2	0.4	0	3
1023	1	0.166666666666667	0	4
1029	1	0.166666666666667	0	4
1033	2	0.333333333333333	0	3
1088	1	0.166666666666667	0	4
1089	1	0.166666666666667	0	4
1101	4	1.33333333333333	0	2
1107	1	0.5	0	4
1108	2	1	0	2
1110	1	0.5	0	4
1111	2	1	0	2
1112	1	0.5	0	4
1114	1	0.5	0	4
1115	1	0.5	0	4
1119	1	0.5	0	4
1120	2	1	0	2
1121	1	0.5	0	4
1122	1	0.5	0	4
1124	5	2.5	0	2
1134	1	0.5	0	4
1135	2	1	0	2
1138	1	0.5	0	4
1144	1	0.5	0	4
1148	1	0.5	0	4
1150	2	1	0	2
1154	1	0.5	0	4
1155	1	0.5	0	4
1166	2	1	0	2
1178	2	1	0	2
1180	1	0.5	0	4
1218	1	0.5	0	4
1268	1	0.5	0	4
1272	1	0.166666666666667	0	4

pto-miR081

category=4, cleavage_site=2288
query=pto-miR081, target=Potri.012G106100.1,
score=3.5, range=2278-2297, strand=1

```
target  5' ACC-CGCCACAcCAGAGCAAG 3'
            ::: ::::::::::: :::.
query   3' UGGCGCGGUGUGGUCUAGUUU 5'
```

>Potri.012G106100.1
#size=4516

55	3	3	3	2
62	1	1	1	4
66	1	1	1	4
67	1	1	1	4
70	1	1	1	4
81	1	1	1	4
82	1	1	1	4
83	1	1	1	4
84	1	1	1	4
91	1	1	1	4
95	1	1	1	4
138	1	1	1	4
148	2	2	2	2

153	1	1	1	4		
161	1	1	1	4		
168	1	1	1	4		
173	1	1	1	4		
179	2	2	2	2		
187	1	1	1	4		
192	1	1	1	4		
209	1	1	1	4		
212	1	1	1	4		
232	1	1	1	4		
242	1	1	1	4		
243	1	1	1	4		
244	1	1	1	4		
255	1	1	1	4		
260	1	1	1	4		
276	2	0.333333333333333			0	3
277	1	0.166666666666667			0	4
278	2	0.333333333333333			0	3
284	1	0.166666666666667			0	4
334	1	0.166666666666667			0	4
387	1	1	1	4		
396	1	1	1	4		
397	1	1	1	4		
417	3	3	3	2		
448	1	1	1	4		
463	1	0.166666666666667			0	4
471	1	0.166666666666667			0	4
517	1	0.166666666666667			0	4
532	1	0.166666666666667			0	4
540	1	0.166666666666667			0	4
545	1	0.166666666666667			0	4
560	1	0.166666666666667			0	4
613	1	0.166666666666667			0	4
615	1	1	1	4		
616	2	0.333333333333333			0	3
624	1	0.1	0	4		
629	1	0.1	0	4		
634	1	0.1	0	4		
654	1	0.166666666666667			0	4
669	1	1	1	4		
716	2	2	2	2		
721	1	1	1	4		
752	2	0.333333333333333			0	3
761	1	0.166666666666667			0	4
765	1	0.166666666666667			0	4
852	1	0.166666666666667			0	4
873	1	0.166666666666667			0	4
878	1	0.166666666666667			0	4
932	2	2	2	2		
970	1	0.166666666666667			0	4
988	1	1	1	4		
1026	1	0.166666666666667			0	4
1043	1	0.166666666666667			0	4
1045	1	0.166666666666667			0	4
1050	1	0.166666666666667			0	4
1056	1	0.166666666666667			0	4
1079	1	0.166666666666667			0	4
1088	1	1	1	4		

1090	1	0.166666666666667	0	4
1093	2	1.16666666666667	1	2
1099	1	0.166666666666667	0	4
1149	1	1	1	4
1151	1	1	1	4
1158	1	0.125	0	4
1169	1	1	1	4
1192	3	0.5	0	3
1198	1	0.166666666666667	0	4
1209	2	0.333333333333333	0	3
1215	1	0.166666666666667	0	4
1244	1	0.166666666666667	0	4
1248	2	0.333333333333333	0	3
1251	1	0.166666666666667	0	4
1253	1	0.166666666666667	0	4
1257	1	0.166666666666667	0	4
1260	1	0.166666666666667	0	4
1263	3	0.5	0	3
1265	1	0.166666666666667	0	4
1286	1	0.166666666666667	0	4
1297	1	0.166666666666667	0	4
1302	1	0.166666666666667	0	4
1308	2	0.333333333333333	0	3
1309	1	0.166666666666667	0	4
1320	1	0.166666666666667	0	4
1327	1	0.166666666666667	0	4
1333	1	1	1	4
1339	1	1	1	4
1341	1	1	1	4
1343	1	1	1	4
1347	1	1	1	4
1356	1	1	1	4
1437	1	1	1	4
1454	1	0.166666666666667	0	4
1465	1	0.166666666666667	0	4
1472	1	0.166666666666667	0	4
1475	1	0.166666666666667	0	4
1490	1	1	1	4
1497	1	1	1	4
1506	1	0.166666666666667	0	4
1510	1	0.166666666666667	0	4
1543	1	1	1	4
1559	1	1	1	4
1594	1	0.166666666666667	0	4
1614	1	0.166666666666667	0	4
1713	1	1	1	4
1714	1	1	1	4
1723	1	1	1	4
1730	1	1	1	4
1731	1	1	1	4
1733	1	1	1	4
1734	1	1	1	4
1736	1	1	1	4
1739	2	2	2	2
1741	1	1	1	4
1743	1	1	1	4
1747	1	1	1	4
1755	1	0.166666666666667	0	4

1756	1	1	1	4
1758	1	0.166666666666667	0	4
1779	1	1	1	4
1840	1	1	1	4
1841	1	1	1	4
1842	1	1	1	4
1845	1	1	1	4
1851	1	1	1	4
1907	1	0.166666666666667	0	4
1914	2	0.333333333333333	0	3
1927	2	0.333333333333333	0	3
1935	1	0.166666666666667	0	4
1956	2	2	2	2
1981	1	1	1	4
1983	1	1	1	4
1987	2	2	2	2
1988	1	1	1	4
2003	1	1	1	4
2011	1	1	1	4
2016	1	1	1	4
2018	1	1	1	4
2020	1	1	1	4
2024	1	1	1	4
2034	1	1	1	4
2035	1	1	1	4
2040	1	1	1	4
2041	1	1	1	4
2053	3	3	3	2
2055	1	1	1	4
2062	2	2	2	2
2089	1	1	1	4
2090	1	1	1	4
2091	1	1	1	4
2101	2	2	2	2
2102	4	4	4	0
2103	2	2	2	2
2111	1	1	1	4
2114	2	2	2	2
2119	1	1	1	4
2128	1	0.166666666666667	0	4
2134	1	0.166666666666667	0	4
2149	1	1	1	4
2159	1	1	1	4
2171	1	1	1	4
2179	1	1	1	4
2180	1	1	1	4
2192	1	1	1	4
2196	2	2	2	2
2200	1	1	1	4
2205	1	1	1	4
2209	2	2	2	2
2210	1	1	1	4
2212	2	2	2	2
2214	1	1	1	4
2216	1	1	1	4
2226	1	1	1	4
2239	1	1	1	4
2244	1	1	1	4

2247	1	1	1	4	
2249	1	1	1	4	
2250	1	1	1	4	
2256	1	1	1	4	
2257	1	1	1	4	
2263	1	1	1	4	
2264	1	1	1	4	
2265	1	1	1	4	
2266	1	1	1	4	
2273	1	1	1	4	
2277	1	1	1	4	
2282	2	2	2	2	
2288	1	1	1	4	<<<
2289	2	2	2	2	
2294	1	1	1	4	
2303	1	1	1	4	
2304	1	1	1	4	
2326	1	1	1	4	
2355	1	1	1	4	
2357	1	1	1	4	
2360	1	1	1	4	
2361	1	1	1	4	
2362	2	2	2	2	
2363	1	1	1	4	
2364	1	1	1	4	
2365	1	1	1	4	
2367	2	2	2	2	
2371	1	1	1	4	
2377	2	2	2	2	
2380	1	1	1	4	
2406	1	1	1	4	
2431	1	1	1	4	
2448	1	1	1	4	
2460	1	1	1	4	
2474	1	1	1	4	
2476	1	1	1	4	
2478	1	1	1	4	
2480	3	3	3	2	
2484	1	1	1	4	
2486	1	1	1	4	
2490	1	0.25	0	4	
2492	1	0.25	0	4	
2495	2	0.5	0	3	
2497	1	0.25	0	4	
2520	3	3	3	2	
2523	1	1	1	4	
2527	1	1	1	4	
2536	1	1	1	4	
2537	3	3	3	2	
2538	1	1	1	4	
2541	1	1	1	4	
2542	1	1	1	4	
2543	2	2	2	2	
2544	1	1	1	4	
2545	1	1	1	4	
2546	1	1	1	4	
2552	1	1	1	4	
2554	1	0.16666666666667	0	4	

2556	1	0.166666666666667	0	4
2557	1	0.166666666666667	0	4
2559	1	0.166666666666667	0	4
2561	3	0.5	0	3
2565	2	0.333333333333333	0	3
2568	1	0.166666666666667	0	4
2569	1	0.166666666666667	0	4
2571	1	0.166666666666667	0	4
2573	1	0.166666666666667	0	4
2575	1	0.166666666666667	0	4
2581	2	0.333333333333333	0	3
2583	4	0.666666666666667	0	3
2585	1	0.166666666666667	0	4
2593	1	1	1	4
2599	1	0.166666666666667	0	4
2606	1	0.166666666666667	0	4
2608	1	0.166666666666667	0	4
2610	1	1	1	4
2614	1	1	1	4
2615	1	0.166666666666667	0	4
2631	1	1	1	4
2645	1	1	1	4
2653	1	0.1	0	4
2656	1	0.1	0	4
2657	2	0.2	0	3
2658	1	0.1	0	4
2659	2	0.2	0	3
2660	1	0.1	0	4
2662	2	0.266666666666667	0	3
2664	1	0.1	0	4
2672	1	0.1	0	4
2673	1	0.2	0	4
2675	1	0.1	0	4
2676	1	0.2	0	4
2677	2	0.4	0	3
2678	1	0.2	0	4
2679	2	0.4	0	3
2680	1	0.2	0	4
2681	1	0.2	0	4
2682	1	0.2	0	4
2684	2	0.4	0	3
2687	3	0.6	0	3
2688	1	0.2	0	4
2689	2	0.4	0	3
2691	1	0.2	0	4
2693	1	1	1	4
2695	1	1	1	4
2696	3	3	3	2
2700	1	1	1	4
2701	1	1	1	4
2703	2	2	2	2
2708	1	1	1	4
2711	1	0.166666666666667	0	4
2712	1	0.166666666666667	0	4
2717	1	1	1	4
2755	1	1	1	4
2757	1	1	1	4
2775	1	1	1	4

2778	1	1	1	4
2785	2	2	2	2
2795	1	1	1	4
2804	1	1	1	4
2807	3	3	3	2
2815	1	1	1	4
2825	1	1	1	4
2833	1	1	1	4
2862	1	1	1	4
2890	1	1	1	4
2906	1	0.2	0	4
2913	1	1	1	4
2938	1	1	1	4
2952	1	1	1	4
2955	1	1	1	4
2956	1	1	1	4
2971	1	1	1	4
2978	1	1	1	4
3001	1	1	1	4
3015	1	1	1	4
3019	2	2	2	2
3027	1	0.2	0	4
3035	1	0.2	0	4
3036	1	0.2	0	4
3039	1	0.2	0	4
3040	1	0.2	0	4
3042	2	0.4	0	3
3050	2	0.4	0	3
3064	1	1	1	4
3073	1	1	1	4
3168	1	1	1	4
3184	1	1	1	4
3187	1	1	1	4
3191	1	1	1	4
3312	1	1	1	4
3403	1	1	1	4
3465	1	1	1	4
3472	1	1	1	4
3561	1	1	1	4

pto-miR084

category=4, cleavage_site=1165
query=pto-miR084, target=Potri.006G055000.1,
score=4, range=1154-1174, strand=1

```
target  5' UCAAGAAUUGCaGGAUCUUGG 3'
            : :::::::::.::::::.
query   3' ACUUCUUAACGUUCUAGAAUA 5'
```

>Potri.006G055000.1
#size=2043

200	1	0.333333333333333	0	4
214	1	0.333333333333333	0	4
230	1	0.333333333333333	0	4
300	1	0.166666666666667	0	4
310	1	0.166666666666667	0	4
334	1	0.166666666666667	0	4
349	1	0.166666666666667	0	4

366	1	0.166666666666667	0	4	
384	1	0.166666666666667	0	4	
396	2	0.333333333333333	0	2	
417	1	0.166666666666667	0	4	
469	1	0.5	0	4	
548	1	0.333333333333333	0	4	
589	1	0.333333333333333	0	4	
643	2	0.285714285714286	0	3	
731	1	0.125	0	4	
736	1	0.125	0	4	
741	1	0.125	0	4	
742	1	0.125	0	4	
781	1	0.333333333333333	0	4	
782	1	0.333333333333333	0	4	
794	1	0.333333333333333	0	4	
805	1	0.333333333333333	0	4	
849	2	0.666666666666667	0	2	
870	2	0.25	0	3	
883	1	0.333333333333333	0	4	
888	1	0.333333333333333	0	4	
910	1	0.333333333333333	0	4	
985	1	0.333333333333333	0	4	
1006	1	0.111111111111111	0	4	
1011	1	0.111111111111111	0	4	
1029	1	0.111111111111111	0	4	
1032	1	0.111111111111111	0	4	
1037	1	0.111111111111111	0	4	
1039	1	0.111111111111111	0	4	
1045	2	0.222222222222222	0	3	
1087	1	0.125	0	4	
1108	1	0.125	0	4	
1110	1	0.125	0	4	
1112	1	0.125	0	4	
1115	1	0.125	0	4	
1126	1	0.333333333333333	0	4	
1149	2	0.666666666666667	0	2	
1165	1	0.333333333333333	0	4	<<<
1168	3	0.375	0	2	
1169	1	0.125	0	4	
1172	1	0.125	0	4	
1174	1	0.125	0	4	
1180	1	0.125	0	4	
1187	1	0.125	0	4	
1195	1	0.333333333333333	0	4	
1208	1	0.333333333333333	0	4	
1265	1	0.5	0	4	
1268	1	0.5	0	4	
1270	4	2	0	2	
1316	2	1	0	2	
1329	1	0.142857142857143	0	4	
1335	2	1	0	2	
1336	1	0.5	0	4	
1338	1	0.5	0	4	
1340	1	0.5	0	4	
1341	5	2.5	0	0	
1342	1	0.5	0	4	
1343	1	0.5	0	4	
1345	1	0.5	0	4	

1348	2	0.666666666666667	0	2
1349	3	1	0	2
1351	2	0.666666666666667	0	2
1356	1	0.333333333333333	0	4
1357	1	0.333333333333333	0	4
1360	1	0.333333333333333	0	4
1371	1	0.333333333333333	0	4
1383	1	0.333333333333333	0	4
1393	1	0.333333333333333	0	4
1397	1	0.333333333333333	0	4
1402	1	0.333333333333333	0	4
1406	1	0.333333333333333	0	4
1413	1	0.333333333333333	0	4
1417	1	0.333333333333333	0	4
1418	1	0.333333333333333	0	4
1419	2	0.666666666666667	0	2
1420	1	0.333333333333333	0	4
1435	1	0.333333333333333	0	4
1436	1	0.333333333333333	0	4
1442	1	0.333333333333333	0	4
1453	1	0.333333333333333	0	4
1459	1	0.333333333333333	0	4
1466	1	0.333333333333333	0	4
1467	1	0.333333333333333	0	4
1475	1	0.333333333333333	0	4
1477	2	0.25	0	3
1478	1	0.125	0	4
1491	1	0.333333333333333	0	4
1494	2	0.666666666666667	0	2

pto-miR087

category=4, cleavage_site=3191
query=pto-miR087, target=Potri.002G188700.1,
score=4, range=3181-3200, strand=1

```
target  5' CUAGCAAUCUcUCAAACAGU 3'
              ::::  :: ::::::::::
query   3' GAUCUAUACAGAGUUUGUCA 5'
```

>Potri.002G188700.1
#size=3699

262	1	0.25	0	4
304	1	0.2	0	4
308	1	0.2	0	4
320	2	0.285714285714286	0	2
349	2	0.285714285714286	0	2
366	1	0.142857142857143	0	4
374	1	0.142857142857143	0	4
427	2	0.4	0	2
428	1	0.2	0	4
485	1	0.2	0	4
500	1	0.2	0	4
512	1	0.2	0	4
523	1	0.2	0	4
621	2	0.285714285714286	0	2
624	1	0.142857142857143	0	4
639	2	0.253968253968254	0	2
671	1	0.2	0	4

694	1	0.2	0	4
724	2	0.4	0	2
728	1	0.2	0	4
757	1	0.2	0	4
761	1	0.2	0	4
763	1	0.142857142857143	0	4
766	1	0.142857142857143	0	4
776	1	0.142857142857143	0	4
787	1	0.142857142857143	0	4
792	2	0.285714285714286	0	2
796	1	0.142857142857143	0	4
826	1	0.142857142857143	0	4
841	1	0.2	0	4
865	1	0.2	0	4
878	1	0.2	0	4
932	1	0.2	0	4
971	1	0.2	0	4
977	2	0.4	0	2
1007	1	0.2	0	4
1051	1	0.2	0	4
1062	1	0.142857142857143	0	4
1143	1	0.2	0	4
1145	2	0.242857142857143	0	2
1147	1	0.1	0	4
1149	1	0.142857142857143	0	4
1151	1	0.142857142857143	0	4
1153	1	0.142857142857143	0	4
1157	1	0.142857142857143	0	4
1159	1	0.142857142857143	0	4
1161	1	0.142857142857143	0	4
1162	1	0.142857142857143	0	4
1163	1	0.142857142857143	0	4
1176	1	0.142857142857143	0	4
1213	1	0.2	0	4
1219	1	0.2	0	4
1242	1	0.142857142857143	0	4
1244	1	0.142857142857143	0	4
1245	1	0.2	0	4
1246	1	0.142857142857143	0	4
1249	1	0.142857142857143	0	4
1250	1	0.142857142857143	0	4
1252	1	0.142857142857143	0	4
1253	1	0.142857142857143	0	4
1255	1	0.142857142857143	0	4
1264	1	0.142857142857143	0	4
1269	2	0.285714285714286	0	2
1275	1	0.142857142857143	0	4
1276	1	0.142857142857143	0	4
1295	2	0.333333333333333	0	2
1296	1	0.166666666666667	0	4
1340	1	0.166666666666667	0	4
1377	1	0.2	0	4
1381	1	0.2	0	4
1386	1	0.2	0	4
1393	1	0.2	0	4
1411	1	0.142857142857143	0	4
1415	1	0.142857142857143	0	4
1441	1	1	1	4

1450	2	2	2	0
1454	1	1	1	4
1471	2	0.4	0	2
1473	2	0.4	0	2
1475	1	0.2	0	4
1481	1	0.2	0	4
1492	1	0.2	0	4
1506	1	0.142857142857143	0	4
1514	1	0.142857142857143	0	4
1515	1	0.2	0	4
1528	1	0.2	0	4
1541	1	0.142857142857143	0	4
1602	2	0.285714285714286	0	2
1605	1	0.142857142857143	0	4
1610	1	0.142857142857143	0	4
1612	1	0.142857142857143	0	4
1614	1	0.2	0	4
1617	2	0.4	0	2
1619	1	0.142857142857143	0	4
1620	2	0.285714285714286	0	2
1621	3	0.485714285714286	0	2
1622	1	0.2	0	4
1630	1	0.142857142857143	0	4
1631	1	0.142857142857143	0	4
1633	1	0.142857142857143	0	4
1635	1	0.142857142857143	0	4
1639	1	0.142857142857143	0	4
1640	1	0.142857142857143	0	4
1649	4	0.571428571428571	0	2
1660	2	0.285714285714286	0	2
1663	1	0.142857142857143	0	4
1676	1	0.142857142857143	0	4
1692	1	0.142857142857143	0	4
1709	1	0.2	0	4
1713	1	0.142857142857143	0	4
1746	1	0.2	0	4
1754	1	0.142857142857143	0	4
1767	1	0.142857142857143	0	4
1781	1	0.142857142857143	0	4
1791	1	0.142857142857143	0	4
1843	1	0.142857142857143	0	4
1873	1	0.2	0	4
1919	1	0.142857142857143	0	4
2059	1	0.2	0	4
2072	1	0.142857142857143	0	4
2088	1	0.2	0	4
2117	1	0.2	0	4
2149	1	0.142857142857143	0	4
2202	1	0.2	0	4
2210	1	0.2	0	4
2230	1	0.142857142857143	0	4
2241	4	0.571428571428571	0	2
2247	1	0.142857142857143	0	4
2258	1	0.142857142857143	0	4
2310	1	0.142857142857143	0	4
2322	1	0.142857142857143	0	4
2331	1	0.142857142857143	0	4
2338	1	0.142857142857143	0	4

2366	1	0.2	0	4
2383	1	0.142857142857143	0	4
2408	1	0.2	0	4
2411	1	0.2	0	4
2448	1	0.142857142857143	0	4
2461	1	0.2	0	4
2478	1	0.2	0	4
2513	2	0.342857142857143	0	2
2534	1	0.2	0	4
2544	1	0.2	0	4
2548	1	0.2	0	4
2555	1	0.2	0	4
2556	1	0.2	0	4
2566	1	0.142857142857143	0	4
2574	2	0.4	0	2
2575	1	0.2	0	4
2592	1	0.2	0	4
2639	1	0.2	0	4
2652	1	0.2	0	4
2660	1	0.2	0	4
2665	1	0.142857142857143	0	4
2682	1	0.2	0	4
2685	1	0.2	0	4
2689	1	0.2	0	4
2693	2	0.4	0	2
2694	2	0.4	0	2
2700	1	0.2	0	4
2705	1	0.2	0	4
2706	2	0.4	0	2
2707	1	0.2	0	4
2713	1	0.2	0	4
2719	1	0.2	0	4
2780	1	0.142857142857143	0	4
2786	1	0.142857142857143	0	4
2809	1	0.142857142857143	0	4
2810	1	0.142857142857143	0	4
2814	1	0.142857142857143	0	4
2829	1	0.142857142857143	0	4
2831	1	0.142857142857143	0	4
2836	1	0.142857142857143	0	4
2838	1	0.2	0	4
2852	1	0.2	0	4
2890	1	0.2	0	4
2893	2	0.285714285714286	0	2
2896	1	0.142857142857143	0	4
2902	1	0.142857142857143	0	4
2906	1	0.142857142857143	0	4
2921	1	0.142857142857143	0	4
2955	1	0.166666666666667	0	4
3008	1	0.2	0	4
3010	1	0.2	0	4
3017	1	0.2	0	4
3034	1	0.2	0	4
3041	1	0.25	0	4
3044	1	0.25	0	4
3047	1	0.25	0	4
3051	1	0.25	0	4
3059	1	0.2	0	4

3064	5	1	0	2	
3070	1	0.25	0	4	
3071	1	0.25	0	4	
3079	2	0.4	0	2	
3080	2	0.4	0	2	
3104	1	0.25	0	4	
3106	1	0.25	0	4	
3108	1	0.25	0	4	
3182	1	0.25	0	4	
3191	1	0.25	0	4	<<<
3193	1	0.25	0	4	
3233	1	0.25	0	4	
3248	1	0.25	0	4	
3250	2	0.5	0	2	
3257	1	0.25	0	4	
3274	1	0.2	0	4	
3275	1	0.2	0	4	
3289	1	0.2	0	4	
3309	1	0.166666666666667	0	4	
3317	2	0.333333333333333	0	2	
3333	1	0.166666666666667	0	4	
3336	1	0.166666666666667	0	4	
3337	1	0.166666666666667	0	4	
3357	1	0.166666666666667	0	4	
3367	1	0.25	0	4	
3395	1	0.25	0	4	
3396	2	0.5	0	2	
3400	1	0.25	0	4	
3401	1	0.25	0	4	
3402	1	0.2	0	4	
3403	2	0.4	0	2	
3404	3	0.6	0	2	
3405	1	0.2	0	4	
3407	1	0.2	0	4	
3409	1	0.2	0	4	
3414	2	0.4	0	2	
3415	1	0.2	0	4	
3417	2	0.4	0	2	
3426	1	0.2	0	4	
3430	1	0.2	0	4	
3431	1	0.2	0	4	
3433	1	0.2	0	4	
3436	1	0.2	0	4	
3440	1	0.2	0	4	
3443	1	0.142857142857143	0	4	
3452	1	0.2	0	4	
3456	1	0.2	0	4	
3462	1	0.2	0	4	
3465	1	0.2	0	4	
3466	1	0.2	0	4	
3470	2	0.4	0	2	
3507	1	0.2	0	4	
3509	3	0.6	0	2	
3514	1	0.2	0	4	
3603	1	0.2	0	4	

pto-miR091

category=3, cleavage_site=215
query=pto-miR091, target=Potri.002G070100.1,
score=4, range=204-224, strand=1

```
target  5' UACAAGUACUCcUCUCCUCCA 3'
             : :: :: ::::.::::::::
query   3' ACGUGCACGAGGGGAGGAGGU 5'
```

>Potri.002G070100.1
#size=858

102	1	0.5	0	4	
104	1	0.5	0	4	
105	1	0.5	0	4	
111	1	0.5	0	4	
115	2	1	0	2	
117	2	1	0	2	
118	1	0.5	0	4	
119	1	0.5	0	4	
122	2	1	0	2	
123	1	0.5	0	4	
125	1	0.5	0	4	
130	2	1	0	2	
131	2	1	0	2	
137	1	0.5	0	4	
140	1	0.5	0	4	
143	1	0.5	0	4	
152	1	0.5	0	4	
155	1	0.5	0	4	
158	1	0.5	0	4	
159	1	0.5	0	4	
161	1	0.5	0	4	
164	1	0.5	0	4	
165	1	0.5	0	4	
170	1	0.5	0	4	
172	1	0.5	0	4	
173	1	0.5	0	4	
175	1	0.5	0	4	
177	1	0.5	0	4	
182	1	0.5	0	4	
183	1	0.5	0	4	
186	1	0.5	0	4	
189	1	0.5	0	4	
190	1	0.5	0	4	
191	1	0.5	0	4	
192	1	0.5	0	4	
194	1	0.5	0	4	
198	2	1	0	2	
201	4	2	0	2	
206	3	1.5	0	2	
209	1	0.333333333333333	0	4	
210	2	0.666666666666667	0	3	
211	2	0.666666666666667	0	3	
212	1	0.333333333333333	0	4	
213	1	0.333333333333333	0	4	
214	1	0.333333333333333	0	4	
215	2	0.666666666666667	0	3	<<<
216	1	0.333333333333333	0	4	

217	1	0.333333333333333	0	4
218	2	0.833333333333333	0	3
219	1	0.333333333333333	0	4
222	1	0.333333333333333	0	4
224	3	1	0	2
225	1	0.333333333333333	0	4
226	2	0.666666666666667	0	3
228	3	1.5	0	2
229	1	0.333333333333333	0	4
230	1	0.333333333333333	0	4
231	2	1	0	2
233	3	1.5	0	2
234	1	0.5	0	4
236	1	0.5	0	4
237	1	0.5	0	4
239	1	0.5	0	4
242	1	0.5	0	4
243	1	0.5	0	4
246	1	0.5	0	4
248	1	0.5	0	4
250	2	1	0	2
252	1	0.5	0	4
253	3	1.5	0	2
254	2	1	0	2
255	1	0.5	0	4
256	1	0.5	0	4
259	4	2	0	2
267	3	1.5	0	2
268	3	1.5	0	2
269	3	1.5	0	2
270	2	1	0	2
462	1	1	1	4
472	1	1	1	4
481	3	1.5	0	2
483	1	0.5	0	4
516	9	4.5	0	2
517	9	4.5	0	2
518	5	2.5	0	2
519	2	1	0	2
520	7	3.5	0	2
521	6	3	0	2
522	11	5.5	0	2
523	2	1	0	2
524	5	2.5	0	2
525	4	2	0	2
527	3	1.5	0	2
528	5	2.5	0	2
531	1	0.5	0	4
533	2	1	0	2
534	1	0.5	0	4
535	1	0.5	0	4
536	2	1	0	2
537	2	1	0	2
538	1	0.5	0	4
539	3	1.5	0	2
540	3	1.5	0	2
541	5	2.5	0	2
542	1	0.5	0	4

543	10	5	0	2
544	6	3	0	2
545	5	2.5	0	2
546	11	5.5	0	2
547	6	3	0	2
548	8	4	0	2
549	6	3	0	2
550	4	2	0	2
551	3	1.5	0	2
552	4	2	0	2
554	1	0.5	0	4
555	4	2	0	2
556	3	1.5	0	2
557	2	1	0	2
559	2	1	0	2
561	2	1	0	2
562	2	1	0	2
563	1	0.5	0	4
564	2	1	0	2
565	7	3.5	0	2
566	3	1.5	0	2
568	2	1	0	2
569	4	2	0	2
570	3	1.5	0	2
571	1	0.5	0	4
572	8	4	0	2
573	3	1.5	0	2
574	4	2	0	2
575	4	2	0	2
576	1	0.5	0	4
577	2	1	0	2
578	7	3.5	0	2
579	2	1	0	2
580	3	1.5	0	2
581	5	2.5	0	2
582	5	2.5	0	2
583	2	1	0	2
584	4	2	0	2
585	6	3	0	2
586	2	1	0	2
587	3	1.5	0	2
588	4	2	0	2
589	5	2.5	0	2
590	9	4.5	0	2
591	4	2	0	2
592	17	8.5	0	2
593	15	7.5	0	2
594	7	3.5	0	2
595	9	4.5	0	2
596	16	8	0	2
597	10	5	0	2
598	9	4.5	0	2
599	20	10	0	0
600	10	5	0	2
601	8	4	0	2
602	8	4	0	2
603	7	3.5	0	2
604	4	2	0	2

605	1	0.5	0	4
606	7	3.5	0	2
607	12	6	0	2
608	5	2.5	0	2
609	7	3.5	0	2
610	8	4	0	2
611	7	3.5	0	2
612	8	4	0	2
613	8	4	0	2
614	5	2.5	0	2
615	6	3	0	2
616	1	0.5	0	4
617	11	5.5	0	2
618	3	1.5	0	2
619	4	2	0	2
620	3	1.5	0	2
621	5	2.5	0	2
622	2	1	0	2
623	2	1	0	2
624	1	0.5	0	4
626	3	1.5	0	2
627	3	1.5	0	2
628	3	1.5	0	2
629	1	0.5	0	4
630	1	0.5	0	4
631	2	1	0	2
633	1	0.5	0	4
634	3	1.5	0	2
635	1	0.5	0	4
636	1	0.5	0	4
637	3	1.5	0	2
638	9	4.5	0	2
639	2	1	0	2
641	2	1	0	2
642	2	1	0	2
644	5	2.5	0	2
646	4	2	0	2
647	1	0.5	0	4
648	1	0.5	0	4
649	2	1	0	2
651	5	2.5	0	2
652	2	1	0	2
654	1	0.5	0	4
656	6	3	0	2
658	1	0.5	0	4
661	2	1	0	2
662	1	0.5	0	4
665	2	1	0	2
667	1	0.5	0	4
668	3	1.5	0	2
669	2	1	0	2
670	1	0.5	0	4
673	1	0.5	0	4
674	1	0.5	0	4
675	1	0.5	0	4
708	2	1	0	2
753	1	0.5	0	4

category=2, cleavage_site=123
query=pto-miR091, target=Potri.008G195200.1,
score=4, range=113-132, strand=1

```
target  5' UG-ACCUCCUCcUCUCCUCCA 3'
           :: :: : ::::.::::::::
query   3' ACGUGCACGAGGGGAGGAGGU 5'
```

>Potri.008G195200.1
#size=2014

75	1	0.125	0	4	
82	1	0.125	0	4	
117	1	0.125	0	4	
118	1	0.125	0	4	
123	2	0.25	0	2	<<<
173	1	0.125	0	4	
208	1	0.125	0	4	
217	1	0.125	0	4	
232	1	0.125	0	4	
246	1	0.125	0	4	
287	1	0.1	0	4	
299	1	0.1	0	4	
314	1	0.125	0	4	
330	2	0.25	0	2	
424	1	0.1	0	4	
441	1	0.1	0	4	
458	1	0.1	0	4	
535	1	0.125	0	4	
573	1	0.125	0	4	
660	1	0.1	0	4	
723	1	0.1	0	4	
795	1	0.1	0	4	
824	2	0.2	0	2	
851	1	0.125	0	4	
890	1	0.125	0	4	
893	1	0.125	0	4	
956	1	0.1	0	4	
963	1	0.1	0	4	
964	1	0.1	0	4	
1033	1	0.125	0	4	
1034	1	0.125	0	4	
1061	1	0.125	0	4	
1062	1	0.125	0	4	
1086	1	0.125	0	4	
1163	1	0.1	0	4	
1219	1	0.1	0	4	
1232	1	0.1	0	4	
1262	1	0.125	0	4	
1280	1	0.1	0	4	
1294	1	0.125	0	4	
1323	1	0.125	0	4	
1334	1	0.1	0	4	
1343	1	0.1	0	4	
1345	1	0.1	0	4	
1357	1	0.1	0	4	
1359	1	0.1	0	4	
1363	1	0.1	0	4	
1407	1	0.1	0	4	
1413	1	0.1	0	4	
1422	1	0.1	0	4	

1505	1	0.5	0	4
1534	1	0.5	0	4
1649	1	0.142857142857143	0	4
1663	1	0.142857142857143	0	4
1702	1	0.142857142857143	0	4
1712	2	0.285714285714286	0	2
1757	1	0.125	0	4
1760	1	0.125	0	4
1765	1	0.125	0	4
1770	1	0.125	0	4
1772	1	0.125	0	4
1774	1	0.125	0	4
1778	1	0.125	0	4
1785	1	0.125	0	4
1796	1	0.125	0	4
1797	2	0.25	0	2
1804	2	0.25	0	2
1806	2	0.25	0	2
1810	2	0.25	0	2
1811	1	0.125	0	4
1934	1	0.1	0	4
1937	1	0.1	0	4
1942	1	0.1	0	4
1946	1	0.1	0	4

pto-miR092

category=4, cleavage_site=2988
query=pto-miR092, target=Potri.001G179000.2,
score=3, range=2978-2997, strand=1

```
target  5' UACCAUUUUAuCAGGUCCUU 3'
           .::::.:.::::::::.:::::
query   3' GUGGUGAGAUAGUCUAGGAA 5'
```

>Potri.001G179000.2
#size=3475

241	1	0.166666666666667	0	4
350	2	0.333333333333333	0	2
555	1	0.166666666666667	0	4
657	2	0.333333333333333	0	2
685	3	0.5	0	1
712	1	0.166666666666667	0	4
721	1	0.166666666666667	0	4
827	1	0.166666666666667	0	4
846	1	0.166666666666667	0	4
857	1	0.166666666666667	0	4
884	1	0.166666666666667	0	4
891	1	0.166666666666667	0	4
895	2	0.333333333333333	0	2
899	2	0.333333333333333	0	2
912	1	0.166666666666667	0	4
913	1	0.166666666666667	0	4
914	1	0.166666666666667	0	4
931	1	0.166666666666667	0	4
935	1	0.166666666666667	0	4
944	1	0.166666666666667	0	4
945	1	0.166666666666667	0	4
968	1	0.166666666666667	0	4

979	1	0.166666666666667	0	4
1005	1	0.166666666666667	0	4
1011	1	0.166666666666667	0	4
1028	1	0.166666666666667	0	4
1031	1	0.166666666666667	0	4
1069	2	0.333333333333333	0	2
1070	1	0.166666666666667	0	4
1076	1	0.166666666666667	0	4
1089	2	0.333333333333333	0	2
1093	1	0.166666666666667	0	4
1094	2	0.333333333333333	0	2
1120	1	0.166666666666667	0	4
1208	2	0.333333333333333	0	2
1293	1	0.166666666666667	0	4
1298	1	0.166666666666667	0	4
1305	1	0.166666666666667	0	4
1425	1	0.166666666666667	0	4
1431	1	0.166666666666667	0	4
1451	1	0.166666666666667	0	4
1488	1	0.166666666666667	0	4
1545	2	0.333333333333333	0	2
1570	1	0.166666666666667	0	4
1641	1	0.166666666666667	0	4
1689	1	0.166666666666667	0	4
1847	1	0.166666666666667	0	4
1940	1	0.166666666666667	0	4
1945	1	0.166666666666667	0	4
1947	2	0.333333333333333	0	2
1953	1	0.166666666666667	0	4
1966	1	0.166666666666667	0	4
1975	1	0.166666666666667	0	4
1988	1	0.166666666666667	0	4
1989	1	0.166666666666667	0	4
2009	1	0.166666666666667	0	4
2024	1	0.166666666666667	0	4
2032	1	0.166666666666667	0	4
2050	1	0.2	0	4
2061	1	0.2	0	4
2096	1	0.2	0	4
2220	1	0.2	0	4
2236	2	0.333333333333333	0	2
2238	1	0.166666666666667	0	4
2249	1	0.166666666666667	0	4
2252	2	0.333333333333333	0	2
2274	1	0.166666666666667	0	4
2276	1	0.166666666666667	0	4
2287	1	0.166666666666667	0	4
2294	1	0.166666666666667	0	4
2320	1	0.166666666666667	0	4
2350	1	0.166666666666667	0	4
2359	2	0.333333333333333	0	2
2371	1	0.166666666666667	0	4
2378	1	0.166666666666667	0	4
2381	2	0.333333333333333	0	2
2384	1	0.166666666666667	0	4
2423	1	0.166666666666667	0	4
2428	1	0.166666666666667	0	4
2433	1	0.166666666666667	0	4

2468	1	0.166666666666667	0	4	
2469	2	0.333333333333333	0	2	
2474	1	0.166666666666667	0	4	
2479	1	0.166666666666667	0	4	
2481	1	0.166666666666667	0	4	
2489	3	0.5	0	1	
2490	1	0.166666666666667	0	4	
2491	1	0.166666666666667	0	4	
2988	1	0.333333333333333	0	4	<<<
2992	1	0.25	0	4	
2995	2	0.5	0	1	
2996	1	0.25	0	4	
2997	1	0.25	0	4	
2998	1	0.25	0	4	
3014	1	0.2	0	4	
3028	1	0.2	0	4	
3031	1	0.2	0	4	
3036	1	0.2	0	4	
3044	1	0.2	0	4	
3048	1	0.2	0	4	
3056	1	0.2	0	4	
3072	1	0.2	0	4	
3074	1	0.2	0	4	
3080	1	0.166666666666667	0	4	
3085	1	0.2	0	4	
3116	1	0.166666666666667	0	4	
3119	1	0.166666666666667	0	4	
3126	1	0.166666666666667	0	4	
3128	1	0.166666666666667	0	4	
3131	2	0.333333333333333	0	2	
3143	1	0.166666666666667	0	4	
3146	1	0.166666666666667	0	4	
3155	1	0.166666666666667	0	4	
3156	1	0.166666666666667	0	4	
3157	1	0.166666666666667	0	4	
3165	1	0.166666666666667	0	4	
3238	1	0.2	0	4	
3247	1	0.2	0	4	
3252	1	0.2	0	4	
3319	1	0.2	0	4	
3323	1	0.2	0	4	
3330	1	0.2	0	4	

pto-miR095

---

category=4, p-value=0.441806103703277, cleavage_site=2500
query= pto-miR095, target=Potri.005G191500.1,
score=4, range=2489-2509, strand=1

```
target  5' CUUUUCUGAGCcUUUAUGAUG 3'
              ::: :::::::.:.::::::
query   3' CAAAUGACUCGGGAGUACUAC 5'
```

>Potri.005G191500.1
#size=3433

6	1	0.333333333333333	0	4
9	1	0.333333333333333	0	4
12	1	0.333333333333333	0	4
20	1	0.333333333333333	0	4

24	2	1	0	2
40	1	0.333333333333333	0	4
62	1	0.5	0	4
89	1	0.333333333333333	0	4
104	1	0.5	0	4
118	1	0.5	0	4
178	1	0.5	0	4
203	1	0.5	0	4
214	4	1.83333333333333	0	0
218	1	0.5	0	4
223	2	0.666666666666667	0	2
259	1	0.333333333333333	0	4
261	2	1	0	2
312	1	0.5	0	4
340	1	0.333333333333333	0	4
349	1	0.5	0	4
350	1	0.333333333333333	0	4
356	1	0.333333333333333	0	4
374	1	0.333333333333333	0	4
474	1	0.333333333333333	0	4
572	1	0.333333333333333	0	4
576	1	0.333333333333333	0	4
610	1	0.5	0	4
613	1	0.333333333333333	0	4
636	1	0.5	0	4
645	1	0.5	0	4
707	1	0.333333333333333	0	4
709	1	0.333333333333333	0	4
741	1	0.333333333333333	0	4
764	1	0.333333333333333	0	4
806	1	0.333333333333333	0	4
813	2	0.666666666666667	0	2
816	1	0.333333333333333	0	4
831	1	0.5	0	4
893	1	0.5	0	4
958	1	0.5	0	4
979	1	0.5	0	4
1094	1	0.5	0	4
1245	1	0.333333333333333	0	4
1283	2	1	0	2
1376	1	0.333333333333333	0	4
1386	1	0.333333333333333	0	4
1449	1	0.333333333333333	0	4
1569	1	0.333333333333333	0	4
1663	1	0.333333333333333	0	4
1670	3	1	0	2
1701	1	0.333333333333333	0	4
1707	1	0.333333333333333	0	4
1714	1	0.333333333333333	0	4
1716	1	0.333333333333333	0	4
1717	1	0.333333333333333	0	4
1814	1	0.333333333333333	0	4
1824	1	0.5	0	4
1871	1	0.333333333333333	0	4
1910	1	0.333333333333333	0	4
1938	1	0.333333333333333	0	4
1939	1	0.333333333333333	0	4
1988	1	0.333333333333333	0	4

1998	1	0.333333333333333	0	4	
2011	1	0.333333333333333	0	4	
2015	1	0.333333333333333	0	4	
2085	1	0.333333333333333	0	4	
2112	1	0.5	0	4	
2133	1	0.333333333333333	0	4	
2197	1	0.5	0	4	
2199	1	0.5	0	4	
2205	1	0.5	0	4	
2211	1	0.333333333333333	0	4	
2240	1	0.333333333333333	0	4	
2338	2	0.833333333333333	0	2	
2370	1	0.5	0	4	
2388	1	0.5	0	4	
2393	1	0.333333333333333	0	4	
2399	3	1	0	2	
2402	1	0.333333333333333	0	4	
2446	1	0.5	0	4	
2500	1	0.5	0	4	<<<
2513	1	0.5	0	4	
2519	1	0.5	0	4	
2536	1	0.333333333333333	0	4	
2588	1	0.333333333333333	0	4	
2605	1	0.333333333333333	0	4	
2673	1	0.5	0	4	
2676	1	0.5	0	4	
2694	2	0.666666666666667	0	2	
2704	1	0.333333333333333	0	4	
2708	1	0.333333333333333	0	4	
2709	1	0.333333333333333	0	4	
2711	2	0.666666666666667	0	2	
2714	1	0.333333333333333	0	4	
2744	1	0.5	0	4	
2752	1	0.5	0	4	
2757	1	1	1	4	
2759	1	1	1	4	
2787	1	0.5	0	4	
2794	1	1	1	4	
2800	2	1	0	2	
2801	1	0.5	0	4	
2814	1	0.5	0	4	
2815	2	1	0	2	
2819	1	0.5	0	4	
2852	1	1	1	4	
2867	1	0.5	0	4	
2873	1	0.5	0	4	
2875	1	0.5	0	4	
2892	1	0.5	0	4	
2901	1	0.5	0	4	
2918	1	1	1	4	
2921	1	1	1	4	
2943	1	1	1	4	
2948	1	1	1	4	
2950	1	1	1	4	
2962	1	0.5	0	4	
2976	1	0.5	0	4	
2977	1	1	1	4	
3076	1	1	1	4	

3107	1	1	1	4
3131	1	1	1	4

pto-miR105

category=2, cleavage_site=922
query=pto-miR105, target=Potri.019G022800.1,
score=2.5, range=911-931, strand=1

```
target  5' CUAUGUCUCGAgAUUUCAGCA 3'
           :. ::::::::::::::::.::
query   3' GGCACAGAGCUCUAAAGUUGU 5'
```

>Potri.019G022800.1
#size=3135

150	1	0.125	0	4	
221	1	1	1	4	
922	2	0.2	0	2	<<<
1228	1	0.1	0	4	
1233	1	0.1	0	4	
1239	1	0.1	0	4	
1357	1	0.1	0	4	
1360	1	0.1	0	4	
1518	1	0.1	0	4	
1569	1	0.1	0	4	
1637	1	0.1	0	4	
2756	1	0.111111111111111	0	4	
2763	1	0.1	0	4	
2765	1	0.1	0	4	
2772	1	0.1	0	4	
2981	1	0.1	0	4	
2986	1	0.1	0	4	
2990	2	0.285714285714286	0	2	

category=4, cleavage_site=712
query=pto-miR105, target=Potri.T013600.1,
score=2, range=701-721, strand=1

```
target  5' CCGUGACUCGAgAUUUCAGCA 3'
           ::::: ::::::::::::.::
query   3' GGCACAGAGCUCUAAAGUUGU 5'
```

>Potri.T013600.1
#size=2760

105	1	0.111111111111111	0	4	
111	1	0.1	0	4	
165	1	0.2	0	4	
230	1	0.1	0	4	
245	1	0.111111111111111	0	4	
251	1	0.111111111111111	0	4	
270	1	0.142857142857143	0	4	
290	1	0.111111111111111	0	4	
297	1	0.125	0	4	
354	1	0.2	0	4	
398	1	0.1	0	4	
411	2	0.2	0	2	
451	1	0.1	0	4	
619	3	3	3	0	
622	1	0.5	0	4	
712	1	0.333333333333333	0	4	<<<
987	1	0.1	0	4	

993	1	0.1	0	4
1000	1	0.1	0	4
1001	1	0.1	0	4
1063	1	0.111111111111111	0	4
1066	1	0.1	0	4
1067	1	0.1	0	4
1073	1	0.1	0	4
1076	1	0.1	0	4
1079	1	0.1	0	4
1092	1	0.125	0	4
1093	1	0.125	0	4
1094	1	0.1	0	4
1099	1	0.125	0	4
1100	1	0.142857142857143	0	4
1143	1	0.1	0	4
1146	1	0.1	0	4
1150	1	0.1	0	4
1190	1	0.166666666666667	0	4
1214	1	0.1	0	4
1215	1	0.1	0	4
1316	2	0.2	0	2
1401	1	0.1	0	4
1488	1	0.1	0	4
1490	1	0.142857142857143	0	4
1519	1	0.1	0	4
1523	1	0.142857142857143	0	4
1627	1	0.1	0	4
1629	1	0.1	0	4
1642	1	0.1	0	4
1752	2	0.285714285714286	0	2
1778	1	0.1	0	4
1814	1	0.1	0	4
1854	2	0.2	0	2
1862	1	0.1	0	4
2036	1	0.142857142857143	0	4
2045	4	0.4	0	2
2046	1	0.166666666666667	0	4
2052	1	0.25	0	4
2053	1	0.142857142857143	0	4
2058	2	0.361111111111111	0	2
2065	1	0.142857142857143	0	4
2290	1	0.1	0	4
2293	1	0.1	0	4
2294	3	0.3	0	2
2296	1	0.1	0	4
2297	1	0.1	0	4
2300	1	0.1	0	4
2347	1	0.111111111111111	0	4
2420	1	0.25	0	4
2466	2	0.433333333333333	0	2
2467	1	0.1	0	4
2470	1	0.1	0	4
2473	1	0.1	0	4
2480	1	0.142857142857143	0	4
2484	1	0.1	0	4
2495	1	0.166666666666667	0	4
2511	1	0.142857142857143	0	4
2624	1	0.25	0	4

category=4, cleavage_site=954
query=pto-miR105, target=Potri.T015900.1,
score=1, range=943-963, strand=1

```
target  5' CCGUGUCUCGAgAUUUCAGCA 3'
            :::::::::::::::::.::
query   3' GGCACAGAGCUCUAAAGUUGU 5'
```

>Potri.T015900.1
#size=3098

3	1	0.2	0	4	
22	1	0.333333333333333	0	4	
110	1	0.25	0	4	
178	1	0.2	0	4	
191	1	0.2	0	4	
347	1	0.111111111111111	0	4	
353	1	0.1	0	4	
472	1	0.1	0	4	
487	1	0.111111111111111	0	4	
488	1	0.125	0	4	
493	1	0.111111111111111	0	4	
512	1	0.142857142857143	0	4	
532	1	0.111111111111111	0	4	
539	1	0.125	0	4	
596	1	0.2	0	4	
640	1	0.1	0	4	
653	2	0.2	0	2	
680	1	0.125	0	4	
928	1	0.166666666666667	0	4	
954	1	0.333333333333333	0	4	<<<
1006	1	0.166666666666667	0	4	
1039	1	0.5	0	4	
1217	1	0.2	0	4	
1301	1	0.1	0	4	
1307	1	0.1	0	4	
1314	1	0.1	0	4	
1315	1	0.1	0	4	
1377	1	0.111111111111111	0	4	
1381	1	0.1	0	4	
1387	2	0.2	0	2	
1390	1	0.1	0	4	
1393	1	0.1	0	4	
1406	1	0.125	0	4	
1407	1	0.125	0	4	
1413	1	0.125	0	4	
1429	1	0.125	0	4	
1447	1	0.25	0	4	
1460	1	0.1	0	4	
1528	1	0.1	0	4	
1529	1	0.1	0	4	
1533	1	0.1	0	4	
1571	1	0.333333333333333	0	4	
1587	1	0.2	0	4	
1654	1	0.1	0	4	
1715	2	0.2	0	2	
1804	1	0.142857142857143	0	4	
1879	1	0.1	0	4	
1897	1	0.1	0	4	
1940	1	0.1	0	4	
1968	1	0.1	0	4	

1972	1	0.1	0	4
1979	1	0.1	0	4
2039	2	0.2	0	2
2066	1	0.1	0	4
2070	1	0.1	0	4
2145	1	0.125	0	4
2228	1	0.111111111111111	0	4
2354	1	0.1	0	4
2419	2	0.2	0	2
2424	2	0.2	0	2
2425	2	0.2	0	2
2469	1	0.111111111111111	0	4
2498	1	0.111111111111111	0	4
2509	1	0.1	0	4
2520	1	0.142857142857143	0	4
2524	1	0.125	0	4
2530	1	0.1	0	4
2536	1	0.1	0	4
2542	2	0.2	0	2
2544	1	0.1	0	4
2545	1	0.1	0	4
2547	1	0.1	0	4
2603	1	0.1	0	4
2611	1	0.1	0	4
2617	1	0.142857142857143	0	4
2632	1	0.166666666666667	0	4
2648	1	0.142857142857143	0	4
2732	1	0.111111111111111	0	4
2733	1	0.111111111111111	0	4
2801	1	0.1	0	4
2802	1	0.1	0	4
2805	2	0.211111111111111	0	2
2808	1	0.1	0	4
2812	1	0.1	0	4
2813	1	0.1	0	4
2817	2	0.2	0	2
2819	1	0.1	0	4
2822	1	0.1	0	4
2829	1	0.1	0	4
2831	1	0.1	0	4
2852	1	0.2	0	4
2964	1	0.333333333333333	0	4
2975	1	0.333333333333333	0	4
2989	1	0.1	0	4
3009	2	0.2	0	2

---

category=2, cleavage_site=1437
query=pto-miR105, target=Potri.T024700.1,
score=3.5, range=1426-1446, strand=1

```
target  5' CUGUGUCUCAAgAUUUCAGCA 3'
            :.::::::: ::::::::.::
query   3' GGCACAGAGCUCUAAAGUUGU 5'
```

>Potri.T024700.1
#size=3728

138	1	0.25	0	4
276	1	0.333333333333333	0	4
307	1	0.25	0	4
318	1	0.2	0	4

375	1	0.2	0	4		
474	1	0.1	0	4		
477	1	0.1	0	4		
478	1	0.111111111111111			0	4
519	1	0.1	0	4		
522	1	0.1	0	4		
621	1	0.25	0	4		
752	1	0.111111111111111			0	4
914	1	0.125	0	4		
931	1	0.125	0	4		
932	1	0.125	0	4		
992	1	0.166666666666667			0	4
1045	1	0.25	0	4		
1050	1	0.333333333333333			0	4
1213	1	0.1	0	4		
1342	1	0.1	0	4		
1344	1	0.1	0	4		
1430	1	0.1	0	4		
1437	2	0.2	0	2	<<<	
1551	1	0.1	0	4		
1552	1	0.1	0	4		
1643	2	1	0	1		
1768	1	0.2	0	4		
1819	1	0.142857142857143			0	4
1827	3	0.325	0	2		
1935	1	1	1	4		
1956	1	0.5	0	4		
2056	2	0.2	0	2		
2060	1	0.2	0	4		
2063	1	0.1	0	4		
2070	2	0.2	0	2		
2072	1	0.1	0	4		
2161	1	0.1	0	4		
2205	1	1	1	4		
2295	1	0.1	0	4		
2315	2	0.2	0	2		
2321	1	0.111111111111111			0	4
2324	1	0.1	0	4		
2399	1	0.1	0	4		
2402	1	0.1	0	4		
2462	1	0.1	0	4		
2467	1	0.1	0	4		
2514	1	0.1	0	4		
2538	1	0.1	0	4		
2551	1	0.1	0	4		
2743	1	0.1	0	4		
2744	1	0.1	0	4		
2822	1	0.1	0	4		
2823	2	0.2	0	2		
2833	1	0.1	0	4		
2892	1	0.1	0	4		
3098	1	0.1	0	4		
3102	1	0.1	0	4		
3104	1	0.1	0	4		
3121	1	0.1	0	4		
3188	1	0.1	0	4		
3190	1	0.1	0	4		
3215	1	0.1	0	4		

3226	1	0.5	0	4	
3227	2	1	0	1	
3303	1	0.125	0	4	
3380	1	0.1	0	4	
3381	1	0.1	0	4	
3428	1	0.142857142857143	0	4	
3485	1	0.1	0	4	
3488	1	0.1	0	4	
3594	1	0.1	0	4	
3654	1	0.1	0	4	
3668	1	0.1	0	4	
3670	1	0.1	0	4	
3675	1	0.1	0	4	

---

category=2, cleavage_site=1203
query=pto-miR105, target=Potri.T024900.1,
score=3.5, range=1192-1212, strand=1

```
target  5' CUGUGUCUCAAgAUUUCAGCA 3'
           :.::::::: :::::::.::
query   3' GGCACAGAGCUCUAAAGUUGU 5'
```

>Potri.T024900.1
#size=3263

142	1	0.25	0	4	
280	1	0.333333333333333	0	4	
311	1	0.25	0	4	
322	1	0.2	0	4	
478	1	0.1	0	4	
481	1	0.1	0	4	
482	1	0.111111111111111	0	4	
765	1	0.111111111111111	0	4	
927	1	0.125	0	4	
979	1	0.1	0	4	
1002	1	0.111111111111111	0	4	
1097	1	0.166666666666667	0	4	
1108	1	0.1	0	4	
1110	13	1.86666666666667	0	0	
1196	1	0.1	0	4	
1203	2	0.2	0	2	<<<
1239	1	0.1	0	4	
1318	1	0.1	0	4	
1389	1	0.1	0	4	
1411	1	0.125	0	4	
1495	1	0.1	0	4	
1498	1	0.1	0	4	
1574	1	0.1	0	4	
1626	1	0.1	0	4	
1634	1	0.1	0	4	
1652	1	0.1	0	4	
1654	1	0.1	0	4	
1655	1	0.1	0	4	
1656	1	0.166666666666667	0	4	
1658	1	0.1	0	4	
1680	1	0.125	0	4	
1684	1	0.1	0	4	
1686	3	0.3	0	2	
1687	1	0.1	0	4	
1698	1	0.1	0	4	
1699	1	0.1	0	4	

1704	1	0.1	0	4
1709	2	0.2	0	2
1715	1	0.1	0	4
1717	1	0.1	0	4
1722	1	0.1	0	4
1783	1	0.1	0	4
1785	1	0.142857142857143	0	4
1797	2	0.2	0	2
1799	1	0.1	0	4
1845	1	0.1	0	4
1852	1	0.1	0	4
1863	1	0.1	0	4
1877	1	0.1	0	4
1879	1	0.1	0	4
1946	2	0.285714285714286	0	2
1962	1	0.1	0	4
1973	1	0.1	0	4
2023	1	0.1	0	4
2025	1	0.1	0	4
2054	1	0.1	0	4
2093	1	0.1	0	4
2097	1	0.1	0	4
2460	1	0.1	0	4
2473	1	0.1	0	4
2474	1	0.1	0	4
2559	1	0.1	0	4
2573	1	0.1	0	4
2574	1	0.1	0	4
2606	1	0.1	0	4
2617	1	0.1	0	4
2822	1	0.1	0	4
2850	1	0.5	0	4
2905	1	0.1	0	4
2906	1	0.1	0	4
2908	1	0.1	0	4
2911	2	0.2	0	2
2914	1	0.1	0	4
2922	1	0.1	0	4
2948	1	0.1	0	4
2949	1	0.1	0	4
3030	1	0.1	0	4
3097	1	0.1	0	4
3098	1	0.1	0	4
3116	1	0.1	0	4
3195	1	0.125	0	4
3197	1	0.1	0	4

---

category=2, cleavage_site=118
query=pto-miR105, target=Potri.T025500.1,
score=3, range=107-127, strand=1

```
target  5' CCGUGUCUCAAgAUUUCAGCA 3'
           ::::::::: ::::::::.::
query   3' GGCACAGAGCUCUAAAGUUGU 5'
```

>Potri.T025500.1
#size=1623

111	1	0.1	0	4	
118	2	0.2	0	2	<<<
203	1	0.1	0	4	

231	1	0.1	0	4
232	1	0.1	0	4
233	1	0.1	0	4
410	1	0.1	0	4
413	1	0.1	0	4
417	1	0.1	0	4
434	1	0.1	0	4
446	1	0.2	0	4
461	1	0.142857142857143	0	4
488	1	0.111111111111111	0	4
503	1	0.1	0	4
509	1	0.1	0	4
547	1	0.1	0	4
549	1	0.1	0	4
616	1	0.166666666666667	0	4
677	1	0.1	0	4
696	1	0.2	0	4
698	1	0.142857142857143	0	4
702	1	0.1	0	4
709	1	0.125	0	4
711	1	0.1	0	4
718	2	0.2	0	2
720	1	0.1	0	4
784	1	0.1	0	4
787	1	0.1	0	4
792	1	0.1	0	4
806	1	0.1	0	4
873	1	0.1	0	4
896	1	0.333333333333333	0	4
927	1	0.1	0	4
969	1	0.111111111111111	0	4
1032	1	0.1	0	4
1047	1	0.1	0	4
1230	1	0.142857142857143	0	4
1468	1	0.1	0	4
1471	1	0.1	0	4
1488	1	0.1	0	4
1490	1	0.1	0	4
1522	2	0.666666666666667	0	0

---

category=4, cleavage_site=199
query=pto-miR105, target=Potri.T025800.1,
score=3, range=188-208, strand=1

```
target  5' CCGUGUCUCAAgAUUUCAGCA 3'
            ::::::::: :::::::.::
query   3' GGCACAGAGCUCUAAAGUUGU 5'
```

>Potri.T025800.1
#size=3056

104	1	0.1	0	4	
106	3	1.26666666666667	1	0	
199	1	0.333333333333333	0	4	<<<
314	1	0.1	0	4	
400	1	0.1	0	4	
433	1	0.125	0	4	
559	1	0.1	0	4	
564	1	0.1	0	4	
570	1	0.1	0	4	
584	1	0.1	0	4	

590	1	0.1	0	4
639	1	0.125	0	4
651	1	0.1	0	4
653	1	0.1	0	4
660	1	0.125	0	4
663	1	0.5	0	4
664	2	0.2	0	2
665	1	0.1	0	4
666	1	0.1	0	4
717	1	0.1	0	4
719	1	0.1	0	4
724	1	0.1	0	4
758	1	0.1	0	4
815	1	0.333333333333333	0	4
823	1	0.125	0	4
866	1	0.1	0	4
881	1	0.1	0	4
889	1	0.2	0	4
890	1	0.1	0	4
892	1	0.2	0	4
899	1	0.1	0	4
900	1	0.1	0	4
964	1	0.1	0	4
968	1	0.1	0	4
975	1	0.1	0	4
1025	1	0.1	0	4
1027	1	0.1	0	4
1056	1	0.1	0	4
1099	1	0.1	0	4
1194	1	0.1	0	4
1246	1	0.1	0	4
1475	1	0.1	0	4
1476	1	0.1	0	4
1615	1	0.1	0	4
1622	1	0.111111111111111	0	4
1625	1	0.1	0	4
1632	1	0.1	0	4
1634	1	0.1	0	4
1684	1	0.1	0	4
1712	1	0.166666666666667	0	4
1717	1	0.333333333333333	0	4
1894	1	0.1	0	4
1896	1	0.1	0	4
1928	1	0.1	0	4
1951	1	0.5	0	4
1969	1	0.333333333333333	0	4
1985	1	0.1	0	4
1988	1	0.1	0	4
2007	1	0.1	0	4
2029	1	0.25	0	4
2045	1	0.333333333333333	0	4
2049	1	1	1	4
2095	1	0.1	0	4
2300	1	0.1	0	4
2356	1	0.333333333333333	0	4
2394	1	0.1	0	4
2482	1	0.1	0	4
2556	1	0.1	0	4

2557	1	0.1	0	4
2559	1	0.1	0	4
2563	1	0.1	0	4
2564	1	0.1	0	4
2577	1	0.1	0	4
2578	1	0.1	0	4
2581	1	0.125	0	4
2591	1	0.1	0	4
2597	2	0.2	0	2
2599	1	0.111111111111111	0	4
2606	1	0.125	0	4
2753	1	0.1	0	4
2756	1	0.1	0	4
2842	1	0.1	0	4
2847	1	0.1	0	4
2849	1	0.1	0	4
2854	1	0.1	0	4

category=4, cleavage_site=1093
query=pto-miR105, target=Potri.T025900.1,
score=2.5, range=1082-1102, strand=1

```
target  5' CUGUGUCUCAAgAUUUCAACA 3'
            :.::::::: ::::::::::
query   3' GGCACAGAGCUCUAAAGUUGU 5'
```

>Potri.T025900.1
#size=2025

10	1	0.2	0	4	
112	1	0.1	0	4	
154	1	0.1	0	4	
157	1	0.1	0	4	
180	1	0.1	0	4	
396	1	0.111111111111111	0	4	
558	1	0.125	0	4	
575	1	0.125	0	4	
576	1	0.125	0	4	
727	2	0.375	0	2	
740	1	0.111111111111111	0	4	
741	1	0.111111111111111	0	4	
745	1	0.125	0	4	
892	1	0.111111111111111	0	4	
907	1	0.1	0	4	
1000	17	4.95	0	0	
1093	1	0.333333333333333	0	4	<<<
1221	2	1	0	2	
1283	1	0.1	0	4	
1318	1	0.125	0	4	
1329	1	0.142857142857143	0	4	
1331	6	1.1	0	2	
1366	2	0.2	0	2	
1380	1	0.1	0	4	
1454	1	0.333333333333333	0	4	
1467	1	0.1	0	4	
1469	1	0.1	0	4	
1476	1	0.125	0	4	
1492	1	0.125	0	4	
1496	1	0.1	0	4	
1498	3	0.3	0	2	
1499	1	0.1	0	4	

1549	1	0.125	0	4
1551	1	0.1	0	4
1558	2	0.2	0	2
1560	1	0.1	0	4
1627	1	0.1	0	4
1648	1	0.2	0	4
1651	1	0.2	0	4
1657	1	0.333333333333333	0	4
1681	1	0.333333333333333	0	4
1685	1	0.2	0	4

---

category=2, cleavage_site=583
query=pto-miR105, target=Potri.T026800.1,
score=3.5, range=572-592, strand=1

```
target  5' CUGUGUCUCAAgAUUUCAGCA 3'
           :.::::::: :::::::.::
query   3' GGCACAGAGCUCUAAAGUUGU 5'
```

>Potri.T026800.1
#size=2859

48	1	0.125	0	4	
65	1	0.125	0	4	
66	1	0.125	0	4	
110	1	0.1	0	4	
119	1	0.125	0	4	
138	1	0.333333333333333	0	4	
170	1	0.333333333333333	0	4	
205	1	1	1	4	
217	2	0.375	0	2	
230	1	0.111111111111111	0	4	
231	1	0.111111111111111	0	4	
235	1	0.125	0	4	
268	1	0.25	0	4	
382	1	0.111111111111111	0	4	
397	1	0.1	0	4	
490	10	4.63333333333333	3	0	
499	1	0.333333333333333	0	4	
576	1	0.1	0	4	
583	2	0.2	0	2	<<<
631	1	1	1	4	
668	1	0.1	0	4	
842	2	0.285714285714286	0	2	
851	2	0.242857142857143	0	2	
857	1	0.1	0	4	
859	1	0.1	0	4	
897	1	0.142857142857143	0	4	
899	1	0.1	0	4	
1061	1	0.25	0	4	
1085	1	0.5	0	4	
1249	1	0.1	0	4	
1263	1	0.1	0	4	
1271	1	0.1	0	4	
1283	1	0.1	0	4	
1431	2	0.2	0	2	
1437	1	0.111111111111111	0	4	
1440	1	0.1	0	4	
1479	1	0.1	0	4	
1578	1	0.1	0	4	
1583	1	0.1	0	4	

1630	1	0.1	0	4
1698	1	0.142857142857143	0	4
1803	1	0.1	0	4
1859	1	0.1	0	4
1939	1	0.1	0	4
1946	1	0.111111111111111	0	4
1949	1	0.1	0	4
2008	1	0.1	0	4
2015	1	0.1	0	4
2028	1	0.1	0	4
2036	1	0.1	0	4
2177	1	0.1	0	4
2190	1	0.25	0	4
2218	1	0.1	0	4
2220	1	0.1	0	4
2237	1	0.1	0	4
2252	1	0.1	0	4
2320	1	0.1	0	4
2331	1	0.1	0	4
2342	1	0.5	0	4
2343	2	1	0	2
2369	1	0.333333333333333	0	4
2414	1	0.333333333333333	0	4
2417	1	0.166666666666667	0	4
2419	1	0.1	0	4
2423	1	0.1	0	4
2425	1	0.1	0	4
2428	1	0.1	0	4
2444	1	0.111111111111111	0	4
2496	1	0.1	0	4
2497	1	0.1	0	4
2502	1	0.1	0	4
2515	1	0.1	0	4
2528	1	0.1	0	4
2534	2	0.2	0	2
2536	1	0.111111111111111	0	4
2543	1	0.125	0	4
2682	1	0.333333333333333	0	4

---

category=4, cleavage_site=340
query=pto-miR105, target=Potri.T028100.1,
score=2.5, range=329-349, strand=1

```
target  5' CUGUGUCUCAAgAUUUCAACA 3'
           :.:::::::: ::::::::::
query   3' GGCACAGAGCUCUAAAGUUGU 5'
```

>Potri.T028100.1
#size=2415

152	1	0.1	0	4	
175	1	0.111111111111111	0	4	
190	1	0.1	0	4	
220	1	0.125	0	4	
241	1	0.1	0	4	
340	1	0.333333333333333	0	4	<<<
541	1	0.1	0	4	
548	1	0.125	0	4	
614	1	0.1	0	4	
616	1	0.1	0	4	
618	1	0.142857142857143	0	4	

637	2	0.25	0	2
642	1	0.333333333333333	0	4
643	1	0.125	0	4
654	1	0.142857142857143	0	4
656	5	0.766666666666667	0	2
658	1	0.333333333333333	0	4
683	1	0.142857142857143	0	4
691	3	0.325	0	2
711	1	0.1	0	4
782	2	0.2	0	2
784	1	0.1	0	4
789	1	0.1	0	4
791	1	0.1	0	4
821	1	0.1	0	4
823	2	0.2	0	2
824	1	0.1	0	4
835	1	0.1	0	4
836	1	0.1	0	4
841	1	0.1	0	4
846	2	0.2	0	2
852	1	0.1	0	4
854	1	0.1	0	4
859	1	0.1	0	4
1031	1	0.1	0	4
1033	1	0.1	0	4
1053	2	0.2	0	2
1062	1	0.1	0	4
1448	1	1	1	4
1462	2	0.2	0	2
1469	1	0.111111111111111	0	4
1472	1	0.1	0	4
1479	1	0.1	0	4
1480	1	0.1	0	4
1481	1	0.1	0	4
1737	1	0.1	0	4
1767	2	0.2	0	2
1775	2	0.225	0	2
1855	1	0.111111111111111	0	4
1861	1	0.1	0	4
1869	1	0.1	0	4
1870	1	0.1	0	4
1872	1	0.1	0	4
1873	1	0.1	0	4
1942	1	0.1	0	4
1967	1	0.111111111111111	0	4
1970	1	0.142857142857143	0	4
2016	2	0.2	0	2
2017	1	0.1	0	4
2038	1	0.1	0	4
2057	2	0.2	0	2
2124	1	0.1	0	4
2127	1	0.1	0	4
2190	1	0.1	0	4
2213	1	0.1	0	4
2216	1	0.1	0	4
2297	1	0.125	0	4
2298	1	0.125	0	4
2304	1	0.1	0	4

2311	1	0.1	0	4	

category=2, cleavage_site=1354
query=pto-miR105, target=Potri.T029000.1,
score=3.5, range=1343-1363, strand=1

```
target  5' CUGUGUCUCAAgAUUUCAGCA 3'
           :.::::::: :::::::::.::
query   3' GGCACAGAGCUCUAAAGUUGU 5'
```

>Potri.T029000.1
#size=4001

324	1	0.111111111111111	0	4	
328	1	0.1	0	4	
331	1	0.1	0	4	
370	1	0.111111111111111	0	4	
373	1	0.1	0	4	
376	1	0.1	0	4	
526	1	0.25	0	4	
657	1	0.111111111111111	0	4	
836	1	0.125	0	4	
881	1	0.1	0	4	
890	1	0.125	0	4	
941	1	0.333333333333333	0	4	
988	1	0.125	0	4	
1001	1	0.111111111111111	0	4	
1002	1	0.111111111111111	0	4	
1006	1	0.125	0	4	
1248	1	0.166666666666667	0	4	
1261	6	0.6	0	2	
1347	1	0.1	0	4	
1354	2	0.2	0	2	<<<
1390	1	0.1	0	4	
1439	1	0.1	0	4	
1467	1	0.1	0	4	
1469	1	0.1	0	4	
1531	1	0.25	0	4	
1540	1	0.1	0	4	
1562	1	0.125	0	4	
1588	1	0.125	0	4	
1593	1	0.333333333333333	0	4	
1613	2	0.285714285714286	0	2	
1622	2	0.242857142857143	0	2	
1628	1	0.1	0	4	
1630	1	0.1	0	4	
1632	1	0.142857142857143	0	4	
1646	1	0.1	0	4	
1651	2	0.25	0	2	
1657	1	0.125	0	4	
1668	1	0.142857142857143	0	4	
1670	6	1.1	0	0	
1739	1	0.1	0	4	
1745	1	0.1	0	4	
1774	1	0.2	0	4	
1777	1	0.1	0	4	
1784	1	0.1	0	4	
1785	1	0.1	0	4	
1787	2	0.2	0	2	
1796	1	0.1	0	4	
1798	1	0.1	0	4	

1803	1	0.1	0	4	
1805	1	0.1	0	4	
1807	1	0.333333333333333	0	4	
1809	1	0.1	0	4	
1819	2	0.2	0	2	
1820	1	0.1	0	4	
1821	1	0.1	0	4	
1832	1	0.25	0	4	
1970	1	0.333333333333333	0	4	
2028	1	0.1	0	4	
2036	1	0.1	0	4	
2045	1	0.1	0	4	
2081	1	0.2	0	4	
2180	1	0.1	0	4	
2250	1	0.166666666666667	0	4	
2438	1	0.1	0	4	
2732	1	0.1	0	4	
2739	1	0.1	0	4	
2741	1	0.1	0	4	
2797	1	0.1	0	4	
2874	1	0.333333333333333	0	4	
3145	1	0.1	0	4	
3155	1	0.111111111111111	0	4	
3282	1	0.1	0	4	
3283	1	0.1	0	4	
3286	1	0.1	0	4	

category=2, cleavage_site=1354
query=pto-miR105, target=Potri.T052000.1,
score=3.5, range=1343-1363, strand=1

```
target  5' CUGUGUCUCAAgAUUUCAGCA 3'
           :.::::::: ::::::::.::
query   3' GGCACAGAGCUCUAAAGUUGU 5'
```

>Potri.T052000.1
#size=3480

324	1	0.111111111111111	0	4	
328	1	0.1	0	4	
331	1	0.1	0	4	
370	1	0.111111111111111	0	4	
373	1	0.1	0	4	
376	1	0.1	0	4	
526	1	0.25	0	4	
657	1	0.111111111111111	0	4	
836	1	0.125	0	4	
881	1	0.1	0	4	
890	1	0.125	0	4	
941	1	0.333333333333333	0	4	
988	1	0.125	0	4	
1001	1	0.111111111111111	0	4	
1002	1	0.111111111111111	0	4	
1006	1	0.125	0	4	
1219	1	0.1	0	4	
1261	6	0.6	0	2	
1347	1	0.1	0	4	
1354	2	0.2	0	2	<<<
1390	1	0.1	0	4	
1439	1	0.1	0	4	
1467	1	0.1	0	4	

1469	1	0.1	0	4	
1531	1	0.25	0	4	
1540	1	0.1	0	4	
1562	1	0.125	0	4	
1588	1	0.125	0	4	
1593	1	0.333333333333333	0	4	
1613	2	0.285714285714286	0	2	
1622	2	0.242857142857143	0	2	
1628	1	0.1	0	4	
1630	1	0.1	0	4	
1632	1	0.142857142857143	0	4	
1646	1	0.1	0	4	
1651	2	0.25	0	2	
1657	1	0.125	0	4	
1668	1	0.142857142857143	0	4	
1670	6	1.1	0	0	
1739	1	0.1	0	4	
1745	1	0.1	0	4	
1774	1	0.2	0	4	
1777	1	0.1	0	4	
1784	1	0.1	0	4	
1785	2	0.2	0	2	
1787	2	0.2	0	2	
1796	2	0.2	0	2	
1798	1	0.1	0	4	
1803	1	0.1	0	4	
1805	1	0.1	0	4	
1807	1	0.333333333333333	0	4	
1809	1	0.1	0	4	
1819	2	0.2	0	2	
1820	1	0.1	0	4	
1821	1	0.1	0	4	
1832	1	0.25	0	4	
1940	1	0.1	0	4	
1970	1	0.333333333333333	0	4	
2028	1	0.1	0	4	
2036	1	0.1	0	4	
2045	1	0.1	0	4	
2081	1	0.2	0	4	
2250	1	0.166666666666667	0	4	
2271	1	0.1	0	4	
2289	1	0.1	0	4	
2438	1	0.1	0	4	
2732	1	0.1	0	4	
2739	1	0.1	0	4	
2741	1	0.1	0	4	
2797	1	0.1	0	4	
2874	1	0.333333333333333	0	4	
3162	2	0.2	0	2	
3163	1	0.1	0	4	
3166	1	0.1	0	4	

category=2, cleavage_site=913
query=pto-miR105, target=Potri.T053000.1,
score=3.5, range=902-922, strand=1

```
target  5' CUGUGUCUCAAgAUUUCAGCA 3'
            :.::::::: :::::::.::
query   3' GGCACAGAGCUCUAAAGUUGU 5'
```

>Potri.T053000.1
#size=3102

84	1	0.333333333333333	0	4	
128	1	0.333333333333333	0	4	
165	1	1	1	4	
173	2	2	2	0	
178	1	1	1	4	
192	1	1	1	4	
193	1	1	1	4	
207	1	0.25	0	4	
211	1	0.111111111111111	0	4	
226	1	0.2	0	4	
284	1	0.111111111111111	0	4	
288	1	0.5	0	4	
291	1	0.111111111111111	0	4	
322	1	0.142857142857143	0	4	
329	1	0.111111111111111	0	4	
333	2	0.222222222222222	0	2	
334	1	0.111111111111111	0	4	
336	1	0.111111111111111	0	4	
342	1	0.142857142857143	0	4	
377	1	0.166666666666667	0	4	
392	1	0.125	0	4	
403	1	0.142857142857143	0	4	
411	1	0.111111111111111	0	4	
415	1	0.111111111111111	0	4	
435	1	0.2	0	4	
438	1	0.111111111111111	0	4	
453	1	0.111111111111111	0	4	
469	1	0.111111111111111	0	4	
487	1	0.142857142857143	0	4	
505	2	0.4	0	2	
547	1	0.333333333333333	0	4	
558	1	0.333333333333333	0	4	
689	1	0.1	0	4	
712	1	0.111111111111111	0	4	
727	1	0.1	0	4	
757	1	0.125	0	4	
807	1	0.166666666666667	0	4	
820	2	0.2	0	2	
906	1	0.1	0	4	
913	2	0.2	0	2	<<<
949	1	0.1	0	4	
975	1	0.5	0	4	
1027	1	0.1	0	4	
1115	1	0.1	0	4	
1133	1	0.1	0	4	
1136	1	0.1	0	4	
1138	2	0.25	0	2	
1140	1	0.1	0	4	
1143	1	0.333333333333333	0	4	
1144	1	0.125	0	4	
1157	1	0.1	0	4	
1159	1	0.333333333333333	0	4	
1261	1	0.2	0	4	
1264	1	0.1	0	4	
1271	1	0.1	0	4	
1272	1	0.1	0	4	

1274	2	0.2	0	2
1283	2	0.2	0	2
1285	1	0.1	0	4
1290	1	0.1	0	4
1292	1	0.1	0	4
1294	1	0.333333333333333	0	4
1296	1	0.1	0	4
1318	1	0.125	0	4
1321	1	0.5	0	4
1322	1	0.1	0	4
1324	3	0.3	0	2
1325	1	0.1	0	4
1336	1	0.1	0	4
1459	1	0.125	0	4
1475	1	0.125	0	4
1483	1	0.1	0	4
1490	1	0.1	0	4
1507	1	0.2	0	4
1584	2	0.285714285714286	0	2
1600	1	0.1	0	4
1611	1	0.1	0	4
1647	1	0.1	0	4
1731	1	0.1	0	4
1882	1	0.1	0	4
1999	1	0.111111111111111	0	4
2014	2	0.666666666666667	0	2
2191	1	0.1	0	4
2283	1	0.1	0	4
2435	1	0.1	0	4
2448	1	0.25	0	4
2472	1	0.1	0	4
2476	1	0.1	0	4
2495	1	0.1	0	4
2590	1	0.111111111111111	0	4
2596	1	0.1	0	4
2604	1	0.1	0	4
2605	1	0.1	0	4
2607	1	0.1	0	4
2608	1	0.1	0	4
2667	1	0.25	0	4
2677	1	0.125	0	4
2754	2	0.2	0	2
2758	1	0.1	0	4
2759	1	0.1	0	4
2772	1	0.1	0	4
2776	1	0.125	0	4
2786	1	0.1	0	4
2792	2	0.2	0	2
2794	1	0.111111111111111	0	4
2801	1	0.125	0	4
2859	1	0.1	0	4
2862	1	0.1	0	4
2924	3	0.3	0	2
2925	1	0.1	0	4
2929	1	0.1	0	4
2932	1	0.1	0	4
2948	1	0.1	0	4
2968	1	0.1	0	4

3037	1	0.1	0	4
3044	1	0.1	0	4

pto-miR107

category=4, cleavage_site=949
query= pto-miR107, target=Potri.001G197400.1,
score=3, range=938-958, strand=1

```
target  5' UCACUUAAAUGcCACAUAUCA 3'
           ::  :: ::::::::::::::
query   3' AGCUAACUUACGGUGUAUAGU 5'
```

>Potri.001G197400.1
#size=2272

108	2	0.5	0	0
121	1	0.25	0	4
139	1	0.25	0	4
142	1	0.25	0	4
146	1	0.25	0	4
171	1	0.25	0	4
213	1	0.1	0	4
223	1	0.1	0	4
229	1	0.1	0	4
233	1	0.1	0	4
236	1	0.2	0	4
239	1	0.2	0	4
242	1	0.2	0	4
255	1	0.2	0	4
260	1	0.2	0	4
277	1	0.1	0	4
306	1	0.1	0	4
318	1	0.2	0	4
332	1	0.2	0	4
334	2	0.4	0	2
347	1	0.2	0	4
348	1	0.2	0	4
350	1	0.2	0	4
355	1	0.2	0	4
356	1	0.1	0	4
359	1	0.1	0	4
361	1	0.2	0	4
366	1	0.2	0	4
383	1	0.2	0	4
400	1	0.2	0	4
408	1	0.1	0	4
434	1	0.1	0	4
551	1	0.2	0	4
624	1	0.1	0	4
628	1	0.2	0	4
635	1	0.1	0	4
636	2	0.2	0	2
638	1	0.1	0	4
641	1	0.1	0	4
642	2	0.2	0	2
645	1	0.1	0	4
652	3	0.3	0	2
653	1	0.1	0	4
654	1	0.1	0	4

656	1	0.1	0	4	
657	1	0.1	0	4	
765	1	0.1	0	4	
852	1	0.1	0	4	
860	1	0.1	0	4	
883	1	0.1	0	4	
885	2	0.2	0	2	
901	1	0.1	0	4	
922	1	0.1	0	4	
924	1	0.2	0	4	
949	1	0.1	0	4	<<<
951	1	0.1	0	4	
956	1	0.1	0	4	
963	1	0.1	0	4	
964	1	0.1	0	4	
965	2	0.2	0	2	
968	1	0.1	0	4	
971	3	0.3	0	2	
972	4	0.4	0	2	
976	1	0.1	0	4	
978	1	0.1	0	4	
990	1	0.1	0	4	
992	1	0.1	0	4	
1027	1	0.2	0	4	
1038	1	0.2	0	4	
1039	1	0.1	0	4	
1042	1	0.1	0	4	
1045	2	0.2	0	2	
1049	1	0.1	0	4	
1054	1	0.1	0	4	
1060	1	0.1	0	4	
1061	1	0.1	0	4	
1063	1	0.1	0	4	
1071	1	0.1	0	4	
1075	1	0.1	0	4	
1081	1	0.1	0	4	
1088	1	0.1	0	4	
1089	1	0.1	0	4	
1090	1	0.1	0	4	
1091	1	0.1	0	4	
1098	1	0.1	0	4	
1106	1	0.1	0	4	
1109	3	0.3	0	2	
1113	1	0.1	0	4	
1120	1	0.1	0	4	
1125	1	0.1	0	4	
1126	1	0.1	0	4	
1127	1	0.1	0	4	
1128	2	0.2	0	2	
1132	1	0.1	0	4	
1142	1	0.1	0	4	
1143	1	0.1	0	4	
1147	1	0.1	0	4	
1149	1	0.25	0	4	
1176	1	0.2	0	4	
1191	1	0.1	0	4	
1201	1	0.1	0	4	
1209	1	0.1	0	4	

1219	1	0.1	0	4
1239	1	0.1	0	4
1268	1	0.2	0	4
1278	1	0.2	0	4
1280	2	0.4	0	2
1320	1	0.2	0	4
1322	2	0.4	0	2
1340	1	0.2	0	4
1346	1	0.2	0	4
1388	1	0.1	0	4
1389	2	0.2	0	2
1392	1	0.1	0	4
1393	1	0.1	0	4
1415	1	0.1	0	4
1433	3	0.3	0	2
1435	1	0.1	0	4
1438	1	0.1	0	4
1439	1	0.1	0	4
1440	1	0.1	0	4
1442	1	0.1	0	4
1443	2	0.2	0	2
1447	1	0.1	0	4
1452	1	0.1	0	4
1453	1	0.1	0	4
1460	1	0.1	0	4
1463	2	0.2	0	2
1464	1	0.1	0	4
1466	1	0.1	0	4
1469	1	0.1	0	4
1474	1	0.1	0	4
1478	1	0.1	0	4
1480	1	0.1	0	4
1482	1	0.1	0	4
1483	1	0.1	0	4
1485	2	0.2	0	2
1486	1	0.1	0	4
1489	2	0.4	0	2
1491	1	0.2	0	4
1499	1	0.2	0	4
1509	1	0.2	0	4
1521	1	0.1	0	4
1530	1	0.1	0	4
1532	1	0.1	0	4
1535	1	0.1	0	4
1537	1	0.1	0	4
1538	1	0.1	0	4
1540	4	0.4	0	2
1545	1	0.1	0	4
1548	1	0.1	0	4
1551	1	0.1	0	4
1559	1	0.1	0	4
1570	1	0.2	0	4
1591	1	0.2	0	4
1597	2	0.4	0	2
1604	1	0.2	0	4
1616	1	0.2	0	4
1635	1	0.2	0	4
1637	1	0.2	0	4

1638	1	0.2	0	4
1693	1	0.2	0	4
1715	1	0.2	0	4
1987	1	0.25	0	4

pto-miR143

category=4, cleavage_site=2672
query= pto-miR143, target=Potri.014G001900.1,
score=0.5, range=2661-2681, strand=1

```
target  5' UCAUGGCUUCAcGUCUCUUCG 3'
           ::::::::::::::::::::.
query   3' AGUACCGAAGUGCAGAGAAGU 5'
```

>Potri.014G001900.1
#size=3567

5	1	0.1	0	4
88	1	0.111111111111111	0	4
256	1	0.1	0	4
267	2	0.2	0	2
273	1	0.1	0	4
282	1	0.1	0	4
283	1	0.111111111111111	0	4
284	2	0.222222222222222	0	2
294	1	0.2	0	4
311	1	0.25	0	4
374	1	0.1	0	4
385	1	0.111111111111111	0	4
397	1	0.1	0	4
401	1	0.1	0	4
425	1	0.1	0	4
518	1	0.1	0	4
520	1	0.1	0	4
529	1	0.1	0	4
534	1	0.1	0	4
545	1	0.1	0	4
553	2	0.2	0	2
602	1	0.1	0	4
609	1	0.1	0	4
620	2	0.2	0	2
630	1	0.333333333333333	0	4
686	8	0.8	0	2
687	1	0.1	0	4
697	4	0.4	0	2
753	1	0.1	0	4
756	1	0.1	0	4
767	1	0.142857142857143	0	4
773	1	0.142857142857143	0	4
780	1	0.1	0	4
783	1	0.1	0	4
790	2	0.2	0	2
799	1	0.1	0	4
837	4	0.4	0	2
838	2	0.2	0	2
839	4	0.4	0	2
852	1	0.1	0	4
854	1	0.1	0	4
856	1	0.1	0	4

857	2	0.2	0	2
858	3	0.3	0	2
859	1	0.1	0	4
860	1	0.1	0	4
864	1	0.1	0	4
868	1	0.1	0	4
871	1	0.1	0	4
872	2	0.3	0	2
891	1	0.1	0	4
892	1	0.1	0	4
893	1	0.1	0	4
898	1	0.1	0	4
909	1	0.1	0	4
1048	1	0.1	0	4
1051	1	0.1	0	4
1059	1	0.1	0	4
1060	1	0.1	0	4
1061	1	0.2	0	4
1062	1	0.1	0	4
1065	1	0.1	0	4
1066	2	0.2	0	2
1067	1	0.166666666666667	0	4
1071	3	0.35	0	2
1072	3	0.336111111111111	0	2
1084	1	0.1	0	4
1109	1	0.142857142857143	0	4
1120	1	0.1	0	4
1126	1	0.1	0	4
1147	1	0.1	0	4
1153	1	0.1	0	4
1154	1	0.166666666666667	0	4
1157	1	0.1	0	4
1160	1	0.1	0	4
1164	1	0.125	0	4
1165	1	0.125	0	4
1206	1	0.2	0	4
1216	1	0.166666666666667	0	4
1219	1	0.166666666666667	0	4
1283	1	0.25	0	4
1292	1	0.1	0	4
1293	1	0.1	0	4
1310	1	0.2	0	4
1345	1	0.1	0	4
1346	1	0.1	0	4
1416	1	0.1	0	4
1434	1	0.1	0	4
1437	1	0.1	0	4
1442	1	0.1	0	4
1444	1	0.1	0	4
1452	2	0.2	0	2
1481	1	0.1	0	4
1490	1	0.1	0	4
1507	1	0.1	0	4
1510	1	0.1	0	4
1511	1	0.1	0	4
1546	1	0.1	0	4
1552	1	0.1	0	4
1554	1	0.1	0	4

1560	2	0.2	0	2
1565	1	0.1	0	4
1568	1	0.1	0	4
1571	1	0.1	0	4
1575	1	0.1	0	4
1625	2	0.2	0	2
1627	1	0.1	0	4
1635	1	0.1	0	4
1642	1	0.1	0	4
1644	1	0.1	0	4
1651	1	0.1	0	4
1673	1	0.1	0	4
1680	1	0.1	0	4
1685	2	0.2	0	2
1688	1	0.1	0	4
1689	1	0.1	0	4
1699	1	0.1	0	4
1704	2	0.2	0	2
1708	2	0.2	0	2
1719	1	0.1	0	4
1722	1	0.1	0	4
1723	1	0.1	0	4
1728	1	0.1	0	4
1730	1	0.1	0	4
1741	1	0.1	0	4
1742	1	0.1	0	4
1745	1	0.1	0	4
1757	1	0.1	0	4
1758	2	0.2	0	2
1765	1	0.1	0	4
1767	1	0.1	0	4
1769	1	0.1	0	4
1780	1	0.125	0	4
1793	1	0.1	0	4
1794	1	0.1	0	4
1796	1	0.1	0	4
1797	1	0.1	0	4
1798	1	0.1	0	4
1799	1	0.1	0	4
1800	1	0.1	0	4
1801	1	0.1	0	4
1802	1	0.1	0	4
1804	3	0.3	0	2
1805	1	0.1	0	4
1806	2	0.2	0	2
1807	2	0.2	0	2
1810	1	0.1	0	4
1811	1	0.1	0	4
1812	1	0.1	0	4
1813	1	0.1	0	4
1815	4	0.4	0	2
1817	2	0.2	0	2
1818	3	0.3	0	2
1820	1	0.1	0	4
1823	1	0.1	0	4
1825	1	0.1	0	4
1826	2	0.2	0	2
1827	2	0.2	0	2

1828	1	0.1	0	4
1829	1	0.1	0	4
1832	1	0.1	0	4
1836	1	0.1	0	4
1837	1	0.1	0	4
1838	1	0.1	0	4
1845	1	0.1	0	4
1846	2	0.2	0	2
1847	1	0.1	0	4
1852	1	0.1	0	4
1857	1	0.1	0	4
1861	1	0.1	0	4
1865	1	0.1	0	4
1868	1	0.1	0	4
1869	1	0.1	0	4
1871	2	0.2	0	2
1873	2	0.2	0	2
1878	2	0.2	0	2
1879	2	0.2	0	2
1882	1	0.1	0	4
1883	1	0.1	0	4
1885	1	0.1	0	4
1886	1	0.1	0	4
1887	1	0.1	0	4
1888	2	0.2	0	2
1889	1	0.1	0	4
1890	1	0.1	0	4
1891	1	0.1	0	4
1893	2	0.2	0	2
1894	2	0.2	0	2
1895	1	0.1	0	4
1897	1	0.1	0	4
1898	1	0.1	0	4
1899	1	0.1	0	4
1901	1	0.1	0	4
1903	1	0.1	0	4
1904	2	0.2	0	2
1905	1	0.1	0	4
1907	2	0.2	0	2
1912	1	0.1	0	4
1913	1	0.1	0	4
1918	1	0.1	0	4
1920	1	0.1	0	4
1921	3	0.3	0	2
1924	1	0.1	0	4
1925	2	0.2	0	2
1928	1	0.1	0	4
1930	1	0.1	0	4
1938	1	0.1	0	4
1941	2	0.2	0	2
1943	2	0.2	0	2
1988	1	0.1	0	4
1990	1	0.1	0	4
1991	1	0.1	0	4
1993	1	0.1	0	4
1994	1	0.1	0	4
1996	1	0.1	0	4
1997	1	0.1	0	4

2002	2	0.2	0	2		
2054	1	0.1	0	4		
2078	1	0.1	0	4		
2140	1	0.1	0	4		
2145	1	0.1	0	4		
2149	1	0.1	0	4		
2367	1	0.1	0	4		
2482	1	0.1	0	4		
2514	2	0.2	0	2		
2515	1	0.1	0	4		
2589	1	0.25	0	4		
2662	1	0.166666666666667			0	4
2672	1	1	1	4	<<<	
2681	1	0.2	0	4		
2687	1	0.142857142857143			0	4
2688	2	1.25	1	0		
2690	1	0.142857142857143			0	4
2692	1	0.2	0	4		
2718	1	0.1	0	4		
2719	1	0.1	0	4		
2879	1	0.142857142857143			0	4
2904	1	0.25	0	4		
2912	1	1	1	4		
2914	1	0.111111111111111			0	4
2919	1	0.166666666666667			0	4
2980	1	0.1	0	4		
2982	1	0.1	0	4		
2989	1	0.1	0	4		
2995	1	0.1	0	4		
3014	1	0.25	0	4		
3123	1	0.1	0	4		
3151	2	0.2	0	2		
3163	1	0.1	0	4		
3200	2	0.5	0	2		
3201	1	0.25	0	4		
3206	1	0.25	0	4		
3221	1	0.25	0	4		
3303	1	0.111111111111111			0	4
3304	1	0.25	0	4		
3309	1	0.142857142857143			0	4
3387	1	0.1	0	4		
3397	1	0.1	0	4		
3399	1	0.1	0	4		
3400	1	0.1	0	4		
3403	1	0.1	0	4		
3472	1	0.111111111111111			0	4
3476	1	0.1	0	4		
3479	1	0.1	0	4		
3481	1	0.1	0	4		
3485	1	0.1	0	4		

pto-miR154

category=3, cleavage_site=1418
query=pto-miR154, target=Potri.001G058600.1,
score=4, range=1407-1427, strand=1

```
target  5' GUGCUCUCUCUcUUCUGUCAU 3'
              :::::::::. ::::::::::
query   3' CACGAGAGGUAGAAGACAGUU 5'
```

>Potri.001G058600.1
#size=2079

177	1	1	1	4			
344	1	1	1	4			
352	1	1	1	4			
863	1	1	1	4			
924	1	0.333333333333333			0	4	
934	1	0.333333333333333			0	4	
966	1	0.333333333333333			0	4	
973	1	0.333333333333333			0	4	
982	1	0.333333333333333			0	4	
984	1	0.142857142857143			0	4	
1007	2	2	2	2			
1013	1	1	1	4			
1016	1	1	1	4			
1022	1	1	1	4			
1024	1	0.333333333333333			0	4	
1029	1	0.333333333333333			0	4	
1044	1	0.333333333333333			0	4	
1059	1	0.333333333333333			0	4	
1064	1	0.333333333333333			0	4	
1102	1	0.333333333333333			0	4	
1140	1	0.333333333333333			0	4	
1174	1	1	1	4			
1196	1	0.333333333333333			0	4	
1201	1	1	1	4			
1228	1	1	1	4			
1300	1	1	1	4			
1379	1	0.333333333333333			0	4	
1385	1	0.333333333333333			0	4	
1397	1	0.333333333333333			0	4	
1405	2	0.666666666666667			0	3	
1408	1	0.333333333333333			0	4	
1417	12	4	0	0			
1418	2	0.666666666666667			0	3	<<<
1428	1	0.333333333333333			0	4	
1439	1	1	1	4			
1442	1	1	1	4			
1444	1	1	1	4			
1446	1	1	1	4			
1447	3	3	3	2			
1454	1	1	1	4			
1477	1	0.333333333333333			0	4	
1515	1	1	1	4			
1529	1	1	1	4			
1530	1	1	1	4			
1543	1	1	1	4			
1554	1	1	1	4			
1573	1	1	1	4			

1634	1	0.333333333333333	0	4
1636	1	0.333333333333333	0	4
1640	1	0.333333333333333	0	4
1641	1	0.333333333333333	0	4
1642	1	0.333333333333333	0	4
1657	1	0.333333333333333	0	4
1667	1	0.333333333333333	0	4
1673	1	0.333333333333333	0	4
1674	1	0.333333333333333	0	4
1678	1	1	1	4
1696	1	1	1	4
1700	1	1	1	4
1701	1	1	1	4
1702	2	2	2	2
1703	2	2	2	2
1704	1	1	1	4
1712	1	1	1	4
1715	2	2	2	2
1722	1	1	1	4
1728	1	1	1	4
1772	1	1	1	4
1811	2	2	2	2
1812	1	1	1	4
1815	1	1	1	4
1817	1	1	1	4
1818	2	2	2	2
1824	1	1	1	4
1837	1	1	1	4
1914	1	1	1	4

---

category=2, cleavage_site=1478
query=pto-miR154, target=Potri.003G169400.1,
score=4, range=1467-1487, strand=1

```
target  5' GUGCUCUCUCUcUUCUGUCAU 3'
           ::::::::. ::::::::::
query   3' CACGAGAGGUAGAAGACAGUU 5'
```

>Potri.003G169400.1
#size=2113

57	1	1	1	4
348	1	1	1	4
529	1	0.5	0	4
530	2	1	0	2
558	1	0.5	0	4
608	2	1	0	2
697	1	0.5	0	4
801	1	0.5	0	4
984	1	0.333333333333333	0	4
994	1	0.333333333333333	0	4
1026	1	0.333333333333333	0	4
1033	1	0.333333333333333	0	4
1042	1	0.333333333333333	0	4
1044	1	0.142857142857143	0	4
1084	1	0.333333333333333	0	4
1089	1	0.333333333333333	0	4
1104	1	0.333333333333333	0	4
1119	1	0.333333333333333	0	4
1124	1	0.333333333333333	0	4
1151	1	0.5	0	4

1162	1	0.333333333333333	0	4	
1177	1	0.5	0	4	
1180	1	0.5	0	4	
1200	1	0.333333333333333	0	4	
1216	2	1	0	2	
1256	1	0.333333333333333	0	4	
1382	1	0.5	0	4	
1394	1	0.5	0	4	
1439	1	0.333333333333333	0	4	
1445	1	0.333333333333333	0	4	
1457	1	0.333333333333333	0	4	
1465	2	0.666666666666667	0	2	
1468	1	0.333333333333333	0	4	
1477	12	4	0	0	
1478	2	0.666666666666667	0	2	<<<
1488	1	0.333333333333333	0	4	
1537	1	0.333333333333333	0	4	
1557	1	0.5	0	4	
1559	1	0.5	0	4	
1567	1	0.5	0	4	
1580	1	0.5	0	4	
1582	1	0.5	0	4	
1585	1	0.5	0	4	
1590	1	0.5	0	4	
1598	1	0.5	0	4	
1630	1	0.5	0	4	
1632	1	0.5	0	4	
1694	1	0.333333333333333	0	4	
1696	1	0.333333333333333	0	4	
1700	1	0.333333333333333	0	4	
1701	1	0.333333333333333	0	4	
1702	1	0.333333333333333	0	4	
1717	1	0.333333333333333	0	4	
1727	1	0.333333333333333	0	4	
1733	1	0.333333333333333	0	4	
1734	1	0.333333333333333	0	4	
1739	2	1	0	2	
1751	1	0.5	0	4	
1754	1	0.5	0	4	

---

category=2, cleavage_site=2199
query=pto-miR154, target=Potri.005G226900.1,
score=4, range=2188-2208, strand=1

```
target  5' UCGCUCUCCAUuUUCUGUCAU 3'
              :::::::::.::::::::
query   3' CACGAGAGGUAGAAGACAGUU 5'
```

>Potri.005G226900.1
#size=2368

24	1	0.125	0	4	
51	1	0.333333333333333	0	4	
122	1	0.333333333333333	0	4	
168	1	0.333333333333333	0	4	
178	1	0.333333333333333	0	4	
179	1	0.333333333333333	0	4	
183	3	0.375	0	3	
185	1	0.125	0	4	
189	2	0.25	0	3	
190	6	0.75	0	2	

191	19	2.375	0	2
192	11	1.375	0	2
197	1	0.125	0	4
201	2	0.25	0	3
327	1	0.125	0	4
328	2	0.25	0	3
333	1	0.125	0	4
339	1	0.333333333333333	0	4
359	3	0.791666666666667	0	2
360	1	0.333333333333333	0	4
363	2	0.458333333333333	0	3
365	3	0.791666666666667	0	2
366	1	0.333333333333333	0	4
370	5	1.66666666666667	0	2
371	2	0.666666666666667	0	2
376	1	0.333333333333333	0	4
378	3	1	0	2
380	7	2.33333333333333	0	2
381	2	0.666666666666667	0	2
382	15	5	0	2
383	3	1	0	2
384	2	0.666666666666667	0	2
385	1	0.333333333333333	0	4
401	1	0.125	0	4
405	2	0.25	0	3
409	1	0.125	0	4
421	1	0.125	0	4
422	1	0.125	0	4
423	2	0.25	0	3
426	1	0.125	0	4
429	1	0.125	0	4
464	2	0.666666666666667	0	2
930	2	0.25	0	3
966	1	0.125	0	4
1036	2	0.2	0	3
1079	1	0.1	0	4
1081	1	0.1	0	4
1100	1	0.1	0	4
1105	1	0.125	0	4
1109	1	0.1	0	4
1113	3	0.35	0	3
1116	1	0.125	0	4
1119	1	0.1	0	4
1126	2	0.25	0	3
1133	11	1.375	0	2
1135	1	0.125	0	4
1144	1	0.1	0	4
1175	2	0.25	0	3
1885	1	0.166666666666667	0	4
1887	1	0.166666666666667	0	4
1919	1	0.333333333333333	0	4
1958	1	0.333333333333333	0	4
2039	1	0.166666666666667	0	4
2042	3	0.5	0	3
2043	2	0.333333333333333	0	3
2046	1	0.166666666666667	0	4
2052	1	0.166666666666667	0	4
2066	2	0.333333333333333	0	3

2079	2	0.666666666666667	0	2
2082	3	1	0	2
2083	1	0.333333333333333	0	4
2084	1	0.333333333333333	0	4
2088	1	0.333333333333333	0	4
2089	3	1	0	2
2091	2	0.666666666666667	0	2
2092	9	3	0	2
2093	7	2.33333333333333	0	2
2095	12	4	0	2
2096	10	3.33333333333333	0	2
2098	3	1	0	2
2101	2	0.666666666666667	0	2
2102	2	0.666666666666667	0	2
2103	5	1.66666666666667	0	2
2104	1	0.333333333333333	0	4
2105	4	1.33333333333333	0	2
2106	1	0.333333333333333	0	4
2107	4	1.33333333333333	0	2
2109	2	0.666666666666667	0	2
2110	4	1.33333333333333	0	2
2111	4	1.33333333333333	0	2
2112	4	1.33333333333333	0	2
2113	3	0.5	0	3
2114	7	1.16666666666667	0	2
2115	6	1	0	2
2117	21	3.5	0	2
2118	4	0.666666666666667	0	2
2119	12	4	0	2
2120	3	1	0	2
2121	2	0.333333333333333	0	3
2123	10	2.83333333333333	0	2
2124	11	2.33333333333333	0	2
2126	1	0.333333333333333	0	4
2127	5	1.66666666666667	0	2
2128	18	6	0	2
2129	23	7.66666666666666	0	2
2130	2	0.666666666666667	0	2
2131	9	3	0	2
2132	10	3.33333333333333	0	2
2133	12	4	0	2
2134	15	5	0	2
2135	3	1	0	2
2136	8	2.66666666666667	0	2
2137	9	3	0	2
2138	7	2.33333333333333	0	2
2139	11	3.66666666666667	0	2
2140	4	1.33333333333333	0	2
2141	1	0.333333333333333	0	4
2142	3	1	0	2
2143	5	1.66666666666667	0	2
2144	10	3.33333333333333	0	2
2145	18	6	0	2
2146	19	6.33333333333333	0	2
2147	5	1.66666666666667	0	2
2148	22	7.33333333333333	0	2
2149	7	2.33333333333333	0	2
2150	10	3.33333333333333	0	2

2151	4	0.666666666666667	0	2	
2152	1	0.333333333333333	0	4	
2153	6	2	0	2	
2155	1	0.333333333333333	0	4	
2157	2	0.333333333333333	0	3	
2158	4	1.33333333333333	0	2	
2161	3	1	0	2	
2163	6	2	0	2	
2164	7	2.33333333333333	0	2	
2165	1	0.333333333333333	0	4	
2166	4	1.33333333333333	0	2	
2167	2	0.666666666666667	0	2	
2169	3	1	0	2	
2172	3	1	0	2	
2173	7	2.33333333333333	0	2	
2174	8	2.66666666666667	0	2	
2176	2	0.666666666666667	0	2	
2177	1	0.333333333333333	0	4	
2178	2	0.666666666666667	0	2	
2180	5	1.66666666666667	0	2	
2181	1	0.333333333333333	0	4	
2182	2	0.666666666666667	0	2	
2183	2	0.666666666666667	0	2	
2184	1	0.333333333333333	0	4	
2188	1	0.333333333333333	0	4	
2190	1	0.333333333333333	0	4	
2191	1	0.333333333333333	0	4	
2192	2	0.666666666666667	0	2	
2193	3	1	0	2	
2194	3	1	0	2	
2195	2	0.666666666666667	0	2	
2197	5	1.66666666666667	0	2	
2198	8	2.66666666666667	0	2	
2199	6	2	0	2	<<<
2200	6	2	0	2	
2201	8	2.66666666666667	0	2	
2203	16	5.33333333333333	0	2	
2204	12	4	0	2	
2205	16	5.33333333333333	0	2	
2206	5	1.66666666666667	0	2	
2207	30	10	0	0	
2208	5	1.66666666666667	0	2	
2209	5	1.66666666666667	0	2	
2210	5	1.66666666666667	0	2	
2211	5	1.66666666666667	0	2	
2212	11	3.66666666666667	0	2	
2213	6	2	0	2	
2214	2	0.666666666666667	0	2	
2215	12	4	0	2	
2216	1	0.333333333333333	0	4	
2217	24	8	0	2	
2218	3	1	0	2	
2224	1	0.333333333333333	0	4	
2226	1	0.333333333333333	0	4	
2227	2	0.666666666666667	0	2	
2231	9	3	0	2	
2232	3	1	0	2	
2233	1	0.333333333333333	0	4	

2235	5	1	0	2
2236	1	0.166666666666667	0	4
2237	11	2.83333333333333	0	2
2239	1	0.166666666666667	0	4
2240	1	0.333333333333333	0	4
2241	2	0.666666666666667	0	2
2243	1	0.333333333333333	0	4
2245	1	0.333333333333333	0	4
2246	1	0.333333333333333	0	4
2248	4	1.33333333333333	0	2
2268	1	0.333333333333333	0	4
2284	1	0.333333333333333	0	4
2294	1	0.333333333333333	0	4
2300	1	0.333333333333333	0	4
2304	1	0.333333333333333	0	4
2305	3	1	0	2
2315	2	0.666666666666667	0	2
2316	4	1.33333333333333	0	2
2337	3	1	0	2

---

category=0, cleavage_site=1186
query=pto-miR154, target=Potri.010G154300.1,
score=3.5, range=1175-1195, strand=1

```
target  5' GUGCUCUCUCUcUUCUGUCAG 3'
             ::::::::. ::::::::::.
query   3' CACGAGAGGUAGAAGACAGUU 5'
```

>Potri.010G154300.1
#size=2131

381	1	0.2	0	4	
396	1	0.2	0	4	
407	1	0.2	0	4	
409	1	0.2	0	4	
673	1	0.2	0	4	
893	1	0.5	0	4	
951	1	0.5	0	4	
1185	1	0.5	0	4	
1186	2	1	0	0	<<<
1294	1	0.5	0	4	
1367	1	0.5	0	4	
1384	1	0.5	0	4	
1533	1	0.2	0	4	
1691	1	0.5	0	4	
1771	1	0.5	0	4	

---

category=4, cleavage_site=740
query=pto-miR154, target=Potri.011G055900.1,
score=4, range=729-749, strand=1

```
target  5' AUGCUCUCUCUcUUCUGUCAA 3'
              :::::::. :::::::::::
query   3' CACGAGAGGUAGAAGACAGUU 5'
```

>Potri.011G055900.1
#size=864

70	1	1	1	4
71	1	1	1	4
82	2	2	2	2
106	1	1	1	4
121	1	1	1	4

148	1	1	1	4	
155	1	1	1	4	
167	1	0.5	0	4	
187	1	0.5	0	4	
192	2	2	2	2	
200	1	1	1	4	
201	1	1	1	4	
207	2	2	2	2	
222	1	1	1	4	
229	3	3	3	0	
231	1	1	1	4	
244	1	0.5	0	4	
256	1	1	1	4	
271	1	1	1	4	
279	1	1	1	4	
293	1	1	1	4	
331	1	1	1	4	
354	1	0.5	0	4	
361	1	0.5	0	4	
372	1	0.5	0	4	
373	1	0.5	0	4	
379	1	0.5	0	4	
402	1	1	1	4	
403	1	1	1	4	
406	1	1	1	4	
431	1	1	1	4	
439	1	1	1	4	
456	1	0.5	0	4	
469	1	0.5	0	4	
486	1	0.5	0	4	
491	1	0.5	0	4	
493	1	0.5	0	4	
499	1	1	1	4	
513	1	0.5	0	4	
515	2	1	0	2	
519	1	0.5	0	4	
521	1	0.5	0	4	
532	1	1	1	4	
552	1	1	1	4	
553	2	2	2	2	
555	1	1	1	4	
556	1	1	1	4	
562	1	1	1	4	
593	1	1	1	4	
633	1	1	1	4	
637	2	2	2	2	
653	1	1	1	4	
657	2	2	2	2	
666	1	1	1	4	
669	1	1	1	4	
673	1	1	1	4	
677	1	1	1	4	
691	1	1	1	4	
739	1	1	1	4	
740	1	1	1	4	<<<

category=2, cleavage_site=1856
query=pto-miR154, target=Potri.018G149900.1,
score=3, range=1845-1865, strand=1

```
target  5' GUGCUCUCUCUcUUCUGUCAA 3'
           ::::::::. :::::::::::
query   3' CACGAGAGGUAGAAGACAGUU 5'
```

>Potri.018G149900.1
#size=2752

80	1	0.333333333333333	0	4	
128	1	0.333333333333333	0	4	
201	1	0.5	0	4	
529	1	0.5	0	4	
535	1	0.5	0	4	
582	1	1	1	4	
653	1	0.25	0	4	
737	1	0.25	0	4	
756	1	0.25	0	4	
778	4	1	0	1	
995	1	0.25	0	4	
1145	1	0.25	0	4	
1196	1	0.25	0	4	
1259	1	0.25	0	4	
1356	1	0.25	0	4	
1388	1	0.2	0	4	
1394	1	0.2	0	4	
1422	1	0.25	0	4	
1425	2	0.5	0	2	
1489	1	0.25	0	4	
1583	1	0.25	0	4	
1646	1	0.25	0	4	
1759	1	0.25	0	4	
1762	1	0.25	0	4	
1812	1	0.25	0	4	
1856	3	0.75	0	2	<<<
2319	1	0.25	0	4	
2321	2	0.5	0	2	
2352	1	0.25	0	4	
2380	1	0.25	0	4	
2395	1	0.25	0	4	

pto-miR163

category=4, cleavage_site=3112
query=pto-miR163, target=Potri.016G054900.1,
score=4, range=3099-3121, strand=1

```
target  5' GUGGUCAGUUCUCcUUGCUUCUA 3'
           .::: :::.:::::::::.:::
query   3' UACC-GUCGAGAGGAACGGAGAC 5'
```

>Potri.016G054900.1
#size=3559

51	1	0.166666666666667	0	4
92	1	0.166666666666667	0	4
103	1	0.166666666666667	0	4
104	1	0.166666666666667	0	4
123	1	0.166666666666667	0	4
141	1	0.166666666666667	0	4
148	2	0.333333333333333	0	2

150	1	0.166666666666667	0	4
153	1	0.1	0	4
154	1	0.166666666666667	0	4
160	1	0.166666666666667	0	4
163	1	0.166666666666667	0	4
164	1	0.166666666666667	0	4
168	2	0.333333333333333	0	2
174	2	0.333333333333333	0	2
175	1	0.166666666666667	0	4
178	1	0.166666666666667	0	4
193	1	0.2	0	4
198	1	0.2	0	4
204	1	0.2	0	4
216	1	0.333333333333333	0	4
227	1	0.333333333333333	0	4
229	1	0.333333333333333	0	4
244	1	0.333333333333333	0	4
260	1	0.333333333333333	0	4
283	1	0.333333333333333	0	4
314	1	0.25	0	4
326	1	0.25	0	4
351	1	0.166666666666667	0	4
354	1	0.166666666666667	0	4
425	1	0.333333333333333	0	4
434	1	0.2	0	4
477	1	0.25	0	4
480	1	0.166666666666667	0	4
501	1	0.25	0	4
521	1	0.25	0	4
562	1	0.166666666666667	0	4
567	1	0.166666666666667	0	4
585	1	0.166666666666667	0	4
590	1	0.166666666666667	0	4
612	1	0.166666666666667	0	4
620	1	0.166666666666667	0	4
625	1	0.166666666666667	0	4
636	1	0.166666666666667	0	4
699	1	0.166666666666667	0	4
839	1	0.25	0	4
883	1	0.166666666666667	0	4
936	1	0.166666666666667	0	4
957	1	0.166666666666667	0	4
976	1	0.166666666666667	0	4
984	1	0.166666666666667	0	4
1062	1	0.166666666666667	0	4
1074	1	0.25	0	4
1092	1	0.166666666666667	0	4
1098	1	0.25	0	4
1115	1	0.166666666666667	0	4
1116	1	0.166666666666667	0	4
1133	1	0.166666666666667	0	4
1146	1	0.166666666666667	0	4
1148	1	0.25	0	4
1185	1	0.166666666666667	0	4
1187	1	0.166666666666667	0	4
1188	2	0.285714285714286	0	2
1257	1	0.25	0	4
1261	1	0.25	0	4

1269	1	0.166666666666667	0	4
1272	1	0.166666666666667	0	4
1278	1	0.166666666666667	0	4
1327	1	0.1	0	4
1328	2	0.2	0	2
1331	2	0.2	0	2
1332	2	0.2	0	2
1334	1	0.166666666666667	0	4
1337	1	0.1	0	4
1338	1	0.166666666666667	0	4
1349	1	0.25	0	4
1436	1	0.166666666666667	0	4
1447	1	0.166666666666667	0	4
1489	1	0.25	0	4
1524	2	0.416666666666667	0	2
1530	1	0.166666666666667	0	4
1531	1	0.166666666666667	0	4
1546	1	0.166666666666667	0	4
1556	1	0.166666666666667	0	4
1565	1	0.166666666666667	0	4
1579	1	0.166666666666667	0	4
1598	1	0.166666666666667	0	4
1610	1	0.166666666666667	0	4
1637	2	0.4	0	2
1639	2	0.4	0	2
1643	1	0.166666666666667	0	4
1714	1	0.166666666666667	0	4
1761	1	0.166666666666667	0	4
1764	1	0.166666666666667	0	4
1769	1	0.166666666666667	0	4
1782	1	0.166666666666667	0	4
1783	1	0.166666666666667	0	4
1785	1	0.166666666666667	0	4
1790	1	0.166666666666667	0	4
1796	1	0.166666666666667	0	4
1797	2	0.333333333333333	0	2
1800	1	0.166666666666667	0	4
1802	1	0.166666666666667	0	4
1803	1	0.166666666666667	0	4
1804	2	0.333333333333333	0	2
1805	1	0.166666666666667	0	4
1806	2	0.333333333333333	0	2
1809	2	0.333333333333333	0	2
1813	1	0.166666666666667	0	4
1815	1	0.166666666666667	0	4
1819	1	0.166666666666667	0	4
1827	2	0.333333333333333	0	2
1835	1	0.166666666666667	0	4
1848	1	0.166666666666667	0	4
1852	1	0.166666666666667	0	4
1861	1	0.166666666666667	0	4
1865	1	0.166666666666667	0	4
1867	1	0.166666666666667	0	4
1884	1	0.166666666666667	0	4
1887	1	0.166666666666667	0	4
1897	1	0.166666666666667	0	4
1902	1	0.166666666666667	0	4
1919	1	0.166666666666667	0	4

1967	1	0.25	0	4
2035	1	0.166666666666667	0	4
2053	1	0.166666666666667	0	4
2102	1	0.166666666666667	0	4
2118	1	0.166666666666667	0	4
2135	1	0.166666666666667	0	4
2140	2	0.333333333333333	0	2
2142	1	0.166666666666667	0	4
2143	1	0.125	0	4
2153	1	0.166666666666667	0	4
2156	2	0.291666666666667	0	2
2191	1	0.166666666666667	0	4
2198	1	0.166666666666667	0	4
2200	1	0.166666666666667	0	4
2214	1	0.166666666666667	0	4
2216	1	0.166666666666667	0	4
2225	1	0.166666666666667	0	4
2234	1	0.1	0	4
2238	1	0.166666666666667	0	4
2245	1	0.166666666666667	0	4
2248	1	0.166666666666667	0	4
2255	1	0.166666666666667	0	4
2292	1	0.166666666666667	0	4
2316	1	0.125	0	4
2319	1	0.1	0	4
2322	1	0.1	0	4
2323	1	0.1	0	4
2327	1	0.166666666666667	0	4
2332	1	0.166666666666667	0	4
2381	1	0.166666666666667	0	4
2386	1	0.166666666666667	0	4
2394	1	0.166666666666667	0	4
2399	1	0.166666666666667	0	4
2400	1	0.166666666666667	0	4
2401	1	0.166666666666667	0	4
2402	1	0.166666666666667	0	4
2403	1	0.166666666666667	0	4
2414	1	0.25	0	4
2415	1	0.25	0	4
2419	1	0.25	0	4
2423	1	0.25	0	4
2424	1	0.25	0	4
2428	1	0.25	0	4
2434	1	0.166666666666667	0	4
2437	2	0.333333333333333	0	2
2442	1	0.166666666666667	0	4
2444	1	0.166666666666667	0	4
2448	1	0.166666666666667	0	4
2451	1	0.166666666666667	0	4
2453	1	0.166666666666667	0	4
2468	1	0.166666666666667	0	4
2472	1	0.166666666666667	0	4
2505	1	0.166666666666667	0	4
2518	1	0.25	0	4
2526	1	0.25	0	4
2545	1	0.166666666666667	0	4
2553	1	0.166666666666667	0	4
2560	1	0.166666666666667	0	4

2577	1	0.25	0	4
2590	1	0.166666666666667	0	4
2593	1	0.166666666666667	0	4
2594	1	0.166666666666667	0	4
2606	1	0.25	0	4
2636	1	0.166666666666667	0	4
2653	1	0.25	0	4
2654	1	0.166666666666667	0	4
2657	1	0.166666666666667	0	4
2659	1	0.166666666666667	0	4
2671	2	0.333333333333333	0	2
2673	1	0.166666666666667	0	4
2678	2	0.333333333333333	0	2
2679	2	0.333333333333333	0	2
2683	1	0.166666666666667	0	4
2697	1	0.1	0	4
2705	1	0.1	0	4
2713	1	0.25	0	4
2734	2	0.333333333333333	0	2
2739	1	0.166666666666667	0	4
2741	1	0.166666666666667	0	4
2753	1	0.166666666666667	0	4
2759	1	0.166666666666667	0	4
2765	1	0.166666666666667	0	4
2795	1	0.166666666666667	0	4
2816	1	0.166666666666667	0	4
2820	1	0.166666666666667	0	4
2828	2	0.266666666666667	0	2
2833	1	0.166666666666667	0	4
2835	1	0.166666666666667	0	4
2862	1	0.166666666666667	0	4
2952	1	0.166666666666667	0	4
2979	1	0.166666666666667	0	4
2993	1	0.1	0	4
2995	1	0.1	0	4
3001	1	0.166666666666667	0	4
3013	2	0.333333333333333	0	2
3024	1	0.166666666666667	0	4
3039	1	0.166666666666667	0	4
3040	1	0.166666666666667	0	4
3048	2	0.333333333333333	0	2
3050	1	0.166666666666667	0	4
3053	1	0.166666666666667	0	4
3056	1	0.25	0	4
3061	1	0.25	0	4
3065	2	0.5	0	2
3067	1	0.25	0	4
3073	1	0.25	0	4
3078	2	0.5	0	2
3079	1	0.25	0	4
3080	2	0.5	0	2
3084	2	0.5	0	2
3086	1	0.25	0	4
3090	1	0.25	0	4
3091	1	0.25	0	4
3099	2	0.5	0	2
3100	2	0.5	0	2
3102	2	0.5	0	2

3110	1	0.25	0	4	
3112	1	0.25	0	4	<<<
3120	1	0.25	0	4	
3121	1	0.25	0	4	
3123	2	0.5	0	2	
3133	1	0.25	0	4	
3135	1	0.25	0	4	
3136	1	0.25	0	4	
3140	2	0.5	0	2	
3141	1	0.25	0	4	
3157	1	0.25	0	4	
3177	1	0.333333333333333	0	4	
3178	1	0.333333333333333	0	4	
3200	1	0.333333333333333	0	4	
3213	1	0.333333333333333	0	4	
3216	1	0.333333333333333	0	4	
3260	1	0.333333333333333	0	4	
3261	1	0.333333333333333	0	4	
3266	1	0.333333333333333	0	4	
3270	1	0.333333333333333	0	4	
3336	1	0.333333333333333	0	4	
3355	2	0.666666666666667	0	0	
3380	1	0.333333333333333	0	4	
3382	1	0.333333333333333	0	4	
3443	1	0.333333333333333	0	4	

pto-miR168

category=4, cleavage_site=828
query= pto-miR168, target=Potri.005G044400.2,
score=4, range=816-836, strand=1

```
target  5' CACAGCACCGCCa-UGGUCUCC 3'
            : :::::::::: .:::::::
query   3' GAGUCGUGGCGGUGGCCAGAGG 5'
```

>Potri.005G044400.2
#size=1369

21	2	0.333333333333333	0	2
26	1	0.166666666666667	0	4
34	2	0.333333333333333	0	2
36	1	0.166666666666667	0	4
61	3	0.566666666666667	0	2
62	1	0.2	0	4
71	3	0.375	0	2
73	3	0.375	0	2
74	3	0.375	0	2
75	2	0.325	0	2
76	1	0.125	0	4
77	2	0.325	0	2
78	2	0.325	0	2
83	4	0.5	0	2
85	1	0.125	0	4
87	2	0.236111111111111	0	3
88	3	0.322222222222222	0	2
89	1	0.1	0	4
91	8	0.8	0	2
92	1	0.1	0	4
93	15	1.5	0	2

94	14	1.53333333333333	0	2
95	3	0.366666666666667	0	2
96	1	0.1	0	4
97	2	0.266666666666667	0	3
98	3	0.3	0	2
99	3	0.342857142857143	0	2
100	6	0.6	0	2
101	1	0.1	0	4
102	1	0.1	0	4
103	1	0.1	0	4
104	7	0.7	0	2
105	2	0.2	0	3
107	3	0.3	0	2
108	1	0.1	0	4
109	2	0.242857142857143	0	3
111	3	0.5	0	2
116	1	0.1	0	4
120	2	0.333333333333333	0	2
121	1	0.166666666666667	0	4
129	1	0.166666666666667	0	4
130	2	0.333333333333333	0	2
132	2	0.333333333333333	0	2
136	1	0.166666666666667	0	4
138	2	0.333333333333333	0	2
140	3	0.5	0	2
142	1	0.166666666666667	0	4
144	1	0.166666666666667	0	4
145	2	0.333333333333333	0	2
147	1	0.166666666666667	0	4
157	1	0.166666666666667	0	4
158	1	0.166666666666667	0	4
160	1	0.1	0	4
161	1	0.1	0	4
162	1	0.1	0	4
163	4	0.4	0	2
165	2	0.2	0	3
167	1	0.1	0	4
168	1	0.1	0	4
169	2	0.2	0	3
170	1	0.1	0	4
173	4	0.666666666666667	0	2
177	1	0.142857142857143	0	4
181	1	0.142857142857143	0	4
183	1	0.142857142857143	0	4
185	1	0.142857142857143	0	4
187	1	0.142857142857143	0	4
189	1	0.142857142857143	0	4
192	1	0.142857142857143	0	4
196	1	0.142857142857143	0	4
202	1	0.142857142857143	0	4
203	1	0.1	0	4
204	2	0.285714285714286	0	3
206	1	0.1	0	4
209	1	0.1	0	4
211	3	0.3	0	2
212	1	0.1	0	4
213	2	0.2	0	3
214	1	0.1	0	4

215	1	0.1	0	4
217	2	0.242857142857143	0	3
219	3	0.3	0	2
220	1	0.1	0	4
222	1	0.1	0	4
227	2	0.242857142857143	0	3
228	2	0.2	0	3
231	2	0.2	0	3
232	2	0.2	0	3
233	1	0.1	0	4
236	2	0.2	0	3
237	1	0.1	0	4
239	2	0.2	0	3
241	1	0.1	0	4
243	4	0.4	0	2
244	2	0.2	0	3
250	1	0.1	0	4
252	2	0.2	0	3
254	1	0.1	0	4
258	1	0.1	0	4
259	1	0.1	0	4
260	1	0.1	0	4
261	1	0.1	0	4
262	3	0.3	0	2
263	2	0.2	0	3
264	3	0.3	0	2
265	2	0.2	0	3
267	2	0.2	0	3
268	3	0.3	0	2
269	3	0.3	0	2
270	1	0.1	0	4
271	4	0.4	0	2
272	2	0.2	0	3
274	4	0.4	0	2
275	4	0.4	0	2
276	2	0.2	0	3
277	2	0.2	0	3
278	4	0.442857142857143	0	2
279	7	0.7	0	2
280	3	0.3	0	2
282	3	0.3	0	2
284	5	0.5	0	2
285	5	0.5	0	2
286	3	0.3	0	2
287	4	0.4	0	2
288	5	0.5	0	2
289	3	0.3	0	2
290	3	0.3	0	2
291	7	0.7	0	2
292	11	1.1	0	2
293	2	0.2	0	3
294	4	0.4	0	2
295	2	0.2	0	3
296	2	0.2	0	3
297	3	0.3	0	2
298	4	0.4	0	2
299	4	0.4	0	2
300	3	0.3	0	2

301	2	0.2	0	3
302	7	0.7	0	2
303	4	0.4	0	2
304	7	0.7	0	2
305	9	0.9	0	2
306	3	0.3	0	2
307	4	0.4	0	2
308	4	0.4	0	2
309	4	0.4	0	2
310	5	0.5	0	2
311	5	0.5	0	2
312	6	0.6	0	2
313	5	0.5	0	2
314	6	0.6	0	2
315	1	0.1	0	4
316	3	0.3	0	2
317	3	0.3	0	2
318	4	0.4	0	2
319	2	0.2	0	3
320	5	0.5	0	2
321	4	0.4	0	2
322	3	0.3	0	2
323	2	0.2	0	3
324	7	0.7	0	2
325	6	0.6	0	2
326	1	0.1	0	4
327	3	0.3	0	2
328	2	0.2	0	3
329	3	0.3	0	2
330	3	0.3	0	2
331	1	0.1	0	4
332	3	0.3	0	2
333	2	0.2	0	3
334	4	0.4	0	2
336	2	0.2	0	3
338	2	0.2	0	3
339	4	0.4	0	2
341	1	0.1	0	4
342	1	0.1	0	4
343	2	0.2	0	3
344	1	0.1	0	4
345	5	0.5	0	2
346	4	0.4	0	2
347	1	0.1	0	4
348	5	0.5	0	2
349	4	0.4	0	2
350	5	0.5	0	2
351	6	0.642857142857143	0	2
353	9	0.9	0	2
354	10	1	0	2
355	6	0.6	0	2
356	2	0.2	0	3
357	3	0.3	0	2
358	2	0.285714285714286	0	3
359	4	0.442857142857143	0	2
360	3	0.385714285714286	0	2
361	2	0.285714285714286	0	3
362	2	0.285714285714286	0	3

363	3	0.342857142857143	0	2
365	1	0.1	0	4
367	2	0.242857142857143	0	3
368	2	0.242857142857143	0	3
369	1	0.1	0	4
370	3	0.3	0	2
372	1	0.142857142857143	0	4
373	2	0.285714285714286	0	3
374	1	0.142857142857143	0	4
375	4	0.571428571428571	0	2
376	2	0.285714285714286	0	3
377	2	0.285714285714286	0	3
378	1	0.142857142857143	0	4
379	1	0.142857142857143	0	4
380	2	0.285714285714286	0	3
381	1	0.142857142857143	0	4
382	5	0.714285714285714	0	2
383	1	0.142857142857143	0	4
384	2	0.285714285714286	0	3
385	3	0.428571428571429	0	2
387	1	0.142857142857143	0	4
391	3	0.3	0	2
392	2	0.2	0	3
394	1	0.1	0	4
395	1	0.1	0	4
396	2	0.2	0	3
397	2	0.2	0	3
398	3	0.3	0	2
399	2	0.2	0	3
400	3	0.3	0	2
402	5	0.5	0	2
403	1	0.142857142857143	0	4
404	1	0.142857142857143	0	4
405	3	0.385714285714286	0	2
406	2	0.285714285714286	0	3
407	5	0.714285714285714	0	2
408	5	0.5	0	2
409	2	0.2	0	3
410	1	0.1	0	4
411	5	0.5	0	2
412	2	0.2	0	3
413	1	0.1	0	4
414	4	0.485714285714286	0	2
415	3	0.3	0	2
416	2	0.2	0	3
417	6	0.6	0	2
418	3	0.3	0	2
419	9	0.9	0	2
420	4	0.4	0	2
421	6	0.6	0	2
422	4	0.4	0	2
423	5	0.5	0	2
424	6	0.6	0	2
425	10	1	0	2
426	6	0.6	0	2
427	11	1.1	0	2
428	5	0.714285714285714	0	2
429	9	1.28571428571429	0	2

430	14	1.4	0	2
431	11	1.14285714285714	0	2
432	11	1.14285714285714	0	2
433	16	1.6	0	2
434	25	2.54285714285714	0	0
435	23	2.3	0	2
436	10	1.08571428571429	0	2
437	12	1.2	0	2
438	9	0.9	0	2
439	16	1.6	0	2
440	16	1.6	0	2
441	7	0.7	0	2
442	14	1.4	0	2
443	5	0.5	0	2
444	4	0.4	0	2
445	1	0.1	0	4
446	3	0.3	0	2
448	4	0.4	0	2
449	5	0.5	0	2
450	2	0.2	0	3
451	4	0.4	0	2
452	3	0.3	0	2
453	1	0.1	0	4
454	4	0.4	0	2
455	3	0.3	0	2
456	4	0.4	0	2
457	5	0.5	0	2
458	4	0.4	0	2
459	7	0.7	0	2
460	3	0.3	0	2
461	4	0.4	0	2
462	9	0.9	0	2
463	4	0.4	0	2
464	9	0.9	0	2
465	6	0.6	0	2
466	6	0.6	0	2
467	10	1	0	2
468	11	1.1	0	2
469	15	1.5	0	2
470	9	0.9	0	2
471	7	0.7	0	2
472	4	0.4	0	2
473	2	0.2	0	3
474	4	0.4	0	2
475	1	0.1	0	4
476	12	1.2	0	2
477	4	0.4	0	2
478	8	0.8	0	2
479	5	0.5	0	2
480	6	0.666666666666667	0	2
481	6	0.666666666666667	0	2
482	5	0.5	0	2
483	7	0.7	0	2
484	6	0.6	0	2
485	7	0.766666666666667	0	2
486	1	0.1	0	4
487	7	0.7	0	2
488	5	0.5	0	2

489	7	0.7	0	2	
490	3	0.385714285714286	0	2	
491	2	0.285714285714286	0	3	
492	7	0.785714285714286	0	2	
493	1	0.142857142857143	0	4	
494	9	1.2	0	2	
495	4	0.528571428571428	0	2	
496	4	0.4	0	2	
497	4	0.485714285714286	0	2	
498	5	0.5	0	2	
499	5	0.628571428571429	0	2	
500	2	0.242857142857143	0	3	
501	5	0.5	0	2	
502	8	0.8	0	2	
503	1	0.1	0	4	
504	5	0.585714285714286	0	2	
505	5	0.5	0	2	
506	5	0.585714285714286	0	2	
507	4	0.442857142857143	0	2	
508	4	0.571428571428571	0	2	
509	1	0.142857142857143	0	4	
511	1	0.142857142857143	0	4	
775	3	0.428571428571429	0	2	
776	2	0.285714285714286	0	3	
778	5	0.714285714285714	0	2	
779	1	0.142857142857143	0	4	
780	3	0.428571428571429	0	2	
781	3	0.428571428571429	0	2	
782	2	0.285714285714286	0	3	
783	2	0.285714285714286	0	3	
784	2	0.285714285714286	0	3	
786	1	0.142857142857143	0	4	
787	1	0.142857142857143	0	4	
788	1	0.142857142857143	0	4	
790	2	0.285714285714286	0	3	
791	2	0.285714285714286	0	3	
792	1	0.142857142857143	0	4	
793	1	0.142857142857143	0	4	
795	2	0.285714285714286	0	3	
796	1	0.142857142857143	0	4	
797	1	0.142857142857143	0	4	
798	1	0.142857142857143	0	4	
799	1	0.142857142857143	0	4	
801	2	0.285714285714286	0	3	
803	1	0.142857142857143	0	4	
804	1	0.142857142857143	0	4	
806	1	0.142857142857143	0	4	
810	2	0.285714285714286	0	3	
811	1	0.142857142857143	0	4	
813	1	0.142857142857143	0	4	
814	1	0.166666666666667	0	4	
819	1	0.166666666666667	0	4	
821	1	0.166666666666667	0	4	
823	1	0.166666666666667	0	4	
825	1	0.166666666666667	0	4	
826	4	0.666666666666667	0	2	
827	1	0.166666666666667	0	4	
828	1	0.166666666666667	0	4	<<<

830	3	0.5	0	2
831	1	0.166666666666667	0	4
832	1	0.166666666666667	0	4
833	1	0.166666666666667	0	4
835	2	0.333333333333333	0	2
837	1	0.166666666666667	0	4
838	3	0.5	0	2
840	1	0.166666666666667	0	4
841	2	0.333333333333333	0	2
842	2	0.333333333333333	0	2
845	1	0.166666666666667	0	4
846	1	0.166666666666667	0	4
847	3	0.5	0	2
848	1	0.166666666666667	0	4
849	1	0.166666666666667	0	4
850	4	0.666666666666667	0	2
854	1	0.166666666666667	0	4
855	1	0.166666666666667	0	4
856	3	0.5	0	2
857	1	0.166666666666667	0	4
858	2	0.333333333333333	0	2
861	3	0.5	0	2
862	3	0.5	0	2
863	2	0.333333333333333	0	2
865	2	0.333333333333333	0	2
866	2	0.333333333333333	0	2
867	3	0.5	0	2
869	1	0.166666666666667	0	4
870	5	0.833333333333333	0	2
871	4	0.571428571428571	0	2
872	3	0.428571428571429	0	2
873	2	0.285714285714286	0	3
874	3	0.428571428571429	0	2
875	6	0.857142857142857	0	2
876	5	0.714285714285714	0	2
877	4	0.571428571428571	0	2
878	2	0.285714285714286	0	3
879	4	0.571428571428571	0	2
880	2	0.285714285714286	0	3
881	5	0.714285714285714	0	2
882	2	0.285714285714286	0	3
884	1	0.142857142857143	0	4
885	1	0.142857142857143	0	4
886	1	0.142857142857143	0	4
889	2	0.285714285714286	0	3
897	1	0.142857142857143	0	4
903	3	0.428571428571429	0	2
905	1	0.142857142857143	0	4
909	1	0.142857142857143	0	4
910	1	0.142857142857143	0	4
913	2	0.285714285714286	0	3
916	1	0.142857142857143	0	4
918	3	0.428571428571429	0	2
922	1	0.142857142857143	0	4
929	1	0.142857142857143	0	4
937	1	0.142857142857143	0	4
938	1	0.142857142857143	0	4
962	1	0.142857142857143	0	4

963	1	0.142857142857143	0	4
964	1	0.142857142857143	0	4
969	1	0.142857142857143	0	4
970	1	0.142857142857143	0	4
975	1	0.142857142857143	0	4
976	1	0.142857142857143	0	4
978	3	0.428571428571429	0	2
980	2	0.285714285714286	0	3
982	1	0.142857142857143	0	4
988	1	0.142857142857143	0	4
989	1	0.142857142857143	0	4
994	1	0.142857142857143	0	4
997	1	0.142857142857143	0	4
1002	1	0.142857142857143	0	4
1221	1	0.142857142857143	0	4

pto-miR177

category=4, cleavage_site=1754
query= pto-miR177, target=Potri.016G113600.1,
score=4, range=1744-1763, strand=1

```
target  5' CCAAC-UCCUCaACCAUUCCA 3'
           ::::: ::::::: :::.:::
query   3' GGUUGAAGGAGUUCGUAGGGU 5'
```

>Potri.016G113600.1
#size=2592

119	1	0.25	0	4
125	1	0.25	0	4
150	1	0.142857142857143	0	4
155	1	0.25	0	4
200	1	0.25	0	4
217	1	0.25	0	4
250	1	0.25	0	4
302	1	0.25	0	4
377	1	0.25	0	4
461	1	0.25	0	4
506	1	0.25	0	4
536	1	0.25	0	4
634	1	0.25	0	4
651	1	0.25	0	4
676	1	0.25	0	4
755	1	0.25	0	4
758	1	0.25	0	4
812	1	0.25	0	4
856	1	0.111111111111111	0	4
869	1	0.111111111111111	0	4
881	2	0.4	0	2
882	1	0.2	0	4
902	1	0.2	0	4
918	1	0.2	0	4
942	2	0.4	0	2
943	1	0.2	0	4
958	2	0.4	0	2
1015	1	0.25	0	4
1037	1	0.25	0	4
1062	1	0.2	0	4
1154	1	0.111111111111111	0	4

1163	1	0.111111111111111	0	4	
1186	1	0.1	0	4	
1187	1	0.111111111111111	0	4	
1190	1	0.2	0	4	
1197	1	0.111111111111111	0	4	
1207	1	0.1	0	4	
1225	1	0.111111111111111	0	4	
1226	1	0.111111111111111	0	4	
1229	2	0.222222222222222	0	2	
1265	1	0.111111111111111	0	4	
1277	1	0.2	0	4	
1289	1	0.2	0	4	
1311	1	0.111111111111111	0	4	
1313	1	0.111111111111111	0	4	
1317	1	0.2	0	4	
1321	3	0.333333333333333	0	2	
1341	1	0.1	0	4	
1350	1	0.1	0	4	
1451	1	0.111111111111111	0	4	
1460	1	0.166666666666667	0	4	
1486	1	0.166666666666667	0	4	
1527	1	0.166666666666667	0	4	
1551	1	0.166666666666667	0	4	
1560	1	0.1	0	4	
1570	1	0.1	0	4	
1590	2	0.2	0	2	
1594	1	0.1	0	4	
1596	1	0.1	0	4	
1597	1	0.1	0	4	
1618	1	0.166666666666667	0	4	
1622	1	0.166666666666667	0	4	
1668	1	0.142857142857143	0	4	
1671	1	0.142857142857143	0	4	
1679	1	0.142857142857143	0	4	
1682	1	0.142857142857143	0	4	
1691	1	0.142857142857143	0	4	
1694	1	0.142857142857143	0	4	
1730	1	0.142857142857143	0	4	
1732	1	0.142857142857143	0	4	
1738	1	0.142857142857143	0	4	
1754	1	0.142857142857143	0	4	<<<
1777	1	0.142857142857143	0	4	
1795	1	0.142857142857143	0	4	
1809	1	0.142857142857143	0	4	
1813	1	0.142857142857143	0	4	
1832	1	0.142857142857143	0	4	
1845	1	0.1	0	4	
1846	1	0.142857142857143	0	4	
1854	1	0.142857142857143	0	4	
1857	1	0.1	0	4	
1861	1	0.1	0	4	
1862	1	0.1	0	4	
1869	1	0.1	0	4	
1883	2	0.285714285714286	0	2	
1886	1	0.142857142857143	0	4	
1887	2	0.285714285714286	0	2	
1894	1	0.142857142857143	0	4	
1895	1	0.142857142857143	0	4	

1896	1	0.1	0	4
1906	2	0.2	0	2
1935	1	0.142857142857143	0	4
1987	1	0.142857142857143	0	4
1990	1	0.142857142857143	0	4
2007	1	0.142857142857143	0	4
2027	1	0.142857142857143	0	4
2034	1	0.142857142857143	0	4
2037	1	0.1	0	4
2056	1	0.1	0	4
2072	1	0.2	0	4
2091	1	0.2	0	4
2096	1	0.2	0	4
2118	1	0.2	0	4
2120	2	0.4	0	2
2121	3	0.6	0	2
2122	4	0.8	0	0
2123	3	0.6	0	2
2124	1	0.2	0	4
2125	3	0.6	0	2
2127	1	0.2	0	4
2128	1	0.2	0	4
2130	1	0.2	0	4
2131	3	0.6	0	2
2134	1	0.125	0	4
2139	1	0.125	0	4
2140	1	0.2	0	4
2141	3	0.525	0	2
2142	1	0.2	0	4
2144	1	0.125	0	4
2147	2	0.25	0	2
2151	1	0.125	0	4
2152	2	0.25	0	2
2153	2	0.25	0	2
2154	1	0.125	0	4
2155	1	0.125	0	4
2156	2	0.25	0	2
2160	1	0.125	0	4
2163	1	0.125	0	4
2168	1	0.125	0	4
2180	1	0.125	0	4
2181	2	0.25	0	2
2184	1	0.125	0	4
2186	1	0.2	0	4
2191	1	0.2	0	4
2224	1	0.2	0	4
2225	2	0.4	0	2
2226	1	0.2	0	4
2227	1	0.2	0	4
2229	1	0.2	0	4
2230	1	0.2	0	4
2231	1	0.2	0	4
2235	2	0.4	0	2
2236	1	0.2	0	4
2239	1	0.2	0	4
2240	3	0.6	0	2
2242	1	0.2	0	4
2245	1	0.2	0	4

2248	1	0.2	0	4
2250	1	0.2	0	4
2251	1	0.2	0	4
2252	1	0.2	0	4
2256	1	0.2	0	4
2257	1	0.2	0	4
2259	1	0.2	0	4
2265	1	0.2	0	4
2266	1	0.2	0	4
2274	1	0.2	0	4
2329	1	0.2	0	4
2331	1	0.2	0	4
2360	1	0.2	0	4
2502	1	0.2	0	4

pto-miR180

category=4, cleavage_site=5810
query=pto-miR180, target=Potri.002G049600.1,
score=4, range=5800-5819, strand=1

```
target  5' ACUUC-UGUUCaUGCGUGCAA 3'
           : :::  ::::::::.:::::
query   3' UAAAGUUCAAGUACGUACGUU 5'
```

>Potri.002G049600.1
#size=6309

294	1	0.5	0	4
512	2	0.4	0	3
1624	1	0.5	0	4
1794	1	0.2	0	4
1921	1	0.2	0	4
1922	1	0.2	0	4
1925	1	0.2	0	4
2472	1	0.2	0	4
2525	2	0.4	0	3
2664	1	0.2	0	4
2785	1	0.5	0	4
2817	2	0.4	0	3
2857	1	0.2	0	4
2876	1	0.2	0	4
2883	2	0.4	0	3
2894	3	0.6	0	2
2916	1	0.5	0	4
3072	3	0.6	0	2
3076	1	0.2	0	4
3077	4	0.8	0	2
3078	4	0.8	0	2
3100	1	0.5	0	4
3102	1	0.5	0	4
3356	1	0.5	0	4
3357	2	1	0	2
3359	1	0.5	0	4
3372	2	1	0	2
3394	1	0.2	0	4
3666	2	0.4	0	3
3692	2	0.4	0	3
3714	2	0.4	0	3
3715	2	0.4	0	3

3757	6	1.2	0	2		
3761	1	0.2	0	4		
3768	4	0.8	0	2		
3806	10	2	0	2		
3819	1	0.2	0	4		
4238	1	0.2	0	4		
4275	3	0.6	0	2		
4277	3	0.6	0	2		
4284	1	0.2	0	4		
4285	1	0.2	0	4		
4286	4	0.8	0	2		
4293	1	0.5	0	4		
4439	1	0.2	0	4		
4736	2	1	0	2		
4868	1	0.5	0	4		
4976	1	0.5	0	4		
5035	2	0.4	0	3		
5047	1	0.5	0	4		
5075	1	0.5	0	4		
5076	9	4.5	0	2		
5078	3	1.5	0	2		
5081	1	0.5	0	4		
5084	2	1	0	2		
5101	1	0.111111111111111			0	4
5134	3	1.5	0	2		
5138	2	0.4	0	3		
5139	1	0.2	0	4		
5140	2	0.4	0	3		
5148	4	0.8	0	2		
5149	1	0.2	0	4		
5232	1	0.25	0	4		
5236	3	0.75	0	2		
5239	1	0.125	0	4		
5240	1	0.125	0	4		
5272	4	1	0	2		
5323	1	0.25	0	4		
5360	1	0.25	0	4		
5361	1	0.142857142857143			0	4
5362	1	0.142857142857143			0	4
5370	1	0.25	0	4		
5377	2	0.5	0	3		
5379	1	0.25	0	4		
5380	1	0.25	0	4		
5383	1	0.25	0	4		
5385	1	0.25	0	4		
5391	1	0.25	0	4		
5395	1	0.25	0	4		
5513	1	0.2	0	4		
5514	1	0.2	0	4		
5560	1	0.2	0	4		
5561	2	0.4	0	3		
5569	3	0.9	0	2		
5570	2	0.4	0	3		
5596	1	0.5	0	4		
5622	1	0.5	0	4		
5623	1	0.2	0	4		
5636	1	0.2	0	4		
5639	2	0.4	0	3		

5642	3	0.6	0	2	
5649	1	0.2	0	4	
5653	5	1	0	2	
5656	1	0.2	0	4	
5658	3	0.6	0	2	
5667	1	0.5	0	4	
5682	1	0.5	0	4	
5685	1	0.5	0	4	
5686	3	1.5	0	2	
5691	1	0.2	0	4	
5728	1	0.5	0	4	
5729	2	1	0	2	
5734	1	0.5	0	4	
5735	1	0.5	0	4	
5738	2	1	0	2	
5739	2	1	0	2	
5740	8	3.4	0	2	
5741	5	2.2	0	2	
5743	3	1.5	0	2	
5747	3	1.5	0	2	
5749	7	3.5	0	2	
5752	2	1	0	2	
5753	1	0.5	0	4	
5757	5	2.5	0	2	
5766	5	2.5	0	2	
5768	1	0.5	0	4	
5788	2	1	0	2	
5791	5	2.5	0	2	
5794	1	0.5	0	4	
5797	3	0.6	0	2	
5801	7	1.4	0	2	
5804	2	0.4	0	3	
5805	2	0.4	0	3	
5809	1	0.2	0	4	
5810	1	0.2	0	4	<<<
5812	1	0.2	0	4	
5815	1	0.2	0	4	
5820	1	0.2	0	4	
5822	1	0.2	0	4	
5824	1	0.5	0	4	
5826	1	0.5	0	4	
5833	1	0.5	0	4	
5845	2	1	0	2	
5852	2	1	0	2	
5856	2	1	0	2	
5859	9	4.5	0	2	
5862	3	1.5	0	2	
5865	1	0.5	0	4	
5867	1	0.5	0	4	
5868	2	1	0	2	
5869	3	1.5	0	2	
5870	1	0.5	0	4	
5872	1	0.5	0	4	
5873	2	1	0	2	
5882	1	0.5	0	4	
5883	3	1.5	0	2	
5885	1	0.5	0	4	
5889	1	0.5	0	4	

5893	10	5	0	2
5896	1	0.5	0	4
5897	12	6	0	2
5898	3	1.5	0	2
5899	14	7	0	2
5900	10	2	0	2
5902	11	2.2	0	2
5903	5	1.6	0	2
5904	7	1.4	0	2
5905	16	4.1	0	2
5906	19	3.8	0	2
5907	4	0.8	0	2
5908	5	1	0	2
5909	34	6.8	0	2
5910	16	3.2	0	2
5912	17	6.1	0	2
5913	4	1.4	0	2
5914	7	2.9	0	2
5915	2	0.7	0	2
5916	2	0.4	0	3
5917	3	0.6	0	2
5918	3	0.6	0	2
5920	9	3	0	2
5921	12	2.4	0	2
5922	13	2.9	0	2
5923	3	1.5	0	2
5927	1	0.5	0	4
5930	4	2	0	2
5931	7	3.5	0	2
5932	6	3	0	2
5933	2	1	0	2
5934	5	2.5	0	2
5935	8	4	0	2
5936	2	1	0	2
5937	1	0.5	0	4
5938	10	5	0	2
5939	3	1.5	0	2
5940	4	2	0	2
5941	14	7	0	2
5942	5	2.5	0	2
5943	2	1	0	2
5944	3	1.5	0	2
5945	12	6	0	2
5946	6	3	0	2
5947	6	3	0	2
5948	4	2	0	2
5949	9	4.5	0	2
5950	2	1	0	2
5951	1	0.5	0	4
5952	9	4.5	0	2
5953	2	1	0	2
5954	2	1	0	2
5970	12	6	0	2
5976	5	2.5	0	2
5977	3	1.5	0	2
5978	2	1	0	2
5979	23	11.5	0	2
5980	10	5	0	2

5981	8	4	0	2
5982	33	16.5	0	0
5983	6	3	0	2
5984	2	1	0	2
5985	2	1	0	2
5986	2	1	0	2
5987	14	7	0	2
5988	31	15.5	0	2
5989	11	5.5	0	2
5990	26	13	0	2
5991	8	4	0	2
5992	2	1	0	2
5993	10	5	0	2
5994	18	9	0	2
5995	24	12	0	2
5996	5	2.5	0	2
5997	3	1.5	0	2
5998	4	2	0	2
6001	2	1	0	2
6003	2	1	0	2
6004	1	0.5	0	4
6011	1	0.2	0	4
6013	3	0.6	0	2
6015	1	0.2	0	4
6019	1	0.2	0	4
6022	9	2.1	0	2
6027	2	0.7	0	2
6028	3	0.9	0	2
6029	5	2.2	0	2
6032	8	4	0	2
6033	2	1	0	2
6034	1	0.5	0	4
6035	1	0.5	0	4
6036	7	3.5	0	2
6037	12	6	0	2
6040	1	0.5	0	4
6041	5	2.5	0	2
6042	1	0.5	0	4
6043	1	0.5	0	4
6044	1	0.5	0	4
6047	2	1	0	2
6051	1	0.5	0	4
6053	1	0.5	0	4
6055	3	1.5	0	2
6061	1	0.2	0	4
6063	2	1	0	2
6064	1	0.5	0	4
6065	8	4	0	2
6067	3	1.2	0	2
6073	1	0.5	0	4
6075	1	0.5	0	4
6076	1	0.5	0	4
6168	2	1	0	2

category=2, cleavage_site=1076
query=pto-miR180, target=Potri.008G225500.1,
score=4, range=1066-1085, strand=1

```
target  5' ACUUCAA-UUCaUGCAUGUAU 3'
              : ::::: ::::::::::.:
query   3' UAAAGUUCAAGUACGUACGUU 5'
```

>Potri.008G225500.1
#size=1183

300	1	0.333333333333333	0	4
327	2	0.666666666666667	0	2
335	1	0.333333333333333	0	4
339	2	0.666666666666667	0	2
359	1	0.333333333333333	0	4
360	1	0.333333333333333	0	4
427	8	2.66666666666667	0	2
436	2	0.666666666666667	0	2
452	5	1.66666666666667	0	2
478	1	0.333333333333333	0	4
498	1	0.333333333333333	0	4
512	2	0.666666666666667	0	2
517	6	2	0	2
525	1	0.1	0	4
543	1	0.333333333333333	0	4
547	2	0.666666666666667	0	2
548	1	0.333333333333333	0	4
550	2	0.666666666666667	0	2
560	1	0.333333333333333	0	4
710	1	0.166666666666667	0	4
810	3	0.75	0	2
920	1	0.333333333333333	0	4
953	1	0.166666666666667	0	4
959	1	0.111111111111111	0	4
963	1	0.111111111111111	0	4
968	1	0.333333333333333	0	4
975	1	0.333333333333333	0	4
977	1	0.333333333333333	0	4
979	1	0.333333333333333	0	4
996	2	1	0	2
998	2	1	0	2
1001	1	0.5	0	4
1002	1	0.5	0	4
1004	2	1	0	2
1006	1	0.5	0	4
1009	1	0.5	0	4
1010	2	1	0	2
1011	3	1.5	0	2
1012	2	1	0	2
1013	2	1	0	2
1014	1	0.5	0	4
1015	1	0.5	0	4
1018	1	0.5	0	4
1019	5	2.5	0	2
1020	5	2.5	0	2
1021	1	0.5	0	4
1022	1	0.5	0	4
1023	1	0.5	0	4
1024	3	1.5	0	2
1026	1	0.5	0	4

1027	2	1	0	2	
1028	1	0.5	0	4	
1030	1	0.5	0	4	
1032	1	0.5	0	4	
1033	4	2	0	2	
1035	1	0.5	0	4	
1036	2	1	0	2	
1037	1	0.5	0	4	
1040	1	0.5	0	4	
1041	4	2	0	2	
1043	3	1.5	0	2	
1044	1	0.5	0	4	
1047	1	0.5	0	4	
1048	1	0.5	0	4	
1049	1	0.5	0	4	
1051	1	0.5	0	4	
1053	1	0.5	0	4	
1072	3	1.5	0	2	
1073	1	0.5	0	4	
1075	5	2.5	0	2	
1076	4	2	0	2	<<<
1078	1	0.5	0	4	
1081	1	0.5	0	4	
1082	2	1	0	2	
1084	2	1	0	2	
1085	1	0.5	0	4	
1086	3	1.5	0	2	
1087	9	4.5	0	2	
1088	13	6.5	0	0	
1089	8	4	0	2	
1092	1	0.5	0	4	
1094	9	4.5	0	2	
1095	8	4	0	2	
1096	1	0.5	0	4	
1099	3	1.5	0	2	
1102	2	1	0	2	
1103	5	2.5	0	2	
1104	4	2	0	2	
1108	1	0.125	0	4	
1109	2	0.4	0	3	
1111	11	5.5	0	2	
1112	1	0.25	0	4	
1114	2	1	0	2	
1115	1	0.5	0	4	
1120	1	0.5	0	4	
1123	1	0.5	0	4	
1128	1	0.5	0	4	
1148	1	0.5	0	4	

---

category=2, cleavage_site=1076
query=pto-miR180, target=Potri.008G225800.1,
score=4, range=1066-1085, strand=1

```
target  5' ACUUCAA-UUCaUGCAUGUAU 3'
            : ::::: ::::::::::.:
query   3' UAAAGUUCAAGUACGUACGUU 5'
```

>Potri.008G225800.1
#size=1183

300	1	0.333333333333333	0	4

327	2	0.666666666666667	0	2
335	1	0.333333333333333	0	4
339	2	0.666666666666667	0	2
359	1	0.333333333333333	0	4
360	1	0.333333333333333	0	4
427	8	2.66666666666667	0	2
436	2	0.666666666666667	0	2
452	5	1.66666666666667	0	2
478	1	0.333333333333333	0	4
498	1	0.333333333333333	0	4
512	2	0.666666666666667	0	2
517	6	2	0	2
525	1	0.1	0	4
543	1	0.333333333333333	0	4
547	2	0.666666666666667	0	2
548	1	0.333333333333333	0	4
550	2	0.666666666666667	0	2
560	1	0.333333333333333	0	4
710	1	0.166666666666667	0	4
810	3	0.75	0	2
920	1	0.333333333333333	0	4
953	1	0.166666666666667	0	4
959	1	0.111111111111111	0	4
963	1	0.111111111111111	0	4
968	1	0.333333333333333	0	4
975	1	0.333333333333333	0	4
977	1	0.333333333333333	0	4
979	1	0.333333333333333	0	4
996	2	1	0	2
998	2	1	0	2
1001	1	0.5	0	4
1002	1	0.5	0	4
1004	2	1	0	2
1006	1	0.5	0	4
1009	1	0.5	0	4
1010	2	1	0	2
1011	3	1.5	0	2
1012	2	1	0	2
1013	2	1	0	2
1014	1	0.5	0	4
1015	1	0.5	0	4
1018	1	0.5	0	4
1019	5	2.5	0	2
1020	5	2.5	0	2
1021	1	0.5	0	4
1022	1	0.5	0	4
1023	1	0.5	0	4
1024	3	1.5	0	2
1026	1	0.5	0	4
1027	2	1	0	2
1028	1	0.5	0	4
1030	1	0.5	0	4
1032	1	0.5	0	4
1033	4	2	0	2
1035	1	0.5	0	4
1036	2	1	0	2
1037	1	0.5	0	4
1040	1	0.5	0	4

1041	4	2	0	2	
1043	3	1.5	0	2	
1044	1	0.5	0	4	
1047	1	0.5	0	4	
1048	1	0.5	0	4	
1049	1	0.5	0	4	
1051	1	0.5	0	4	
1053	1	0.5	0	4	
1072	3	1.5	0	2	
1073	1	0.5	0	4	
1075	5	2.5	0	2	
1076	4	2	0	2	<<<
1078	1	0.5	0	4	
1081	1	0.5	0	4	
1082	2	1	0	2	
1084	2	1	0	2	
1085	1	0.5	0	4	
1086	3	1.5	0	2	
1087	9	4.5	0	2	
1088	13	6.5	0	0	
1089	8	4	0	2	
1092	1	0.5	0	4	
1094	9	4.5	0	2	
1095	8	4	0	2	
1096	1	0.5	0	4	
1099	3	1.5	0	2	
1102	2	1	0	2	
1103	5	2.5	0	2	
1104	4	2	0	2	
1108	1	0.125	0	4	
1109	2	0.4	0	3	
1111	11	5.5	0	2	
1112	1	0.25	0	4	
1114	2	1	0	2	
1115	1	0.5	0	4	
1120	1	0.5	0	4	
1123	1	0.5	0	4	
1128	1	0.5	0	4	
1148	1	0.5	0	4	
